# Whole blood transcriptional signatures associated with rapid antidepressant response to ketamine in patients with treatment resistant depression

**DOI:** 10.1038/s41398-021-01712-0

**Published:** 2022-01-10

**Authors:** Flurin Cathomas, Laura Bevilacqua, Aarthi Ramakrishnan, Hope Kronman, Sara Costi, Molly Schneider, Kenny L. Chan, Long Li, Eric J. Nestler, Li Shen, Dennis S. Charney, Scott J. Russo, James W. Murrough

**Affiliations:** 1 Nash Family Department of Neuroscience and Friedman Brain Institute, Icahn School of Medicine at Mount Sinai, New York, NY 10029 USA; 2 Depression and Anxiety Center for Discovery and Treatment, Department of Psychiatry, Icahn School of Medicine of Mount Sinai, New York, NY 10029 USA; 3 Department of Pharmacology and Systems Therapeutics, Icahn School of Medicine at Mount Sinai, New York, NY USA

**Keywords:** Diagnostic markers, Molecular neuroscience

## Abstract

Ketamine has rapid and sustained antidepressant effects in patients with treatment-resistant depression (TRD). However, the underlying mechanisms of action are not well understood. There is increasing evidence that TRD is associated with a pro-inflammatory state and that ketamine may inhibit inflammatory processes. We thus investigated whole blood transcriptional profiles related to TRD and gene expression changes associated with treatment response to ketamine. Whole blood was collected at baseline (21 healthy controls [HC], 26 patients with TRD) and then again in patients with TRD 24 hours following a single intravenous infusion of ketamine (0.5 mg/kg). We performed RNA-sequencing and analyzed (a) baseline transcriptional profiles between patients with TRD and HC, (b) responders vs. non-responders before ketamine treatment, and (c) gene expression signatures associated with clinical improvement. At baseline, patients with TRD compared to HC showed a gene expression signature indicative of interferon signaling pathway activation. Prior to ketamine administration, the metabotropic glutamate receptor gene *GRM2* and the ionotropic glutamate receptor gene *GRIN2D* were upregulated in responders compared to non-responders. Response to ketamine was associated with a distinct transcriptional signature, however, we did not observe gene expression changes indicative of an anti-inflammatory effect. Future studies are needed to determine the role of the peripheral immune system in the antidepressant effect of ketamine.

## Introduction

Major depressive disorder (MDD) is a debilitating disorder with high individual morbidity and substantial socioeconomic burden [1]. Despite many psychopharmacological and psychotherapeutic treatment options available, approximately one third of patients do not show remission, even after multiple treatment attempts [2]. The most commonly used definition of treatment-resistance (treatment-resistant depression [TRD]) is based on clinical criteria and entails the failure to respond to at least two antidepressant trials with an adequate dose and duration [3, 4] Despite the high clinical relevance, little is known about the underlying etio-pathological mechanisms of TRD [5]. As a result, therapeutic options for patients with TRD are limited, and usually include increasing doses of conventional monoaminergic antidepressants, pharmacological augmentation strategies, and modalities of brain stimulation [6]. While these treatments can be effective, they are each accompanied by an increased risk of side effects [5]. Thus, there is a clear need to improve our understanding of the biology of TRD and identify novel treatment targets and potential blood biomarkers of treatment response at baseline.

Clinical trials have shown that a single administration of a sub-anaesthetic dose of ketamine can exert antidepressant effects as fast as a few hours after infusion, and last up to one week [7–11] While ketamine is clinically effective in patients who have failed to respond to conventional monoaminergic antidepressants, the underlying mechanisms of its actions are not well understood. In particular, the antidepressant effects of ketamine may not be entirely explained by its actions on neuronal N-methyl-D-aspartate receptors (NMDARs) and therefore investigating cellular and molecular targets of its mechanisms of action is warranted (for review [12]).

There is increasing evidence suggesting a bi-directional interaction between the peripheral immune system and brain relevant to the etio-pathology of neuropsychiatric disorders [13, 14] Indeed, it is well established that a subset of patients with MDD show chronic low-grade inflammation, e.g. indexed by increased pro-inflammatory cytokines in the blood (for meta-analysis: [15]). In addition, changes in peripheral cytokine levels have been associated with antidepressant treatment response in MDD [16, 17] and studies have shown that a pro-inflammatory state is associated with treatment resistance [18–20] Preclinical studies in rodents have indicated that the antidepressant like the effect of ketamine is associated with a decrease of cytokines. In mice, intraperitoneal injection of ketamine reduced chronic restraint stress-induced decreased sucrose preference paralleled by a decrease in serum interleukin (IL)-6, tumor necrosis factor (TNF)-α and IL-1β [21]. In rats, ketamine attenuated chronic unpredictable mild stress-induced behavioral changes and increases in hippocampal IL-6, TNF-α and IL-1β [22]. In humans, despite evidence linking a pro-inflammatory state to MDD in general and TRD in particular, only a few studies have investigated ketamine’s antidepressant effect on the peripheral immune system, yielding inconclusive results. In one study, IL-6 levels in blood were shown to be predictive of treatment response to ketamine, with responders showing increased serum levels of IL-6 compared to non-responders before ketamine treatment [23]. Conversely, Kiraly et al. reported that fibroblast growth factor 2, but not cytokine levels, were associated with treatment response [20]. This finding was in line with another study showing that cytokine changes did not correlate with changes in the mood nor did they predict ketamine-induced symptom improvements [24]. These studies, however, analyzed only a small subset of canonical immune markers and did not capture the extent of potential biological changes in the blood induced by ketamine. We therefore aimed to address this important question by conducting an exploratory study analyzing transcriptional differences at baseline between unmedicated TRD patients and healthy control (HC) subjects as well as changes associated with ketamine treatment response in the TRD group. We hypothesized that compared to HCs, patients with TRD will show a transcriptional signature in blood indicative of immune pathway activation at baseline and that this signature will be reversed by ketamine treatment.

We first compared whole blood transcriptional profiles between HC and patients with TRD at baseline prior to receiving ketamine treatment. We analyzed differently expressed genes, biological pathways, and performed weighted gene coexpression network analysis (WGCNA). In order to investigate pre-existing differences between ketamine responders and non-responders, we then compared transcriptional signatures between those groups at baseline, prior to treatment. Lastly, we investigated changes in genes and pathways from baseline to post-treatment that were associated with clinical improvement, as measured by the change in depression symptom severity.

## Methods

### Study participants and design

26 patients with MDD as assessed by the Structured Clinical Interview for the Diagnostic and Statistical Manual of Mental Disorders–Fourth Edition (SCID-IV) and Fifth Edition (SCID-5) [25, 26] were recruited through the Depression and Anxiety Center for Discovery and Treatment at the Icahn School of Medicine at Mount Sinai between 2012 and 2018. Subjects provided demographic information and underwent a psychiatric evaluation using the SCID-IV and SCID-5 conducted by trained study staff, which took place within 28 days before the ketamine infusion. All patients met diagnostic criteria for MDD in a current major depressive episode of at least moderate severity as measured by the Clinical Global Impression – Severity scale (CGI-S) [27]. Patients had a lifetime history of non-response to at least two trials of an antidepressant according to the Antidepressant Treatment History Form [3], and on average did not respond to 4.9 adequate trials (Table 1), therefore meeting the definition of TRD [28]. All participants were naïve to ketamine treatment. Depression severity at baseline (assessed on the morning prior to the ketamine infusion) and 24 hours post-infusion (post-treatment) was determined with the Montgomery-Åsberg Depression Rating Scale (MADRS) [29]. Participants in the TRD group received a single ketamine infusion (see below for details) and were defined as ketamine responders if they showed a ≥50% reduction in MADRS total score post-treatment compared to baseline, otherwise, they were classified as non-responders. Patients were excluded if they had a lifetime history of a psychotic disorder, bipolar disorder, alcohol or other substance abuse in the previous 6 months, any unstable medical condition, or any systemic inflammatory or autoimmune disease. Twenty-one HC were recruited via public advertisement. At the time of enrollment, all participants were free of antidepressant and anxiolytic medications and were free of medications known to affect the immune system for at least 2 weeks. Participants were free of active infections or systemic illness as confirmed by medical history, complete review of systems, physical exams, and laboratory testing. Participants were free of current substances of abuse as determined by history and by a urine toxicology test at the time of screening. Pregnant or nursing women were not included in the study. TRD and HC groups were matched on the variables of age, sex, and body mass index (BMI), as these variables are known to affect inflammatory markers.

**Table 1:** Sociodemographic and clinical data.

	HC (*N* = 21)	TRD (*N* = 26)	*p*- value (HC vs. TRD)	Non-responders (*N* = 8)	Responders (*N* = 18)	*p*- value (Responders vs. non-responders)
Age	39.2 ± 10.5	41.4 ± 12.2	0.49^d^	35.5 ± 10.7	44.1 ± 12.2	0.10^c^
Sex (M/F)	11/10	12/14	0.67^e^	3/5	9/9	0.56^e^
BMI	26.0 ± 4.8	26.7 ± 5.5	0.61^d^	25.1 ± 4.2	27.4 ± 6.0	0.34^c^
Ethnicity			**0.01**^e^			0.62^e^
Caucasian	9 (43%)	21 (81%)		6 (75%)	15 (83%)	
African-American	7 (33%)	1 (4%)		0	1 (6%)	
Other	5 (24%)	4 (15%)		2 (25%)	2 (11%)	
Age of illness onset		16.7 ± 11.0		16.5 ± 7.2	16.9 ± 12.6	0.61^d^
Recurrent illness (Y/N)		17/9		6/2	11/7	0.49^e^
Chronic episode (Y/N)^a^		17/9		6/2	11/7	0.49^e^
Psychiatric hospitalizations (Y/N)		9/17		3/5	6/12	0.84^e^
Suicide attempts (Y/N)		5/21		0/8	5/13	0.39^e^
Number of failed ADT		4.9 ± 2.5		5.9 ± 2.6	4.4 ± 2.4	0.18^c^
Psychiatric comorbidities (Y/N)^b^		9/17		2/6	7/11	0.49^e^
MADRS (baseline)		31.3 ± 5.7		30.6 ± 7.7	31.6 ± 4.8	0.71^c^

Significant *p*-values of <0.05 are indicated in bold.

*ADT* antidepressant treatment (lifetime), *BMI* body mass index, *MADRS* Montgomery-Åsberg Depression Rating Scale, *TRD* treatment-resistant depression.

^a^Chronic episode was defined as >2 years.

^b^Including any comorbidities identified with the Statistical Manual of Mental Disorders–Fourth and Fifth Edition (SCID). Data are presented as mean ± standard deviation.

Statistics: ^c^Student’s *t*-test.

^d^Mann–Whitney *U* test.

^e^Chi-square test.

The Program for the Protection of Human subjects at Mount Sinai approved the protocol, consent forms and all study procedures. Each participant was originally enrolled in only one of the following ketamine studies registered on clinicaltrials.gov: NCT01507181, NCT00548964, NCT01880593, NCT03102736. Participants were provided a thorough description of each study, and written informed consent was obtained prior to any study procedures being performed. Participants also provided informed consent to have blood samples and psychosocial data collected, which were analyzed in the present study.

### Ketamine infusion and blood draw

Blood draw of all eligible patients with TRD was performed immediately prior to receiving a single ketamine infusion (0.5 mg/kg over 40 min). Participants were monitored continuously by study staff throughout the infusion and for at least 60 minutes post-infusion with a three-lead electrocardiography (ECG), pulse oximetry, and repeated non-invasive blood pressure measurements. Patients were assessed for side effects by a study clinician. TRD participants returned to the clinic after 24 h following the infusion to assess the antidepressant effects of ketamine, undergo a safety assessment, and a 24-h post infusion blood draw. HC underwent a similar screening procedure, and on the day of testing received a baseline blood draw. Blood was drawn into PAXgene tubes (PAXgene Blood RNA Tubes, Qiagen). PAXgene tubes were immediately frozen and stored at −80 °C. All participants were instructed to fast for a minimum of 8 h before the blood draw.

### RNA extraction and library preparation

RNA was extracted using the PAXgene Blood RNA Kit (Qiagen) according to the manufacturer’s instructions. RNA quality, RNA integrity number (RIN), and RNA concentrations were assessed using Nanodrop (Thermo Fischer Scientific) and Bionalyzer (Agilent RNA 6000 Nano Kit). 750 ng of purified RNA was used for library preparation, which was performed using the TruSeq Stranded mRNA kit (Illumina). Libraries were barcoded for multiplexing. Before sequencing, library quality and concentration were measured using Bioanalyzer (Agilent RNA 6000 Nano Kit). Libraries were sequenced (2 × 150 base pair, paired-end reads configuration, v4 chemistry) on an Illumina HiSeq machine at a minimum of 40 million reads per sample. Sequencing was performed at Genewiz.

### RNA-sequencing data processing and differential expression analysis

We first performed quality control using FastQC [30]. The script Trim Galore! was used to trim adapter sequences, and raw RNA-sequencing reads from the human genome were aligned to hg38 Ensembl v90 using HISAT2 [31, 32] Counts of reads mapped to genes were obtained using HTSeq [33]. The threshold for filtering out low expressed genes was set to at least 5 reads across 60% of the samples. variancePartition package in R was used to compute the percentage of variance explained by each covariate within the data set. Differential gene expression analysis was carried out using DESeq2 [34]. Models for differential expression analysis were adjusted for age, RIN, gender, BMI, and ethnicity, based on the results of variancePartition [35]. Significance was set at an uncorrected *p* < 0.05 for broad pattern identification. A fold change (FC) threshold was set at 1.3 (i.e., log2 FC, |LFC| > 0.3785) for each comparison. Comparison groups included TRD (*N* = 26) vs. HC (*N* = 21) at baseline, TRD responders (*N* = 18) vs. non responders (*N* = 8) at baseline (pre-ketamine infusion) and finally TRD post- vs. pre-ketamine infusion (*N* = 21). To identify genes associated with clinical improvement, i.e., change in MADRS score, we computed a regression coefficient using DESeq2. MADRS score and subject ID were added as a covariate to the DESeq2 model, along with controlling for the covariates age, gender, BMI, ethnicity, and RIN score, as described above. For all the analyses (computation of differentially expressed genes between patients with TRD and HC, responders vs. non-responders before ketamine treatment and gene expression signatures associated with clinical improvement) 19,175 genes were tested. The lists of all DEG meeting significance thresholds are available in supplementary file 1. Sequencing data are available on the NCBI GEO website (accession code: GSE185855).

### Weighted gene co-expression network analysis (WGCNA)

WGCNA analysis was performed according to [36]. 19,175 genes were included in the WGCNA. We utilized the normalized and variance stabilizing transformation expression matrix of MDD patients from which the effects of gender, age, BMI, ethnicity, and RIN scores were regressed out using limma [37]. An unsigned co-expression network was set up using a co-expression similarity matrix. The similarity matrix was generated using the absolute values of the correlation coefficient between expression profiles of pairs of genes. Adjacency matrix was derived from the co-expression similarity matrix by raising all values of the matrix to a power of 7. This matrix provided the measure of network interconnectedness for pairs of genes. Topological overlap matrix (TOM) was generated using the transformed adjacency matrix, and average linkage hierarchical clustering was applied to find modules. To summarize the expression profile of genes in each module, module eigengene was calculated using the first principal component of each module. A total of 11 modules were identified (a complete list of genes per module is provided in supplementary file 2). To correlate eigengenes of each module with clinical features, Pearson correlation coefficients were computed. In total, eigengenes (one eigengene per module, 11 eigengenes in total) were correlated with seven clinical features: age of illness onset, chronicity of illness (current episode lasting >2 yrs) (Yes/No), recurrence of major depressive episodes (Yes/No), psychiatric comorbidities (Yes/No), psychiatric hospitalizations (Yes/No), suicide attempts (Yes/No), and baseline MADRS score.

### Module differential connectivity analysis (MDC)

Module differential connectivity (MDC) analysis was used to identify modules in the co-expression network that were unique to TRD patients in comparison to control subjects. MDC was carried out using modules identified from the WGCNA analysis for TRD and control subjects. An MDC score of >1 indicated gain of connectivity while an MDC score of <1 indicated loss of connectivity.

### Ingenuity pathway and upstream regulator analysis

Biological pathways and upstream regulators were identified using Ingenuity Pathway Analysis (IPA, QIAGEN Inc., v. 49932394) and functional annotation for gene ontology (GO) of biological processes was performed using David Bioinformatics Resource 6.8 [38, 39]. We performed pathway, upstream regulator, and GO analysis for all the three lists of DEGs. In total, we included 560 DEGs for the TRD vs. HC comparison at baseline, 331 DEGs for the comparison between ketamine responders vs. non-responders at baseline and 464 genes when investigating transcriptional signatures associated with clinical improvement after ketamine treatment. These genes met the significance threshold of FC >or <1.3 and *p* < 0.05 (see supplementary file 1). The following cutoffs were used: For IPA pathway analysis: pathways that were significantly (*p* < 0.05) upregulated (z-score > 0) or downregulated (z-score < 0). For upstream analysis: *p* < 0.05, |z-score| > 2 and upstream regulators were filtered for genes, RNAs, and proteins. For GO analyses: GO terms with >5 genes and *p* < 0.05. All *p*-values for IPA and GO were computed using Fisher’s exact tests.

### Statistical analysis of demographic data and clinical characteristics

Demographic data and clinical characteristics were analyzed using summary statistics: normally distributed data (as assessed by the Shapiro–Wilk test) were analyzed with two-tailed Student’s *t*-test, non-normally distributed data were analyzed with the Mann–Whitney *U* test and the Chi-Square test was applied for categorical variables (SPSS version 24 (IBM Corp., SPSS Inc., Chicago IL, USA)). The level of statistical significance was set at *p* < 0.05. Due to the exploratory nature of the study, we did not correct for multiple testing.

## Results

### Sociodemographic and clinical characteristics

Sociodemographic and clinical variables are presented in Table 1. HC and patients with TRD did not differ in age, gender, and BMI but differed in ethnicity. 24 h after ketamine infusion, 67% (*N* = 18) of participants with TRD showed a clinical improvement of ≥50% on the MADRS, and were therefore classified as responders, while the rest (*N* = 8) did not show significant clinical improvement (MADRS change <50%), and were therefore classified as non-responders. Ketamine responders and non-responders did not differ in sociodemographic characteristics or baseline clinical features.

### Differential gene expression between TRD and HC at baseline reveals activation of the interferon signaling pathway

**Fig. 1:**
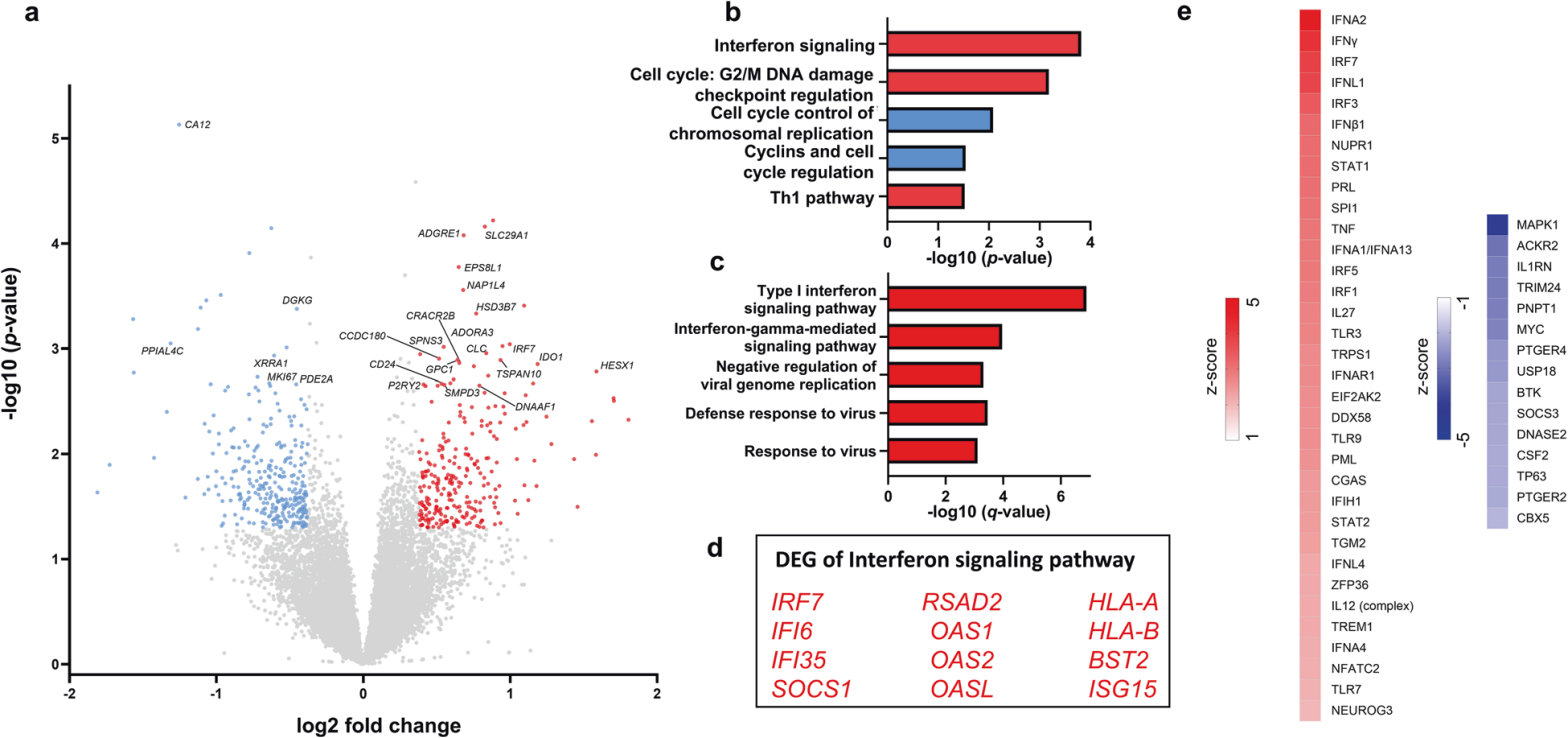
Transcriptional changes in patients with treatment-resistant depression (TRD) compared to healthy controls (HC). **a** Volcano plot of all 19175 genes; significantly (*p* < 0.05) differentially expressed genes (fold change > or <1.3) are indicated in red (upregulated), or blue (downregulated). Gene names are indicated for the top 25 differentially expressed protein-coding genes. **b** Pathways enriched in TRD vs. HC subjects (red = positive z-score, blue = negative z-score) identified by Ingenuity Pathway Analyzer (IPA). **c** 5 most significant gene ontology (GO) terms for upregulated genes enriched in TRD vs. HC. No significant GO terms in downregulated genes. **d** Genes enriched in Interferon signaling pathway combined from both IPA and GO (red indicates upregulated in TRD vs. HC). **e** Predicted upstream regulators using IPA (red = predicted activation, blue = predicted inhibition).

We first investigated differences in transcriptional signatures in whole blood at baseline between patients with TRD and HC. In total, 560 genes were differently expressed (FC: > or <1.3 and *p* < 0.05): 262 genes were up- and 298 genes downregulated. The top 25 differentially expressed protein-coding genes are shown in a volcano plot (Fig. 1a). With the genes that were differentially expressed between groups, we then performed pathway analysis and gene ontology analysis using IPA [40] and gene ontology [38], respectively. Type I interferon signaling was the biological pathway that was most significantly enriched in DEGs between TRD patients and HC (Fig. 1b, c) and IPA indicated that this pathway was activated in TRD patients vs. HC. This finding is in line with a recent study that showed an association of recurrent MDD with increased expression of interferon signaling pathway genes in whole blood [41]. Comparing DEGs of the Interferon signaling pathway (Fig. 1d) between the two studies revealed upregulation of *IRF7, IFI6, IFI35, SOCS1, OAS1, OAS2, OASL, HLA-A, HLA-B*, and *ISG15* in both study populations, resulting in an overlap of more than 80% of the pathway associated genes. We then identified several upstream regulators using IPA (Fig. 1e). Interestingly, a large number of the upstream regulators that were predicted to be activated were genes associated with interferon signaling, further indicating the importance of interferon signaling in TRD patients vs. HC at baseline (pre-treatment). In addition, using weighted coexpression network analysis (WGCNA), we identified several modules that were correlated with clinical features and associated with biological pathways indicative of immune-inflammatory processes (Fig. S1a). The yellow module significantly correlated with disease recurrence, as a clinical descriptor of illness course, and genes of the module were enriched for the interferon signaling pathway (Fig. S1b and Table S1). Using modular differential connectivity (MDC) metrics, we identified the blue (*p* = 0.02) and pink (*p* = 0.04) modules with a significant gain of connectivity in the TRD group. Pathways involved in the cell cycle, DNA replication, recombination, and repair and translation, as well as those involved in the classical complement pathway, were associated with the blue and pink modules, respectively.

### Glutamate receptor genes are upregulated in ketamine responders vs. non-responders at baseline

**Fig. 2:**
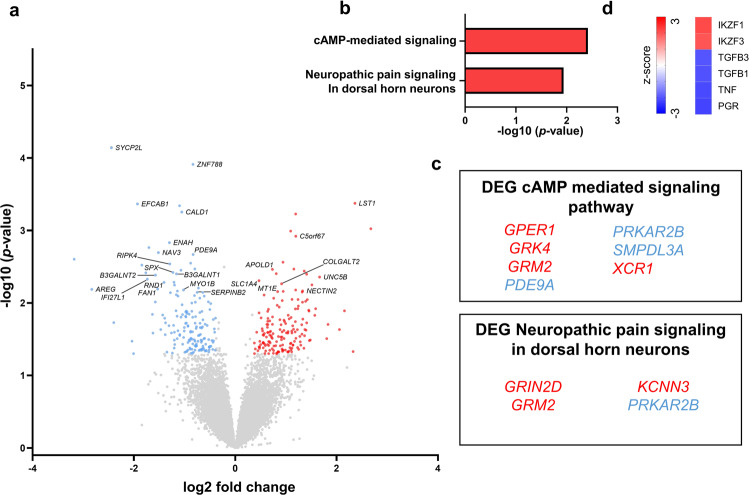
Transcriptional signatures of ketamine responders vs. non-responders at baseline. **a** Volcano plots showing significantly (*p* < 0.05, fold change > or <1.3) differentially expressed genes (DEG) [red = upregulated and blue = downregulated]. Gene names are indicated for the top 25 differentially expressed protein-coding genes. **b** Ingenuity Pathway Analysis (IPA) identified two biological pathways (red = activation) enriched at baseline in responders vs. non-responders. **c** DEGs within each pathway. **d** Predicted upstream regulators identified by IPA (red indicates predicted activation, blue indicates predicted inhibition).

In order to assess differences between transcriptional signatures in patients responding to ketamine and those that did not show a clinical improvement, we first compared the DEGs of the two groups before they received treatment with ketamine. Compared to non-responders, responders showed 331 differentially expressed genes; 166 were up- and 165 were downregulated (Fig. 2a). Two pathways were activated in responders compared to non-responders at baseline: cAMP-mediated signaling and neuropathic pain signaling in dorsal horn neurons (Fig. 2b). Moreover, two genes of these pathways involved in glutamate signaling (the metabotropic glutamate receptor gene *GRM2* and the ionotropic glutamate receptor gene *GRIN2D*) were enriched in responders compared to non-responders (Fig. 2c). An interesting finding, given ketamine’s action as an NMDA receptor modulator [12]. Upstream regulator analysis identified activation of the transcription factors IKZF3 and IKZF1 and inhibition of TGFB1/3, TNF, and PGR in responders compared to non-responders (Fig. 2d).

### Transcriptional signatures associated with clinical improvement after ketamine treatment

**Fig. 3:**
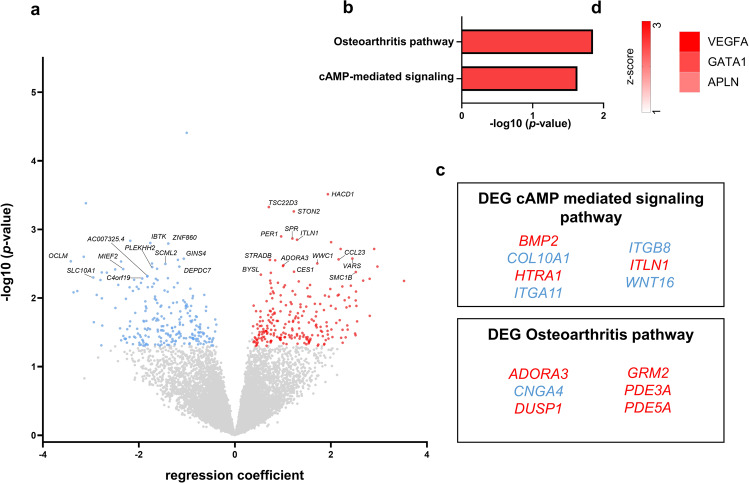
Genes, biological pathways, and transcription factors associated with ketamine treatment response. **a** Volcano plot showing the top 25 protein-coding genes associated with ketamine treatment response. Positive regression coefficient (red) indicates decreased gene expression with clinical improvements (i.e., higher delta MADRS), negative regression coefficient (blue) indicates that clinical improvement is associated with increased gene expression. **b** Ingenuity Pathway Analyzer (IPA) identified several pathways associated with ketamine treatment response (red = positive z-score)). **c** Genes enriched in the top two pathways (red = positive regression coefficient, blue = negative regression coefficient). **d** Predicted upstream regulators using Ingenuity Pathway Analysis (red = predicted activation).

Lastly, we investigated genes that were associated with clinical response to ketamine treatment by computing a regression coefficient between the change in MADRS score vs. change in gene expression. Positive regression coefficient indicates downregulation of gene expression with clinical improvements (i.e., higher delta MADRS), negative regression coefficients indicate that clinical improvement is associated with upregulation of gene expression. Overall, of 464 significant DEGs, 233 genes showed positive and 231 showed negative regression coefficients (Fig. 3a). IPA revealed the *cAMP signaling* and the *osteoarthritis* pathways to be associated with treatment response (Fig. 3b, c). Upstream regulator analysis showed activation of VEGFA, GATA1 and APLN (Fig. 3d). In contrast to our initial hypothesis, treatment response was not associated with an anti-inflammatory gene expression signature or an effect on the interferon pathway.

## Discussion

In the current study we investigated transcriptional changes in whole blood associated with treatment response to ketamine. In a cohort of unmedicated subjects, we show that, compared to HC, patients with TRD are characterized by an activation of the interferon signaling pathway. Prior to ketamine administration, two genes involved in glutamate signaling, the metabotropic glutamate receptor gene *GRM2* and the ionotropic glutamate receptor gene *GRIN2D*, are enriched in ketamine responders vs. non-responders, indicating that these receptors might play a role in the response to ketamine. Response to ketamine was associated with a distinct transcriptional signature in whole blood, however, we did not observe gene expression changes indicative of an anti-inflammatory effect.

To our knowledge, this is the first study utilizing an unbiased transcriptomic approach to investigate rapid antidepressant effects of ketamine on whole blood gene expression signatures in a sample of medication-free patients with TRD. A few studies have investigated transcriptional signatures between HC and MDD patients at baseline using microarray techniques [42–46] and RNA-sequencing [41]. Given the large sample size and rigorous control for multiple covariates, comparing the present findings with the study by Mostavi et al. is of particular relevance [41]. Both studies report an association of MDD and TRD with activation of the interferon signaling pathway. Comparing specific differentially expressed genes of the Interferon signaling pathway between the two studies reveals an overlap of more than 80% of the pathway-associated genes. We hereby not only replicate the potential importance of this pathway, but this also may indicate that our relatively small sample is representative of the larger MDD population.

Interferons and interferon-associated molecules are versatile modulators of the immune system [47]. They coordinate both innate and adaptive immune responses by activating macrophages and natural killer cells, up-regulating major histocompatibility complex (MHC) antigen presentation proteins, and promoting B cell immunoglobulin class switching [48]. As such, they play an important role in protection against pathogens such as viruses but have also been associated with autoimmune disorders and autoinflammation [49]. Moreover, one of the first observations linking immune activation and clinically relevant depression stems from patients with hepatitis C treated with interferon-α that developed severe depressive symptoms as a direct consequence of the interferon treatment [50, 51]. In summary, at baseline and compared to HC, patients with TRD show a pro-inflammatory transcriptional signature characterized by an activation of the interferon signaling pathway.

Comparing transcriptional signatures between responders and non-responders at baseline identified the upregulation of the metabotropic glutamate receptor gene *GRM2* and the ionotropic glutamate receptor gene *GRIN2D* in blood. Both receptors have been previously implicated in ketamine’s antidepressant mechanism of action [52, 53]. The precise mechanisms underlying ketamine’s antidepressant response is still largely unknown, but studies have implicated NMDAR antagonism, glutamate surge, and α-amino-3-hydroxy-5-methyl-4-isoxazole propionic acid (AMPA) receptor activation as candidate mechanisms, among others [54]. Preclinical data provide evidence that ketamine and its metabolites may enhance synaptic activity via a glutamate-associated pathway [12] that is modulated by the actions of group II metabotropic glutamate receptors subtypes 2 and 3 (mGlu2/3), encoded by *GRM2* [12]. Less data are available regarding the relationship between *GRIN2D* and ketamine. The *GRIN2D* gene encodes for the 2D subunit of the NMDAR receptor (GluN2D), which is highly expressed in forebrain inhibitory interneurons [55] and exhibits high affinity for ketamine [56]. It has been suggested that inhibition of GABAergic inhibitory interneurons by NMDAR may lead to the dysinhibition of glutamatergic pyramidal cells and an increase in excitatory glutamatergic neurotransmission with downstream effects on protein synthesis that could play a role in mood regulation [12]. Human evidence that ketamine can act on this subunit comes from a case report investigating a mutation in GRIN2D in a child with refractory status epilepticus reporting a dramatic electrical and clinical improvement upon treatment with ketamine [53]. Additional studies investigating the role of GRIN2D in the context of TRD and its implication for ketamine antidepressant effect are needed.

Given the evidence that whole blood shares significant gene expression signatures with multiple central nervous system (CNS) tissues [57–59], it can be speculated that the observed changes seen in glutamate receptor genes might reflect changes in the CNS.

Further, these data indicate the potential for using whole blood transcriptomics to identify reliable markers of antidepressant response to ketamine [20, 23, 60, 61]. The availability of an accurate biomarker to predict which patients will respond to ketamine will be important to facilitate a shift towards precision medicine approaches to treat MDD. The ability to select a treatment tailored to the individual, which is particularly relevant for treatment-refractory patients, will reduce risk for suicidality and poor clinical outcomes [62]. Of interest, biological factors associated with treatment response may represent a combination of treatment-specific factors (factors that are mechanistically related to the benefit of the treatment) and non-specific factors (that are associated with the placebo response). In the current population of TRD patients, it is possible that their response to nonspecific factors is minimized. In addition, non-response to ketamine may suggest that the pathogenesis of depression in those patients might involve systems that are not affected by ketamine. Therefore, further studies in TRD populations are needed.

Finally, considering the small sample size, treatment response to ketamine appears to be associated with a distinct gene expression signature in blood but not with a large anti-inflammatory effect. In contrast to our initial hypothesis, the current study does not provide evidence that clinical improvement after ketamine treatment at 24 h post-infusion is associated with an anti-inflammatory signature in general or an attenuation of genes related to the interferon pathway. A small or moderate anti-inflammatory effect could be present, but, given the limited power, not detected with the current sample size.

To the best of our knowledge, no study has thus far investigated such transcriptional changes in whole blood associated with ketamine treatment response in depression. A few prior studies have so far investigated the association between treatment response to ketamine in patients with MDD and alterations in cytokines in blood, with inconclusive results. A study by Yang et al. demonstrated an association of treatment response with downregulation of the pro-inflammatory cytokines IL-1β and TNF-α [23]. Kiraly et al. have shown an anti-inflammatory effect of ketamine, but no association with improvement of depressive symptoms [20], while Park et al. did not find an association between ketamine treatment response and cytokine levels [24]. While both of these studies are in line with the current results, future studies are needed.

There are strengths and limitations of the study that should be considered. The major strength is the well-characterized sample of patients with TRD free of any medication and the unbiased transcriptional approach performed within subjects. However, the sample size was relatively small so the nature of the study is exploratory. In addition, we did not include a HC group receiving ketamine treatment and thus did not control for potential physiological fluctuations in transcriptional profiles over time. We also cannot exclude that some of the changes observed in the responders group are due to the placebo effect. The analysis of whole blood does not provide any information about the cell types involved in the reported biological processes and might obscure smaller biologically relevant changes in immune cells with low frequencies. Cell type-specific transcriptional analyses are warranted to gain a more detailed understanding of the cellular processes involved. The current study has been performed in circulation and does not provide any information on transcriptional changes in the central nervous system. Finally, there is an under-representation of ethnic diversity in our sample.

In summary, the results of the current study indicate that patients with TRD, compared to HCs, show a gene expression signature indicative of interferon signaling pathway activation. Prior to ketamine administration, the metabotropic glutamate receptor gene *GRM2* and the ionotropic glutamate receptor gene *GRIN2D* were upregulated in ketamine responders compared to non-responders. Finally, treatment response to ketamine is associated with a distinct gene expression signature in blood but not with an anti-inflammatory effect.

## Supplementary information

Supplementary Material

Figure S1. Weighted gene co-expression analysis (WGCNA).

Supplementary file 1

Supplementary file 2
