# Supplementary material for: Whole blood transcriptional signatures associated with rapid antidepressant response to ketamine in patients with treatment resistant depression
Source: Transl Psychiatry. 2022 Jan 10;12:12. doi: 10.1038/s41398-021-01712-0 (PMC8748646; doi:10.1038/s41398-021-01712-0)
Supplement: Supplementary file 1 — Supplementary Material [file 41398_2021_1712_MOESM1_ESM.docx]

**Supplementary Material**

**Table S1. Biological pathways associated with modules and clinical features.** Genes belonging to modules with a significant correlation with clinical characteristics were analyzed using Ingenuity Pathway Analyzer (IPA) for biological pathway analysis and Gene Ontology (GO). The biological pathways identified are reported in the table along with Pearson’s R correlation and *p*-values. ^1^ Performing IPA was not possible due to the large number of genes (> 8000 genes). ^2^no significant gene ontology term.

**Supplementary file 1.** List of differentially expressed genes, meeting significance threshold of fold change > or < 1.3 and *p* < 0.05, at baseline and pre and post ketamine treatment.

**Supplementary file 2.** List of genes per module identified with weighted gene co-expression analysis (WCGNA).

| **Modules** | **Pearson’s R (*p*-value)** | **Clinical features** | **Biological pathways** | **Gene ontology terms** |
| --- | --- | --- | --- | --- |
| Brown | 0.58  (0.002) | Age of onset | - nNOS signaling in skeletal muscle cells  - Calcium signaling  - Cellular effects of sildenafil  - Phenylethylamine degradation I  - Serine biosynthesis | - transcription, DNA-template  - cilium movement  - mitochondrial translational elongation  - mitochondrial translational termination  - regulation of protein kinase activity |
| Green | 0.58  (0.002) | Recurrent illness | - DNA double-strand break repair by homologous recombination  - Pyrimidine ribonucleotides de novo biosynthesis  - Granzyme B signaling  - T cell receptor signaling  - Ovarian cancer signaling | - beta-catenin-TCF complex assembly  - T-cell costimulation  - negative regulation of transcription, DNA-template  - regulation of cellular response to heat  - mitochondrion organization |
| Red | −0.47  (0.02) | Psychiatric hospitali-zations | - Iron homeostasis signaling pathway  - Heme biosynthesis II  - Tetrapyrrole biosynthesis II  - Clathrin-mediated endocytosis signaling  - Caveolar-mediated endocytosis signaling | - oxygen transport  - bicarbonate transport  - erythrocyte differentiation  - blood coagulation  - transforming growth factor beta receptor signaling pathway |
| Black | −0.44  (0.02) | Age of onset | - Cytotoxic T lymphocyte-mediated apoptosis of target cells  - Tumoricidal function of hepatic natural killer cells  - Eicosanoid signaling  - Natural killer cell signaling  - Crosstalk between dendritic cells and natural killer cells | - cell surface receptor signaling pathway  - apoptotic process  - regulation of immune response  - cell adhesion  - G-protein coupled receptor signaling pathway |
| Magenta | −0.43  (0.03) | Age of onset | - Type II diabetes mellitus signaling  - FcγRIIB signaling in B lymphocytes  - IL-15 signaling  - Sorbitol degradation I  - Histamine biosynthesis | -^2^ |
| Turquoise | −0.41  (0.04) | Psychiatric comor-  bidities | -^1^ | - cell division  - protein phosphorylation  - protein transport  - transcription, DNA-template  - mitotic nuclear division |
| Yellow | −0.45  (0.02) | Recurrent illness | - Interferon signaling  - TREM1 signaling  - Role of pattern recognition receptors in recognition of bacteria and viruses  - Prolactin signaling  - Role of macrophages, fibroblasts and endothelial cells in rheumatoid arthritis | - type I interferon signaling pathway  - defense response to virus  - negative regulation of viral genome replication  - interferon-gamma-mediated signaling pathway |
| Grey | −0.4  (0.04) | Psychiatric hospitali-zations | - Bile acid biosynthesis, neutral pathway  - γ-linolenate Biosynthesis II  - Glycine degradation (creatine biosynthesis)  - Cysteine biosynthesis/ homocysteine degradation  - HIPPO signaling | - Cell-cell signaling  - signal transduction |
| Grey | −0.46  (0.02) | Suicide attempts |  |  |

**Table S1**
