# Supplementary material for: Whole blood transcriptional signatures associated with rapid antidepressant response to ketamine in patients with treatment resistant depression
Source: Transl Psychiatry. 2022 Jan 10;12:12. doi: 10.1038/s41398-021-01712-0 (PMC8748646; doi:10.1038/s41398-021-01712-0)

Figure S1. Weighted gene co-expression analysis (WGCNA). a) Cluster dendrogram showing how genes with similar expression levels cluster together. 11 modules were identified and assigned different colors. **b)** Heat map showing correlations between individual modules and clinical characteristics of depression measured in the TRD sample. The clinical features included are: age of illness onset, chronicity of illness (current episode lasting > 2yrs) (Yes/No), recurrence of major depressive episodes (Yes/No), psychiatric comorbidities (Yes/No), psychiatric hospitalizations (Yes/No), suicide attempts (Yes/No), baseline MADRS score. On the Y axis WGCNA modules by color (numbers in parenthesis indicating the number of genes per each module). * $p < 0.01$, *** $p < 0.001$.

Fig. S1

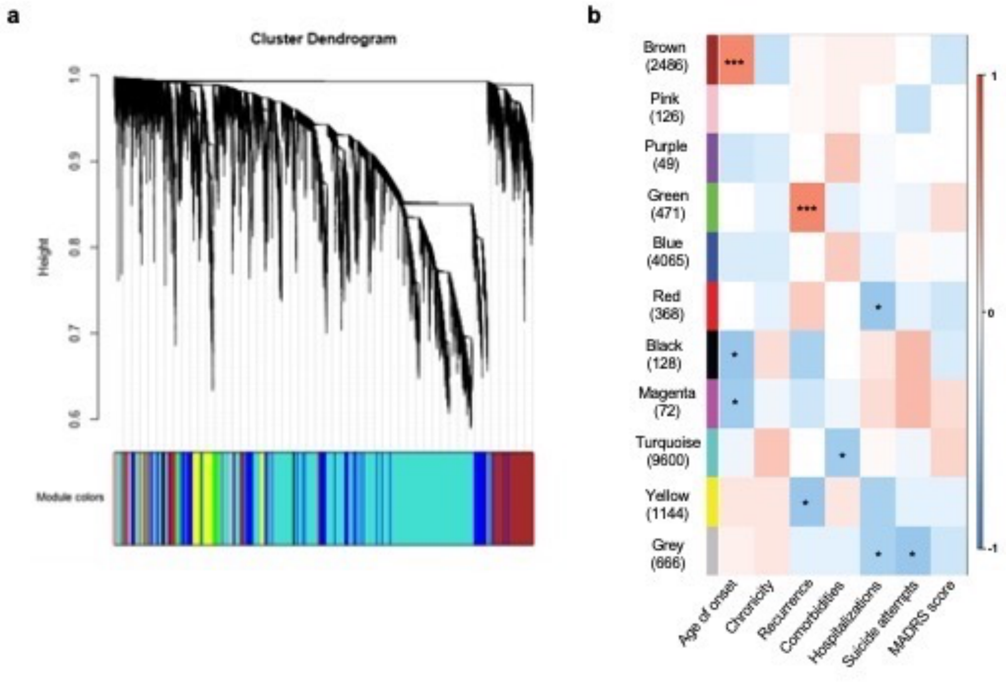

Supplement: Supplementary file 2 — Figure S1. Weighted gene co-expression analysis (WGCNA). [file 41398_2021_1712_MOESM2_ESM.pdf]
