# Supplementary material for: Whole blood transcriptional signatures associated with rapid antidepressant response to ketamine in patients with treatment resistant depression
Source: Transl Psychiatry. 2022 Jan 10;12:12. doi: 10.1038/s41398-021-01712-0 (PMC8748646; doi:10.1038/s41398-021-01712-0)
Supplement: Supplementary file 3 — Supplementary file 1 [file 41398_2021_1712_MOESM3_ESM.pdf]

Baseline TRD vs. HC

Gene ID	Gene Name	Base Mean	log2 Fold Change	p-value
ENSG00000200087	SNORA73B	219.1423864	-1.809810018	0.023210495
ENSG00000230076	RPL10P6	214.1557388	-1.726692752	0.012672226
ENSG00000255893	AP000786.1	22.18367837	-1.566781604	0.000524284
ENSG00000234449	FAM239A	72.21721869	-1.562589644	0.001690464
ENSG00000236242	MYO16-AS1	20.61983994	-1.424713658	0.010893509
ENSG00000166831	RBPMS2	84.70977461	-1.337156461	0.003962405
ENSG00000203801	LINC00222	8.415550844	-1.311293366	0.000895148
ENSG00000074410	CA12	10.44025701	-1.253624432	7.40E-06
ENSG00000152463	OLAH	26.35822973	-1.210426178	0.025906653
ENSG00000146122	DAAM2	302.886716	-1.126446667	0.017244073
ENSG00000263812	LINC00908	19.48099635	-1.125078673	0.000652244
ENSG00000261055	AL450468.2	10.8982335	-1.107721711	0.000408472
ENSG00000249835	VCAN-AS1	15.99315303	-1.082223821	0.024110982
ENSG00000270550	IGHV3-30	16.53265346	-1.080316644	0.005153374
ENSG00000200204	RNU1-22P	16.62289176	-1.06795971	0.000348428
ENSG00000120156	TEK	51.41195849	-1.058935072	0.016442274
ENSG00000180535	BHLHA15	15.36704491	-1.053413323	0.021768301
ENSG00000268199	AC010335.1	11.14075695	-1.048386715	0.006103586
ENSG00000263464	PPIAL4C	13.04508252	-1.039408331	0.002167989
ENSG00000163431	LMOD1	9.712884618	-1.02430935	0.009896394
ENSG00000041515	MYO16	16.06993399	-1.01909738	0.004287959
ENSG00000162594	IL23R	17.41668059	-1.003024632	0.020217058
ENSG00000211941	IGHV3-11	35.68728756	-0.999487705	0.014592987
ENSG00000175899	A2M	97.53062019	-0.985657697	0.024425453
ENSG00000100196	KDELR3	16.06308234	-0.983596587	0.008094145
ENSG00000136848	DAB2IP	59.5480675	-0.982717489	0.00638941
ENSG00000237879	LINC00398	14.96821655	-0.970428111	0.000309388
ENSG00000260075	AC217777.1	22.01639966	-0.964356602	0.048198156
ENSG00000154165	GPR15	201.4589046	-0.960109106	0.016839731
ENSG00000178445	GLDC	48.02053385	-0.958597553	0.028000937
ENSG00000188056	TREML4	493.3974375	-0.954851194	0.04567625
ENSG00000125845	BMP2	13.49874097	-0.943183416	0.038579631
ENSG00000169607	CKAP2L	17.78610963	-0.940087945	0.002492497
ENSG00000236679	RPL23AP24	7.908786872	-0.93903062	0.019045613
ENSG00000103723	AP3B2	33.28553566	-0.932019894	0.009775213
ENSG00000187166	H1FNT	15.88091142	-0.920786805	0.002299393
ENSG00000224207	AC018797.1	9.644814559	-0.903397298	0.027709692
ENSG00000230359	TPI1P2	9.244508009	-0.899652109	0.005966758
ENSG00000222365	SNORD12B	30.08387554	-0.897099623	0.017135435
ENSG00000129354	AP1M2	127.7561617	-0.892311049	0.008597878
ENSG00000165125	TRPV6	10.54192128	-0.88342858	0.020472731
ENSG00000126838	PZP	95.24037637	-0.882973701	0.005686007
ENSG00000279164	AL118508.3	22.69716711	-0.882922222	0.014678447
ENSG00000249160	LINC02213	11.55395099	-0.880575367	0.008690999

ENSG00000181800	CELF2-AS1	223.2131479	-0.872927033	0.037787253
ENSG00000215267	AKR1C7P	13.18642218	-0.865808458	0.014427485
ENSG00000130202	NECTIN2	657.164553	-0.859157295	0.046656204
ENSG00000201441	RNU6-646P	10.10845814	-0.850497691	0.020685857
ENSG00000235674	LDHAP2	7.753085767	-0.848020035	0.024295224
ENSG00000066279	ASPM	38.17216033	-0.844703359	0.008707791
ENSG00000054356	PTPRN	55.88026765	-0.843607938	0.011024729
ENSG00000144290	SLC4A10	527.9786937	-0.839866613	0.023864516
ENSG00000278896	AC025031.5	11.99146906	-0.839821297	0.027133095
ENSG00000085491	SLC25A24	22.72927165	-0.83862035	0.024591298
ENSG00000254165	AC090739.1	15.1906757	-0.831775559	0.026226826
ENSG00000248516	AC105415.1	12.61407398	-0.827159868	0.041084443
ENSG00000255874	LINC00346	16.82886547	-0.821251415	0.013841437
ENSG00000211663	IGLV3-19	548.3400262	-0.81223737	0.032870115
ENSG00000274290	HIST1H2BE	46.83980578	-0.810754207	0.013197572
ENSG00000278661	TRAJ37	7.975953214	-0.810627829	0.030591223
ENSG00000259268	AC007950.1	8.371893599	-0.808985788	0.028587001
ENSG00000249601	LINC01187	13.1856974	-0.806951322	0.030195762
ENSG00000280161	AC022413.1	11.59745789	-0.806524961	0.007575248
ENSG00000239264	TXNDC5	20.89038393	-0.802739135	0.01170512
ENSG00000251441	RTEL1P1	33.23271758	-0.787557656	0.027316327
ENSG00000214659	KRT8P26	18.55693272	-0.785975502	0.008351184
ENSG00000269877	AC008753.3	10.74305132	-0.784048254	0.033171775
ENSG00000226306	NPY6R	10.53694414	-0.780917131	0.023148023
ENSG00000241644	INMT	9.702872313	-0.78091526	0.011798106
ENSG00000233077	LINC01271	41.34311002	-0.778729865	0.002700855
ENSG00000248905	FMN1	31.57539786	-0.776300979	0.045018636
ENSG00000279078	SND1-IT1	32.52383848	-0.776245993	0.014842155
ENSG00000228113	AC003991.1	41.92879544	-0.775937792	0.026047624
ENSG00000269924	AC024451.4	18.14901796	-0.775619837	0.000123394
ENSG00000184260	HIST2H2AC	52.77260846	-0.7755898	0.01748493
ENSG00000142621	FHAD1	28.29687501	-0.774184907	0.004674706
ENSG00000250644	AC068580.4	23.34697302	-0.769090954	0.019031712
ENSG00000225402	AC010878.1	54.59625115	-0.749508679	0.020260236
ENSG00000278002	AL627171.1	10.4641824	-0.745146393	0.045767275
ENSG00000277224	HIST1H2BF	35.04779237	-0.744799718	0.010637132
ENSG00000245105	A2M-AS1	52.88753702	-0.741064856	0.009279026
ENSG00000248265	FLJ12825	14.05629251	-0.73471943	0.023385384
ENSG00000266980	AC087289.1	17.59879025	-0.733017479	0.002329955
ENSG00000274419	TBC1D3D	35.39211051	-0.727573261	0.00871732
ENSG00000157554	ERG	27.46850775	-0.725309391	0.017982913
ENSG00000260070	AC006960.3	26.14075757	-0.724598691	0.012193562
ENSG00000177173	NAP1L4P1	15.23637347	-0.723903694	0.034346049
ENSG00000166435	XRRA1	344.480251	-0.720029464	0.001854359
ENSG00000228329	LINC01890	11.10337393	-0.718180895	0.008344549
ENSG00000078399	HOXA9	22.73336921	-0.714460737	0.002484658
ENSG00000008056	SYN1	20.81239193	-0.714410999	0.021561574

ENSG00000170955	CAVIN3	55.3065677	-0.712646171	0.024318516
ENSG00000112984	KIF20A	23.53460803	-0.709223691	0.014784357
ENSG00000165626	BEND7	156.2395911	-0.706758963	0.003163025
ENSG00000170231	FABP6	45.13020708	-0.702693479	0.019867969
ENSG00000218357	LINC01644	30.59709269	-0.702451132	0.021595273
ENSG00000224863	LINC01398	14.95388757	-0.700161489	0.026467609
ENSG00000188487	INSC	37.60658754	-0.698107883	0.016838204
ENSG00000258824	AL122035.1	20.72131532	-0.694416115	0.011532048
ENSG00000257335	MGAM	1735.032491	-0.692908044	0.036214467
ENSG00000274423	AC242843.1	39.02004355	-0.686808494	0.02024559
ENSG00000134058	CDK7	45.03450652	-0.684488657	0.006240362
ENSG00000228915	OR7E128P	10.39116124	-0.683634702	0.044524903
ENSG00000227339	THRAP3P1	8.624060417	-0.683471642	0.049736341
ENSG00000229000	SEPT7P8	12.30658104	-0.681893769	0.020242208
ENSG00000235962	RPL7AP53	13.21174472	-0.680452811	0.034054246
ENSG00000267934	AC010300.1	29.98364042	-0.679997981	0.00525028
ENSG00000230006	ANKRD36BP2	146.2240705	-0.679095303	0.029236466
ENSG00000260808	AP003096.1	9.316396373	-0.675534283	0.021471705
ENSG00000151789	ZNF385D	25.17511841	-0.67041276	0.03698415
ENSG00000234617	SNRK-AS1	14.35240746	-0.670015901	0.019031837
ENSG00000228906	AL353804.1	20.82526854	-0.669870498	0.014651258
ENSG00000206754	SNORD101	8.750715198	-0.668475314	0.033012869
ENSG00000137801	THBS1	819.7125383	-0.666371098	0.02653471
ENSG00000277157	HIST1H4D	31.43949516	-0.663781314	0.045940919
ENSG00000138079	SLC3A1	15.45378138	-0.659121467	0.018954839
ENSG00000170312	CDK1	38.32390467	-0.658178701	0.005460582
ENSG00000226393	IFNA20P	16.64947059	-0.656075204	0.024029189
ENSG00000279803	AC009090.5	8.175806325	-0.653028819	0.029346277
ENSG00000230069	LRRC37A15P	74.97950739	-0.645360639	0.013605175
ENSG00000182253	SYNM	130.1475015	-0.644506348	0.016215016
ENSG00000124657	OR2B6	33.59561678	-0.642766648	0.03936062
ENSG00000123485	HJURP	37.75952903	-0.641909302	0.012846372
ENSG00000148773	MKI67	336.4902135	-0.639516679	0.002121301
ENSG00000180138	CSNK1A1L	50.388092	-0.637219288	0.021121734
ENSG00000214700	C12orf71	11.24025726	-0.636855218	0.016696852
ENSG00000216657	GLRX3P2	9.622469791	-0.635174767	0.026409477
ENSG00000131747	TOP2A	151.7218058	-0.633944366	0.002249054
ENSG00000198580	AC073343.1	11.65107209	-0.633725889	0.038519772
ENSG00000253328	SUMO2P19	119.4843124	-0.632733337	0.012923539
ENSG00000253919	THAP12P7	28.28814271	-0.629884111	0.005203531
ENSG00000259351	AC015914.1	30.50583309	-0.628684554	0.024989558
ENSG00000205038	PKHD1L1	43.50991477	-0.628649219	0.004585447
ENSG00000232702	AL158050.1	15.17659278	-0.628446559	0.014289567
ENSG00000229419	RALGAPA1P1	63.79225196	-0.626628603	7.15E-05
ENSG00000258439	AC007956.1	21.30079211	-0.624804732	0.011585224
ENSG00000266651	AC093484.5	9.30365843	-0.624757163	0.023047902
ENSG00000112584	FAM120B	73.01777169	-0.622484182	0.035648418

ENSG00000224216	AC234781.1	10.77710285	-0.617758058	0.032630214
ENSG00000074527	NTN4	39.69849822	-0.616578788	0.03037132
ENSG00000123689	G0S2	91.19184709	-0.614329082	0.042398293
ENSG00000182397	DNM1P46	11.35764864	-0.612448742	0.042563992
ENSG00000237877	LINC01473	10.00505563	-0.610285613	0.047025962
ENSG00000253981	ALG1L13P	126.1550012	-0.607109013	0.001165412
ENSG00000171848	RRM2	100.2364082	-0.601202233	0.015485723
ENSG00000239998	LILRA2	665.4070908	-0.601116357	0.040902533
ENSG00000261229	AC021483.2	12.55456912	-0.60070232	0.018639745
ENSG00000189238	LINC00943	58.01327854	-0.599179141	0.034984605
ENSG00000223886	AC073073.1	20.5569901	-0.598396717	0.002644009
ENSG00000272102	AL133406.3	10.75582936	-0.596433494	0.024511877
ENSG00000111058	ACSS3	69.33039833	-0.593817013	0.013439051
ENSG00000086289	EPDR1	34.40718219	-0.593359483	0.03230668
ENSG00000259703	LINC00593	42.00703887	-0.592851983	0.033101131
ENSG00000230280	HNRNPA1P59	49.0855857	-0.591331816	0.025486147
ENSG00000242154	AC004884.2	13.27781578	-0.589117543	0.041627593
ENSG00000231826	LINC01819	49.68960255	-0.588262538	0.008086643
ENSG00000160224	AIRE	183.9364779	-0.586447534	0.046524373
ENSG00000105996	HOXA2	26.78092346	-0.585896769	0.038728721
ENSG00000171617	ENC1	1101.63082	-0.583259317	0.003323269
ENSG00000071282	LMCD1	17.77646435	-0.582729988	0.027698838
ENSG00000240135	AC114876.1	9.837413382	-0.581202823	0.028478192
ENSG00000260660	AC113208.3	119.2382523	-0.579924969	0.028482846
ENSG00000246203	AL353807.3	111.8597995	-0.577463666	0.043340124
ENSG00000255733	IFNG-AS1	50.84204439	-0.575952381	0.039270145
ENSG00000178075	GRAMD1C	629.7884133	-0.573167731	0.020563621
ENSG00000238160	AC116366.2	825.4232714	-0.571701556	0.007762958
ENSG00000158023	WDR66	40.71068152	-0.570455195	0.003856703
ENSG00000157456	CCNB2	57.55594544	-0.570006681	0.016171092
ENSG00000251161	AC020661.1	18.42065406	-0.56879258	0.016123531
ENSG00000129173	E2F8	15.21034568	-0.568509851	0.049520165
ENSG00000186185	KIF18B	28.61145664	-0.564047068	0.027731425
ENSG00000124875	CXCL6	44.50567005	-0.561981457	0.044865169
ENSG00000254750	CASP1P2	90.63530022	-0.560953735	0.013905843
ENSG00000162630	B3GALT2	101.045348	-0.560564186	0.043895051
ENSG00000248544	AC008676.1	35.06569769	-0.560038658	0.008664769
ENSG00000273249	BX649632.1	12.72168893	-0.559181115	0.049065769
ENSG00000161040	FBXL13	184.6942133	-0.558337403	0.008345069
ENSG00000118402	ELOVL4	86.95540827	-0.557528068	0.021680718
ENSG00000272084	AL137127.1	60.95736006	-0.554626011	0.026687562
ENSG00000267544	AC007229.1	15.31743656	-0.554004542	0.016563813
ENSG00000272902	TBC1D8-AS1	15.8222423	-0.55061432	0.036356287
ENSG00000248971	KRT8P46	53.83935991	-0.545851647	0.030785197
ENSG00000148848	ADAM12	109.9493351	-0.545467317	0.038671504
ENSG00000180769	WDFY3-AS2	35.33890355	-0.545065121	0.027414102
ENSG00000238005	AL391832.2	185.7177152	-0.54285759	0.014925117

ENSG00000051341	POLQ	34.3440421	-0.542029293	0.033506454
ENSG00000238098	ABCA17P	21.50443753	-0.537946556	0.032210467
ENSG00000232713	AC010733.1	36.31587612	-0.536389413	0.016326027
ENSG00000211459	MT-RNR1	101674.1977	-0.536221384	0.008293541
ENSG00000123473	STIL	78.59313795	-0.533779721	0.011829184
ENSG00000272839	AC011899.3	72.24875512	-0.53358593	0.041607141
ENSG00000137261	KIAA0319	366.5220501	-0.532411456	0.032086219
ENSG00000275367	AC092111.1	30.75307312	-0.528856446	0.031441563
ENSG00000092853	CLSPN	37.78467387	-0.528694162	0.011274524
ENSG00000259144	RANBP20P	51.50439734	-0.526426995	0.026990057
ENSG00000279386	AC021106.3	57.49752636	-0.523768278	0.029490036
ENSG00000280173	AC104447.1	102.1419255	-0.52172017	0.000977414
ENSG00000122861	PLAU	55.77957754	-0.519349414	0.043822029
ENSG00000224596	ZMIZ1-AS1	37.20484717	-0.516892192	0.029262058
ENSG00000133026	MYH10	208.3090964	-0.514352106	0.010952041
ENSG00000269028	MTRNR2L12	28.28741771	-0.507295591	0.036095036
ENSG00000253320	AZIN1-AS1	23.28742708	-0.505405886	0.031423769
ENSG00000280159	AC016583.1	42.6416103	-0.50358937	0.021639108
ENSG00000163701	IL17RE	103.8411266	-0.502688995	0.039301463
ENSG00000092200	RPGRIP1	747.6283749	-0.502515394	0.004420706
ENSG00000172771	EFCAB12	233.7422815	-0.496116303	0.02683683
ENSG00000094804	CDC6	55.21203569	-0.495627289	0.012195744
ENSG00000181350	LRRC75A	531.4109362	-0.495071266	0.01085364
ENSG00000180316	PNPLA1	274.6972446	-0.491031528	0.00484434
ENSG00000234614	AL450992.2	125.9582143	-0.489760365	0.009487639
ENSG00000188219	POTEE	9.648677876	-0.48778363	0.016177943
ENSG00000242324	AL049634.1	74.8703975	-0.487663525	0.005368018
ENSG00000249863	AC021106.1	153.9410198	-0.48543855	0.030411418
ENSG00000248734	AC008906.1	72.83825205	-0.485321032	0.039101404
ENSG00000005249	PRKAR2B	335.3366309	-0.483438846	0.049983916
ENSG00000212916	MAP10	101.6917685	-0.481971602	0.018524677
ENSG00000184451	CCR10	52.8480364	-0.480920031	0.022766228
ENSG00000133800	LYVE1	74.1593036	-0.478672916	0.033171891
ENSG00000250492	INTS6P1	22.50327653	-0.476994743	0.047213321
ENSG00000121152	NCAPH	74.75809065	-0.473802161	0.015879689
ENSG00000062524	LTK	1316.131415	-0.473461555	0.044939782
ENSG00000227354	RBM26-AS1	47.8118183	-0.473141519	0.037414026
ENSG00000070371	CLTCL1	559.6119616	-0.470620662	0.004383456
ENSG00000166359	WDR88	60.6062664	-0.46884134	0.027833896
ENSG00000163739	CXCL1	444.6454602	-0.465798978	0.012986962
ENSG00000262655	SPON1	140.7582259	-0.465278467	0.025831515
ENSG00000196549	MME	18188.52207	-0.464496949	0.026481635
ENSG00000175773	AP002986.1	26.37158081	-0.464342173	0.021620094
ENSG00000175711	B3GNTL1	421.2789311	-0.463617162	0.007366478
ENSG00000165115	KIF27	309.0709733	-0.462322803	0.030675794
ENSG00000171595	DNAI2	46.26100208	-0.460307449	0.00829202
ENSG00000269564	AC008753.2	23.05719975	-0.457701079	0.04521703

ENSG00000228623	ZNF883	40.18358929	-0.456528473	0.019053311
ENSG00000232742	RHOQP2	30.82562462	-0.456387771	0.023777081
ENSG00000186642	PDE2A	85.02942931	-0.456386587	0.002172019
ENSG00000089505	CMTM1	216.8478734	-0.453036604	0.027263573
ENSG00000058866	DGKG	563.4756673	-0.452608154	0.000418717
ENSG00000173585	CCR9	155.682911	-0.452547437	0.03929486
ENSG00000177570	SAMD12	91.73888599	-0.445148573	0.035973257
ENSG00000086570	FAT2	42.79055897	-0.444745277	0.026114138
ENSG00000087303	NID2	28.89594903	-0.44466578	0.029562395
ENSG00000214688	C10orf105	520.48949	-0.443940743	0.017021093
ENSG00000183690	EFHC2	208.0974243	-0.44206455	0.045177498
ENSG00000157985	AGAP1	262.758785	-0.439763107	0.034507715
ENSG00000203819	HIST2H2BC	42.45577517	-0.436668763	0.045785209
ENSG00000226121	AC009487.2	390.1412256	-0.434338746	0.023255255
ENSG00000189233	NUGGC	141.0616271	-0.431396614	0.033468295
ENSG00000172748	ZNF596	116.2745881	-0.430852302	0.026087969
ENSG00000187699	C2orf88	707.6463598	-0.430537002	0.02893861
ENSG00000112782	CLIC5	213.3386089	-0.429789556	0.014684683
ENSG00000228382	ITPKB-IT1	176.0756672	-0.429045439	0.040954299
ENSG00000259248	USP3-AS1	71.2730581	-0.427650148	0.025849693
ENSG00000155749	ALS2CR12	103.0111389	-0.426072467	0.022051721
ENSG00000179299	NSUN7	475.2022511	-0.423802514	0.044753021
ENSG00000183044	ABAT	1979.425142	-0.423542717	0.003014628
ENSG00000072422	RHOBTB1	120.5124464	-0.423064149	0.034798433
ENSG00000234337	AC026462.1	39.91897513	-0.422058469	0.00535302
ENSG00000100024	UPB1	214.5670352	-0.421719732	0.010963431
ENSG00000203876	ADD3-AS1	39.13870918	-0.42151147	0.012660737
ENSG00000212283	SNORD89	81.30807961	-0.421083699	0.033299362
ENSG00000243960	AL390195.1	20.76757128	-0.420765516	0.039666111
ENSG00000266601	AC018521.6	37.17498647	-0.420096702	0.008551051
ENSG00000258082	AL391832.3	68.20100065	-0.41703932	0.033277898
ENSG00000072952	MRVI1	5126.080896	-0.415632551	0.02689382
ENSG00000174028	FAM3C2	65.10096817	-0.415297701	0.048378984
ENSG00000244486	SCARF2	152.2271137	-0.415057057	0.020141028
ENSG00000004660	CAMKK1	2081.315591	-0.414909646	0.007816881
ENSG00000278311	GGNBP2	147.2471151	-0.414397519	0.01858164
ENSG00000135083	CCNJL	4160.029726	-0.413426201	0.031574123
ENSG00000187695	AC112484.1	125.0360655	-0.413259759	0.038880619
ENSG00000205890	AC108134.1	477.1190936	-0.412047223	0.047899065
ENSG00000278231	AL133342.1	113.3713341	-0.411439414	0.036569773
ENSG00000257226	AC079584.2	30.04287716	-0.409271009	0.049604851
ENSG00000233609	RPL10P19	151.4883775	-0.408782759	0.016165624
ENSG00000164953	TMEM67	76.68350988	-0.408411188	0.044284235
ENSG00000103512	NOMO1	151.8546611	-0.406483215	0.027915096
ENSG00000232952	AL512844.1	34.01577225	-0.404423083	0.035727055
ENSG00000280953	LINC01163	83.38321967	-0.404098741	0.025860924
ENSG00000215529	EFCAB8	95.9949931	-0.402433801	0.023882851

ENSG00000246263	UBR5-AS1	1236.045617	-0.401124068	0.032341151
ENSG00000233369	GTF2IP4	2186.795713	-0.400885432	0.003406042
ENSG00000249476	AC008467.1	310.3199348	-0.398915824	0.020118135
ENSG00000134571	MYBPC3	893.4193394	-0.398510848	0.033527091
ENSG00000075218	GTSE1	49.45114019	-0.397909938	0.045747247
ENSG00000132405	TBC1D14	3380.502632	-0.397266097	0.024082769
ENSG00000108932	SLC16A6	1152.500974	-0.397078064	0.012155411
ENSG00000236345	AL354719.2	230.4390018	-0.395692346	0.030504692
ENSG00000133808	MICALCL	279.1467198	-0.393270071	0.048416967
ENSG00000210082	MT-RNR2	289812.3275	-0.392861586	0.022780148
ENSG00000223750	SIRPB3P	39.88830774	-0.389802879	0.036485726
ENSG00000076356	PLXNA2	413.4635707	-0.388349125	0.02399458
ENSG00000169679	BUB1	112.8121238	-0.387833885	0.046902396
ENSG00000188107	EYS	86.46217065	-0.38678374	0.024851468
ENSG00000155657	TTN	1767.125207	-0.386684042	0.034585844
ENSG00000262001	DLGAP1-AS2	190.7403473	-0.386612606	0.040342163
ENSG00000119408	NEK6	2088.474729	-0.385788442	0.003746254
ENSG00000138160	KIF11	141.2836016	-0.385208719	0.009900356
ENSG00000119121	TRPM6	1396.757952	-0.383000461	0.039732839
ENSG00000159228	CBR1	1049.192822	0.381249761	0.009556258
ENSG00000173991	TCAP	224.7147618	0.382246802	0.037771677
ENSG00000255559	ZNF252P-AS1	45.79789416	0.382784628	0.029440156
ENSG00000152207	CYSLTR2	760.8115509	0.386349725	0.042730864
ENSG00000174137	FAM53A	69.61646071	0.386502957	0.020740774
ENSG00000228427	AL590764.1	184.1583508	0.386762335	0.001130832
ENSG00000197933	ZNF823	124.1244584	0.388764997	0.011214787
ENSG00000127184	COX7C	2524.970894	0.391158057	0.047809146
ENSG00000238058	AL355574.1	24.72289408	0.391181954	0.034604419
ENSG00000251432	AC108062.1	53.12185581	0.394165521	0.022551002
ENSG00000180061	TMEM150B	585.1196493	0.395588278	0.038883884
ENSG00000123975	CKS2	115.3569532	0.398735515	0.028724787
ENSG00000099889	ARVCF	297.1825104	0.399049086	0.03700055
ENSG00000164258	NDUFS4	245.5950919	0.400204411	0.047241401
ENSG00000164512	ANKRD55	449.1936183	0.40023061	0.030444077
ENSG00000278126	AC139768.1	54.15106574	0.400301993	0.01499029
ENSG00000005187	ACSM3	307.7973719	0.400781871	0.007388518
ENSG00000238000	AC116347.1	94.48434612	0.401174992	0.040950683
ENSG00000261222	AC064805.1	953.0834996	0.402440208	0.047167156
ENSG00000004838	ZMYND10	173.1957785	0.404314159	0.015969036
ENSG00000257702	LBX2-AS1	131.9482024	0.405416491	0.016498503
ENSG00000184221	OLIG1	1241.902385	0.406937569	0.016791924
ENSG00000234648	AL162151.2	220.4903335	0.409505995	0.040985494
ENSG00000162373	BEND5	123.194778	0.410300375	0.031030937
ENSG00000175591	P2RY2	408.1361869	0.410707156	0.002180877
ENSG00000138606	SHF	58.33816639	0.413361785	0.020178555
ENSG00000155363	MOV10	4304.899879	0.413547862	0.031302669
ENSG00000198417	MT1F	266.721998	0.421656773	0.014691673

ENSG00000113504	SLC12A7	903.9807731	0.424030518	0.033587059
ENSG00000243199	AC115223.1	198.6298549	0.424105695	0.00225653
ENSG00000115657	ABCB6	32.25176822	0.424543873	0.016168803
ENSG00000278607	AC015819.2	27.35602101	0.42470527	0.042921684
ENSG00000059915	PSD	222.2871179	0.42814874	0.038375184
ENSG00000144485	HES6	127.0292647	0.428710171	0.032854204
ENSG00000165457	FOLR2	290.7346421	0.429531344	0.014641631
ENSG00000243824	RPL12P6	30.22286627	0.431106898	0.027967432
ENSG00000178685	PARP10	12257.06533	0.431410064	0.009866146
ENSG00000168961	LGALS9	14270.15765	0.432382077	0.025996496
ENSG00000279332	AC090772.4	46.85201125	0.434988592	0.049579471
ENSG00000238103	RPL9P7	96.90753966	0.438286493	0.04980992
ENSG00000272343	AC107952.2	41.72705354	0.439615318	0.029391022
ENSG00000031691	CENPQ	87.23127557	0.439939263	0.041706737
ENSG00000140464	PML	6620.632666	0.440306749	0.04979526
ENSG00000173926	3-Mar	246.76655	0.442162532	0.014440409
ENSG00000171860	C3AR1	2454.252711	0.443298209	0.04255644
ENSG00000236383	LINC00854	185.3827474	0.444273449	0.011983368
ENSG00000179965	ZNF771	57.75725784	0.444540645	0.026805666
ENSG00000226525	RPS7P10	105.6398625	0.444753781	0.045372927
ENSG00000118162	KPTN	492.6961393	0.449033345	0.020756964
ENSG00000146677	AC004453.1	181.7355631	0.449406664	0.036101038
ENSG00000217716	RPS10P3	112.0914774	0.449775667	0.030270158
ENSG00000179921	GPBAR1	2128.791955	0.455685218	0.012534017
ENSG00000253865	AC131025.1	22.85036969	0.45577843	0.042063906
ENSG00000180340	FZD2	311.5864948	0.464082374	0.015689086
ENSG00000260401	AP002761.4	702.2044627	0.465286129	0.003183362
ENSG00000272644	AC097468.3	47.41739229	0.471834918	0.040939281
ENSG00000180917	CMTR2	89.73836417	0.474873787	0.018625444
ENSG00000260807	AC009041.2	751.3230361	0.477054087	0.049295467
ENSG00000185614	FAM212A	172.1484118	0.485988359	0.012783472
ENSG00000188282	RUFY4	202.0784285	0.48626754	0.036815091
ENSG00000282851	BISPR	769.256151	0.486908408	0.016314333
ENSG00000114315	HES1	101.3393146	0.493025209	0.033304407
ENSG00000267390	AC036176.1	36.55940347	0.496687381	0.027729379
ENSG00000059378	PARP12	7179.436122	0.49699015	0.031790561
ENSG00000103319	EEF2K	42.03433474	0.497217515	0.019244454
ENSG00000175287	PHYHD1	41.84692704	0.49738263	0.034908928
ENSG00000268510	IFNL3P1	20.55495017	0.498847594	0.045886391
ENSG00000261061	AC092718.4	35.95079076	0.499644035	0.031650924
ENSG00000134809	TIMM10	663.1060129	0.503263707	0.020807426
ENSG00000233223	AC016876.1	132.7388245	0.506388545	0.002243801
ENSG00000152942	RAD17	129.7838502	0.508245564	0.022151808
ENSG00000246560	AC018797.2	29.16463731	0.508885093	0.017121474
ENSG00000247934	AC022364.1	24.96784327	0.509238806	0.019090394
ENSG00000160223	ICOSLG	214.8866016	0.509952281	0.034337162
ENSG00000123685	BATF3	163.9052	0.511224071	0.018009687

ENSG00000272988	AC022392.1	20.21218733	0.514431828	0.049989286
ENSG00000166016	ABTB2	363.3889741	0.515073892	0.009240744
ENSG00000197816	CCDC180	141.933184	0.515821708	0.001248627
ENSG00000278974	AC093909.6	94.31340989	0.521379531	0.008312849
ENSG00000119632	IFI27L2	30.89923806	0.522070483	0.035855699
ENSG00000003096	KLHL13	29.25704251	0.524749293	0.042588616
ENSG00000108771	DHX58	3016.009727	0.525197395	0.033385886
ENSG00000168528	SERINC2	238.3210193	0.529944655	0.03755108
ENSG00000111247	RAD51AP1	33.22284556	0.530195531	0.046736286
ENSG00000108679	LGALS3BP	2413.572519	0.53192531	0.024150376
ENSG00000272398	CD24	1456.17268	0.532170758	0.002149096
ENSG00000166743	ACSM1	40.89666807	0.534178712	0.010381674
ENSG00000225131	PSME2P2	59.71014683	0.538882293	0.010880185
ENSG00000224892	RPS4XP16	115.3862516	0.539019851	0.008565948
ENSG00000168026	TTC21A	556.0243309	0.539560578	0.008995525
ENSG00000189269	DRICH1	58.29219709	0.544441109	0.044985387
ENSG00000130303	BST2	8252.21836	0.544506369	0.00639821
ENSG00000182557	SPNS3	800.1804141	0.546644553	0.000964815
ENSG00000114942	EEF1B2	174.3773564	0.546883352	0.007003525
ENSG00000241657	TRBV11-2	184.6747102	0.54729665	0.013496796
ENSG00000162490	DRAXIN	150.1915182	0.54833337	0.021406647
ENSG00000103056	SMPD3	1731.414463	0.551251201	0.00219971
ENSG00000144395	CCDC150	12.0511187	0.557493981	0.039085631
ENSG00000270020	AC009108.3	13.53040393	0.558719939	0.043608246
ENSG00000092067	CEBPE	752.1821727	0.560977339	0.007641124
ENSG00000228106	AL392172.1	38.13828701	0.563596421	0.017056138
ENSG00000005513	SOX8	123.8883171	0.565657027	0.01689351
ENSG00000228203	RNF144A-AS1	37.04718942	0.567838463	0.032564322
ENSG00000139438	FAM222A	19.91144917	0.56785586	0.04409245
ENSG00000144642	RBMS3	11.93869302	0.56932476	0.043570616
ENSG00000269946	AL158152.2	18.58197248	0.57021059	0.043204681
ENSG00000279254	AC020604.1	32.64179284	0.573299314	0.032665094
ENSG00000279518	AC083843.3	24.92810218	0.576975891	0.015722028
ENSG00000223749	MIR503HG	25.00380762	0.57969086	0.007433956
ENSG00000282885	AL627171.2	14.61074799	0.581419443	0.024835435
ENSG00000272142	AL359643.3	24.63982608	0.581903513	0.005012888
ENSG00000274897	PANO1	20.11925774	0.583214711	0.023584543
ENSG00000256393	AC138123.1	36.45710491	0.586347717	0.027322711
ENSG00000247134	AC090204.1	32.31318711	0.586793336	0.011621269
ENSG00000021488	SLC7A9	15.46383985	0.587835286	0.018752359
ENSG00000243517	AC024940.2	16.76576754	0.590012302	0.017695484
ENSG00000272677	AC124016.1	27.58004176	0.5926233	0.002127534
ENSG00000149571	KIRREL3	43.67216048	0.592751968	0.024349534
ENSG00000272696	AL359091.3	24.92221346	0.598281947	0.016040587
ENSG00000234184	LINC01781	51.72895685	0.6018095	0.016995463
ENSG00000158747	NBL1	40.17164295	0.602314323	0.040448167
ENSG00000068079	IFI35	4723.250619	0.60243708	0.027467181

ENSG00000238186	AL603839.2	10.86800334	0.602834309	0.019245067
ENSG00000132698	RAB25	29.68864458	0.603865707	0.018482054
ENSG00000130433	CACNG6	226.189409	0.613345686	0.016294529
ENSG00000237550	RPL9P9	45.52358993	0.614675374	0.046152143
ENSG00000278668	AC005899.7	23.64855456	0.615022869	0.00195897
ENSG00000259479	SORD2P	78.75924893	0.61721033	0.031066913
ENSG00000127743	IL17B	22.5873432	0.618722049	0.012291807
ENSG00000187690	CXorf67	14.09648209	0.61900422	0.047888676
ENSG00000234493	RHOXF1P1	34.63215092	0.621124219	0.049277951
ENSG00000167779	IGFBP6	33.2114837	0.621258502	0.021789723
ENSG00000198400	NTRK1	65.70779907	0.624104043	0.010698564
ENSG00000272010	AC100814.1	18.34951673	0.62869637	0.02344404
ENSG00000099957	P2RX6	36.62324853	0.62976522	0.008980197
ENSG00000091181	IL5RA	958.7311957	0.63078683	0.017002446
ENSG00000136315	AL355922.1	48.56066212	0.632851297	0.042457295
ENSG00000156194	PPEF2	12.2105003	0.634349243	0.04376948
ENSG00000104918	RETN	438.9419301	0.636553647	0.044140806
ENSG00000090104	RGS1	47.0016444	0.638058007	0.032241773
ENSG00000063660	GPC1	44.89095784	0.640863078	0.001280388
ENSG00000248677	LINC02102	7.496682708	0.647411261	0.04431191
ENSG00000131037	EPS8L1	60.73730105	0.649234857	0.000167704
ENSG00000020577	SAMD4A	317.1227446	0.650035238	0.018510191
ENSG00000266524	GDF10	12.36849291	0.650925568	0.020440361
ENSG00000169385	RNASE2	1706.923205	0.652015693	0.013468075
ENSG00000177685	CRACR2B	278.4652812	0.653352956	0.001372941
ENSG00000080166	DCT	11.81273153	0.65416302	0.031682325
ENSG00000142408	CACNG8	199.3040132	0.656708431	0.003416365
ENSG00000274191	AC026333.4	10.84125758	0.658003769	0.034963334
ENSG00000264281	AC016596.1	72.05132032	0.658928862	0.004327695
ENSG00000183134	PTGDR2	1334.626949	0.660799493	0.003983844
ENSG00000175643	RMI2	152.4775795	0.66647727	0.018951509
ENSG00000204619	PPP1R11	102.0084759	0.674505459	0.039443813
ENSG00000205531	NAP1L4	632.3157857	0.67941589	0.000277163
ENSG00000174837	ADGRE1	3774.021533	0.682842318	8.35E-05
ENSG00000260428	SCX	30.24816809	0.686024318	0.004542651
ENSG00000092068	SLC7A8	156.9223678	0.686964291	0.04027009
ENSG00000186715	MST1L	59.12705648	0.691776988	0.004963577
ENSG00000273240	AC013468.1	14.08209366	0.695687264	0.010031211
ENSG00000224635	AL391095.1	39.87268788	0.697932353	0.016175255
ENSG00000227527	AC096540.1	11.55920934	0.699906258	0.008882366
ENSG00000226155	AC124944.1	11.17005023	0.702690471	0.038145952
ENSG00000232346	Z74021.1	56.07747265	0.703348087	0.0290494
ENSG00000100376	FAM118A	3687.731704	0.704431982	0.046290546
ENSG00000131650	KREMEN2	11.65347521	0.704770116	0.013731973
ENSG00000128242	GAL3ST1	8.193345293	0.704896256	0.036305625
ENSG00000240509	RPL34P18	28.33778755	0.70566175	0.025565148
ENSG00000125148	MT2A	1501.809374	0.707841345	0.014333335

ENSG00000134030	CTIF	32.64817006	0.713396707	0.010096583
ENSG00000128606	LRRC17	15.34495503	0.71586992	0.010274692
ENSG00000129757	CDKN1C	23.83448905	0.716487312	0.03172611
ENSG00000282933	RHOXF1P3	16.57392812	0.722930526	0.028745865
ENSG00000160180	TFF3	34.66810954	0.724648738	0.04514458
ENSG00000274373	AC148476.1	30.12786778	0.728730337	0.018825712
ENSG00000234456	MAGI2-AS3	45.64885641	0.734487862	0.011077252
ENSG00000128342	LIF	16.16770112	0.736760694	0.032685979
ENSG00000185338	SOCS1	372.2902682	0.73840683	0.003580482
ENSG00000172602	RND1	14.13023054	0.739364503	0.040725
ENSG00000125266	EFNB2	15.02060807	0.742080701	0.006393703
ENSG00000282608	ADORA3	351.1440801	0.752370939	0.001470371
ENSG00000134824	FADS2	1166.345722	0.755905898	0.033594872
ENSG00000250057	AC114781.2	9.192215862	0.756650629	0.012374498
ENSG00000123843	C4BPB	14.53465481	0.756861512	0.039310739
ENSG00000277511	AC116407.2	79.03824164	0.759839517	0.012565817
ENSG00000111335	OAS2	13215.75606	0.764705763	0.028338967
ENSG00000277491	AC087392.5	13.4941909	0.767029666	0.034930801
ENSG00000099377	HSD3B7	98.23461712	0.768484788	0.000463765
ENSG00000135114	OASL	5472.777329	0.774261362	0.048133682
ENSG00000255478	AP000944.1	8.274527533	0.775266431	0.011673619
ENSG00000234776	C11orf94	8.349835937	0.781276429	0.013580916
ENSG00000254017	IGHEP2	13.74247626	0.790025525	0.04930902
ENSG00000154099	DNAAF1	13.62279352	0.790397858	0.002238826
ENSG00000254648	AP000911.3	7.154024321	0.795005225	0.02904278
ENSG00000282850	RHOXF1P2	16.89372056	0.796218596	0.034545354
ENSG00000215148	PRSS41	25.71688421	0.796371099	0.015219792
ENSG00000233966	UBE2SP1	8.661206302	0.798552834	0.044809954
ENSG00000229605	RPL21P93	10.12134956	0.805561771	0.005401795
ENSG00000105366	SIGLEC8	646.2224644	0.805897633	0.00483872
ENSG00000161328	LRRC56	35.33225336	0.807771409	0.042847639
ENSG00000170486	KRT72	679.1092697	0.809774555	0.042839964
ENSG00000054179	ENTPD2	126.2059909	0.817507974	0.04988964
ENSG00000197728	RPS26	11775.34263	0.820010135	0.019948271
ENSG00000089127	OAS1	9233.019636	0.820895271	0.026236242
ENSG00000258811	AL583810.2	15.59836919	0.823569114	0.016853339
ENSG00000103355	PRSS33	1094.696821	0.825450397	0.002612422
ENSG00000112759	SLC29A1	1598.201671	0.826786658	6.91E-05
ENSG00000105205	CLC	6791.854548	0.836444205	0.001108064
ENSG00000228495	LINC01013	22.96771622	0.837706222	0.0109838
ENSG00000272733	AP000345.2	30.09436267	0.842872471	0.005328833
ENSG00000166592	RRAD	12.00068527	0.849118817	0.019147228
ENSG00000131773	KHDRBS3	10.53868549	0.850470157	0.010917076
ENSG00000275329	AL138781.2	14.56328803	0.850497981	0.001810365
ENSG00000168004	HRASLS5	57.13463181	0.851973711	0.003618526
ENSG00000196141	SPATS2L	573.5661953	0.863012904	0.025563861
ENSG00000188157	AGRN	482.3955877	0.863972495	0.006109117

ENSG00000205927	OLIG2	242.8736343	0.864977842	0.005066163
ENSG00000111344	RASAL1	16.09702061	0.873914552	0.014660057
ENSG00000260081	AF274858.3	24.03190843	0.883125513	6.03E-05
ENSG00000148204	CRB2	28.25214434	0.889011586	0.006769567
ENSG00000204256	BRD2	362.2848621	0.890170781	0.029099413
ENSG00000204482	LST1	11.07710969	0.896094985	0.046676636
ENSG00000256826	ATP5J2P4	10.27246034	0.897154318	0.032125226
ENSG00000188290	HES4	242.0346504	0.898370992	0.021671852
ENSG00000160932	LY6E	560.0061398	0.89839277	0.00349705
ENSG00000212994	RPS26P6	59.59767124	0.904692195	0.025777734
ENSG00000196656	AC004057.1	709.837289	0.908079533	0.012061839
ENSG00000284690	AC079325.2	1041.869197	0.908153536	0.007195576
ENSG00000126709	IFI6	10375.43157	0.919210541	0.03640824
ENSG00000204209	DAXX	40.85150158	0.926352151	0.020099702
ENSG00000138755	CXCL9	29.39701857	0.931444666	0.005008834
ENSG00000182612	TSPAN10	14.65255966	0.93385946	0.001285721
ENSG00000178752	ERFE	17.47000974	0.934338276	0.045410186
ENSG00000254389	RHPN1-AS1	8.624540615	0.947204616	0.000946719
ENSG00000100628	ASB2	20.83497025	0.953344551	0.00354131
ENSG00000157368	IL34	46.65101086	0.962509019	0.002641819
ENSG00000223313	RNU6-516P	12.32699112	0.963342814	0.00413446
ENSG00000185507	IRF7	488.4203743	0.99682651	0.000908531
ENSG00000187608	ISG15	8303.39895	1.032306459	0.02778545
ENSG00000126467	TSKS	80.51764382	1.037390644	0.005738677
ENSG00000175356	SCUBE2	10.42611782	1.044973175	0.011167053
ENSG00000078081	LAMP3	372.7131071	1.050332537	0.037080299
ENSG00000133169	BEX1	11.7465822	1.090279951	0.005301008
ENSG00000275106	AC025594.3	29.1824646	1.095296085	0.000390312
ENSG00000247627	MTND4P12	126.933877	1.096405255	0.019833181
ENSG00000269720	CCDC194	18.16413327	1.105136819	0.00276136
ENSG00000137672	TRPC6	37.81560906	1.110869346	0.004970791
ENSG00000234745	HLA-B	46932.00045	1.123710061	0.027510853
ENSG00000225431	LINC01671	8.876966827	1.155610712	0.002130021
ENSG00000225964	NRIR	157.7716771	1.164691577	0.011545949
ENSG00000225071	AC004552.1	12.53825696	1.179327409	0.020162929
ENSG00000131203	IDO1	591.0394144	1.185990185	0.001404377
ENSG00000184979	USP18	386.3323467	1.24636106	0.004409958
ENSG00000169245	CXCL10	159.5420988	1.280293572	0.008047931
ENSG00000272512	AL645608.8	21.58800224	1.435017641	0.011193969
ENSG00000134321	RSAD2	8039.476399	1.458053632	0.031771793
ENSG00000276566	IGKV1D-13	51.24442257	1.555660421	0.004864349
ENSG00000158525	CPA5	98.67529285	1.583224614	0.010157972
ENSG00000163666	HESX1	18.70534655	1.58613055	0.001653358
ENSG00000109321	AREG	21.26010596	1.703704663	0.00294204
ENSG00000205837	LINC00487	35.48160773	1.70534527	0.003115973
ENSG00000206503	HLA-A	14370.23025	1.804552712	0.00471681

Baseline responders vs. non-responders

Gene ID	Gene Name	Base Mean	log2 Fold Change	p-value
ENSG00000225690	TREML5P	83.92634681	-3.178281735	0.002493791
ENSG00000109321	AREG	31.53818795	-2.830957559	0.006488382
ENSG00000153157	SYCP2L	35.07253022	-2.442217197	7.23E-05
ENSG00000230076	RPL10P6	122.4024464	-2.396636821	0.018643438
ENSG00000131042	LILRB2	1127.20738	-2.035552894	0.033669941
ENSG00000165949	IFI27	107.1508879	-2.00663783	0.049948008
ENSG00000034239	EFCAB1	9.342617075	-1.927370872	0.000431489
ENSG00000226102	SEPT7P3	17.1013296	-1.841403488	0.003003959
ENSG00000254810	AP001189.3	16.48749845	-1.763939272	0.003836969
ENSG00000165948	IFI27L1	14.12033814	-1.734133245	0.004703996
ENSG00000283403	AC005394.2	16.90301167	-1.702319131	0.001720586
ENSG00000101115	SALL4	10.61086968	-1.632941107	0.014388446
ENSG00000186115	CYP4F2	18.77040433	-1.623376504	0.026053424
ENSG00000248126	AC091849.1	10.67367778	-1.576606271	0.009678753
ENSG00000162885	B3GALNT2	15.46665776	-1.574427152	0.004137494
ENSG00000198690	FAN1	30.71427648	-1.52962958	0.00643621
ENSG00000067798	NAV3	35.74581997	-1.515659509	0.00201861
ENSG00000278661	TRAJ37	6.130250009	-1.468735741	0.023287348
ENSG00000212541	RNU6-510P	15.52102465	-1.431150369	0.014687305
ENSG00000172602	RND1	17.38184213	-1.403359365	0.005206426
ENSG00000109674	NEIL3	8.741517359	-1.392283768	0.016261151
ENSG00000278745	AL391903.3	56.05067616	-1.381401736	0.027430023
ENSG00000213316	LTC4S	22.9876735	-1.375054793	0.018791251
ENSG00000237926	AC239367.3	20.0470388	-1.34798276	0.047639808
ENSG00000104524	PYCR3	13.27140574	-1.306044697	0.020598837
ENSG00000154380	ENAH	17.94287314	-1.298339232	0.001471727
ENSG00000183421	RIPK4	19.45559226	-1.289681947	0.002892405
ENSG00000224550	AC114491.1	11.87224037	-1.280202303	0.007210708
ENSG00000132622	HSPA12B	17.66565969	-1.258616973	0.025932686
ENSG00000167880	EVPL	88.28980665	-1.240193963	0.049053187
ENSG00000134548	SPX	44.80121624	-1.228209806	0.003795456
ENSG00000228079	AC012368.2	15.08632727	-1.215440323	0.020392451
ENSG00000260423	LINC02367	11.2149439	-1.208857323	0.005186436
ENSG00000205221	VIT	13.40875659	-1.204067386	0.041881528
ENSG00000123219	CENPK	197.9943609	-1.202263872	0.032527582
ENSG00000253552	HOXA-AS2	21.80500962	-1.20040807	0.025361651
ENSG00000250326	AC104596.1	27.00845802	-1.198169298	0.005661441
ENSG00000172640	OR10AD1	9.427617614	-1.192820788	0.037432452
ENSG00000174697	LEP	26.08098158	-1.167119437	0.012796259
ENSG00000129204	USP6	52.48373214	-1.161148585	0.007730595
ENSG00000169255	B3GALNT1	29.61562261	-1.160449875	0.003962178
ENSG00000236397	DDX11L2	284.1689593	-1.146901117	0.015226728
ENSG00000173546	CSPG4	8.778865896	-1.145181063	0.031041661
ENSG00000227359	AC017074.1	12.93114637	-1.141156359	0.006858658

ENSG00000164929	BAALC	21.45652018	-1.130915171	0.024188125
ENSG00000198732	SMOC1	17.78756637	-1.127641568	0.008031797
ENSG00000166770	ZNF667-AS1	123.7861992	-1.097712368	0.000456676
ENSG00000152760	TCTEX1D1	13.31489187	-1.094728943	0.027299057
ENSG00000139044	B4GALNT3	469.6170274	-1.094060872	0.028092418
ENSG00000248971	KRT8P46	47.97481951	-1.072039435	0.003532612
ENSG00000197134	ZNF257	64.06609913	-1.06962979	0.015673749
ENSG00000122786	CALD1	54.7223877	-1.053311717	0.000556868
ENSG00000249646	OR7E94P	33.34958563	-1.04828438	0.010583714
ENSG00000135919	SERPINE2	118.2208034	-1.039086191	0.014467613
ENSG00000238041	AP004245.1	6.87458392	-1.036639696	0.037081592
ENSG00000128641	MYO1B	45.70665275	-1.01965273	0.006621375
ENSG00000259803	SLC22A31	25.04315038	-1.017527064	0.029943892
ENSG00000225544	AC245452.2	73.46291385	-1.00609319	0.024375829
ENSG00000256582	LINC02390	31.87086334	-1.004266589	0.021798064
ENSG00000078018	MAP2	20.24666782	-0.994242613	0.047981737
ENSG00000137831	UACA	73.78042182	-0.979098154	0.015293549
ENSG00000236090	LDHAP3	13.9782895	-0.978207358	0.019155168
ENSG00000106772	PRUNE2	171.4157507	-0.976244574	0.013275809
ENSG00000197283	SYNGAP1	104.7974443	-0.971136332	0.01558609
ENSG00000236792	AL513175.2	7.902304576	-0.970022425	0.047028771
ENSG00000085741	WNT11	21.77282252	-0.965691342	0.0129296
ENSG00000196581	AJAP1	56.97217508	-0.963577727	0.018925107
ENSG00000172322	CLEC12A	8855.364358	-0.953253828	0.013974523
ENSG00000204381	LAYN	21.16567213	-0.950847182	0.046629756
ENSG00000184500	PROS1	64.53330261	-0.948330881	0.013335177
ENSG00000005700	IBTK	96.06199394	-0.936335026	0.044599362
ENSG00000230006	ANKRD36BP2	118.8536958	-0.932921743	0.02056605
ENSG00000238241	CCR12P	106.942841	-0.932148604	0.035074749
ENSG00000159860	TCAF2P1	21.7338724	-0.931584036	0.020948357
ENSG00000099250	NRP1	76.45437422	-0.911213682	0.032438288
ENSG00000174123	TLR10	1633.81039	-0.908546039	0.014250096
ENSG00000275854	AC084824.4	19.88994972	-0.898786564	0.033192539
ENSG00000054690	PLEKHH1	17.59966924	-0.893149151	0.041707328
ENSG00000205670	SMIM11A	33.03378589	-0.889346147	0.043248804
ENSG00000272555	AC009974.1	49.02682134	-0.882077606	0.025319112
ENSG00000280079	AC011447.7	23.57306924	-0.8801472	0.002822745
ENSG00000026559	KCNG1	147.6966519	-0.878726061	0.024553566
ENSG00000171798	KNDC1	131.30778	-0.877741957	0.031545121
ENSG00000164056	SPRY1	43.9489627	-0.875348988	0.030631015
ENSG00000219222	RPL12P47	13.34684234	-0.872259172	0.04951295
ENSG00000117519	CNN3	103.8699279	-0.857017874	0.013004106
ENSG00000152990	ADGRA3	88.29251226	-0.855284448	0.010027658
ENSG00000184226	PCDH9	181.6896977	-0.853991661	0.043505159
ENSG00000183691	NOG	483.1635715	-0.847526052	0.031595048
ENSG00000167414	GNG8	31.53455127	-0.842649348	0.049325142
ENSG00000163735	CXCL5	190.8724247	-0.84126802	0.025030297

ENSG00000260773	AC055855.2	20.35609808	-0.839963721	0.015761279
ENSG00000267226	AC104971.2	9.679553005	-0.838056172	0.036668202
ENSG00000214189	ZNF788	60.89329258	-0.832392693	0.000122485
ENSG00000160191	PDE9A	243.5961685	-0.831511107	0.002145174
ENSG00000153253	SCN3A	75.14749595	-0.824302485	0.047874376
ENSG00000183960	KCNH8	106.8141539	-0.804818284	0.032229411
ENSG00000091972	CD200	261.8953351	-0.803568459	0.007916521
ENSG00000261754	AC008555.1	64.16726667	-0.798250306	0.003390528
ENSG00000167191	GPRC5B	54.76211908	-0.79284731	0.038822846
ENSG00000154914	USP43	29.51798919	-0.792787168	0.033663874
ENSG00000164120	HPGD	301.3447291	-0.792368349	0.044631959
ENSG00000254473	AL354920.1	46.55130673	-0.791173651	0.018960195
ENSG00000272092	AC087623.3	18.29138542	-0.786608482	0.009751717
ENSG00000246430	LINC00968	43.88740046	-0.782149204	0.015418143
ENSG00000108852	MPP2	81.14555269	-0.775761908	0.0396558
ENSG00000163736	PPBP	2550.943597	-0.772812541	0.022517151
ENSG00000142188	TMEM50B	52.64097154	-0.769468513	0.038732059
ENSG00000272034	SNORD14A	84.93824007	-0.766443367	0.036891332
ENSG00000236762	RPL19P16	16.26522156	-0.762683891	0.039300213
ENSG00000104267	CA2	778.6405946	-0.762578308	0.021214242
ENSG00000197632	SERPINB2	88.33068513	-0.751869057	0.007129642
ENSG00000104341	LAPTM4B	243.9156749	-0.734201673	0.033837554
ENSG00000143942	CHAC2	62.79933534	-0.726865415	0.028123108
ENSG00000224183	SDHDP6	85.15089948	-0.726663071	0.006222753
ENSG00000240875	LINC00886	66.33138855	-0.7123675	0.045243813
ENSG00000211771	TRBJ2-7	102.4040892	-0.70719249	0.027966787
ENSG00000205038	PKHD1L1	38.72115552	-0.698847867	0.008562773
ENSG00000002587	HS3ST1	78.0332351	-0.697267377	0.013556609
ENSG00000250334	LINC00989	269.1096757	-0.695594035	0.044282886
ENSG00000266931	AC125232.2	32.66241252	-0.694236951	0.019023969
ENSG00000267508	ZNF285	97.42799436	-0.690615188	0.021798955
ENSG00000106537	TSPAN13	520.0516732	-0.684739772	0.027101034
ENSG00000188739	RBM34	42.21919529	-0.67429034	0.03246837
ENSG00000117013	KCNQ4	26.12656142	-0.668986056	0.034823799
ENSG00000005513	SOX8	151.4831205	-0.666778621	0.045099078
ENSG00000125869	LAMP5	138.9384641	-0.66211805	0.022192689
ENSG00000165682	CLEC1B	373.4979918	-0.661439234	0.042997011
ENSG00000005249	PRKAR2B	279.8653181	-0.659882908	0.043630972
ENSG00000105971	CAV2	26.58471469	-0.658625388	0.033086527
ENSG00000091622	PITPNM3	38.2928158	-0.656445721	0.013057718
ENSG00000240356	RPL23AP7	267.4880402	-0.645249793	0.021365995
ENSG00000172985	SH3RF3	531.4271983	-0.639409891	0.007160186
ENSG00000171291	ZNF439	518.8930258	-0.637062601	0.017067513
ENSG00000229939	AL589880.1	39.83507648	-0.636653417	0.039409838
ENSG00000105426	PTPRS	297.1964602	-0.629198753	0.042042345
ENSG00000136999	NOV	883.8384105	-0.622088435	0.043744773
ENSG00000169439	SDC2	179.5713257	-0.618624466	0.031135637

ENSG00000270562	AC097634.1	137.8846103	-0.612774439	0.008047286
ENSG00000188171	ZNF626	184.0462752	-0.6037681	0.030721899
ENSG00000121570	DPPA4	40.1788173	-0.595376261	0.045978266
ENSG00000226352	PSPC1-AS2	46.16830963	-0.593978343	0.019639826
ENSG00000179889	PDXDC1	162.1276489	-0.583436792	0.03710436
ENSG00000112655	PTK7	271.8167509	-0.572291602	0.045185073
ENSG00000233355	CHRM3-AS2	2211.409249	-0.568924469	0.04579287
ENSG00000139880	CDH24	89.80963532	-0.553960533	0.020434811
ENSG00000275964	AL355001.2	91.88865686	-0.528918525	0.009655171
ENSG00000165943	MOAP1	46.31187138	-0.523961686	0.032044968
ENSG00000164512	ANKRD55	503.1575541	-0.52215214	0.047442444
ENSG00000197744	PTMAP2	131.3743887	-0.515752401	0.040506747
ENSG00000236345	AL354719.2	220.7389476	-0.513619748	0.044347859
ENSG00000172594	SMPDL3A	418.5073417	-0.511023334	0.040818389
ENSG00000154153	RETREG1	752.7897246	-0.503244639	0.03981984
ENSG00000110042	DTX4	1343.977208	-0.500037954	0.012332519
ENSG00000124225	PMEPA1	297.1385963	-0.484243833	0.010192379
ENSG00000099139	PCSK5	519.593061	-0.478710786	0.039452882
ENSG00000126217	MCF2L	325.9048161	-0.459965444	0.032860026
ENSG00000274712	AC005332.7	470.8803545	-0.44393328	0.043753592
ENSG00000140092	FBLN5	680.2658028	-0.436025128	0.034864153
ENSG00000169762	TAPT1	1556.2627	-0.433754028	0.016909966
ENSG00000106484	MEST	156.3493416	-0.428412502	0.03321992
ENSG00000164649	CDCA7L	697.9530523	-0.412637381	0.022821728
ENSG00000144218	AFF3	868.1156225	-0.405582425	0.025345641
ENSG00000213213	CCDC183	68.74897917	-0.3918615	0.044849708
ENSG00000120451	SNX19	4108.440407	-0.389522633	0.01617797
ENSG00000186187	ZNRF1	359.8223184	0.37893987	0.03574889
ENSG00000125388	GRK4	81.29327983	0.380855337	0.049944754
ENSG00000106327	TFR2	271.5227955	0.397628278	0.043001035
ENSG00000214975	PPIAP29	145.679054	0.425128379	0.025345461
ENSG00000147454	SLC25A37	104626.8985	0.447067309	0.04559021
ENSG00000115902	SLC1A4	653.9175321	0.467677318	0.004933834
ENSG00000236565	HNRNPA3P5	90.5437156	0.470803281	0.013706798
ENSG00000168487	BMP1	334.7572301	0.471528286	0.040355224
ENSG00000162461	SLC25A34	110.6025758	0.479032164	0.042262579
ENSG00000107954	NEURL1	761.284512	0.481869206	0.040417983
ENSG00000166446	CDYL2	448.6730756	0.484705495	0.033834036
ENSG00000172965	MIR4435-2HG	1230.68593	0.500602122	0.037508826
ENSG00000065621	GSTO2	82.82796807	0.510401386	0.016065928
ENSG00000112599	GUCA1B	118.6071902	0.531044684	0.022645686
ENSG00000235505	AP002004.1	352.643936	0.53942062	0.0243548
ENSG00000172215	CXCR6	842.8030181	0.54612564	0.035674462
ENSG00000266088	AC004585.1	167.5646039	0.54825847	0.027435029
ENSG00000143554	SLC27A3	219.4725598	0.552724895	0.034923755
ENSG00000223551	TMSB4XP4	371.1023276	0.552777576	0.041520917
ENSG00000254206	NPIPB11	244.5743285	0.558528721	0.035512901

ENSG00000230747	AC021188.1	52.33730636	0.561397254	0.027903729
ENSG00000222041	CYTOR	525.6450831	0.569723372	0.007776137
ENSG00000146054	TRIM7	213.9511041	0.576749266	0.026284949
ENSG00000141028	CDRT15P1	63.67230361	0.581144421	0.036883773
ENSG00000186715	MST1L	69.9061874	0.584497782	0.049856464
ENSG00000162438	CTRC	46.65714017	0.595895566	0.038758042
ENSG00000244617	ASPRV1	1356.092687	0.601241922	0.045820182
ENSG00000267281	AC023509.3	97.44772023	0.616429585	0.02948283
ENSG00000174871	CNIH2	59.83658826	0.624496398	0.019795843
ENSG00000241657	TRBV11-2	214.9532597	0.635164975	0.021641735
ENSG00000129038	LOXL1	56.20910545	0.639923114	0.03742517
ENSG00000072952	MRVI1	4921.236666	0.640462723	0.013077743
ENSG00000265787	CYP4F35P	71.32488577	0.640526112	0.02549685
ENSG00000260517	AC009093.2	99.56236899	0.641296262	0.017889249
ENSG00000133800	LYVE1	70.65394747	0.643768405	0.02556854
ENSG00000170396	ZNF804A	29.03622768	0.646355075	0.019853924
ENSG00000089692	LAG3	619.2571761	0.64650101	0.016353951
ENSG00000158423	RIBC1	34.51053604	0.656081053	0.012225883
ENSG00000105464	GRIN2D	35.49857268	0.659861409	0.034024579
ENSG00000101307	SIRPB1	15225.11426	0.660111513	0.038613532
ENSG00000240914	AL121612.1	29.13218451	0.660448235	0.049891429
ENSG00000163975	MELTF	44.58548244	0.677381849	0.034273278
ENSG00000233392	AC104809.2	626.5947749	0.680444895	0.02518366
ENSG00000252965	Y_RNA	36.2451722	0.681046085	0.027794078
ENSG00000203812	HIST2H2AA3	326.1351575	0.681924572	0.044928742
ENSG00000159640	ACE	125.4141709	0.68240224	0.017681666
ENSG00000164989	CCDC171	28.09860969	0.689213965	0.035528257
ENSG00000260810	AL135818.2	49.34068985	0.698265937	0.024570393
ENSG00000111728	ST8SIA1	197.6423215	0.711012514	0.028301449
ENSG00000125462	C1orf61	38.28056337	0.717909652	0.029805864
ENSG00000133661	SFTPD	33.496714	0.729432796	0.042939113
ENSG00000233750	CICP27	126.4387325	0.731200465	0.03078787
ENSG00000105246	EBI3	24.22196502	0.733927118	0.019730862
ENSG00000178878	APOLD1	115.4434656	0.734639399	0.003443725
ENSG00000164082	GRM2	59.23905201	0.737149883	0.015576694
ENSG00000272196	HIST2H2AA4	92.5033273	0.74682721	0.026441863
ENSG00000104883	PEX11G	74.36941311	0.753201165	0.049449373
ENSG00000272009	AL121944.1	19.75548667	0.757937984	0.04531994
ENSG00000189233	NUGGC	126.9845978	0.758783214	0.008592349
ENSG00000228834	AL445189.2	44.42643917	0.763543881	0.03923205
ENSG00000172927	MYEOV	66.52107388	0.799576746	0.038826678
ENSG00000237169	RPL12P27	79.75792952	0.810154301	0.003944289
ENSG00000151952	TMEM132D	48.95904326	0.816302518	0.034152059
ENSG00000198468	FLVCR1-AS1	173.4271921	0.816830804	0.049445451
ENSG00000237575	PYY2	18.75363229	0.819720375	0.049717978
ENSG00000227376	FTH1P16	17.9027667	0.828110438	0.010754168
ENSG00000169715	MT1E	90.45393515	0.836955483	0.006962775

ENSG00000237927	AL078604.2	48.42323612	0.84466275	0.047415593
ENSG00000233214	AC002511.2	118.583544	0.845724627	0.04704503
ENSG00000214867	SRSF9P1	18.93086939	0.852148347	0.029029756
ENSG00000218357	LINC01644	32.90295011	0.863509217	0.047353117
ENSG00000273032	DGCR9	32.0588605	0.868969044	0.011410649
ENSG00000197353	LYPD2	163.9595629	0.880642152	0.017038414
ENSG00000183570	PCBP3	202.0635901	0.883806986	0.02326779
ENSG00000173578	XCR1	55.14953649	0.890787254	0.039042874
ENSG00000176834	VSIG10	226.7856426	0.900314265	0.02947516
ENSG00000198883	PNMA5	29.01336372	0.904296319	0.019951154
ENSG00000262636	AC099489.2	24.90520726	0.913007673	0.032083222
ENSG00000198756	COLGALT2	140.8597228	0.914398288	0.005444544
ENSG00000240583	AQP1	114.8429521	0.923466048	0.029482046
ENSG00000234776	C11orf94	9.546177006	0.923795016	0.043229693
ENSG00000226469	ADAM1B	22.76123478	0.926380265	0.025047556
ENSG00000162551	ALPL	23823.46893	0.927345223	0.009280528
ENSG00000230734	RPL10P3	26.57623535	0.934650969	0.046537472
ENSG00000016391	CHDH	17.89101162	0.935734479	0.037925343
ENSG00000164850	GPER1	115.2320774	0.93697538	0.044931604
ENSG00000272690	LINC02018	27.074439	0.942604312	0.002744033
ENSG00000166736	HTR3A	32.03825129	0.944634922	0.044842926
ENSG00000256581	NLRP9P1	70.93936791	0.947772649	0.006932208
ENSG00000259182	AC019254.1	53.19408013	0.962163412	0.023873552
ENSG00000245888	FLJ21408	42.01388003	0.962544392	0.017262797
ENSG00000249896	LINC02495	10.42592547	0.963247853	0.030929889
ENSG00000223722	AC023157.1	102.3756215	0.973213697	0.018019115
ENSG00000278611	AC008567.3	14.7283207	0.988320125	0.044594102
ENSG00000232748	AC135050.1	17.81964718	0.996166358	0.019212377
ENSG00000234076	TPRG1-AS1	10.81279528	1.009686863	0.025869591
ENSG00000250486	FAM218A	12.76289773	1.034537553	0.047319736
ENSG00000237276	ANO7L1	78.35481972	1.038944794	0.02600384
ENSG00000170231	FABP6	41.19331895	1.042661317	0.024798435
ENSG00000166025	AMOTL1	111.8599945	1.042838578	0.047432924
ENSG00000223935	AC008074.1	28.22319707	1.045436501	0.014761138
ENSG00000233791	LINC01136	52.14546674	1.056977968	0.046387376
ENSG00000267197	AC011461.1	18.44495485	1.064623437	0.016550634
ENSG00000239300	AC080162.1	21.57070225	1.079773602	0.041266494
ENSG00000197766	CFD	246.6864385	1.087742569	0.03645212
ENSG00000235978	AC018816.1	19.88552238	1.093764547	0.001018759
ENSG00000182586	LINC00334	15.79901394	1.100793545	0.044250223
ENSG00000270972	AC136475.9	361.9737289	1.101497159	0.035760447
ENSG00000266274	RN7SL138P	25.87343295	1.102046063	0.015835166
ENSG00000238062	SPATA3-AS1	21.23489327	1.113229228	0.047179262
ENSG00000167992	VWCE	852.2292583	1.115816659	0.024408503
ENSG00000278985	AC092718.7	13.04750809	1.118899487	0.041979674
ENSG00000095739	BAMBI	18.03188484	1.119822285	0.025959543
ENSG00000099998	GGT5	111.3816437	1.120702894	0.008927848

ENSG00000247317	AC105202.1	15.06206748	1.133942241	0.020633673
ENSG00000255328	AC136475.5	251.1304095	1.137529495	0.038246489
ENSG00000261433	AC002347.1	7.557003828	1.140252596	0.038101255
ENSG00000235150	RCBTB2P1	14.94471894	1.150910735	0.023071059
ENSG00000253736	AC022217.3	16.1013279	1.156910425	0.003407129
ENSG00000270030	AC136475.7	28.40776091	1.169124007	0.033748613
ENSG00000277152	AC110048.2	28.10038009	1.181773358	0.025102537
ENSG00000279164	AL118508.3	20.08118009	1.186789825	0.01808249
ENSG00000250966	AC023886.2	9.342163714	1.186833431	0.012074457
ENSG00000229515	FLT1P1	97.54650186	1.193100186	0.006628064
ENSG00000250155	AC008957.1	48.51962503	1.193310494	0.000593244
ENSG00000225940	C5orf67	40.84526919	1.194909156	0.001199222
ENSG00000254017	IGHEP2	17.92361526	1.19789814	0.030780963
ENSG00000267496	FAM215A	12.13514483	1.225923172	0.023368614
ENSG00000223298	RNY3P8	16.34363378	1.231746268	0.041104278
ENSG00000272223	AL136304.1	13.00805018	1.234921912	0.012013396
ENSG00000143603	KCNN3	12.95580591	1.240336431	0.00897608
ENSG00000084710	EFR3B	45.69043671	1.245665652	0.011035548
ENSG00000240563	L1TD1	24.65648428	1.251393133	0.043601191
ENSG00000180071	ANKRD18A	17.42913891	1.275361578	0.028931508
ENSG00000274695	AC108704.1	11.42665395	1.28457389	0.034358187
ENSG00000241244	IGKV1D-16	53.91595039	1.293560859	0.038182467
ENSG00000282024	AL009031.1	29.72620737	1.313552035	0.019954768
ENSG00000189337	KAZN	429.8843051	1.318420531	0.026948097
ENSG00000229331	GK-IT1	27.4282255	1.322688422	0.007076679
ENSG00000266970	AC061992.1	14.52693786	1.327826264	0.009748874
ENSG00000130202	NECTIN2	506.9945399	1.329407544	0.006824897
ENSG00000278599	TBC1D3E	87.12672438	1.334312635	0.016674286
ENSG00000161055	SCGB3A1	48.23963777	1.355484383	0.04212921
ENSG00000235888	AF064858.1	13.88945565	1.361484448	0.003634016
ENSG00000197993	KEL	29.56691161	1.368247841	0.028380151
ENSG00000272541	AL021368.3	10.52127083	1.374546949	0.023283379
ENSG00000187244	BCAM	63.52637187	1.388937089	0.009498633
ENSG00000168917	SLC35G2	22.27307628	1.39838697	0.023786768
ENSG00000279641	AC120057.4	12.49159	1.409988916	0.004009299
ENSG00000275719	AC008622.2	8.118752709	1.410734936	0.018776654
ENSG00000240890	AC020633.1	14.43606905	1.422919102	0.008999518
ENSG00000237973	MTCO1P12	1545.069163	1.449219879	0.012585175
ENSG00000158865	SLC5A11	14.59811178	1.456195381	0.012910915
ENSG00000283294	AP005212.5	10.7234581	1.462019863	0.033052366
ENSG00000278635	AC141557.2	19.56053636	1.481664475	0.032467719
ENSG00000227189	AC092535.1	11.41364223	1.512594933	0.005621514
ENSG00000175445	LPL	42.9406978	1.570459754	0.014820557
ENSG00000214944	ARHGEF28	18.82643894	1.586952265	0.011982705
ENSG00000107731	UNC5B	31.51702769	1.664562922	0.004387605
ENSG00000279942	AL353597.3	130.492449	1.806605144	0.031057869
ENSG00000172348	RCAN2	16.66512973	1.832009694	0.019771089

ENSG00000204933	CD177P1	20.62550911	2.055862448	0.023265056
ENSG00000204110	LINC02520	50.66906735	2.156243639	0.012859797
ENSG00000224114	AL591846.1	73.99551896	2.325767964	0.046842991
ENSG00000204482	LST1	14.15695287	2.362896329	0.000422498
ENSG00000262902	MTCO1P40	11.98291798	2.676989822	0.000946296

MADRS

Gene ID	Gene Name	Base Mean	Regression Coefficient	p-value
ENSG00000262180	OCLM	10.12766313	-3.418086703	0.002916003
ENSG00000228028	AC069257.1	8.546826043	-3.360952058	0.008268987
ENSG00000257594	GALNT4	10.07790391	-3.282611277	0.00791509
ENSG00000270115	AL513327.3	9.816827726	-3.14863819	0.002496924
ENSG00000264188	AC106037.1	20.1895707	-3.103067804	0.000414522
ENSG00000100652	SLC10A1	14.25858923	-2.950764836	0.005023338
ENSG00000280355	AL132656.4	10.14664358	-2.938245165	0.022481625
ENSG00000274276	CBSL	24.26109755	-2.878198951	0.008993041
ENSG00000269937	AC093525.7	11.76060169	-2.801151981	0.005459622
ENSG00000259379	MTND5P32	12.2493515	-2.780589418	0.048932922
ENSG00000230289	AL358781.2	8.20024321	-2.773437384	0.004238696
ENSG00000226012	AP001434.1	11.34838494	-2.770609001	0.010079264
ENSG00000123500	COL10A1	8.683649247	-2.762065168	0.025192378
ENSG00000278881	FP325331.1	18.72001666	-2.666862497	0.004261037
ENSG00000260618	AC025917.1	37.80580841	-2.491920966	0.003828056
ENSG00000263627	PPP4R1-AS1	8.929610026	-2.48851791	0.013948644
ENSG00000114204	SERPINI2	16.54594333	-2.430380262	0.006421863
ENSG00000145623	OSMR	11.022553	-2.392383309	0.027698274
ENSG00000271580	AL583832.1	16.34154627	-2.371565378	0.002938075
ENSG00000279608	AL353795.3	10.63167457	-2.368926992	0.041279839
ENSG00000177427	MIEF2	17.88099623	-2.325990961	0.003781349
ENSG00000279430	AL590560.3	7.952713059	-2.324087408	0.035667996
ENSG00000241547	ACTG1P20	9.863185902	-2.297390204	0.039118617
ENSG00000250893	AC098869.2	15.56038099	-2.2791917	0.039620237
ENSG00000276593	AC022306.3	9.256436984	-2.271959431	0.039172337
ENSG00000213316	LTC4S	22.25597472	-2.269457816	0.010273859
ENSG00000180535	BHLHA15	12.50865427	-2.248384453	0.04466813
ENSG00000281195	AC007878.1	22.3595521	-2.242996117	0.028436048
ENSG00000235910	APOA1-AS	8.184228292	-2.194287963	0.037048906
ENSG00000062370	ZNF112	15.3626459	-2.192798187	0.006970881
ENSG00000258919	AL049836.1	12.0674799	-2.188550543	0.025622218
ENSG00000215267	AKR1C7P	10.02191353	-2.181438388	0.021864078
ENSG00000281183	NPTN-IT1	88.31188936	-2.179560183	0.001463
ENSG00000258725	PRC1-AS1	7.938186887	-2.124920004	0.046921495
ENSG00000257094	AC016957.1	14.70051025	-2.123347858	0.007880387
ENSG00000261208	AL365475.1	22.61732673	-2.099139557	0.036569256
ENSG00000235499	AC073046.1	36.14020358	-2.099056674	0.005418095
ENSG00000258824	AL122035.1	17.58432241	-2.07530226	0.01645802
ENSG00000256357	AL132708.1	8.067936464	-2.072973275	0.037630975
ENSG00000277224	HIST1H2BF	27.65230329	-2.043894347	0.006861733
ENSG00000236194	AC099811.1	14.60432294	-2.036674246	0.0280205
ENSG00000251229	AL645924.2	13.22413062	-2.019397444	0.032925004
ENSG00000223553	SMPD4P1	38.06869649	-2.005501542	0.007359848
ENSG00000185674	LYG2	6.207792536	-1.9923159	0.044741342

ENSG00000137809	ITGA11	12.02576999	-1.986409109	0.048338324
ENSG00000180447	GAS1	14.32498189	-1.978809594	0.034687032
ENSG00000279541	AC005261.6	11.88584372	-1.962317462	0.036949345
ENSG00000228477	AL663070.1	9.245292888	-1.957589179	0.047809974
ENSG00000276248	AL442125.1	30.6851303	-1.956198718	0.006006099
ENSG00000154274	C4orf19	29.10449247	-1.930699411	0.005181739
ENSG00000132464	ENAM	13.6350703	-1.92731429	0.040600742
ENSG00000252391	RNU6-638P	14.58176067	-1.91330792	0.042025059
ENSG00000120729	MYOT	11.89646066	-1.907362055	0.049294032
ENSG00000279412	AC020763.3	34.55463022	-1.903618654	0.021540697
ENSG00000104524	PYCR3	13.88648854	-1.902371193	0.044642066
ENSG00000266983	AC011444.1	11.4217424	-1.892923542	0.02197731
ENSG00000279356	AC007610.4	13.2831253	-1.878774533	0.048698433
ENSG00000181227	DLSTP1	17.44293129	-1.873344098	0.028030235
ENSG00000271725	AC103858.1	23.00878696	-1.866054245	0.0111578
ENSG00000211452	DIO1	20.64990681	-1.863804905	0.015440823
ENSG00000272416	AC025175.1	11.98023108	-1.860438391	0.035221674
ENSG00000160200	CBS	28.21026486	-1.854301202	0.013234456
ENSG00000258922	AC106028.2	11.60644685	-1.84935113	0.047485016
ENSG00000230191	AC006970.1	21.63621675	-1.845749974	0.046589381
ENSG00000178440	LINC00843	20.47001162	-1.845145479	0.021524895
ENSG00000233436	BTBD18	15.5606223	-1.844051158	0.014116379
ENSG00000230373	GOLGA6L5P	20.1890306	-1.8309531	0.029146237
ENSG00000253854	AC010834.3	37.78546627	-1.827154351	0.00485201
ENSG00000278817	AC007325.4	33.40391346	-1.821830305	0.004780892
ENSG00000263370	AC104564.2	17.27305823	-1.817137785	0.048002074
ENSG00000281530	AC004461.2	49.13147542	-1.815409846	0.006942863
ENSG00000235101	SETP9	13.06793432	-1.805850098	0.027580809
ENSG00000105855	ITGB8	43.60483977	-1.797478748	0.036819586
ENSG00000263786	AC022211.1	21.08875187	-1.76765693	0.028461111
ENSG00000005700	IBTK	94.72975234	-1.762447785	0.001578486
ENSG00000273306	AC018690.1	22.79680254	-1.726974159	0.003140828
ENSG00000276603	AL109614.1	21.10238472	-1.721466793	0.006853415
ENSG00000152527	PLEKHH2	32.93911986	-1.721060504	0.003540277
ENSG00000232334	AL683842.1	56.17903906	-1.708427015	0.020518673
ENSG00000232713	AC010733.1	29.84362803	-1.689270663	0.006140982
ENSG00000107249	GLIS3	32.39022722	-1.677820093	0.019594717
ENSG00000058335	RASGRF1	20.78286946	-1.666669178	0.01024579
ENSG00000248971	KRT8P46	46.86495509	-1.66369649	0.009505595
ENSG00000065328	MCM10	16.14765056	-1.661583607	0.04762084
ENSG00000280604	AJ239328.1	37.30223619	-1.645555858	0.005241809
ENSG00000007314	SCN4A	29.04275061	-1.641842158	0.010652398
ENSG00000211664	IGLV2-18	78.68057873	-1.638360083	0.021300668
ENSG00000273724	AC106782.5	32.08402996	-1.621427358	0.003742793
ENSG00000228434	AC004951.1	27.93013266	-1.619519755	0.020068618
ENSG00000279145	AC011912.1	31.14938137	-1.616540993	0.011784164
ENSG00000243659	FO393419.3	22.3891952	-1.611282506	0.016839656

ENSG00000232630	PRPS1P2	60.2121005	-1.611048964	0.005487109
ENSG00000251194	AL133330.1	41.86918574	-1.608886923	0.028912504
ENSG00000254756	AP001107.6	17.05656217	-1.605011037	0.030702888
ENSG00000239264	TXNDC5	17.03596607	-1.598608551	0.047701691
ENSG00000274751	AC120498.9	23.25636894	-1.59555005	0.020990144
ENSG00000198690	FAN1	31.10585787	-1.574546483	0.03650213
ENSG00000177103	DSCAML1	14.69070747	-1.563586657	0.0495953
ENSG00000231595	AC005224.1	19.8948004	-1.549211129	0.013398006
ENSG00000146122	DAAM2	208.8334161	-1.540418754	0.042827496
ENSG00000273812	BX640514.2	19.14196374	-1.53904298	0.038262421
ENSG00000111537	IFNG	26.93418748	-1.538982284	0.029483089
ENSG00000206965	RNU6-5P	25.92844726	-1.53178648	0.012887205
ENSG00000230756	RHOQP3	14.77806638	-1.529958265	0.047826305
ENSG00000279583	AC009086.3	18.73964818	-1.526087318	0.018472542
ENSG00000157999	ANKRD61	22.85017456	-1.524791008	0.025784744
ENSG00000274290	HIST1H2BE	39.75525424	-1.516420966	0.029462597
ENSG00000168852	TPTE2P5	19.05759601	-1.493038519	0.025666246
ENSG00000272402	AL031775.2	17.45656661	-1.489091282	0.02991618
ENSG00000221937	TAS2R40	26.6048154	-1.475349285	0.045128801
ENSG00000234911	TEX21P	35.61560322	-1.475010138	0.019880162
ENSG00000230562	FAM133DP	25.61916506	-1.464369744	0.007733046
ENSG00000102098	SCML2	40.62101919	-1.448455229	0.003181803
ENSG00000182327	GLTPD2	19.27284145	-1.433049179	0.032931741
ENSG00000253559	OSGEPL1-AS1	19.7803518	-1.425305784	0.041854825
ENSG00000267385	AC011498.4	42.92172982	-1.408500984	0.024851165
ENSG00000188211	NCR3LG1	81.61188721	-1.406711536	0.048168565
ENSG00000184361	SPATA32	37.71671292	-1.39877177	0.008082792
ENSG00000284713	AP003071.5	22.42406632	-1.397835436	0.03307678
ENSG00000248124	RRN3P1	82.83546591	-1.395051041	0.005136235
ENSG00000197385	ZNF860	87.9845777	-1.38757614	0.001608827
ENSG00000233672	RNASEH2B-AS1	19.57888334	-1.38420012	0.034711357
ENSG00000267274	AC008770.4	31.4825329	-1.373242029	0.048336309
ENSG00000238018	AC093110.1	46.1463646	-1.368517914	0.013626142
ENSG00000260588	AC027702.1	22.01431668	-1.346342232	0.03665738
ENSG00000269843	AC008537.2	30.76680716	-1.340352903	0.034847674
ENSG00000143702	CEP170	71.18633211	-1.327174532	0.016608293
ENSG00000255557	AP001266.2	25.57006685	-1.319873607	0.043613815
ENSG00000101280	ANGPT4	25.44307981	-1.309512402	0.026990516
ENSG00000092853	CLSPN	31.21431714	-1.30689744	0.027495227
ENSG00000279617	AC005796.1	16.28511008	-1.301111787	0.039566254
ENSG00000258181	AC008083.3	26.72153001	-1.291031097	0.042179725
ENSG00000279933	AL031595.1	32.53835816	-1.290609766	0.030382107
ENSG00000242622	AC092910.3	57.41795047	-1.285087465	0.01174228
ENSG00000256706	AC005342.2	33.99151591	-1.267336251	0.006966348
ENSG00000230454	U73166.1	32.90389661	-1.251376677	0.047168138
ENSG00000268170	AC073342.2	99.87929584	-1.244867838	0.026421488
ENSG00000091622	PITPNM3	33.14630292	-1.236801706	0.042809374

ENSG00000132259	CNGA4	25.35007119	-1.233851495	0.042056032
ENSG00000124251	TP53TG5	44.98267913	-1.222876384	0.034186903
ENSG00000233885	YEATS2-AS1	44.95183704	-1.208598499	0.011543061
ENSG00000002745	WNT16	43.88382974	-1.19479914	0.03307716
ENSG00000177932	ZNF354C	156.9074627	-1.192850406	0.006022845
ENSG00000232254	CSF2RBP1	96.4124058	-1.187193785	0.002778189
ENSG00000270168	AC004233.2	30.53236702	-1.162072578	0.032372039
ENSG00000272693	AC073107.1	28.71271822	-1.161255068	0.03080181
ENSG00000232104	RFX3-AS1	40.30264779	-1.161172299	0.037854967
ENSG00000121690	DEPDC7	56.72277861	-1.157893083	0.003483413
ENSG00000164114	MAP9	100.2970703	-1.155341559	0.020458936
ENSG00000118276	B4GALT6	67.92803202	-1.14596251	0.024337153
ENSG00000152782	PANK1	43.94929165	-1.144810276	0.022706785
ENSG00000280035	AC011676.5	60.3916332	-1.139376108	0.015621231
ENSG00000256673	AC141557.1	32.00224676	-1.133323505	0.033258827
ENSG00000170954	ZNF415	40.38407417	-1.127952067	0.035768315
ENSG00000075223	SEMA3C	202.9632559	-1.119352339	0.018923564
ENSG00000258682	AL132989.1	91.74917802	-1.080943508	0.024647673
ENSG00000238005	AL391832.2	156.4291636	-1.075633437	0.010796978
ENSG00000147536	GINS4	60.8286493	-1.064075095	0.002664309
ENSG00000248734	AC008906.1	67.70964487	-1.061902952	0.038374668
ENSG00000267355	AC022966.1	35.72146751	-1.054577367	0.046073222
ENSG00000121769	FABP3	46.22089377	-1.041349588	0.011494545
ENSG00000220804	LINC01881	54.75233426	-1.022735319	0.01407249
ENSG00000260467	AC018552.2	228.2458713	-1.021803668	0.013082117
ENSG00000247950	SEC24B-AS1	38.50983095	-1.017314551	0.038608704
ENSG00000211653	IGLV1-40	111.0586101	-1.014570721	0.012982379
ENSG00000214140	PRCD	67.03984418	-1.00681932	0.012355582
ENSG00000215244	AL137145.2	180.0143804	-1.003555305	3.91E-05
ENSG00000228340	MIR646HG	154.1962487	-0.989595227	0.013283921
ENSG00000205918	PDPK2P	84.5135111	-0.981979721	0.016561658
ENSG00000225963	AC009950.1	349.6390973	-0.948922526	0.04685952
ENSG00000123219	CENPK	191.0361825	-0.941738949	0.045645048
ENSG00000235316	DUSP8P5	79.8036706	-0.938438264	0.037242882
ENSG00000125531	FNDC11	75.49347326	-0.937615512	0.018294207
ENSG00000253981	ALG1L13P	107.743223	-0.932356461	0.015303303
ENSG00000106714	CNTNAP3	760.5874355	-0.927215475	0.007303372
ENSG00000138036	DYNC2LI1	66.55945663	-0.922469285	0.021627834
ENSG00000198707	CEP290	353.1406599	-0.911455738	0.013004147
ENSG00000114346	ECT2	190.5516755	-0.906376155	0.022307219
ENSG00000273599	AL731571.1	181.8961317	-0.899863067	0.03787937
ENSG00000248323	LUCAT1	1512.671993	-0.886995208	0.033278938
ENSG00000211640	IGLV6-57	249.9272966	-0.886962332	0.021211347
ENSG00000207561	MIR635	66.35262017	-0.88267123	0.034294894
ENSG00000211802	TRAV22	84.8963145	-0.882220852	0.010075359
ENSG00000198146	ZNF770	866.4660104	-0.873715097	0.009343472
ENSG00000183822	NCF4-AS1	52.54435032	-0.870053921	0.038256298

ENSG00000176659	C20orf197	126.9572232	-0.868475045	0.038482791
ENSG00000246575	AC093162.2	528.9009937	-0.868001332	0.039569775
ENSG00000232284	GNG12-AS1	50.10832566	-0.861173858	0.03945946
ENSG00000268575	AL031282.2	125.3663411	-0.833615447	0.035345606
ENSG00000113638	TTC33	318.579548	-0.828694275	0.020738176
ENSG00000235958	UBOX5-AS1	49.86885832	-0.828545359	0.028905307
ENSG00000064932	SBNO2	1284.555991	-0.811486839	0.018467426
ENSG00000260698	AL591848.3	82.37865508	-0.807886997	0.039389389
ENSG00000224614	TNK2-AS1	102.3811878	-0.802506937	0.025252829
ENSG00000175768	TOMM5	58.30497093	-0.80213627	0.041846103
ENSG00000233527	ZNF529-AS1	96.59567353	-0.793134872	0.018776174
ENSG00000102908	NFAT5	2022.963314	-0.772265505	0.039407282
ENSG00000174405	LIG4	568.8654436	-0.767579526	0.047233841
ENSG00000181690	PLAG1	214.7527968	-0.760182755	0.031667909
ENSG00000204304	PBX2	288.2524289	-0.756057143	0.02632877
ENSG00000214106	PAXIP1-AS2	191.6003932	-0.755238221	0.042871425
ENSG00000166398	KIAA0355	559.0053746	-0.750494704	0.035283448
ENSG00000103154	NECAB2	746.4325668	-0.744924995	0.031897893
ENSG00000155744	FAM126B	3865.326698	-0.726892099	0.026491761
ENSG00000164187	LMBRD2	711.103789	-0.726520973	0.028059541
ENSG00000066651	TRMT11	241.6638512	-0.722160211	0.024239475
ENSG00000213073	AL353625.1	521.3443585	-0.715325546	0.031529315
ENSG00000173889	PHC3	1998.903171	-0.714572511	0.046031798
ENSG00000212743	AL137145.1	120.4644438	-0.706271711	0.036410556
ENSG00000182885	ADGRG3	16042.75561	-0.670625873	0.031890018
ENSG00000230551	AC021078.1	853.2544205	-0.657062049	0.033742613
ENSG00000248996	AC145098.1	1245.761222	-0.651224863	0.046914232
ENSG00000236438	FAM157A	1419.453897	-0.644627598	0.032666491
ENSG00000135960	EDAR	303.9603979	-0.638168301	0.009000121
ENSG00000188227	ZNF793	221.3487781	-0.631870857	0.042393516
ENSG00000184557	SOCS3	3267.552673	-0.60339408	0.01535406
ENSG00000211666	IGLV2-14	1318.219767	-0.596517428	0.013375283
ENSG00000129116	PALLD	244.2940454	-0.593024346	0.044338343
ENSG00000253106	AC090198.1	165.1119822	-0.579786512	0.036065726
ENSG00000124549	BTN2A3P	166.6829289	-0.55753992	0.027144913
ENSG00000203666	EFCAB2	216.3164556	-0.536619877	0.04359976
ENSG00000163534	FCRL1	1749.851093	-0.535353696	0.039089054
ENSG00000183762	KREMEN1	2792.205045	-0.517687594	0.038841519
ENSG00000251247	ZNF345	327.5269836	-0.488431754	0.049132655
ENSG00000265666	RARA-AS1	646.5682209	-0.482349355	0.04357455
ENSG00000114904	NEK4	537.4001497	-0.467589063	0.040863337
ENSG00000197619	ZNF615	365.8381741	-0.458724163	0.048938924
ENSG00000196967	ZNF585A	387.0113172	-0.448958741	0.038449189
ENSG00000087157	PGS1	6583.709162	-0.412030125	0.030705675
ENSG00000185219	ZNF445	1987.738491	-0.410727106	0.020496839
ENSG00000136940	PDCL	691.7029417	-0.385611529	0.024393255
ENSG00000198960	ARMCX6	611.7810975	0.380638524	0.025775562

ENSG00000049449	RCN1	598.3146158	0.388977802	0.043953274
ENSG00000170260	ZNF212	1270.102748	0.389845705	0.012901096
ENSG00000279884	AC010894.5	554.3754521	0.402924429	0.042771776
ENSG00000100372	SLC25A17	719.7294862	0.415250358	0.037478575
ENSG00000196914	ARHGEF12	1168.333583	0.430200305	0.038751681
ENSG00000114473	IQCG	220.2088672	0.430498931	0.044218441
ENSG00000172006	ZNF554	284.6725781	0.437851116	0.049811108
ENSG00000149218	ENDOD1	964.0961741	0.441713201	0.015518204
ENSG00000169398	PTK2	472.9972521	0.455170569	0.04080771
ENSG00000134297	PLEKHA8P1	239.2899869	0.456219546	0.04617401
ENSG00000099284	H2AFY2	246.6118719	0.458743445	0.048352656
ENSG00000136463	TACO1	699.5119901	0.459817238	0.040675068
ENSG00000156804	FBXO32	1010.481509	0.464987711	0.024209738
ENSG00000182518	FAM104B	227.5080281	0.474906888	0.044532402
ENSG00000066926	FECH	1362.23167	0.475478182	0.027873664
ENSG00000127922	SEM1	414.0448934	0.494589407	0.038652258
ENSG00000143507	DUSP10	350.5475133	0.497668264	0.034942941
ENSG00000221926	TRIM16	185.8949037	0.506537603	0.033858003
ENSG00000185986	SDHAP3	229.1002069	0.51600349	0.031221277
ENSG00000149485	FADS1	647.1658718	0.518814481	0.025589961
ENSG00000109534	GAR1	649.4383404	0.52184554	0.021812006
ENSG00000120129	DUSP1	16794.93657	0.532505561	0.019011235
ENSG00000168785	TSPAN5	2233.05023	0.532943597	0.015577544
ENSG00000112578	BYSL	410.9333913	0.540160645	0.004554731
ENSG00000154917	RAB6B	151.928857	0.547434208	0.038717653
ENSG00000184221	OLIG1	1401.564037	0.552947865	0.025416833
ENSG00000169397	RNASE3	336.7563732	0.567164361	0.041423859
ENSG00000229754	CXCR2P1	1292.54045	0.567317401	0.033684314
ENSG00000269973	AC010969.2	216.0247873	0.570601818	0.031015982
ENSG00000138735	PDE5A	335.1180145	0.576096804	0.037462805
ENSG00000165914	TTC7B	308.9129163	0.57976758	0.021490864
ENSG00000123427	EEF1AKMT3	176.082083	0.592435874	0.02829094
ENSG00000065534	MYLK	410.3888366	0.60479785	0.030545336
ENSG00000049759	NEDD4L	322.0048152	0.606254851	0.036824773
ENSG00000242732	RTL5	265.4024036	0.606496742	0.031782822
ENSG00000100298	APOBEC3H	154.5289897	0.626733645	0.039512949
ENSG00000072195	SPEG	388.68336	0.647227453	0.030215675
ENSG00000095397	WHRN	336.6931058	0.647824869	0.030496487
ENSG00000261662	AL359752.1	166.1040528	0.653759161	0.030087725
ENSG00000158292	GPR153	163.088413	0.659566605	0.049610906
ENSG00000119705	SLIRP	226.4901348	0.66068593	0.011083872
ENSG00000182253	SYNM	123.8961186	0.67033774	0.033141352
ENSG00000196565	HBG2	13041.09831	0.67274568	0.025379823
ENSG00000264577	AC010761.1	90.74400327	0.674064242	0.043409259
ENSG00000167768	KRT1	1540.701664	0.678142674	0.031799067
ENSG00000136842	TMOD1	348.7151286	0.679697796	0.027665473
ENSG00000238243	OR2W3	615.7634529	0.694682472	0.017665148

ENSG00000251136	AF117829.1	123.6252482	0.704207193	0.021110747
ENSG00000157514	TSC22D3	62233.2147	0.70645136	0.000472417
ENSG00000082146	STRADB	8959.25145	0.729987593	0.002775273
ENSG00000278828	HIST1H3H	917.2629981	0.730696437	0.015345303
ENSG00000260228	AC009119.1	74.90944229	0.733755252	0.022302232
ENSG00000254614	AP003068.2	346.6391902	0.734389461	0.005580266
ENSG00000047597	XK	286.2659247	0.743389184	0.008659809
ENSG00000162367	TAL1	1306.733323	0.744204839	0.006094961
ENSG00000047648	ARHGAP6	87.07858072	0.744551463	0.044392362
ENSG00000198513	ATL1	60.23608883	0.7447883	0.043064486
ENSG00000104518	GSDMD	483.0866264	0.746583319	0.033712822
ENSG00000119326	CTNNAL1	157.4676921	0.747414303	0.015591989
ENSG00000270696	AC005034.3	202.2556666	0.749121451	0.004290546
ENSG00000175518	UBQLNL	87.56379891	0.749292226	0.041986148
ENSG00000255422	AP002954.1	72.84278755	0.753447168	0.019107269
ENSG00000005471	ABCB4	162.7377995	0.756048535	0.028979137
ENSG00000243064	ABCC13	306.037995	0.760098694	0.041977186
ENSG00000103044	HAS3	109.4476273	0.762034451	0.007105092
ENSG00000004939	SLC4A1	14929.72318	0.767863553	0.023350654
ENSG00000185630	PBX1	311.2270924	0.779283849	0.01006964
ENSG00000141252	VPS53	259.6346887	0.78631672	0.017363904
ENSG00000273117	AC144652.1	98.47719132	0.791832696	0.008000173
ENSG00000266904	LINC00663	147.853657	0.792521015	0.014319532
ENSG00000105507	CABP5	107.8515884	0.795957334	0.020979109
ENSG00000204947	ZNF425	99.35081744	0.808799346	0.008477966
ENSG00000204872	NAT8B	93.23692253	0.810506754	0.034922872
ENSG00000225460	AL590233.1	81.88303804	0.813768818	0.032713617
ENSG00000149516	MS4A3	405.2107097	0.817349785	0.025401291
ENSG00000204010	IFIT1B	477.02136	0.824610923	0.016340646
ENSG00000177465	ACOT4	72.47421929	0.825778492	0.016835845
ENSG00000259250	AC018904.1	129.6417445	0.840135886	0.002812538
ENSG00000260101	AC008074.3	58.05511419	0.842011647	0.029316754
ENSG00000197465	GYPE	76.54000182	0.873056653	0.021531851
ENSG00000242445	RPL7AP11	46.88565082	0.87944954	0.036751275
ENSG00000185338	SOCS1	438.16997	0.896952486	0.021568179
ENSG00000164821	DEFA4	440.7990706	0.897234402	0.005989257
ENSG00000170412	GPRC5C	55.66061941	0.926273809	0.038078154
ENSG00000211667	IGLV3-12	98.8945107	0.936099103	0.039213412
ENSG00000164082	GRM2	55.3070003	0.94224018	0.038057379
ENSG00000187244	BCAM	66.26811487	0.947030871	0.040708095
ENSG00000087884	AAMDC	62.05384813	0.949431676	0.015561487
ENSG00000206047	DEFA1	338.4112289	0.952931081	0.009112747
ENSG00000166033	HTRA1	45.11159874	0.954820193	0.040528033
ENSG00000236830	CBR3-AS1	50.19204916	0.956439465	0.011346834
ENSG00000170835	CEL	46.71402651	0.957358562	0.037664628
ENSG00000179094	PER1	2516.978218	0.963778249	0.001264418
ENSG00000151491	EPS8	102.6946251	0.965270503	0.034220435

ENSG00000224177	LINC00570	219.3156536	1.001731353	0.003418908
ENSG00000282608	ADORA3	431.1475726	1.009048925	0.003325294
ENSG00000240914	AL121612.1	31.45135821	1.009049054	0.045704542
ENSG00000166831	RBPMS2	46.97243492	1.009314247	0.03205491
ENSG00000267871	AC005261.1	54.81710619	1.018033479	0.006940496
ENSG00000275772	AC244157.2	61.37736487	1.027096888	0.027058433
ENSG00000189238	LINC00943	46.13151961	1.035621218	0.037263732
ENSG00000280047	AC091825.1	100.5196548	1.042219077	0.006506123
ENSG00000231369	Z97353.1	32.73833722	1.042685126	0.037005961
ENSG00000168004	HRASLS5	66.94362283	1.053129036	0.023981332
ENSG00000277218	AL139123.1	29.34695994	1.057253568	0.033157414
ENSG00000272799	AC006238.1	37.60920056	1.062829641	0.012698346
ENSG00000145423	SFRP2	120.219956	1.07503622	0.046781253
ENSG00000171189	GRIK1	29.97186209	1.126987057	0.03463052
ENSG00000272338	AC067838.1	48.13680812	1.127163376	0.022130763
ENSG00000198680	TUSC1	31.99222202	1.138496676	0.017545365
ENSG00000266401	AP002478.1	37.31576377	1.143786882	0.037127588
ENSG00000165917	RAPSN	58.73742914	1.155421735	0.015788118
ENSG00000276517	AL133243.3	34.49144772	1.161078811	0.011465076
ENSG00000234902	AC007879.3	30.20115893	1.179772017	0.035207527
ENSG00000273619	AL121832.2	42.77264894	1.180538376	0.012191789
ENSG00000133116	KL	27.58149907	1.183496129	0.038766287
ENSG00000203780	FANK1	28.43787642	1.192772819	0.02783105
ENSG00000116096	SPR	82.93657869	1.197623247	0.001359245
ENSG00000196188	CTSE	42.58848105	1.198715569	0.029463084
ENSG00000124780	KCNK17	51.78239821	1.201707306	0.007894475
ENSG00000186081	KRT5	60.09303494	1.210126506	0.022154808
ENSG00000180815	MAP3K15	29.37574662	1.214724912	0.02139202
ENSG00000140022	STON2	126.9894009	1.226562665	0.000546196
ENSG00000198848	CES1	182.9582534	1.230355241	0.004127646
ENSG00000267934	AC010300.1	25.21387773	1.248542373	0.037543428
ENSG00000102904	TSNAXIP1	26.18968039	1.254781839	0.019795509
ENSG00000237989	LINC01679	48.45188539	1.255970467	0.008893125
ENSG00000153531	ADPRHL1	38.09420086	1.271264548	0.018526578
ENSG00000273628	AL354798.1	15.78420886	1.291578161	0.033461235
ENSG00000276968	AL158196.1	23.5395713	1.293957372	0.035220209
ENSG00000179914	ITLN1	66.67710374	1.294328085	0.001413848
ENSG00000170421	KRT8	20.60627481	1.30546018	0.046289825
ENSG00000263823	AC009831.1	31.72753387	1.31115065	0.01664724
ENSG00000167306	MYO5B	34.18464741	1.320580456	0.019475817
ENSG00000197279	ZNF165	27.50462096	1.325822617	0.022636022
ENSG00000266601	AC018521.6	33.24456814	1.33103144	0.005727683
ENSG00000163923	RPL39L	50.57310296	1.337885714	0.027681889
ENSG00000262636	AC099489.2	20.38714576	1.347339146	0.039026546
ENSG00000270084	GAS5-AS1	22.65774076	1.357649039	0.036189159
ENSG00000279265	AC000123.2	35.41783187	1.365223272	0.01354018
ENSG00000153558	FBXL2	46.74409673	1.365800975	0.012365063

ENSG00000261187	AC079322.1	23.21149936	1.398987269	0.018146266
ENSG00000214357	NEURL1B	27.10085496	1.404992697	0.032207459
ENSG00000006704	GTF2IRD1	15.13550891	1.425849421	0.036625164
ENSG00000283317	AL831711.1	27.12608148	1.433688858	0.015604156
ENSG00000272009	AL121944.1	22.70940002	1.434860457	0.034472982
ENSG00000172572	PDE3A	14.32621935	1.447135751	0.045297066
ENSG00000283399	AC004381.2	32.27254825	1.484847479	0.037431286
ENSG00000250471	GMPSP1	20.92229564	1.485007659	0.013346373
ENSG00000149201	CCDC81	16.05949667	1.487951776	0.04709634
ENSG00000272902	TBC1D8-AS1	14.80255807	1.495318461	0.039883122
ENSG00000283236	AC074141.1	41.78728994	1.50950144	0.007154021
ENSG00000261097	LINC00563	18.88020204	1.512735561	0.037021165
ENSG00000234705	HMGA1P4	17.97261078	1.515443545	0.040124015
ENSG00000235001	EIF4A1P2	33.81299804	1.525642722	0.009464612
ENSG00000235478	LINC01664	13.77122555	1.535577259	0.045784952
ENSG00000157554	ERG	26.00491508	1.540080899	0.022732362
ENSG00000189253	TRIM64B	32.0644654	1.540364983	0.042930548
ENSG00000183742	MACC1	23.91179322	1.546059145	0.044154449
ENSG00000130037	KCNA5	14.50309697	1.548786576	0.042736137
ENSG00000065618	COL17A1	36.03297275	1.566418849	0.006442557
ENSG00000211454	AKR7L	12.73378732	1.568021832	0.041093808
ENSG00000258521	AL157871.2	27.02359887	1.568793674	0.009564059
ENSG00000229785	SLC25A38P1	14.17478569	1.573870386	0.039286967
ENSG00000284052	AC006460.2	22.5932895	1.599779275	0.020530395
ENSG00000274191	AC026333.4	13.75151366	1.610636391	0.034686345
ENSG00000275484	AP003419.4	24.40242093	1.64165441	0.021522293
ENSG00000155066	PROM2	14.51946402	1.665982055	0.012143753
ENSG00000118473	SGIP1	21.00398441	1.685543473	0.025261111
ENSG00000273483	AL354760.1	12.10081386	1.687029195	0.018495183
ENSG00000221955	SLC12A8	14.28566705	1.694576736	0.024380105
ENSG00000163885	CFAP100	13.19314729	1.699242768	0.015868381
ENSG00000150672	DLG2	14.3393169	1.704192332	0.026775277
ENSG00000113645	WWC1	26.54493371	1.716675089	0.003131341
ENSG00000272163	AF106564.1	11.15085935	1.726609913	0.049427915
ENSG00000237575	PYY2	17.43240338	1.72984158	0.034932296
ENSG00000217702	AC073263.1	12.13458584	1.739603613	0.047681129
ENSG00000130656	HBZ	98.64585819	1.776533358	0.012201404
ENSG00000140534	TICRR	13.11480955	1.784871165	0.026300272
ENSG00000127252	HRASLS	14.2477804	1.790934409	0.030036959
ENSG00000197993	KEL	25.38376008	1.814856606	0.030990144
ENSG00000118939	UCHL3	17.00678459	1.830672064	0.035942625
ENSG00000130023	ERMARD	107.6250819	1.85495271	0.014988511
ENSG00000275719	AC008622.2	7.901438	1.858861579	0.047592402
ENSG00000109321	AREG	31.46733587	1.862953954	0.046581212
ENSG00000223711	AC069213.1	18.23772988	1.875017031	0.009182078
ENSG00000270194	AC097359.2	13.10602261	1.892854044	0.04101691
ENSG00000206557	TRIM71	9.67980565	1.909965825	0.022553239

ENSG00000172348	RCAN2	17.74955232	1.914587761	0.025789284
ENSG00000239203	AC093484.1	15.10196783	1.930501809	0.03012121
ENSG00000254502	AP003097.1	9.967130479	1.934552333	0.034022063
ENSG00000165996	HACD1	27.66130098	1.936009243	0.000306361
ENSG00000226686	LINC01535	9.426509122	1.949191744	0.039014741
ENSG00000170180	GYPA	29.30206582	1.957804033	0.009541718
ENSG00000225992	TRGVA	13.59346438	1.988029769	0.007398392
ENSG00000121594	CD80	16.30876057	1.999096102	0.012963125
ENSG00000182057	OGFRP1	21.66324966	2.003217095	0.001534589
ENSG00000260082	AC106886.1	9.781949697	2.011137708	0.028699218
ENSG00000262482	AC004034.1	12.18343255	2.03168877	0.037058284
ENSG00000257557	PPP1R12A-AS1	13.76724709	2.098434144	0.014284718
ENSG00000183722	LHFPL6	10.4776609	2.111944225	0.02304623
ENSG00000258659	TRIM34	14.04807362	2.142146449	0.009167226
ENSG00000274736	CCL23	25.7867236	2.159836754	0.002733176
ENSG00000275763	C18orf65	13.53017753	2.163253117	0.008947303
ENSG00000125845	BMP2	10.16063454	2.183369524	0.021002116
ENSG00000264968	AC090844.2	20.61065043	2.201227966	0.00192505
ENSG00000164684	ZNF704	8.537695439	2.213206389	0.037995924
ENSG00000258469	CHMP4BP1	50.36435517	2.238612217	0.006775824
ENSG00000258534	AL132712.1	13.60741125	2.242829684	0.029012919
ENSG00000254593	OR7E126P	10.50697172	2.245270014	0.044878553
ENSG00000122870	BICC1	10.29643756	2.28676095	0.010780586
ENSG00000143816	WNT9A	9.978384183	2.307766566	0.037371488
ENSG00000273139	AC007663.3	8.386398985	2.324298979	0.013687319
ENSG00000271938	AC103724.4	9.18848197	2.340699483	0.025101947
ENSG00000261448	AC109446.3	10.08080699	2.35065396	0.028831951
ENSG00000124564	SLC17A3	11.76890835	2.37589379	0.009823396
ENSG00000158865	SLC5A11	14.65628692	2.391640227	0.013091953
ENSG00000198576	ARC	9.531038961	2.415655488	0.006604033
ENSG00000204394	VARS	32.06626838	2.445809578	0.002660419
ENSG00000211884	TRAJ5	8.649184198	2.50358209	0.032911442
ENSG00000248719	AC021127.1	8.04053181	2.514040445	0.034865738
ENSG00000189045	ANKDD1B	15.09165016	2.515025208	0.010827443
ENSG00000077935	SMC1B	15.83418691	2.519135742	0.004172445
ENSG00000085552	IGSF9	10.59884805	2.522148074	0.008044344
ENSG00000166823	MESP1	12.60621619	2.530526743	0.013029757
ENSG00000223901	AP001469.1	8.161894986	2.546558036	0.021886355
ENSG00000105865	DUS4L	10.39239889	2.672023355	0.005571679
ENSG00000242220	TCP10L	9.712899575	2.806498825	0.005236929
ENSG00000105997	HOXA3	7.418815671	2.808490782	0.018181627
ENSG00000271967	AL583856.2	12.76749818	2.901220304	0.001927124
ENSG00000278978	AC092611.3	10.46148567	2.968613876	0.003471012
ENSG00000223929	MIR4432HG	10.15138831	3.521992404	0.005631628
