# Supplementary material for: Whole blood transcriptional signatures associated with rapid antidepressant response to ketamine in patients with treatment resistant depression
Source: Transl Psychiatry. 2022 Jan 10;12:12. doi: 10.1038/s41398-021-01712-0 (PMC8748646; doi:10.1038/s41398-021-01712-0)
Supplement: Supplementary file 4 — Supplementary file 2 [file 41398_2021_1712_MOESM4_ESM.pdf]

| Gene_ID | Gene_Name | Module |
|---|---|---|
| ENSG00000005379 | FGR | black |
| ENSG00000007264 | NIPAL3 | black |
| ENSG00000007350 | CYP51A1 | black |
| ENSG00000020633 | RAD52 | black |
| ENSG00000026751 | CD99 | black |
| ENSG00000067840 | SNX11 | black |
| ENSG00000073861 | DBNDD1 | black |
| ENSG00000074590 | RBM5 | black |
| ENSG00000075234 | POLDIP2 | black |
| ENSG00000099889 | AK2 | black |
| ENSG00000100385 | FKBP4 | black |
| ENSG00000100427 | RBM6 | black |
| ENSG00000100450 | NDUFAB1 | black |
| ENSG00000100453 | SLC22A16 | black |
| ENSG00000104490 | ST7 | black |
| ENSG00000105370 | UPF1 | black |
| ENSG00000105374 | SLC25A5 | black |
| ENSG00000106018 | MEOX1 | black |
| ENSG00000108370 | FAM214B | black |
| ENSG00000109956 | COPZ2 | black |
| ENSG00000112796 | TSPOAP1 | black |
| ENSG00000115523 | WDR54 | black |
| ENSG00000116667 | KMT2E | black |
| ENSG00000117281 | IBTK | black |
| ENSG00000117560 | C19orf60 | black |
| ENSG00000122223 | AP2B1 | black |
| ENSG00000125864 | FARP2 | black |
| ENSG00000132718 | PAF1 | black |
| ENSG00000133067 | PNPLA4 | black |
| ENSG00000134539 | CCDC124 | black |
| ENSG00000135362 | GAS7 | black |
| ENSG00000136213 | TRAPPC6A | black |
| ENSG00000137441 | MATK | black |
| ENSG00000137824 | CD79B | black |
| ENSG00000137843 | ST7L | black |
| ENSG00000141622 | TKTL1 | black |
| ENSG00000141736 | LUC7L | black |
| ENSG00000141756 | TSR3 | black |
| ENSG00000145107 | PSMB1 | black |
| ENSG00000145649 | MPND | black |
| ENSG00000145990 | MGST1 | black |
| ENSG00000148019 | PGLYRP1 | black |
| ENSG00000149294 | MMP25 | black |
| ENSG00000149557 | IL32 | black |
| ENSG00000150045 | MAPK8IP2 | black |
| ENSG00000150687 | MED24 | black |
| ENSG00000151715 | RPS20 | black |

| | | |
|---|---|---|
| ENSG00000154734 | TENM1 | black |
| ENSG00000154760 | TRAF3IP3 | black |
| ENSG00000156453 | METTL13 | black |
| ENSG00000156475 | DYRK4 | black |
| ENSG00000156966 | UQCRC1 | black |
| ENSG00000158292 | CD4 | black |
| ENSG00000159618 | HFE | black |
| ENSG00000159674 | FYN | black |
| ENSG00000159733 | LYPLA2 | black |
| ENSG00000160318 | BTBD7 | black |
| ENSG00000160856 | CEP68 | black |
| ENSG00000162383 | CD22 | black |
| ENSG00000162398 | ALOX5 | black |
| ENSG00000163106 | EHD3 | black |
| ENSG00000163508 | MAN2B2 | black |
| ENSG00000163564 | MVP | black |
| ENSG00000164483 | MTMR11 | black |
| ENSG00000164929 | COX15 | black |
| ENSG00000166289 | CCDC88C | black |
| ENSG00000167723 | WAS | black |
| ENSG00000167912 | BID | black |
| ENSG00000168229 | XYLT2 | black |
| ENSG00000169583 | RGPD5 | black |
| ENSG00000169744 | CHDH | black |
| ENSG00000170298 | RUFY3 | black |
| ENSG00000170962 | MARCO | black |
| ENSG00000171101 | CD74 | black |
| ENSG00000171476 | RUNX3 | black |
| ENSG00000171596 | OSBPL5 | black |
| ENSG00000172031 | RTFDC1 | black |
| ENSG00000172543 | ZNF839 | black |
| ENSG00000173068 | ALAS1 | black |
| ENSG00000174348 | GLRX2 | black |
| ENSG00000176485 | ABCC2 | black |
| ENSG00000176720 | PLEKHO1 | black |
| ENSG00000177465 | NCAPH2 | black |
| ENSG00000180644 | TOMM34 | black |
| ENSG00000180739 | KCNG1 | black |
| ENSG00000181036 | SLAMF7 | black |
| ENSG00000182534 | B4GALT7 | black |
| ENSG00000184349 | SH2D2A | black |
| ENSG00000189350 | TNFRSF1B | black |
| ENSG00000196139 | SLC39A9 | black |
| ENSG00000196169 | HMGB3 | black |
| ENSG00000197057 | BAK1 | black |
| ENSG00000198574 | GRN | black |
| ENSG00000198821 | ARHGAP31 | black |
| ENSG00000198885 | SARS | black |

| | | |
|---|---|---|
| ENSG00000205221 | EIPR1 | black |
| ENSG00000205336 | ALG1 | black |
| ENSG00000211688 | ABCF2 | black |
| ENSG00000211793 | CHPF2 | black |
| ENSG00000211825 | APBA2 | black |
| ENSG00000211829 | TMSB10 | black |
| ENSG00000213071 | ASTE1 | black |
| ENSG00000214402 | SH3YL1 | black |
| ENSG00000214944 | TIMP2 | black |
| ENSG00000225079 | FBXO42 | black |
| ENSG00000225302 | MFAP3 | black |
| ENSG00000225720 | METTL1 | black |
| ENSG00000227191 | CTNS | black |
| ENSG00000228172 | INPP4A | black |
| ENSG00000229373 | PARP3 | black |
| ENSG00000231769 | THAP3 | black |
| ENSG00000232320 | MED17 | black |
| ENSG00000232434 | AP2S1 | black |
| ENSG00000240771 | ADAM28 | black |
| ENSG00000246582 | CTNNA1 | black |
| ENSG00000255545 | DTNBP1 | black |
| ENSG00000259719 | TSPAN17 | black |
| ENSG00000260196 | CELF2 | black |
| ENSG00000260828 | ADAMTS6 | black |
| ENSG00000267248 | H6PD | black |
| ENSG00000269403 | SLC4A8 | black |
| ENSG00000269693 | PTGER3 | black |
| ENSG00000271503 | TNIP3 | black |
| ENSG00000272282 | HERPUD1 | black |
| ENSG00000272908 | RRP12 | black |
| ENSG00000273179 | FOXN3 | black |
| ENSG00000274588 | AKR7A2 | black |
| ENSG00000278709 | MRTO4 | black |
| ENSG00000000938 | USE1 | blue |
| ENSG00000001461 | MCF2L2 | blue |
| ENSG00000001630 | KCNQ1 | blue |
| ENSG00000002016 | TRAPPC3 | blue |
| ENSG00000002586 | THRAP3 | blue |
| ENSG00000002919 | PHPT1 | blue |
| ENSG00000003249 | SDCCAG8 | blue |
| ENSG00000003756 | PTPRN | blue |
| ENSG00000004142 | ATP9A | blue |
| ENSG00000004455 | SZRD1 | blue |
| ENSG00000004478 | KCNH2 | blue |
| ENSG00000004534 | MCOLN3 | blue |
| ENSG00000004779 | MRPL43 | blue |
| ENSG00000004809 | IL17RB | blue |
| ENSG00000004866 | TRAF3IP2 | blue |

| | | |
|---|---|---|
| ENSG00000005007 | MSH4 | blue |
| ENSG00000005022 | SEC61A1 | blue |
| ENSG00000005102 | DGKG | blue |
| ENSG00000005238 | TBXAS1 | blue |
| ENSG00000005243 | GATB | blue |
| ENSG00000005448 | STYK1 | blue |
| ENSG00000005483 | GNA15 | blue |
| ENSG00000005700 | SNRNP40 | blue |
| ENSG00000006015 | ACAA1 | blue |
| ENSG00000006125 | TNK2 | blue |
| ENSG00000006607 | DGAT2 | blue |
| ENSG00000006712 | CS | blue |
| ENSG00000006757 | RPL18 | blue |
| ENSG00000007080 | CA11 | blue |
| ENSG00000007237 | ISOC2 | blue |
| ENSG00000007255 | ZNF275 | blue |
| ENSG00000007312 | RNF4 | blue |
| ENSG00000007341 | LIMCH1 | blue |
| ENSG00000007392 | SPA17 | blue |
| ENSG00000007520 | ATP2C2 | blue |
| ENSG00000008018 | RFXANK | blue |
| ENSG00000008382 | TMEM161A | blue |
| ENSG00000008394 | LPAR2 | blue |
| ENSG00000008438 | CTSA | blue |
| ENSG00000008516 | SBNO2 | blue |
| ENSG00000008517 | AP3D1 | blue |
| ENSG00000008735 | NTHL1 | blue |
| ENSG00000008838 | UHRF1BP1 | blue |
| ENSG00000008988 | WDR18 | blue |
| ENSG00000009694 | NTN1 | blue |
| ENSG00000009790 | ANKRD44 | blue |
| ENSG00000010165 | ADAT1 | blue |
| ENSG00000010219 | PDIA5 | blue |
| ENSG00000010256 | NDUFB4 | blue |
| ENSG00000010610 | SPEN | blue |
| ENSG00000010704 | TBC1D1 | blue |
| ENSG00000010810 | CDK13 | blue |
| ENSG00000011009 | FOXJ2 | blue |
| ENSG00000011114 | TIE1 | blue |
| ENSG00000011523 | NFYC | blue |
| ENSG00000012124 | CD84 | blue |
| ENSG00000012779 | SPI1 | blue |
| ENSG00000013016 | ZNRD1 | blue |
| ENSG00000013288 | MTFR1 | blue |
| ENSG00000013364 | MYO9A | blue |
| ENSG00000014914 | PFKP | blue |
| ENSG00000014919 | NEO1 | blue |
| ENSG00000015133 | TNFRSF1A | blue |

| | | |
|---|---|---|
| ENSG00000015285 | STOML1 | blue |
| ENSG00000015475 | PKM | blue |
| ENSG00000015532 | RRP15 | blue |
| ENSG00000015568 | RHOA | blue |
| ENSG00000016391 | DHX8 | blue |
| ENSG00000018189 | IDH3G | blue |
| ENSG00000019169 | PDZD4 | blue |
| ENSG00000019582 | MEF2A | blue |
| ENSG00000021762 | OTUD5 | blue |
| ENSG00000022277 | TFE3 | blue |
| ENSG00000022976 | GPKOW | blue |
| ENSG00000023330 | GRIPAP1 | blue |
| ENSG00000023572 | ATP11A | blue |
| ENSG00000023839 | TTC7A | blue |
| ENSG00000023902 | VPS35 | blue |
| ENSG00000025770 | BCL3 | blue |
| ENSG00000025772 | HDHD5 | blue |
| ENSG00000026559 | TMEM260 | blue |
| ENSG00000027847 | DGCR2 | blue |
| ENSG00000027869 | RNF126 | blue |
| ENSG00000028137 | ZXDC | blue |
| ENSG00000029364 | ST6GALNAC1 | blue |
| ENSG00000029993 | NDST1 | blue |
| ENSG00000030110 | ASNS | blue |
| ENSG00000030582 | AP3M2 | blue |
| ENSG00000031081 | EIF2B3 | blue |
| ENSG00000031698 | RAD18 | blue |
| ENSG00000032389 | WDR1 | blue |
| ENSG00000033011 | MBD3 | blue |
| ENSG00000033050 | FAM50A | blue |
| ENSG00000033100 | FAM3A | blue |
| ENSG00000034053 | PRKACA | blue |
| ENSG00000034510 | EPN2 | blue |
| ENSG00000034533 | LIMS2 | blue |
| ENSG00000035115 | HSD17B10 | blue |
| ENSG00000035862 | MARK2 | blue |
| ENSG00000037637 | HMMR | blue |
| ENSG00000037749 | DERL2 | blue |
| ENSG00000037897 | SIDT1 | blue |
| ENSG00000040531 | AP1M1 | blue |
| ENSG00000040933 | XRCC1 | blue |
| ENSG00000041880 | PDE8A | blue |
| ENSG00000041988 | SDHA | blue |
| ENSG00000042429 | ADAM11 | blue |
| ENSG00000042753 | PPP2R3A | blue |
| ENSG00000042980 | FERMT2 | blue |
| ENSG00000044115 | TBX21 | blue |
| ENSG00000047579 | MRPS34 | blue |

| | | |
|---|---|---|
| ENSG00000048140 | TSG101 | blue |
| ENSG00000048740 | NCBP3 | blue |
| ENSG00000049192 | NUAK1 | blue |
| ENSG00000049239 | ENO1 | blue |
| ENSG00000050438 | MYDGF | blue |
| ENSG00000050628 | ARHGEF10L | blue |
| ENSG00000050730 | SRI | blue |
| ENSG00000051108 | TTC38 | blue |
| ENSG00000052749 | TIMM21 | blue |
| ENSG00000053254 | ADD2 | blue |
| ENSG00000053371 | VPS9D1 | blue |
| ENSG00000053372 | SLC25A3 | blue |
| ENSG00000053501 | FSCN1 | blue |
| ENSG00000053524 | ACTB | blue |
| ENSG00000053918 | EXOSC7 | blue |
| ENSG00000054116 | KLHL20 | blue |
| ENSG00000054118 | GPATCH1 | blue |
| ENSG00000054148 | ICAM3 | blue |
| ENSG00000054282 | NT5C2 | blue |
| ENSG00000054356 | XAB2 | blue |
| ENSG00000054793 | GTF3C1 | blue |
| ENSG00000055070 | IL4R | blue |
| ENSG00000055118 | PAK3 | blue |
| ENSG00000055732 | SNRPA | blue |
| ENSG00000055950 | EXOSC5 | blue |
| ENSG00000056736 | APBB1IP | blue |
| ENSG00000056972 | SIRT6 | blue |
| ENSG00000057468 | CAPZB | blue |
| ENSG00000058262 | SMC1B | blue |
| ENSG00000058866 | GNB1 | blue |
| ENSG00000059377 | VDAC3 | blue |
| ENSG00000059691 | CBFA2T2 | blue |
| ENSG00000060140 | FDFT1 | blue |
| ENSG00000060558 | PAFAH1B3 | blue |
| ENSG00000060688 | CARMIL1 | blue |
| ENSG00000060971 | KEAP1 | blue |
| ENSG00000061938 | SLC35C2 | blue |
| ENSG00000062282 | CRYBG3 | blue |
| ENSG00000062485 | MAGI3 | blue |
| ENSG00000063177 | IMPG2 | blue |
| ENSG00000063180 | SMARCD3 | blue |
| ENSG00000063241 | WDR70 | blue |
| ENSG00000063587 | COL19A1 | blue |
| ENSG00000063978 | NFE2L1 | blue |
| ENSG00000064042 | ULK2 | blue |
| ENSG00000064199 | ZNF671 | blue |
| ENSG00000064270 | ZNF416 | blue |
| ENSG00000064490 | RPS5 | blue |

| | | |
|---|---|---|
| ENSG00000064545 | SMAP2 | blue |
| ENSG00000064547 | PPIE | blue |
| ENSG00000064601 | GSTP1 | blue |
| ENSG00000064932 | APLP2 | blue |
| ENSG00000065000 | FAM234B | blue |
| ENSG00000065057 | EIF3I | blue |
| ENSG00000065060 | KIF3C | blue |
| ENSG00000065268 | HADHA | blue |
| ENSG00000065320 | BCORL1 | blue |
| ENSG00000065413 | FCN1 | blue |
| ENSG00000065457 | SLC25A24 | blue |
| ENSG00000065485 | PILRA | blue |
| ENSG00000065518 | AKR1B1 | blue |
| ENSG00000065526 | MGST2 | blue |
| ENSG00000065882 | B4GALT1 | blue |
| ENSG00000065883 | SEPHS1 | blue |
| ENSG00000065970 | MRPL28 | blue |
| ENSG00000066056 | LAT2 | blue |
| ENSG00000066136 | PPP1R15A | blue |
| ENSG00000066294 | FTL | blue |
| ENSG00000066336 | SRRT | blue |
| ENSG00000066379 | BAX | blue |
| ENSG00000066855 | PSMC5 | blue |
| ENSG00000066933 | OGFOD1 | blue |
| ENSG00000067057 | ADD1 | blue |
| ENSG00000067141 | SF3B2 | blue |
| ENSG00000067182 | GNAS | blue |
| ENSG00000067221 | AAMDC | blue |
| ENSG00000067225 | KHSRP | blue |
| ENSG00000067533 | EDEM2 | blue |
| ENSG00000067560 | TPX2 | blue |
| ENSG00000067596 | DOCK3 | blue |
| ENSG00000067829 | COQ9 | blue |
| ENSG00000068305 | TMEM40 | blue |
| ENSG00000068308 | FKBP1A | blue |
| ENSG00000068323 | CPXM1 | blue |
| ENSG00000068394 | DYNLL1 | blue |
| ENSG00000068400 | DZANK1 | blue |
| ENSG00000068650 | KDM2B | blue |
| ENSG00000068724 | RPLP0 | blue |
| ENSG00000069329 | PXN | blue |
| ENSG00000069399 | SIRT4 | blue |
| ENSG00000069998 | BRAP | blue |
| ENSG00000070269 | ERP29 | blue |
| ENSG00000070413 | FXYD5 | blue |
| ENSG00000070423 | GANAB | blue |
| ENSG00000070476 | GMIP | blue |
| ENSG00000070526 | GPATCH2L | blue |

| | | |
|---|---|---|
| ENSG00000070614 | SLC9A1 | blue |
| ENSG00000070669 | CCNK | blue |
| ENSG00000070718 | NDUFB2 | blue |
| ENSG00000070785 | NUDC | blue |
| ENSG00000070950 | SPG21 | blue |
| ENSG00000071127 | AARS | blue |
| ENSG00000071655 | NAT14 | blue |
| ENSG00000071859 | TXNL1 | blue |
| ENSG00000071889 | FH | blue |
| ENSG00000072062 | ALKBH5 | blue |
| ENSG00000072134 | SPAG7 | blue |
| ENSG00000072163 | ZC3HC1 | blue |
| ENSG00000072506 | TMEM101 | blue |
| ENSG00000072518 | PSME1 | blue |
| ENSG00000072571 | SLC7A8 | blue |
| ENSG00000072849 | TOX4 | blue |
| ENSG00000072858 | TINF2 | blue |
| ENSG00000072958 | MYL6 | blue |
| ENSG00000073050 | AGO1 | blue |
| ENSG00000073417 | CLSPN | blue |
| ENSG00000073578 | UNC13D | blue |
| ENSG00000073670 | CDC45 | blue |
| ENSG00000073711 | COMT | blue |
| ENSG00000073712 | ADA2 | blue |
| ENSG00000074071 | AAAS | blue |
| ENSG00000074319 | DHPS | blue |
| ENSG00000074356 | HOOK2 | blue |
| ENSG00000074800 | SH2D3C | blue |
| ENSG00000074842 | NANS | blue |
| ENSG00000074964 | PDE6C | blue |
| ENSG00000075142 | NUBP2 | blue |
| ENSG00000075336 | SMIM24 | blue |
| ENSG00000075340 | BRPF3 | blue |
| ENSG00000075399 | ACOT7 | blue |
| ENSG00000075415 | SYDE2 | blue |
| ENSG00000075618 | ABLIM1 | blue |
| ENSG00000075624 | H2AFY2 | blue |
| ENSG00000075914 | OCEL1 | blue |
| ENSG00000076321 | ARVCF | blue |
| ENSG00000076650 | IL2RB | blue |
| ENSG00000076662 | MLC1 | blue |
| ENSG00000076685 | GZMH | blue |
| ENSG00000076924 | GZMB | blue |
| ENSG00000077235 | NCALD | blue |
| ENSG00000077238 | LIM2 | blue |
| ENSG00000077264 | NKG7 | blue |
| ENSG00000077312 | VIPR2 | blue |
| ENSG00000077348 | RGS9 | blue |

| | | |
|---|---|---|
| ENSG00000077420 | B3GAT1 | blue |
| ENSG00000077463 | ENPP5 | blue |
| ENSG00000077549 | GNLY | blue |
| ENSG00000077935 | C1orf21 | blue |
| ENSG00000078369 | CD160 | blue |
| ENSG00000078668 | FASLG | blue |
| ENSG00000078699 | CD244 | blue |
| ENSG00000079459 | BFSP1 | blue |
| ENSG00000079462 | SYT11 | blue |
| ENSG00000079691 | LGR6 | blue |
| ENSG00000079999 | KLRD1 | blue |
| ENSG00000080189 | PRR5L | blue |
| ENSG00000080200 | CHST12 | blue |
| ENSG00000081026 | FGFBP2 | blue |
| ENSG00000081148 | RMDN3 | blue |
| ENSG00000082014 | PAK6 | blue |
| ENSG00000082068 | RNF165 | blue |
| ENSG00000082293 | ERBB2 | blue |
| ENSG00000082641 | FKBP10 | blue |
| ENSG00000083290 | TM4SF19 | blue |
| ENSG00000083814 | GZMA | blue |
| ENSG00000083817 | GFOD1 | blue |
| ENSG00000083845 | CEP78 | blue |
| ENSG00000084070 | NCAM1 | blue |
| ENSG00000084072 | FEZ1 | blue |
| ENSG00000084207 | KLRF1 | blue |
| ENSG00000084234 | PRSS23 | blue |
| ENSG00000084444 | TMEM45B | blue |
| ENSG00000084623 | ADAMTS1 | blue |
| ENSG00000084731 | SLFN13 | blue |
| ENSG00000084754 | PCDH1 | blue |
| ENSG00000085185 | PPP2R2B | blue |
| ENSG00000085265 | B3GNT7 | blue |
| ENSG00000085491 | GPR153 | blue |
| ENSG00000085514 | ADGRG5 | blue |
| ENSG00000085662 | SPON2 | blue |
| ENSG00000085871 | ZFYVE28 | blue |
| ENSG00000086062 | CLDND2 | blue |
| ENSG00000086475 | FCRL3 | blue |
| ENSG00000086504 | SLC1A7 | blue |
| ENSG00000086730 | LEXM | blue |
| ENSG00000087074 | HPGDS | blue |
| ENSG00000087086 | EOMES | blue |
| ENSG00000087087 | PYHIN1 | blue |
| ENSG00000087088 | SAMD3 | blue |
| ENSG00000087191 | BAALC | blue |
| ENSG00000087263 | PLEKHF1 | blue |
| ENSG00000087274 | TRPV3 | blue |

| | | |
|---|---|---|
| ENSG00000087365 | AC090152.1 | blue |
| ENSG00000087460 | PTGDR | blue |
| ENSG00000087884 | CLIC3 | blue |
| ENSG00000088247 | LDB2 | blue |
| ENSG00000088298 | LGALS9B | blue |
| ENSG00000088325 | PDGFD | blue |
| ENSG00000088538 | SIGLEC17P | blue |
| ENSG00000088682 | HOPX | blue |
| ENSG00000088726 | NMUR1 | blue |
| ENSG00000088832 | EPHX4 | blue |
| ENSG00000088882 | CTSW | blue |
| ENSG00000088986 | BNC2 | blue |
| ENSG00000089091 | PODN | blue |
| ENSG00000089094 | PLA2G16 | blue |
| ENSG00000089157 | BOK | blue |
| ENSG00000089159 | ACOT4 | blue |
| ENSG00000089163 | PRF1 | blue |
| ENSG00000089234 | S1PR5 | blue |
| ENSG00000089248 | FCRL6 | blue |
| ENSG00000089327 | MXRA7 | blue |
| ENSG00000089597 | EFNA5 | blue |
| ENSG00000089639 | TOGARAM2 | blue |
| ENSG00000089916 | AKR1C3 | blue |
| ENSG00000090020 | KIF19 | blue |
| ENSG00000090061 | DTHD1 | blue |
| ENSG00000090266 | SH2D1B | blue |
| ENSG00000090273 | CD247 | blue |
| ENSG00000090487 | ITPRIPL1 | blue |
| ENSG00000090861 | VIT | blue |
| ENSG00000090971 | ADGRG1 | blue |
| ENSG00000091164 | TRGJP2 | blue |
| ENSG00000091483 | TRAV9-2 | blue |
| ENSG00000091542 | TRDJ1 | blue |
| ENSG00000091640 | TRDC | blue |
| ENSG00000091732 | LPAL2 | blue |
| ENSG00000091947 | LCNL1 | blue |
| ENSG00000092010 | ARHGEF28 | blue |
| ENSG00000092068 | FTH1P22 | blue |
| ENSG00000092203 | AC023283.1 | blue |
| ENSG00000092330 | AL031846.1 | blue |
| ENSG00000092841 | TRGC2 | blue |
| ENSG00000092847 | AL020996.1 | blue |
| ENSG00000092853 | LINC00452 | blue |
| ENSG00000092929 | AL035701.1 | blue |
| ENSG00000093009 | AC009299.2 | blue |
| ENSG00000093010 | C9orf172 | blue |
| ENSG00000093072 | ARHGEF25 | blue |
| ENSG00000094914 | AC100861.1 | blue |

| | | |
|---|---|---|
| ENSG00000095059 | AP004608.1 | blue |
| ENSG00000095066 | LINC02284 | blue |
| ENSG00000095370 | AC124798.1 | blue |
| ENSG00000095380 | AC018552.3 | blue |
| ENSG00000095464 | AC025048.2 | blue |
| ENSG00000095906 | AC008750.8 | blue |
| ENSG00000095932 | AC010422.6 | blue |
| ENSG00000096070 | CCL5 | blue |
| ENSG00000097021 | LINC02084 | blue |
| ENSG00000097096 | AC006033.2 | blue |
| ENSG00000099204 | AC092535.4 | blue |
| ENSG00000099284 | DGKK | blue |
| ENSG00000099330 | NKILA | blue |
| ENSG00000099341 | PSMD8 | blue |
| ENSG00000099377 | HSD3B7 | blue |
| ENSG00000099385 | BCL7C | blue |
| ENSG00000099624 | ATP5D | blue |
| ENSG00000099795 | NDUFB7 | blue |
| ENSG00000099797 | TECR | blue |
| ENSG00000099800 | TIMM13 | blue |
| ENSG00000099817 | POLR2E | blue |
| ENSG00000099821 | POLRMT | blue |
| ENSG00000099901 | RANBP1 | blue |
| ENSG00000099940 | SNAP29 | blue |
| ENSG00000099995 | SF3A1 | blue |
| ENSG00000099998 | GGT5 | blue |
| ENSG00000100014 | SPECC1L | blue |
| ENSG00000100027 | YPEL1 | blue |
| ENSG00000100028 | SNRPD3 | blue |
| ENSG00000100029 | PES1 | blue |
| ENSG00000100036 | SLC35E4 | blue |
| ENSG00000100055 | CYTH4 | blue |
| ENSG00000100060 | MFNG | blue |
| ENSG00000100075 | SLC25A1 | blue |
| ENSG00000100079 | LGALS2 | blue |
| ENSG00000100084 | HIRA | blue |
| ENSG00000100097 | LGALS1 | blue |
| ENSG00000100116 | GCAT | blue |
| ENSG00000100124 | ANKRD54 | blue |
| ENSG00000100129 | EIF3L | blue |
| ENSG00000100138 | SNU13 | blue |
| ENSG00000100162 | CENPM | blue |
| ENSG00000100196 | KDELR3 | blue |
| ENSG00000100201 | DDX17 | blue |
| ENSG00000100207 | TCF20 | blue |
| ENSG00000100209 | HSCB | blue |
| ENSG00000100211 | CBY1 | blue |
| ENSG00000100216 | TOMM22 | blue |

| | | |
|---|---|---|
| ENSG00000100219 | XBP1 | blue |
| ENSG00000100227 | POLDIP3 | blue |
| ENSG00000100242 | SUN2 | blue |
| ENSG00000100243 | CYB5R3 | blue |
| ENSG00000100280 | AP1B1 | blue |
| ENSG00000100285 | NEFH | blue |
| ENSG00000100290 | BIK | blue |
| ENSG00000100292 | HMOX1 | blue |
| ENSG00000100296 | THOC5 | blue |
| ENSG00000100297 | MCM5 | blue |
| ENSG00000100298 | APOBEC3H | blue |
| ENSG00000100311 | PDGFB | blue |
| ENSG00000100316 | RPL3 | blue |
| ENSG00000100345 | MYH9 | blue |
| ENSG00000100347 | SAMM50 | blue |
| ENSG00000100348 | TXN2 | blue |
| ENSG00000100353 | EIF3D | blue |
| ENSG00000100354 | TNRC6B | blue |
| ENSG00000100364 | KIAA0930 | blue |
| ENSG00000100365 | NCF4 | blue |
| ENSG00000100373 | UPK3A | blue |
| ENSG00000100393 | EP300 | blue |
| ENSG00000100395 | L3MBTL2 | blue |
| ENSG00000100401 | RANGAP1 | blue |
| ENSG00000100412 | ACO2 | blue |
| ENSG00000100429 | HDAC10 | blue |
| ENSG00000100441 | KHNYN | blue |
| ENSG00000100461 | RBM23 | blue |
| ENSG00000100490 | CDKL1 | blue |
| ENSG00000100505 | TRIM9 | blue |
| ENSG00000100591 | AHSA1 | blue |
| ENSG00000100601 | ALKBH1 | blue |
| ENSG00000100612 | DHRS7 | blue |
| ENSG00000100697 | DICER1 | blue |
| ENSG00000100721 | TCL1A | blue |
| ENSG00000100722 | ZC3H14 | blue |
| ENSG00000100784 | RPS6KA5 | blue |
| ENSG00000100802 | C14orf93 | blue |
| ENSG00000100804 | PSMB5 | blue |
| ENSG00000100814 | CCNB1IP1 | blue |
| ENSG00000100823 | APEX1 | blue |
| ENSG00000100836 | PABPN1 | blue |
| ENSG00000100888 | CHD8 | blue |
| ENSG00000100889 | PCK2 | blue |
| ENSG00000100897 | DCAF11 | blue |
| ENSG00000100908 | EMC9 | blue |
| ENSG00000100968 | NFATC4 | blue |
| ENSG00000100983 | GSS | blue |

| | | |
|---|---|---|
| ENSG00000100985 | MMP9 | blue |
| ENSG00000100991 | TRPC4AP | blue |
| ENSG00000100997 | ABHD12 | blue |
| ENSG00000101017 | CD40 | blue |
| ENSG00000101052 | IFT52 | blue |
| ENSG00000101082 | SLA2 | blue |
| ENSG00000101138 | CSTF1 | blue |
| ENSG00000101146 | RAE1 | blue |
| ENSG00000101152 | DNAJC5 | blue |
| ENSG00000101160 | CTSZ | blue |
| ENSG00000101161 | PRPF6 | blue |
| ENSG00000101181 | MTG2 | blue |
| ENSG00000101182 | PSMA7 | blue |
| ENSG00000101187 | SLCO4A1 | blue |
| ENSG00000101220 | C20orf27 | blue |
| ENSG00000101224 | CDC25B | blue |
| ENSG00000101246 | ARFRP1 | blue |
| ENSG00000101290 | CDS2 | blue |
| ENSG00000101336 | HCK | blue |
| ENSG00000101337 | TM9SF4 | blue |
| ENSG00000101353 | MROH8 | blue |
| ENSG00000101365 | IDH3B | blue |
| ENSG00000101412 | E2F1 | blue |
| ENSG00000101421 | CHMP4B | blue |
| ENSG00000101439 | CST3 | blue |
| ENSG00000101444 | AHCY | blue |
| ENSG00000101457 | DNTTIP1 | blue |
| ENSG00000101460 | MAP1LC3A | blue |
| ENSG00000101464 | PIGU | blue |
| ENSG00000101608 | MYL12A | blue |
| ENSG00000101654 | RNMT | blue |
| ENSG00000101811 | CSTF2 | blue |
| ENSG00000101883 | RHOXF1 | blue |
| ENSG00000101898 | MCTS2P | blue |
| ENSG00000101945 | SUV39H1 | blue |
| ENSG00000101997 | CCDC22 | blue |
| ENSG00000102057 | KCND1 | blue |
| ENSG00000102100 | SLC35A2 | blue |
| ENSG00000102103 | PQBP1 | blue |
| ENSG00000102109 | PCSK1N | blue |
| ENSG00000102144 | PGK1 | blue |
| ENSG00000102178 | UBL4A | blue |
| ENSG00000102181 | CD99L2 | blue |
| ENSG00000102230 | PCYT1B | blue |
| ENSG00000102316 | MAGED2 | blue |
| ENSG00000102349 | KLF8 | blue |
| ENSG00000102393 | GLA | blue |
| ENSG00000102575 | ACP5 | blue |

| | | |
|---|---|---|
| ENSG00000102678 | FGF9 | blue |
| ENSG00000102710 | SUPT20H | blue |
| ENSG00000102780 | DGKH | blue |
| ENSG00000102786 | INTS6 | blue |
| ENSG00000102796 | DHRS12 | blue |
| ENSG00000102858 | MGRN1 | blue |
| ENSG00000102870 | ZNF629 | blue |
| ENSG00000102898 | NUTF2 | blue |
| ENSG00000102901 | CENPT | blue |
| ENSG00000102931 | ARL2BP | blue |
| ENSG00000102977 | ACD | blue |
| ENSG00000103005 | USB1 | blue |
| ENSG00000103024 | NME3 | blue |
| ENSG00000103035 | PSMD7 | blue |
| ENSG00000103044 | HAS3 | blue |
| ENSG00000103145 | HCFC1R1 | blue |
| ENSG00000103152 | MPG | blue |
| ENSG00000103174 | NAGPA | blue |
| ENSG00000103187 | COTL1 | blue |
| ENSG00000103196 | CRISPLD2 | blue |
| ENSG00000103226 | NOMO3 | blue |
| ENSG00000103254 | FAM173A | blue |
| ENSG00000103266 | STUB1 | blue |
| ENSG00000103274 | NUBP1 | blue |
| ENSG00000103363 | ELOB | blue |
| ENSG00000103381 | CPPED1 | blue |
| ENSG00000103404 | USP31 | blue |
| ENSG00000103494 | RPGRIP1L | blue |
| ENSG00000103512 | NOMO1 | blue |
| ENSG00000103544 | C16orf62 | blue |
| ENSG00000103647 | CORO2B | blue |
| ENSG00000103653 | CSK | blue |
| ENSG00000103876 | FAH | blue |
| ENSG00000103942 | HOMER2 | blue |
| ENSG00000103966 | EHD4 | blue |
| ENSG00000103994 | ZNF106 | blue |
| ENSG00000104081 | BMF | blue |
| ENSG00000104129 | DNAJC17 | blue |
| ENSG00000104133 | SPG11 | blue |
| ENSG00000104177 | MYEF2 | blue |
| ENSG00000104221 | BRF2 | blue |
| ENSG00000104299 | INTS9 | blue |
| ENSG00000104324 | CPQ | blue |
| ENSG00000104325 | DECR1 | blue |
| ENSG00000104327 | CALB1 | blue |
| ENSG00000104361 | NIPAL2 | blue |
| ENSG00000104419 | NDRG1 | blue |
| ENSG00000104472 | CHRAC1 | blue |

| | | |
|---|---|---|
| ENSG00000104611 | SH2D4A | blue |
| ENSG00000104679 | R3HCC1 | blue |
| ENSG00000104805 | NUCB1 | blue |
| ENSG00000104808 | DHDH | blue |
| ENSG00000104814 | MAP4K1 | blue |
| ENSG00000104856 | RELB | blue |
| ENSG00000104859 | CLASRP | blue |
| ENSG00000104863 | LIN7B | blue |
| ENSG00000104872 | PIH1D1 | blue |
| ENSG00000104886 | PLEKHJ1 | blue |
| ENSG00000104889 | RNASEH2A | blue |
| ENSG00000104899 | AMH | blue |
| ENSG00000104904 | OAZ1 | blue |
| ENSG00000104907 | TRMT1 | blue |
| ENSG00000104921 | FCER2 | blue |
| ENSG00000104964 | AES | blue |
| ENSG00000104976 | SNAPC2 | blue |
| ENSG00000104979 | C19orf53 | blue |
| ENSG00000104983 | CCDC61 | blue |
| ENSG00000105053 | VRK3 | blue |
| ENSG00000105127 | AKAP8 | blue |
| ENSG00000105171 | POP4 | blue |
| ENSG00000105193 | RPS16 | blue |
| ENSG00000105202 | FBL | blue |
| ENSG00000105220 | GPI | blue |
| ENSG00000105223 | PLD3 | blue |
| ENSG00000105248 | CCDC94 | blue |
| ENSG00000105254 | TBCB | blue |
| ENSG00000105258 | POLR2I | blue |
| ENSG00000105281 | SLC1A5 | blue |
| ENSG00000105287 | PRKD2 | blue |
| ENSG00000105323 | HNRNPUL1 | blue |
| ENSG00000105355 | PLIN3 | blue |
| ENSG00000105364 | MRPL4 | blue |
| ENSG00000105369 | CD79A | blue |
| ENSG00000105372 | RPS19 | blue |
| ENSG00000105373 | NOP53 | blue |
| ENSG00000105379 | ETFB | blue |
| ENSG00000105393 | BABAM1 | blue |
| ENSG00000105401 | CDC37 | blue |
| ENSG00000105404 | RABAC1 | blue |
| ENSG00000105409 | ATP1A3 | blue |
| ENSG00000105426 | PTPRS | blue |
| ENSG00000105438 | KDELR1 | blue |
| ENSG00000105472 | CLEC11A | blue |
| ENSG00000105483 | CARD8 | blue |
| ENSG00000105501 | SIGLEC5 | blue |
| ENSG00000105516 | DBP | blue |

| | | |
|---|---|---|
| ENSG00000105518 | TMEM205 | blue |
| ENSG00000105538 | RASIP1 | blue |
| ENSG00000105552 | BCAT2 | blue |
| ENSG00000105556 | MIER2 | blue |
| ENSG00000105559 | PLEKHA4 | blue |
| ENSG00000105568 | PPP2R1A | blue |
| ENSG00000105583 | WDR83OS | blue |
| ENSG00000105607 | GCDH | blue |
| ENSG00000105612 | DNASE2 | blue |
| ENSG00000105639 | JAK3 | blue |
| ENSG00000105640 | RPL18A | blue |
| ENSG00000105643 | ARRDC2 | blue |
| ENSG00000105655 | ISYNA1 | blue |
| ENSG00000105669 | COPE | blue |
| ENSG00000105671 | DDX49 | blue |
| ENSG00000105672 | ETV2 | blue |
| ENSG00000105676 | ARMC6 | blue |
| ENSG00000105677 | TMEM147 | blue |
| ENSG00000105698 | USF2 | blue |
| ENSG00000105737 | GRIK5 | blue |
| ENSG00000105755 | ETHE1 | blue |
| ENSG00000105771 | SMG9 | blue |
| ENSG00000105778 | AVL9 | blue |
| ENSG00000105851 | PIK3CG | blue |
| ENSG00000105926 | MPP6 | blue |
| ENSG00000105953 | OGDH | blue |
| ENSG00000106003 | LFNG | blue |
| ENSG00000106028 | SSBP1 | blue |
| ENSG00000106066 | CPVL | blue |
| ENSG00000106080 | FKBP14 | blue |
| ENSG00000106105 | GARS | blue |
| ENSG00000106153 | CHCHD2 | blue |
| ENSG00000106211 | HSPB1 | blue |
| ENSG00000106245 | BUD31 | blue |
| ENSG00000106263 | EIF3B | blue |
| ENSG00000106266 | SNX8 | blue |
| ENSG00000106268 | NUDT1 | blue |
| ENSG00000106305 | AIMP2 | blue |
| ENSG00000106348 | IMPDH1 | blue |
| ENSG00000106367 | AP1S1 | blue |
| ENSG00000106397 | PLOD3 | blue |
| ENSG00000106400 | ZNHIT1 | blue |
| ENSG00000106477 | CEP41 | blue |
| ENSG00000106484 | MEST | blue |
| ENSG00000106554 | CHCHD3 | blue |
| ENSG00000106609 | TMEM248 | blue |
| ENSG00000106624 | AEBP1 | blue |
| ENSG00000106628 | POLD2 | blue |

| | | |
|---|---|---|
| ENSG00000106635 | BCL7B | blue |
| ENSG00000106638 | TBL2 | blue |
| ENSG00000106682 | EIF4H | blue |
| ENSG00000106683 | LIMK1 | blue |
| ENSG00000106714 | CNTNAP3 | blue |
| ENSG00000106803 | SEC61B | blue |
| ENSG00000107018 | RLN1 | blue |
| ENSG00000107130 | NCS1 | blue |
| ENSG00000107175 | CREB3 | blue |
| ENSG00000107223 | EDF1 | blue |
| ENSG00000107249 | GLIS3 | blue |
| ENSG00000107438 | PDLIM1 | blue |
| ENSG00000107551 | RASSF4 | blue |
| ENSG00000107554 | DNMBP | blue |
| ENSG00000107738 | VSIR | blue |
| ENSG00000107745 | MICU1 | blue |
| ENSG00000107829 | FBXW4 | blue |
| ENSG00000107833 | NPM3 | blue |
| ENSG00000107862 | GBF1 | blue |
| ENSG00000107864 | CPEB3 | blue |
| ENSG00000107872 | FBXL15 | blue |
| ENSG00000107874 | CUEDC2 | blue |
| ENSG00000107902 | LHPP | blue |
| ENSG00000107959 | PITRM1 | blue |
| ENSG00000108039 | XPNPEP1 | blue |
| ENSG00000108106 | UBE2S | blue |
| ENSG00000108107 | RPL28 | blue |
| ENSG00000108179 | PPIF | blue |
| ENSG00000108219 | TSPAN14 | blue |
| ENSG00000108298 | RPL19 | blue |
| ENSG00000108306 | FBXL20 | blue |
| ENSG00000108344 | PSMD3 | blue |
| ENSG00000108349 | CASC3 | blue |
| ENSG00000108389 | MTMR4 | blue |
| ENSG00000108423 | TUBD1 | blue |
| ENSG00000108433 | GOSR2 | blue |
| ENSG00000108518 | PFN1 | blue |
| ENSG00000108557 | RAI1 | blue |
| ENSG00000108561 | C1QBP | blue |
| ENSG00000108592 | FTSJ3 | blue |
| ENSG00000108604 | SMARCD2 | blue |
| ENSG00000108639 | SYNGR2 | blue |
| ENSG00000108671 | PSMD11 | blue |
| ENSG00000108774 | RAB5C | blue |
| ENSG00000108826 | MRPL27 | blue |
| ENSG00000108828 | VAT1 | blue |
| ENSG00000108829 | LRRC59 | blue |
| ENSG00000108848 | LUC7L3 | blue |

| | | |
|---|---|---|
| ENSG00000108883 | EFTUD2 | blue |
| ENSG00000108958 | AC130689.1 | blue |
| ENSG00000109079 | TNFAIP1 | blue |
| ENSG00000109089 | CDR2L | blue |
| ENSG00000109103 | UNC119 | blue |
| ENSG00000109111 | SUPT6H | blue |
| ENSG00000109113 | RAB34 | blue |
| ENSG00000109390 | NDUFC1 | blue |
| ENSG00000109458 | GAB1 | blue |
| ENSG00000109519 | GRPEL1 | blue |
| ENSG00000109534 | GAR1 | blue |
| ENSG00000109684 | CLNK | blue |
| ENSG00000109881 | CCDC34 | blue |
| ENSG00000109917 | ZPR1 | blue |
| ENSG00000109919 | MTCH2 | blue |
| ENSG00000109943 | CRTAM | blue |
| ENSG00000110011 | DNAJC4 | blue |
| ENSG00000110031 | LPXN | blue |
| ENSG00000110042 | DTX4 | blue |
| ENSG00000110063 | DCPS | blue |
| ENSG00000110080 | ST3GAL4 | blue |
| ENSG00000110107 | PRPF19 | blue |
| ENSG00000110344 | UBE4A | blue |
| ENSG00000110442 | COMMD9 | blue |
| ENSG00000110446 | SLC15A3 | blue |
| ENSG00000110536 | PTPMT1 | blue |
| ENSG00000110583 | NAA40 | blue |
| ENSG00000110651 | CD81 | blue |
| ENSG00000110665 | C11orf21 | blue |
| ENSG00000110700 | RPS13 | blue |
| ENSG00000110711 | AIP | blue |
| ENSG00000110717 | NDUFS8 | blue |
| ENSG00000110719 | TCIRG1 | blue |
| ENSG00000110876 | SELPLG | blue |
| ENSG00000110888 | CAPRIN2 | blue |
| ENSG00000110917 | MLEC | blue |
| ENSG00000110931 | CAMKK2 | blue |
| ENSG00000110934 | BIN2 | blue |
| ENSG00000110955 | ATP5B | blue |
| ENSG00000111052 | LIN7A | blue |
| ENSG00000111057 | KRT18 | blue |
| ENSG00000111144 | LTA4H | blue |
| ENSG00000111206 | FOXM1 | blue |
| ENSG00000111229 | ARPC3 | blue |
| ENSG00000111249 | CUX2 | blue |
| ENSG00000111321 | LTBR | blue |
| ENSG00000111364 | DDX55 | blue |
| ENSG00000111481 | COPZ1 | blue |

| | | |
|---|---|---|
| ENSG00000111537 | IFNG | blue |
| ENSG00000111540 | RAB5B | blue |
| ENSG00000111639 | MRPL51 | blue |
| ENSG00000111640 | GAPDH | blue |
| ENSG00000111653 | ING4 | blue |
| ENSG00000111667 | USP5 | blue |
| ENSG00000111669 | TPI1 | blue |
| ENSG00000111678 | C12orf57 | blue |
| ENSG00000111679 | PTPN6 | blue |
| ENSG00000111737 | RAB35 | blue |
| ENSG00000111775 | COX6A1 | blue |
| ENSG00000111785 | RIC8B | blue |
| ENSG00000111788 | AC009533.1 | blue |
| ENSG00000111850 | SMIM8 | blue |
| ENSG00000111885 | MAN1A1 | blue |
| ENSG00000111886 | GABRR2 | blue |
| ENSG00000112130 | RNF8 | blue |
| ENSG00000112195 | TREML2 | blue |
| ENSG00000112293 | GPLD1 | blue |
| ENSG00000112297 | CRYBG1 | blue |
| ENSG00000112299 | VNN1 | blue |
| ENSG00000112306 | RPS12 | blue |
| ENSG00000112514 | CUTA | blue |
| ENSG00000112561 | TFEB | blue |
| ENSG00000112576 | CCND3 | blue |
| ENSG00000112640 | PPP2R5D | blue |
| ENSG00000112651 | MRPL2 | blue |
| ENSG00000112667 | DNPH1 | blue |
| ENSG00000112679 | DUSP22 | blue |
| ENSG00000112695 | COX7A2 | blue |
| ENSG00000112699 | GMDS | blue |
| ENSG00000112715 | VEGFA | blue |
| ENSG00000112799 | LY86 | blue |
| ENSG00000112855 | HARS2 | blue |
| ENSG00000113068 | PFDN1 | blue |
| ENSG00000113141 | IK | blue |
| ENSG00000113312 | TTC1 | blue |
| ENSG00000113552 | GNPDA1 | blue |
| ENSG00000113645 | WWC1 | blue |
| ENSG00000113721 | PDGFRB | blue |
| ENSG00000113732 | ATP6V0E1 | blue |
| ENSG00000113749 | HRH2 | blue |
| ENSG00000113966 | ARL6 | blue |
| ENSG00000114021 | NIT2 | blue |
| ENSG00000114023 | FAM162A | blue |
| ENSG00000114030 | KPNA1 | blue |
| ENSG00000114270 | COL7A1 | blue |
| ENSG00000114353 | GNAI2 | blue |

| | | |
|---|---|---|
| ENSG00000114391 | RPL24 | blue |
| ENSG00000114395 | CYB561D2 | blue |
| ENSG00000114529 | C3orf52 | blue |
| ENSG00000114767 | RRP9 | blue |
| ENSG00000114770 | ABCC5 | blue |
| ENSG00000114779 | ABHD14B | blue |
| ENSG00000114854 | TNNC1 | blue |
| ENSG00000114857 | NKTR | blue |
| ENSG00000114902 | SPCS1 | blue |
| ENSG00000114942 | EEF1B2 | blue |
| ENSG00000114956 | DGUOK | blue |
| ENSG00000114993 | RTKN | blue |
| ENSG00000115084 | SLC35F5 | blue |
| ENSG00000115145 | STAM2 | blue |
| ENSG00000115183 | TANC1 | blue |
| ENSG00000115234 | SNX17 | blue |
| ENSG00000115239 | ASB3 | blue |
| ENSG00000115241 | PPM1G | blue |
| ENSG00000115257 | PCSK4 | blue |
| ENSG00000115268 | RPS15 | blue |
| ENSG00000115286 | NDUFS7 | blue |
| ENSG00000115289 | PCGF1 | blue |
| ENSG00000115318 | LOXL3 | blue |
| ENSG00000115350 | POLE4 | blue |
| ENSG00000115423 | DNAH6 | blue |
| ENSG00000115464 | USP34 | blue |
| ENSG00000115507 | OTX1 | blue |
| ENSG00000115526 | CHST10 | blue |
| ENSG00000115539 | PDCL3 | blue |
| ENSG00000115590 | IL1R2 | blue |
| ENSG00000115641 | FHL2 | blue |
| ENSG00000115649 | CNPPD1 | blue |
| ENSG00000115685 | PPP1R7 | blue |
| ENSG00000115761 | NOL10 | blue |
| ENSG00000115902 | SLC1A4 | blue |
| ENSG00000115998 | C2orf42 | blue |
| ENSG00000116017 | ARID3A | blue |
| ENSG00000116117 | PARD3B | blue |
| ENSG00000116120 | FARSB | blue |
| ENSG00000116147 | TNR | blue |
| ENSG00000116157 | GPX7 | blue |
| ENSG00000116194 | ANGPTL1 | blue |
| ENSG00000116221 | MRPL37 | blue |
| ENSG00000116254 | CHD5 | blue |
| ENSG00000116260 | QSOX1 | blue |
| ENSG00000116288 | PARK7 | blue |
| ENSG00000116329 | OPRD1 | blue |
| ENSG00000116350 | SRSF4 | blue |

| | | |
|---|---|---|
| ENSG00000116353 | MECR | blue |
| ENSG00000116478 | HDAC1 | blue |
| ENSG00000116580 | GON4L | blue |
| ENSG00000116586 | LAMTOR2 | blue |
| ENSG00000116649 | SRM | blue |
| ENSG00000116670 | MAD2L2 | blue |
| ENSG00000116698 | SMG7 | blue |
| ENSG00000116731 | PRDM2 | blue |
| ENSG00000116761 | CTH | blue |
| ENSG00000116819 | TFAP2E | blue |
| ENSG00000116824 | CD2 | blue |
| ENSG00000116857 | TMEM9 | blue |
| ENSG00000116863 | ADPRHL2 | blue |
| ENSG00000116871 | MAP7D1 | blue |
| ENSG00000116883 | AL591845.1 | blue |
| ENSG00000116983 | HPCAL4 | blue |
| ENSG00000117118 | SDHB | blue |
| ENSG00000117298 | ECE1 | blue |
| ENSG00000117305 | HMGCL | blue |
| ENSG00000117362 | APH1A | blue |
| ENSG00000117395 | EBNA1BP2 | blue |
| ENSG00000117408 | IPO13 | blue |
| ENSG00000117448 | AKR1A1 | blue |
| ENSG00000117450 | PRDX1 | blue |
| ENSG00000117592 | PRDX6 | blue |
| ENSG00000117595 | IRF6 | blue |
| ENSG00000117691 | NENF | blue |
| ENSG00000117834 | SLC5A9 | blue |
| ENSG00000117868 | ESYT2 | blue |
| ENSG00000118096 | IFT46 | blue |
| ENSG00000118257 | NRP2 | blue |
| ENSG00000118263 | KLF7 | blue |
| ENSG00000118307 | CASC1 | blue |
| ENSG00000118363 | SPCS2 | blue |
| ENSG00000118420 | UBE3D | blue |
| ENSG00000118520 | ARG1 | blue |
| ENSG00000118640 | VAMP8 | blue |
| ENSG00000118690 | ARMC2 | blue |
| ENSG00000118707 | TGIF2 | blue |
| ENSG00000118785 | SPP1 | blue |
| ENSG00000118804 | STBD1 | blue |
| ENSG00000118997 | DNAH7 | blue |
| ENSG00000119121 | TRPM6 | blue |
| ENSG00000119231 | SENP5 | blue |
| ENSG00000119280 | C1orf198 | blue |
| ENSG00000119333 | WDR34 | blue |
| ENSG00000119383 | PTPA | blue |
| ENSG00000119403 | PHF19 | blue |

| | | |
|---|---|---|
| ENSG00000119408 | NEK6 | blue |
| ENSG00000119411 | BSPRY | blue |
| ENSG00000119421 | NDUFA8 | blue |
| ENSG00000119431 | HDHD3 | blue |
| ENSG00000119487 | MAPKAP1 | blue |
| ENSG00000119509 | INVS | blue |
| ENSG00000119522 | DENND1A | blue |
| ENSG00000119535 | CSF3R | blue |
| ENSG00000119574 | ZBTB45 | blue |
| ENSG00000119608 | PROX2 | blue |
| ENSG00000119632 | IFI27L2 | blue |
| ENSG00000119655 | NPC2 | blue |
| ENSG00000119681 | LTBP2 | blue |
| ENSG00000119688 | ABCD4 | blue |
| ENSG00000119699 | TGFB3 | blue |
| ENSG00000119705 | SLIRP | blue |
| ENSG00000119711 | ALDH6A1 | blue |
| ENSG00000119718 | EIF2B2 | blue |
| ENSG00000119986 | AVPI1 | blue |
| ENSG00000120029 | C10orf76 | blue |
| ENSG00000120156 | TEK | blue |
| ENSG00000120253 | NUP43 | blue |
| ENSG00000120306 | CYSTM1 | blue |
| ENSG00000120334 | CENPL | blue |
| ENSG00000120440 | TTLL2 | blue |
| ENSG00000120458 | MSANTD2 | blue |
| ENSG00000120509 | PDZD11 | blue |
| ENSG00000120659 | TNFSF11 | blue |
| ENSG00000120662 | MTRF1 | blue |
| ENSG00000120708 | TGFBI | blue |
| ENSG00000120725 | SIL1 | blue |
| ENSG00000120733 | KDM3B | blue |
| ENSG00000120742 | SERP1 | blue |
| ENSG00000120832 | MTERF2 | blue |
| ENSG00000120833 | SOCS2 | blue |
| ENSG00000120868 | APAF1 | blue |
| ENSG00000120896 | SORBS3 | blue |
| ENSG00000120899 | PTK2B | blue |
| ENSG00000121064 | SCPEP1 | blue |
| ENSG00000121073 | SLC35B1 | blue |
| ENSG00000121089 | NACA3P | blue |
| ENSG00000121316 | PLBD1 | blue |
| ENSG00000121406 | ZNF549 | blue |
| ENSG00000121417 | ZNF211 | blue |
| ENSG00000121570 | DPPA4 | blue |
| ENSG00000121594 | CD80 | blue |
| ENSG00000121680 | PEX16 | blue |
| ENSG00000121690 | DEPDC7 | blue |

| | | |
|---|---|---|
| ENSG00000121775 | TMEM39B | blue |
| ENSG00000121940 | CLCC1 | blue |
| ENSG00000121957 | GPSM2 | blue |
| ENSG00000122008 | POLK | blue |
| ENSG00000122012 | SV2C | blue |
| ENSG00000122025 | FLT3 | blue |
| ENSG00000122034 | GTF3A | blue |
| ENSG00000122085 | MTERF4 | blue |
| ENSG00000122121 | XPNPEP2 | blue |
| ENSG00000122140 | MRPS2 | blue |
| ENSG00000122188 | LAX1 | blue |
| ENSG00000122218 | COPA | blue |
| ENSG00000122490 | PQLC1 | blue |
| ENSG00000122512 | PMS2 | blue |
| ENSG00000122642 | FKBP9 | blue |
| ENSG00000122687 | MRM2 | blue |
| ENSG00000122694 | GLIPR2 | blue |
| ENSG00000122705 | CLTA | blue |
| ENSG00000122882 | ECD | blue |
| ENSG00000122912 | SLC25A16 | blue |
| ENSG00000122970 | IFT81 | blue |
| ENSG00000122971 | ACADS | blue |
| ENSG00000123066 | MED13L | blue |
| ENSG00000123096 | SSPN | blue |
| ENSG00000123104 | ITPR2 | blue |
| ENSG00000123119 | NECAB1 | blue |
| ENSG00000123131 | PRDX4 | blue |
| ENSG00000123143 | PKN1 | blue |
| ENSG00000123144 | TRIR | blue |
| ENSG00000123146 | ADGRE5 | blue |
| ENSG00000123159 | GIPC1 | blue |
| ENSG00000123297 | TSFM | blue |
| ENSG00000123329 | ARHGAP9 | blue |
| ENSG00000123338 | NCKAP1L | blue |
| ENSG00000123342 | MMP19 | blue |
| ENSG00000123352 | SPATS2 | blue |
| ENSG00000123384 | LRP1 | blue |
| ENSG00000123395 | ATG101 | blue |
| ENSG00000123405 | NFE2 | blue |
| ENSG00000123427 | EEF1AKMT3 | blue |
| ENSG00000123643 | SLC36A1 | blue |
| ENSG00000123843 | C4BPB | blue |
| ENSG00000123908 | AGO2 | blue |
| ENSG00000123933 | MXD4 | blue |
| ENSG00000123965 | PMS2P5 | blue |
| ENSG00000123992 | DNPEP | blue |
| ENSG00000124103 | FAM209A | blue |
| ENSG00000124107 | SLPI | blue |

| | | |
|---|---|---|
| ENSG00000124120 | TTPAL | blue |
| ENSG00000124145 | SDC4 | blue |
| ENSG00000124151 | NCOA3 | blue |
| ENSG00000124177 | CHD6 | blue |
| ENSG00000124203 | ZNF831 | blue |
| ENSG00000124215 | CDH26 | blue |
| ENSG00000124216 | SNAI1 | blue |
| ENSG00000124225 | PMEPA1 | blue |
| ENSG00000124233 | SEMG1 | blue |
| ENSG00000124299 | PEPD | blue |
| ENSG00000124374 | PAIP2B | blue |
| ENSG00000124444 | ZNF576 | blue |
| ENSG00000124496 | TRERF1 | blue |
| ENSG00000124549 | BTN2A3P | blue |
| ENSG00000124562 | SNRPC | blue |
| ENSG00000124570 | SERPINB6 | blue |
| ENSG00000124614 | RPS10 | blue |
| ENSG00000124657 | OR2B6 | blue |
| ENSG00000124701 | APOBEC2 | blue |
| ENSG00000124702 | KLHDC3 | blue |
| ENSG00000124731 | TREM1 | blue |
| ENSG00000124733 | MEA1 | blue |
| ENSG00000124743 | KLHL31 | blue |
| ENSG00000124762 | CDKN1A | blue |
| ENSG00000124782 | RREB1 | blue |
| ENSG00000124788 | ATXN1 | blue |
| ENSG00000124831 | LRRFIP1 | blue |
| ENSG00000124875 | CXCL6 | blue |
| ENSG00000125124 | BBS2 | blue |
| ENSG00000125166 | GOT2 | blue |
| ENSG00000125352 | RNF113A | blue |
| ENSG00000125386 | FAM193A | blue |
| ENSG00000125445 | MRPS7 | blue |
| ENSG00000125457 | MIF4GD | blue |
| ENSG00000125458 | NT5C | blue |
| ENSG00000125520 | SLC2A4RG | blue |
| ENSG00000125534 | PPDPF | blue |
| ENSG00000125651 | GTF2F1 | blue |
| ENSG00000125652 | ALKBH7 | blue |
| ENSG00000125656 | CLPP | blue |
| ENSG00000125743 | SNRPD2 | blue |
| ENSG00000125746 | EML2 | blue |
| ENSG00000125753 | VASP | blue |
| ENSG00000125812 | GZF1 | blue |
| ENSG00000125818 | PSMF1 | blue |
| ENSG00000125821 | DTD1 | blue |
| ENSG00000125835 | SNRPB | blue |
| ENSG00000125841 | NRSN2 | blue |

| | | |
|---|---|---|
| ENSG00000125844 | RRBP1 | blue |
| ENSG00000125877 | ITPA | blue |
| ENSG00000125885 | MCM8 | blue |
| ENSG00000125901 | MRPS26 | blue |
| ENSG00000125971 | DYNLRB1 | blue |
| ENSG00000125991 | ERGIC3 | blue |
| ENSG00000125995 | ROMO1 | blue |
| ENSG00000126003 | PLAGL2 | blue |
| ENSG00000126005 | MMP24-AS1 | blue |
| ENSG00000126062 | TMEM115 | blue |
| ENSG00000126070 | AGO3 | blue |
| ENSG00000126088 | UROD | blue |
| ENSG00000126106 | TMEM53 | blue |
| ENSG00000126247 | CAPNS1 | blue |
| ENSG00000126249 | PDCD2L | blue |
| ENSG00000126254 | RBM42 | blue |
| ENSG00000126264 | HCST | blue |
| ENSG00000126267 | COX6B1 | blue |
| ENSG00000126391 | FRMD8 | blue |
| ENSG00000126432 | PRDX5 | blue |
| ENSG00000126453 | BCL2L12 | blue |
| ENSG00000126456 | IRF3 | blue |
| ENSG00000126457 | PRMT1 | blue |
| ENSG00000126458 | RRAS | blue |
| ENSG00000126561 | STAT5A | blue |
| ENSG00000126756 | UXT | blue |
| ENSG00000126768 | TIMM17B | blue |
| ENSG00000126790 | L3HYPDH | blue |
| ENSG00000126970 | ZC4H2 | blue |
| ENSG00000127080 | IPPK | blue |
| ENSG00000127084 | FGD3 | blue |
| ENSG00000127191 | TRAF2 | blue |
| ENSG00000127220 | ABHD8 | blue |
| ENSG00000127311 | HELB | blue |
| ENSG00000127377 | CRYGN | blue |
| ENSG00000127528 | KLF2 | blue |
| ENSG00000127530 | OR7C1 | blue |
| ENSG00000127554 | GFER | blue |
| ENSG00000127561 | SYNGR3 | blue |
| ENSG00000127580 | WDR24 | blue |
| ENSG00000127589 | TUBBP1 | blue |
| ENSG00000127666 | TICAM1 | blue |
| ENSG00000127837 | AAMP | blue |
| ENSG00000127838 | PNKD | blue |
| ENSG00000127884 | ECHS1 | blue |
| ENSG00000127946 | HIP1 | blue |
| ENSG00000127955 | GNAI1 | blue |
| ENSG00000127990 | SGCE | blue |

| | | |
|---|---|---|
| ENSG00000128050 | PAICS | blue |
| ENSG00000128185 | DGCR6L | blue |
| ENSG00000128218 | VPREB3 | blue |
| ENSG00000128228 | SDF2L1 | blue |
| ENSG00000128245 | YWHAH | blue |
| ENSG00000128272 | ATF4 | blue |
| ENSG00000128463 | EMC4 | blue |
| ENSG00000128487 | SPECC1 | blue |
| ENSG00000128524 | ATP6V1F | blue |
| ENSG00000128563 | PRKRIP1 | blue |
| ENSG00000128567 | PODXL | blue |
| ENSG00000128585 | MKLN1 | blue |
| ENSG00000128596 | CCDC136 | blue |
| ENSG00000128602 | SMO | blue |
| ENSG00000128606 | LRRC17 | blue |
| ENSG00000128692 | EIF2S2P4 | blue |
| ENSG00000128694 | OSGEPL1 | blue |
| ENSG00000128739 | SNRPN | blue |
| ENSG00000128805 | ARHGAP22 | blue |
| ENSG00000128833 | MYO5C | blue |
| ENSG00000128872 | TMOD2 | blue |
| ENSG00000128881 | TTBK2 | blue |
| ENSG00000128923 | MINDY2 | blue |
| ENSG00000129007 | CALML4 | blue |
| ENSG00000129103 | SUMF2 | blue |
| ENSG00000129158 | SERGEF | blue |
| ENSG00000129167 | TPH1 | blue |
| ENSG00000129187 | DCTD | blue |
| ENSG00000129197 | RPAIN | blue |
| ENSG00000129250 | KIF1C | blue |
| ENSG00000129255 | MPDU1 | blue |
| ENSG00000129353 | SLC44A2 | blue |
| ENSG00000129354 | AP1M2 | blue |
| ENSG00000129355 | CDKN2D | blue |
| ENSG00000129467 | ADCY4 | blue |
| ENSG00000129474 | AJUBA | blue |
| ENSG00000129493 | HEATR5A | blue |
| ENSG00000129521 | EGLN3 | blue |
| ENSG00000129535 | NRL | blue |
| ENSG00000129538 | RNASE1 | blue |
| ENSG00000129559 | NEDD8 | blue |
| ENSG00000129562 | DAD1 | blue |
| ENSG00000129566 | TEP1 | blue |
| ENSG00000129696 | TTI2 | blue |
| ENSG00000129946 | SHC2 | blue |
| ENSG00000129993 | CBFA2T3 | blue |
| ENSG00000130005 | GAMT | blue |
| ENSG00000130035 | GALNT8 | blue |

| | | |
|---|---|---|
| ENSG00000130052 | STARD8 | blue |
| ENSG00000130158 | DOCK6 | blue |
| ENSG00000130159 | ECSIT | blue |
| ENSG00000130165 | ELOF1 | blue |
| ENSG00000130193 | THEM6 | blue |
| ENSG00000130204 | TOMM40 | blue |
| ENSG00000130255 | RPL36 | blue |
| ENSG00000130299 | GTPBP3 | blue |
| ENSG00000130305 | NSUN5 | blue |
| ENSG00000130312 | MRPL34 | blue |
| ENSG00000130332 | LSM7 | blue |
| ENSG00000130340 | SNX9 | blue |
| ENSG00000130347 | RTN4IP1 | blue |
| ENSG00000130396 | AFDN | blue |
| ENSG00000130413 | STK33 | blue |
| ENSG00000130520 | LSM4 | blue |
| ENSG00000130522 | JUND | blue |
| ENSG00000130560 | UBAC1 | blue |
| ENSG00000130643 | CALY | blue |
| ENSG00000130653 | PNPLA7 | blue |
| ENSG00000130684 | ZNF337 | blue |
| ENSG00000130699 | TAF4 | blue |
| ENSG00000130706 | ADRM1 | blue |
| ENSG00000130724 | CHMP2A | blue |
| ENSG00000130725 | UBE2M | blue |
| ENSG00000130726 | TRIM28 | blue |
| ENSG00000130731 | METTL26 | blue |
| ENSG00000130748 | TMEM160 | blue |
| ENSG00000130770 | ATPIF1 | blue |
| ENSG00000130775 | THEMIS2 | blue |
| ENSG00000130779 | CLIP1 | blue |
| ENSG00000130783 | CCDC62 | blue |
| ENSG00000130803 | ZNF317 | blue |
| ENSG00000130811 | EIF3G | blue |
| ENSG00000130844 | ZNF331 | blue |
| ENSG00000130921 | C12orf65 | blue |
| ENSG00000130939 | UBE4B | blue |
| ENSG00000130940 | CASZ1 | blue |
| ENSG00000130948 | HSD17B3 | blue |
| ENSG00000130958 | SLC35D2 | blue |
| ENSG00000130985 | UBA1 | blue |
| ENSG00000131042 | LILRB2 | blue |
| ENSG00000131043 | AAR2 | blue |
| ENSG00000131089 | ARHGEF9 | blue |
| ENSG00000131095 | GFAP | blue |
| ENSG00000131116 | ZNF428 | blue |
| ENSG00000131127 | ZNF141 | blue |
| ENSG00000131143 | COX4I1 | blue |

| | | |
|---|---|---|
| ENSG00000131148 | EMC8 | blue |
| ENSG00000131149 | GSE1 | blue |
| ENSG00000131153 | GINS2 | blue |
| ENSG00000131368 | MRPS25 | blue |
| ENSG00000131381 | RBSN | blue |
| ENSG00000131401 | NAPSB | blue |
| ENSG00000131467 | PSME3 | blue |
| ENSG00000131469 | RPL27 | blue |
| ENSG00000131475 | VPS25 | blue |
| ENSG00000131495 | NDUFA2 | blue |
| ENSG00000131504 | DIAPH1 | blue |
| ENSG00000131652 | THOC6 | blue |
| ENSG00000131697 | NPHP4 | blue |
| ENSG00000131746 | TNS4 | blue |
| ENSG00000131759 | RARA | blue |
| ENSG00000131779 | PEX11B | blue |
| ENSG00000131828 | PDHA1 | blue |
| ENSG00000131981 | LGALS3 | blue |
| ENSG00000132002 | DNAJB1 | blue |
| ENSG00000132122 | SPATA6 | blue |
| ENSG00000132185 | FCRLA | blue |
| ENSG00000132199 | ENOSF1 | blue |
| ENSG00000132205 | EMILIN2 | blue |
| ENSG00000132305 | IMMT | blue |
| ENSG00000132323 | ILKAP | blue |
| ENSG00000132329 | RAMP1 | blue |
| ENSG00000132361 | CLUH | blue |
| ENSG00000132394 | EEFSEC | blue |
| ENSG00000132405 | TBC1D14 | blue |
| ENSG00000132432 | SEC61G | blue |
| ENSG00000132471 | WBP2 | blue |
| ENSG00000132507 | EIF5A | blue |
| ENSG00000132510 | KDM6B | blue |
| ENSG00000132514 | CLEC10A | blue |
| ENSG00000132549 | VPS13B | blue |
| ENSG00000132570 | PCBD2 | blue |
| ENSG00000132589 | FLOT2 | blue |
| ENSG00000132591 | ERAL1 | blue |
| ENSG00000132604 | TERF2 | blue |
| ENSG00000132622 | HSPA12B | blue |
| ENSG00000132661 | NXT1 | blue |
| ENSG00000132676 | DAP3 | blue |
| ENSG00000132698 | RAB25 | blue |
| ENSG00000132744 | ACY3 | blue |
| ENSG00000132773 | TOE1 | blue |
| ENSG00000132781 | MUTYH | blue |
| ENSG00000132792 | CTNNBL1 | blue |
| ENSG00000132823 | OSER1 | blue |

| | | |
|---|---|---|
| ENSG00000132879 | FBXO44 | blue |
| ENSG00000132963 | POMP | blue |
| ENSG00000133059 | DSTYK | blue |
| ENSG00000133134 | BEX2 | blue |
| ENSG00000133250 | ZNF414 | blue |
| ENSG00000133256 | PDE6B | blue |
| ENSG00000133265 | HSPBP1 | blue |
| ENSG00000133315 | MACROD1 | blue |
| ENSG00000133316 | WDR74 | blue |
| ENSG00000133321 | RARRES3 | blue |
| ENSG00000133597 | ADCK2 | blue |
| ENSG00000133627 | ACTR3B | blue |
| ENSG00000133678 | TMEM254 | blue |
| ENSG00000133794 | ARNTL | blue |
| ENSG00000133805 | AMPD3 | blue |
| ENSG00000133874 | RNF122 | blue |
| ENSG00000133884 | DPF2 | blue |
| ENSG00000133935 | ERG28 | blue |
| ENSG00000134014 | ELP3 | blue |
| ENSG00000134028 | ADAMDEC1 | blue |
| ENSG00000134057 | CCNB1 | blue |
| ENSG00000134086 | VHL | blue |
| ENSG00000134153 | EMC7 | blue |
| ENSG00000134202 | GSTM3 | blue |
| ENSG00000134243 | SORT1 | blue |
| ENSG00000134256 | CD101 | blue |
| ENSG00000134278 | SPIRE1 | blue |
| ENSG00000134287 | ARF3 | blue |
| ENSG00000134291 | TMEM106C | blue |
| ENSG00000134490 | TMEM241 | blue |
| ENSG00000134508 | CABLES1 | blue |
| ENSG00000134516 | DOCK2 | blue |
| ENSG00000134590 | RTL8C | blue |
| ENSG00000134594 | RAB33A | blue |
| ENSG00000134668 | SPOCD1 | blue |
| ENSG00000134684 | YARS | blue |
| ENSG00000134686 | PHC2 | blue |
| ENSG00000134690 | CDCA8 | blue |
| ENSG00000134802 | SLC43A3 | blue |
| ENSG00000134825 | TMEM258 | blue |
| ENSG00000134897 | BIVM | blue |
| ENSG00000134899 | ERCC5 | blue |
| ENSG00000134901 | KDELC1 | blue |
| ENSG00000134905 | CARS2 | blue |
| ENSG00000135074 | ADAM19 | blue |
| ENSG00000135077 | HAVCR2 | blue |
| ENSG00000135083 | CCNJL | blue |
| ENSG00000135127 | BICDL1 | blue |

| | | |
|---|---|---|
| ENSG00000135144 | DTX1 | blue |
| ENSG00000135299 | ANKRD6 | blue |
| ENSG00000135318 | NT5E | blue |
| ENSG00000135390 | ATP5G2 | blue |
| ENSG00000135404 | CD63 | blue |
| ENSG00000135423 | GLS2 | blue |
| ENSG00000135446 | CDK4 | blue |
| ENSG00000135451 | TROAP | blue |
| ENSG00000135473 | PAN2 | blue |
| ENSG00000135476 | ESPL1 | blue |
| ENSG00000135506 | OS9 | blue |
| ENSG00000135622 | SEMA4F | blue |
| ENSG00000135624 | CCT7 | blue |
| ENSG00000135677 | GNS | blue |
| ENSG00000135686 | KLHL36 | blue |
| ENSG00000135720 | DYNC1LI2 | blue |
| ENSG00000135898 | GPR55 | blue |
| ENSG00000135913 | USP37 | blue |
| ENSG00000135919 | SERPINE2 | blue |
| ENSG00000135924 | DNAJB2 | blue |
| ENSG00000135925 | WNT10A | blue |
| ENSG00000135926 | TMBIM1 | blue |
| ENSG00000135930 | EIF4E2 | blue |
| ENSG00000135940 | COX5B | blue |
| ENSG00000135956 | TMEM127 | blue |
| ENSG00000136003 | ISCU | blue |
| ENSG00000136011 | STAB2 | blue |
| ENSG00000136026 | CKAP4 | blue |
| ENSG00000136044 | APPL2 | blue |
| ENSG00000136052 | SLC41A2 | blue |
| ENSG00000136149 | RPL13AP25 | blue |
| ENSG00000136205 | TNS3 | blue |
| ENSG00000136235 | GPNMB | blue |
| ENSG00000136238 | RAC1 | blue |
| ENSG00000136240 | KDELR2 | blue |
| ENSG00000136250 | AOAH | blue |
| ENSG00000136295 | TTYH3 | blue |
| ENSG00000136404 | TM6SF1 | blue |
| ENSG00000136444 | RSAD1 | blue |
| ENSG00000136463 | TACO1 | blue |
| ENSG00000136492 | BRIP1 | blue |
| ENSG00000136518 | ACTL6A | blue |
| ENSG00000136541 | ERMN | blue |
| ENSG00000136630 | HLX | blue |
| ENSG00000136631 | VPS45 | blue |
| ENSG00000136694 | IL36A | blue |
| ENSG00000136715 | SAP130 | blue |
| ENSG00000136717 | BIN1 | blue |

| | | |
|---|---|---|
| ENSG00000136718 | IMP4 | blue |
| ENSG00000136731 | UGGT1 | blue |
| ENSG00000136811 | ODF2 | blue |
| ENSG00000136828 | RALGPS1 | blue |
| ENSG00000136830 | FAM129B | blue |
| ENSG00000136856 | SLC2A8 | blue |
| ENSG00000136874 | STX17 | blue |
| ENSG00000136877 | FPGS | blue |
| ENSG00000136925 | TSTD2 | blue |
| ENSG00000136930 | PSMB7 | blue |
| ENSG00000136942 | RPL35 | blue |
| ENSG00000136950 | ARPC5L | blue |
| ENSG00000137076 | TLN1 | blue |
| ENSG00000137078 | SIT1 | blue |
| ENSG00000137100 | DCTN3 | blue |
| ENSG00000137101 | CD72 | blue |
| ENSG00000137103 | TMEM8B | blue |
| ENSG00000137106 | GRHPR | blue |
| ENSG00000137133 | HINT2 | blue |
| ENSG00000137154 | RPS6 | blue |
| ENSG00000137207 | YIPF3 | blue |
| ENSG00000137267 | TUBB2A | blue |
| ENSG00000137270 | GCM1 | blue |
| ENSG00000137309 | HMGA1 | blue |
| ENSG00000137337 | MDC1 | blue |
| ENSG00000137509 | PRCP | blue |
| ENSG00000137563 | GGH | blue |
| ENSG00000137656 | BUD13 | blue |
| ENSG00000137764 | MAP2K5 | blue |
| ENSG00000137801 | THBS1 | blue |
| ENSG00000137806 | NDUFAF1 | blue |
| ENSG00000137818 | RPLP1 | blue |
| ENSG00000137822 | TUBGCP4 | blue |
| ENSG00000138031 | ADCY3 | blue |
| ENSG00000138073 | PREB | blue |
| ENSG00000138081 | FBXO11 | blue |
| ENSG00000138085 | ATRAID | blue |
| ENSG00000138115 | CYP2C8 | blue |
| ENSG00000138161 | CUZD1 | blue |
| ENSG00000138279 | ANXA7 | blue |
| ENSG00000138311 | ZNF365 | blue |
| ENSG00000138363 | ATIC | blue |
| ENSG00000138375 | SMARCAL1 | blue |
| ENSG00000138395 | CDK15 | blue |
| ENSG00000138442 | WDR12 | blue |
| ENSG00000138495 | COX17 | blue |
| ENSG00000138592 | USP8 | blue |
| ENSG00000138621 | PPCDC | blue |

| | | |
|---|---|---|
| ENSG00000138629 | UBL7 | blue |
| ENSG00000138640 | FAM13A | blue |
| ENSG00000138641 | HERC3 | blue |
| ENSG00000138722 | MMRN1 | blue |
| ENSG00000138744 | NAAA | blue |
| ENSG00000138796 | HADH | blue |
| ENSG00000138829 | FBN2 | blue |
| ENSG00000138835 | RGS3 | blue |
| ENSG00000138942 | RNF185 | blue |
| ENSG00000139044 | B4GALNT3 | blue |
| ENSG00000139132 | FGD4 | blue |
| ENSG00000139160 | ETFBKMT | blue |
| ENSG00000139180 | NDUFA9 | blue |
| ENSG00000139193 | CD27 | blue |
| ENSG00000139194 | RBP5 | blue |
| ENSG00000139239 | AC078814.1 | blue |
| ENSG00000139266 | 9-Mar | blue |
| ENSG00000139343 | SNRPF | blue |
| ENSG00000139344 | AMDHD1 | blue |
| ENSG00000139405 | RITA1 | blue |
| ENSG00000139436 | GIT2 | blue |
| ENSG00000139546 | TARBP2 | blue |
| ENSG00000139579 | NABP2 | blue |
| ENSG00000139624 | CERS5 | blue |
| ENSG00000139631 | CSAD | blue |
| ENSG00000139637 | C12orf10 | blue |
| ENSG00000139644 | TMBIM6 | blue |
| ENSG00000139684 | ESD | blue |
| ENSG00000139832 | RAB20 | blue |
| ENSG00000139890 | REM2 | blue |
| ENSG00000139899 | CBLN3 | blue |
| ENSG00000139914 | FITM1 | blue |
| ENSG00000139990 | DCAF5 | blue |
| ENSG00000140043 | PTGR2 | blue |
| ENSG00000140264 | SERF2 | blue |
| ENSG00000140307 | GTF2A2 | blue |
| ENSG00000140365 | COMMD4 | blue |
| ENSG00000140382 | HMG20A | blue |
| ENSG00000140416 | TPM1 | blue |
| ENSG00000140451 | PIF1 | blue |
| ENSG00000140470 | ADAMTS17 | blue |
| ENSG00000140497 | SCAMP2 | blue |
| ENSG00000140553 | UNC45A | blue |
| ENSG00000140564 | FURIN | blue |
| ENSG00000140691 | ARMC5 | blue |
| ENSG00000140740 | UQCRC2 | blue |
| ENSG00000140830 | TXNL4B | blue |
| ENSG00000140876 | NUDT7 | blue |

| | | |
|---|---|---|
| ENSG00000140931 | CMTM3 | blue |
| ENSG00000140988 | RPS2 | blue |
| ENSG00000140990 | NDUFB10 | blue |
| ENSG00000140993 | TIGD7 | blue |
| ENSG00000141012 | GALNS | blue |
| ENSG00000141030 | COPS3 | blue |
| ENSG00000141040 | ZNF287 | blue |
| ENSG00000141068 | KSR1 | blue |
| ENSG00000141198 | TOM1L1 | blue |
| ENSG00000141293 | SKAP1 | blue |
| ENSG00000141349 | G6PC3 | blue |
| ENSG00000141449 | GREB1L | blue |
| ENSG00000141452 | C18orf8 | blue |
| ENSG00000141480 | ARRB2 | blue |
| ENSG00000141499 | WRAP53 | blue |
| ENSG00000141504 | SAT2 | blue |
| ENSG00000141505 | ASGR1 | blue |
| ENSG00000141510 | TP53 | blue |
| ENSG00000141522 | ARHGDIA | blue |
| ENSG00000141526 | SLC16A3 | blue |
| ENSG00000141543 | EIF4A3 | blue |
| ENSG00000141551 | CSNK1D | blue |
| ENSG00000141552 | ANAPC11 | blue |
| ENSG00000141556 | TBCD | blue |
| ENSG00000141562 | NARF | blue |
| ENSG00000141627 | DYM | blue |
| ENSG00000141644 | MBD1 | blue |
| ENSG00000141665 | FBXO15 | blue |
| ENSG00000141699 | RETREG3 | blue |
| ENSG00000141741 | MIEN1 | blue |
| ENSG00000141753 | IGFBP4 | blue |
| ENSG00000141759 | TXNL4A | blue |
| ENSG00000141837 | CACNA1A | blue |
| ENSG00000141854 | MISP3 | blue |
| ENSG00000141858 | SAMD1 | blue |
| ENSG00000141867 | BRD4 | blue |
| ENSG00000141959 | PFKL | blue |
| ENSG00000141968 | VAV1 | blue |
| ENSG00000142168 | SOD1 | blue |
| ENSG00000142185 | TRPM2 | blue |
| ENSG00000142227 | EMP3 | blue |
| ENSG00000142233 | NTN5 | blue |
| ENSG00000142252 | GEMIN7 | blue |
| ENSG00000142327 | RNPEPL1 | blue |
| ENSG00000142347 | MYO1F | blue |
| ENSG00000142507 | PSMB6 | blue |
| ENSG00000142534 | RPS11 | blue |
| ENSG00000142541 | RPL13A | blue |

| | | |
|---|---|---|
| ENSG00000142546 | NOSIP | blue |
| ENSG00000142634 | EFHD2 | blue |
| ENSG00000142657 | PGD | blue |
| ENSG00000142687 | KIAA0319L | blue |
| ENSG00000142937 | RPS8 | blue |
| ENSG00000143106 | PSMA5 | blue |
| ENSG00000143110 | C1orf162 | blue |
| ENSG00000143119 | CD53 | blue |
| ENSG00000143127 | ITGA10 | blue |
| ENSG00000143158 | MPC2 | blue |
| ENSG00000143179 | UCK2 | blue |
| ENSG00000143185 | XCL2 | blue |
| ENSG00000143198 | MGST3 | blue |
| ENSG00000143222 | UFC1 | blue |
| ENSG00000143314 | MRPL24 | blue |
| ENSG00000143315 | PIGM | blue |
| ENSG00000143319 | ISG20L2 | blue |
| ENSG00000143367 | TUFT1 | blue |
| ENSG00000143379 | SETDB1 | blue |
| ENSG00000143420 | ENSA | blue |
| ENSG00000143436 | MRPL9 | blue |
| ENSG00000143452 | HORMAD1 | blue |
| ENSG00000143476 | DTL | blue |
| ENSG00000143507 | DUSP10 | blue |
| ENSG00000143543 | JTB | blue |
| ENSG00000143549 | TPM3 | blue |
| ENSG00000143553 | SNAPIN | blue |
| ENSG00000143554 | SLC27A3 | blue |
| ENSG00000143569 | UBAP2L | blue |
| ENSG00000143570 | SLC39A1 | blue |
| ENSG00000143575 | HAX1 | blue |
| ENSG00000143590 | EFNA3 | blue |
| ENSG00000143603 | KCNN3 | blue |
| ENSG00000143621 | ILF2 | blue |
| ENSG00000143641 | GALNT2 | blue |
| ENSG00000143702 | CEP170 | blue |
| ENSG00000143761 | ARF1 | blue |
| ENSG00000143786 | CNIH3 | blue |
| ENSG00000143801 | PSEN2 | blue |
| ENSG00000143862 | ARL8A | blue |
| ENSG00000143919 | CAMKMT | blue |
| ENSG00000143947 | RPS27A | blue |
| ENSG00000144021 | CIAO1 | blue |
| ENSG00000144026 | ZNF514 | blue |
| ENSG00000144029 | MRPS5 | blue |
| ENSG00000144061 | NPHP1 | blue |
| ENSG00000144231 | POLR2D | blue |
| ENSG00000144283 | PKP4 | blue |

| | | |
|---|---|---|
| ENSG00000144290 | SLC4A10 | blue |
| ENSG00000144362 | PHOSPHO2 | blue |
| ENSG00000144369 | FAM171B | blue |
| ENSG00000144401 | METTL21A | blue |
| ENSG00000144445 | KANSL1L | blue |
| ENSG00000144455 | SUMF1 | blue |
| ENSG00000144468 | RHBDD1 | blue |
| ENSG00000144485 | HES6 | blue |
| ENSG00000144524 | COPS7B | blue |
| ENSG00000144567 | RETREG2 | blue |
| ENSG00000144580 | CNOT9 | blue |
| ENSG00000144591 | GMPPA | blue |
| ENSG00000144659 | SLC25A38 | blue |
| ENSG00000144713 | RPL32 | blue |
| ENSG00000144843 | ADPRH | blue |
| ENSG00000145012 | LPP | blue |
| ENSG00000145103 | ILDR1 | blue |
| ENSG00000145191 | EIF2B5 | blue |
| ENSG00000145194 | ECE2 | blue |
| ENSG00000145216 | FIP1L1 | blue |
| ENSG00000145247 | OCIAD2 | blue |
| ENSG00000145491 | ROPN1L | blue |
| ENSG00000145494 | NDUFS6 | blue |
| ENSG00000145592 | RPL37 | blue |
| ENSG00000145757 | SPATA9 | blue |
| ENSG00000145908 | ZNF300 | blue |
| ENSG00000145912 | NHP2 | blue |
| ENSG00000145982 | FARS2 | blue |
| ENSG00000146066 | HIGD2A | blue |
| ENSG00000146070 | PLA2G7 | blue |
| ENSG00000146094 | DOK3 | blue |
| ENSG00000146109 | ABT1 | blue |
| ENSG00000146215 | CRIP3 | blue |
| ENSG00000146232 | NFKBIE | blue |
| ENSG00000146243 | IRAK1BP1 | blue |
| ENSG00000146530 | VWDE | blue |
| ENSG00000146535 | GNA12 | blue |
| ENSG00000146676 | PURB | blue |
| ENSG00000146701 | MDH2 | blue |
| ENSG00000146776 | ATXN7L1 | blue |
| ENSG00000146834 | MEPCE | blue |
| ENSG00000147010 | SH3KBP1 | blue |
| ENSG00000147044 | CASK | blue |
| ENSG00000147065 | MSN | blue |
| ENSG00000147117 | ZNF157 | blue |
| ENSG00000147123 | NDUFB11 | blue |
| ENSG00000147155 | EBP | blue |
| ENSG00000147164 | SNX12 | blue |

| | | |
|---|---|---|
| ENSG00000147168 | IL2RG | blue |
| ENSG00000147383 | NSDHL | blue |
| ENSG00000147403 | RPL10 | blue |
| ENSG00000147439 | BIN3 | blue |
| ENSG00000147443 | DOK2 | blue |
| ENSG00000147526 | TACC1 | blue |
| ENSG00000147536 | GINS4 | blue |
| ENSG00000147677 | EIF3H | blue |
| ENSG00000147684 | NDUFB9 | blue |
| ENSG00000148057 | IDNK | blue |
| ENSG00000148158 | SNX30 | blue |
| ENSG00000148180 | GSN | blue |
| ENSG00000148303 | RPL7A | blue |
| ENSG00000148308 | GTF3C5 | blue |
| ENSG00000148362 | PAXX | blue |
| ENSG00000148396 | SEC16A | blue |
| ENSG00000148450 | MSRB2 | blue |
| ENSG00000148516 | ZEB1 | blue |
| ENSG00000148688 | RPP30 | blue |
| ENSG00000148730 | EIF4EBP2 | blue |
| ENSG00000148803 | FUOM | blue |
| ENSG00000148834 | GSTO1 | blue |
| ENSG00000149016 | TUT1 | blue |
| ENSG00000149150 | SLC43A1 | blue |
| ENSG00000149182 | ARFGAP2 | blue |
| ENSG00000149187 | CELF1 | blue |
| ENSG00000149196 | HIKESHI | blue |
| ENSG00000149260 | CAPN5 | blue |
| ENSG00000149273 | RPS3 | blue |
| ENSG00000149289 | ZC3H12C | blue |
| ENSG00000149292 | TTC12 | blue |
| ENSG00000149357 | LAMTOR1 | blue |
| ENSG00000149476 | TKFC | blue |
| ENSG00000149480 | MTA2 | blue |
| ENSG00000149485 | FADS1 | blue |
| ENSG00000149541 | B3GAT3 | blue |
| ENSG00000149554 | CHEK1 | blue |
| ENSG00000149577 | SIDT2 | blue |
| ENSG00000149600 | COMMD7 | blue |
| ENSG00000149679 | CABLES2 | blue |
| ENSG00000149781 | FERMT3 | blue |
| ENSG00000149792 | MRPL49 | blue |
| ENSG00000149806 | FAU | blue |
| ENSG00000149809 | TM7SF2 | blue |
| ENSG00000149823 | VPS51 | blue |
| ENSG00000149929 | HIRIP3 | blue |
| ENSG00000149932 | TMEM219 | blue |
| ENSG00000150048 | CLEC1A | blue |

| | | |
|---|---|---|
| ENSG00000150401 | DCUN1D2 | blue |
| ENSG00000150403 | TMCO3 | blue |
| ENSG00000150456 | EEF1AKMT1 | blue |
| ENSG00000150477 | KIAA1328 | blue |
| ENSG00000151062 | CACNA2D4 | blue |
| ENSG00000151148 | UBE3B | blue |
| ENSG00000151240 | DIP2C | blue |
| ENSG00000151366 | NDUFC2 | blue |
| ENSG00000151474 | FRMD4A | blue |
| ENSG00000151498 | ACAD8 | blue |
| ENSG00000151500 | THYN1 | blue |
| ENSG00000151575 | TEX9 | blue |
| ENSG00000151612 | ZNF827 | blue |
| ENSG00000151623 | NR3C2 | blue |
| ENSG00000151640 | DPYSL4 | blue |
| ENSG00000151650 | VENTX | blue |
| ENSG00000151651 | ADAM8 | blue |
| ENSG00000151693 | ASAP2 | blue |
| ENSG00000151694 | ADAM17 | blue |
| ENSG00000151743 | AMN1 | blue |
| ENSG00000151773 | CCDC122 | blue |
| ENSG00000151778 | SERP2 | blue |
| ENSG00000151779 | NBAS | blue |
| ENSG00000151789 | ZNF385D | blue |
| ENSG00000151806 | GUF1 | blue |
| ENSG00000151876 | FBXO4 | blue |
| ENSG00000151948 | GLT1D1 | blue |
| ENSG00000152056 | AP1S3 | blue |
| ENSG00000152082 | MZT2B | blue |
| ENSG00000152234 | ATP5A1 | blue |
| ENSG00000152242 | C18orf25 | blue |
| ENSG00000152256 | PDK1 | blue |
| ENSG00000152315 | KCNK13 | blue |
| ENSG00000152377 | SPOCK1 | blue |
| ENSG00000152413 | HOMER1 | blue |
| ENSG00000152433 | ZNF547 | blue |
| ENSG00000152443 | ZNF776 | blue |
| ENSG00000152455 | SUV39H2 | blue |
| ENSG00000152457 | DCLRE1C | blue |
| ENSG00000152527 | PLEKHH2 | blue |
| ENSG00000152767 | FARP1 | blue |
| ENSG00000152782 | PANK1 | blue |
| ENSG00000152784 | PRDM8 | blue |
| ENSG00000152953 | STK32B | blue |
| ENSG00000153066 | TXNDC11 | blue |
| ENSG00000153208 | MERTK | blue |
| ENSG00000153283 | CD96 | blue |
| ENSG00000153391 | INO80C | blue |

| | | |
|---|---|---|
| ENSG00000153551 | CMTM7 | blue |
| ENSG00000153563 | CD8A | blue |
| ENSG00000153786 | ZDHHC7 | blue |
| ENSG00000153896 | ZNF599 | blue |
| ENSG00000153933 | DGKE | blue |
| ENSG00000153976 | HS3ST3A1 | blue |
| ENSG00000154079 | SDHAF4 | blue |
| ENSG00000154124 | OTULIN | blue |
| ENSG00000154144 | TBRG1 | blue |
| ENSG00000154188 | ANGPT1 | blue |
| ENSG00000154227 | CERS3 | blue |
| ENSG00000154473 | BUB3 | blue |
| ENSG00000154518 | ATP5G3 | blue |
| ENSG00000154608 | CEP170P1 | blue |
| ENSG00000154743 | TSEN2 | blue |
| ENSG00000154781 | CCDC174 | blue |
| ENSG00000154832 | CXXC1 | blue |
| ENSG00000154930 | ACSS1 | blue |
| ENSG00000154957 | ZNF18 | blue |
| ENSG00000154978 | VOPP1 | blue |
| ENSG00000155085 | AK9 | blue |
| ENSG00000155115 | GTF3C6 | blue |
| ENSG00000155252 | PI4K2A | blue |
| ENSG00000155463 | OXA1L | blue |
| ENSG00000155465 | SLC7A7 | blue |
| ENSG00000155744 | FAM126B | blue |
| ENSG00000155849 | ELMO1 | blue |
| ENSG00000155966 | AFF2 | blue |
| ENSG00000155974 | GRIP1 | blue |
| ENSG00000155980 | KIF5A | blue |
| ENSG00000156030 | ELMSAN1 | blue |
| ENSG00000156127 | BATF | blue |
| ENSG00000156239 | N6AMT1 | blue |
| ENSG00000156414 | TDRD9 | blue |
| ENSG00000156471 | PTDSS1 | blue |
| ENSG00000156510 | HKDC1 | blue |
| ENSG00000156599 | ZDHHC5 | blue |
| ENSG00000156697 | UTP14A | blue |
| ENSG00000156709 | AIFM1 | blue |
| ENSG00000156873 | PHKG2 | blue |
| ENSG00000156928 | MALSU1 | blue |
| ENSG00000156983 | BRPF1 | blue |
| ENSG00000157014 | TATDN2 | blue |
| ENSG00000157020 | SEC13 | blue |
| ENSG00000157110 | RBPMS | blue |
| ENSG00000157152 | SYN2 | blue |
| ENSG00000157184 | CPT2 | blue |
| ENSG00000157191 | NECAP2 | blue |

| | | |
|---|---|---|
| ENSG00000157216 | SSBP3 | blue |
| ENSG00000157326 | DHRS4 | blue |
| ENSG00000157350 | ST3GAL2 | blue |
| ENSG00000157379 | DHRS1 | blue |
| ENSG00000157445 | CACNA2D3 | blue |
| ENSG00000157483 | MYO1E | blue |
| ENSG00000157538 | DSCR3 | blue |
| ENSG00000157578 | LCA5L | blue |
| ENSG00000157593 | SLC35B2 | blue |
| ENSG00000157637 | SLC38A10 | blue |
| ENSG00000157778 | PSMG3 | blue |
| ENSG00000157837 | SPPL3 | blue |
| ENSG00000157856 | DRC1 | blue |
| ENSG00000157895 | C12orf43 | blue |
| ENSG00000158156 | XKR8 | blue |
| ENSG00000158234 | FAIM | blue |
| ENSG00000158286 | RNF207 | blue |
| ENSG00000158402 | CDC25C | blue |
| ENSG00000158427 | TMSB15B | blue |
| ENSG00000158457 | TSPAN33 | blue |
| ENSG00000158473 | CD1D | blue |
| ENSG00000158517 | NCF1 | blue |
| ENSG00000158526 | TSR2 | blue |
| ENSG00000158545 | ZC3H18 | blue |
| ENSG00000158623 | COPG2 | blue |
| ENSG00000158683 | PKD1L1 | blue |
| ENSG00000158710 | TAGLN2 | blue |
| ENSG00000158747 | NBL1 | blue |
| ENSG00000158773 | USF1 | blue |
| ENSG00000158793 | NIT1 | blue |
| ENSG00000158796 | DEDD | blue |
| ENSG00000158825 | CDA | blue |
| ENSG00000158850 | B4GALT3 | blue |
| ENSG00000158864 | NDUFS2 | blue |
| ENSG00000158869 | FCER1G | blue |
| ENSG00000158941 | CCAR2 | blue |
| ENSG00000159063 | ALG8 | blue |
| ENSG00000159079 | C21orf59 | blue |
| ENSG00000159110 | IFNAR2 | blue |
| ENSG00000159111 | MRPL10 | blue |
| ENSG00000159199 | ATP5G1 | blue |
| ENSG00000159210 | SNF8 | blue |
| ENSG00000159231 | CBR3 | blue |
| ENSG00000159259 | CHAF1B | blue |
| ENSG00000159267 | HLCS | blue |
| ENSG00000159335 | PTMS | blue |
| ENSG00000159348 | CYB5R1 | blue |
| ENSG00000159352 | PSMD4 | blue |

| | | |
|---|---|---|
| ENSG00000159377 | PSMB4 | blue |
| ENSG00000159423 | ALDH4A1 | blue |
| ENSG00000159445 | THEM4 | blue |
| ENSG00000159479 | MED8 | blue |
| ENSG00000159496 | RGL4 | blue |
| ENSG00000159625 | DRC7 | blue |
| ENSG00000159648 | TEPP | blue |
| ENSG00000159685 | CHCHD6 | blue |
| ENSG00000159692 | CTBP1 | blue |
| ENSG00000159720 | ATP6V0D1 | blue |
| ENSG00000159753 | CARMIL2 | blue |
| ENSG00000159840 | ZYX | blue |
| ENSG00000159899 | NPR2 | blue |
| ENSG00000159917 | ZNF235 | blue |
| ENSG00000159958 | TNFRSF13C | blue |
| ENSG00000160013 | PTGIR | blue |
| ENSG00000160055 | TMEM234 | blue |
| ENSG00000160058 | BSDC1 | blue |
| ENSG00000160075 | SSU72 | blue |
| ENSG00000160094 | ZNF362 | blue |
| ENSG00000160199 | PKNOX1 | blue |
| ENSG00000160211 | G6PD | blue |
| ENSG00000160213 | CSTB | blue |
| ENSG00000160214 | RRP1 | blue |
| ENSG00000160218 | TRAPPC10 | blue |
| ENSG00000160223 | ICOSLG | blue |
| ENSG00000160229 | ZNF66 | blue |
| ENSG00000160233 | LRRC3 | blue |
| ENSG00000160255 | ITGB2 | blue |
| ENSG00000160256 | FAM207A | blue |
| ENSG00000160294 | MCM3AP | blue |
| ENSG00000160305 | DIP2A | blue |
| ENSG00000160321 | ZNF208 | blue |
| ENSG00000160352 | ZNF714 | blue |
| ENSG00000160460 | SPTBN4 | blue |
| ENSG00000160563 | MED27 | blue |
| ENSG00000160570 | DEDD2 | blue |
| ENSG00000160584 | SIK3 | blue |
| ENSG00000160593 | JAML | blue |
| ENSG00000160633 | SAFB | blue |
| ENSG00000160654 | CD3G | blue |
| ENSG00000160688 | FLAD1 | blue |
| ENSG00000160714 | UBE2Q1 | blue |
| ENSG00000160752 | FDPS | blue |
| ENSG00000160783 | PMF1 | blue |
| ENSG00000160789 | LMNA | blue |
| ENSG00000160799 | CCDC12 | blue |
| ENSG00000160803 | UBQLN4 | blue |

| | | |
|---|---|---|
| ENSG00000160813 | PPP1R35 | blue |
| ENSG00000160818 | GPATCH4 | blue |
| ENSG00000160883 | HK3 | blue |
| ENSG00000160888 | IER2 | blue |
| ENSG00000160917 | CPSF4 | blue |
| ENSG00000160963 | COL26A1 | blue |
| ENSG00000161016 | RPL8 | blue |
| ENSG00000161040 | FBXL13 | blue |
| ENSG00000161132 | AC007663.1 | blue |
| ENSG00000161179 | YDJC | blue |
| ENSG00000161203 | AP2M1 | blue |
| ENSG00000161249 | DMKN | blue |
| ENSG00000161395 | PGAP3 | blue |
| ENSG00000161513 | FDXR | blue |
| ENSG00000161533 | ACOX1 | blue |
| ENSG00000161551 | ZNF577 | blue |
| ENSG00000161618 | ALDH16A1 | blue |
| ENSG00000161671 | EMC10 | blue |
| ENSG00000161677 | JOSD2 | blue |
| ENSG00000161888 | SPC24 | blue |
| ENSG00000161912 | ADCY10P1 | blue |
| ENSG00000161921 | CXCL16 | blue |
| ENSG00000161929 | SCIMP | blue |
| ENSG00000161955 | TNFSF13 | blue |
| ENSG00000161956 | SENP3 | blue |
| ENSG00000161973 | CCDC42 | blue |
| ENSG00000161980 | POLR3K | blue |
| ENSG00000162062 | C16orf59 | blue |
| ENSG00000162063 | CCNF | blue |
| ENSG00000162076 | FLYWCH2 | blue |
| ENSG00000162104 | ADCY9 | blue |
| ENSG00000162174 | ASRGL1 | blue |
| ENSG00000162191 | UBXN1 | blue |
| ENSG00000162244 | RPL29 | blue |
| ENSG00000162337 | LRP5 | blue |
| ENSG00000162373 | BEND5 | blue |
| ENSG00000162408 | NOL9 | blue |
| ENSG00000162415 | ZSWIM5 | blue |
| ENSG00000162419 | GMEB1 | blue |
| ENSG00000162437 | RAVER2 | blue |
| ENSG00000162517 | PEF1 | blue |
| ENSG00000162526 | TSSK3 | blue |
| ENSG00000162543 | UBXN10 | blue |
| ENSG00000162551 | ALPL | blue |
| ENSG00000162585 | FAAP20 | blue |
| ENSG00000162599 | NFIA | blue |
| ENSG00000162711 | NLRP3 | blue |
| ENSG00000162729 | IGSF8 | blue |

| | | |
|---|---|---|
| ENSG00000162734 | PEA15 | blue |
| ENSG00000162735 | PEX19 | blue |
| ENSG00000162736 | NCSTN | blue |
| ENSG00000162739 | SLAMF6 | blue |
| ENSG00000162755 | KLHDC9 | blue |
| ENSG00000162757 | C1orf74 | blue |
| ENSG00000162873 | KLHDC8A | blue |
| ENSG00000162889 | MAPKAPK2 | blue |
| ENSG00000162910 | MRPL55 | blue |
| ENSG00000162913 | OBSCN-AS1 | blue |
| ENSG00000162929 | KIAA1841 | blue |
| ENSG00000162994 | CLHC1 | blue |
| ENSG00000162997 | PRORSD1P | blue |
| ENSG00000163159 | VPS72 | blue |
| ENSG00000163170 | BOLA3 | blue |
| ENSG00000163191 | S100A11 | blue |
| ENSG00000163221 | S100A12 | blue |
| ENSG00000163344 | PMVK | blue |
| ENSG00000163382 | NAXE | blue |
| ENSG00000163453 | IGFBP7 | blue |
| ENSG00000163464 | CXCR1 | blue |
| ENSG00000163466 | ARPC2 | blue |
| ENSG00000163467 | TSACC | blue |
| ENSG00000163468 | CCT3 | blue |
| ENSG00000163479 | SSR2 | blue |
| ENSG00000163481 | RNF25 | blue |
| ENSG00000163492 | CCDC141 | blue |
| ENSG00000163528 | CHCHD4 | blue |
| ENSG00000163534 | FCRL1 | blue |
| ENSG00000163545 | NUAK2 | blue |
| ENSG00000163596 | ICA1L | blue |
| ENSG00000163624 | CDS1 | blue |
| ENSG00000163697 | APBB2 | blue |
| ENSG00000163794 | UCN | blue |
| ENSG00000163803 | PLB1 | blue |
| ENSG00000163812 | ZDHHC3 | blue |
| ENSG00000163832 | ELP6 | blue |
| ENSG00000163864 | NMNAT3 | blue |
| ENSG00000163882 | POLR2H | blue |
| ENSG00000163885 | CFAP100 | blue |
| ENSG00000163900 | TMEM41A | blue |
| ENSG00000163935 | SFMBT1 | blue |
| ENSG00000163960 | UBXN7 | blue |
| ENSG00000164038 | SLC9B2 | blue |
| ENSG00000164051 | CCDC51 | blue |
| ENSG00000164062 | APEH | blue |
| ENSG00000164066 | INTU | blue |
| ENSG00000164105 | SAP30 | blue |

| | | |
|---|---|---|
| ENSG00000164142 | FAM160A1 | blue |
| ENSG00000164167 | LSM6 | blue |
| ENSG00000164182 | NDUFAF2 | blue |
| ENSG00000164300 | SERINC5 | blue |
| ENSG00000164402 | 8-Sep | blue |
| ENSG00000164403 | SHROOM1 | blue |
| ENSG00000164404 | GDF9 | blue |
| ENSG00000164466 | SFXN1 | blue |
| ENSG00000164530 | PI16 | blue |
| ENSG00000164556 | FAM183BP | blue |
| ENSG00000164587 | RPS14 | blue |
| ENSG00000164610 | RP9 | blue |
| ENSG00000164611 | PTTG1 | blue |
| ENSG00000164638 | SLC29A4 | blue |
| ENSG00000164683 | HEY1 | blue |
| ENSG00000164713 | BRI3 | blue |
| ENSG00000164733 | CTSB | blue |
| ENSG00000164885 | CDK5 | blue |
| ENSG00000164896 | FASTK | blue |
| ENSG00000164897 | TMUB1 | blue |
| ENSG00000164953 | TMEM67 | blue |
| ENSG00000164967 | RPP25L | blue |
| ENSG00000164972 | C9orf24 | blue |
| ENSG00000164975 | SNAPC3 | blue |
| ENSG00000165025 | SYK | blue |
| ENSG00000165028 | NIPSNAP3B | blue |
| ENSG00000165060 | FXN | blue |
| ENSG00000165140 | FBP1 | blue |
| ENSG00000165175 | MID1IP1 | blue |
| ENSG00000165178 | NCF1C | blue |
| ENSG00000165264 | NDUFB6 | blue |
| ENSG00000165275 | TRMT10B | blue |
| ENSG00000165280 | VCP | blue |
| ENSG00000165283 | STOML2 | blue |
| ENSG00000165288 | BRWD3 | blue |
| ENSG00000165392 | WRN | blue |
| ENSG00000165457 | FOLR2 | blue |
| ENSG00000165494 | PCF11 | blue |
| ENSG00000165525 | NEMF | blue |
| ENSG00000165548 | TMEM63C | blue |
| ENSG00000165555 | NOXRED1 | blue |
| ENSG00000165568 | AKR1E2 | blue |
| ENSG00000165637 | VDAC2 | blue |
| ENSG00000165688 | PMPCA | blue |
| ENSG00000165699 | TSC1 | blue |
| ENSG00000165702 | GFI1B | blue |
| ENSG00000165724 | ZMYND19 | blue |
| ENSG00000165879 | FRAT1 | blue |

| | | |
|---|---|---|
| ENSG00000165898 | ISCA2 | blue |
| ENSG00000165905 | LARGE2 | blue |
| ENSG00000165916 | PSMC3 | blue |
| ENSG00000165943 | MOAP1 | blue |
| ENSG00000166004 | CEP295 | blue |
| ENSG00000166033 | HTRA1 | blue |
| ENSG00000166046 | TCP11L2 | blue |
| ENSG00000166133 | RPUSD2 | blue |
| ENSG00000166136 | NDUFB8 | blue |
| ENSG00000166165 | CKB | blue |
| ENSG00000166166 | TRMT61A | blue |
| ENSG00000166171 | DPCD | blue |
| ENSG00000166189 | HPS6 | blue |
| ENSG00000166199 | ALKBH3 | blue |
| ENSG00000166224 | SGPL1 | blue |
| ENSG00000166228 | PCBD1 | blue |
| ENSG00000166260 | COX11 | blue |
| ENSG00000166265 | CYYR1 | blue |
| ENSG00000166321 | NUDT13 | blue |
| ENSG00000166337 | TAF10 | blue |
| ENSG00000166347 | CYB5A | blue |
| ENSG00000166387 | PPFIBP2 | blue |
| ENSG00000166405 | RIC3 | blue |
| ENSG00000166428 | PLD4 | blue |
| ENSG00000166439 | RNF169 | blue |
| ENSG00000166441 | RPL27A | blue |
| ENSG00000166452 | AKIP1 | blue |
| ENSG00000166508 | MCM7 | blue |
| ENSG00000166557 | TMED3 | blue |
| ENSG00000166595 | FAM96B | blue |
| ENSG00000166682 | TMPRSS5 | blue |
| ENSG00000166704 | ZNF606 | blue |
| ENSG00000166707 | ZCCHC18 | blue |
| ENSG00000166743 | ACSM1 | blue |
| ENSG00000166770 | ZNF667-AS1 | blue |
| ENSG00000166794 | PPIB | blue |
| ENSG00000166813 | KIF7 | blue |
| ENSG00000166823 | MESP1 | blue |
| ENSG00000166825 | ANPEP | blue |
| ENSG00000166847 | DCTN5 | blue |
| ENSG00000166851 | PLK1 | blue |
| ENSG00000166902 | MRPL16 | blue |
| ENSG00000166913 | YWHAB | blue |
| ENSG00000166925 | TSC22D4 | blue |
| ENSG00000166963 | MAP1A | blue |
| ENSG00000166965 | RCCD1 | blue |
| ENSG00000166986 | MARS | blue |
| ENSG00000167083 | GNGT2 | blue |

| | | |
|---|---|---|
| ENSG00000167085 | PHB | blue |
| ENSG00000167112 | TRUB2 | blue |
| ENSG00000167118 | URM1 | blue |
| ENSG00000167130 | DOLPP1 | blue |
| ENSG00000167136 | ENDOG | blue |
| ENSG00000167173 | C15orf39 | blue |
| ENSG00000167182 | SP2 | blue |
| ENSG00000167193 | CRK | blue |
| ENSG00000167202 | TBC1D2B | blue |
| ENSG00000167220 | HDHD2 | blue |
| ENSG00000167244 | IGF2 | blue |
| ENSG00000167272 | POP5 | blue |
| ENSG00000167286 | CD3D | blue |
| ENSG00000167377 | ZNF23 | blue |
| ENSG00000167397 | VKORC1 | blue |
| ENSG00000167468 | GPX4 | blue |
| ENSG00000167491 | GATAD2A | blue |
| ENSG00000167508 | MVD | blue |
| ENSG00000167515 | TRAPPC2L | blue |
| ENSG00000167523 | SPATA33 | blue |
| ENSG00000167526 | RPL13 | blue |
| ENSG00000167536 | DHRS13 | blue |
| ENSG00000167550 | RHEBL1 | blue |
| ENSG00000167552 | TUBA1A | blue |
| ENSG00000167554 | ZNF610 | blue |
| ENSG00000167562 | ZNF701 | blue |
| ENSG00000167565 | SERTAD3 | blue |
| ENSG00000167632 | TRAPPC9 | blue |
| ENSG00000167641 | PPP1R14A | blue |
| ENSG00000167642 | SPINT2 | blue |
| ENSG00000167645 | YIF1B | blue |
| ENSG00000167658 | EEF2 | blue |
| ENSG00000167670 | CHAF1A | blue |
| ENSG00000167674 | HDGFL2 | blue |
| ENSG00000167680 | SEMA6B | blue |
| ENSG00000167685 | ZNF444 | blue |
| ENSG00000167700 | MFSD3 | blue |
| ENSG00000167747 | C19orf48 | blue |
| ENSG00000167766 | ZNF83 | blue |
| ENSG00000167770 | OTUB1 | blue |
| ENSG00000167775 | CD320 | blue |
| ENSG00000167792 | NDUFV1 | blue |
| ENSG00000167797 | CDK2AP2 | blue |
| ENSG00000167799 | NUDT8 | blue |
| ENSG00000167815 | PRDX2 | blue |
| ENSG00000167850 | CD300C | blue |
| ENSG00000167851 | CD300A | blue |
| ENSG00000167862 | MRPL58 | blue |

| | | |
|---|---|---|
| ENSG00000167863 | ATP5H | blue |
| ENSG00000167881 | SRP68 | blue |
| ENSG00000167914 | GSDMA | blue |
| ENSG00000167920 | TMEM99 | blue |
| ENSG00000167925 | GHDC | blue |
| ENSG00000167930 | FAM234A | blue |
| ENSG00000167965 | MLST8 | blue |
| ENSG00000167969 | ECI1 | blue |
| ENSG00000167984 | NLRC3 | blue |
| ENSG00000167985 | SDHAF2 | blue |
| ENSG00000167992 | VWCE | blue |
| ENSG00000168002 | POLR2G | blue |
| ENSG00000168003 | SLC3A2 | blue |
| ENSG00000168005 | C11orf84 | blue |
| ENSG00000168014 | C2CD3 | blue |
| ENSG00000168028 | RPSA | blue |
| ENSG00000168061 | SAC3D1 | blue |
| ENSG00000168079 | SCARA5 | blue |
| ENSG00000168081 | PNOC | blue |
| ENSG00000168090 | COPS6 | blue |
| ENSG00000168096 | ANKS3 | blue |
| ENSG00000168101 | NUDT16L1 | blue |
| ENSG00000168159 | RNF187 | blue |
| ENSG00000168256 | NKIRAS2 | blue |
| ENSG00000168273 | SMIM4 | blue |
| ENSG00000168291 | PDHB | blue |
| ENSG00000168297 | PXK | blue |
| ENSG00000168393 | DTYMK | blue |
| ENSG00000168395 | ING5 | blue |
| ENSG00000168421 | RHOH | blue |
| ENSG00000168439 | STIP1 | blue |
| ENSG00000168476 | REEP4 | blue |
| ENSG00000168575 | SLC20A2 | blue |
| ENSG00000168591 | TMUB2 | blue |
| ENSG00000168615 | ADAM9 | blue |
| ENSG00000168653 | NDUFS5 | blue |
| ENSG00000168672 | FAM84B | blue |
| ENSG00000168675 | LDLRAD4 | blue |
| ENSG00000168701 | TMEM208 | blue |
| ENSG00000168734 | PKIG | blue |
| ENSG00000168778 | TCTN2 | blue |
| ENSG00000168792 | ABHD15 | blue |
| ENSG00000168807 | SNTB2 | blue |
| ENSG00000168811 | IL12A | blue |
| ENSG00000168818 | STX18 | blue |
| ENSG00000168874 | ATOH8 | blue |
| ENSG00000168883 | USP39 | blue |
| ENSG00000168894 | RNF181 | blue |

| | | |
|---|---|---|
| ENSG00000168899 | VAMP5 | blue |
| ENSG00000168906 | MAT2A | blue |
| ENSG00000168916 | ZNF608 | blue |
| ENSG00000168936 | TMEM129 | blue |
| ENSG00000168995 | SIGLEC7 | blue |
| ENSG00000169020 | ATP5I | blue |
| ENSG00000169026 | MFSD7 | blue |
| ENSG00000169031 | COL4A3 | blue |
| ENSG00000169084 | DHRSX | blue |
| ENSG00000169100 | SLC25A6 | blue |
| ENSG00000169105 | CHST14 | blue |
| ENSG00000169122 | FAM110B | blue |
| ENSG00000169180 | XPO6 | blue |
| ENSG00000169188 | APEX2 | blue |
| ENSG00000169189 | NSMCE1 | blue |
| ENSG00000169230 | PRELID1 | blue |
| ENSG00000169239 | CA5B | blue |
| ENSG00000169241 | SLC50A1 | blue |
| ENSG00000169249 | ZRSR2 | blue |
| ENSG00000169371 | SNUPN | blue |
| ENSG00000169385 | RNASE2 | blue |
| ENSG00000169397 | RNASE3 | blue |
| ENSG00000169403 | PTAFR | blue |
| ENSG00000169413 | RNASE6 | blue |
| ENSG00000169435 | RASSF6 | blue |
| ENSG00000169508 | GPR183 | blue |
| ENSG00000169564 | PCBP1 | blue |
| ENSG00000169627 | BOLA2B | blue |
| ENSG00000169629 | RGPD8 | blue |
| ENSG00000169683 | LRRC45 | blue |
| ENSG00000169689 | CENPX | blue |
| ENSG00000169696 | ASPSCR1 | blue |
| ENSG00000169718 | DUS1L | blue |
| ENSG00000169727 | GPS1 | blue |
| ENSG00000169733 | RFNG | blue |
| ENSG00000169738 | DCXR | blue |
| ENSG00000169857 | AVEN | blue |
| ENSG00000169896 | ITGAM | blue |
| ENSG00000169919 | GUSB | blue |
| ENSG00000169955 | ZNF747 | blue |
| ENSG00000169976 | SF3B5 | blue |
| ENSG00000170006 | TMEM154 | blue |
| ENSG00000170011 | MYRIP | blue |
| ENSG00000170037 | CNTROB | blue |
| ENSG00000170043 | TRAPPC1 | blue |
| ENSG00000170161 | AL512625.1 | blue |
| ENSG00000170260 | ZNF212 | blue |
| ENSG00000170266 | GLB1 | blue |

| | | |
|---|---|---|
| ENSG00000170275 | CRTAP | blue |
| ENSG00000170291 | ELP5 | blue |
| ENSG00000170310 | STX8 | blue |
| ENSG00000170412 | GPRC5C | blue |
| ENSG00000170421 | KRT8 | blue |
| ENSG00000170430 | MGMT | blue |
| ENSG00000170458 | CD14 | blue |
| ENSG00000170464 | DNAJC18 | blue |
| ENSG00000170473 | PYM1 | blue |
| ENSG00000170485 | NPAS2 | blue |
| ENSG00000170486 | KRT72 | blue |
| ENSG00000170515 | PA2G4 | blue |
| ENSG00000170571 | EMB | blue |
| ENSG00000170627 | GTSF1 | blue |
| ENSG00000170629 | DPY19L2P2 | blue |
| ENSG00000170634 | ACYP2 | blue |
| ENSG00000170819 | BFSP2 | blue |
| ENSG00000170891 | CYTL1 | blue |
| ENSG00000170921 | TANC2 | blue |
| ENSG00000170955 | CAVIN3 | blue |
| ENSG00000170956 | CEACAM3 | blue |
| ENSG00000171044 | XKR6 | blue |
| ENSG00000171051 | FPR1 | blue |
| ENSG00000171056 | SOX7 | blue |
| ENSG00000171105 | INSR | blue |
| ENSG00000171135 | JAGN1 | blue |
| ENSG00000171159 | C9orf16 | blue |
| ENSG00000171206 | TRIM8 | blue |
| ENSG00000171222 | SCAND1 | blue |
| ENSG00000171236 | LRG1 | blue |
| ENSG00000171302 | CANT1 | blue |
| ENSG00000171311 | EXOSC1 | blue |
| ENSG00000171385 | KCND3 | blue |
| ENSG00000171421 | MRPL36 | blue |
| ENSG00000171425 | ZNF581 | blue |
| ENSG00000171443 | ZNF524 | blue |
| ENSG00000171444 | MCC | blue |
| ENSG00000171453 | POLR1C | blue |
| ENSG00000171490 | RSL1D1 | blue |
| ENSG00000171604 | CXXC5 | blue |
| ENSG00000171606 | ZNF274 | blue |
| ENSG00000171612 | SLC25A33 | blue |
| ENSG00000171621 | SPSB1 | blue |
| ENSG00000171649 | ZIK1 | blue |
| ENSG00000171659 | GPR34 | blue |
| ENSG00000171681 | ATF7IP | blue |
| ENSG00000171700 | RGS19 | blue |
| ENSG00000171720 | HDAC3 | blue |

| | | |
|---|---|---|
| ENSG00000171735 | CAMTA1 | blue |
| ENSG00000171777 | RASGRP4 | blue |
| ENSG00000171811 | CFAP46 | blue |
| ENSG00000171812 | COL8A2 | blue |
| ENSG00000171823 | FBXL14 | blue |
| ENSG00000171858 | RPS21 | blue |
| ENSG00000171954 | CYP4F22 | blue |
| ENSG00000171960 | PPIH | blue |
| ENSG00000171984 | C20orf196 | blue |
| ENSG00000172046 | USP19 | blue |
| ENSG00000172053 | QARS | blue |
| ENSG00000172057 | ORMDL3 | blue |
| ENSG00000172081 | MOB3A | blue |
| ENSG00000172171 | TEFM | blue |
| ENSG00000172244 | C5orf34 | blue |
| ENSG00000172269 | DPAGT1 | blue |
| ENSG00000172270 | BSG | blue |
| ENSG00000172301 | COPRS | blue |
| ENSG00000172336 | POP7 | blue |
| ENSG00000172354 | GNB2 | blue |
| ENSG00000172409 | CLP1 | blue |
| ENSG00000172428 | COPS9 | blue |
| ENSG00000172500 | FIBP | blue |
| ENSG00000172531 | PPP1CA | blue |
| ENSG00000172572 | PDE3A | blue |
| ENSG00000172586 | CHCHD1 | blue |
| ENSG00000172590 | MRPL52 | blue |
| ENSG00000172640 | OR10AD1 | blue |
| ENSG00000172650 | AGAP5 | blue |
| ENSG00000172809 | RPL38 | blue |
| ENSG00000172828 | CES3 | blue |
| ENSG00000172840 | PDP2 | blue |
| ENSG00000172922 | RNASEH2C | blue |
| ENSG00000172943 | PHF8 | blue |
| ENSG00000172965 | MIR4435-2HG | blue |
| ENSG00000172974 | AC007318.1 | blue |
| ENSG00000172992 | DCAKD | blue |
| ENSG00000173064 | HECTD4 | blue |
| ENSG00000173065 | FAM222B | blue |
| ENSG00000173083 | HPSE | blue |
| ENSG00000173113 | TRMT112 | blue |
| ENSG00000173141 | MRPL57 | blue |
| ENSG00000173163 | COMMD1 | blue |
| ENSG00000173166 | RAPH1 | blue |
| ENSG00000173209 | AHSA2 | blue |
| ENSG00000173262 | SLC2A14 | blue |
| ENSG00000173272 | MZT2A | blue |
| ENSG00000173275 | ZNF449 | blue |

| | | |
|---|---|---|
| ENSG00000173372 | C1QA | blue |
| ENSG00000173402 | DAG1 | blue |
| ENSG00000173442 | EHBP1L1 | blue |
| ENSG00000173457 | PPP1R14B | blue |
| ENSG00000173465 | SSSCA1 | blue |
| ENSG00000173486 | FKBP2 | blue |
| ENSG00000173511 | VEGFB | blue |
| ENSG00000173535 | TNFRSF10C | blue |
| ENSG00000173559 | NABP1 | blue |
| ENSG00000173581 | CCDC106 | blue |
| ENSG00000173588 | CEP83 | blue |
| ENSG00000173611 | SCAI | blue |
| ENSG00000173653 | RCE1 | blue |
| ENSG00000173660 | UQCRH | blue |
| ENSG00000173681 | BCLAF3 | blue |
| ENSG00000173692 | PSMD1 | blue |
| ENSG00000173762 | CD7 | blue |
| ENSG00000173801 | JUP | blue |
| ENSG00000173812 | EIF1 | blue |
| ENSG00000173889 | PHC3 | blue |
| ENSG00000173928 | SWSAP1 | blue |
| ENSG00000173933 | RBM4 | blue |
| ENSG00000173988 | LRRC63 | blue |
| ENSG00000173992 | CCS | blue |
| ENSG00000174004 | NRROS | blue |
| ENSG00000174021 | GNG5 | blue |
| ENSG00000174028 | FAM3C2 | blue |
| ENSG00000174109 | C16orf91 | blue |
| ENSG00000174130 | TLR6 | blue |
| ENSG00000174136 | RGMB | blue |
| ENSG00000174137 | FAM53A | blue |
| ENSG00000174177 | CTU2 | blue |
| ENSG00000174227 | PIGG | blue |
| ENSG00000174238 | PITPNA | blue |
| ENSG00000174243 | DDX23 | blue |
| ENSG00000174255 | ZNF80 | blue |
| ENSG00000174276 | ZNHIT2 | blue |
| ENSG00000174307 | PHLDA3 | blue |
| ENSG00000174371 | EXO1 | blue |
| ENSG00000174373 | RALGAPA1 | blue |
| ENSG00000174444 | RPL4 | blue |
| ENSG00000174529 | TMEM81 | blue |
| ENSG00000174547 | MRPL11 | blue |
| ENSG00000174600 | CMKLR1 | blue |
| ENSG00000174705 | SH3PXD2B | blue |
| ENSG00000174748 | RPL15 | blue |
| ENSG00000174791 | RIN1 | blue |
| ENSG00000174796 | THAP6 | blue |

| | | |
|---|---|---|
| ENSG00000174844 | DNAH12 | blue |
| ENSG00000174851 | YIF1A | blue |
| ENSG00000174917 | C19orf70 | blue |
| ENSG00000175003 | SLC22A1 | blue |
| ENSG00000175040 | CHST2 | blue |
| ENSG00000175066 | GK5 | blue |
| ENSG00000175130 | MARCKSL1 | blue |
| ENSG00000175137 | SH3BP5L | blue |
| ENSG00000175166 | PSMD2 | blue |
| ENSG00000175193 | PARL | blue |
| ENSG00000175198 | PCCA | blue |
| ENSG00000175220 | ARHGAP1 | blue |
| ENSG00000175283 | DOLK | blue |
| ENSG00000175294 | CATSPER1 | blue |
| ENSG00000175305 | CCNE2 | blue |
| ENSG00000175334 | BANF1 | blue |
| ENSG00000175352 | NRIP3 | blue |
| ENSG00000175376 | EIF1AD | blue |
| ENSG00000175390 | EIF3F | blue |
| ENSG00000175414 | ARL10 | blue |
| ENSG00000175416 | CLTB | blue |
| ENSG00000175445 | LPL | blue |
| ENSG00000175467 | SART1 | blue |
| ENSG00000175489 | LRRC25 | blue |
| ENSG00000175505 | CLCF1 | blue |
| ENSG00000175556 | LONRF3 | blue |
| ENSG00000175567 | UCP2 | blue |
| ENSG00000175575 | PAAF1 | blue |
| ENSG00000175592 | FOSL1 | blue |
| ENSG00000175611 | LINC00476 | blue |
| ENSG00000175701 | LINC00116 | blue |
| ENSG00000175727 | MLXIP | blue |
| ENSG00000175756 | AURKAIP1 | blue |
| ENSG00000175792 | RUVBL1 | blue |
| ENSG00000175806 | MSRA | blue |
| ENSG00000175826 | CTDNEP1 | blue |
| ENSG00000175854 | SWI5 | blue |
| ENSG00000175886 | RPL7AP66 | blue |
| ENSG00000175984 | DENND2C | blue |
| ENSG00000176014 | TUBB6 | blue |
| ENSG00000176058 | TPRN | blue |
| ENSG00000176076 | KCNE5 | blue |
| ENSG00000176087 | SLC35A4 | blue |
| ENSG00000176095 | IP6K1 | blue |
| ENSG00000176101 | SSNA1 | blue |
| ENSG00000176340 | COX8A | blue |
| ENSG00000176393 | RNPEP | blue |
| ENSG00000176401 | EID2B | blue |

| | | |
|---|---|---|
| ENSG00000176438 | SYNE3 | blue |
| ENSG00000176463 | SLCO3A1 | blue |
| ENSG00000176472 | ZNF575 | blue |
| ENSG00000176473 | WDR25 | blue |
| ENSG00000176476 | SGF29 | blue |
| ENSG00000176731 | C8orf59 | blue |
| ENSG00000176946 | THAP4 | blue |
| ENSG00000176973 | FAM89B | blue |
| ENSG00000176974 | SHMT1 | blue |
| ENSG00000176978 | DPP7 | blue |
| ENSG00000176994 | SMCR8 | blue |
| ENSG00000177030 | DEAF1 | blue |
| ENSG00000177103 | DSCAML1 | blue |
| ENSG00000177105 | RHOG | blue |
| ENSG00000177144 | NUDT4P1 | blue |
| ENSG00000177156 | TALDO1 | blue |
| ENSG00000177173 | NAP1L4P1 | blue |
| ENSG00000177192 | PUS1 | blue |
| ENSG00000177301 | KCNA2 | blue |
| ENSG00000177374 | HIC1 | blue |
| ENSG00000177455 | CD19 | blue |
| ENSG00000177463 | NR2C2 | blue |
| ENSG00000177469 | CAVIN1 | blue |
| ENSG00000177483 | RBM44 | blue |
| ENSG00000177542 | SLC25A22 | blue |
| ENSG00000177576 | C18orf32 | blue |
| ENSG00000177600 | RPLP2 | blue |
| ENSG00000177646 | ACAD9 | blue |
| ENSG00000177669 | MBOAT4 | blue |
| ENSG00000177697 | CD151 | blue |
| ENSG00000177700 | POLR2L | blue |
| ENSG00000177706 | FAM20C | blue |
| ENSG00000177721 | ANXA2R | blue |
| ENSG00000177731 | FLII | blue |
| ENSG00000177757 | FAM87B | blue |
| ENSG00000177830 | CHID1 | blue |
| ENSG00000177854 | TMEM187 | blue |
| ENSG00000177868 | SVBP | blue |
| ENSG00000177873 | ZNF619 | blue |
| ENSG00000177971 | IMP3 | blue |
| ENSG00000177990 | DPY19L2 | blue |
| ENSG00000178035 | IMPDH2 | blue |
| ENSG00000178057 | NDUFAF3 | blue |
| ENSG00000178075 | GRAMD1C | blue |
| ENSG00000178078 | STAP2 | blue |
| ENSG00000178096 | BOLA1 | blue |
| ENSG00000178104 | PDE4DIP | blue |
| ENSG00000178217 | SH2D4B | blue |

| | | |
|---|---|---|
| ENSG00000178295 | GEN1 | blue |
| ENSG00000178297 | TMPRSS9 | blue |
| ENSG00000178338 | ZNF354B | blue |
| ENSG00000178385 | PLEKHM3 | blue |
| ENSG00000178449 | COX14 | blue |
| ENSG00000178464 | RPL10P16 | blue |
| ENSG00000178567 | EPM2AIP1 | blue |
| ENSG00000178605 | GTPBP6 | blue |
| ENSG00000178636 | AC092656.1 | blue |
| ENSG00000178660 | ARMC10P1 | blue |
| ENSG00000178741 | COX5A | blue |
| ENSG00000178764 | ZHX2 | blue |
| ENSG00000178802 | MPI | blue |
| ENSG00000178814 | OPLAH | blue |
| ENSG00000178852 | EFCAB13 | blue |
| ENSG00000178896 | EXOSC4 | blue |
| ENSG00000178904 | DPY19L3 | blue |
| ENSG00000178927 | C17orf62 | blue |
| ENSG00000178952 | TUFM | blue |
| ENSG00000178977 | LINC00324 | blue |
| ENSG00000178980 | SELENOW | blue |
| ENSG00000178982 | EIF3K | blue |
| ENSG00000178999 | AURKB | blue |
| ENSG00000179085 | DPM3 | blue |
| ENSG00000179091 | CYC1 | blue |
| ENSG00000179115 | FARSA | blue |
| ENSG00000179163 | FUCA1 | blue |
| ENSG00000179218 | CALR | blue |
| ENSG00000179240 | AP002360.1 | blue |
| ENSG00000179253 | AL162457.1 | blue |
| ENSG00000179262 | RAD23A | blue |
| ENSG00000179271 | GADD45GIP1 | blue |
| ENSG00000179314 | WSCD1 | blue |
| ENSG00000179397 | CATSPERE | blue |
| ENSG00000179526 | SHARPIN | blue |
| ENSG00000179562 | GCC1 | blue |
| ENSG00000179604 | CDC42EP4 | blue |
| ENSG00000179632 | MAF1 | blue |
| ENSG00000179750 | APOBEC3B | blue |
| ENSG00000179862 | CITED4 | blue |
| ENSG00000179912 | R3HDM2 | blue |
| ENSG00000179921 | GPBAR1 | blue |
| ENSG00000179935 | LINC00652 | blue |
| ENSG00000179950 | PUF60 | blue |
| ENSG00000179958 | DCTPP1 | blue |
| ENSG00000179967 | PPP1R14BP3 | blue |
| ENSG00000180061 | TMEM150B | blue |
| ENSG00000180089 | TMEM86B | blue |

| | | |
|---|---|---|
| ENSG00000180096 | 1-Sep | blue |
| ENSG00000180098 | TRNAU1AP | blue |
| ENSG00000180138 | CSNK1A1L | blue |
| ENSG00000180279 | LINC01869 | blue |
| ENSG00000180304 | OAZ2 | blue |
| ENSG00000180316 | PNPLA1 | blue |
| ENSG00000180336 | MEIOC | blue |
| ENSG00000180340 | FZD2 | blue |
| ENSG00000180353 | HCLS1 | blue |
| ENSG00000180481 | GLIPR1L2 | blue |
| ENSG00000180539 | C9orf139 | blue |
| ENSG00000180787 | ZFP3 | blue |
| ENSG00000180822 | PSMG4 | blue |
| ENSG00000180879 | SSR4 | blue |
| ENSG00000180891 | CUEDC1 | blue |
| ENSG00000180992 | MRPL14 | blue |
| ENSG00000181016 | LSMEM1 | blue |
| ENSG00000181019 | NQO1 | blue |
| ENSG00000181031 | RPH3AL | blue |
| ENSG00000181074 | OR52N4 | blue |
| ENSG00000181222 | POLR2A | blue |
| ENSG00000181404 | WASHC1 | blue |
| ENSG00000181444 | ZNF467 | blue |
| ENSG00000181450 | ZNF678 | blue |
| ENSG00000181524 | RPL24P4 | blue |
| ENSG00000181544 | FANCB | blue |
| ENSG00000181588 | MEX3D | blue |
| ENSG00000181619 | GPR135 | blue |
| ENSG00000181754 | AMIGO1 | blue |
| ENSG00000181789 | COPG1 | blue |
| ENSG00000181852 | RNF41 | blue |
| ENSG00000181885 | CLDN7 | blue |
| ENSG00000181924 | COA4 | blue |
| ENSG00000181982 | CCDC149 | blue |
| ENSG00000182154 | MRPL41 | blue |
| ENSG00000182165 | TP53TG1 | blue |
| ENSG00000182173 | TSEN54 | blue |
| ENSG00000182180 | MRPS16 | blue |
| ENSG00000182195 | LDOC1 | blue |
| ENSG00000182199 | SHMT2 | blue |
| ENSG00000182389 | CACNB4 | blue |
| ENSG00000182397 | DNM1P46 | blue |
| ENSG00000182405 | PGBD4 | blue |
| ENSG00000182463 | TSHZ2 | blue |
| ENSG00000182481 | KPNA2 | blue |
| ENSG00000182487 | NCF1B | blue |
| ENSG00000182489 | XKRX | blue |
| ENSG00000182511 | FES | blue |

| | | |
|---|---|---|
| ENSG00000182518 | FAM104B | blue |
| ENSG00000182544 | MFSD5 | blue |
| ENSG00000182584 | ACTL10 | blue |
| ENSG00000182636 | NDN | blue |
| ENSG00000182670 | TTC3 | blue |
| ENSG00000182718 | ANXA2 | blue |
| ENSG00000182742 | HOXB4 | blue |
| ENSG00000182749 | PAQR7 | blue |
| ENSG00000182768 | NGRN | blue |
| ENSG00000182809 | CRIP2 | blue |
| ENSG00000182810 | DDX28 | blue |
| ENSG00000182853 | VMO1 | blue |
| ENSG00000182885 | ADGRG3 | blue |
| ENSG00000182890 | GLUD2 | blue |
| ENSG00000182899 | RPL35A | blue |
| ENSG00000182919 | C11orf54 | blue |
| ENSG00000182934 | SRPRA | blue |
| ENSG00000182973 | CNOT10 | blue |
| ENSG00000182983 | ZNF662 | blue |
| ENSG00000183011 | NAA38 | blue |
| ENSG00000183019 | MCEMP1 | blue |
| ENSG00000183044 | ABAT | blue |
| ENSG00000183048 | SLC25A10 | blue |
| ENSG00000183066 | WBP2NL | blue |
| ENSG00000183207 | RUVBL2 | blue |
| ENSG00000183246 | RIMBP3C | blue |
| ENSG00000183258 | DDX41 | blue |
| ENSG00000183298 | RPSAP19 | blue |
| ENSG00000183354 | KIAA2026 | blue |
| ENSG00000183431 | SF3A3 | blue |
| ENSG00000183444 | OR7E38P | blue |
| ENSG00000183458 | AC138932.1 | blue |
| ENSG00000183496 | MEX3B | blue |
| ENSG00000183530 | PRR14L | blue |
| ENSG00000183604 | SMG1P5 | blue |
| ENSG00000183617 | MRPL54 | blue |
| ENSG00000183665 | TRMT12 | blue |
| ENSG00000183684 | ALYREF | blue |
| ENSG00000183690 | EFHC2 | blue |
| ENSG00000183723 | CMTM4 | blue |
| ENSG00000183734 | ASCL2 | blue |
| ENSG00000183751 | TBL3 | blue |
| ENSG00000183779 | ZNF703 | blue |
| ENSG00000183793 | NPIPA5 | blue |
| ENSG00000183828 | NUDT14 | blue |
| ENSG00000183856 | IQGAP3 | blue |
| ENSG00000183977 | PP2D1 | blue |
| ENSG00000183978 | COA3 | blue |

| | | |
|---|---|---|
| ENSG00000184009 | ACTG1 | blue |
| ENSG00000184056 | VPS33B | blue |
| ENSG00000184060 | ADAP2 | blue |
| ENSG00000184076 | UQCR10 | blue |
| ENSG00000184083 | FAM120C | blue |
| ENSG00000184110 | EIF3C | blue |
| ENSG00000184156 | KCNQ3 | blue |
| ENSG00000184162 | NR2C2AP | blue |
| ENSG00000184164 | CRELD2 | blue |
| ENSG00000184194 | GPR173 | blue |
| ENSG00000184209 | SNRNP35 | blue |
| ENSG00000184216 | IRAK1 | blue |
| ENSG00000184227 | ACOT1 | blue |
| ENSG00000184281 | TSSC4 | blue |
| ENSG00000184293 | CLECL1 | blue |
| ENSG00000184402 | SS18L1 | blue |
| ENSG00000184428 | TOP1MT | blue |
| ENSG00000184432 | COPB2 | blue |
| ENSG00000184436 | THAP7 | blue |
| ENSG00000184465 | WDR27 | blue |
| ENSG00000184508 | HDDC3 | blue |
| ENSG00000184515 | BEX5 | blue |
| ENSG00000184574 | LPAR5 | blue |
| ENSG00000184584 | TMEM173 | blue |
| ENSG00000184613 | NELL2 | blue |
| ENSG00000184675 | AMER1 | blue |
| ENSG00000184730 | APOBR | blue |
| ENSG00000184787 | UBE2G2 | blue |
| ENSG00000184831 | APOO | blue |
| ENSG00000184840 | TMED9 | blue |
| ENSG00000184860 | SDR42E1 | blue |
| ENSG00000184863 | RBM33 | blue |
| ENSG00000184897 | H1FX | blue |
| ENSG00000184900 | SUMO3 | blue |
| ENSG00000184916 | JAG2 | blue |
| ENSG00000184922 | FMNL1 | blue |
| ENSG00000184924 | PTRHD1 | blue |
| ENSG00000184967 | NOC4L | blue |
| ENSG00000184984 | CHRM5 | blue |
| ENSG00000185015 | CA13 | blue |
| ENSG00000185024 | BRF1 | blue |
| ENSG00000185031 | SLC2A3P2 | blue |
| ENSG00000185033 | SEMA4B | blue |
| ENSG00000185043 | CIB1 | blue |
| ENSG00000185049 | NELFA | blue |
| ENSG00000185065 | AC000068.1 | blue |
| ENSG00000185085 | INTS5 | blue |
| ENSG00000185104 | FAF1 | blue |

| | | |
|---|---|---|
| ENSG00000185158 | LRRC37B | blue |
| ENSG00000185187 | SIGIRR | blue |
| ENSG00000185198 | PRSS57 | blue |
| ENSG00000185201 | IFITM2 | blue |
| ENSG00000185222 | TCEAL9 | blue |
| ENSG00000185261 | KIAA0825 | blue |
| ENSG00000185262 | UBALD2 | blue |
| ENSG00000185278 | ZBTB37 | blue |
| ENSG00000185298 | CCDC137 | blue |
| ENSG00000185339 | TCN2 | blue |
| ENSG00000185340 | GAS2L1 | blue |
| ENSG00000185345 | PRKN | blue |
| ENSG00000185420 | SMYD3 | blue |
| ENSG00000185442 | FAM174B | blue |
| ENSG00000185453 | C19orf68 | blue |
| ENSG00000185475 | TMEM179B | blue |
| ENSG00000185480 | PARPBP | blue |
| ENSG00000185527 | PDE6G | blue |
| ENSG00000185585 | OLFML2A | blue |
| ENSG00000185619 | PCGF3 | blue |
| ENSG00000185624 | P4HB | blue |
| ENSG00000185627 | PSMD13 | blue |
| ENSG00000185641 | AC034236.1 | blue |
| ENSG00000185651 | UBE2L3 | blue |
| ENSG00000185716 | C16orf52 | blue |
| ENSG00000185721 | DRG1 | blue |
| ENSG00000185803 | SLC52A2 | blue |
| ENSG00000185808 | PIGP | blue |
| ENSG00000185829 | ARL17A | blue |
| ENSG00000185834 | RPL12P4 | blue |
| ENSG00000185838 | GNB1L | blue |
| ENSG00000185896 | LAMP1 | blue |
| ENSG00000185963 | BICD2 | blue |
| ENSG00000185986 | SDHAP3 | blue |
| ENSG00000186020 | ZNF529 | blue |
| ENSG00000186047 | DLEU7 | blue |
| ENSG00000186074 | CD300LF | blue |
| ENSG00000186076 | AC012085.1 | blue |
| ENSG00000186141 | POLR3C | blue |
| ENSG00000186153 | WWOX | blue |
| ENSG00000186184 | POLR1D | blue |
| ENSG00000186281 | GPAT2 | blue |
| ENSG00000186283 | TOR3A | blue |
| ENSG00000186310 | NAP1L3 | blue |
| ENSG00000186350 | RXRA | blue |
| ENSG00000186364 | NUDT17 | blue |
| ENSG00000186395 | KRT10 | blue |
| ENSG00000186470 | BTN3A2 | blue |

| | | |
|---|---|---|
| ENSG00000186517 | ARHGAP30 | blue |
| ENSG00000186577 | SMIM29 | blue |
| ENSG00000186635 | ARAP1 | blue |
| ENSG00000186638 | KIF24 | blue |
| ENSG00000186792 | HYAL3 | blue |
| ENSG00000186810 | CXCR3 | blue |
| ENSG00000186812 | ZNF397 | blue |
| ENSG00000186814 | ZSCAN30 | blue |
| ENSG00000186827 | TNFRSF4 | blue |
| ENSG00000186871 | ERCC6L | blue |
| ENSG00000186891 | TNFRSF18 | blue |
| ENSG00000186998 | EMID1 | blue |
| ENSG00000187017 | ESPN | blue |
| ENSG00000187051 | RPS19BP1 | blue |
| ENSG00000187147 | RNF220 | blue |
| ENSG00000187187 | ZNF546 | blue |
| ENSG00000187193 | MT1X | blue |
| ENSG00000187240 | DYNC2H1 | blue |
| ENSG00000187498 | COL4A1 | blue |
| ENSG00000187531 | SIRT7 | blue |
| ENSG00000187566 | NHLRC1 | blue |
| ENSG00000187621 | TCL6 | blue |
| ENSG00000187630 | DHRS4L2 | blue |
| ENSG00000187688 | TRPV2 | blue |
| ENSG00000187713 | TMEM203 | blue |
| ENSG00000187778 | MCRS1 | blue |
| ENSG00000187801 | ZFP69B | blue |
| ENSG00000187815 | ZFP69 | blue |
| ENSG00000187840 | EIF4EBP1 | blue |
| ENSG00000187862 | TTC24 | blue |
| ENSG00000187950 | OVCH1 | blue |
| ENSG00000187953 | PMS2CL | blue |
| ENSG00000188051 | TMEM221 | blue |
| ENSG00000188070 | C11orf95 | blue |
| ENSG00000188107 | EYS | blue |
| ENSG00000188186 | LAMTOR4 | blue |
| ENSG00000188219 | POTEE | blue |
| ENSG00000188234 | AGAP4 | blue |
| ENSG00000188242 | PP7080 | blue |
| ENSG00000188305 | C19orf35 | blue |
| ENSG00000188459 | WASF4P | blue |
| ENSG00000188460 | ACTBP11 | blue |
| ENSG00000188486 | H2AFX | blue |
| ENSG00000188511 | C22orf34 | blue |
| ENSG00000188687 | SLC4A5 | blue |
| ENSG00000188690 | UROS | blue |
| ENSG00000188760 | TMEM198 | blue |
| ENSG00000188779 | SKOR1 | blue |

| | | |
|---|---|---|
| ENSG00000188818 | ZDHHC11 | blue |
| ENSG00000188846 | RPL14 | blue |
| ENSG00000188895 | MSL1 | blue |
| ENSG00000188976 | NOC2L | blue |
| ENSG00000188985 | DHFRP1 | blue |
| ENSG00000188986 | NELFB | blue |
| ENSG00000188997 | KCTD21 | blue |
| ENSG00000189127 | ANKRD34B | blue |
| ENSG00000189159 | JPT1 | blue |
| ENSG00000189180 | ZNF33A | blue |
| ENSG00000189238 | LINC00943 | blue |
| ENSG00000189306 | RRP7A | blue |
| ENSG00000189308 | LIN54 | blue |
| ENSG00000189343 | AC004448.1 | blue |
| ENSG00000189362 | NEMP2 | blue |
| ENSG00000189420 | ZFP92 | blue |
| ENSG00000189423 | USP32P3 | blue |
| ENSG00000196154 | S100A4 | blue |
| ENSG00000196155 | PLEKHG4 | blue |
| ENSG00000196182 | STK40 | blue |
| ENSG00000196189 | SEMA4A | blue |
| ENSG00000196199 | MPHOSPH8 | blue |
| ENSG00000196209 | SIRPB2 | blue |
| ENSG00000196218 | RYR1 | blue |
| ENSG00000196233 | LCOR | blue |
| ENSG00000196262 | PPIA | blue |
| ENSG00000196268 | ZNF493 | blue |
| ENSG00000196275 | GTF2IRD2 | blue |
| ENSG00000196313 | POM121 | blue |
| ENSG00000196335 | STK31 | blue |
| ENSG00000196365 | LONP1 | blue |
| ENSG00000196372 | ASB13 | blue |
| ENSG00000196391 | ZNF774 | blue |
| ENSG00000196396 | PTPN1 | blue |
| ENSG00000196407 | THEM5 | blue |
| ENSG00000196417 | ZNF765 | blue |
| ENSG00000196419 | XRCC6 | blue |
| ENSG00000196433 | ASMT | blue |
| ENSG00000196436 | NPIPB15 | blue |
| ENSG00000196453 | ZNF777 | blue |
| ENSG00000196456 | ZNF775 | blue |
| ENSG00000196460 | RFX8 | blue |
| ENSG00000196505 | GDAP2 | blue |
| ENSG00000196510 | ANAPC7 | blue |
| ENSG00000196531 | NACA | blue |
| ENSG00000196544 | BORCS6 | blue |
| ENSG00000196547 | MAN2A2 | blue |
| ENSG00000196576 | PLXNB2 | blue |

| | | |
|---|---|---|
| ENSG00000196588 | MKL1 | blue |
| ENSG00000196642 | RABL6 | blue |
| ENSG00000196659 | TTC30B | blue |
| ENSG00000196668 | LINC00173 | blue |
| ENSG00000196705 | ZNF431 | blue |
| ENSG00000196724 | ZNF418 | blue |
| ENSG00000196741 | LINC01560 | blue |
| ENSG00000196757 | ZNF700 | blue |
| ENSG00000196821 | C6orf106 | blue |
| ENSG00000196867 | ZFP28 | blue |
| ENSG00000196876 | SCN8A | blue |
| ENSG00000196912 | ANKRD36B | blue |
| ENSG00000196933 | RPS26P11 | blue |
| ENSG00000196951 | SCOC-AS1 | blue |
| ENSG00000196967 | ZNF585A | blue |
| ENSG00000196976 | LAGE3 | blue |
| ENSG00000197008 | ZNF138 | blue |
| ENSG00000197013 | ZNF429 | blue |
| ENSG00000197016 | ZNF470 | blue |
| ENSG00000197019 | SERTAD1 | blue |
| ENSG00000197020 | ZNF100 | blue |
| ENSG00000197021 | CXorf40B | blue |
| ENSG00000197043 | ANXA6 | blue |
| ENSG00000197046 | SIGLEC15 | blue |
| ENSG00000197111 | PCBP2 | blue |
| ENSG00000197121 | PGAP1 | blue |
| ENSG00000197122 | SRC | blue |
| ENSG00000197157 | SND1 | blue |
| ENSG00000197183 | NOL4L | blue |
| ENSG00000197217 | ENTPD4 | blue |
| ENSG00000197249 | SERPINA1 | blue |
| ENSG00000197251 | LINC00336 | blue |
| ENSG00000197258 | EIF4BP6 | blue |
| ENSG00000197272 | IL27 | blue |
| ENSG00000197296 | FITM2 | blue |
| ENSG00000197324 | LRP10 | blue |
| ENSG00000197343 | ZNF655 | blue |
| ENSG00000197345 | MRPL21 | blue |
| ENSG00000197355 | UAP1L1 | blue |
| ENSG00000197362 | ZNF786 | blue |
| ENSG00000197375 | SLC22A5 | blue |
| ENSG00000197385 | ZNF860 | blue |
| ENSG00000197403 | OR6N1 | blue |
| ENSG00000197451 | HNRNPAB | blue |
| ENSG00000197461 | PDGFA | blue |
| ENSG00000197465 | GYPE | blue |
| ENSG00000197467 | COL13A1 | blue |
| ENSG00000197497 | ZNF665 | blue |

| | | |
|---|---|---|
| ENSG00000197535 | MYO5A | blue |
| ENSG00000197540 | GZMM | blue |
| ENSG00000197548 | ATG7 | blue |
| ENSG00000197568 | HHLA3 | blue |
| ENSG00000197647 | ZNF433 | blue |
| ENSG00000197712 | FAM114A1 | blue |
| ENSG00000197746 | PSAP | blue |
| ENSG00000197747 | S100A10 | blue |
| ENSG00000197756 | RPL37A | blue |
| ENSG00000197779 | ZNF81 | blue |
| ENSG00000197782 | ZNF780A | blue |
| ENSG00000197785 | ATAD3A | blue |
| ENSG00000197818 | SLC9A8 | blue |
| ENSG00000197863 | ZNF790 | blue |
| ENSG00000197943 | PLCG2 | blue |
| ENSG00000197956 | S100A6 | blue |
| ENSG00000197958 | RPL12 | blue |
| ENSG00000197961 | ZNF121 | blue |
| ENSG00000197978 | GOLGA6L9 | blue |
| ENSG00000197982 | C1orf122 | blue |
| ENSG00000198003 | CCDC151 | blue |
| ENSG00000198018 | ENTPD7 | blue |
| ENSG00000198034 | RPS4X | blue |
| ENSG00000198046 | ZNF667 | blue |
| ENSG00000198105 | ZNF248 | blue |
| ENSG00000198113 | TOR4A | blue |
| ENSG00000198155 | ZNF876P | blue |
| ENSG00000198171 | DDRGK1 | blue |
| ENSG00000198242 | RPL23A | blue |
| ENSG00000198258 | UBL5 | blue |
| ENSG00000198270 | TMEM116 | blue |
| ENSG00000198298 | ZNF485 | blue |
| ENSG00000198342 | ZNF442 | blue |
| ENSG00000198353 | HOXC4 | blue |
| ENSG00000198356 | ASNA1 | blue |
| ENSG00000198373 | WWP2 | blue |
| ENSG00000198399 | ITSN2 | blue |
| ENSG00000198416 | ZNF658B | blue |
| ENSG00000198417 | MT1F | blue |
| ENSG00000198520 | C1orf228 | blue |
| ENSG00000198546 | ZNF511 | blue |
| ENSG00000198561 | CTNND1 | blue |
| ENSG00000198563 | DDX39B | blue |
| ENSG00000198618 | PPIAP22 | blue |
| ENSG00000198663 | C6orf89 | blue |
| ENSG00000198690 | FAN1 | blue |
| ENSG00000198695 | MT-ND6 | blue |
| ENSG00000198712 | MT-CO2 | blue |

| | | |
|---|---|---|
| ENSG00000198736 | MSRB1 | blue |
| ENSG00000198740 | ZNF652 | blue |
| ENSG00000198744 | MTCO3P12 | blue |
| ENSG00000198746 | GPATCH3 | blue |
| ENSG00000198755 | RPL10A | blue |
| ENSG00000198763 | MT-ND2 | blue |
| ENSG00000198786 | MT-ND5 | blue |
| ENSG00000198804 | MT-CO1 | blue |
| ENSG00000198805 | PNP | blue |
| ENSG00000198816 | ZNF358 | blue |
| ENSG00000198825 | INPP5F | blue |
| ENSG00000198832 | SELENOM | blue |
| ENSG00000198835 | GJC2 | blue |
| ENSG00000198840 | MT-ND3 | blue |
| ENSG00000198841 | KTI12 | blue |
| ENSG00000198886 | MT-ND4 | blue |
| ENSG00000198888 | MT-ND1 | blue |
| ENSG00000198899 | MT-ATP6 | blue |
| ENSG00000198901 | PRC1 | blue |
| ENSG00000198908 | BHLHB9 | blue |
| ENSG00000198911 | SREBF2 | blue |
| ENSG00000198915 | RASGEF1A | blue |
| ENSG00000198917 | SPOUT1 | blue |
| ENSG00000198920 | KIAA0753 | blue |
| ENSG00000198931 | APRT | blue |
| ENSG00000198937 | CCDC167 | blue |
| ENSG00000198938 | MT-CO3 | blue |
| ENSG00000198951 | NAGA | blue |
| ENSG00000198960 | ARMCX6 | blue |
| ENSG00000199082 | MIR342 | blue |
| ENSG00000199477 | SNORA31 | blue |
| ENSG00000199691 | RN7SKP173 | blue |
| ENSG00000199753 | SNORD104 | blue |
| ENSG00000200170 | Y_RNA | blue |
| ENSG00000200204 | RNU1-22P | blue |
| ENSG00000201441 | RNU6-646P | blue |
| ENSG00000203288 | TDRKH-AS1 | blue |
| ENSG00000203363 | AC012454.1 | blue |
| ENSG00000203392 | AC105020.1 | blue |
| ENSG00000203497 | PDCD4-AS1 | blue |
| ENSG00000203734 | ECT2L | blue |
| ENSG00000203760 | CENPW | blue |
| ENSG00000203812 | HIST2H2AA3 | blue |
| ENSG00000203875 | SNHG5 | blue |
| ENSG00000203950 | RTL8A | blue |
| ENSG00000204020 | LIPN | blue |
| ENSG00000204070 | SYS1 | blue |
| ENSG00000204084 | INPP5B | blue |

| | | |
|---|---|---|
| ENSG00000204149 | AGAP6 | blue |
| ENSG00000204160 | ZDHHC18 | blue |
| ENSG00000204172 | AGAP9 | blue |
| ENSG00000204231 | RXRB | blue |
| ENSG00000204237 | OXLD1 | blue |
| ENSG00000204272 | NBDY | blue |
| ENSG00000204287 | HLA-DRA | blue |
| ENSG00000204305 | AGER | blue |
| ENSG00000204311 | DFNB59 | blue |
| ENSG00000204323 | SMIM5 | blue |
| ENSG00000204352 | C9orf129 | blue |
| ENSG00000204469 | PRRC2A | blue |
| ENSG00000204592 | HLA-E | blue |
| ENSG00000204628 | RACK1 | blue |
| ENSG00000204673 | AKT1S1 | blue |
| ENSG00000204842 | ATXN2 | blue |
| ENSG00000204856 | FAM216A | blue |
| ENSG00000204933 | CD177P1 | blue |
| ENSG00000204936 | CD177 | blue |
| ENSG00000204946 | ZNF783 | blue |
| ENSG00000204977 | TRIM13 | blue |
| ENSG00000205045 | SLFN12L | blue |
| ENSG00000205090 | TMEM240 | blue |
| ENSG00000205138 | SDHAF1 | blue |
| ENSG00000205220 | PSMB10 | blue |
| ENSG00000205309 | NT5M | blue |
| ENSG00000205352 | PRR13 | blue |
| ENSG00000205464 | ATP6AP1L | blue |
| ENSG00000205609 | EIF3CL | blue |
| ENSG00000205629 | LCMT1 | blue |
| ENSG00000205683 | DPF3 | blue |
| ENSG00000205746 | AC126755.1 | blue |
| ENSG00000205758 | CRYZL1 | blue |
| ENSG00000205809 | KLRC2 | blue |
| ENSG00000205918 | PDPK2P | blue |
| ENSG00000205959 | AC105345.1 | blue |
| ENSG00000206077 | ZDHHC11B | blue |
| ENSG00000206199 | ANKUB1 | blue |
| ENSG00000206337 | HCP5 | blue |
| ENSG00000206384 | COL6A6 | blue |
| ENSG00000206530 | CFAP44 | blue |
| ENSG00000206535 | LNP1 | blue |
| ENSG00000206562 | METTL6 | blue |
| ENSG00000206573 | THUMPD3-AS1 | blue |
| ENSG00000206754 | SNORD101 | blue |
| ENSG00000206965 | RNU6-5P | blue |
| ENSG00000207034 | Y_RNA | blue |
| ENSG00000207523 | SNORA66 | blue |

| | | |
|---|---|---|
| ENSG00000207547 | MIR25 | blue |
| ENSG00000207721 | MIR186 | blue |
| ENSG00000209482 | SNORD83A | blue |
| ENSG00000210140 | MT-TC | blue |
| ENSG00000210196 | MT-TP | blue |
| ENSG00000211450 | SELENOH | blue |
| ENSG00000211459 | MT-RNR1 | blue |
| ENSG00000211595 | IGKJ3 | blue |
| ENSG00000211658 | IGLV3-27 | blue |
| ENSG00000211689 | TRGC1 | blue |
| ENSG00000211696 | TRGV8 | blue |
| ENSG00000211697 | TRGV5 | blue |
| ENSG00000211698 | TRGV4 | blue |
| ENSG00000211727 | TRBV7-6 | blue |
| ENSG00000211767 | TRBJ2-3 | blue |
| ENSG00000211771 | TRBJ2-7 | blue |
| ENSG00000211772 | TRBC2 | blue |
| ENSG00000211787 | TRAV8-3 | blue |
| ENSG00000211799 | TRAV19 | blue |
| ENSG00000211801 | TRAV21 | blue |
| ENSG00000211804 | TRDV1 | blue |
| ENSG00000211809 | TRAV27 | blue |
| ENSG00000211850 | TRAJ39 | blue |
| ENSG00000211871 | TRAJ18 | blue |
| ENSG00000211887 | TRAJ2 | blue |
| ENSG00000211898 | IGHD | blue |
| ENSG00000211899 | IGHM | blue |
| ENSG00000212541 | RNU6-510P | blue |
| ENSG00000212607 | SNORA3B | blue |
| ENSG00000212694 | LINC01089 | blue |
| ENSG00000212789 | ST13P5 | blue |
| ENSG00000212802 | RPL15P3 | blue |
| ENSG00000212864 | RNF208 | blue |
| ENSG00000213057 | C1orf220 | blue |
| ENSG00000213066 | FGFR1OP | blue |
| ENSG00000213073 | AL353625.1 | blue |
| ENSG00000213145 | CRIP1 | blue |
| ENSG00000213225 | NOC2LP1 | blue |
| ENSG00000213228 | RPL12P38 | blue |
| ENSG00000213250 | RBMS2P1 | blue |
| ENSG00000213260 | YWHAZP5 | blue |
| ENSG00000213293 | AC012618.1 | blue |
| ENSG00000213300 | HNRNPA3P6 | blue |
| ENSG00000213339 | QTRT1 | blue |
| ENSG00000213347 | MXD3 | blue |
| ENSG00000213413 | PVRIG | blue |
| ENSG00000213442 | RPL18AP3 | blue |
| ENSG00000213462 | ERV3-1 | blue |

| | | |
|---|---|---|
| ENSG00000213465 | ARL2 | blue |
| ENSG00000213553 | RPLP0P6 | blue |
| ENSG00000213585 | VDAC1 | blue |
| ENSG00000213593 | TMX2 | blue |
| ENSG00000213614 | HEXA | blue |
| ENSG00000213619 | NDUFS3 | blue |
| ENSG00000213693 | SEC14L1P1 | blue |
| ENSG00000213699 | SLC35F6 | blue |
| ENSG00000213741 | RPS29 | blue |
| ENSG00000213763 | ACTBP2 | blue |
| ENSG00000213839 | TMX2P1 | blue |
| ENSG00000213859 | KCTD11 | blue |
| ENSG00000213885 | RPL13AP7 | blue |
| ENSG00000213904 | LIPE-AS1 | blue |
| ENSG00000213939 | AC091153.1 | blue |
| ENSG00000214013 | GANC | blue |
| ENSG00000214106 | PAXIP1-AS2 | blue |
| ENSG00000214113 | LYRM4 | blue |
| ENSG00000214174 | AMZ2P1 | blue |
| ENSG00000214198 | TTC41P | blue |
| ENSG00000214212 | C19orf38 | blue |
| ENSG00000214253 | FIS1 | blue |
| ENSG00000214274 | ANG | blue |
| ENSG00000214413 | BBIP1 | blue |
| ENSG00000214425 | LRRC37A4P | blue |
| ENSG00000214439 | FAM185BP | blue |
| ENSG00000214535 | RPS15AP1 | blue |
| ENSG00000214544 | GTF2IRD2P1 | blue |
| ENSG00000214595 | EML6 | blue |
| ENSG00000214654 | AL161911.1 | blue |
| ENSG00000214659 | KRT8P26 | blue |
| ENSG00000214694 | ARHGEF33 | blue |
| ENSG00000214706 | IFRD2 | blue |
| ENSG00000214717 | ZBED1 | blue |
| ENSG00000214753 | HNRNPUL2 | blue |
| ENSG00000214826 | DDX12P | blue |
| ENSG00000214827 | MTCP1 | blue |
| ENSG00000214940 | NPIPA8 | blue |
| ENSG00000214967 | NPIPA7 | blue |
| ENSG00000215021 | PHB2 | blue |
| ENSG00000215030 | AC073621.1 | blue |
| ENSG00000215105 | TTC3P1 | blue |
| ENSG00000215252 | GOLGA8B | blue |
| ENSG00000215256 | DHRS4-AS1 | blue |
| ENSG00000215267 | AKR1C7P | blue |
| ENSG00000215271 | HOMEZ | blue |
| ENSG00000215302 | AC127502.1 | blue |
| ENSG00000215374 | FAM66B | blue |

| | | |
|---|---|---|
| ENSG00000215548 | FRG1JP | blue |
| ENSG00000215845 | TSTD1 | blue |
| ENSG00000215883 | CYB5RL | blue |
| ENSG00000216866 | RPS2P55 | blue |
| ENSG00000216895 | AC009403.1 | blue |
| ENSG00000216937 | CCDC7 | blue |
| ENSG00000217083 | MTCO2P33 | blue |
| ENSG00000217094 | PPIAP31 | blue |
| ENSG00000217130 | AL139100.1 | blue |
| ENSG00000217258 | AC007249.1 | blue |
| ENSG00000217275 | AL031777.1 | blue |
| ENSG00000217325 | PRELID1P1 | blue |
| ENSG00000217624 | AL627402.1 | blue |
| ENSG00000217643 | PTGES3P2 | blue |
| ENSG00000217716 | RPS10P3 | blue |
| ENSG00000218018 | AL109955.1 | blue |
| ENSG00000218175 | AC016739.1 | blue |
| ENSG00000218227 | AC136632.1 | blue |
| ENSG00000218510 | LINC00339 | blue |
| ENSG00000219023 | AL033519.2 | blue |
| ENSG00000219391 | AC019129.1 | blue |
| ENSG00000219665 | AC008770.1 | blue |
| ENSG00000219682 | AL133268.1 | blue |
| ENSG00000219928 | AL161787.1 | blue |
| ENSG00000220205 | VAMP2 | blue |
| ENSG00000220472 | AL139095.2 | blue |
| ENSG00000221539 | SNORD99 | blue |
| ENSG00000221817 | PPP3CB-AS1 | blue |
| ENSG00000221821 | C6orf226 | blue |
| ENSG00000221838 | AP4M1 | blue |
| ENSG00000221937 | TAS2R40 | blue |
| ENSG00000221944 | TIGD1 | blue |
| ENSG00000221990 | EXOC3-AS1 | blue |
| ENSG00000221994 | ZNF630 | blue |
| ENSG00000222037 | IGLC6 | blue |
| ENSG00000222057 | RNU4-62P | blue |
| ENSG00000222345 | SNORD19 | blue |
| ENSG00000222375 | RN7SKP127 | blue |
| ENSG00000223309 | RNU6-722P | blue |
| ENSG00000223442 | TH2LCRR | blue |
| ENSG00000223459 | TCAF1P1 | blue |
| ENSG00000223482 | NUTM2A-AS1 | blue |
| ENSG00000223549 | MTND5P28 | blue |
| ENSG00000223551 | TMSB4XP4 | blue |
| ENSG00000223583 | AL513365.1 | blue |
| ENSG00000223697 | AF230666.1 | blue |
| ENSG00000223725 | AC009226.1 | blue |
| ENSG00000223749 | MIR503HG | blue |

| | | |
|---|---|---|
| ENSG00000223797 | ENTPD3-AS1 | blue |
| ENSG00000223803 | RPS20P14 | blue |
| ENSG00000223873 | SAP18P2 | blue |
| ENSG00000223877 | RPS8P10 | blue |
| ENSG00000223878 | AC005517.1 | blue |
| ENSG00000223922 | ASS1P2 | blue |
| ENSG00000223935 | AC008074.1 | blue |
| ENSG00000223959 | AFG3L1P | blue |
| ENSG00000223972 | DDX11L1 | blue |
| ENSG00000223991 | AC104809.1 | blue |
| ENSG00000224032 | EPB41L4A-AS1 | blue |
| ENSG00000224067 | AL354877.1 | blue |
| ENSG00000224137 | LINC01857 | blue |
| ENSG00000224138 | AC000123.1 | blue |
| ENSG00000224195 | AC022400.1 | blue |
| ENSG00000224207 | AC018797.1 | blue |
| ENSG00000224334 | AP000357.1 | blue |
| ENSG00000224376 | AC017104.1 | blue |
| ENSG00000224397 | SMIM25 | blue |
| ENSG00000224429 | LINC00539 | blue |
| ENSG00000224437 | PIGUP1 | blue |
| ENSG00000224543 | SNRPGP15 | blue |
| ENSG00000224597 | SVIL-AS1 | blue |
| ENSG00000224769 | MUC20P1 | blue |
| ENSG00000224805 | LINC00853 | blue |
| ENSG00000224831 | AC117395.2 | blue |
| ENSG00000224839 | RPL12P17 | blue |
| ENSG00000224877 | NDUFAF8 | blue |
| ENSG00000225093 | RPL3P7 | blue |
| ENSG00000225177 | AL590617.2 | blue |
| ENSG00000225178 | RPSAP58 | blue |
| ENSG00000225200 | AC246787.1 | blue |
| ENSG00000225205 | AC078883.1 | blue |
| ENSG00000225462 | FDPSP1 | blue |
| ENSG00000225465 | RFPL1S | blue |
| ENSG00000225470 | JPX | blue |
| ENSG00000225527 | AL450384.1 | blue |
| ENSG00000225544 | AC245452.2 | blue |
| ENSG00000225611 | LINC02158 | blue |
| ENSG00000225663 | MCRIP1 | blue |
| ENSG00000225766 | DHRS4L1 | blue |
| ENSG00000225791 | TRAM2-AS1 | blue |
| ENSG00000225792 | AC004540.2 | blue |
| ENSG00000225873 | LINC00694 | blue |
| ENSG00000225880 | LINC00115 | blue |
| ENSG00000225921 | NOL7 | blue |
| ENSG00000225940 | C5orf67 | blue |
| ENSG00000225963 | AC009950.1 | blue |

| | | |
|---|---|---|
| ENSG00000225975 | LINC01534 | blue |
| ENSG00000226057 | PHF2P2 | blue |
| ENSG00000226067 | LINC00623 | blue |
| ENSG00000226121 | AC009487.2 | blue |
| ENSG00000226167 | AP4B1-AS1 | blue |
| ENSG00000226200 | SGMS1-AS1 | blue |
| ENSG00000226243 | RPL37AP1 | blue |
| ENSG00000226281 | AL031123.1 | blue |
| ENSG00000226287 | TMEM191A | blue |
| ENSG00000226306 | NPY6R | blue |
| ENSG00000226310 | AL022157.1 | blue |
| ENSG00000226321 | CROCC2 | blue |
| ENSG00000226352 | PSPC1-AS2 | blue |
| ENSG00000226360 | AC096919.1 | blue |
| ENSG00000226393 | IFNA20P | blue |
| ENSG00000226415 | TPI1P1 | blue |
| ENSG00000226608 | FTLP3 | blue |
| ENSG00000226648 | PLCG1-AS1 | blue |
| ENSG00000226659 | AC021028.1 | blue |
| ENSG00000226677 | IGBP1P1 | blue |
| ENSG00000226688 | ENTPD1-AS1 | blue |
| ENSG00000226742 | HSBP1L1 | blue |
| ENSG00000226752 | PSMD5-AS1 | blue |
| ENSG00000226853 | AC010894.2 | blue |
| ENSG00000226950 | DANCR | blue |
| ENSG00000226986 | AC092017.1 | blue |
| ENSG00000227001 | NBPF2P | blue |
| ENSG00000227051 | C14orf132 | blue |
| ENSG00000227070 | AC104170.1 | blue |
| ENSG00000227097 | RPS28P7 | blue |
| ENSG00000227124 | ZNF717 | blue |
| ENSG00000227253 | AL158834.2 | blue |
| ENSG00000227347 | HNRNPKP2 | blue |
| ENSG00000227492 | AL138921.1 | blue |
| ENSG00000227508 | LINC01624 | blue |
| ENSG00000227533 | SLC2A1-AS1 | blue |
| ENSG00000227603 | AL583839.1 | blue |
| ENSG00000227615 | AP001324.1 | blue |
| ENSG00000227671 | AL390728.4 | blue |
| ENSG00000227678 | AL355581.1 | blue |
| ENSG00000227775 | AL031282.1 | blue |
| ENSG00000227782 | AC002553.1 | blue |
| ENSG00000227825 | SLC9A7P1 | blue |
| ENSG00000227855 | DPY19L2P3 | blue |
| ENSG00000227905 | MED6P1 | blue |
| ENSG00000227908 | FLJ31104 | blue |
| ENSG00000228002 | DHX9P1 | blue |
| ENSG00000228079 | AC012368.2 | blue |

| | | |
|---|---|---|
| ENSG00000228109 | MELTF-AS1 | blue |
| ENSG00000228196 | PTPN2P1 | blue |
| ENSG00000228205 | AC131235.1 | blue |
| ENSG00000228300 | C19orf24 | blue |
| ENSG00000228305 | AC016734.1 | blue |
| ENSG00000228315 | GUSBP11 | blue |
| ENSG00000228352 | AL354989.1 | blue |
| ENSG00000228382 | ITPKB-IT1 | blue |
| ENSG00000228403 | AC035139.1 | blue |
| ENSG00000228427 | AL590764.1 | blue |
| ENSG00000228444 | AL137244.1 | blue |
| ENSG00000228452 | AC098484.1 | blue |
| ENSG00000228463 | AP006222.1 | blue |
| ENSG00000228474 | OST4 | blue |
| ENSG00000228487 | AL450263.1 | blue |
| ENSG00000228506 | AL513550.1 | blue |
| ENSG00000228594 | FNDC10 | blue |
| ENSG00000228686 | AL590723.1 | blue |
| ENSG00000228696 | ARL17B | blue |
| ENSG00000228705 | LINC00659 | blue |
| ENSG00000228716 | DHFR | blue |
| ENSG00000228797 | FAM207BP | blue |
| ENSG00000228834 | AL445189.2 | blue |
| ENSG00000228857 | AC104653.1 | blue |
| ENSG00000228878 | SEPT7-AS1 | blue |
| ENSG00000228887 | EEF1DP1 | blue |
| ENSG00000228906 | AL353804.1 | blue |
| ENSG00000228915 | OR7E128P | blue |
| ENSG00000228929 | RPS13P2 | blue |
| ENSG00000228956 | SATB1-AS1 | blue |
| ENSG00000229018 | PMS2P7 | blue |
| ENSG00000229097 | CALM2P2 | blue |
| ENSG00000229119 | AC026403.1 | blue |
| ENSG00000229127 | AC007038.1 | blue |
| ENSG00000229132 | EIF4A1P10 | blue |
| ENSG00000229152 | ANKRD10-IT1 | blue |
| ENSG00000229314 | ORM1 | blue |
| ENSG00000229368 | AC090587.2 | blue |
| ENSG00000229436 | AC073850.1 | blue |
| ENSG00000229473 | RGS17P1 | blue |
| ENSG00000229598 | PRDX3P1 | blue |
| ENSG00000229638 | RPL4P4 | blue |
| ENSG00000229700 | AL008627.1 | blue |
| ENSG00000229715 | EEF1DP3 | blue |
| ENSG00000229780 | UBE2Q1-AS1 | blue |
| ENSG00000229806 | RPS15P5 | blue |
| ENSG00000229808 | AL391825.1 | blue |
| ENSG00000229809 | ZNF688 | blue |

| | | |
|---|---|---|
| ENSG00000229817 | AL133412.1 | blue |
| ENSG00000229833 | PET100 | blue |
| ENSG00000229870 | RPL21P89 | blue |
| ENSG00000229931 | AL137003.1 | blue |
| ENSG00000229939 | AL589880.1 | blue |
| ENSG00000229979 | AC243428.1 | blue |
| ENSG00000230002 | ALMS1-IT1 | blue |
| ENSG00000230069 | LRRC37A15P | blue |
| ENSG00000230092 | AL669831.4 | blue |
| ENSG00000230124 | ACBD6 | blue |
| ENSG00000230202 | AL450405.1 | blue |
| ENSG00000230207 | RPL4P5 | blue |
| ENSG00000230228 | AL590609.2 | blue |
| ENSG00000230257 | NFE4 | blue |
| ENSG00000230325 | AL359921.1 | blue |
| ENSG00000230373 | GOLGA6L5P | blue |
| ENSG00000230445 | LRRC37A6P | blue |
| ENSG00000230457 | PA2G4P4 | blue |
| ENSG00000230530 | LIMD1-AS1 | blue |
| ENSG00000230539 | AOAH-IT1 | blue |
| ENSG00000230565 | ZNF32-AS2 | blue |
| ENSG00000230592 | RPSAP8 | blue |
| ENSG00000230593 | AC090804.1 | blue |
| ENSG00000230641 | USP12-AS2 | blue |
| ENSG00000230658 | KLHL7-AS1 | blue |
| ENSG00000230724 | LINC01001 | blue |
| ENSG00000230725 | AL035252.2 | blue |
| ENSG00000230736 | AL021937.1 | blue |
| ENSG00000230773 | AC092650.1 | blue |
| ENSG00000230897 | AC013248.2 | blue |
| ENSG00000230910 | AL391807.1 | blue |
| ENSG00000230979 | AC079250.1 | blue |
| ENSG00000230989 | HSBP1 | blue |
| ENSG00000231079 | AC105402.3 | blue |
| ENSG00000231128 | AL137856.1 | blue |
| ENSG00000231245 | C1DP1 | blue |
| ENSG00000231249 | ITPR1-AS1 | blue |
| ENSG00000231312 | AC007388.1 | blue |
| ENSG00000231466 | AL022324.2 | blue |
| ENSG00000231494 | AC104634.1 | blue |
| ENSG00000231609 | AC009501.1 | blue |
| ENSG00000231628 | AL133406.2 | blue |
| ENSG00000231663 | AL355472.1 | blue |
| ENSG00000231721 | LINC-PINT | blue |
| ENSG00000231728 | AC234782.2 | blue |
| ENSG00000231858 | AC067945.3 | blue |
| ENSG00000231889 | TRAF3IP2-AS1 | blue |
| ENSG00000231940 | RPS7P3 | blue |

| | | |
|---|---|---|
| ENSG00000231991 | ANXA2P2 | blue |
| ENSG00000231993 | EP300-AS1 | blue |
| ENSG00000232004 | CAP1P2 | blue |
| ENSG00000232104 | RFX3-AS1 | blue |
| ENSG00000232118 | BACH1-AS1 | blue |
| ENSG00000232160 | RAP2C-AS1 | blue |
| ENSG00000232186 | AL137013.1 | blue |
| ENSG00000232229 | LINC00865 | blue |
| ENSG00000232284 | GNG12-AS1 | blue |
| ENSG00000232388 | SMIM26 | blue |
| ENSG00000232442 | MHENCR | blue |
| ENSG00000232470 | AL136368.1 | blue |
| ENSG00000232504 | ST3GAL5-AS1 | blue |
| ENSG00000232573 | RPL3P4 | blue |
| ENSG00000232630 | PRPS1P2 | blue |
| ENSG00000232671 | AL391069.2 | blue |
| ENSG00000232774 | FLJ22447 | blue |
| ENSG00000232860 | SMG7-AS1 | blue |
| ENSG00000232874 | AC080129.2 | blue |
| ENSG00000232912 | AL096855.1 | blue |
| ENSG00000232931 | LINC00342 | blue |
| ENSG00000232934 | AL157786.1 | blue |
| ENSG00000232956 | SNHG15 | blue |
| ENSG00000232995 | RGS5 | blue |
| ENSG00000233016 | SNHG7 | blue |
| ENSG00000233045 | AC097523.1 | blue |
| ENSG00000233087 | WTH3DI | blue |
| ENSG00000233205 | AC108479.1 | blue |
| ENSG00000233266 | HMGB1P31 | blue |
| ENSG00000233276 | GPX1 | blue |
| ENSG00000233306 | TRGV2 | blue |
| ENSG00000233325 | MIPEPP3 | blue |
| ENSG00000233328 | PFN1P1 | blue |
| ENSG00000233337 | UBE2FP3 | blue |
| ENSG00000233411 | AL359962.1 | blue |
| ENSG00000233426 | EIF3FP3 | blue |
| ENSG00000233461 | AL445524.1 | blue |
| ENSG00000233493 | TMEM238 | blue |
| ENSG00000233527 | ZNF529-AS1 | blue |
| ENSG00000233750 | CICP27 | blue |
| ENSG00000233757 | AC092835.1 | blue |
| ENSG00000233762 | AC007969.1 | blue |
| ENSG00000233806 | LINC01237 | blue |
| ENSG00000233820 | AL589843.2 | blue |
| ENSG00000233836 | AC139769.1 | blue |
| ENSG00000233903 | Z83851.1 | blue |
| ENSG00000233927 | RPS28 | blue |
| ENSG00000233937 | AC008443.1 | blue |

| | | |
|---|---|---|
| ENSG00000233967 | AL359715.2 | blue |
| ENSG00000234043 | NUDT9P1 | blue |
| ENSG00000234171 | RNASEH1-AS1 | blue |
| ENSG00000234268 | AP000936.3 | blue |
| ENSG00000234290 | AC116366.1 | blue |
| ENSG00000234329 | AL604028.2 | blue |
| ENSG00000234335 | RPS4XP11 | blue |
| ENSG00000234383 | CTBP2P8 | blue |
| ENSG00000234389 | AC007278.1 | blue |
| ENSG00000234390 | USP27X-AS1 | blue |
| ENSG00000234420 | ZNF37BP | blue |
| ENSG00000234478 | ACBD3-AS1 | blue |
| ENSG00000234484 | AL032821.1 | blue |
| ENSG00000234506 | LINC01506 | blue |
| ENSG00000234511 | C5orf58 | blue |
| ENSG00000234553 | AC022431.2 | blue |
| ENSG00000234585 | CCT6P3 | blue |
| ENSG00000234614 | AL450992.2 | blue |
| ENSG00000234629 | WDR82P1 | blue |
| ENSG00000234719 | NPIPB2 | blue |
| ENSG00000234743 | EIF5AP4 | blue |
| ENSG00000234769 | WASH4P | blue |
| ENSG00000234773 | AC012618.3 | blue |
| ENSG00000234810 | AL603840.1 | blue |
| ENSG00000234825 | XRCC6P2 | blue |
| ENSG00000234882 | EIF3EP1 | blue |
| ENSG00000234883 | MIR155HG | blue |
| ENSG00000234964 | FABP5P7 | blue |
| ENSG00000234996 | AC098934.2 | blue |
| ENSG00000235001 | EIF4A1P2 | blue |
| ENSG00000235043 | TECRP1 | blue |
| ENSG00000235065 | RPL24P2 | blue |
| ENSG00000235078 | AC231981.1 | blue |
| ENSG00000235092 | ID2-AS1 | blue |
| ENSG00000235101 | SETP9 | blue |
| ENSG00000235109 | ZSCAN31 | blue |
| ENSG00000235173 | HGH1 | blue |
| ENSG00000235241 | BX284668.4 | blue |
| ENSG00000235297 | AC008021.1 | blue |
| ENSG00000235298 | AL354733.3 | blue |
| ENSG00000235304 | LINC01281 | blue |
| ENSG00000235453 | SMIM27 | blue |
| ENSG00000235472 | EIF4A1P7 | blue |
| ENSG00000235488 | JARID2-AS1 | blue |
| ENSG00000235508 | RPS2P7 | blue |
| ENSG00000235531 | MSC-AS1 | blue |
| ENSG00000235532 | LINC00402 | blue |
| ENSG00000235568 | NFAM1 | blue |

| | | |
|---|---|---|
| ENSG00000235586 | AC011247.2 | blue |
| ENSG00000235688 | AC116614.1 | blue |
| ENSG00000235698 | PA2G4P2 | blue |
| ENSG00000235724 | AC009299.3 | blue |
| ENSG00000235776 | AC000089.1 | blue |
| ENSG00000235795 | AC093157.2 | blue |
| ENSG00000235831 | BHLHE40-AS1 | blue |
| ENSG00000235919 | ASH1L-AS1 | blue |
| ENSG00000235946 | AC234783.1 | blue |
| ENSG00000235954 | TTC28-AS1 | blue |
| ENSG00000235962 | RPL7AP53 | blue |
| ENSG00000236090 | LDHAP3 | blue |
| ENSG00000236140 | AC245014.1 | blue |
| ENSG00000236320 | SLFN14 | blue |
| ENSG00000236552 | RPL13AP5 | blue |
| ENSG00000236577 | SNRPGP14 | blue |
| ENSG00000236675 | MTX1P1 | blue |
| ENSG00000236739 | CLIC4P1 | blue |
| ENSG00000236756 | DNAJC9-AS1 | blue |
| ENSG00000236778 | INTS6-AS1 | blue |
| ENSG00000236790 | LINC00299 | blue |
| ENSG00000236801 | RPL24P8 | blue |
| ENSG00000236876 | TMSB4XP1 | blue |
| ENSG00000236894 | AL160287.1 | blue |
| ENSG00000236991 | EDRF1-AS1 | blue |
| ENSG00000236992 | RPL12P12 | blue |
| ENSG00000237036 | ZEB1-AS1 | blue |
| ENSG00000237073 | AL162727.2 | blue |
| ENSG00000237181 | AC147651.3 | blue |
| ENSG00000237206 | IMPDH1P4 | blue |
| ENSG00000237214 | AL080243.2 | blue |
| ENSG00000237248 | LINC00987 | blue |
| ENSG00000237298 | TTN-AS1 | blue |
| ENSG00000237484 | LINC01684 | blue |
| ENSG00000237489 | LINC00959 | blue |
| ENSG00000237491 | AL669831.5 | blue |
| ENSG00000237522 | NONOP2 | blue |
| ENSG00000237576 | LINC01888 | blue |
| ENSG00000237651 | C2orf74 | blue |
| ENSG00000237774 | AC126615.1 | blue |
| ENSG00000237877 | LINC01473 | blue |
| ENSG00000237879 | LINC00398 | blue |
| ENSG00000237926 | AC239367.3 | blue |
| ENSG00000237973 | MTCO1P12 | blue |
| ENSG00000237991 | RPL35P1 | blue |
| ENSG00000238018 | AC093110.1 | blue |
| ENSG00000238098 | ABCA17P | blue |
| ENSG00000238142 | BX284668.5 | blue |

| | | |
|---|---|---|
| ENSG00000238160 | AC116366.2 | blue |
| ENSG00000238197 | PAXBP1-AS1 | blue |
| ENSG00000238201 | AC114752.2 | blue |
| ENSG00000238227 | TMEM250 | blue |
| ENSG00000238390 | SNORA81 | blue |
| ENSG00000239149 | SNORA59A | blue |
| ENSG00000239246 | AC008026.1 | blue |
| ENSG00000239332 | LINC01119 | blue |
| ENSG00000239388 | ASB14 | blue |
| ENSG00000239653 | PSMD6-AS2 | blue |
| ENSG00000239672 | NME1 | blue |
| ENSG00000239697 | TNFSF12 | blue |
| ENSG00000239789 | MRPS17 | blue |
| ENSG00000239884 | RN7SL608P | blue |
| ENSG00000239941 | AC108718.1 | blue |
| ENSG00000239998 | LILRA2 | blue |
| ENSG00000240036 | AC104563.1 | blue |
| ENSG00000240057 | AC078785.1 | blue |
| ENSG00000240087 | RPSAP12 | blue |
| ENSG00000240184 | PCDHGC3 | blue |
| ENSG00000240207 | AC080013.1 | blue |
| ENSG00000240225 | ZNF542P | blue |
| ENSG00000240322 | RN7SL481P | blue |
| ENSG00000240376 | AC010343.1 | blue |
| ENSG00000240395 | RPL5P23 | blue |
| ENSG00000240429 | LRRFIP1P1 | blue |
| ENSG00000240527 | AL365273.1 | blue |
| ENSG00000240535 | AC034238.1 | blue |
| ENSG00000240666 | MME-AS1 | blue |
| ENSG00000240682 | ISY1 | blue |
| ENSG00000240751 | AC026348.1 | blue |
| ENSG00000240849 | TMEM189 | blue |
| ENSG00000240890 | AC020633.1 | blue |
| ENSG00000240950 | AC021074.1 | blue |
| ENSG00000241014 | AC114490.1 | blue |
| ENSG00000241015 | TPM3P9 | blue |
| ENSG00000241343 | RPL36A | blue |
| ENSG00000241351 | IGKV3-11 | blue |
| ENSG00000241352 | AC007688.1 | blue |
| ENSG00000241399 | CD302 | blue |
| ENSG00000241431 | RPL37P6 | blue |
| ENSG00000241468 | ATP5J2 | blue |
| ENSG00000241490 | AC093010.2 | blue |
| ENSG00000241549 | GUSBP2 | blue |
| ENSG00000241666 | AL031733.2 | blue |
| ENSG00000241679 | AC018450.1 | blue |
| ENSG00000241685 | ARPC1A | blue |
| ENSG00000241741 | RPL7AP30 | blue |

| | | |
|---|---|---|
| ENSG00000241782 | AP002812.1 | blue |
| ENSG00000241839 | PLEKHO2 | blue |
| ENSG00000241923 | RPL14P3 | blue |
| ENSG00000241975 | ELOCP19 | blue |
| ENSG00000242071 | RPL7AP6 | blue |
| ENSG00000242076 | IGKV1-33 | blue |
| ENSG00000242114 | MTFP1 | blue |
| ENSG00000242125 | SNHG3 | blue |
| ENSG00000242198 | AC008897.1 | blue |
| ENSG00000242252 | BGLAP | blue |
| ENSG00000242282 | AC108488.1 | blue |
| ENSG00000242299 | AC073861.1 | blue |
| ENSG00000242314 | RPL12P32 | blue |
| ENSG00000242324 | AL049634.1 | blue |
| ENSG00000242337 | TFP1 | blue |
| ENSG00000242371 | IGKV1-39 | blue |
| ENSG00000242372 | EIF6 | blue |
| ENSG00000242477 | AC091429.1 | blue |
| ENSG00000242485 | MRPL20 | blue |
| ENSG00000242553 | AP001432.1 | blue |
| ENSG00000242580 | IGKV1D-43 | blue |
| ENSG00000242689 | CNTF | blue |
| ENSG00000242866 | STRC | blue |
| ENSG00000243055 | GK-AS1 | blue |
| ENSG00000243335 | KCTD7 | blue |
| ENSG00000243368 | MCCC1-AS1 | blue |
| ENSG00000243417 | AC023424.2 | blue |
| ENSG00000243449 | C4orf48 | blue |
| ENSG00000243547 | HNRNPKP4 | blue |
| ENSG00000243650 | RN7SL834P | blue |
| ENSG00000243725 | TTC4 | blue |
| ENSG00000243759 | ST13P15 | blue |
| ENSG00000243811 | APOBEC3D | blue |
| ENSG00000243836 | WDR86-AS1 | blue |
| ENSG00000243926 | TIPARP-AS1 | blue |
| ENSG00000243964 | RPL23AP65 | blue |
| ENSG00000243970 | PPIEL | blue |
| ENSG00000244038 | DDOST | blue |
| ENSG00000244041 | LINC01011 | blue |
| ENSG00000244116 | IGKV2-28 | blue |
| ENSG00000244161 | FLNB-AS1 | blue |
| ENSG00000244167 | AC005532.2 | blue |
| ENSG00000244313 | AC024293.1 | blue |
| ENSG00000244405 | ETV5 | blue |
| ENSG00000244509 | APOBEC3C | blue |
| ENSG00000244513 | AC109587.1 | blue |
| ENSG00000244720 | AC055748.1 | blue |
| ENSG00000244754 | N4BP2L2 | blue |

| | | |
|---|---|---|
| ENSG00000245060 | LINC00847 | blue |
| ENSG00000245146 | LINC01024 | blue |
| ENSG00000245498 | AP000866.1 | blue |
| ENSG00000245848 | CEBPA | blue |
| ENSG00000245904 | AC025164.2 | blue |
| ENSG00000245958 | AC093752.1 | blue |
| ENSG00000246082 | NUDT16P1 | blue |
| ENSG00000246090 | AP002026.1 | blue |
| ENSG00000246526 | LINC002481 | blue |
| ENSG00000246627 | CACNA1C-AS1 | blue |
| ENSG00000246889 | AP000487.1 | blue |
| ENSG00000247092 | SNHG10 | blue |
| ENSG00000247271 | ZBED5-AS1 | blue |
| ENSG00000247774 | PCED1B-AS1 | blue |
| ENSG00000247828 | TMEM161B-AS1 | blue |
| ENSG00000247982 | LINC00926 | blue |
| ENSG00000248099 | INSL3 | blue |
| ENSG00000248334 | WHAMMP2 | blue |
| ENSG00000248472 | DDX11L9 | blue |
| ENSG00000248485 | PCP4L1 | blue |
| ENSG00000248487 | ABHD14A | blue |
| ENSG00000248489 | LINC02062 | blue |
| ENSG00000248503 | AL356235.1 | blue |
| ENSG00000248527 | MTATP6P1 | blue |
| ENSG00000248546 | ANP32C | blue |
| ENSG00000248593 | DSTNP2 | blue |
| ENSG00000248626 | GAPDHP40 | blue |
| ENSG00000248697 | TOX4P1 | blue |
| ENSG00000248719 | AC021127.1 | blue |
| ENSG00000248780 | ARL4AP2 | blue |
| ENSG00000248866 | USP46-AS1 | blue |
| ENSG00000248905 | FMN1 | blue |
| ENSG00000249264 | EEF1A1P9 | blue |
| ENSG00000249272 | UNC93B8 | blue |
| ENSG00000249274 | PDLIM1P4 | blue |
| ENSG00000249492 | AC114956.3 | blue |
| ENSG00000249601 | LINC01187 | blue |
| ENSG00000249667 | LINC01259 | blue |
| ENSG00000249806 | AC139720.1 | blue |
| ENSG00000249846 | LINC02021 | blue |
| ENSG00000249855 | EEF1A1P19 | blue |
| ENSG00000249896 | LINC02495 | blue |
| ENSG00000249931 | GOLGA8K | blue |
| ENSG00000249965 | CDC42P4 | blue |
| ENSG00000249978 | TRGV7 | blue |
| ENSG00000249986 | YWHAQP6 | blue |
| ENSG00000249992 | TMEM158 | blue |
| ENSG00000250057 | AC114781.2 | blue |

| | | |
|---|---|---|
| ENSG00000250081 | AC025176.1 | blue |
| ENSG00000250182 | EEF1A1P13 | blue |
| ENSG00000250222 | AC008443.5 | blue |
| ENSG00000250251 | PKD1P6 | blue |
| ENSG00000250312 | ZNF718 | blue |
| ENSG00000250348 | AC113404.1 | blue |
| ENSG00000250461 | AC122718.1 | blue |
| ENSG00000250462 | LRRC37BP1 | blue |
| ENSG00000250474 | WBP1LP2 | blue |
| ENSG00000250490 | LINC02145 | blue |
| ENSG00000250536 | ABHD17AP3 | blue |
| ENSG00000250548 | AL355916.1 | blue |
| ENSG00000250608 | AC010210.1 | blue |
| ENSG00000250687 | AC146944.2 | blue |
| ENSG00000250790 | AC127070.3 | blue |
| ENSG00000250802 | ZBED3-AS1 | blue |
| ENSG00000250889 | LINC01336 | blue |
| ENSG00000250893 | AC098869.2 | blue |
| ENSG00000251000 | AC008592.3 | blue |
| ENSG00000251023 | AC114980.1 | blue |
| ENSG00000251034 | AC037459.2 | blue |
| ENSG00000251093 | AC093281.2 | blue |
| ENSG00000251131 | AC025171.3 | blue |
| ENSG00000251136 | AF117829.1 | blue |
| ENSG00000251158 | AC131392.2 | blue |
| ENSG00000251230 | MIR3945HG | blue |
| ENSG00000251414 | AC138956.2 | blue |
| ENSG00000251432 | AC108062.1 | blue |
| ENSG00000251450 | RASGRF2-AS1 | blue |
| ENSG00000251495 | PPIAP11 | blue |
| ENSG00000251513 | LIX1-AS1 | blue |
| ENSG00000251596 | HADHAP1 | blue |
| ENSG00000251600 | AC139713.2 | blue |
| ENSG00000251692 | PTX4 | blue |
| ENSG00000251739 | RNU6-1053P | blue |
| ENSG00000251819 | RNU6-322P | blue |
| ENSG00000252464 | RN7SKP70 | blue |
| ENSG00000252503 | RNU6-531P | blue |
| ENSG00000252690 | SCARNA15 | blue |
| ENSG00000253210 | AC040970.1 | blue |
| ENSG00000253330 | AC024451.2 | blue |
| ENSG00000253341 | AC115837.1 | blue |
| ENSG00000253352 | TUG1 | blue |
| ENSG00000253476 | AC091185.1 | blue |
| ENSG00000253506 | NACA2 | blue |
| ENSG00000253590 | IGLV3-13 | blue |
| ENSG00000253626 | EIF5AL1 | blue |
| ENSG00000253641 | LINCR-0001 | blue |

| | | |
|---|---|---|
| ENSG00000253710 | ALG11 | blue |
| ENSG00000253736 | AC022217.3 | blue |
| ENSG00000253865 | AC131025.1 | blue |
| ENSG00000253878 | AC087752.3 | blue |
| ENSG00000254093 | PINX1 | blue |
| ENSG00000254198 | AC113191.1 | blue |
| ENSG00000254317 | AC022973.4 | blue |
| ENSG00000254389 | RHPN1-AS1 | blue |
| ENSG00000254528 | AP000757.1 | blue |
| ENSG00000254681 | PKD1P5 | blue |
| ENSG00000254704 | AP002358.2 | blue |
| ENSG00000254718 | AL157756.1 | blue |
| ENSG00000254750 | CASP1P2 | blue |
| ENSG00000254774 | AC022239.1 | blue |
| ENSG00000254783 | AP003084.1 | blue |
| ENSG00000254802 | AC022182.2 | blue |
| ENSG00000254876 | AL590705.5 | blue |
| ENSG00000254941 | AP000866.4 | blue |
| ENSG00000254986 | DPP3 | blue |
| ENSG00000254999 | BRK1 | blue |
| ENSG00000255089 | AC136475.4 | blue |
| ENSG00000255135 | AP002360.2 | blue |
| ENSG00000255139 | AP000442.2 | blue |
| ENSG00000255153 | TOLLIP-AS1 | blue |
| ENSG00000255198 | SNHG9 | blue |
| ENSG00000255328 | AC136475.5 | blue |
| ENSG00000255363 | AP001189.5 | blue |
| ENSG00000255422 | AP002954.1 | blue |
| ENSG00000255443 | CD44-AS1 | blue |
| ENSG00000255455 | AP003486.1 | blue |
| ENSG00000255559 | ZNF252P-AS1 | blue |
| ENSG00000255624 | AC073585.1 | blue |
| ENSG00000255642 | PABPC1P4 | blue |
| ENSG00000255893 | AP000786.1 | blue |
| ENSG00000255970 | LINC02421 | blue |
| ENSG00000256053 | APOPT1 | blue |
| ENSG00000256069 | A2MP1 | blue |
| ENSG00000256073 | URB1-AS1 | blue |
| ENSG00000256092 | AC137767.1 | blue |
| ENSG00000256128 | LINC00944 | blue |
| ENSG00000256155 | AC022075.2 | blue |
| ENSG00000256249 | AC026333.3 | blue |
| ENSG00000256269 | HMBS | blue |
| ENSG00000256271 | CACNA1C-AS2 | blue |
| ENSG00000256293 | AC135279.2 | blue |
| ENSG00000256433 | AC005840.3 | blue |
| ENSG00000256582 | LINC02390 | blue |
| ENSG00000256612 | CYP2B7P | blue |

| | | |
|---|---|---|
| ENSG00000256667 | KLRA1P | blue |
| ENSG00000256720 | BTG1P1 | blue |
| ENSG00000256806 | C17orf100 | blue |
| ENSG00000257122 | RRN3P3 | blue |
| ENSG00000257167 | TMPO-AS1 | blue |
| ENSG00000257226 | AC079584.2 | blue |
| ENSG00000257243 | AC020612.1 | blue |
| ENSG00000257261 | AC008014.1 | blue |
| ENSG00000257335 | MGAM | blue |
| ENSG00000257433 | AC004241.1 | blue |
| ENSG00000257556 | LINC02298 | blue |
| ENSG00000257594 | GALNT4 | blue |
| ENSG00000257596 | AC078778.1 | blue |
| ENSG00000257605 | AC073611.1 | blue |
| ENSG00000257800 | FNBP1P1 | blue |
| ENSG00000257809 | AC034102.5 | blue |
| ENSG00000257815 | LINC01481 | blue |
| ENSG00000257878 | AC007298.2 | blue |
| ENSG00000257923 | CUX1 | blue |
| ENSG00000258057 | BCDIN3D-AS1 | blue |
| ENSG00000258136 | AC007622.2 | blue |
| ENSG00000258199 | AC073896.4 | blue |
| ENSG00000258297 | AP001157.1 | blue |
| ENSG00000258302 | AC025034.1 | blue |
| ENSG00000258376 | AC004846.1 | blue |
| ENSG00000258405 | ZNF578 | blue |
| ENSG00000258441 | LINC00641 | blue |
| ENSG00000258512 | LINC00239 | blue |
| ENSG00000258534 | AL132712.1 | blue |
| ENSG00000258545 | RHOXF1-AS1 | blue |
| ENSG00000258646 | AL049780.1 | blue |
| ENSG00000258654 | AC026495.1 | blue |
| ENSG00000258702 | AL137786.1 | blue |
| ENSG00000258741 | AC091544.3 | blue |
| ENSG00000258926 | AL355916.2 | blue |
| ENSG00000259005 | AC005479.2 | blue |
| ENSG00000259030 | FPGT-TNNI3K | blue |
| ENSG00000259205 | PRKXP1 | blue |
| ENSG00000259211 | AC013356.2 | blue |
| ENSG00000259236 | GOLGA8VP | blue |
| ENSG00000259248 | USP3-AS1 | blue |
| ENSG00000259250 | AC018904.1 | blue |
| ENSG00000259298 | AC012170.2 | blue |
| ENSG00000259349 | AC011921.1 | blue |
| ENSG00000259363 | AC090825.1 | blue |
| ENSG00000259381 | AC090695.1 | blue |
| ENSG00000259408 | AC010809.2 | blue |
| ENSG00000259418 | AC091117.2 | blue |

| | | |
|---|---|---|
| ENSG00000259436 | AC010247.2 | blue |
| ENSG00000259456 | ADNP-AS1 | blue |
| ENSG00000259494 | MRPL46 | blue |
| ENSG00000259529 | AL136295.5 | blue |
| ENSG00000259577 | CERNA1 | blue |
| ENSG00000259628 | AC007000.3 | blue |
| ENSG00000259642 | ST20-AS1 | blue |
| ENSG00000259668 | AC066613.1 | blue |
| ENSG00000259673 | IQCH-AS1 | blue |
| ENSG00000259802 | AC012640.2 | blue |
| ENSG00000259917 | HNRNPLP2 | blue |
| ENSG00000259935 | AC009754.1 | blue |
| ENSG00000259943 | AL050341.2 | blue |
| ENSG00000259953 | AL138756.1 | blue |
| ENSG00000259962 | AC007342.2 | blue |
| ENSG00000259994 | AL353796.1 | blue |
| ENSG00000260007 | AC107871.1 | blue |
| ENSG00000260027 | HOXB7 | blue |
| ENSG00000260077 | AC104794.2 | blue |
| ENSG00000260081 | AF274858.3 | blue |
| ENSG00000260101 | AC008074.3 | blue |
| ENSG00000260160 | AC011468.1 | blue |
| ENSG00000260260 | SNHG19 | blue |
| ENSG00000260306 | AC092375.2 | blue |
| ENSG00000260331 | AC079148.1 | blue |
| ENSG00000260335 | AC133555.4 | blue |
| ENSG00000260428 | SCX | blue |
| ENSG00000260464 | AL049796.1 | blue |
| ENSG00000260490 | MYL12BP1 | blue |
| ENSG00000260641 | AC114811.2 | blue |
| ENSG00000260661 | AC116903.2 | blue |
| ENSG00000260668 | AC093536.1 | blue |
| ENSG00000260708 | AL118516.1 | blue |
| ENSG00000260742 | AC009962.1 | blue |
| ENSG00000260774 | AC021087.3 | blue |
| ENSG00000260778 | AC009065.4 | blue |
| ENSG00000260808 | AP003096.1 | blue |
| ENSG00000260810 | AL135818.2 | blue |
| ENSG00000260816 | AC027279.1 | blue |
| ENSG00000260903 | XKR7 | blue |
| ENSG00000260917 | AL158212.3 | blue |
| ENSG00000260979 | AC022167.3 | blue |
| ENSG00000260997 | AC004847.1 | blue |
| ENSG00000261000 | AC244034.2 | blue |
| ENSG00000261019 | AC010132.4 | blue |
| ENSG00000261040 | WFDC21P | blue |
| ENSG00000261055 | AL450468.2 | blue |
| ENSG00000261061 | AC092718.4 | blue |

| | | |
|---|---|---|
| ENSG00000261094 | AC007066.2 | blue |
| ENSG00000261114 | AC012181.2 | blue |
| ENSG00000261208 | AL365475.1 | blue |
| ENSG00000261211 | AL031123.2 | blue |
| ENSG00000261220 | AC103706.1 | blue |
| ENSG00000261222 | AC064805.1 | blue |
| ENSG00000261229 | AC021483.2 | blue |
| ENSG00000261253 | AC137932.2 | blue |
| ENSG00000261308 | FIGNL2 | blue |
| ENSG00000261324 | AC010168.2 | blue |
| ENSG00000261351 | AC116913.1 | blue |
| ENSG00000261360 | AC010491.1 | blue |
| ENSG00000261371 | PECAM1 | blue |
| ENSG00000261433 | AC002347.1 | blue |
| ENSG00000261441 | AC124068.2 | blue |
| ENSG00000261455 | LINC01003 | blue |
| ENSG00000261485 | PAN3-AS1 | blue |
| ENSG00000261490 | AC005674.2 | blue |
| ENSG00000261526 | AC012615.1 | blue |
| ENSG00000261584 | AL513548.1 | blue |
| ENSG00000261596 | AC005632.2 | blue |
| ENSG00000261643 | AL590235.1 | blue |
| ENSG00000261716 | AC239868.2 | blue |
| ENSG00000261737 | AL049597.2 | blue |
| ENSG00000261804 | AC007342.4 | blue |
| ENSG00000262039 | AC091180.5 | blue |
| ENSG00000262147 | AC124283.2 | blue |
| ENSG00000262251 | AC087388.1 | blue |
| ENSG00000262370 | AC108134.3 | blue |
| ENSG00000262434 | AC087392.4 | blue |
| ENSG00000262655 | SPON1 | blue |
| ENSG00000262814 | MRPL12 | blue |
| ENSG00000262902 | MTCO1P40 | blue |
| ENSG00000262919 | FAM58A | blue |
| ENSG00000263002 | ZNF234 | blue |
| ENSG00000263142 | LRRC37A17P | blue |
| ENSG00000263307 | AC007216.4 | blue |
| ENSG00000263326 | AC133552.4 | blue |
| ENSG00000263366 | ABHD17AP5 | blue |
| ENSG00000263412 | AC004477.1 | blue |
| ENSG00000263464 | PPIAL4C | blue |
| ENSG00000263482 | ANTXRLP1 | blue |
| ENSG00000263787 | SKAP1-AS1 | blue |
| ENSG00000263843 | AC022211.2 | blue |
| ENSG00000263874 | LINC00672 | blue |
| ENSG00000263887 | AC053481.2 | blue |
| ENSG00000263916 | AC100778.2 | blue |
| ENSG00000264006 | AKR1C8P | blue |

| | | |
|---|---|---|
| ENSG00000264187 | AC055811.2 | blue |
| ENSG00000264278 | AC027575.2 | blue |
| ENSG00000264281 | AC016596.1 | blue |
| ENSG00000264659 | AC064805.2 | blue |
| ENSG00000264745 | TTC39C-AS1 | blue |
| ENSG00000264812 | AC132938.2 | blue |
| ENSG00000265148 | TSPOAP1-AS1 | blue |
| ENSG00000265298 | AC132812.1 | blue |
| ENSG00000265354 | TIMM23 | blue |
| ENSG00000265579 | AC023301.1 | blue |
| ENSG00000265666 | RARA-AS1 | blue |
| ENSG00000265798 | AC138207.6 | blue |
| ENSG00000266074 | BAHCC1 | blue |
| ENSG00000266088 | AC004585.1 | blue |
| ENSG00000266145 | AP001004.1 | blue |
| ENSG00000266378 | AC005224.3 | blue |
| ENSG00000266389 | AC002091.2 | blue |
| ENSG00000266402 | SNHG25 | blue |
| ENSG00000266472 | MRPS21 | blue |
| ENSG00000266524 | GDF10 | blue |
| ENSG00000266601 | AC018521.6 | blue |
| ENSG00000266708 | AP001793.2 | blue |
| ENSG00000266783 | AP005136.2 | blue |
| ENSG00000266907 | AC006116.1 | blue |
| ENSG00000266970 | AC061992.1 | blue |
| ENSG00000267009 | AC007780.1 | blue |
| ENSG00000267023 | LRRC37A16P | blue |
| ENSG00000267049 | AC002398.1 | blue |
| ENSG00000267078 | AC015802.1 | blue |
| ENSG00000267100 | ILF3-AS1 | blue |
| ENSG00000267123 | LINC02081 | blue |
| ENSG00000267213 | AC007773.1 | blue |
| ENSG00000267219 | AC010504.1 | blue |
| ENSG00000267254 | ZNF790-AS1 | blue |
| ENSG00000267279 | AC090409.1 | blue |
| ENSG00000267312 | AC015911.4 | blue |
| ENSG00000267317 | AC027307.2 | blue |
| ENSG00000267322 | SNHG22 | blue |
| ENSG00000267336 | EIF4A2P1 | blue |
| ENSG00000267390 | AC036176.1 | blue |
| ENSG00000267419 | AC011477.1 | blue |
| ENSG00000267472 | AC005332.3 | blue |
| ENSG00000267475 | AC008736.1 | blue |
| ENSG00000267534 | S1PR2 | blue |
| ENSG00000267544 | AC007229.1 | blue |
| ENSG00000267565 | AC011477.3 | blue |
| ENSG00000267568 | AC016168.2 | blue |
| ENSG00000267710 | EDDM13 | blue |

| | | |
|---|---|---|
| ENSG00000267809 | NDUFV2P1 | blue |
| ENSG00000267852 | AOC1 | blue |
| ENSG00000268027 | SARM1 | blue |
| ENSG00000268043 | TMEM132A | blue |
| ENSG00000268225 | BAIAP2L1 | blue |
| ENSG00000268279 | ADIPOR2 | blue |
| ENSG00000268357 | PROM1 | blue |
| ENSG00000268412 | CDK11A | blue |
| ENSG00000268439 | DLEC1 | blue |
| ENSG00000268510 | PLEKHG6 | blue |
| ENSG00000268521 | MASP2 | blue |
| ENSG00000268658 | CLCN6 | blue |
| ENSG00000268751 | UTP18 | blue |
| ENSG00000268836 | EXTL3 | blue |
| ENSG00000268903 | CALCOCO1 | blue |
| ENSG00000268970 | POLR3B | blue |
| ENSG00000269001 | MATR3 | blue |
| ENSG00000269176 | SLC11A1 | blue |
| ENSG00000269235 | WWTR1 | blue |
| ENSG00000269246 | SLC7A9 | blue |
| ENSG00000269293 | SLC45A4 | blue |
| ENSG00000269656 | HSD17B6 | blue |
| ENSG00000269713 | RTEL1-TNFRSF6B | blue |
| ENSG00000269814 | MIPEP | blue |
| ENSG00000269929 | TFB1M | blue |
| ENSG00000269934 | PEX3 | blue |
| ENSG00000269951 | DAPK2 | blue |
| ENSG00000269973 | FLT4 | blue |
| ENSG00000269981 | MRI1 | blue |
| ENSG00000270011 | RIPOR3 | blue |
| ENSG00000270031 | RETSAT | blue |
| ENSG00000270067 | TG | blue |
| ENSG00000270147 | TRIT1 | blue |
| ENSG00000270175 | PHKA2 | blue |
| ENSG00000270339 | GPM6B | blue |
| ENSG00000270344 | CP | blue |
| ENSG00000270419 | KIAA0556 | blue |
| ENSG00000270504 | CC2D2A | blue |
| ENSG00000270589 | MRPS10 | blue |
| ENSG00000270605 | TNFRSF9 | blue |
| ENSG00000270659 | CLPTM1L | blue |
| ENSG00000270872 | PTCD2 | blue |
| ENSG00000270878 | FAM160A2 | blue |
| ENSG00000270882 | THOC3 | blue |
| ENSG00000270972 | PRSS8 | blue |
| ENSG00000271046 | NNAT | blue |
| ENSG00000271254 | NRIP2 | blue |
| ENSG00000271259 | PLEKHH1 | blue |

| | | |
|---|---|---|
| ENSG00000271270 | FAM168A | blue |
| ENSG00000271452 | RELT | blue |
| ENSG00000271538 | ZC3H11A | blue |
| ENSG00000271551 | UNKL | blue |
| ENSG00000271576 | ALDH18A1 | blue |
| ENSG00000271614 | HDAC7 | blue |
| ENSG00000271631 | MRPS35 | blue |
| ENSG00000271643 | SEZ6 | blue |
| ENSG00000271734 | EIF4B | blue |
| ENSG00000271757 | AHRR | blue |
| ENSG00000271781 | SUGP2 | blue |
| ENSG00000271862 | BCAS1 | blue |
| ENSG00000271869 | ATXN3 | blue |
| ENSG00000271966 | STAG3 | blue |
| ENSG00000272002 | PLPP1 | blue |
| ENSG00000272037 | METTL22 | blue |
| ENSG00000272054 | GUCY2C | blue |
| ENSG00000272076 | MNT | blue |
| ENSG00000272087 | WIPI1 | blue |
| ENSG00000272097 | GBA2 | blue |
| ENSG00000272112 | TRIP13 | blue |
| ENSG00000272123 | CHFR | blue |
| ENSG00000272145 | P4HA2 | blue |
| ENSG00000272153 | MCM2 | blue |
| ENSG00000272226 | PANX2 | blue |
| ENSG00000272240 | SLC24A1 | blue |
| ENSG00000272255 | SLC12A1 | blue |
| ENSG00000272263 | TACR2 | blue |
| ENSG00000272341 | BCAP29 | blue |
| ENSG00000272375 | TUBA3D | blue |
| ENSG00000272382 | UNG | blue |
| ENSG00000272447 | PLXNA2 | blue |
| ENSG00000272476 | SPAG5 | blue |
| ENSG00000272523 | UBE2T | blue |
| ENSG00000272525 | PPP1R12B | blue |
| ENSG00000272644 | AMPH | blue |
| ENSG00000272650 | ZCWPW1 | blue |
| ENSG00000272677 | PKD2L2 | blue |
| ENSG00000272711 | TP53INP2 | blue |
| ENSG00000272719 | MYH7B | blue |
| ENSG00000272746 | DUSP13 | blue |
| ENSG00000272758 | DNM2 | blue |
| ENSG00000272799 | SRCAP | blue |
| ENSG00000272800 | CROCCP3 | blue |
| ENSG00000272810 | CXCL2 | blue |
| ENSG00000272832 | COL4A4 | blue |
| ENSG00000272839 | ATP8B1 | blue |
| ENSG00000272842 | NUFIP1 | blue |

| | | |
|---|---|---|
| ENSG00000272871 | ZNF586 | blue |
| ENSG00000272894 | ZNF264 | blue |
| ENSG00000272910 | STARD7 | blue |
| ENSG00000272927 | FAT2 | blue |
| ENSG00000272931 | MYBPC2 | blue |
| ENSG00000272977 | SH3BP2 | blue |
| ENSG00000272979 | NID2 | blue |
| ENSG00000272994 | SULT2B1 | blue |
| ENSG00000273001 | CNOT3 | blue |
| ENSG00000273017 | SLC4A11 | blue |
| ENSG00000273033 | MAVS | blue |
| ENSG00000273117 | GRAMD1A | blue |
| ENSG00000273129 | CMTM1 | blue |
| ENSG00000273136 | KCNH4 | blue |
| ENSG00000273174 | BIRC5 | blue |
| ENSG00000273249 | RAB11FIP3 | blue |
| ENSG00000273284 | DTX2 | blue |
| ENSG00000273306 | LAMB1 | blue |
| ENSG00000273353 | NLRP1 | blue |
| ENSG00000273448 | ESR1 | blue |
| ENSG00000273456 | ANGPT2 | blue |
| ENSG00000273464 | RPGRIP1 | blue |
| ENSG00000273486 | SEMA6A | blue |
| ENSG00000273489 | CAPN3 | blue |
| ENSG00000273576 | SEMA4G | blue |
| ENSG00000273619 | ITPR3 | blue |
| ENSG00000273749 | MAST3 | blue |
| ENSG00000273802 | MYO9B | blue |
| ENSG00000273837 | MKNK2 | blue |
| ENSG00000274105 | UPB1 | blue |
| ENSG00000274173 | PPM1F | blue |
| ENSG00000274184 | TOP3B | blue |
| ENSG00000274210 | CCDC134 | blue |
| ENSG00000274211 | SLC25A17 | blue |
| ENSG00000274227 | FAM118A | blue |
| ENSG00000274245 | CDKN3 | blue |
| ENSG00000274373 | SAMD15 | blue |
| ENSG00000274376 | LGMN | blue |
| ENSG00000274460 | SLC10A1 | blue |
| ENSG00000274561 | KIAA0391 | blue |
| ENSG00000274653 | PABPC1L | blue |
| ENSG00000274824 | DIDO1 | blue |
| ENSG00000274964 | GID8 | blue |
| ENSG00000274987 | RPS10P5 | blue |
| ENSG00000275120 | ANGPT4 | blue |
| ENSG00000275131 | CDH20 | blue |
| ENSG00000275234 | MYOM1 | blue |
| ENSG00000275278 | GPR143 | blue |

| | | |
|---|---|---|
| ENSG00000275318 | RBBP7 | blue |
| ENSG00000275413 | PBDC1 | blue |
| ENSG00000275426 | BEX4 | blue |
| ENSG00000275454 | SLC25A15 | blue |
| ENSG00000275494 | CLN5 | blue |
| ENSG00000275582 | NFAT5 | blue |
| ENSG00000275601 | LONP2 | blue |
| ENSG00000275632 | CYB5B | blue |
| ENSG00000275713 | SETD6 | blue |
| ENSG00000275793 | SLC7A6OS | blue |
| ENSG00000275835 | SLC7A6 | blue |
| ENSG00000275881 | ESRP2 | blue |
| ENSG00000276045 | ABCC1 | blue |
| ENSG00000276058 | KNOP1 | blue |
| ENSG00000276071 | AAGAB | blue |
| ENSG00000276115 | ACSBG1 | blue |
| ENSG00000276136 | CEMIP | blue |
| ENSG00000276248 | IKBKB | blue |
| ENSG00000276345 | INTS10 | blue |
| ENSG00000276476 | FAM32A | blue |
| ENSG00000276517 | EPHX3 | blue |
| ENSG00000276529 | CCNE1 | blue |
| ENSG00000276593 | RAB3D | blue |
| ENSG00000276651 | PLPPR2 | blue |
| ENSG00000276672 | HPN | blue |
| ENSG00000276710 | ERF | blue |
| ENSG00000276814 | WDR91 | blue |
| ENSG00000276916 | DNAH11 | blue |
| ENSG00000277020 | H2AFV | blue |
| ENSG00000277130 | RNF32 | blue |
| ENSG00000277152 | HOXA2 | blue |
| ENSG00000277218 | HOXA3 | blue |
| ENSG00000277246 | FSCN3 | blue |
| ENSG00000277299 | FBXO24 | blue |
| ENSG00000277406 | PPP1R17 | blue |
| ENSG00000277450 | RBM28 | blue |
| ENSG00000277595 | EZH2 | blue |
| ENSG00000277654 | AKNA | blue |
| ENSG00000277692 | KCNT1 | blue |
| ENSG00000277778 | RAPGEF1 | blue |
| ENSG00000277851 | ABCA2 | blue |
| ENSG00000278053 | PBLD | blue |
| ENSG00000278107 | RAPGEFL1 | blue |
| ENSG00000278156 | P2RX1 | blue |
| ENSG00000278206 | PIGL | blue |
| ENSG00000278311 | CAMTA2 | blue |
| ENSG00000278383 | BLMH | blue |
| ENSG00000278661 | HSD17B1P1 | blue |

| | | |
|---|---|---|
| ENSG00000278768 | HSD17B1 | blue |
| ENSG00000278834 | CNTNAP1 | blue |
| ENSG00000278917 | FAM20A | blue |
| ENSG00000278959 | MYH3 | blue |
| ENSG00000278970 | PHF12 | blue |
| ENSG00000279058 | LRP2BP | blue |
| ENSG00000279088 | UFSP2 | blue |
| ENSG00000279092 | ZBTB16 | blue |
| ENSG00000279117 | SNX15 | blue |
| ENSG00000279118 | ATG2A | blue |
| ENSG00000279149 | ARHGEF17 | blue |
| ENSG00000279227 | NECTIN1 | blue |
| ENSG00000279277 | ACCS | blue |
| ENSG00000279311 | PRPF40B | blue |
| ENSG00000279329 | IL23A | blue |
| ENSG00000279342 | BCL7A | blue |
| ENSG00000279356 | CYP27B1 | blue |
| ENSG00000279392 | TNS2 | blue |
| ENSG00000279423 | MAGOHB | blue |
| ENSG00000279452 | OGFOD2 | blue |
| ENSG00000279519 | EIF2B1 | blue |
| ENSG00000279528 | ENDOU | blue |
| ENSG00000279561 | PHC1 | blue |
| ENSG00000279602 | SRSF9 | blue |
| ENSG00000279622 | MRPL18 | blue |
| ENSG00000279689 | SAYSD1 | blue |
| ENSG00000279693 | B3GAT2 | blue |
| ENSG00000279722 | TTK | blue |
| ENSG00000279741 | TMCO6 | blue |
| ENSG00000279926 | HAVCR1 | blue |
| ENSG00000279989 | THG1L | blue |
| ENSG00000280057 | SKP1 | blue |
| ENSG00000280091 | ERGIC1 | blue |
| ENSG00000280107 | BNIP1 | blue |
| ENSG00000280129 | UNC5A | blue |
| ENSG00000280159 | TIMMDC1 | blue |
| ENSG00000280173 | USP4 | blue |
| ENSG00000280347 | HYAL1 | blue |
| ENSG00000280355 | KIAA1257 | blue |
| ENSG00000280739 | DNAH1 | blue |
| ENSG00000280789 | INO80B | blue |
| ENSG00000280832 | CCT4 | blue |
| ENSG00000281026 | PNO1 | blue |
| ENSG00000281087 | GRIN3B | blue |
| ENSG00000281344 | AC119396.2 | blue |
| ENSG00000281398 | AC243960.1 | blue |
| ENSG00000281468 | NBPF12 | blue |
| ENSG00000281501 | AC010487.1 | blue |

| | | |
|---|---|---|
| ENSG00000281706 | AC090004.1 | blue |
| ENSG00000281958 | VN1R81P | blue |
| ENSG00000282034 | TRMT112P6 | blue |
| ENSG00000282051 | EMG1 | blue |
| ENSG00000282780 | IFNL3P1 | blue |
| ENSG00000282939 | VN1R83P | blue |
| ENSG00000283041 | LINC00664 | blue |
| ENSG00000283138 | SCGB1B2P | blue |
| ENSG00000283288 | Z69706.1 | blue |
| ENSG00000283312 | AL627309.7 | blue |
| ENSG00000283361 | AC022150.2 | blue |
| ENSG00000283429 | AC092070.2 | blue |
| ENSG00000283929 | AP001160.3 | blue |
| ENSG00000284024 | ZNF350-AS1 | blue |
| ENSG00000284657 | AC011445.2 | blue |
| ENSG00000284690 | ZSCAN16-AS1 | blue |
| ENSG00000284713 | NOP53-AS1 | blue |
| ENSG00000284747 | NBPF9 | blue |
| ENSG00000002726 | AC008403.3 | brown |
| ENSG00000004139 | AL158152.1 | brown |
| ENSG00000006118 | AL353593.2 | brown |
| ENSG00000006453 | AC090181.2 | brown |
| ENSG00000006831 | AC010969.2 | brown |
| ENSG00000007062 | AL627309.8 | brown |
| ENSG00000008128 | ZNF559-ZNF177 | brown |
| ENSG00000008226 | AL020997.3 | brown |
| ENSG00000008323 | AC008536.1 | brown |
| ENSG00000009724 | AC068620.3 | brown |
| ENSG00000011021 | AC023509.4 | brown |
| ENSG00000011260 | AC243756.1 | brown |
| ENSG00000012232 | POC1B-AS1 | brown |
| ENSG00000012822 | CAHM | brown |
| ENSG00000013503 | AL391422.3 | brown |
| ENSG00000015479 | AL158163.1 | brown |
| ENSG00000018280 | AL353622.1 | brown |
| ENSG00000018408 | AC079610.2 | brown |
| ENSG00000021488 | SRGAP2D | brown |
| ENSG00000022567 | AL136038.4 | brown |
| ENSG00000025423 | HIST2H4A | brown |
| ENSG00000026036 | AC136475.9 | brown |
| ENSG00000027001 | AL512631.1 | brown |
| ENSG00000029639 | AC240274.1 | brown |
| ENSG00000034693 | AC010201.1 | brown |
| ENSG00000035664 | TMCC1-AS1 | brown |
| ENSG00000037280 | AC005034.5 | brown |
| ENSG00000037757 | LINC02427 | brown |
| ENSG00000042062 | AL355297.4 | brown |
| ENSG00000042445 | AL359504.2 | brown |

| | | |
|---|---|---|
| ENSG00000042832 | ATP2B1-AS1 | brown |
| ENSG00000043514 | AL139041.1 | brown |
| ENSG00000044446 | AC112220.4 | brown |
| ENSG00000046653 | Z98200.1 | brown |
| ENSG00000047457 | AP002360.3 | brown |
| ENSG00000047578 | AC026740.1 | brown |
| ENSG00000048342 | AC104118.1 | brown |
| ENSG00000048544 | AC026979.3 | brown |
| ENSG00000049249 | AC021321.1 | brown |
| ENSG00000049656 | AC010904.2 | brown |
| ENSG00000049883 | AP002907.1 | brown |
| ENSG00000051009 | AC007390.2 | brown |
| ENSG00000051596 | AC090186.1 | brown |
| ENSG00000052344 | AC080013.4 | brown |
| ENSG00000053438 | AL024498.1 | brown |
| ENSG00000053702 | AC011374.2 | brown |
| ENSG00000054690 | AC008966.2 | brown |
| ENSG00000054965 | NFYC-AS1 | brown |
| ENSG00000054967 | AL365330.1 | brown |
| ENSG00000058673 | AL136985.2 | brown |
| ENSG00000059145 | AC004908.1 | brown |
| ENSG00000059573 | AC113361.1 | brown |
| ENSG00000061273 | AC034198.2 | brown |
| ENSG00000061794 | AL137003.2 | brown |
| ENSG00000063015 | AC026979.4 | brown |
| ENSG00000063046 | AC025171.5 | brown |
| ENSG00000063438 | AL135925.1 | brown |
| ENSG00000064607 | AL024507.2 | brown |
| ENSG00000064787 | LINC01023 | brown |
| ENSG00000066427 | AC099522.2 | brown |
| ENSG00000066923 | AC097468.3 | brown |
| ENSG00000067113 | AC110792.4 | brown |
| ENSG00000067365 | AC124016.1 | brown |
| ENSG00000070019 | AC019069.1 | brown |
| ENSG00000070444 | AC006483.2 | brown |
| ENSG00000070540 | AP005131.7 | brown |
| ENSG00000070610 | AC083798.2 | brown |
| ENSG00000071539 | AC006238.1 | brown |
| ENSG00000072609 | AC021851.1 | brown |
| ENSG00000072682 | U91328.4 | brown |
| ENSG00000073111 | AC022296.3 | brown |
| ENSG00000073150 | AC011899.3 | brown |
| ENSG00000074621 | AL391834.1 | brown |
| ENSG00000074803 | AL159169.2 | brown |
| ENSG00000075073 | AC004982.2 | brown |
| ENSG00000075790 | AC090425.2 | brown |
| ENSG00000075886 | AC107464.3 | brown |
| ENSG00000076248 | AC099568.2 | brown |

| | | |
|---|---|---|
| ENSG00000076356 | AL008721.2 | brown |
| ENSG00000076382 | AC093388.1 | brown |
| ENSG00000077152 | AC012360.3 | brown |
| ENSG00000077157 | AL731533.2 | brown |
| ENSG00000078053 | AP000240.1 | brown |
| ENSG00000078487 | LINC02035 | brown |
| ENSG00000078795 | AC144652.1 | brown |
| ENSG00000078804 | PACERR | brown |
| ENSG00000078814 | NBPF26 | brown |
| ENSG00000079393 | AC108673.2 | brown |
| ENSG00000079805 | BX649632.1 | brown |
| ENSG00000080603 | AP001033.2 | brown |
| ENSG00000080947 | AC018690.1 | brown |
| ENSG00000081041 | AL008718.3 | brown |
| ENSG00000081052 | AC006480.2 | brown |
| ENSG00000081923 | AC064836.3 | brown |
| ENSG00000083635 | AP000238.1 | brown |
| ENSG00000083828 | AC096992.2 | brown |
| ENSG00000083844 | AC008264.2 | brown |
| ENSG00000084090 | AC009283.1 | brown |
| ENSG00000086570 | AL121832.2 | brown |
| ENSG00000086967 | CYFIP1 | brown |
| ENSG00000087266 | HIST1H2BG | brown |
| ENSG00000087303 | AC018755.4 | brown |
| ENSG00000088002 | AC084824.3 | brown |
| ENSG00000088038 | AL035661.1 | brown |
| ENSG00000088836 | AC011815.2 | brown |
| ENSG00000088888 | U1 | brown |
| ENSG00000089351 | SOCS7 | brown |
| ENSG00000089505 | AC073575.2 | brown |
| ENSG00000089558 | AL391597.1 | brown |
| ENSG00000089685 | AC148476.1 | brown |
| ENSG00000090565 | ADAMTS7P1 | brown |
| ENSG00000091073 | AC092119.2 | brown |
| ENSG00000091136 | AC005332.6 | brown |
| ENSG00000091592 | AC106782.6 | brown |
| ENSG00000091831 | MIR7152 | brown |
| ENSG00000091879 | AC026356.1 | brown |
| ENSG00000092200 | AC092794.1 | brown |
| ENSG00000092421 | AC048382.5 | brown |
| ENSG00000092529 | AC241952.1 | brown |
| ENSG00000095539 | AC010503.4 | brown |
| ENSG00000096433 | AC012150.2 | brown |
| ENSG00000099308 | AL136981.2 | brown |
| ENSG00000099331 | AC002553.4 | brown |
| ENSG00000099875 | AC253576.2 | brown |
| ENSG00000100024 | AC105020.6 | brown |
| ENSG00000100034 | AC133552.6 | brown |

| | | |
|---|---|---|
| ENSG00000100038 | AL031670.1 | brown |
| ENSG00000100147 | AC011330.2 | brown |
| ENSG00000100372 | AL035461.2 | brown |
| ENSG00000100376 | HIST1H2BH | brown |
| ENSG00000100526 | RIMBP3 | brown |
| ENSG00000100583 | TUBGCP5 | brown |
| ENSG00000100600 | Metazoa_SRP | brown |
| ENSG00000100652 | ORAI1 | brown |
| ENSG00000100890 | STMN1P1 | brown |
| ENSG00000101104 | AC074138.1 | brown |
| ENSG00000101191 | AC026356.2 | brown |
| ENSG00000101193 | AC016957.2 | brown |
| ENSG00000101278 | AL442125.1 | brown |
| ENSG00000101280 | AC004556.1 | brown |
| ENSG00000101542 | LINC00540 | brown |
| ENSG00000101605 | AL133243.3 | brown |
| ENSG00000101850 | AP001505.2 | brown |
| ENSG00000102054 | AC022306.3 | brown |
| ENSG00000102390 | AC007950.2 | brown |
| ENSG00000102409 | AL161891.1 | brown |
| ENSG00000102743 | CSPG4P10 | brown |
| ENSG00000102805 | AC004801.6 | brown |
| ENSG00000102908 | AL442125.2 | brown |
| ENSG00000102910 | AL590096.1 | brown |
| ENSG00000103018 | AC073569.3 | brown |
| ENSG00000103037 | AC110048.2 | brown |
| ENSG00000103061 | AL139123.1 | brown |
| ENSG00000103064 | AL157762.1 | brown |
| ENSG00000103067 | AC084876.1 | brown |
| ENSG00000103222 | AC243756.2 | brown |
| ENSG00000103550 | AC002094.4 | brown |
| ENSG00000103591 | AC007546.1 | brown |
| ENSG00000103740 | AC087633.2 | brown |
| ENSG00000103888 | AL121583.1 | brown |
| ENSG00000104365 | PGM5P2 | brown |
| ENSG00000104613 | LINC02391 | brown |
| ENSG00000105058 | DDX52 | brown |
| ENSG00000105131 | AC027575.4 | brown |
| ENSG00000105173 | TSC22D1-AS1 | brown |
| ENSG00000105514 | AL031320.2 | brown |
| ENSG00000105520 | GGNBP2 | brown |
| ENSG00000105707 | AL031673.1 | brown |
| ENSG00000105722 | TRAJ37 | brown |
| ENSG00000105875 | BACE1-AS | brown |
| ENSG00000105877 | AC073508.3 | brown |
| ENSG00000105968 | AC006213.6 | brown |
| ENSG00000105982 | AC006378.2 | brown |
| ENSG00000105996 | HEIH | brown |

| | | |
|---|---|---|
| ENSG00000105997 | AGAP14 | brown |
| ENSG00000106328 | AC022400.7 | brown |
| ENSG00000106336 | AC025678.3 | brown |
| ENSG00000106341 | AP001972.5 | brown |
| ENSG00000106344 | AC093535.2 | brown |
| ENSG00000106462 | AL356750.1 | brown |
| ENSG00000106948 | AC009303.4 | brown |
| ENSG00000107147 | AC012020.1 | brown |
| ENSG00000107263 | AC092999.1 | brown |
| ENSG00000107331 | AC020910.5 | brown |
| ENSG00000108187 | AP000866.6 | brown |
| ENSG00000108352 | AC007610.4 | brown |
| ENSG00000108405 | AL158801.5 | brown |
| ENSG00000108474 | AL445363.3 | brown |
| ENSG00000108509 | AC006277.1 | brown |
| ENSG00000108578 | AC007382.1 | brown |
| ENSG00000108785 | AC115618.3 | brown |
| ENSG00000108786 | AL845472.1 | brown |
| ENSG00000108797 | AC109326.1 | brown |
| ENSG00000108950 | AC025280.2 | brown |
| ENSG00000109063 | AC022400.8 | brown |
| ENSG00000109118 | AC099521.3 | brown |
| ENSG00000109771 | AC007342.7 | brown |
| ENSG00000109775 | AC007342.8 | brown |
| ENSG00000109906 | AL138831.3 | brown |
| ENSG00000110025 | AC011815.3 | brown |
| ENSG00000110046 | AL022069.2 | brown |
| ENSG00000110237 | AC020895.2 | brown |
| ENSG00000110400 | AL022393.1 | brown |
| ENSG00000110455 | AL132780.5 | brown |
| ENSG00000110844 | AC016583.1 | brown |
| ENSG00000110944 | AC104447.1 | brown |
| ENSG00000110987 | AC000123.3 | brown |
| ENSG00000111012 | AL132656.4 | brown |
| ENSG00000111077 | EIF1B-AS1 | brown |
| ENSG00000111196 | PAGR1 | brown |
| ENSG00000111325 | GSEC | brown |
| ENSG00000111361 | N4BP2L2-IT2 | brown |
| ENSG00000111405 | AC135068.6 | brown |
| ENSG00000111752 | HELLPAR | brown |
| ENSG00000111786 | SNHG4 | brown |
| ENSG00000112110 | AC006504.8 | brown |
| ENSG00000112167 | AC104662.3 | brown |
| ENSG00000112309 | LINC01012 | brown |
| ENSG00000112742 | AC245427.3 | brown |
| ENSG00000113119 | AC106886.5 | brown |
| ENSG00000113249 | AC010507.1 | brown |
| ENSG00000113272 | AC245427.9 | brown |

| | | |
|---|---|---|
| ENSG00000113558 | AC245088.2 | brown |
| ENSG00000113719 | AC008038.1 | brown |
| ENSG00000113734 | AC006207.1 | brown |
| ENSG00000113763 | AC138517.2 | brown |
| ENSG00000113845 | AC017104.4 | brown |
| ENSG00000114316 | AL160396.2 | brown |
| ENSG00000114378 | MIR1244-3 | brown |
| ENSG00000114656 | MIR5010 | brown |
| ENSG00000114841 | AC069544.2 | brown |
| ENSG00000115274 | AL031432.5 | brown |
| ENSG00000115484 | AC079325.2 | brown |
| ENSG00000115946 | AP003071.5 | brown |
| ENSG00000116032 | AL034417.4 | brown |
| ENSG00000116133 | DHCR24 | brown |
| ENSG00000116205 | TCEANC2 | brown |
| ENSG00000116212 | LRRC42 | brown |
| ENSG00000116237 | ICMT | brown |
| ENSG00000116251 | RPL22 | brown |
| ENSG00000116337 | AMPD2 | brown |
| ENSG00000116396 | KCNC4 | brown |
| ENSG00000116455 | WDR77 | brown |
| ENSG00000116525 | TRIM62 | brown |
| ENSG00000116584 | ARHGEF2 | brown |
| ENSG00000116604 | MEF2D | brown |
| ENSG00000116852 | KIF21B | brown |
| ENSG00000117115 | PADI2 | brown |
| ENSG00000117242 | PINK1-AS | brown |
| ENSG00000117400 | MPL | brown |
| ENSG00000117632 | STMN1 | brown |
| ENSG00000117899 | MESD | brown |
| ENSG00000118292 | C1orf54 | brown |
| ENSG00000118298 | CA14 | brown |
| ENSG00000118369 | USP35 | brown |
| ENSG00000118777 | ABCG2 | brown |
| ENSG00000118816 | CCNI | brown |
| ENSG00000118898 | PPL | brown |
| ENSG00000118939 | UCHL3 | brown |
| ENSG00000119185 | ITGB1BP1 | brown |
| ENSG00000119242 | CCDC92 | brown |
| ENSG00000119703 | ZC2HC1C | brown |
| ENSG00000119725 | ZNF410 | brown |
| ENSG00000119737 | GPR75 | brown |
| ENSG00000119771 | KLHL29 | brown |
| ENSG00000119772 | DNMT3A | brown |
| ENSG00000120055 | C10orf95 | brown |
| ENSG00000120093 | HOXB3 | brown |
| ENSG00000120215 | MLANA | brown |
| ENSG00000120333 | MRPS14 | brown |

| | | |
|---|---|---|
| ENSG00000120437 | ACAT2 | brown |
| ENSG00000120645 | IQSEC3 | brown |
| ENSG00000120656 | TAF12 | brown |
| ENSG00000120903 | CHRNA2 | brown |
| ENSG00000120913 | PDLIM2 | brown |
| ENSG00000120963 | ZNF706 | brown |
| ENSG00000121101 | TEX14 | brown |
| ENSG00000121152 | NCAPH | brown |
| ENSG00000121281 | ADCY7 | brown |
| ENSG00000121413 | ZSCAN18 | brown |
| ENSG00000121454 | LHX4 | brown |
| ENSG00000121764 | HCRTR1 | brown |
| ENSG00000121769 | FABP3 | brown |
| ENSG00000121933 | TMIGD3 | brown |
| ENSG00000122861 | PLAU | brown |
| ENSG00000122952 | ZWINT | brown |
| ENSG00000123179 | EBPL | brown |
| ENSG00000123201 | GUCY1B2 | brown |
| ENSG00000123454 | DBH | brown |
| ENSG00000123472 | ATPAF1 | brown |
| ENSG00000123485 | HJURP | brown |
| ENSG00000123500 | COL10A1 | brown |
| ENSG00000123689 | G0S2 | brown |
| ENSG00000123870 | ZNF137P | brown |
| ENSG00000123892 | RAB38 | brown |
| ENSG00000124067 | SLC12A4 | brown |
| ENSG00000124091 | GCNT7 | brown |
| ENSG00000124134 | KCNS1 | brown |
| ENSG00000124251 | TP53TG5 | brown |
| ENSG00000124557 | BTN1A1 | brown |
| ENSG00000124574 | ABCC10 | brown |
| ENSG00000124596 | OARD1 | brown |
| ENSG00000124602 | UNC5CL | brown |
| ENSG00000124721 | DNAH8 | brown |
| ENSG00000124787 | RPP40 | brown |
| ENSG00000124920 | MYRF | brown |
| ENSG00000125084 | WNT1 | brown |
| ENSG00000125170 | DOK4 | brown |
| ENSG00000125356 | NDUFA1 | brown |
| ENSG00000125375 | ATP5S | brown |
| ENSG00000125447 | GGA3 | brown |
| ENSG00000125510 | OPRL1 | brown |
| ENSG00000125618 | PAX8 | brown |
| ENSG00000125735 | TNFSF14 | brown |
| ENSG00000125772 | GPCPD1 | brown |
| ENSG00000125810 | CD93 | brown |
| ENSG00000125966 | MMP24 | brown |
| ENSG00000125997 | BPIFB9P | brown |

| | | |
|---|---|---|
| ENSG00000126500 | FLRT1 | brown |
| ENSG00000126698 | DNAJC8 | brown |
| ENSG00000126746 | ZNF384 | brown |
| ENSG00000126838 | PZP | brown |
| ENSG00000126895 | AVPR2 | brown |
| ENSG00000127252 | HRASLS | brown |
| ENSG00000127366 | TAS2R5 | brown |
| ENSG00000127418 | FGFRL1 | brown |
| ENSG00000127507 | ADGRE2 | brown |
| ENSG00000127527 | EPS15L1 | brown |
| ENSG00000127663 | KDM4B | brown |
| ENSG00000127922 | SEM1 | brown |
| ENSG00000127957 | PMS2P3 | brown |
| ENSG00000128285 | MCHR1 | brown |
| ENSG00000128346 | C22orf23 | brown |
| ENSG00000128731 | HERC2 | brown |
| ENSG00000128789 | PSMG2 | brown |
| ENSG00000128918 | ALDH1A2 | brown |
| ENSG00000129450 | SIGLEC9 | brown |
| ENSG00000129484 | PARP2 | brown |
| ENSG00000129757 | CDKN1C | brown |
| ENSG00000130119 | GNL3L | brown |
| ENSG00000130167 | TSPAN16 | brown |
| ENSG00000130177 | CDC16 | brown |
| ENSG00000130307 | USHBP1 | brown |
| ENSG00000130348 | QRSL1 | brown |
| ENSG00000130363 | RSPH3 | brown |
| ENSG00000130544 | ZNF557 | brown |
| ENSG00000130638 | ATXN10 | brown |
| ENSG00000130751 | NPAS1 | brown |
| ENSG00000130764 | LRRC47 | brown |
| ENSG00000130943 | PKDREJ | brown |
| ENSG00000131044 | TTLL9 | brown |
| ENSG00000131097 | HIGD1B | brown |
| ENSG00000131100 | ATP6V1E1 | brown |
| ENSG00000131242 | RAB11FIP4 | brown |
| ENSG00000131269 | ABCB7 | brown |
| ENSG00000131351 | HAUS8 | brown |
| ENSG00000131355 | ADGRE3 | brown |
| ENSG00000131373 | HACL1 | brown |
| ENSG00000131378 | RFTN1 | brown |
| ENSG00000131470 | PSMC3IP | brown |
| ENSG00000131471 | AOC3 | brown |
| ENSG00000131480 | AOC2 | brown |
| ENSG00000131503 | ANKHD1 | brown |
| ENSG00000131508 | UBE2D2 | brown |
| ENSG00000131730 | CKMT2 | brown |
| ENSG00000131797 | CLUHP3 | brown |

| | | |
|---|---|---|
| ENSG00000131876 | SNRPA1 | brown |
| ENSG00000131944 | FAAP24 | brown |
| ENSG00000131969 | ABHD12B | brown |
| ENSG00000132010 | ZNF20 | brown |
| ENSG00000132330 | SCLY | brown |
| ENSG00000132341 | RAN | brown |
| ENSG00000132423 | COQ3 | brown |
| ENSG00000132464 | ENAM | brown |
| ENSG00000132470 | ITGB4 | brown |
| ENSG00000132801 | ZSWIM3 | brown |
| ENSG00000132874 | SLC14A2 | brown |
| ENSG00000133026 | MYH10 | brown |
| ENSG00000133028 | SCO1 | brown |
| ENSG00000133121 | STARD13 | brown |
| ENSG00000133142 | TCEAL4 | brown |
| ENSG00000133401 | PDZD2 | brown |
| ENSG00000133983 | COX16 | brown |
| ENSG00000133997 | MED6 | brown |
| ENSG00000134013 | LOXL2 | brown |
| ENSG00000134056 | MRPS36 | brown |
| ENSG00000134121 | CHL1 | brown |
| ENSG00000134262 | AP4B1 | brown |
| ENSG00000134597 | RBMX2 | brown |
| ENSG00000134716 | CYP2J2 | brown |
| ENSG00000134812 | GIF | brown |
| ENSG00000134815 | DHX34 | brown |
| ENSG00000134830 | C5AR2 | brown |
| ENSG00000134864 | GGACT | brown |
| ENSG00000134882 | UBAC2 | brown |
| ENSG00000135407 | AVIL | brown |
| ENSG00000135436 | FAM186B | brown |
| ENSG00000135437 | RDH5 | brown |
| ENSG00000135486 | HNRNPA1 | brown |
| ENSG00000135547 | HEY2 | brown |
| ENSG00000135643 | KCNMB4 | brown |
| ENSG00000135709 | KIAA0513 | brown |
| ENSG00000135749 | PCNX2 | brown |
| ENSG00000135775 | COG2 | brown |
| ENSG00000135835 | KIAA1614 | brown |
| ENSG00000135845 | PIGC | brown |
| ENSG00000135953 | MFSD9 | brown |
| ENSG00000135972 | MRPS9 | brown |
| ENSG00000135976 | ANKRD36 | brown |
| ENSG00000136010 | ALDH1L2 | brown |
| ENSG00000136045 | PWP1 | brown |
| ENSG00000136104 | RNASEH2B | brown |
| ENSG00000136159 | NUDT15 | brown |
| ENSG00000136197 | C7orf25 | brown |

| | | |
|---|---|---|
| ENSG00000136206 | SPDYE1 | brown |
| ENSG00000136261 | BZW2 | brown |
| ENSG00000136367 | ZFHX2 | brown |
| ENSG00000136371 | MTHFS | brown |
| ENSG00000136383 | ALPK3 | brown |
| ENSG00000136710 | CCDC115 | brown |
| ENSG00000136848 | DAB2IP | brown |
| ENSG00000136867 | SLC31A2 | brown |
| ENSG00000136872 | ALDOB | brown |
| ENSG00000136895 | GARNL3 | brown |
| ENSG00000136935 | GOLGA1 | brown |
| ENSG00000136938 | ANP32B | brown |
| ENSG00000137074 | APTX | brown |
| ENSG00000137265 | IRF4 | brown |
| ENSG00000137809 | ITGA11 | brown |
| ENSG00000137857 | DUOX1 | brown |
| ENSG00000138463 | DIRC2 | brown |
| ENSG00000138614 | INTS14 | brown |
| ENSG00000138615 | CILP | brown |
| ENSG00000138617 | PARP16 | brown |
| ENSG00000138794 | CASP6 | brown |
| ENSG00000138834 | MAPK8IP3 | brown |
| ENSG00000139053 | PDE6H | brown |
| ENSG00000139351 | SYCP3 | brown |
| ENSG00000139433 | GLTP | brown |
| ENSG00000139610 | CELA1 | brown |
| ENSG00000139625 | MAP3K12 | brown |
| ENSG00000139668 | WDFY2 | brown |
| ENSG00000139908 | TSSK4 | brown |
| ENSG00000140009 | ESR2 | brown |
| ENSG00000140323 | DISP2 | brown |
| ENSG00000140374 | ETFA | brown |
| ENSG00000140395 | WDR61 | brown |
| ENSG00000140548 | ZNF710 | brown |
| ENSG00000140650 | PMM2 | brown |
| ENSG00000140678 | ITGAX | brown |
| ENSG00000140688 | C16orf58 | brown |
| ENSG00000140950 | TLDC1 | brown |
| ENSG00000140987 | ZSCAN32 | brown |
| ENSG00000140995 | DEF8 | brown |
| ENSG00000141028 | CDRT15P1 | brown |
| ENSG00000141127 | PRPSAP2 | brown |
| ENSG00000141337 | ARSG | brown |
| ENSG00000141404 | GNAL | brown |
| ENSG00000141519 | CCDC40 | brown |
| ENSG00000141527 | CARD14 | brown |
| ENSG00000141956 | PRDM15 | brown |
| ENSG00000141977 | CIB3 | brown |

| | | |
|---|---|---|
| ENSG00000142405 | NLRP12 | brown |
| ENSG00000142686 | C1orf216 | brown |
| ENSG00000142864 | SERBP1 | brown |
| ENSG00000143156 | NME7 | brown |
| ENSG00000143199 | ADCY10 | brown |
| ENSG00000143256 | PFDN2 | brown |
| ENSG00000143257 | NR1I3 | brown |
| ENSG00000143333 | RGS16 | brown |
| ENSG00000143373 | ZNF687 | brown |
| ENSG00000143409 | MINDY1 | brown |
| ENSG00000143412 | ANXA9 | brown |
| ENSG00000143418 | CERS2 | brown |
| ENSG00000143450 | OAZ3 | brown |
| ENSG00000143545 | RAB13 | brown |
| ENSG00000143552 | NUP210L | brown |
| ENSG00000143614 | GATAD2B | brown |
| ENSG00000143653 | SCCPDH | brown |
| ENSG00000143816 | WNT9A | brown |
| ENSG00000143858 | SYT2 | brown |
| ENSG00000144031 | ANKRD53 | brown |
| ENSG00000144426 | NBEAL1 | brown |
| ENSG00000144741 | SLC25A26 | brown |
| ENSG00000144791 | LIMD1 | brown |
| ENSG00000144867 | SRPRB | brown |
| ENSG00000145050 | MANF | brown |
| ENSG00000145217 | SLC26A1 | brown |
| ENSG00000145220 | LYAR | brown |
| ENSG00000145623 | OSMR | brown |
| ENSG00000145850 | TIMD4 | brown |
| ENSG00000145920 | CPLX2 | brown |
| ENSG00000145996 | CDKAL1 | brown |
| ENSG00000146197 | SCUBE3 | brown |
| ENSG00000146670 | CDCA5 | brown |
| ENSG00000146733 | PSPH | brown |
| ENSG00000146828 | SLC12A9 | brown |
| ENSG00000146955 | RAB19 | brown |
| ENSG00000147099 | HDAC8 | brown |
| ENSG00000147224 | PRPS1 | brown |
| ENSG00000147400 | CETN2 | brown |
| ENSG00000147485 | PXDNL | brown |
| ENSG00000147586 | MRPS28 | brown |
| ENSG00000147799 | ARHGAP39 | brown |
| ENSG00000147872 | PLIN2 | brown |
| ENSG00000148120 | C9orf3 | brown |
| ENSG00000148200 | NR6A1 | brown |
| ENSG00000148468 | FAM171A1 | brown |
| ENSG00000148484 | RSU1 | brown |
| ENSG00000148660 | CAMK2G | brown |

| | | |
|---|---|---|
| ENSG00000148795 | CYP17A1 | brown |
| ENSG00000148848 | ADAM12 | brown |
| ENSG00000149100 | EIF3M | brown |
| ENSG00000149418 | ST14 | brown |
| ENSG00000149474 | KAT14 | brown |
| ENSG00000149527 | PLCH2 | brown |
| ENSG00000149532 | CPSF7 | brown |
| ENSG00000149547 | EI24 | brown |
| ENSG00000149571 | KIRREL3 | brown |
| ENSG00000150455 | TIRAP | brown |
| ENSG00000150459 | SAP18 | brown |
| ENSG00000150753 | CCT5 | brown |
| ENSG00000151006 | PRSS53 | brown |
| ENSG00000151067 | CACNA1C | brown |
| ENSG00000151320 | AKAP6 | brown |
| ENSG00000151327 | FAM177A1 | brown |
| ENSG00000152104 | PTPN14 | brown |
| ENSG00000152128 | TMEM163 | brown |
| ENSG00000152439 | ZNF773 | brown |
| ENSG00000153012 | LGI2 | brown |
| ENSG00000153044 | CENPH | brown |
| ENSG00000153086 | ACMSD | brown |
| ENSG00000153157 | SYCP2L | brown |
| ENSG00000153234 | NR4A2 | brown |
| ENSG00000153558 | FBXL2 | brown |
| ENSG00000153574 | RPIA | brown |
| ENSG00000154040 | CABYR | brown |
| ENSG00000154274 | C4orf19 | brown |
| ENSG00000154548 | SRSF12 | brown |
| ENSG00000154582 | ELOC | brown |
| ENSG00000154710 | RABGEF1 | brown |
| ENSG00000154856 | APCDD1 | brown |
| ENSG00000154874 | CCDC144B | brown |
| ENSG00000154889 | MPPE1 | brown |
| ENSG00000155657 | TTN | brown |
| ENSG00000155749 | ALS2CR12 | brown |
| ENSG00000155876 | RRAGA | brown |
| ENSG00000155906 | RMND1 | brown |
| ENSG00000156042 | CFAP70 | brown |
| ENSG00000156261 | CCT8 | brown |
| ENSG00000156398 | SFXN2 | brown |
| ENSG00000156502 | SUPV3L1 | brown |
| ENSG00000156574 | NODAL | brown |
| ENSG00000156711 | MAPK13 | brown |
| ENSG00000157036 | EXOG | brown |
| ENSG00000157111 | TMEM171 | brown |
| ENSG00000157315 | TMED6 | brown |
| ENSG00000157349 | DDX19B | brown |

| | | |
|---|---|---|
| ENSG00000157388 | CACNA1D | brown |
| ENSG00000157429 | ZNF19 | brown |
| ENSG00000157456 | CCNB2 | brown |
| ENSG00000157881 | PANK4 | brown |
| ENSG00000157890 | MEGF11 | brown |
| ENSG00000157999 | ANKRD61 | brown |
| ENSG00000158042 | MRPL17 | brown |
| ENSG00000158089 | GALNT14 | brown |
| ENSG00000158296 | SLC13A3 | brown |
| ENSG00000158482 | SNX29P1 | brown |
| ENSG00000158486 | DNAH3 | brown |
| ENSG00000158525 | CPA5 | brown |
| ENSG00000158528 | PPP1R9A | brown |
| ENSG00000158604 | TMED4 | brown |
| ENSG00000158805 | ZNF276 | brown |
| ENSG00000158813 | EDA | brown |
| ENSG00000158874 | APOA2 | brown |
| ENSG00000158887 | MPZ | brown |
| ENSG00000159314 | ARHGAP27 | brown |
| ENSG00000159433 | STARD9 | brown |
| ENSG00000159588 | CCDC17 | brown |
| ENSG00000159723 | AGRP | brown |
| ENSG00000159871 | LYPD5 | brown |
| ENSG00000160051 | IQCC | brown |
| ENSG00000160193 | WDR4 | brown |
| ENSG00000160285 | LSS | brown |
| ENSG00000160401 | CFAP157 | brown |
| ENSG00000160606 | TLCD1 | brown |
| ENSG00000160613 | PCSK7 | brown |
| ENSG00000160685 | ZBTB7B | brown |
| ENSG00000160716 | CHRNB2 | brown |
| ENSG00000160796 | NBEAL2 | brown |
| ENSG00000160961 | ZNF333 | brown |
| ENSG00000160991 | ORAI2 | brown |
| ENSG00000161010 | MRNIP | brown |
| ENSG00000161542 | PRPSAP1 | brown |
| ENSG00000161664 | ASB16 | brown |
| ENSG00000161960 | EIF4A1 | brown |
| ENSG00000162341 | TPCN2 | brown |
| ENSG00000162385 | MAGOH | brown |
| ENSG00000162396 | PARS2 | brown |
| ENSG00000162490 | DRAXIN | brown |
| ENSG00000162552 | WNT4 | brown |
| ENSG00000162746 | FCRLB | brown |
| ENSG00000162804 | SNED1 | brown |
| ENSG00000162888 | C1orf147 | brown |
| ENSG00000162924 | REL | brown |
| ENSG00000162931 | TRIM17 | brown |

| | | |
|---|---|---|
| ENSG00000162959 | MEMO1 | brown |
| ENSG00000163050 | COQ8A | brown |
| ENSG00000163449 | TMEM169 | brown |
| ENSG00000163541 | SUCLG1 | brown |
| ENSG00000163806 | SPDYA | brown |
| ENSG00000163875 | MEAF6 | brown |
| ENSG00000163945 | UVSSA | brown |
| ENSG00000164032 | H2AFZ | brown |
| ENSG00000164082 | GRM2 | brown |
| ENSG00000164106 | SCRG1 | brown |
| ENSG00000164296 | TIGD6 | brown |
| ENSG00000164308 | ERAP2 | brown |
| ENSG00000164663 | USP49 | brown |
| ENSG00000164687 | FABP5 | brown |
| ENSG00000164776 | PHKG1 | brown |
| ENSG00000164818 | DNAAF5 | brown |
| ENSG00000164855 | TMEM184A | brown |
| ENSG00000164970 | FAM219A | brown |
| ENSG00000165046 | LETM2 | brown |
| ENSG00000165061 | ZMAT4 | brown |
| ENSG00000165115 | KIF27 | brown |
| ENSG00000165181 | C9orf84 | brown |
| ENSG00000165480 | SKA3 | brown |
| ENSG00000165502 | RPL36AL | brown |
| ENSG00000165507 | C10orf10 | brown |
| ENSG00000165511 | C10orf25 | brown |
| ENSG00000165678 | GHITM | brown |
| ENSG00000165801 | ARHGEF40 | brown |
| ENSG00000165819 | METTL3 | brown |
| ENSG00000165863 | C10orf82 | brown |
| ENSG00000165923 | AGBL2 | brown |
| ENSG00000165995 | CACNB2 | brown |
| ENSG00000166035 | LIPC | brown |
| ENSG00000166323 | C11orf65 | brown |
| ENSG00000166333 | ILK | brown |
| ENSG00000166343 | MSS51 | brown |
| ENSG00000166348 | USP54 | brown |
| ENSG00000166359 | WDR88 | brown |
| ENSG00000166398 | KIAA0355 | brown |
| ENSG00000166402 | TUB | brown |
| ENSG00000166482 | MFAP4 | brown |
| ENSG00000166503 | HDGFL3 | brown |
| ENSG00000166546 | BEAN1 | brown |
| ENSG00000166592 | RRAD | brown |
| ENSG00000166619 | BLCAP | brown |
| ENSG00000166716 | ZNF592 | brown |
| ENSG00000166788 | SAAL1 | brown |
| ENSG00000166800 | LDHAL6A | brown |

| | | |
|---|---|---|
| ENSG00000166839 | ANKDD1A | brown |
| ENSG00000166888 | STAT6 | brown |
| ENSG00000166987 | MBD6 | brown |
| ENSG00000167210 | LOXHD1 | brown |
| ENSG00000167257 | RNF214 | brown |
| ENSG00000167315 | ACAA2 | brown |
| ENSG00000167395 | ZNF646 | brown |
| ENSG00000167434 | CA4 | brown |
| ENSG00000167524 | SGK494 | brown |
| ENSG00000167548 | KMT2D | brown |
| ENSG00000167595 | PROSER3 | brown |
| ENSG00000167615 | LENG8 | brown |
| ENSG00000167676 | PLIN4 | brown |
| ENSG00000167699 | GLOD4 | brown |
| ENSG00000167840 | ZNF232 | brown |
| ENSG00000167987 | VPS37C | brown |
| ENSG00000168010 | ATG16L2 | brown |
| ENSG00000168038 | ULK4 | brown |
| ENSG00000168234 | TTC39C | brown |
| ENSG00000168298 | HIST1H1E | brown |
| ENSG00000168386 | FILIP1L | brown |
| ENSG00000168496 | FEN1 | brown |
| ENSG00000168679 | SLC16A4 | brown |
| ENSG00000168781 | PPIP5K1 | brown |
| ENSG00000168852 | TPTE2P5 | brown |
| ENSG00000168917 | SLC35G2 | brown |
| ENSG00000168939 | SPRY3 | brown |
| ENSG00000168970 | JMJD7-PLA2G4B | brown |
| ENSG00000169021 | UQCRFS1 | brown |
| ENSG00000169087 | HSPBAP1 | brown |
| ENSG00000169291 | SHE | brown |
| ENSG00000169439 | SDC2 | brown |
| ENSG00000169495 | HTRA4 | brown |
| ENSG00000169609 | C15orf40 | brown |
| ENSG00000169635 | HIC2 | brown |
| ENSG00000169682 | SPNS1 | brown |
| ENSG00000169758 | TMEM266 | brown |
| ENSG00000169902 | TPST1 | brown |
| ENSG00000169918 | OTUD7A | brown |
| ENSG00000169991 | IFFO2 | brown |
| ENSG00000170075 | GPR37L1 | brown |
| ENSG00000170092 | SPDYE5 | brown |
| ENSG00000170231 | FABP6 | brown |
| ENSG00000170382 | LRRN2 | brown |
| ENSG00000170396 | ZNF804A | brown |
| ENSG00000170482 | SLC23A1 | brown |
| ENSG00000170667 | RASA4B | brown |
| ENSG00000170734 | POLH | brown |

| | | |
|---|---|---|
| ENSG00000170917 | NUDT6 | brown |
| ENSG00000171094 | ALK | brown |
| ENSG00000171217 | CLDN20 | brown |
| ENSG00000171295 | ZNF440 | brown |
| ENSG00000171401 | KRT13 | brown |
| ENSG00000171466 | ZNF562 | brown |
| ENSG00000171695 | LKAAEAR1 | brown |
| ENSG00000171757 | LRRC34 | brown |
| ENSG00000171790 | SLFNL1 | brown |
| ENSG00000171865 | RNASEH1 | brown |
| ENSG00000171962 | DRC3 | brown |
| ENSG00000172006 | ZNF554 | brown |
| ENSG00000172315 | TP53RK | brown |
| ENSG00000172568 | FNDC9 | brown |
| ENSG00000172687 | ZNF738 | brown |
| ENSG00000172771 | EFCAB12 | brown |
| ENSG00000172803 | SNX32 | brown |
| ENSG00000172830 | SSH3 | brown |
| ENSG00000172985 | SH3RF3 | brown |
| ENSG00000173212 | MAB21L3 | brown |
| ENSG00000173214 | MFSD4B | brown |
| ENSG00000173269 | MMRN2 | brown |
| ENSG00000173421 | CCDC36 | brown |
| ENSG00000173480 | ZNF417 | brown |
| ENSG00000173545 | ZNF622 | brown |
| ENSG00000173567 | ADGRF3 | brown |
| ENSG00000173614 | NMNAT1 | brown |
| ENSG00000173638 | SLC19A1 | brown |
| ENSG00000173678 | SPDYE2B | brown |
| ENSG00000173698 | ADGRG2 | brown |
| ENSG00000173727 | AP000769.1 | brown |
| ENSG00000173728 | C1orf100 | brown |
| ENSG00000173875 | ZNF791 | brown |
| ENSG00000174038 | C9orf131 | brown |
| ENSG00000174225 | ARL13A | brown |
| ENSG00000174353 | STAG3L3 | brown |
| ENSG00000174428 | GTF2IRD2B | brown |
| ENSG00000174482 | LINGO2 | brown |
| ENSG00000174483 | BBS1 | brown |
| ENSG00000174501 | ANKRD36C | brown |
| ENSG00000174586 | ZNF497 | brown |
| ENSG00000174611 | KY | brown |
| ENSG00000174628 | IQCK | brown |
| ENSG00000174672 | BRSK2 | brown |
| ENSG00000174775 | HRAS | brown |
| ENSG00000174827 | PDZK1 | brown |
| ENSG00000174885 | NLRP6 | brown |
| ENSG00000174945 | AMZ1 | brown |

| | | |
|---|---|---|
| ENSG00000174989 | FBXW8 | brown |
| ENSG00000175084 | DES | brown |
| ENSG00000175106 | TVP23C | brown |
| ENSG00000175182 | FAM131A | brown |
| ENSG00000175274 | TP53I11 | brown |
| ENSG00000175544 | CABP4 | brown |
| ENSG00000175782 | SLC35E3 | brown |
| ENSG00000175787 | ZNF169 | brown |
| ENSG00000175894 | TSPEAR | brown |
| ENSG00000175899 | A2M | brown |
| ENSG00000176124 | DLEU1 | brown |
| ENSG00000176148 | TCP11L1 | brown |
| ENSG00000176153 | GPX2 | brown |
| ENSG00000176208 | ATAD5 | brown |
| ENSG00000176289 | IDSP1 | brown |
| ENSG00000176358 | TAC4 | brown |
| ENSG00000176396 | EID2 | brown |
| ENSG00000176563 | CNTD1 | brown |
| ENSG00000176593 | AC008969.1 | brown |
| ENSG00000176920 | FUT2 | brown |
| ENSG00000176927 | EFCAB5 | brown |
| ENSG00000176953 | NFATC2IP | brown |
| ENSG00000177045 | SIX5 | brown |
| ENSG00000177076 | ACER2 | brown |
| ENSG00000177082 | WDR73 | brown |
| ENSG00000177169 | ULK1 | brown |
| ENSG00000177191 | B3GNT8 | brown |
| ENSG00000177369 | FLJ40194 | brown |
| ENSG00000177406 | AC021054.1 | brown |
| ENSG00000177410 | ZFAS1 | brown |
| ENSG00000177494 | ZBED2 | brown |
| ENSG00000177590 | GIMAP3P | brown |
| ENSG00000177733 | HNRNPA0 | brown |
| ENSG00000177947 | ODF3 | brown |
| ENSG00000178127 | NDUFV2 | brown |
| ENSG00000178229 | ZNF543 | brown |
| ENSG00000178381 | ZFAND2A | brown |
| ENSG00000178386 | ZNF223 | brown |
| ENSG00000178404 | CEP295NL | brown |
| ENSG00000178440 | LINC00843 | brown |
| ENSG00000178460 | MCMDC2 | brown |
| ENSG00000178502 | KLHL11 | brown |
| ENSG00000178761 | FAM219B | brown |
| ENSG00000178803 | ADORA2A-AS1 | brown |
| ENSG00000178809 | TRIM73 | brown |
| ENSG00000178935 | ZNF552 | brown |
| ENSG00000179041 | RRS1 | brown |
| ENSG00000179082 | C9orf106 | brown |

| | | |
|---|---|---|
| ENSG00000179304 | FAM156B | brown |
| ENSG00000179528 | LBX2 | brown |
| ENSG00000179743 | FLJ37453 | brown |
| ENSG00000179761 | PIPOX | brown |
| ENSG00000179818 | PCBP1-AS1 | brown |
| ENSG00000179820 | MYADM | brown |
| ENSG00000179840 | PIK3CD-AS1 | brown |
| ENSG00000179899 | PHC1P1 | brown |
| ENSG00000179909 | ZNF154 | brown |
| ENSG00000179933 | C14orf119 | brown |
| ENSG00000180011 | ZADH2 | brown |
| ENSG00000180071 | ANKRD18A | brown |
| ENSG00000180139 | ACTA2-AS1 | brown |
| ENSG00000180198 | RCC1 | brown |
| ENSG00000180385 | EMC3-AS1 | brown |
| ENSG00000180409 | OR10AA1P | brown |
| ENSG00000180509 | KCNE1 | brown |
| ENSG00000180610 | ZBTB12BP | brown |
| ENSG00000180611 | MB21D2 | brown |
| ENSG00000180747 | SMG1P3 | brown |
| ENSG00000180846 | CSNK1G2-AS1 | brown |
| ENSG00000180878 | C11orf42 | brown |
| ENSG00000180979 | LRRC57 | brown |
| ENSG00000181090 | EHMT1 | brown |
| ENSG00000181191 | PJA1 | brown |
| ENSG00000181227 | DLSTP1 | brown |
| ENSG00000181322 | NME9 | brown |
| ENSG00000181409 | AATK | brown |
| ENSG00000181722 | ZBTB20 | brown |
| ENSG00000181800 | CELF2-AS1 | brown |
| ENSG00000181826 | RELL1 | brown |
| ENSG00000182095 | TNRC18 | brown |
| ENSG00000182108 | DEXI | brown |
| ENSG00000182257 | PRR34 | brown |
| ENSG00000182362 | YBEY | brown |
| ENSG00000182376 | AC138028.1 | brown |
| ENSG00000182586 | LINC00334 | brown |
| ENSG00000182648 | LINC01006 | brown |
| ENSG00000182841 | RRP7BP | brown |
| ENSG00000182993 | C12orf60 | brown |
| ENSG00000183091 | NEB | brown |
| ENSG00000183250 | LINC01547 | brown |
| ENSG00000183323 | CCDC125 | brown |
| ENSG00000183506 | PI4KAP2 | brown |
| ENSG00000183520 | UTP11 | brown |
| ENSG00000183569 | SERHL2 | brown |
| ENSG00000183666 | GUSBP1 | brown |
| ENSG00000183688 | RFLNB | brown |

| | | |
|---|---|---|
| ENSG00000183742 | MACC1 | brown |
| ENSG00000184154 | LRTOMT | brown |
| ENSG00000184220 | CMSS1 | brown |
| ENSG00000184260 | HIST2H2AC | brown |
| ENSG00000184261 | KCNK12 | brown |
| ENSG00000184361 | SPATA32 | brown |
| ENSG00000184389 | A3GALT2 | brown |
| ENSG00000184619 | KRBA2 | brown |
| ENSG00000184719 | RNLS | brown |
| ENSG00000184731 | FAM110C | brown |
| ENSG00000184828 | ZBTB7C | brown |
| ENSG00000184923 | NUTM2A | brown |
| ENSG00000185022 | MAFF | brown |
| ENSG00000185156 | MFSD6L | brown |
| ENSG00000185189 | NRBP2 | brown |
| ENSG00000185219 | ZNF445 | brown |
| ENSG00000185245 | GP1BA | brown |
| ENSG00000185359 | HGS | brown |
| ENSG00000185513 | L3MBTL1 | brown |
| ENSG00000185567 | AHNAK2 | brown |
| ENSG00000185664 | PMEL | brown |
| ENSG00000185684 | EP400NL | brown |
| ENSG00000185689 | C6orf201 | brown |
| ENSG00000185710 | SMG1P4 | brown |
| ENSG00000185730 | ZNF696 | brown |
| ENSG00000185761 | ADAMTSL5 | brown |
| ENSG00000185842 | DNAH14 | brown |
| ENSG00000185864 | NPIPB4 | brown |
| ENSG00000185900 | POMK | brown |
| ENSG00000186026 | ZNF284 | brown |
| ENSG00000186174 | BCL9L | brown |
| ENSG00000186222 | BLOC1S4 | brown |
| ENSG00000186354 | C9orf47 | brown |
| ENSG00000186481 | ANKRD20A5P | brown |
| ENSG00000186529 | CYP4F3 | brown |
| ENSG00000186532 | SMYD4 | brown |
| ENSG00000186704 | DTX2P1 | brown |
| ENSG00000186710 | CFAP73 | brown |
| ENSG00000186787 | SPIN2B | brown |
| ENSG00000186866 | POFUT2 | brown |
| ENSG00000186918 | ZNF395 | brown |
| ENSG00000186919 | ZACN | brown |
| ENSG00000187134 | AKR1C1 | brown |
| ENSG00000187514 | PTMA | brown |
| ENSG00000187650 | VMAC | brown |
| ENSG00000187653 | TMSB4XP8 | brown |
| ENSG00000187726 | DNAJB13 | brown |
| ENSG00000187792 | ZNF70 | brown |

| | | |
|---|---|---|
| ENSG00000187824 | TMEM220 | brown |
| ENSG00000187980 | PLA2G2C | brown |
| ENSG00000187984 | ANKRD19P | brown |
| ENSG00000188026 | RILPL1 | brown |
| ENSG00000188037 | CLCN1 | brown |
| ENSG00000188042 | ARL4C | brown |
| ENSG00000188060 | RAB42 | brown |
| ENSG00000188152 | NUTM2G | brown |
| ENSG00000188167 | TMPPE | brown |
| ENSG00000188185 | LINC00265 | brown |
| ENSG00000188211 | NCR3LG1 | brown |
| ENSG00000188266 | HYKK | brown |
| ENSG00000188277 | C15orf62 | brown |
| ENSG00000188315 | C3orf62 | brown |
| ENSG00000188352 | FOCAD | brown |
| ENSG00000188365 | AC092171.1 | brown |
| ENSG00000188487 | INSC | brown |
| ENSG00000188596 | CFAP54 | brown |
| ENSG00000188603 | CLN3 | brown |
| ENSG00000188739 | RBM34 | brown |
| ENSG00000189051 | RNF222 | brown |
| ENSG00000189129 | PLAC9 | brown |
| ENSG00000189134 | NKAPL | brown |
| ENSG00000189136 | UBE2Q2P1 | brown |
| ENSG00000189144 | ZNF573 | brown |
| ENSG00000196110 | ZNF699 | brown |
| ENSG00000196152 | ZNF79 | brown |
| ENSG00000196187 | TMEM63A | brown |
| ENSG00000196236 | XPNPEP3 | brown |
| ENSG00000196295 | AC005154.1 | brown |
| ENSG00000196312 | MFSD14C | brown |
| ENSG00000196329 | GIMAP5 | brown |
| ENSG00000196388 | INCA1 | brown |
| ENSG00000196422 | PPP1R26 | brown |
| ENSG00000196557 | CACNA1H | brown |
| ENSG00000196562 | SULF2 | brown |
| ENSG00000196584 | XRCC2 | brown |
| ENSG00000196605 | ZNF846 | brown |
| ENSG00000196811 | CHRNG | brown |
| ENSG00000196872 | KIAA1211L | brown |
| ENSG00000196935 | SRGAP1 | brown |
| ENSG00000196993 | NPIPB9 | brown |
| ENSG00000197283 | SYNGAP1 | brown |
| ENSG00000197353 | LYPD2 | brown |
| ENSG00000197361 | FBXL22 | brown |
| ENSG00000197429 | IPP | brown |
| ENSG00000197582 | GPX1P1 | brown |
| ENSG00000197608 | ZNF841 | brown |

| | | |
|---|---|---|
| ENSG00000197622 | CDC42SE1 | brown |
| ENSG00000197670 | AL157838.1 | brown |
| ENSG00000197714 | ZNF460 | brown |
| ENSG00000197813 | AC011450.1 | brown |
| ENSG00000197815 | AC122129.1 | brown |
| ENSG00000197816 | CCDC180 | brown |
| ENSG00000197837 | HIST4H4 | brown |
| ENSG00000197852 | FAM212B | brown |
| ENSG00000197857 | ZNF44 | brown |
| ENSG00000197976 | AKAP17A | brown |
| ENSG00000198178 | CLEC4C | brown |
| ENSG00000198223 | CSF2RA | brown |
| ENSG00000198400 | NTRK1 | brown |
| ENSG00000198466 | ZNF587 | brown |
| ENSG00000198482 | ZNF808 | brown |
| ENSG00000198496 | NBR2 | brown |
| ENSG00000198517 | MAFK | brown |
| ENSG00000198521 | ZNF43 | brown |
| ENSG00000198553 | KCNRG | brown |
| ENSG00000198556 | ZNF789 | brown |
| ENSG00000198625 | MDM4 | brown |
| ENSG00000198668 | CALM1 | brown |
| ENSG00000198711 | SSBP3-AS1 | brown |
| ENSG00000198722 | UNC13B | brown |
| ENSG00000198728 | LDB1 | brown |
| ENSG00000198837 | DENND4B | brown |
| ENSG00000198838 | RYR3 | brown |
| ENSG00000198909 | MAP3K3 | brown |
| ENSG00000200087 | SNORA73B | brown |
| ENSG00000200090 | Y_RNA | brown |
| ENSG00000200235 | SNORA27 | brown |
| ENSG00000200502 | Y_RNA | brown |
| ENSG00000200737 | Y_RNA | brown |
| ENSG00000200814 | RNU6-595P | brown |
| ENSG00000201098 | RNY1 | brown |
| ENSG00000201185 | RNA5SP202 | brown |
| ENSG00000201544 | SNORA16B | brown |
| ENSG00000201772 | SNORA5C | brown |
| ENSG00000202058 | RN7SKP80 | brown |
| ENSG00000202538 | RNU4-2 | brown |
| ENSG00000203416 | FAM32BP | brown |
| ENSG00000203667 | COX20 | brown |
| ENSG00000203709 | C1orf132 | brown |
| ENSG00000203721 | LINC00862 | brown |
| ENSG00000203739 | AL645568.1 | brown |
| ENSG00000203761 | MSTO2P | brown |
| ENSG00000203801 | LINC00222 | brown |
| ENSG00000203819 | HIST2H2BC | brown |

| | | |
|---|---|---|
| ENSG00000203872 | C6orf163 | brown |
| ENSG00000203914 | HSP90B3P | brown |
| ENSG00000204110 | LINC02520 | brown |
| ENSG00000204131 | NHSL2 | brown |
| ENSG00000204152 | TIMM23B | brown |
| ENSG00000204188 | GGNBP1 | brown |
| ENSG00000204253 | HNRNPCP2 | brown |
| ENSG00000204277 | LINC01993 | brown |
| ENSG00000204283 | LINC01973 | brown |
| ENSG00000204304 | PBX2 | brown |
| ENSG00000204345 | CD300LD | brown |
| ENSG00000204650 | LINC02210 | brown |
| ENSG00000204767 | FAM196B | brown |
| ENSG00000205037 | AC134312.1 | brown |
| ENSG00000205041 | AC118344.1 | brown |
| ENSG00000205085 | FAM71F2 | brown |
| ENSG00000205181 | LINC00654 | brown |
| ENSG00000205238 | SPDYE2 | brown |
| ENSG00000205277 | MUC12 | brown |
| ENSG00000205323 | SARNP | brown |
| ENSG00000205476 | CCDC85C | brown |
| ENSG00000205482 | SPDYE18 | brown |
| ENSG00000205542 | TMSB4X | brown |
| ENSG00000205559 | CHKB-AS1 | brown |
| ENSG00000205578 | POM121B | brown |
| ENSG00000205670 | SMIM11A | brown |
| ENSG00000205740 | AL359878.1 | brown |
| ENSG00000205755 | CRLF2 | brown |
| ENSG00000205763 | RP9P | brown |
| ENSG00000205771 | CATSPER2P1 | brown |
| ENSG00000205786 | LINC01531 | brown |
| ENSG00000205885 | C1RL-AS1 | brown |
| ENSG00000206052 | DOK6 | brown |
| ENSG00000206127 | GOLGA8O | brown |
| ENSG00000206149 | HERC2P9 | brown |
| ENSG00000206838 | SNORA5A | brown |
| ENSG00000207031 | SNORD59A | brown |
| ENSG00000207088 | SNORA7B | brown |
| ENSG00000207425 | Y_RNA | brown |
| ENSG00000207445 | SNORD15B | brown |
| ENSG00000207561 | MIR635 | brown |
| ENSG00000207741 | MIR590 | brown |
| ENSG00000208028 | MIR616 | brown |
| ENSG00000208772 | SNORD94 | brown |
| ENSG00000209042 | SNORD12C | brown |
| ENSG00000211454 | AKR7L | brown |
| ENSG00000211513 | MIR320E | brown |
| ENSG00000211643 | IGLV5-52 | brown |

| | | |
|---|---|---|
| ENSG00000211701 | TRGV1 | brown |
| ENSG00000211734 | TRBV5-1 | brown |
| ENSG00000211770 | TRBJ2-6 | brown |
| ENSG00000211778 | TRAV4 | brown |
| ENSG00000211780 | TRAV6 | brown |
| ENSG00000211788 | TRAV13-1 | brown |
| ENSG00000211789 | TRAV12-2 | brown |
| ENSG00000211790 | TRAV8-4 | brown |
| ENSG00000211884 | TRAJ5 | brown |
| ENSG00000211885 | TRAJ4 | brown |
| ENSG00000211886 | TRAJ3 | brown |
| ENSG00000211888 | TRAJ1 | brown |
| ENSG00000211900 | IGHJ6 | brown |
| ENSG00000212232 | SNORD17 | brown |
| ENSG00000212242 | RNA5SP219 | brown |
| ENSG00000212464 | SNORA12 | brown |
| ENSG00000212743 | AL137145.1 | brown |
| ENSG00000213018 | AL590762.1 | brown |
| ENSG00000213020 | ZNF611 | brown |
| ENSG00000213080 | AL354714.2 | brown |
| ENSG00000213149 | CNN2P9 | brown |
| ENSG00000213178 | AC074033.1 | brown |
| ENSG00000213190 | MLLT11 | brown |
| ENSG00000213204 | AL049697.1 | brown |
| ENSG00000213212 | NCLP1 | brown |
| ENSG00000213277 | MARCKSL1P1 | brown |
| ENSG00000213420 | GPC2 | brown |
| ENSG00000213433 | AC091982.1 | brown |
| ENSG00000213600 | U73169.1 | brown |
| ENSG00000213684 | LDHBP2 | brown |
| ENSG00000213713 | PIGCP1 | brown |
| ENSG00000213782 | DDX47 | brown |
| ENSG00000213801 | ZNF321P | brown |
| ENSG00000213809 | KLRK1 | brown |
| ENSG00000213865 | C8orf44 | brown |
| ENSG00000213888 | LINC01521 | brown |
| ENSG00000213901 | SLC23A3 | brown |
| ENSG00000213906 | LTB4R2 | brown |
| ENSG00000213918 | DNASE1 | brown |
| ENSG00000213963 | AC019080.1 | brown |
| ENSG00000214019 | AL034370.1 | brown |
| ENSG00000214021 | TTLL3 | brown |
| ENSG00000214029 | ZNF891 | brown |
| ENSG00000214046 | SMIM7 | brown |
| ENSG00000214135 | AC132008.2 | brown |
| ENSG00000214176 | PLEKHM1P1 | brown |
| ENSG00000214182 | PTMAP5 | brown |
| ENSG00000214279 | SCART1 | brown |

| | | |
|---|---|---|
| ENSG00000214331 | AC009053.1 | brown |
| ENSG00000214359 | RPL18P10 | brown |
| ENSG00000214391 | TUBAP2 | brown |
| ENSG00000214456 | PLIN5 | brown |
| ENSG00000214548 | MEG3 | brown |
| ENSG00000214655 | ZSWIM8 | brown |
| ENSG00000214708 | AC116407.1 | brown |
| ENSG00000214837 | LINC01347 | brown |
| ENSG00000214851 | LINC00612 | brown |
| ENSG00000214900 | LINC01588 | brown |
| ENSG00000215045 | GRID2IP | brown |
| ENSG00000215067 | ALOX12-AS1 | brown |
| ENSG00000215068 | AC025171.2 | brown |
| ENSG00000215154 | AC141586.1 | brown |
| ENSG00000215156 | AC138409.1 | brown |
| ENSG00000215158 | AC138409.2 | brown |
| ENSG00000215193 | PEX26 | brown |
| ENSG00000215196 | AC026790.1 | brown |
| ENSG00000215241 | LINC02449 | brown |
| ENSG00000215244 | AL137145.2 | brown |
| ENSG00000215388 | ACTG1P3 | brown |
| ENSG00000215397 | SCRT2 | brown |
| ENSG00000215424 | MCM3AP-AS1 | brown |
| ENSG00000215447 | BX322557.1 | brown |
| ENSG00000215492 | HNRNPA1P7 | brown |
| ENSG00000215493 | AC007731.2 | brown |
| ENSG00000215559 | ANKRD20A11P | brown |
| ENSG00000215630 | GUSBP9 | brown |
| ENSG00000215790 | SLC35E2 | brown |
| ENSG00000216331 | HIST1H1PS1 | brown |
| ENSG00000216809 | AL589993.1 | brown |
| ENSG00000217555 | CKLF | brown |
| ENSG00000217930 | PAM16 | brown |
| ENSG00000218226 | TATDN2P2 | brown |
| ENSG00000218357 | LINC01644 | brown |
| ENSG00000218502 | H2AFZP3 | brown |
| ENSG00000218713 | AL512378.1 | brown |
| ENSG00000218809 | AL391903.1 | brown |
| ENSG00000219102 | HNRNPA3P12 | brown |
| ENSG00000219222 | RPL12P47 | brown |
| ENSG00000219355 | RPL31P52 | brown |
| ENSG00000219392 | ZNF602P | brown |
| ENSG00000219410 | AC125494.1 | brown |
| ENSG00000219626 | FAM228B | brown |
| ENSG00000219891 | ZSCAN12P1 | brown |
| ENSG00000220008 | LINGO3 | brown |
| ENSG00000220305 | HNRNPH1P1 | brown |
| ENSG00000220323 | AC239868.1 | brown |

| | | |
|---|---|---|
| ENSG00000220804 | LINC01881 | brown |
| ENSG00000220937 | HNRNPA1P41 | brown |
| ENSG00000221025 | MIR1250 | brown |
| ENSG00000221949 | LINC01465 | brown |
| ENSG00000221962 | TMEM14EP | brown |
| ENSG00000221970 | OR2A1 | brown |
| ENSG00000221995 | TIAF1 | brown |
| ENSG00000222005 | LINC01118 | brown |
| ENSG00000222009 | BTBD19 | brown |
| ENSG00000222011 | FAM185A | brown |
| ENSG00000222046 | DCDC2B | brown |
| ENSG00000222112 | RN7SKP16 | brown |
| ENSG00000222365 | SNORD12B | brown |
| ENSG00000222937 | SNORD63B | brown |
| ENSG00000223060 | Y_RNA | brown |
| ENSG00000223117 | RN7SKP296 | brown |
| ENSG00000223313 | RNU6-516P | brown |
| ENSG00000223336 | RNU2-6P | brown |
| ENSG00000223356 | AL590666.1 | brown |
| ENSG00000223396 | RPS10P7 | brown |
| ENSG00000223401 | AC072022.1 | brown |
| ENSG00000223461 | AC004471.1 | brown |
| ENSG00000223522 | AC093690.1 | brown |
| ENSG00000223547 | ZNF844 | brown |
| ENSG00000223561 | AC005165.1 | brown |
| ENSG00000223571 | DHRSX-IT1 | brown |
| ENSG00000223704 | LINC01422 | brown |
| ENSG00000223745 | CCDC18-AS1 | brown |
| ENSG00000223750 | SIRPB3P | brown |
| ENSG00000223799 | IL10RB-AS1 | brown |
| ENSG00000223901 | AP001469.1 | brown |
| ENSG00000224046 | AC005076.1 | brown |
| ENSG00000224066 | AL049795.1 | brown |
| ENSG00000224086 | AC245452.1 | brown |
| ENSG00000224186 | C5orf66 | brown |
| ENSG00000224358 | AL451074.2 | brown |
| ENSG00000224505 | AC138150.1 | brown |
| ENSG00000224578 | HNRNPA1P48 | brown |
| ENSG00000224635 | AL391095.1 | brown |
| ENSG00000224660 | SH3BP5-AS1 | brown |
| ENSG00000224699 | LAMTOR5-AS1 | brown |
| ENSG00000224875 | AC083949.1 | brown |
| ENSG00000224888 | AC138028.2 | brown |
| ENSG00000224892 | RPS4XP16 | brown |
| ENSG00000224971 | SUMO2P3 | brown |
| ENSG00000224975 | INE1 | brown |
| ENSG00000224988 | AL158207.1 | brown |
| ENSG00000225032 | AL162586.1 | brown |

| | | |
|---|---|---|
| ENSG00000225094 | SETP20 | brown |
| ENSG00000225190 | PLEKHM1 | brown |
| ENSG00000225213 | AC073367.1 | brown |
| ENSG00000225313 | AL513327.1 | brown |
| ENSG00000225339 | AL354740.1 | brown |
| ENSG00000225377 | NRSN2-AS1 | brown |
| ENSG00000225422 | RBMS1P1 | brown |
| ENSG00000225460 | AL590233.1 | brown |
| ENSG00000225484 | NUTM2B-AS1 | brown |
| ENSG00000225526 | MKRN2OS | brown |
| ENSG00000225556 | C2CD4D | brown |
| ENSG00000225573 | RPL35P5 | brown |
| ENSG00000225580 | AL358942.1 | brown |
| ENSG00000225695 | HNRNPA1P35 | brown |
| ENSG00000225774 | SIRPAP1 | brown |
| ENSG00000225783 | MIAT | brown |
| ENSG00000225793 | AL080250.1 | brown |
| ENSG00000225806 | AL121917.1 | brown |
| ENSG00000225831 | RPS18P1 | brown |
| ENSG00000225850 | AL355490.1 | brown |
| ENSG00000225855 | RUSC1-AS1 | brown |
| ENSG00000225871 | AC245100.2 | brown |
| ENSG00000225891 | AL513365.2 | brown |
| ENSG00000225920 | RIMKLBP2 | brown |
| ENSG00000225948 | AL158210.1 | brown |
| ENSG00000225992 | TRGVA | brown |
| ENSG00000226002 | GTF2IP14 | brown |
| ENSG00000226012 | AP001434.1 | brown |
| ENSG00000226015 | CCT8P1 | brown |
| ENSG00000226029 | LINC01772 | brown |
| ENSG00000226055 | PAICSP1 | brown |
| ENSG00000226210 | AC215219.1 | brown |
| ENSG00000226266 | AC009961.1 | brown |
| ENSG00000226312 | CFLAR-AS1 | brown |
| ENSG00000226318 | RPS3AP38 | brown |
| ENSG00000226380 | AC016831.1 | brown |
| ENSG00000226419 | SLC16A1-AS1 | brown |
| ENSG00000226471 | Z93930.2 | brown |
| ENSG00000226491 | FTOP1 | brown |
| ENSG00000226699 | AL360181.1 | brown |
| ENSG00000226763 | SRRM5 | brown |
| ENSG00000226803 | AL136311.1 | brown |
| ENSG00000226891 | LINC01359 | brown |
| ENSG00000226945 | AC098935.1 | brown |
| ENSG00000226987 | AL157938.1 | brown |
| ENSG00000227028 | SLC8A1-AS1 | brown |
| ENSG00000227155 | AL161725.1 | brown |
| ENSG00000227212 | PFN1P6 | brown |

| | | |
|---|---|---|
| ENSG00000227252 | AC105760.2 | brown |
| ENSG00000227413 | AL022238.1 | brown |
| ENSG00000227486 | AL445472.1 | brown |
| ENSG00000227512 | AL592301.1 | brown |
| ENSG00000227536 | SOCS5P4 | brown |
| ENSG00000227543 | SPAG5-AS1 | brown |
| ENSG00000227598 | Z94721.1 | brown |
| ENSG00000227688 | HNRNPA3P2 | brown |
| ENSG00000227799 | AC012358.2 | brown |
| ENSG00000227920 | AL353597.1 | brown |
| ENSG00000228013 | AL162591.1 | brown |
| ENSG00000228028 | AC069257.1 | brown |
| ENSG00000228049 | POLR2J2 | brown |
| ENSG00000228107 | AP000692.1 | brown |
| ENSG00000228113 | AC003991.1 | brown |
| ENSG00000228137 | AP001469.2 | brown |
| ENSG00000228146 | CASP16P | brown |
| ENSG00000228168 | HNRNPA1P21 | brown |
| ENSG00000228192 | AL512353.1 | brown |
| ENSG00000228201 | AL022341.1 | brown |
| ENSG00000228218 | ATF4P3 | brown |
| ENSG00000228242 | AC093495.1 | brown |
| ENSG00000228251 | AC012442.1 | brown |
| ENSG00000228261 | AL162742.1 | brown |
| ENSG00000228274 | AL021707.2 | brown |
| ENSG00000228302 | AL512770.1 | brown |
| ENSG00000228323 | AC008440.1 | brown |
| ENSG00000228327 | AL669831.1 | brown |
| ENSG00000228328 | AL158201.1 | brown |
| ENSG00000228340 | MIR646HG | brown |
| ENSG00000228363 | AC015971.1 | brown |
| ENSG00000228395 | AL356481.1 | brown |
| ENSG00000228434 | AC004951.1 | brown |
| ENSG00000228436 | AL139260.1 | brown |
| ENSG00000228492 | RAB11FIP1P1 | brown |
| ENSG00000228532 | AC005000.1 | brown |
| ENSG00000228590 | AC007381.1 | brown |
| ENSG00000228606 | AL139011.1 | brown |
| ENSG00000228634 | AL136115.1 | brown |
| ENSG00000228638 | FCF1P2 | brown |
| ENSG00000228649 | SNHG26 | brown |
| ENSG00000228663 | PSMD10P1 | brown |
| ENSG00000228782 | MRPL45P2 | brown |
| ENSG00000228784 | LINC00954 | brown |
| ENSG00000228801 | AC064807.1 | brown |
| ENSG00000228804 | AC072022.2 | brown |
| ENSG00000228817 | BACH1-IT2 | brown |
| ENSG00000228852 | AC092802.2 | brown |

| | | |
|---|---|---|
| ENSG00000228863 | AL121985.1 | brown |
| ENSG00000228889 | UBAC2-AS1 | brown |
| ENSG00000228923 | AP000355.1 | brown |
| ENSG00000228925 | AC016722.2 | brown |
| ENSG00000229153 | EPHA1-AS1 | brown |
| ENSG00000229222 | KRT18P4 | brown |
| ENSG00000229325 | ACAP2-IT1 | brown |
| ENSG00000229334 | AC046143.1 | brown |
| ENSG00000229431 | AL139289.1 | brown |
| ENSG00000229447 | AC114495.2 | brown |
| ENSG00000229512 | AC068580.1 | brown |
| ENSG00000229539 | AL353194.1 | brown |
| ENSG00000229585 | AC110792.1 | brown |
| ENSG00000229591 | AC006017.1 | brown |
| ENSG00000229619 | MBNL1-AS1 | brown |
| ENSG00000229677 | AC018644.1 | brown |
| ENSG00000229728 | AL136531.1 | brown |
| ENSG00000229759 | MRPS18AP1 | brown |
| ENSG00000229848 | AC139149.1 | brown |
| ENSG00000229852 | AC019205.2 | brown |
| ENSG00000229873 | OGFR-AS1 | brown |
| ENSG00000229961 | AL357143.1 | brown |
| ENSG00000229990 | AC022400.2 | brown |
| ENSG00000230021 | AL669831.3 | brown |
| ENSG00000230042 | AK3P3 | brown |
| ENSG00000230068 | CDC42-IT1 | brown |
| ENSG00000230071 | RPL4P6 | brown |
| ENSG00000230074 | AL162231.2 | brown |
| ENSG00000230084 | AC006059.1 | brown |
| ENSG00000230155 | FO393401.1 | brown |
| ENSG00000230185 | C9orf147 | brown |
| ENSG00000230224 | PHBP9 | brown |
| ENSG00000230280 | HNRNPA1P59 | brown |
| ENSG00000230295 | GTF2IP23 | brown |
| ENSG00000230319 | AL022476.1 | brown |
| ENSG00000230322 | AL136320.1 | brown |
| ENSG00000230330 | HMGN2P3 | brown |
| ENSG00000230358 | SPDYE21P | brown |
| ENSG00000230415 | LINC01786 | brown |
| ENSG00000230424 | AL035413.1 | brown |
| ENSG00000230454 | U73166.1 | brown |
| ENSG00000230492 | AL049651.1 | brown |
| ENSG00000230510 | PPP5D1 | brown |
| ENSG00000230551 | AC021078.1 | brown |
| ENSG00000230587 | AC093609.1 | brown |
| ENSG00000230606 | AC092683.1 | brown |
| ENSG00000230623 | AC104461.1 | brown |
| ENSG00000230673 | PABPC1P3 | brown |

| | | |
|---|---|---|
| ENSG00000230701 | FBXW4P1 | brown |
| ENSG00000230732 | AC016949.1 | brown |
| ENSG00000230735 | AC093423.2 | brown |
| ENSG00000230839 | AL121760.1 | brown |
| ENSG00000230896 | AL604028.1 | brown |
| ENSG00000230912 | AL021707.4 | brown |
| ENSG00000230918 | AC008063.1 | brown |
| ENSG00000230953 | AC099677.1 | brown |
| ENSG00000230982 | DSTNP1 | brown |
| ENSG00000231010 | AL121672.1 | brown |
| ENSG00000231073 | AL590133.1 | brown |
| ENSG00000231102 | AL035588.1 | brown |
| ENSG00000231105 | AL031728.1 | brown |
| ENSG00000231123 | SPATA20P1 | brown |
| ENSG00000231125 | AF129075.1 | brown |
| ENSG00000231160 | KLF3-AS1 | brown |
| ENSG00000231329 | AL031772.1 | brown |
| ENSG00000231346 | LINC01160 | brown |
| ENSG00000231369 | Z97353.1 | brown |
| ENSG00000231412 | AC005392.2 | brown |
| ENSG00000231551 | AC245100.4 | brown |
| ENSG00000231587 | SNORD62B | brown |
| ENSG00000231731 | AC010976.1 | brown |
| ENSG00000231760 | AL355312.2 | brown |
| ENSG00000231789 | PIK3CD-AS2 | brown |
| ENSG00000231799 | PA2G4P6 | brown |
| ENSG00000231826 | LINC01819 | brown |
| ENSG00000231948 | HS1BP3-IT1 | brown |
| ENSG00000231964 | AL731567.1 | brown |
| ENSG00000231989 | PPP1R2P3 | brown |
| ENSG00000232043 | AL133230.1 | brown |
| ENSG00000232098 | AC012313.1 | brown |
| ENSG00000232133 | IMPDH1P10 | brown |
| ENSG00000232218 | AL021937.2 | brown |
| ENSG00000232254 | CSF2RBP1 | brown |
| ENSG00000232295 | AL589935.1 | brown |
| ENSG00000232334 | AL683842.1 | brown |
| ENSG00000232344 | AC087163.2 | brown |
| ENSG00000232354 | VIPR1-AS1 | brown |
| ENSG00000232439 | RPL18AP7 | brown |
| ENSG00000232450 | AL133517.1 | brown |
| ENSG00000232528 | AL109809.1 | brown |
| ENSG00000232545 | AC253536.3 | brown |
| ENSG00000232546 | AC027644.1 | brown |
| ENSG00000232611 | AL683813.1 | brown |
| ENSG00000232615 | AC026412.2 | brown |
| ENSG00000232680 | AC002511.1 | brown |
| ENSG00000232712 | KIZ-AS1 | brown |

| | | |
|---|---|---|
| ENSG00000232871 | SEC1P | brown |
| ENSG00000232909 | AL157823.2 | brown |
| ENSG00000232926 | AC000078.1 | brown |
| ENSG00000233006 | MIR3936HG | brown |
| ENSG00000233013 | FAM157B | brown |
| ENSG00000233056 | ERVH48-1 | brown |
| ENSG00000233122 | CTAGE7P | brown |
| ENSG00000233170 | AC138356.2 | brown |
| ENSG00000233175 | AC008105.1 | brown |
| ENSG00000233231 | HNRNPA1P49 | brown |
| ENSG00000233334 | FAM53B-AS1 | brown |
| ENSG00000233360 | Z83844.2 | brown |
| ENSG00000233382 | NKAPP1 | brown |
| ENSG00000233431 | AL359815.1 | brown |
| ENSG00000233436 | BTBD18 | brown |
| ENSG00000233478 | AL031280.1 | brown |
| ENSG00000233483 | AC008105.2 | brown |
| ENSG00000233559 | LINC00513 | brown |
| ENSG00000233578 | EIF4EP1 | brown |
| ENSG00000233614 | DDX11L10 | brown |
| ENSG00000233885 | YEATS2-AS1 | brown |
| ENSG00000234004 | AC092017.2 | brown |
| ENSG00000234174 | AC016683.1 | brown |
| ENSG00000234185 | AC068533.3 | brown |
| ENSG00000234219 | CDCA4P4 | brown |
| ENSG00000234263 | AL024508.1 | brown |
| ENSG00000234281 | LANCL1-AS1 | brown |
| ENSG00000234292 | AC123595.1 | brown |
| ENSG00000234327 | AC012146.1 | brown |
| ENSG00000234338 | AC073349.2 | brown |
| ENSG00000234456 | MAGI2-AS3 | brown |
| ENSG00000234494 | SP2-AS1 | brown |
| ENSG00000234616 | JRK | brown |
| ENSG00000234617 | SNRK-AS1 | brown |
| ENSG00000234630 | AC245060.2 | brown |
| ENSG00000234684 | SDCBP2-AS1 | brown |
| ENSG00000234750 | AC012618.2 | brown |
| ENSG00000234789 | AL590369.1 | brown |
| ENSG00000234902 | AC007879.3 | brown |
| ENSG00000234911 | TEX21P | brown |
| ENSG00000234945 | GTF3C2-AS1 | brown |
| ENSG00000235027 | AC068580.3 | brown |
| ENSG00000235079 | ZRANB2-AS1 | brown |
| ENSG00000235082 | SUMO1P3 | brown |
| ENSG00000235138 | AL445931.1 | brown |
| ENSG00000235159 | AL121672.2 | brown |
| ENSG00000235204 | AL162724.2 | brown |
| ENSG00000235245 | AL360181.2 | brown |

| | | |
|---|---|---|
| ENSG00000235308 | AL445991.1 | brown |
| ENSG00000235313 | HM13-IT1 | brown |
| ENSG00000235316 | DUSP8P5 | brown |
| ENSG00000235328 | AC006946.1 | brown |
| ENSG00000235351 | AC114730.3 | brown |
| ENSG00000235381 | AL596202.1 | brown |
| ENSG00000235408 | SNORA71B | brown |
| ENSG00000235419 | AC010149.1 | brown |
| ENSG00000235423 | AC068768.1 | brown |
| ENSG00000235475 | LINC01372 | brown |
| ENSG00000235499 | AC073046.1 | brown |
| ENSG00000235530 | AC087294.1 | brown |
| ENSG00000235579 | AC007283.2 | brown |
| ENSG00000235602 | POU5F1P3 | brown |
| ENSG00000235703 | LINC00894 | brown |
| ENSG00000235865 | GSN-AS1 | brown |
| ENSG00000235890 | TSPEAR-AS1 | brown |
| ENSG00000235908 | RHOA-IT1 | brown |
| ENSG00000235910 | APOA1-AS | brown |
| ENSG00000235944 | ZNF815P | brown |
| ENSG00000235961 | PNMA6A | brown |
| ENSG00000236015 | AC011290.2 | brown |
| ENSG00000236035 | AL513343.1 | brown |
| ENSG00000236194 | AC099811.1 | brown |
| ENSG00000236200 | KDM4A-AS1 | brown |
| ENSG00000236255 | AC009404.1 | brown |
| ENSG00000236337 | FMR1-IT1 | brown |
| ENSG00000236383 | LINC00854 | brown |
| ENSG00000236438 | FAM157A | brown |
| ENSG00000236496 | GPS2P1 | brown |
| ENSG00000236514 | AL135791.1 | brown |
| ENSG00000236525 | AC007278.2 | brown |
| ENSG00000236540 | AC006547.1 | brown |
| ENSG00000236559 | AL117381.1 | brown |
| ENSG00000236670 | KRT18P5 | brown |
| ENSG00000236753 | MKLN1-AS | brown |
| ENSG00000236772 | AL034550.1 | brown |
| ENSG00000236830 | CBR3-AS1 | brown |
| ENSG00000236861 | AC006378.1 | brown |
| ENSG00000236871 | LINC00106 | brown |
| ENSG00000236878 | MTATP6P26 | brown |
| ENSG00000236901 | MIR600HG | brown |
| ENSG00000236911 | AL137789.1 | brown |
| ENSG00000236953 | ZDHHC20-IT1 | brown |
| ENSG00000236977 | ANKRD44-IT1 | brown |
| ENSG00000237054 | PRMT5-AS1 | brown |
| ENSG00000237082 | COX5BP6 | brown |
| ENSG00000237094 | AL732372.2 | brown |

| | | |
|---|---|---|
| ENSG00000237118 | CYP2F2P | brown |
| ENSG00000237172 | B3GNT9 | brown |
| ENSG00000237188 | AC242426.3 | brown |
| ENSG00000237301 | AL121992.1 | brown |
| ENSG00000237356 | AL365295.1 | brown |
| ENSG00000237437 | ASS1P12 | brown |
| ENSG00000237493 | AC034102.1 | brown |
| ENSG00000237593 | AL445220.1 | brown |
| ENSG00000237637 | FRY-AS1 | brown |
| ENSG00000237672 | KRR1P1 | brown |
| ENSG00000237718 | AC009095.1 | brown |
| ENSG00000237719 | Z95152.1 | brown |
| ENSG00000237854 | LINC00674 | brown |
| ENSG00000237892 | KLF7-IT1 | brown |
| ENSG00000237914 | SIRPG-AS1 | brown |
| ENSG00000237938 | AL450998.3 | brown |
| ENSG00000237945 | LINC00649 | brown |
| ENSG00000238000 | AC116347.1 | brown |
| ENSG00000238009 | AL627309.1 | brown |
| ENSG00000238035 | AC138035.1 | brown |
| ENSG00000238058 | AL355574.1 | brown |
| ENSG00000238072 | AC009244.1 | brown |
| ENSG00000238152 | AC092865.3 | brown |
| ENSG00000238184 | CD81-AS1 | brown |
| ENSG00000238228 | OR7E7P | brown |
| ENSG00000238231 | AL512288.2 | brown |
| ENSG00000238278 | ALG1L6P | brown |
| ENSG00000238287 | AL603839.3 | brown |
| ENSG00000238829 | RNU7-45P | brown |
| ENSG00000239300 | AC080162.1 | brown |
| ENSG00000239377 | AC073111.1 | brown |
| ENSG00000239419 | RN7SL535P | brown |
| ENSG00000239480 | AC073517.1 | brown |
| ENSG00000239486 | AC091390.3 | brown |
| ENSG00000239521 | GATS | brown |
| ENSG00000239665 | AL157392.3 | brown |
| ENSG00000239670 | AL355864.2 | brown |
| ENSG00000239704 | CDRT4 | brown |
| ENSG00000239732 | TLR9 | brown |
| ENSG00000239763 | AC009120.1 | brown |
| ENSG00000239827 | SUGT1P3 | brown |
| ENSG00000239857 | GET4 | brown |
| ENSG00000239883 | PARGP1 | brown |
| ENSG00000239900 | ADSL | brown |
| ENSG00000239906 | AL627309.2 | brown |
| ENSG00000240041 | IGHJ4 | brown |
| ENSG00000240135 | AC114876.1 | brown |
| ENSG00000240137 | ERICH6-AS1 | brown |

| | | |
|---|---|---|
| ENSG00000240288 | GHRLOS | brown |
| ENSG00000240342 | AC026366.1 | brown |
| ENSG00000240449 | AC005586.1 | brown |
| ENSG00000240463 | RPS19P3 | brown |
| ENSG00000240487 | AC093010.1 | brown |
| ENSG00000240497 | AC092919.1 | brown |
| ENSG00000240498 | CDKN2B-AS1 | brown |
| ENSG00000240522 | RPL7AP10 | brown |
| ENSG00000240616 | RPS6P25 | brown |
| ENSG00000240710 | AL512306.3 | brown |
| ENSG00000240731 | AL139287.1 | brown |
| ENSG00000240877 | RN7SL521P | brown |
| ENSG00000241081 | AF111169.1 | brown |
| ENSG00000241170 | AP001992.1 | brown |
| ENSG00000241258 | CRCP | brown |
| ENSG00000241269 | AC093620.1 | brown |
| ENSG00000241288 | AC092902.2 | brown |
| ENSG00000241316 | SUCLG2-AS1 | brown |
| ENSG00000241360 | PDXP | brown |
| ENSG00000241464 | RPL39P38 | brown |
| ENSG00000241489 | AC244197.3 | brown |
| ENSG00000241511 | AC026979.1 | brown |
| ENSG00000241527 | CA15P1 | brown |
| ENSG00000241547 | ACTG1P20 | brown |
| ENSG00000241769 | LINC00893 | brown |
| ENSG00000241860 | AL627309.5 | brown |
| ENSG00000241878 | PISD | brown |
| ENSG00000241886 | AC112496.1 | brown |
| ENSG00000241983 | RN7SL566P | brown |
| ENSG00000242028 | HYPK | brown |
| ENSG00000242154 | AC004884.2 | brown |
| ENSG00000242294 | STAG3L5P | brown |
| ENSG00000242574 | HLA-DMB | brown |
| ENSG00000242588 | AC108010.1 | brown |
| ENSG00000242607 | AC068587.1 | brown |
| ENSG00000242622 | AC092910.3 | brown |
| ENSG00000242797 | GLYCTK-AS1 | brown |
| ENSG00000242798 | AC073842.2 | brown |
| ENSG00000243066 | RN7SL842P | brown |
| ENSG00000243147 | MRPL33 | brown |
| ENSG00000243156 | MICAL3 | brown |
| ENSG00000243176 | AC092944.1 | brown |
| ENSG00000243224 | AC006252.1 | brown |
| ENSG00000243284 | VSIG8 | brown |
| ENSG00000243289 | AGAP13P | brown |
| ENSG00000243302 | AC018638.4 | brown |
| ENSG00000243323 | PTPRVP | brown |
| ENSG00000243406 | MRPS31P5 | brown |

| | | |
|---|---|---|
| ENSG00000243431 | RPL5P30 | brown |
| ENSG00000243488 | RN7SL337P | brown |
| ENSG00000243544 | RN7SL172P | brown |
| ENSG00000243554 | AC004967.1 | brown |
| ENSG00000243607 | AP001318.1 | brown |
| ENSG00000243680 | RPL37P23 | brown |
| ENSG00000243708 | PLA2G4B | brown |
| ENSG00000243789 | JMJD7 | brown |
| ENSG00000243797 | AC004917.1 | brown |
| ENSG00000243896 | OR2A7 | brown |
| ENSG00000243910 | TUBA4B | brown |
| ENSG00000243927 | MRPS6 | brown |
| ENSG00000243943 | ZNF512 | brown |
| ENSG00000243959 | RN7SL684P | brown |
| ENSG00000244055 | AC007566.1 | brown |
| ENSG00000244071 | RPL9P33 | brown |
| ENSG00000244124 | ATP1B3-AS1 | brown |
| ENSG00000244134 | RPS12P20 | brown |
| ENSG00000244171 | PBX2P1 | brown |
| ENSG00000244300 | GATA2-AS1 | brown |
| ENSG00000244357 | RN7SL145P | brown |
| ENSG00000244361 | RPL30P7 | brown |
| ENSG00000244459 | AC147067.1 | brown |
| ENSG00000244480 | AC005154.3 | brown |
| ENSG00000244490 | RWDD4P1 | brown |
| ENSG00000244515 | KRT18P34 | brown |
| ENSG00000244560 | AC004890.2 | brown |
| ENSG00000244627 | Z98749.2 | brown |
| ENSG00000244687 | UBE2V1 | brown |
| ENSG00000244723 | ASLP1 | brown |
| ENSG00000244879 | GABPB1-AS1 | brown |
| ENSG00000244932 | AL449212.1 | brown |
| ENSG00000245105 | A2M-AS1 | brown |
| ENSG00000245148 | ARAP1-AS2 | brown |
| ENSG00000245149 | RNF139-AS1 | brown |
| ENSG00000245275 | SAP30L-AS1 | brown |
| ENSG00000245466 | AL357153.1 | brown |
| ENSG00000245571 | AP001258.1 | brown |
| ENSG00000245598 | DACT3-AS1 | brown |
| ENSG00000245667 | AC006064.1 | brown |
| ENSG00000245748 | AC097382.2 | brown |
| ENSG00000246016 | LINC01513 | brown |
| ENSG00000246067 | RAB30-AS1 | brown |
| ENSG00000246130 | AC107959.2 | brown |
| ENSG00000246203 | AL353807.3 | brown |
| ENSG00000246273 | SBF2-AS1 | brown |
| ENSG00000246308 | AC116535.1 | brown |
| ENSG00000246334 | PRR7-AS1 | brown |

| | | |
|---|---|---|
| ENSG00000246339 | EXTL3-AS1 | brown |
| ENSG00000246363 | LINC02458 | brown |
| ENSG00000246451 | AL049840.1 | brown |
| ENSG00000246477 | AF131216.1 | brown |
| ENSG00000246575 | AC093162.2 | brown |
| ENSG00000246596 | AC139795.1 | brown |
| ENSG00000246790 | AP000977.1 | brown |
| ENSG00000246922 | UBAP1L | brown |
| ENSG00000246982 | Z84485.1 | brown |
| ENSG00000247137 | AP000873.2 | brown |
| ENSG00000247199 | AC091948.1 | brown |
| ENSG00000247287 | AL359220.1 | brown |
| ENSG00000247595 | SPTY2D1-AS1 | brown |
| ENSG00000247746 | USP51 | brown |
| ENSG00000247853 | AC006064.2 | brown |
| ENSG00000248015 | AC005329.1 | brown |
| ENSG00000248098 | BCKDHA | brown |
| ENSG00000248213 | CICP16 | brown |
| ENSG00000248323 | LUCAT1 | brown |
| ENSG00000248469 | AC139491.1 | brown |
| ENSG00000248476 | BACH1-IT1 | brown |
| ENSG00000248483 | POU5F2 | brown |
| ENSG00000248544 | AC008676.1 | brown |
| ENSG00000248559 | AC109454.2 | brown |
| ENSG00000248571 | AC106882.1 | brown |
| ENSG00000248643 | RBM14-RBM4 | brown |
| ENSG00000248714 | AC091180.2 | brown |
| ENSG00000248727 | LINC01948 | brown |
| ENSG00000248734 | AC008906.1 | brown |
| ENSG00000248774 | AC097534.1 | brown |
| ENSG00000248791 | AC010627.1 | brown |
| ENSG00000249014 | HMGN2P4 | brown |
| ENSG00000249031 | SUMO2P6 | brown |
| ENSG00000249096 | LINC02362 | brown |
| ENSG00000249160 | LINC02213 | brown |
| ENSG00000249207 | AC079921.1 | brown |
| ENSG00000249307 | LINC01088 | brown |
| ENSG00000249348 | UGDH-AS1 | brown |
| ENSG00000249353 | NPM1P27 | brown |
| ENSG00000249396 | LINC02212 | brown |
| ENSG00000249592 | AC139887.2 | brown |
| ENSG00000249685 | AC079921.2 | brown |
| ENSG00000249700 | SRD5A3-AS1 | brown |
| ENSG00000249709 | ZNF564 | brown |
| ENSG00000249741 | AC093890.1 | brown |
| ENSG00000249786 | EAF1-AS1 | brown |
| ENSG00000249825 | CTD-2201I18.1 | brown |
| ENSG00000249835 | VCAN-AS1 | brown |

| | | |
|---|---|---|
| ENSG00000249839 | AC011330.1 | brown |
| ENSG00000249850 | KRT18P31 | brown |
| ENSG00000249863 | AC021106.1 | brown |
| ENSG00000249898 | MCPH1-AS1 | brown |
| ENSG00000249947 | XBP1P1 | brown |
| ENSG00000250031 | AC009927.1 | brown |
| ENSG00000250073 | AP000866.2 | brown |
| ENSG00000250116 | AC018682.1 | brown |
| ENSG00000250130 | AC090519.1 | brown |
| ENSG00000250365 | AL139353.2 | brown |
| ENSG00000250471 | GMPSP1 | brown |
| ENSG00000250568 | AC098591.2 | brown |
| ENSG00000250569 | NTAN1P2 | brown |
| ENSG00000250659 | AP001363.1 | brown |
| ENSG00000250751 | AC015795.2 | brown |
| ENSG00000250848 | AC021087.2 | brown |
| ENSG00000250903 | GMDS-AS1 | brown |
| ENSG00000250909 | AC138956.1 | brown |
| ENSG00000250948 | AC091180.4 | brown |
| ENSG00000250959 | GLUD1P3 | brown |
| ENSG00000250990 | AC098851.1 | brown |
| ENSG00000250995 | AL391280.1 | brown |
| ENSG00000251139 | AC084871.1 | brown |
| ENSG00000251143 | AP002490.1 | brown |
| ENSG00000251194 | AL133330.1 | brown |
| ENSG00000251215 | GOLGA5P1 | brown |
| ENSG00000251229 | AL645924.2 | brown |
| ENSG00000251287 | ALG1L2 | brown |
| ENSG00000251288 | AC018797.3 | brown |
| ENSG00000251364 | AC107884.1 | brown |
| ENSG00000251393 | AC005280.1 | brown |
| ENSG00000251417 | AC145285.2 | brown |
| ENSG00000251441 | RTEL1P1 | brown |
| ENSG00000251453 | HAUS1P1 | brown |
| ENSG00000251474 | RPL32P3 | brown |
| ENSG00000251562 | MALAT1 | brown |
| ENSG00000251661 | AC136475.1 | brown |
| ENSG00000251666 | ZNF346-IT1 | brown |
| ENSG00000251675 | AC010260.1 | brown |
| ENSG00000251988 | RNU4ATAC18P | brown |
| ENSG00000252311 | RNU1-103P | brown |
| ENSG00000252391 | RNU6-638P | brown |
| ENSG00000252835 | SCARNA21 | brown |
| ENSG00000253005 | RNU6-176P | brown |
| ENSG00000253106 | AC090198.1 | brown |
| ENSG00000253167 | WASHC5-AS1 | brown |
| ENSG00000253200 | AC037459.3 | brown |
| ENSG00000253309 | SERPINE3 | brown |

| | | |
|---|---|---|
| ENSG00000253320 | AZIN1-AS1 | brown |
| ENSG00000253328 | SUMO2P19 | brown |
| ENSG00000253438 | PCAT1 | brown |
| ENSG00000253540 | FAM86HP | brown |
| ENSG00000253636 | AC022893.1 | brown |
| ENSG00000253649 | PRSS51 | brown |
| ENSG00000253720 | AC022973.2 | brown |
| ENSG00000253731 | PCDHGA6 | brown |
| ENSG00000253854 | AC010834.3 | brown |
| ENSG00000253921 | AC091982.2 | brown |
| ENSG00000253948 | AC104986.2 | brown |
| ENSG00000253981 | ALG1L13P | brown |
| ENSG00000254030 | IGLC5 | brown |
| ENSG00000254064 | AC105206.2 | brown |
| ENSG00000254165 | AC090739.1 | brown |
| ENSG00000254263 | AC022973.3 | brown |
| ENSG00000254281 | AP003354.2 | brown |
| ENSG00000254325 | AC018607.1 | brown |
| ENSG00000254362 | AC011726.3 | brown |
| ENSG00000254413 | CHKB-CPT1B | brown |
| ENSG00000254461 | AP001107.4 | brown |
| ENSG00000254463 | AC087276.1 | brown |
| ENSG00000254469 | AP002495.1 | brown |
| ENSG00000254477 | AP000640.1 | brown |
| ENSG00000254595 | AC084337.1 | brown |
| ENSG00000254634 | SMG1P6 | brown |
| ENSG00000254648 | AP000911.3 | brown |
| ENSG00000254760 | AC008750.1 | brown |
| ENSG00000254826 | AP001922.3 | brown |
| ENSG00000254860 | TMEM9B-AS1 | brown |
| ENSG00000254901 | BORCS8 | brown |
| ENSG00000254964 | AP001458.1 | brown |
| ENSG00000254995 | STX16-NPEPL1 | brown |
| ENSG00000254996 | ANKHD1-EIF4EBP3 | brown |
| ENSG00000255026 | AC136475.3 | brown |
| ENSG00000255031 | AP002807.1 | brown |
| ENSG00000255092 | AC010768.2 | brown |
| ENSG00000255142 | AP006621.2 | brown |
| ENSG00000255163 | HSPE1P18 | brown |
| ENSG00000255186 | AC087277.2 | brown |
| ENSG00000255197 | AC090559.1 | brown |
| ENSG00000255200 | PGAM1P8 | brown |
| ENSG00000255222 | SETP17 | brown |
| ENSG00000255299 | AP003557.1 | brown |
| ENSG00000255306 | AC004923.4 | brown |
| ENSG00000255310 | AF131215.5 | brown |
| ENSG00000255423 | EBLN2 | brown |
| ENSG00000255438 | AL354813.1 | brown |

| | | |
|---|---|---|
| ENSG00000255441 | AC008750.2 | brown |
| ENSG00000255495 | AC145124.1 | brown |
| ENSG00000255507 | AP003031.2 | brown |
| ENSG00000255524 | NPIPB8 | brown |
| ENSG00000255538 | OR10V2P | brown |
| ENSG00000255557 | AP001266.2 | brown |
| ENSG00000255568 | BRWD1-AS2 | brown |
| ENSG00000255582 | OR10G2 | brown |
| ENSG00000255585 | AL590627.1 | brown |
| ENSG00000255741 | AP000808.2 | brown |
| ENSG00000255801 | AC092746.1 | brown |
| ENSG00000255838 | AC069262.1 | brown |
| ENSG00000255857 | PXN-AS1 | brown |
| ENSG00000255883 | FUNDC2P1 | brown |
| ENSG00000255974 | CYP2A6 | brown |
| ENSG00000255987 | TOMM20P2 | brown |
| ENSG00000256139 | AC007637.1 | brown |
| ENSG00000256235 | SMIM3 | brown |
| ENSG00000256280 | AP006289.1 | brown |
| ENSG00000256357 | AL132708.1 | brown |
| ENSG00000256591 | AP003108.2 | brown |
| ENSG00000256705 | AL137779.1 | brown |
| ENSG00000256706 | AC005342.2 | brown |
| ENSG00000256742 | AC145422.1 | brown |
| ENSG00000256751 | PLBD1-AS1 | brown |
| ENSG00000256967 | AC018653.3 | brown |
| ENSG00000256973 | AC053513.1 | brown |
| ENSG00000256987 | AC023050.4 | brown |
| ENSG00000257094 | AC016957.1 | brown |
| ENSG00000257176 | AC009318.1 | brown |
| ENSG00000257221 | AC007569.1 | brown |
| ENSG00000257270 | AL928654.3 | brown |
| ENSG00000257337 | AC068888.1 | brown |
| ENSG00000257354 | AC048341.2 | brown |
| ENSG00000257446 | ZNF878 | brown |
| ENSG00000257489 | AC010203.1 | brown |
| ENSG00000257497 | AC121761.1 | brown |
| ENSG00000257509 | AC073487.1 | brown |
| ENSG00000257696 | AC010203.2 | brown |
| ENSG00000257698 | AC084033.3 | brown |
| ENSG00000257953 | AC083805.2 | brown |
| ENSG00000258082 | AL391832.3 | brown |
| ENSG00000258086 | AC079313.1 | brown |
| ENSG00000258102 | MAP1LC3B2 | brown |
| ENSG00000258172 | AC073655.1 | brown |
| ENSG00000258227 | CLEC5A | brown |
| ENSG00000258317 | AC034102.6 | brown |
| ENSG00000258344 | AC078778.2 | brown |

| | | |
|---|---|---|
| ENSG00000258365 | AC073655.2 | brown |
| ENSG00000258366 | RTEL1 | brown |
| ENSG00000258384 | AC068831.3 | brown |
| ENSG00000258424 | AL512791.1 | brown |
| ENSG00000258439 | AC007956.1 | brown |
| ENSG00000258449 | AC023510.1 | brown |
| ENSG00000258461 | AC012651.1 | brown |
| ENSG00000258484 | SPESP1 | brown |
| ENSG00000258515 | AL355075.2 | brown |
| ENSG00000258539 | AC068896.1 | brown |
| ENSG00000258546 | CENPUP2 | brown |
| ENSG00000258559 | AC005519.2 | brown |
| ENSG00000258568 | RHOQP1 | brown |
| ENSG00000258682 | AL132989.1 | brown |
| ENSG00000258701 | LINC00638 | brown |
| ENSG00000258704 | SRP54-AS1 | brown |
| ENSG00000258760 | AL355076.2 | brown |
| ENSG00000258794 | DUX4L27 | brown |
| ENSG00000258831 | AC103996.2 | brown |
| ENSG00000258904 | AL157871.5 | brown |
| ENSG00000258959 | AL118558.1 | brown |
| ENSG00000259020 | AL049872.1 | brown |
| ENSG00000259022 | DNAJC8P1 | brown |
| ENSG00000259075 | POC1B-GALNT4 | brown |
| ENSG00000259116 | AL049869.3 | brown |
| ENSG00000259118 | AL139022.1 | brown |
| ENSG00000259120 | SMIM6 | brown |
| ENSG00000259169 | GNRHR2P1 | brown |
| ENSG00000259212 | AC103739.1 | brown |
| ENSG00000259287 | AC010809.1 | brown |
| ENSG00000259307 | PLCB2-AS1 | brown |
| ENSG00000259326 | AC116158.1 | brown |
| ENSG00000259351 | AC015914.1 | brown |
| ENSG00000259366 | AC108449.2 | brown |
| ENSG00000259379 | MTND5P32 | brown |
| ENSG00000259407 | AC021739.2 | brown |
| ENSG00000259442 | AC105339.2 | brown |
| ENSG00000259488 | AC023355.1 | brown |
| ENSG00000259514 | AC027243.1 | brown |
| ENSG00000259548 | AC021483.1 | brown |
| ENSG00000259556 | AC090971.3 | brown |
| ENSG00000259562 | AC090607.2 | brown |
| ENSG00000259635 | AC100830.1 | brown |
| ENSG00000259659 | AC009996.1 | brown |
| ENSG00000259660 | DNM1P47 | brown |
| ENSG00000259687 | LINC01220 | brown |
| ENSG00000259703 | LINC00593 | brown |
| ENSG00000259717 | LINC00677 | brown |

| | | |
|---|---|---|
| ENSG00000259792 | AC104758.2 | brown |
| ENSG00000259804 | AC027682.1 | brown |
| ENSG00000259823 | LYPD8 | brown |
| ENSG00000259826 | AC072061.1 | brown |
| ENSG00000259856 | RAB43P1 | brown |
| ENSG00000259863 | SH3RF3-AS1 | brown |
| ENSG00000259881 | AC092384.2 | brown |
| ENSG00000259891 | AC107375.1 | brown |
| ENSG00000259922 | AC023825.1 | brown |
| ENSG00000259924 | AC011939.1 | brown |
| ENSG00000259940 | AC109449.1 | brown |
| ENSG00000259954 | IL21R-AS1 | brown |
| ENSG00000259972 | AC009120.2 | brown |
| ENSG00000259984 | AL928711.1 | brown |
| ENSG00000259985 | AC017100.1 | brown |
| ENSG00000260022 | AL031716.1 | brown |
| ENSG00000260034 | LCMT1-AS2 | brown |
| ENSG00000260051 | AL031600.1 | brown |
| ENSG00000260059 | AC092620.3 | brown |
| ENSG00000260097 | SPDYE6 | brown |
| ENSG00000260114 | AC120114.2 | brown |
| ENSG00000260121 | AC138028.4 | brown |
| ENSG00000260145 | AC023825.2 | brown |
| ENSG00000260190 | AL807752.5 | brown |
| ENSG00000260233 | SSSCA1-AS1 | brown |
| ENSG00000260249 | AC007608.3 | brown |
| ENSG00000260257 | AL035071.1 | brown |
| ENSG00000260261 | AC124944.3 | brown |
| ENSG00000260267 | AC026471.1 | brown |
| ENSG00000260279 | AC137932.1 | brown |
| ENSG00000260280 | SLX1B-SULT1A4 | brown |
| ENSG00000260286 | C6orf229 | brown |
| ENSG00000260293 | AC106820.4 | brown |
| ENSG00000260325 | HSPB9 | brown |
| ENSG00000260378 | AC109597.1 | brown |
| ENSG00000260417 | AC092127.1 | brown |
| ENSG00000260425 | AL031709.1 | brown |
| ENSG00000260448 | LCMT1-AS1 | brown |
| ENSG00000260452 | TPRKBP2 | brown |
| ENSG00000260467 | AC018552.2 | brown |
| ENSG00000260487 | AC116348.1 | brown |
| ENSG00000260507 | AC133919.1 | brown |
| ENSG00000260528 | FAM157C | brown |
| ENSG00000260549 | MT1L | brown |
| ENSG00000260565 | ERVK13-1 | brown |
| ENSG00000260618 | AC025917.1 | brown |
| ENSG00000260671 | AC010536.2 | brown |
| ENSG00000260719 | AC009133.3 | brown |

| | | |
|---|---|---|
| ENSG00000260727 | SLC7A5P1 | brown |
| ENSG00000260729 | AC009690.1 | brown |
| ENSG00000260751 | AC008870.2 | brown |
| ENSG00000260852 | FBXL19-AS1 | brown |
| ENSG00000260853 | AC109460.2 | brown |
| ENSG00000260884 | AC009120.4 | brown |
| ENSG00000260898 | ADPGK-AS1 | brown |
| ENSG00000260953 | AC009093.4 | brown |
| ENSG00000261096 | AC073476.3 | brown |
| ENSG00000261097 | LINC00563 | brown |
| ENSG00000261115 | TMEM178B | brown |
| ENSG00000261118 | AC092123.1 | brown |
| ENSG00000261136 | AC023908.3 | brown |
| ENSG00000261140 | AC093525.4 | brown |
| ENSG00000261158 | AC109597.2 | brown |
| ENSG00000261207 | AL031717.1 | brown |
| ENSG00000261245 | AC093520.2 | brown |
| ENSG00000261267 | AC026470.3 | brown |
| ENSG00000261270 | AC012181.3 | brown |
| ENSG00000261373 | VPS9D1-AS1 | brown |
| ENSG00000261442 | AC023830.1 | brown |
| ENSG00000261451 | AC104964.2 | brown |
| ENSG00000261460 | AC009690.2 | brown |
| ENSG00000261471 | AC092145.1 | brown |
| ENSG00000261505 | AL031714.1 | brown |
| ENSG00000261546 | AC135782.3 | brown |
| ENSG00000261556 | SMG1P7 | brown |
| ENSG00000261582 | AL121753.1 | brown |
| ENSG00000261592 | AC010531.3 | brown |
| ENSG00000261654 | AL360270.2 | brown |
| ENSG00000261656 | BEAN1-AS1 | brown |
| ENSG00000261740 | BOLA2-SMG1P6 | brown |
| ENSG00000261744 | AC116552.1 | brown |
| ENSG00000261766 | AC133550.2 | brown |
| ENSG00000261770 | AC006504.1 | brown |
| ENSG00000261786 | AC006058.1 | brown |
| ENSG00000261840 | AC093249.6 | brown |
| ENSG00000261888 | AC144831.1 | brown |
| ENSG00000261971 | MMP25-AS1 | brown |
| ENSG00000261996 | AC004706.1 | brown |
| ENSG00000262180 | OCLM | brown |
| ENSG00000262227 | AC004771.3 | brown |
| ENSG00000262312 | AC004494.1 | brown |
| ENSG00000262319 | AC007952.6 | brown |
| ENSG00000262362 | AC004233.1 | brown |
| ENSG00000262488 | AC133065.3 | brown |
| ENSG00000262663 | AC087222.1 | brown |
| ENSG00000262678 | AC004771.5 | brown |

| | | |
|---|---|---|
| ENSG00000262766 | AC135050.5 | brown |
| ENSG00000262874 | C19orf84 | brown |
| ENSG00000262884 | AC015921.1 | brown |
| ENSG00000262999 | AC099489.3 | brown |
| ENSG00000263013 | AC133065.4 | brown |
| ENSG00000263069 | AC124319.2 | brown |
| ENSG00000263235 | AC006111.2 | brown |
| ENSG00000263264 | AC119396.1 | brown |
| ENSG00000263327 | TAPT1-AS1 | brown |
| ENSG00000263345 | AC006435.2 | brown |
| ENSG00000263370 | AC104564.2 | brown |
| ENSG00000263466 | AC006441.1 | brown |
| ENSG00000263531 | AC130324.1 | brown |
| ENSG00000263563 | UBBP4 | brown |
| ENSG00000263624 | AC055811.1 | brown |
| ENSG00000263667 | AL035696.3 | brown |
| ENSG00000263740 | RN7SL4P | brown |
| ENSG00000263786 | AC022211.1 | brown |
| ENSG00000263818 | AC091178.1 | brown |
| ENSG00000263826 | AC112907.3 | brown |
| ENSG00000263847 | AP005899.1 | brown |
| ENSG00000263884 | AP000845.1 | brown |
| ENSG00000263934 | SNORD3A | brown |
| ENSG00000263968 | RN7SL381P | brown |
| ENSG00000264112 | AC015813.1 | brown |
| ENSG00000264188 | AC106037.1 | brown |
| ENSG00000264290 | AC104564.4 | brown |
| ENSG00000264304 | AC024267.3 | brown |
| ENSG00000264343 | NOTCH2NL | brown |
| ENSG00000264357 | MIR4648 | brown |
| ENSG00000264364 | DYNLL2 | brown |
| ENSG00000264384 | RN7SL431P | brown |
| ENSG00000264386 | MIR4513 | brown |
| ENSG00000264456 | AC138207.4 | brown |
| ENSG00000264520 | AC005154.5 | brown |
| ENSG00000264538 | SUZ12P1 | brown |
| ENSG00000264553 | MIR4257 | brown |
| ENSG00000264577 | AC010761.1 | brown |
| ENSG00000264608 | AC005726.4 | brown |
| ENSG00000264743 | DPRXP4 | brown |
| ENSG00000264769 | AC145207.8 | brown |
| ENSG00000264773 | MIR4420 | brown |
| ENSG00000264853 | AC011933.2 | brown |
| ENSG00000264937 | AC100830.2 | brown |
| ENSG00000265194 | AL359922.2 | brown |
| ENSG00000265195 | MIR4312 | brown |
| ENSG00000265205 | AC010761.3 | brown |
| ENSG00000265241 | RBM8A | brown |

| | | |
|---|---|---|
| ENSG00000265293 | ARGFXP2 | brown |
| ENSG00000265399 | AP005329.2 | brown |
| ENSG00000265479 | DTX2P1-UPK3BP1 | brown |
| ENSG00000265625 | AC104564.6 | brown |
| ENSG00000265656 | AC106037.2 | brown |
| ENSG00000265658 | MIR3690 | brown |
| ENSG00000265678 | AC129510.1 | brown |
| ENSG00000265735 | RN7SL5P | brown |
| ENSG00000265749 | AC135178.3 | brown |
| ENSG00000265778 | AC018413.1 | brown |
| ENSG00000265791 | AC127024.4 | brown |
| ENSG00000265800 | AC022211.3 | brown |
| ENSG00000265806 | MIR4292 | brown |
| ENSG00000265845 | AC024267.4 | brown |
| ENSG00000265908 | AC024267.5 | brown |
| ENSG00000265996 | MIR3671 | brown |
| ENSG00000266124 | MIR5587 | brown |
| ENSG00000266126 | AC005730.3 | brown |
| ENSG00000266173 | STRADA | brown |
| ENSG00000266235 | MIR3176 | brown |
| ENSG00000266236 | NARF-IT1 | brown |
| ENSG00000266338 | NBPF15 | brown |
| ENSG00000266445 | AC124287.1 | brown |
| ENSG00000266490 | AC127024.5 | brown |
| ENSG00000266677 | AC087164.1 | brown |
| ENSG00000266707 | AC006120.1 | brown |
| ENSG00000266718 | AC079336.5 | brown |
| ENSG00000266733 | TBC1D29 | brown |
| ENSG00000266824 | AC129492.4 | brown |
| ENSG00000266913 | LINC01841 | brown |
| ENSG00000266934 | AC005746.1 | brown |
| ENSG00000266983 | AC011444.1 | brown |
| ENSG00000266993 | AL050343.1 | brown |
| ENSG00000267016 | AC111170.1 | brown |
| ENSG00000267033 | AC020911.1 | brown |
| ENSG00000267041 | ZNF850 | brown |
| ENSG00000267046 | E2F3P1 | brown |
| ENSG00000267074 | AC015911.3 | brown |
| ENSG00000267096 | AC008735.1 | brown |
| ENSG00000267136 | AP005131.1 | brown |
| ENSG00000267212 | AC018761.3 | brown |
| ENSG00000267244 | AC012615.6 | brown |
| ENSG00000267262 | AC011444.2 | brown |
| ENSG00000267274 | AC008770.4 | brown |
| ENSG00000267278 | MAP3K14-AS1 | brown |
| ENSG00000267293 | AC012569.1 | brown |
| ENSG00000267348 | AC011489.1 | brown |
| ENSG00000267385 | AC011498.4 | brown |

| | | |
|---|---|---|
| ENSG00000267394 | AC004596.1 | brown |
| ENSG00000267474 | AC008569.2 | brown |
| ENSG00000267481 | AC011477.2 | brown |
| ENSG00000267491 | AC100788.1 | brown |
| ENSG00000267493 | CIRBP-AS1 | brown |
| ENSG00000267523 | AC008735.2 | brown |
| ENSG00000267546 | AC015802.4 | brown |
| ENSG00000267551 | AC005264.1 | brown |
| ENSG00000267554 | AC015911.8 | brown |
| ENSG00000267571 | AC104532.2 | brown |
| ENSG00000267586 | LINC00907 | brown |
| ENSG00000267595 | AC060780.3 | brown |
| ENSG00000267632 | AC067852.6 | brown |
| ENSG00000267654 | LINC01882 | brown |
| ENSG00000267655 | AC125437.1 | brown |
| ENSG00000267702 | AP005131.6 | brown |
| ENSG00000267834 | AL592211.1 | brown |
| ENSG00000267895 | AC063977.1 | brown |
| ENSG00000267904 | AC024075.1 | brown |
| ENSG00000267934 | AC010300.1 | brown |
| ENSG00000267990 | AC063977.2 | brown |
| ENSG00000268030 | AC005253.1 | brown |
| ENSG00000268047 | AC018766.1 | brown |
| ENSG00000268049 | AC012313.2 | brown |
| ENSG00000268069 | AC004466.1 | brown |
| ENSG00000268087 | AC008764.2 | brown |
| ENSG00000268093 | AC022154.1 | brown |
| ENSG00000268201 | AC020915.3 | brown |
| ENSG00000268218 | AC137932.3 | brown |
| ENSG00000268366 | AC010271.1 | brown |
| ENSG00000268516 | AC020915.4 | brown |
| ENSG00000268555 | AC123912.4 | brown |
| ENSG00000268568 | AC007228.2 | brown |
| ENSG00000268573 | AC011815.1 | brown |
| ENSG00000268575 | AL031282.2 | brown |
| ENSG00000268583 | AC011466.1 | brown |
| ENSG00000268618 | AC092316.1 | brown |
| ENSG00000268670 | AC016586.1 | brown |
| ENSG00000268713 | AC005261.4 | brown |
| ENSG00000268743 | AC008737.1 | brown |
| ENSG00000268833 | AC243967.2 | brown |
| ENSG00000268849 | SIGLEC22P | brown |
| ENSG00000268947 | AC002128.1 | brown |
| ENSG00000269028 | MTRNR2L12 | brown |
| ENSG00000269038 | AP001462.1 | brown |
| ENSG00000269044 | AC024075.2 | brown |
| ENSG00000269086 | AC008555.2 | brown |
| ENSG00000269226 | TMSB15B | brown |

| | | |
|---|---|---|
| ENSG00000269243 | AC008894.2 | brown |
| ENSG00000269318 | AC007292.2 | brown |
| ENSG00000269335 | IKBKG | brown |
| ENSG00000269343 | ZNF587B | brown |
| ENSG00000269427 | AC024075.3 | brown |
| ENSG00000269473 | AC012313.8 | brown |
| ENSG00000269487 | AC008635.1 | brown |
| ENSG00000269514 | AC024257.3 | brown |
| ENSG00000269553 | U62631.1 | brown |
| ENSG00000269564 | AC008753.2 | brown |
| ENSG00000269680 | AC008760.1 | brown |
| ENSG00000269737 | AL691432.1 | brown |
| ENSG00000269815 | AC010463.3 | brown |
| ENSG00000269821 | KCNQ1OT1 | brown |
| ENSG00000269825 | AC022150.4 | brown |
| ENSG00000269843 | AC008537.2 | brown |
| ENSG00000269899 | AC025857.2 | brown |
| ENSG00000269903 | AC025165.4 | brown |
| ENSG00000269918 | AF131215.6 | brown |
| ENSG00000269925 | Z98884.2 | brown |
| ENSG00000269937 | AC093525.7 | brown |
| ENSG00000269956 | MKNK1-AS1 | brown |
| ENSG00000269967 | AL136115.2 | brown |
| ENSG00000269984 | AC078795.1 | brown |
| ENSG00000270001 | AL121894.2 | brown |
| ENSG00000270015 | AC087481.3 | brown |
| ENSG00000270019 | AC110769.2 | brown |
| ENSG00000270020 | AC009108.3 | brown |
| ENSG00000270049 | AC009061.2 | brown |
| ENSG00000270060 | AC090589.3 | brown |
| ENSG00000270105 | AC136475.8 | brown |
| ENSG00000270108 | AL049840.5 | brown |
| ENSG00000270120 | AC007728.3 | brown |
| ENSG00000270149 | AL591806.3 | brown |
| ENSG00000270165 | AC010530.1 | brown |
| ENSG00000270168 | AC004233.2 | brown |
| ENSG00000270177 | AC104109.2 | brown |
| ENSG00000270184 | AC018695.4 | brown |
| ENSG00000270231 | NBPF8 | brown |
| ENSG00000270264 | AC004686.1 | brown |
| ENSG00000270392 | PFN1P2 | brown |
| ENSG00000270427 | NRBF2P5 | brown |
| ENSG00000270433 | AL049829.3 | brown |
| ENSG00000270614 | AC011451.2 | brown |
| ENSG00000270640 | AC104695.3 | brown |
| ENSG00000270681 | AC095055.1 | brown |
| ENSG00000270696 | AC005034.3 | brown |
| ENSG00000270720 | AC104785.1 | brown |

| | | |
|---|---|---|
| ENSG00000270728 | AL035413.2 | brown |
| ENSG00000270755 | AL136141.1 | brown |
| ENSG00000270820 | AC016727.1 | brown |
| ENSG00000270903 | HNRNPA3P9 | brown |
| ENSG00000270959 | LPP-AS2 | brown |
| ENSG00000270964 | AC016355.1 | brown |
| ENSG00000271032 | AC020907.4 | brown |
| ENSG00000271105 | AC133552.5 | brown |
| ENSG00000271119 | AC026412.3 | brown |
| ENSG00000271204 | AC016831.5 | brown |
| ENSG00000271267 | AL353807.4 | brown |
| ENSG00000271327 | AC010201.2 | brown |
| ENSG00000271461 | AL109809.4 | brown |
| ENSG00000271580 | AL583832.1 | brown |
| ENSG00000271646 | AC099343.3 | brown |
| ENSG00000271664 | AC004890.3 | brown |
| ENSG00000271680 | AC098935.2 | brown |
| ENSG00000271725 | AC103858.1 | brown |
| ENSG00000271789 | AL080317.2 | brown |
| ENSG00000271797 | AC008494.3 | brown |
| ENSG00000271816 | BMS1P4 | brown |
| ENSG00000271819 | RNU6-94P | brown |
| ENSG00000271828 | AC008937.3 | brown |
| ENSG00000271843 | AC012557.1 | brown |
| ENSG00000271888 | AL136162.1 | brown |
| ENSG00000271913 | AL035530.2 | brown |
| ENSG00000271933 | AL603756.1 | brown |
| ENSG00000272034 | SNORD14A | brown |
| ENSG00000272053 | AL160400.1 | brown |
| ENSG00000272079 | AC004233.3 | brown |
| ENSG00000272140 | AC022400.5 | brown |
| ENSG00000272183 | AC005041.3 | brown |
| ENSG00000272211 | AC114760.2 | brown |
| ENSG00000272269 | AL138724.1 | brown |
| ENSG00000272277 | AL031963.3 | brown |
| ENSG00000272305 | AC096887.1 | brown |
| ENSG00000272325 | NUDT3 | brown |
| ENSG00000272333 | KMT2B | brown |
| ENSG00000272367 | AC018754.1 | brown |
| ENSG00000272368 | AC074032.1 | brown |
| ENSG00000272402 | AL031775.2 | brown |
| ENSG00000272419 | AC241585.2 | brown |
| ENSG00000272444 | AL118558.4 | brown |
| ENSG00000272468 | AL021807.1 | brown |
| ENSG00000272486 | AC090922.1 | brown |
| ENSG00000272498 | AC090948.2 | brown |
| ENSG00000272505 | AC104964.3 | brown |
| ENSG00000272555 | AC009974.1 | brown |

| | | |
|---|---|---|
| ENSG00000272562 | AL512343.2 | brown |
| ENSG00000272568 | AC005162.3 | brown |
| ENSG00000272572 | AL138762.1 | brown |
| ENSG00000272573 | MUSTN1 | brown |
| ENSG00000272578 | AP000347.1 | brown |
| ENSG00000272588 | AC139887.4 | brown |
| ENSG00000272604 | AC073073.2 | brown |
| ENSG00000272630 | AL731563.3 | brown |
| ENSG00000272754 | AL133245.1 | brown |
| ENSG00000272834 | AL022238.3 | brown |
| ENSG00000272853 | AC069544.1 | brown |
| ENSG00000272858 | Z93930.3 | brown |
| ENSG00000272909 | AL122035.2 | brown |
| ENSG00000272918 | TSPAN6 | brown |
| ENSG00000273032 | KDM1A | brown |
| ENSG00000273076 | CIAPIN1 | brown |
| ENSG00000273112 | SOX8 | brown |
| ENSG00000273151 | TBXA2R | brown |
| ENSG00000273160 | ELAC2 | brown |
| ENSG00000273188 | NCAPD2 | brown |
| ENSG00000273243 | PTBP1 | brown |
| ENSG00000273311 | CD6 | brown |
| ENSG00000273319 | NUDCD3 | brown |
| ENSG00000273329 | PLEKHB1 | brown |
| ENSG00000273343 | KPNA6 | brown |
| ENSG00000273521 | RTN4R | brown |
| ENSG00000273568 | TDP1 | brown |
| ENSG00000273599 | JADE2 | brown |
| ENSG00000273687 | MAP4 | brown |
| ENSG00000273691 | PPP1R3F | brown |
| ENSG00000273729 | LETMD1 | brown |
| ENSG00000273747 | RAD51 | brown |
| ENSG00000273812 | CYFIP2 | brown |
| ENSG00000273828 | USP36 | brown |
| ENSG00000273891 | MTA3 | brown |
| ENSG00000273893 | PSD | brown |
| ENSG00000273973 | ST3GAL6 | brown |
| ENSG00000274008 | CDON | brown |
| ENSG00000274012 | TLE2 | brown |
| ENSG00000274024 | ELAVL1 | brown |
| ENSG00000274070 | FTSJ1 | brown |
| ENSG00000274124 | POLR1A | brown |
| ENSG00000274180 | SDK2 | brown |
| ENSG00000274225 | CFAP20 | brown |
| ENSG00000274290 | TCOF1 | brown |
| ENSG00000274292 | BUD23 | brown |
| ENSG00000274383 | TCF3 | brown |
| ENSG00000274403 | SPEG | brown |

| | | |
|---|---|---|
| ENSG00000274425 | SREBF1 | brown |
| ENSG00000274512 | NLE1 | brown |
| ENSG00000274605 | MCM6 | brown |
| ENSG00000274627 | MLH1 | brown |
| ENSG00000274695 | PMS2P1 | brown |
| ENSG00000274925 | TCF7 | brown |
| ENSG00000274963 | AACS | brown |
| ENSG00000275022 | ERC1 | brown |
| ENSG00000275056 | NOA1 | brown |
| ENSG00000275092 | CAD | brown |
| ENSG00000275158 | HUWE1 | brown |
| ENSG00000275198 | NOP14 | brown |
| ENSG00000275202 | LZTS3 | brown |
| ENSG00000275215 | SIRPG | brown |
| ENSG00000275432 | GCN1 | brown |
| ENSG00000275449 | PEBP1 | brown |
| ENSG00000275560 | DDX24 | brown |
| ENSG00000275757 | TFAP4 | brown |
| ENSG00000275784 | GOLGA3 | brown |
| ENSG00000275833 | PABPC4 | brown |
| ENSG00000275888 | GLG1 | brown |
| ENSG00000275936 | PITPNM2 | brown |
| ENSG00000276007 | SLC22A17 | brown |
| ENSG00000276043 | PHGDH | brown |
| ENSG00000276073 | EZR | brown |
| ENSG00000276103 | CBX5 | brown |
| ENSG00000276256 | NUP188 | brown |
| ENSG00000276337 | HSP90AB1 | brown |
| ENSG00000276449 | ABL1 | brown |
| ENSG00000276488 | SETD1A | brown |
| ENSG00000276523 | HNRNPM | brown |
| ENSG00000276570 | SEC14L2 | brown |
| ENSG00000276603 | PPIL2 | brown |
| ENSG00000276649 | PIK3IP1 | brown |
| ENSG00000276691 | PATZ1 | brown |
| ENSG00000276840 | TAB1 | brown |
| ENSG00000276900 | CACNA1I | brown |
| ENSG00000276931 | GRAP2 | brown |
| ENSG00000276952 | NDRG3 | brown |
| ENSG00000276961 | NELFCD | brown |
| ENSG00000276968 | SNPH | brown |
| ENSG00000277072 | VSIG1 | brown |
| ENSG00000277117 | USP11 | brown |
| ENSG00000277157 | CD40LG | brown |
| ENSG00000277224 | FGD1 | brown |
| ENSG00000277290 | RGCC | brown |
| ENSG00000277349 | PLLP | brown |
| ENSG00000277400 | DHODH | brown |

| | | |
|---|---|---|
| ENSG00000277425 | FBXO31 | brown |
| ENSG00000277449 | UBE2I | brown |
| ENSG00000277452 | EEF2K | brown |
| ENSG00000277481 | IL21R | brown |
| ENSG00000277548 | IL27RA | brown |
| ENSG00000277586 | OLFM2 | brown |
| ENSG00000277678 | CNTD2 | brown |
| ENSG00000277687 | PRX | brown |
| ENSG00000277728 | TJP3 | brown |
| ENSG00000277942 | GRWD1 | brown |
| ENSG00000278058 | LIG1 | brown |
| ENSG00000278090 | SLC5A5 | brown |
| ENSG00000278129 | LSR | brown |
| ENSG00000278175 | COA1 | brown |
| ENSG00000278341 | URGCP | brown |
| ENSG00000278384 | SPOCK2 | brown |
| ENSG00000278600 | TWNK | brown |
| ENSG00000278601 | LZTS2 | brown |
| ENSG00000278665 | SUFU | brown |
| ENSG00000278725 | UBTF | brown |
| ENSG00000278765 | ABCC3 | brown |
| ENSG00000278771 | MPP2 | brown |
| ENSG00000278811 | ALDOC | brown |
| ENSG00000278829 | WFS1 | brown |
| ENSG00000278831 | TMEM109 | brown |
| ENSG00000278845 | IL10RA | brown |
| ENSG00000278864 | CD5 | brown |
| ENSG00000278867 | AMBRA1 | brown |
| ENSG00000278875 | MADD | brown |
| ENSG00000278881 | ASIC1 | brown |
| ENSG00000278897 | CHD4 | brown |
| ENSG00000278918 | ADTRP | brown |
| ENSG00000278949 | MCM3 | brown |
| ENSG00000278987 | PTK7 | brown |
| ENSG00000278991 | MRPS27 | brown |
| ENSG00000278997 | C3orf14 | brown |
| ENSG00000279010 | VIPR1 | brown |
| ENSG00000279019 | FOXP1 | brown |
| ENSG00000279048 | EIF4G1 | brown |
| ENSG00000279059 | KANSL3 | brown |
| ENSG00000279078 | NCL | brown |
| ENSG00000279080 | EFHD1 | brown |
| ENSG00000279089 | UXS1 | brown |
| ENSG00000279106 | GORASP2 | brown |
| ENSG00000279133 | BCL9 | brown |
| ENSG00000279145 | FBXO2 | brown |
| ENSG00000279155 | AGMAT | brown |
| ENSG00000279159 | MAN1C1 | brown |

| | | |
|---|---|---|
| ENSG00000279161 | RPN2 | brown |
| ENSG00000279162 | C2orf40 | brown |
| ENSG00000279166 | RBM18 | brown |
| ENSG00000279179 | GOT1 | brown |
| ENSG00000279187 | SFRP5 | brown |
| ENSG00000279191 | EGR1 | brown |
| ENSG00000279192 | EPHX2 | brown |
| ENSG00000279199 | UBIAD1 | brown |
| ENSG00000279203 | GJB6 | brown |
| ENSG00000279259 | LY9 | brown |
| ENSG00000279278 | DDX54 | brown |
| ENSG00000279283 | CHPF | brown |
| ENSG00000279328 | NCOA5 | brown |
| ENSG00000279330 | PLCG1 | brown |
| ENSG00000279339 | TOX2 | brown |
| ENSG00000279344 | STX16 | brown |
| ENSG00000279349 | DDX27 | brown |
| ENSG00000279370 | BCAS4 | brown |
| ENSG00000279382 | LYPD3 | brown |
| ENSG00000279386 | XPO5 | brown |
| ENSG00000279407 | MOCS1 | brown |
| ENSG00000279412 | C1orf61 | brown |
| ENSG00000279415 | SLC25A23 | brown |
| ENSG00000279425 | LRFN3 | brown |
| ENSG00000279432 | CCR7 | brown |
| ENSG00000279433 | TSKS | brown |
| ENSG00000279434 | AHDC1 | brown |
| ENSG00000279447 | ELK1 | brown |
| ENSG00000279456 | FBXL12 | brown |
| ENSG00000279464 | EMC1 | brown |
| ENSG00000279469 | FBXL16 | brown |
| ENSG00000279495 | SMARCA4 | brown |
| ENSG00000279500 | METTL16 | brown |
| ENSG00000279507 | TNFRSF19 | brown |
| ENSG00000279520 | DGCR8 | brown |
| ENSG00000279541 | GAL3ST1 | brown |
| ENSG00000279554 | IFT22 | brown |
| ENSG00000279555 | OPN1SW | brown |
| ENSG00000279568 | ILF3 | brown |
| ENSG00000279570 | FOXJ1 | brown |
| ENSG00000279608 | DOHH | brown |
| ENSG00000279611 | CRB3 | brown |
| ENSG00000279613 | HABP4 | brown |
| ENSG00000279631 | NFATC1 | brown |
| ENSG00000279649 | TMEM204 | brown |
| ENSG00000279662 | CA6 | brown |
| ENSG00000279672 | ZSWIM4 | brown |
| ENSG00000279699 | DHX30 | brown |

| | | |
|---|---|---|
| ENSG00000279700 | NUP210 | brown |
| ENSG00000279738 | MYBBP1A | brown |
| ENSG00000279744 | RPA1 | brown |
| ENSG00000279748 | MORC2 | brown |
| ENSG00000279758 | REG4 | brown |
| ENSG00000279759 | TSPAN2 | brown |
| ENSG00000279765 | STT3A | brown |
| ENSG00000279789 | NAT10 | brown |
| ENSG00000279792 | TESPA1 | brown |
| ENSG00000279794 | REPS1 | brown |
| ENSG00000279805 | USP20 | brown |
| ENSG00000279811 | MYC | brown |
| ENSG00000279821 | UBAP2 | brown |
| ENSG00000279825 | ZSCAN9 | brown |
| ENSG00000279833 | SLC22A23 | brown |
| ENSG00000279838 | SEC31A | brown |
| ENSG00000279873 | LEF1 | brown |
| ENSG00000279880 | GLIPR1 | brown |
| ENSG00000279900 | ESYT1 | brown |
| ENSG00000279901 | EFCAB11 | brown |
| ENSG00000279933 | FBLN5 | brown |
| ENSG00000279942 | HAPLN3 | brown |
| ENSG00000279948 | MFGE8 | brown |
| ENSG00000279982 | CRTC3 | brown |
| ENSG00000280010 | SLC5A2 | brown |
| ENSG00000280020 | FTO | brown |
| ENSG00000280039 | ZFHX3 | brown |
| ENSG00000280046 | MED9 | brown |
| ENSG00000280054 | UTP4 | brown |
| ENSG00000280063 | NOB1 | brown |
| ENSG00000280067 | AC005070.3 | brown |
| ENSG00000280080 | DGCR9 | brown |
| ENSG00000280099 | AL021707.7 | brown |
| ENSG00000280138 | AL590385.2 | brown |
| ENSG00000280181 | AC073957.3 | brown |
| ENSG00000280183 | AL359962.2 | brown |
| ENSG00000280213 | AL022328.3 | brown |
| ENSG00000280214 | Z82243.1 | brown |
| ENSG00000280248 | DGCR11 | brown |
| ENSG00000280287 | AC058791.1 | brown |
| ENSG00000280295 | AC078846.1 | brown |
| ENSG00000280300 | AC007663.4 | brown |
| ENSG00000280332 | AL162274.1 | brown |
| ENSG00000280353 | AC131009.3 | brown |
| ENSG00000280378 | AL731571.1 | brown |
| ENSG00000280383 | AC004223.4 | brown |
| ENSG00000280387 | AC087284.1 | brown |
| ENSG00000280388 | AC007686.3 | brown |

| | | |
|---|---|---|
| ENSG00000280407 | AC022558.3 | brown |
| ENSG00000280441 | BX640514.2 | brown |
| ENSG00000280537 | AL133227.1 | brown |
| ENSG00000280614 | AL731566.1 | brown |
| ENSG00000280800 | AL133520.1 | brown |
| ENSG00000280828 | AC025162.2 | brown |
| ENSG00000280927 | Metazoa_SRP | brown |
| ENSG00000280987 | RN7SL2 | brown |
| ENSG00000281005 | AL590282.1 | brown |
| ENSG00000281106 | CASTOR2 | brown |
| ENSG00000281181 | AC074029.3 | brown |
| ENSG00000281183 | NATD1 | brown |
| ENSG00000281195 | AP001065.2 | brown |
| ENSG00000281207 | HIST1H2BE | brown |
| ENSG00000281357 | AC084018.2 | brown |
| ENSG00000281383 | AC103691.1 | brown |
| ENSG00000281490 | AC090510.2 | brown |
| ENSG00000281530 | AC114271.1 | brown |
| ENSG00000281691 | TBC1D3L | brown |
| ENSG00000281896 | AL355338.1 | brown |
| ENSG00000282133 | AC092123.2 | brown |
| ENSG00000282508 | AC108704.1 | brown |
| ENSG00000282572 | AC008741.2 | brown |
| ENSG00000282826 | RN7SL600P | brown |
| ENSG00000282827 | MIR6753 | brown |
| ENSG00000282936 | AC020663.3 | brown |
| ENSG00000282980 | AL031710.2 | brown |
| ENSG00000282988 | AC244196.2 | brown |
| ENSG00000283036 | AL512791.2 | brown |
| ENSG00000283050 | AL161421.1 | brown |
| ENSG00000283160 | RNA5-8SN4 | brown |
| ENSG00000283208 | AC022149.2 | brown |
| ENSG00000283294 | MIR6859-3 | brown |
| ENSG00000283297 | AC008115.3 | brown |
| ENSG00000283341 | RNA5-8SN2 | brown |
| ENSG00000283378 | AL034549.1 | brown |
| ENSG00000283406 | AC073107.2 | brown |
| ENSG00000283526 | AC132872.3 | brown |
| ENSG00000283528 | AC004263.2 | brown |
| ENSG00000283597 | AC079414.3 | brown |
| ENSG00000283674 | UHRF1 | brown |
| ENSG00000283743 | AL034549.2 | brown |
| ENSG00000283907 | U4 | brown |
| ENSG00000283930 | AC011043.1 | brown |
| ENSG00000283994 | AC105429.1 | brown |
| ENSG00000284052 | AC004076.2 | brown |
| ENSG00000284154 | AC008735.4 | brown |
| ENSG00000284373 | AC025287.3 | brown |

| | | |
|---|---|---|
| ENSG00000284428 | AC010327.5 | brown |
| ENSG00000284526 | AL109614.1 | brown |
| ENSG00000284602 | AL117335.1 | brown |
| ENSG00000284633 | AC004466.3 | brown |
| ENSG00000284634 | PMS2P10 | brown |
| ENSG00000284669 | AC023157.3 | brown |
| ENSG00000284685 | AC009041.4 | brown |
| ENSG00000284738 | AL121772.3 | brown |
| ENSG00000000003 | MIR7848 | green |
| ENSG00000004487 | AL158196.1 | green |
| ENSG00000005194 | STAG3L2 | green |
| ENSG00000005513 | FP565260.3 | green |
| ENSG00000006638 | HIST1H4D | green |
| ENSG00000006744 | HIST1H2BF | green |
| ENSG00000010292 | AC136475.10 | green |
| ENSG00000011304 | AC133919.3 | green |
| ENSG00000013725 | AC145212.2 | green |
| ENSG00000015676 | AL121890.4 | green |
| ENSG00000021300 | CEBPB-AS1 | green |
| ENSG00000025800 | RN7SL473P | green |
| ENSG00000040608 | PKD1L3 | green |
| ENSG00000042088 | AC018926.3 | green |
| ENSG00000043143 | NEFL | green |
| ENSG00000047849 | U1 | green |
| ENSG00000049769 | AL139407.1 | green |
| ENSG00000050426 | AC097641.3 | green |
| ENSG00000051180 | MIR8075 | green |
| ENSG00000055163 | AC009159.3 | green |
| ENSG00000055483 | AC036108.4 | green |
| ENSG00000057935 | ZNF8 | green |
| ENSG00000059915 | GLIDR | green |
| ENSG00000064225 | AC138028.6 | green |
| ENSG00000064309 | AL354822.1 | green |
| ENSG00000065717 | AC015871.3 | green |
| ENSG00000066044 | AL158163.2 | green |
| ENSG00000068438 | AC020765.3 | green |
| ENSG00000068654 | AC020765.4 | green |
| ENSG00000069188 | AC004477.3 | green |
| ENSG00000070761 | RN7SL3 | green |
| ENSG00000070814 | LINC00624 | green |
| ENSG00000071462 | AC099811.5 | green |
| ENSG00000071564 | AL513190.1 | green |
| ENSG00000072195 | MRPL45 | green |
| ENSG00000072310 | AC055811.4 | green |
| ENSG00000073536 | AC090616.7 | green |
| ENSG00000076003 | AC025809.2 | green |
| ENSG00000076242 | FP325331.1 | green |
| ENSG00000078319 | AC020951.1 | green |

| | | |
|---|---|---|
| ENSG00000081059 | AC080112.4 | green |
| ENSG00000081760 | AC127070.5 | green |
| ENSG00000082805 | AL031009.1 | green |
| ENSG00000084092 | AC090181.3 | green |
| ENSG00000084774 | AL662907.1 | green |
| ENSG00000086758 | AL031587.6 | green |
| ENSG00000087269 | AC009090.4 | green |
| ENSG00000088899 | AC080080.1 | green |
| ENSG00000089012 | AC007485.2 | green |
| ENSG00000089154 | SND1-IT1 | green |
| ENSG00000089220 | AL022322.2 | green |
| ENSG00000089737 | AC005839.1 | green |
| ENSG00000090447 | AC009093.7 | green |
| ENSG00000090615 | AC018628.1 | green |
| ENSG00000090621 | AC011912.1 | green |
| ENSG00000090863 | AC233300.1 | green |
| ENSG00000090975 | AC003681.1 | green |
| ENSG00000092096 | AC093503.3 | green |
| ENSG00000092621 | AC141586.5 | green |
| ENSG00000092820 | AC009951.1 | green |
| ENSG00000094916 | AL662907.2 | green |
| ENSG00000095319 | AC027601.5 | green |
| ENSG00000096384 | AC068491.4 | green |
| ENSG00000097007 | PWAR5 | green |
| ENSG00000099381 | AC068669.1 | green |
| ENSG00000099783 | AC005785.2 | green |
| ENSG00000100003 | AC087741.3 | green |
| ENSG00000100023 | AC245060.6 | green |
| ENSG00000100100 | AC131009.4 | green |
| ENSG00000100105 | AC073439.1 | green |
| ENSG00000100324 | AJ003147.3 | green |
| ENSG00000100346 | AC100788.2 | green |
| ENSG00000100351 | AC007342.6 | green |
| ENSG00000101079 | AC092692.1 | green |
| ENSG00000101158 | AC004777.1 | green |
| ENSG00000101298 | AC018665.1 | green |
| ENSG00000101842 | AC021106.3 | green |
| ENSG00000102226 | AC007191.1 | green |
| ENSG00000102245 | AC020763.3 | green |
| ENSG00000102302 | AC099494.2 | green |
| ENSG00000102760 | AC092279.2 | green |
| ENSG00000102934 | AC015799.1 | green |
| ENSG00000102967 | AC018529.3 | green |
| ENSG00000103264 | AL049776.1 | green |
| ENSG00000103275 | AL118508.4 | green |
| ENSG00000103319 | AL353763.1 | green |
| ENSG00000103522 | AC096720.2 | green |
| ENSG00000104998 | AC109454.4 | green |

| | | |
|---|---|---|
| ENSG00000105088 | AL928654.5 | green |
| ENSG00000105219 | AC108704.2 | green |
| ENSG00000105227 | AC112484.5 | green |
| ENSG00000105289 | AC093525.8 | green |
| ENSG00000105447 | AC005261.6 | green |
| ENSG00000105486 | AC130448.2 | green |
| ENSG00000105641 | AC091181.1 | green |
| ENSG00000105699 | AC093525.9 | green |
| ENSG00000106603 | AC099804.1 | green |
| ENSG00000106608 | AL353795.3 | green |
| ENSG00000107742 | AC012313.10 | green |
| ENSG00000107815 | AC124283.5 | green |
| ENSG00000107816 | AL158211.5 | green |
| ENSG00000107882 | AC020978.10 | green |
| ENSG00000108312 | AC131649.2 | green |
| ENSG00000108846 | AP006621.5 | green |
| ENSG00000108852 | AC004264.2 | green |
| ENSG00000109107 | AC131212.2 | green |
| ENSG00000109501 | AL022311.1 | green |
| ENSG00000110108 | AC132938.6 | green |
| ENSG00000110324 | AC008764.9 | green |
| ENSG00000110448 | AC105339.4 | green |
| ENSG00000110497 | AC118344.2 | green |
| ENSG00000110514 | AC013394.1 | green |
| ENSG00000110881 | AC120114.4 | green |
| ENSG00000111642 | AC015909.5 | green |
| ENSG00000111863 | AC024580.1 | green |
| ENSG00000112118 | Z95114.3 | green |
| ENSG00000112655 | AC093330.2 | green |
| ENSG00000113048 | AC145098.2 | green |
| ENSG00000114405 | AC003957.1 | green |
| ENSG00000114812 | AL031846.2 | green |
| ENSG00000114861 | AL356273.3 | green |
| ENSG00000114867 | LINC01126 | green |
| ENSG00000114982 | AC134407.3 | green |
| ENSG00000115053 | AP001767.4 | green |
| ENSG00000115468 | AC092117.2 | green |
| ENSG00000115652 | AL031595.1 | green |
| ENSG00000115806 | AL353597.3 | green |
| ENSG00000116128 | AC008895.1 | green |
| ENSG00000116661 | AL162274.3 | green |
| ENSG00000116771 | AP001350.2 | green |
| ENSG00000117643 | AC116407.3 | green |
| ENSG00000118705 | RN7SKP23 | green |
| ENSG00000119147 | AC104581.5 | green |
| ENSG00000119446 | AC004241.5 | green |
| ENSG00000120053 | AC012676.5 | green |
| ENSG00000120057 | AC023818.2 | green |

| | | |
|---|---|---|
| ENSG00000120738 | TBC1D22A-AS1 | green |
| ENSG00000120915 | AL603750.1 | green |
| ENSG00000120942 | AC027290.2 | green |
| ENSG00000121742 | AC025262.2 | green |
| ENSG00000122224 | AC067852.7 | green |
| ENSG00000123064 | UCKL1-AS1 | green |
| ENSG00000123989 | AC027682.7 | green |
| ENSG00000124160 | AC124319.4 | green |
| ENSG00000124181 | AC131212.3 | green |
| ENSG00000124191 | AC099811.6 | green |
| ENSG00000124222 | AC117503.5 | green |
| ENSG00000124228 | AC020917.4 | green |
| ENSG00000124243 | AC011466.4 | green |
| ENSG00000124466 | AL353898.3 | green |
| ENSG00000124571 | Z95331.1 | green |
| ENSG00000124615 | AL109806.1 | green |
| ENSG00000125462 | AC006330.1 | green |
| ENSG00000125648 | AC132872.4 | green |
| ENSG00000126243 | FP236383.1 | green |
| ENSG00000126353 | AC068946.1 | green |
| ENSG00000126467 | FP236383.2 | green |
| ENSG00000126705 | FP671120.3 | green |
| ENSG00000126767 | AC090114.3 | green |
| ENSG00000127452 | CTBP1-AS | green |
| ENSG00000127463 | MATR3 | green |
| ENSG00000127585 | LINC00921 | green |
| ENSG00000127616 | LINC00282 | green |
| ENSG00000127804 | FP236383.3 | green |
| ENSG00000127863 | NPTN-IT1 | green |
| ENSG00000128191 | AC007878.1 | green |
| ENSG00000128242 | SLFNL1-AS1 | green |
| ENSG00000128581 | ARRDC3-AS1 | green |
| ENSG00000128617 | FP671120.4 | green |
| ENSG00000129351 | CICP14 | green |
| ENSG00000129654 | AC004461.2 | green |
| ENSG00000129932 | RBM5-AS1 | green |
| ENSG00000130545 | AC018638.8 | green |
| ENSG00000130956 | AC245427.4 | green |
| ENSG00000131196 | LINC01002 | green |
| ENSG00000131634 | AC215522.2 | green |
| ENSG00000131686 | FRG1CP | green |
| ENSG00000132003 | AC134772.2 | green |
| ENSG00000132153 | AC004706.4 | green |
| ENSG00000132182 | AC055839.1 | green |
| ENSG00000132382 | HIST1H3D | green |
| ENSG00000132383 | LINC01988 | green |
| ENSG00000133422 | GTF2IP12 | green |
| ENSG00000134193 | MIR4521 | green |

| | | |
|---|---|---|
| ENSG00000134198 | AC001226.2 | green |
| ENSG00000134910 | AP005212.5 | green |
| ENSG00000135372 | AC005841.2 | green |
| ENSG00000135426 | AC068205.2 | green |
| ENSG00000135597 | BX088645.1 | green |
| ENSG00000136878 | DGCR5 | green |
| ENSG00000136997 | AL160276.1 | green |
| ENSG00000137073 | AC073264.3 | green |
| ENSG00000137185 | FAM169B | green |
| ENSG00000137266 | AC068587.6 | green |
| ENSG00000138674 | AL009172.2 | green |
| ENSG00000138795 | AD000090.1 | green |
| ENSG00000139278 | AL117339.5 | green |
| ENSG00000139641 | AC092652.3 | green |
| ENSG00000140025 | AC006460.2 | green |
| ENSG00000140092 | MIR3605 | green |
| ENSG00000140511 | AC106774.8 | green |
| ENSG00000140545 | AC092329.4 | green |
| ENSG00000140577 | AC015802.6 | green |
| ENSG00000140675 | AL031432.4 | green |
| ENSG00000140718 | AL031590.1 | green |
| ENSG00000140836 | AC092821.3 | green |
| ENSG00000141026 | AC092053.3 | green |
| ENSG00000141076 | AC009093.10 | green |
| ENSG00000141101 | AL358472.5 | green |
| ENSG00000141456 | STPG1 | green |
| ENSG00000141564 | WNT16 | green |
| ENSG00000141576 | TMEM176A | green |
| ENSG00000142156 | TFPI | green |
| ENSG00000142230 | ABCB4 | green |
| ENSG00000142235 | SCIN | green |
| ENSG00000142655 | TEAD3 | green |
| ENSG00000142798 | SYN1 | green |
| ENSG00000143167 | CELSR3 | green |
| ENSG00000143772 | TMEM159 | green |
| ENSG00000143793 | NDUFS1 | green |
| ENSG00000143799 | SNAPC1 | green |
| ENSG00000143811 | SLC18A1 | green |
| ENSG00000143850 | TNC | green |
| ENSG00000144028 | UTS2 | green |
| ENSG00000144034 | LZTS1 | green |
| ENSG00000144120 | MCM10 | green |
| ENSG00000144152 | GSTO2 | green |
| ENSG00000144635 | ME1 | green |
| ENSG00000144647 | KIF26A | green |
| ENSG00000145911 | TRO | green |
| ENSG00000145945 | PLEKHH3 | green |
| ENSG00000146904 | CLCN4 | green |

| | | |
|---|---|---|
| ENSG00000147130 | GTSE1 | green |
| ENSG00000147140 | COL5A3 | green |
| ENSG00000147144 | IL12RB2 | green |
| ENSG00000147324 | ITGAE | green |
| ENSG00000147457 | EFR3B | green |
| ENSG00000148229 | RAD54L | green |
| ENSG00000148606 | HSD17B14 | green |
| ENSG00000148840 | CDIP1 | green |
| ENSG00000148843 | LAG3 | green |
| ENSG00000149115 | P3H2 | green |
| ENSG00000149136 | PDPR | green |
| ENSG00000149582 | TF | green |
| ENSG00000150990 | PITPNM3 | green |
| ENSG00000151065 | CCDC80 | green |
| ENSG00000151929 | COL9A3 | green |
| ENSG00000152127 | BLNK | green |
| ENSG00000152503 | CEP170B | green |
| ENSG00000154016 | SEZ6L | green |
| ENSG00000154027 | CRYBB1 | green |
| ENSG00000154309 | POLR2F | green |
| ENSG00000154328 | ZFYVE21 | green |
| ENSG00000154358 | PSMA6 | green |
| ENSG00000154764 | NTSR1 | green |
| ENSG00000155265 | EEF1A2 | green |
| ENSG00000155660 | SCML2 | green |
| ENSG00000156304 | FLT1 | green |
| ENSG00000156515 | BFAR | green |
| ENSG00000156853 | SYT17 | green |
| ENSG00000157303 | IQCH | green |
| ENSG00000157570 | TTC23 | green |
| ENSG00000157978 | EBI3 | green |
| ENSG00000158169 | SHD | green |
| ENSG00000158373 | PLA2G4C | green |
| ENSG00000158467 | STEAP1B | green |
| ENSG00000158485 | DFNA5 | green |
| ENSG00000159164 | CRHR2 | green |
| ENSG00000159208 | TMEM176B | green |
| ENSG00000160049 | PALD1 | green |
| ENSG00000160185 | HLF | green |
| ENSG00000162496 | TMEM97 | green |
| ENSG00000162892 | AREG | green |
| ENSG00000162894 | TECTA | green |
| ENSG00000163359 | FOLR3 | green |
| ENSG00000163399 | RASAL1 | green |
| ENSG00000164265 | ST8SIA1 | green |
| ENSG00000164362 | AICDA | green |
| ENSG00000164867 | TPD52L1 | green |
| ENSG00000165171 | PHACTR1 | green |

| | | |
|---|---|---|
| ENSG00000165269 | GZMK | green |
| ENSG00000165271 | NPR3 | green |
| ENSG00000165272 | SLC12A7 | green |
| ENSG00000165475 | EHHADH | green |
| ENSG00000165733 | ACTR8 | green |
| ENSG00000165821 | EFCC1 | green |
| ENSG00000166311 | GRB14 | green |
| ENSG00000166313 | FN1 | green |
| ENSG00000166340 | ABCB6 | green |
| ENSG00000166526 | CD207 | green |
| ENSG00000166681 | PRG4 | green |
| ENSG00000166886 | OLFML3 | green |
| ENSG00000167106 | NEK2 | green |
| ENSG00000167191 | 2-Mar | green |
| ENSG00000167414 | CTGF | green |
| ENSG00000167664 | PIGZ | green |
| ENSG00000167861 | BCL11A | green |
| ENSG00000167880 | CNNM1 | green |
| ENSG00000167986 | EPX | green |
| ENSG00000168286 | ADGRB2 | green |
| ENSG00000168434 | ZSCAN20 | green |
| ENSG00000168872 | GTSF1L | green |
| ENSG00000169710 | EDN3 | green |
| ENSG00000170004 | PACSIN1 | green |
| ENSG00000170468 | SLC17A3 | green |
| ENSG00000171130 | KCNK17 | green |
| ENSG00000171219 | SRMS | green |
| ENSG00000171603 | PLGLB2 | green |
| ENSG00000171793 | FOSB | green |
| ENSG00000172005 | TMEM74B | green |
| ENSG00000172534 | AMOT | green |
| ENSG00000172893 | CANX | green |
| ENSG00000173530 | STRIP2 | green |
| ENSG00000174306 | USP6 | green |
| ENSG00000174684 | EPB41L4A | green |
| ENSG00000174807 | SLC34A1 | green |
| ENSG00000175707 | GFPT2 | green |
| ENSG00000176022 | RAI2 | green |
| ENSG00000176531 | RHPN2 | green |
| ENSG00000176619 | SLC52A1 | green |
| ENSG00000177096 | FCRL2 | green |
| ENSG00000177602 | WASF3 | green |
| ENSG00000177685 | PTGFRN | green |
| ENSG00000178038 | FADS2 | green |
| ENSG00000178409 | ADAMTS8 | green |
| ENSG00000178921 | HRK | green |
| ENSG00000179222 | RAB11FIP5 | green |
| ENSG00000179409 | LAMC1 | green |

| | | |
|---|---|---|
| ENSG00000179715 | ARMC9 | green |
| ENSG00000179855 | NEK3 | green |
| ENSG00000181035 | NACAD | green |
| ENSG00000181585 | FRS3 | green |
| ENSG00000182162 | LRRC32 | green |
| ENSG00000182307 | SLCO5A1 | green |
| ENSG00000182866 | FXYD2 | green |
| ENSG00000183624 | NUSAP1 | green |
| ENSG00000183655 | SLC39A5 | green |
| ENSG00000183784 | SLC27A2 | green |
| ENSG00000184117 | TSPAN3 | green |
| ENSG00000184178 | TICRR | green |
| ENSG00000184271 | PELP1 | green |
| ENSG00000184640 | RPTOR | green |
| ENSG00000185361 | RNF157 | green |
| ENSG00000185933 | COL6A1 | green |
| ENSG00000186001 | SAE1 | green |
| ENSG00000186049 | LMTK3 | green |
| ENSG00000186409 | SLC47A1 | green |
| ENSG00000186575 | PEX14 | green |
| ENSG00000186854 | HSPG2 | green |
| ENSG00000187535 | IGSF3 | green |
| ENSG00000188191 | GPR161 | green |
| ENSG00000188807 | POGK | green |
| ENSG00000189091 | GPA33 | green |
| ENSG00000189283 | ITPKB | green |
| ENSG00000196363 | C1orf35 | green |
| ENSG00000196507 | PARP1 | green |
| ENSG00000196526 | PYCR2 | green |
| ENSG00000196700 | PLEKHA6 | green |
| ENSG00000196812 | WDPCP | green |
| ENSG00000197006 | SNRNP200 | green |
| ENSG00000197093 | TPRKB | green |
| ENSG00000197102 | TMEM177 | green |
| ENSG00000197386 | FBLN7 | green |
| ENSG00000197496 | AL078621.1 | green |
| ENSG00000197635 | DYNC1LI1 | green |
| ENSG00000197694 | OSBPL10 | green |
| ENSG00000197859 | POMGNT2 | green |
| ENSG00000197880 | STAC | green |
| ENSG00000198218 | N4BP3 | green |
| ENSG00000198286 | FAM50B | green |
| ENSG00000198429 | PSD2 | green |
| ENSG00000198483 | TNFRSF21 | green |
| ENSG00000198851 | SDK1 | green |
| ENSG00000203485 | EPHA1 | green |
| ENSG00000203814 | TMEM27 | green |
| ENSG00000204055 | RAB41 | green |

| | | |
|---|---|---|
| ENSG00000204138 | ZMYM3 | green |
| ENSG00000204525 | NONO | green |
| ENSG00000204745 | CCDC120 | green |
| ENSG00000204947 | MFHAS1 | green |
| ENSG00000205937 | CHMP7 | green |
| ENSG00000207110 | ZNF462 | green |
| ENSG00000211667 | POLE3 | green |
| ENSG00000211776 | POLR3A | green |
| ENSG00000211777 | PPRC1 | green |
| ENSG00000211785 | PDCD11 | green |
| ENSG00000211791 | TNKS1BP1 | green |
| ENSG00000211794 | SSRP1 | green |
| ENSG00000211795 | TMEM25 | green |
| ENSG00000211797 | ADRA2A | green |
| ENSG00000211800 | DHX37 | green |
| ENSG00000211803 | DCP1B | green |
| ENSG00000211807 | THRB | green |
| ENSG00000211810 | AKR1C2 | green |
| ENSG00000211814 | BAG3 | green |
| ENSG00000211816 | MGAT5 | green |
| ENSG00000211818 | OLAH | green |
| ENSG00000211819 | TRIM36 | green |
| ENSG00000213024 | TCTEX1D1 | green |
| ENSG00000213075 | PLOD2 | green |
| ENSG00000213203 | ACOXL | green |
| ENSG00000213626 | LPCAT1 | green |
| ENSG00000214653 | GRAP | green |
| ENSG00000214772 | AK5 | green |
| ENSG00000215012 | CEP112 | green |
| ENSG00000215475 | DISP1 | green |
| ENSG00000215859 | NEIL2 | green |
| ENSG00000223650 | OBSCN | green |
| ENSG00000225342 | MAGED4 | green |
| ENSG00000225431 | WNT7A | green |
| ENSG00000225434 | FLCN | green |
| ENSG00000225632 | RSPH10B | green |
| ENSG00000229036 | PTPRN2 | green |
| ENSG00000229191 | GOLGA7B | green |
| ENSG00000229358 | PDIA4 | green |
| ENSG00000229474 | KCNMA1 | green |
| ENSG00000229647 | SCAF4 | green |
| ENSG00000230487 | HK1 | green |
| ENSG00000231043 | LRFN2 | green |
| ENSG00000232021 | ZNF689 | green |
| ENSG00000232208 | SUSD3 | green |
| ENSG00000232850 | CLEC18C | green |
| ENSG00000233850 | TSPAN18 | green |
| ENSG00000234699 | LDLRAP1 | green |

| | | |
|---|---|---|
| ENSG00000235192 | FANCC | green |
| ENSG00000235280 | HIST1H2BD | green |
| ENSG00000235823 | AHCYL2 | green |
| ENSG00000236533 | FAM86C1 | green |
| ENSG00000237693 | CD1B | green |
| ENSG00000237765 | SV2A | green |
| ENSG00000237943 | CIART | green |
| ENSG00000240143 | TUBBP5 | green |
| ENSG00000242498 | CELF3 | green |
| ENSG00000242861 | ACE | green |
| ENSG00000243678 | DFFA | green |
| ENSG00000246223 | TMPRSS3 | green |
| ENSG00000247077 | UBASH3A | green |
| ENSG00000249859 | CBS | green |
| ENSG00000250731 | AIRE | green |
| ENSG00000254615 | VAV2 | green |
| ENSG00000255967 | C9orf116 | green |
| ENSG00000257495 | BRSK1 | green |
| ENSG00000258511 | CXCR5 | green |
| ENSG00000259803 | MAML1 | green |
| ENSG00000259956 | NAPEPLD | green |
| ENSG00000260236 | SIGLEC11 | green |
| ENSG00000260496 | SLC45A1 | green |
| ENSG00000260807 | DHRS3 | green |
| ENSG00000260910 | IL23R | green |
| ENSG00000260947 | VANGL2 | green |
| ENSG00000261087 | C1orf115 | green |
| ENSG00000261150 | IL24 | green |
| ENSG00000266289 | FCMR | green |
| ENSG00000267128 | ALMS1P1 | green |
| ENSG00000268240 | ERCC3 | green |
| ENSG00000271009 | COL6A3 | green |
| ENSG00000272386 | LMOD3 | green |
| ENSG00000272483 | ATP1A1 | green |
| ENSG00000273247 | ADORA1 | green |
| ENSG00000273680 | C4orf36 | green |
| ENSG00000274021 | CXCL3 | green |
| ENSG00000274523 | EXO5 | green |
| ENSG00000275342 | SCGB3A2 | green |
| ENSG00000275395 | CMYA5 | green |
| ENSG00000275759 | TERT | green |
| ENSG00000277301 | ANKRD55 | green |
| ENSG00000277734 | KIAA1324L | green |
| ENSG00000277978 | INTS4P1 | green |
| ENSG00000278126 | NOS3 | green |
| ENSG00000278195 | CCDC171 | green |
| ENSG00000278993 | METTL27 | green |
| ENSG00000001460 | AQP7 | grey |

| | | |
|---|---|---|
| ENSG00000002745 | NOL6 | grey |
| ENSG00000002933 | AQP3 | grey |
| ENSG00000003436 | MELK | grey |
| ENSG00000005471 | CRYL1 | grey |
| ENSG00000006747 | STOX1 | grey |
| ENSG00000007866 | BMS1 | grey |
| ENSG00000008056 | SALL2 | grey |
| ENSG00000008300 | PRAP1 | grey |
| ENSG00000011638 | HACD1 | grey |
| ENSG00000023228 | C16orf71 | grey |
| ENSG00000023608 | SMPD1 | grey |
| ENSG00000036565 | APBB1 | grey |
| ENSG00000041982 | TPP1 | grey |
| ENSG00000049247 | NETO1 | grey |
| ENSG00000061337 | ZNF3 | grey |
| ENSG00000065328 | BEX3 | grey |
| ENSG00000065621 | PLEKHA7 | grey |
| ENSG00000065833 | STRCP1 | grey |
| ENSG00000066735 | C16orf45 | grey |
| ENSG00000067445 | RBPMS2 | grey |
| ENSG00000068137 | NAB2 | grey |
| ENSG00000073464 | MTMR10 | grey |
| ENSG00000075218 | FAM102A | grey |
| ENSG00000080573 | GPRC5B | grey |
| ENSG00000081985 | PRRT2 | grey |
| ENSG00000083457 | GNG8 | grey |
| ENSG00000084710 | TMIGD2 | grey |
| ENSG00000085999 | SRR | grey |
| ENSG00000087076 | HID1 | grey |
| ENSG00000089486 | EVPL | grey |
| ENSG00000089692 | DDB1 | grey |
| ENSG00000090530 | THAP11 | grey |
| ENSG00000090857 | COG7 | grey |
| ENSG00000091513 | KCTD19 | grey |
| ENSG00000091622 | DDX19A | grey |
| ENSG00000091986 | AR | grey |
| ENSG00000092758 | EFNA1 | grey |
| ENSG00000095585 | CRADD | grey |
| ENSG00000099814 | CKAP2L | grey |
| ENSG00000100095 | FASN | grey |
| ENSG00000100122 | MT1E | grey |
| ENSG00000100142 | TMEM42 | grey |
| ENSG00000100711 | CHD3 | grey |
| ENSG00000100902 | OR2A20P | grey |
| ENSG00000101188 | DCLK2 | grey |
| ENSG00000101210 | KRT86 | grey |
| ENSG00000102098 | RIOX1 | grey |
| ENSG00000102755 | ZNF16 | grey |

| | | |
|---|---|---|
| ENSG00000103429 | RPS9 | grey |
| ENSG00000103528 | GSTA4 | grey |
| ENSG00000103599 | ATP6V0E2 | grey |
| ENSG00000103852 | CDC42BPG | grey |
| ENSG00000105246 | PDE7B | grey |
| ENSG00000105251 | DNAI2 | grey |
| ENSG00000105499 | CLSTN1 | grey |
| ENSG00000105889 | GATM | grey |
| ENSG00000105928 | CTPS1 | grey |
| ENSG00000106113 | FAM90A1 | grey |
| ENSG00000106565 | RRM2 | grey |
| ENSG00000107719 | MAL | grey |
| ENSG00000108924 | CXCR6 | grey |
| ENSG00000109084 | NEGR1 | grey |
| ENSG00000109321 | CFAP53 | grey |
| ENSG00000109927 | HCFC1 | grey |
| ENSG00000110203 | FUT10 | grey |
| ENSG00000111344 | DHCR7 | grey |
| ENSG00000111728 | PLAC8L1 | grey |
| ENSG00000111732 | TNFRSF10D | grey |
| ENSG00000111907 | CSPG4 | grey |
| ENSG00000112137 | HOXB2 | grey |
| ENSG00000113088 | ZHX3 | grey |
| ENSG00000113389 | C11orf45 | grey |
| ENSG00000113504 | B4GAT1 | grey |
| ENSG00000113790 | CD248 | grey |
| ENSG00000113812 | KDF1 | grey |
| ENSG00000114654 | B3GALT6 | grey |
| ENSG00000115290 | PHLDB3 | grey |
| ENSG00000115414 | LMNB2 | grey |
| ENSG00000115657 | FAM109B | grey |
| ENSG00000116031 | HASPIN | grey |
| ENSG00000116690 | CRACR2B | grey |
| ENSG00000116774 | ALS2CL | grey |
| ENSG00000117650 | BEND3 | grey |
| ENSG00000117791 | PFAS | grey |
| ENSG00000118523 | MAGED1 | grey |
| ENSG00000119227 | GEMIN4 | grey |
| ENSG00000119866 | PCED1B | grey |
| ENSG00000119946 | GIPC3 | grey |
| ENSG00000121053 | SLC25A42 | grey |
| ENSG00000121753 | TMIE | grey |
| ENSG00000121903 | P2RY8 | grey |
| ENSG00000124196 | C8orf33 | grey |
| ENSG00000124205 | LCK | grey |
| ENSG00000124507 | HMCES | grey |
| ENSG00000124564 | KLHL25 | grey |
| ENSG00000124780 | C9orf66 | grey |

| | | |
|---|---|---|
| ENSG00000125508 | NIPSNAP1 | grey |
| ENSG00000125551 | SCFD2 | grey |
| ENSG00000125740 | POU6F1 | grey |
| ENSG00000125895 | 9-Sep | grey |
| ENSG00000126016 | TNFAIP8L1 | grey |
| ENSG00000127022 | CALHM1 | grey |
| ENSG00000128578 | LRCH3 | grey |
| ENSG00000129204 | KRT73 | grey |
| ENSG00000129595 | CCDC30 | grey |
| ENSG00000131183 | NF2 | grey |
| ENSG00000131459 | TRABD2A | grey |
| ENSG00000131831 | IFT140 | grey |
| ENSG00000131941 | PRKAR1B | grey |
| ENSG00000132517 | TMEM201 | grey |
| ENSG00000132704 | SF3B3 | grey |
| ENSG00000132970 | FHIT | grey |
| ENSG00000134247 | WDR5 | grey |
| ENSG00000134824 | TCEAL3 | grey |
| ENSG00000134917 | AFAP1 | grey |
| ENSG00000135116 | ZNF512B | grey |
| ENSG00000135631 | ZSCAN16 | grey |
| ENSG00000135862 | METTL9 | grey |
| ENSG00000135931 | GAL3ST4 | grey |
| ENSG00000136098 | DYNC1H1 | grey |
| ENSG00000136274 | HTT | grey |
| ENSG00000137218 | SLC2A10 | grey |
| ENSG00000137507 | DPP4 | grey |
| ENSG00000137571 | SPTAN1 | grey |
| ENSG00000137731 | ADAMTSL2 | grey |
| ENSG00000137804 | MDS2 | grey |
| ENSG00000139540 | QRICH1 | grey |
| ENSG00000140284 | CARD11 | grey |
| ENSG00000140391 | ZNF69 | grey |
| ENSG00000140534 | ANKRD35 | grey |
| ENSG00000142494 | CD3E | grey |
| ENSG00000143061 | INF2 | grey |
| ENSG00000143147 | HIST2H2BF | grey |
| ENSG00000143157 | AL158151.1 | grey |
| ENSG00000143951 | PHACTR4 | grey |
| ENSG00000144158 | HLA-C | grey |
| ENSG00000144645 | AC083899.1 | grey |
| ENSG00000144681 | ZNF425 | grey |
| ENSG00000146005 | RNPS1 | grey |
| ENSG00000146072 | RNU1-106P | grey |
| ENSG00000146555 | IGLV3-12 | grey |
| ENSG00000147003 | TRAV2 | grey |
| ENSG00000147127 | TRAV3 | grey |
| ENSG00000148143 | TRAV12-1 | grey |

| | | |
|---|---|---|
| ENSG00000150594 | TRAV13-2 | grey |
| ENSG00000151090 | TRAV12-3 | grey |
| ENSG00000151632 | TRAV8-6 | grey |
| ENSG00000152463 | TRAV17 | grey |
| ENSG00000152760 | TRAV20 | grey |
| ENSG00000152952 | TRAV23DV6 | grey |
| ENSG00000153093 | TRAV26-1 | grey |
| ENSG00000153395 | TRAV29DV5 | grey |
| ENSG00000154240 | TRAV35 | grey |
| ENSG00000154545 | TRAV38-1 | grey |
| ENSG00000154803 | TRAV39 | grey |
| ENSG00000155026 | TRAV40 | grey |
| ENSG00000155093 | NUP62 | grey |
| ENSG00000156113 | RPL31P11 | grey |
| ENSG00000156564 | GIMAP1 | grey |
| ENSG00000157335 | LBH | grey |
| ENSG00000158483 | HNRNPA3P3 | grey |
| ENSG00000159247 | AC010168.1 | grey |
| ENSG00000159409 | RTL10 | grey |
| ENSG00000159640 | SIAH3 | grey |
| ENSG00000160183 | PDZK1P1 | grey |
| ENSG00000160200 | UHRF2P1 | grey |
| ENSG00000160224 | AC079630.1 | grey |
| ENSG00000160293 | LINC01671 | grey |
| ENSG00000160345 | LINC01504 | grey |
| ENSG00000160469 | AL161644.1 | grey |
| ENSG00000160683 | VDAC1P8 | grey |
| ENSG00000161021 | AL358473.1 | grey |
| ENSG00000161048 | DPY19L1P1 | grey |
| ENSG00000161640 | PATL2 | grey |
| ENSG00000162426 | MYOSLID | grey |
| ENSG00000162594 | PSMG3-AS1 | grey |
| ENSG00000162738 | AC007238.1 | grey |
| ENSG00000162817 | LEF1-AS1 | grey |
| ENSG00000163016 | AL139415.1 | grey |
| ENSG00000163161 | PTGES2-AS1 | grey |
| ENSG00000163380 | AC103563.7 | grey |
| ENSG00000163485 | AL139339.1 | grey |
| ENSG00000163633 | AC009495.3 | grey |
| ENSG00000163734 | MCF2L-AS1 | grey |
| ENSG00000164002 | OLMALINC | grey |
| ENSG00000164309 | AC009413.1 | grey |
| ENSG00000164512 | IRGM | grey |
| ENSG00000164659 | FAM200B | grey |
| ENSG00000164669 | PRKCQ-AS1 | grey |
| ENSG00000164989 | AL023653.1 | grey |
| ENSG00000165304 | ARPIN | grey |
| ENSG00000165730 | AL591895.1 | grey |

| | | |
|---|---|---|
| ENSG00000165828 | NME2 | grey |
| ENSG00000165996 | LINC01550 | grey |
| ENSG00000166246 | PGAM5 | grey |
| ENSG00000166342 | PVT1 | grey |
| ENSG00000166689 | TPM3P6 | grey |
| ENSG00000166763 | AC027031.2 | grey |
| ENSG00000166780 | HADHAP2 | grey |
| ENSG00000166831 | KRT73-AS1 | grey |
| ENSG00000166912 | LINC02295 | grey |
| ENSG00000167371 | SLC22A31 | grey |
| ENSG00000167720 | RBM15B | grey |
| ENSG00000168676 | AC099778.1 | grey |
| ENSG00000169083 | AC009041.1 | grey |
| ENSG00000169242 | AC009041.2 | grey |
| ENSG00000169372 | LINC00565 | grey |
| ENSG00000169607 | AL356489.2 | grey |
| ENSG00000169715 | AP003469.4 | grey |
| ENSG00000169964 | EPPK1 | grey |
| ENSG00000170356 | AC012213.3 | grey |
| ENSG00000170390 | RNF157-AS1 | grey |
| ENSG00000170442 | AC123912.2 | grey |
| ENSG00000170631 | AC116667.1 | grey |
| ENSG00000170889 | AC015802.5 | grey |
| ENSG00000170899 | AC022001.3 | grey |
| ENSG00000171408 | AC097376.2 | grey |
| ENSG00000171595 | AC009318.2 | grey |
| ENSG00000171766 | AC024909.3 | grey |
| ENSG00000171847 | RCC1L | grey |
| ENSG00000171848 | PRAG1 | grey |
| ENSG00000172215 | FCGBP | grey |
| ENSG00000172260 | AC026367.3 | grey |
| ENSG00000172361 | AL034550.2 | grey |
| ENSG00000172728 | TRAC | grey |
| ENSG00000173261 | AC010542.5 | grey |
| ENSG00000173546 | AC139768.1 | grey |
| ENSG00000173917 | SSTR3 | grey |
| ENSG00000174370 | AC002350.2 | grey |
| ENSG00000174697 | REXO5 | grey |
| ENSG00000174776 | PRSS21 | grey |
| ENSG00000175600 | GIPR | grey |
| ENSG00000175746 | CHI3L2 | grey |
| ENSG00000176884 | ARHGAP10 | grey |
| ENSG00000176890 | IL5RA | grey |
| ENSG00000177098 | CEBPE | grey |
| ENSG00000177181 | SMPD3 | grey |
| ENSG00000177324 | PRSS33 | grey |
| ENSG00000177453 | CLC | grey |
| ENSG00000177606 | SIGLEC8 | grey |

| | | |
|---|---|---|
| ENSG00000177807 | ITGB8 | grey |
| ENSG00000178184 | PMP22 | grey |
| ENSG00000178201 | SLC29A1 | grey |
| ENSG00000178752 | THBS4 | grey |
| ENSG00000178860 | IL4 | grey |
| ENSG00000179029 | PYROXD2 | grey |
| ENSG00000179477 | ZNF835 | grey |
| ENSG00000179913 | CACNG6 | grey |
| ENSG00000180229 | AKAP12 | grey |
| ENSG00000180764 | LGALS12 | grey |
| ENSG00000180767 | LYVE1 | grey |
| ENSG00000180834 | SEMA7A | grey |
| ENSG00000180900 | SORD | grey |
| ENSG00000180914 | HDC | grey |
| ENSG00000181023 | CACNG8 | grey |
| ENSG00000181234 | PPFIA4 | grey |
| ENSG00000181291 | TAMM41 | grey |
| ENSG00000182484 | MS4A2 | grey |
| ENSG00000183020 | INPP1 | grey |
| ENSG00000183077 | CNKSR3 | grey |
| ENSG00000183780 | KCTD15 | grey |
| ENSG00000183837 | ENPP3 | grey |
| ENSG00000183873 | IL34 | grey |
| ENSG00000183955 | FAM46B | grey |
| ENSG00000184106 | SLC45A3 | grey |
| ENSG00000184385 | TFF3 | grey |
| ENSG00000184785 | ALOX15 | grey |
| ENSG00000184905 | ACOT11 | grey |
| ENSG00000184985 | CPA3 | grey |
| ENSG00000185052 | CORO6 | grey |
| ENSG00000185055 | HRASLS5 | grey |
| ENSG00000185304 | LEP | grey |
| ENSG00000185839 | WDR49 | grey |
| ENSG00000186188 | SUGCT | grey |
| ENSG00000186235 | C15orf54 | grey |
| ENSG00000186301 | GRIN1 | grey |
| ENSG00000186314 | TYMS | grey |
| ENSG00000186715 | SCN4B | grey |
| ENSG00000186889 | RIMKLA | grey |
| ENSG00000187010 | BEND2 | grey |
| ENSG00000187122 | NIM1K | grey |
| ENSG00000187243 | JUN | grey |
| ENSG00000187391 | KCNJ10 | grey |
| ENSG00000187627 | PARD6G | grey |
| ENSG00000187690 | VN1R1 | grey |
| ENSG00000187951 | ERFE | grey |
| ENSG00000188076 | MSC | grey |
| ENSG00000188312 | TMEM107 | grey |

| | | |
|---|---|---|
| ENSG00000188761 | GATA2 | grey |
| ENSG00000188981 | ALOX12B | grey |
| ENSG00000189233 | ZBTB42 | grey |
| ENSG00000189253 | B3GNT3 | grey |
| ENSG00000189419 | HERC2P3 | grey |
| ENSG00000196092 | PIPSL | grey |
| ENSG00000196151 | CHST13 | grey |
| ENSG00000196159 | MAP6D1 | grey |
| ENSG00000196167 | SCRIB | grey |
| ENSG00000196208 | OXTR | grey |
| ENSG00000196814 | OR56B1 | grey |
| ENSG00000197702 | TMEM132C | grey |
| ENSG00000197888 | TMEM132E | grey |
| ENSG00000198169 | BACE2 | grey |
| ENSG00000198331 | WASH6P | grey |
| ENSG00000198576 | SPNS3 | grey |
| ENSG00000198626 | AP2A2 | grey |
| ENSG00000198680 | AFMID | grey |
| ENSG00000198753 | PTGDR2 | grey |
| ENSG00000198848 | SLC35F3 | grey |
| ENSG00000203588 | PNMA3 | grey |
| ENSG00000204351 | SCN5A | grey |
| ENSG00000204381 | KMT5A | grey |
| ENSG00000204439 | TREML3P | grey |
| ENSG00000204577 | OLIG1 | grey |
| ENSG00000204624 | UMODL1-AS1 | grey |
| ENSG00000204681 | SMIM10 | grey |
| ENSG00000204710 | TCEAL2 | grey |
| ENSG00000205038 | SORCS2 | grey |
| ENSG00000205531 | SLC24A3 | grey |
| ENSG00000205611 | EFCAB10 | grey |
| ENSG00000205663 | RGPD2 | grey |
| ENSG00000206228 | AL035411.1 | grey |
| ENSG00000206557 | FFAR4 | grey |
| ENSG00000206885 | AC016757.1 | grey |
| ENSG00000208036 | MST1P2 | grey |
| ENSG00000211746 | PRELID2 | grey |
| ENSG00000211749 | MST1L | grey |
| ENSG00000211750 | TMEM17 | grey |
| ENSG00000211752 | RHD | grey |
| ENSG00000211753 | SLIT1 | grey |
| ENSG00000211766 | MAGED4B | grey |
| ENSG00000211817 | MAGI2 | grey |
| ENSG00000211820 | RGPD1 | grey |
| ENSG00000212747 | CXorf67 | grey |
| ENSG00000212978 | ARHGAP11B | grey |
| ENSG00000213453 | SCGB1C1 | grey |
| ENSG00000213542 | CENPP | grey |

| | | |
|---|---|---|
| ENSG00000213557 | BCL2L15 | grey |
| ENSG00000213569 | MSANTD1 | grey |
| ENSG00000213640 | NUGGC | grey |
| ENSG00000214189 | TRIM64B | grey |
| ENSG00000214226 | SPATA41 | grey |
| ENSG00000214465 | PAX5 | grey |
| ENSG00000214700 | WDSUB1 | grey |
| ENSG00000214719 | FAT4 | grey |
| ENSG00000215187 | COLCA1 | grey |
| ENSG00000215190 | GREB1 | grey |
| ENSG00000215482 | SRGAP3 | grey |
| ENSG00000215835 | MVB12B | grey |
| ENSG00000215840 | PARVA | grey |
| ENSG00000215861 | UGT2B17 | grey |
| ENSG00000218052 | ZNF251 | grey |
| ENSG00000218631 | HYLS1 | grey |
| ENSG00000220614 | ARC | grey |
| ENSG00000221164 | RYR2 | grey |
| ENSG00000221510 | TUSC1 | grey |
| ENSG00000222019 | PLXNB3 | grey |
| ENSG00000223508 | COLGALT2 | grey |
| ENSG00000223612 | CES1 | grey |
| ENSG00000223886 | IGBP1-AS1 | grey |
| ENSG00000224034 | SYT15 | grey |
| ENSG00000224183 | SKIV2L | grey |
| ENSG00000224216 | LAYN | grey |
| ENSG00000224914 | C6orf47 | grey |
| ENSG00000225163 | TRIM51BP | grey |
| ENSG00000225231 | LILRB3 | grey |
| ENSG00000225335 | DISP3 | grey |
| ENSG00000225361 | GABBR1 | grey |
| ENSG00000225411 | SPDYC | grey |
| ENSG00000225612 | PKHD1L1 | grey |
| ENSG00000225762 | NAP1L4 | grey |
| ENSG00000225932 | LINC01597 | grey |
| ENSG00000225972 | FAM239B | grey |
| ENSG00000226025 | OLIG2 | grey |
| ENSG00000226054 | HNRNPA1P4 | grey |
| ENSG00000226085 | CD200R1L | grey |
| ENSG00000226102 | TRIM71 | grey |
| ENSG00000226245 | SNORA75 | grey |
| ENSG00000226259 | MIR106B | grey |
| ENSG00000226445 | TRBV19 | grey |
| ENSG00000226571 | TRBV23-1 | grey |
| ENSG00000226660 | TRBV24-1 | grey |
| ENSG00000226816 | TRBV27 | grey |
| ENSG00000226935 | TRBV28 | grey |
| ENSG00000227036 | TRBJ2-2P | grey |

| | | |
|---|---|---|
| ENSG00000227073 | TRAV38-2DV8 | grey |
| ENSG00000227077 | TRAV41 | grey |
| ENSG00000227719 | RTL8B | grey |
| ENSG00000227934 | AC016747.1 | grey |
| ENSG00000227953 | FTH1P3 | grey |
| ENSG00000228265 | AC007000.1 | grey |
| ENSG00000228335 | AC068050.1 | grey |
| ENSG00000228526 | AL390039.1 | grey |
| ENSG00000228653 | EEF1DP4 | grey |
| ENSG00000228800 | ZNF788 | grey |
| ENSG00000228960 | C17orf67 | grey |
| ENSG00000229017 | SMARCE1P6 | grey |
| ENSG00000229204 | C12orf71 | grey |
| ENSG00000229409 | AC005562.1 | grey |
| ENSG00000229417 | PRSS41 | grey |
| ENSG00000229659 | FAM166B | grey |
| ENSG00000230006 | LINC00680 | grey |
| ENSG00000230177 | CALM2P3 | grey |
| ENSG00000230306 | AL596087.1 | grey |
| ENSG00000230397 | AL592295.1 | grey |
| ENSG00000230568 | AC245297.1 | grey |
| ENSG00000231503 | ADAMTS7P4 | grey |
| ENSG00000232063 | AL117344.1 | grey |
| ENSG00000232150 | Z85996.1 | grey |
| ENSG00000232389 | AL583834.1 | grey |
| ENSG00000232485 | SNORA11F | grey |
| ENSG00000232553 | MIR548O | grey |
| ENSG00000233093 | URAHP | grey |
| ENSG00000233429 | RPL23AP53 | grey |
| ENSG00000233547 | AC241585.1 | grey |
| ENSG00000233609 | AC073073.1 | grey |
| ENSG00000233922 | LINC02561 | grey |
| ENSG00000234062 | SDHDP6 | grey |
| ENSG00000234425 | AC234781.1 | grey |
| ENSG00000234558 | LINC00863 | grey |
| ENSG00000234776 | LINC00618 | grey |
| ENSG00000234785 | LINC02470 | grey |
| ENSG00000234814 | AC016027.1 | grey |
| ENSG00000234937 | PPP1R26-AS1 | grey |
| ENSG00000235084 | CR786580.1 | grey |
| ENSG00000235111 | AC099552.2 | grey |
| ENSG00000235150 | LINC01389 | grey |
| ENSG00000235169 | CTAGE4 | grey |
| ENSG00000235172 | MTND1P23 | grey |
| ENSG00000235257 | LGALS17A | grey |
| ENSG00000235505 | MEMO1P1 | grey |
| ENSG00000235527 | UQCRFS1P1 | grey |
| ENSG00000235554 | SEPT7P3 | grey |

| | | |
|---|---|---|
| ENSG00000235734 | ZNF32-AS1 | grey |
| ENSG00000235847 | GTF2H2B | grey |
| ENSG00000235989 | BX322234.1 | grey |
| ENSG00000236137 | AL158850.1 | grey |
| ENSG00000236875 | TRBV2 | grey |
| ENSG00000237133 | AC005082.1 | grey |
| ENSG00000237753 | LINC00161 | grey |
| ENSG00000237868 | LINC00511 | grey |
| ENSG00000237883 | SDHDP2 | grey |
| ENSG00000237989 | AC107983.1 | grey |
| ENSG00000238083 | AC006042.1 | grey |
| ENSG00000238084 | AL732414.1 | grey |
| ENSG00000238249 | LINC01341 | grey |
| ENSG00000239203 | RALY-AS1 | grey |
| ENSG00000239636 | AC073063.1 | grey |
| ENSG00000240291 | MIR34AHG | grey |
| ENSG00000240350 | HNRNPCP7 | grey |
| ENSG00000240563 | AL590068.1 | grey |
| ENSG00000241560 | OR2A9P | grey |
| ENSG00000241644 | LINC01277 | grey |
| ENSG00000241657 | PTGES3P3 | grey |
| ENSG00000242110 | AL117328.1 | grey |
| ENSG00000242220 | NPM1P25 | grey |
| ENSG00000243155 | RPL26P6 | grey |
| ENSG00000243244 | ANKRD36BP2 | grey |
| ENSG00000243304 | AL080317.1 | grey |
| ENSG00000243629 | BANF1P2 | grey |
| ENSG00000243709 | SPTLC1P1 | grey |
| ENSG00000243795 | SF3A3P1 | grey |
| ENSG00000243859 | PTMAP4 | grey |
| ENSG00000243960 | AL691447.2 | grey |
| ENSG00000244198 | ST13P4 | grey |
| ENSG00000245080 | AL583856.1 | grey |
| ENSG00000245468 | AC098820.1 | grey |
| ENSG00000245614 | CLK2P1 | grey |
| ENSG00000246214 | LINC00892 | grey |
| ENSG00000246228 | HOTAIRM1 | grey |
| ENSG00000247240 | AL158212.2 | grey |
| ENSG00000247498 | RPL10P19 | grey |
| ENSG00000248278 | LINC01694 | grey |
| ENSG00000248677 | AL390879.2 | grey |
| ENSG00000249042 | AL138930.1 | grey |
| ENSG00000249572 | API5P1 | grey |
| ENSG00000249684 | C11orf94 | grey |
| ENSG00000250155 | EEF1GP5 | grey |
| ENSG00000250254 | SVILP1 | grey |
| ENSG00000250444 | AL139128.1 | grey |
| ENSG00000250508 | CHCHD2P6 | grey |

| | | |
|---|---|---|
| ENSG00000251235 | Z97192.3 | grey |
| ENSG00000252481 | RCBTB2P1 | grey |
| ENSG00000252916 | SMIM1 | grey |
| ENSG00000253293 | LINC01366 | grey |
| ENSG00000253368 | ITGA9-AS1 | grey |
| ENSG00000253552 | AP002004.1 | grey |
| ENSG00000253559 | HIPK1-AS1 | grey |
| ENSG00000253704 | AC005822.1 | grey |
| ENSG00000253930 | HMGN1P36 | grey |
| ENSG00000253955 | LDHAP7 | grey |
| ENSG00000254449 | MORC2-AS1 | grey |
| ENSG00000254473 | AL445231.1 | grey |
| ENSG00000254521 | DDX11L5 | grey |
| ENSG00000254598 | AC020594.1 | grey |
| ENSG00000254676 | AC079922.2 | grey |
| ENSG00000254777 | AC097533.1 | grey |
| ENSG00000254827 | DGUOK-AS1 | grey |
| ENSG00000254997 | LINC01679 | grey |
| ENSG00000255062 | LRRC37A2 | grey |
| ENSG00000255122 | AL031284.1 | grey |
| ENSG00000255189 | HMGN2P17 | grey |
| ENSG00000255274 | AC093484.1 | grey |
| ENSG00000255397 | AC004865.2 | grey |
| ENSG00000255874 | AL450384.2 | grey |
| ENSG00000256238 | AC017002.3 | grey |
| ENSG00000256262 | L1TD1 | grey |
| ENSG00000256347 | ZBTB20-AS1 | grey |
| ENSG00000257103 | INMT | grey |
| ENSG00000257156 | TRBV11-2 | grey |
| ENSG00000257175 | AMACR | grey |
| ENSG00000257681 | TCP10L | grey |
| ENSG00000257740 | AL162431.2 | grey |
| ENSG00000258443 | STON1 | grey |
| ENSG00000258659 | AC008494.1 | grey |
| ENSG00000258689 | LINC00880 | grey |
| ENSG00000258826 | LEFTY1 | grey |
| ENSG00000258999 | LINC02044 | grey |
| ENSG00000259062 | RPL5P17 | grey |
| ENSG00000259268 | AL390195.1 | grey |
| ENSG00000259295 | AC004889.1 | grey |
| ENSG00000259319 | AC068189.1 | grey |
| ENSG00000259483 | LINC02447 | grey |
| ENSG00000259516 | DDX11-AS1 | grey |
| ENSG00000259583 | AC022113.1 | grey |
| ENSG00000259838 | CASC8 | grey |
| ENSG00000260075 | UBL7-AS1 | grey |
| ENSG00000260083 | GPRC5D-AS1 | grey |
| ENSG00000260176 | SUMO2P17 | grey |

| | | |
|---|---|---|
| ENSG00000260300 | LINC02102 | grey |
| ENSG00000260456 | AC008771.1 | grey |
| ENSG00000260572 | AC034231.1 | grey |
| ENSG00000260625 | AC106795.2 | grey |
| ENSG00000260711 | AC008957.1 | grey |
| ENSG00000260792 | PTTG2 | grey |
| ENSG00000260868 | CCT5P1 | grey |
| ENSG00000260923 | AP000808.1 | grey |
| ENSG00000261251 | SNRPCP2 | grey |
| ENSG00000261327 | SCARNA13 | grey |
| ENSG00000261377 | RNU6-762P | grey |
| ENSG00000261604 | HOXA10 | grey |
| ENSG00000261707 | TRNP1 | grey |
| ENSG00000261801 | HOXA-AS2 | grey |
| ENSG00000261889 | OSGEPL1-AS1 | grey |
| ENSG00000262228 | AC023632.2 | grey |
| ENSG00000262587 | TNFRSF10A-AS1 | grey |
| ENSG00000262692 | AC008663.2 | grey |
| ENSG00000263535 | AP002765.1 | grey |
| ENSG00000263606 | AL354920.1 | grey |
| ENSG00000263731 | SIGLEC12 | grey |
| ENSG00000263823 | CSNK2A3 | grey |
| ENSG00000263990 | AP000873.4 | grey |
| ENSG00000264954 | AC022182.1 | grey |
| ENSG00000264964 | SLC22A18AS | grey |
| ENSG00000265681 | KRTAP5-9 | grey |
| ENSG00000266075 | AP001318.3 | grey |
| ENSG00000267082 | AC068587.4 | grey |
| ENSG00000267226 | GLYATL1P1 | grey |
| ENSG00000267366 | TMPRSS4-AS1 | grey |
| ENSG00000267453 | AC022182.3 | grey |
| ENSG00000267868 | LINC00346 | grey |
| ENSG00000268336 | SUPT16HP1 | grey |
| ENSG00000268362 | USP30-AS1 | grey |
| ENSG00000268798 | OR8R1P | grey |
| ENSG00000269489 | LSM14A | grey |
| ENSG00000269894 | AC024909.1 | grey |
| ENSG00000269904 | CR383656.2 | grey |
| ENSG00000269949 | AC025265.1 | grey |
| ENSG00000270091 | AC073896.3 | grey |
| ENSG00000270194 | AC005225.1 | grey |
| ENSG00000270362 | TRIM34 | grey |
| ENSG00000270574 | LINC01269 | grey |
| ENSG00000270673 | AL157955.2 | grey |
| ENSG00000270948 | AL136040.1 | grey |
| ENSG00000270951 | ACTN1-AS1 | grey |
| ENSG00000270953 | AC007950.1 | grey |
| ENSG00000270956 | CSPG4P12 | grey |

| | | |
|---|---|---|
| ENSG00000271265 | AF111167.2 | grey |
| ENSG00000271303 | AL355073.1 | grey |
| ENSG00000271335 | ANP32AP1 | grey |
| ENSG00000271550 | AC015712.2 | grey |
| ENSG00000271553 | ELOCP2 | grey |
| ENSG00000271732 | AC217777.1 | grey |
| ENSG00000271752 | MIR762HG | grey |
| ENSG00000271780 | AC141586.2 | grey |
| ENSG00000271833 | AC009119.2 | grey |
| ENSG00000271936 | C16orf95 | grey |
| ENSG00000271993 | AC069224.1 | grey |
| ENSG00000272008 | AC026471.2 | grey |
| ENSG00000272092 | AL121839.2 | grey |
| ENSG00000272142 | LINC02280 | grey |
| ENSG00000272195 | LINC01960 | grey |
| ENSG00000272264 | LINC02193 | grey |
| ENSG00000272308 | Z97055.2 | grey |
| ENSG00000272417 | AC134312.5 | grey |
| ENSG00000272420 | PDCD6IPP2 | grey |
| ENSG00000272432 | AC114947.2 | grey |
| ENSG00000272509 | AC092134.1 | grey |
| ENSG00000272620 | LOXL1-AS1 | grey |
| ENSG00000272690 | AC108134.2 | grey |
| ENSG00000272720 | AC087392.3 | grey |
| ENSG00000272779 | AC133552.2 | grey |
| ENSG00000273026 | AC116914.2 | grey |
| ENSG00000273062 | AC134669.1 | grey |
| ENSG00000273183 | AP000919.1 | grey |
| ENSG00000273226 | AC145207.5 | grey |
| ENSG00000273240 | AC009831.1 | grey |
| ENSG00000273432 | AC004253.1 | grey |
| ENSG00000273445 | PRR29-AS1 | grey |
| ENSG00000273483 | AP001033.1 | grey |
| ENSG00000273487 | RPL17 | grey |
| ENSG00000273542 | RN7SL574P | grey |
| ENSG00000273628 | AC011472.1 | grey |
| ENSG00000273702 | AC104971.2 | grey |
| ENSG00000274026 | AP005131.2 | grey |
| ENSG00000274191 | LINC01835 | grey |
| ENSG00000274256 | AL356740.1 | grey |
| ENSG00000274386 | SIGLEC20P | grey |
| ENSG00000274487 | AC092279.1 | grey |
| ENSG00000275401 | AC027307.3 | grey |
| ENSG00000275457 | AL589765.6 | grey |
| ENSG00000275491 | AC018809.1 | grey |
| ENSG00000275703 | MAP2K4P1 | grey |
| ENSG00000275719 | AC069307.1 | grey |
| ENSG00000275763 | AC015726.1 | grey |

| | | |
|---|---|---|
| ENSG00000275963 | AC097359.2 | grey |
| ENSG00000275993 | HMGN3-AS1 | grey |
| ENSG00000276557 | AC010680.2 | grey |
| ENSG00000276966 | YTHDF3-AS1 | grey |
| ENSG00000277283 | MTDHP1 | grey |
| ENSG00000277879 | AL121917.2 | grey |
| ENSG00000278017 | AC007938.3 | grey |
| ENSG00000278356 | AC009948.4 | grey |
| ENSG00000278416 | AL355297.3 | grey |
| ENSG00000278611 | SRXN1 | grey |
| ENSG00000278627 | AL117336.3 | grey |
| ENSG00000278635 | BNIP3P11 | grey |
| ENSG00000278668 | AC018638.7 | grey |
| ENSG00000278730 | AL137798.1 | grey |
| ENSG00000278816 | AC112187.3 | grey |
| ENSG00000279026 | AL118558.3 | grey |
| ENSG00000279031 | AL445222.1 | grey |
| ENSG00000279082 | AC012073.1 | grey |
| ENSG00000279108 | AC126118.1 | grey |
| ENSG00000279148 | AL139274.2 | grey |
| ENSG00000279196 | AC087623.3 | grey |
| ENSG00000279233 | AL359643.3 | grey |
| ENSG00000279254 | AL356512.1 | grey |
| ENSG00000279357 | AC009686.2 | grey |
| ENSG00000279617 | AC104113.1 | grey |
| ENSG00000279656 | AC034229.4 | grey |
| ENSG00000279692 | AL513477.2 | grey |
| ENSG00000279863 | AL031432.3 | grey |
| ENSG00000280011 | AC087752.4 | grey |
| ENSG00000280047 | AFAP1-AS1 | grey |
| ENSG00000280120 | LINC02018 | grey |
| ENSG00000280156 | AL022322.1 | grey |
| ENSG00000280161 | AC245060.4 | grey |
| ENSG00000280184 | AL358472.3 | grey |
| ENSG00000280193 | AL449106.1 | grey |
| ENSG00000280195 | AC093726.2 | grey |
| ENSG00000280953 | AL391834.2 | grey |
| ENSG00000281741 | AC013468.1 | grey |
| ENSG00000281912 | AC004951.4 | grey |
| ENSG00000282885 | AC133644.2 | grey |
| ENSG00000283403 | AL354760.1 | grey |
| ENSG00000005189 | AC104836.2 | magenta |
| ENSG00000007038 | HIST1H4K | magenta |
| ENSG00000010310 | AL354798.1 | magenta |
| ENSG00000064886 | AC091271.1 | magenta |
| ENSG00000071205 | FAM27E3 | magenta |
| ENSG00000091181 | AC026333.4 | magenta |
| ENSG00000092067 | AL391903.2 | magenta |

| | | |
|---|---|---|
| ENSG00000103056 | TMEM269 | magenta |
| ENSG00000103355 | AC244154.1 | magenta |
| ENSG00000105205 | AL391095.3 | magenta |
| ENSG00000105366 | AL117332.1 | magenta |
| ENSG00000105855 | LINC01730 | magenta |
| ENSG00000109099 | U47924.3 | magenta |
| ENSG00000112759 | AC008622.2 | magenta |
| ENSG00000113296 | C18orf65 | magenta |
| ENSG00000113520 | AC008115.4 | magenta |
| ENSG00000119943 | CU639417.2 | magenta |
| ENSG00000127903 | AC244472.3 | magenta |
| ENSG00000130433 | HIST1H4E | magenta |
| ENSG00000131016 | AC004812.2 | magenta |
| ENSG00000133317 | AL391988.1 | magenta |
| ENSG00000133800 | AC064801.1 | magenta |
| ENSG00000138623 | AC005911.1 | magenta |
| ENSG00000140263 | PMS2P2 | magenta |
| ENSG00000140287 | AC008567.3 | magenta |
| ENSG00000142408 | AC005962.2 | magenta |
| ENSG00000143847 | AC141557.2 | magenta |
| ENSG00000144559 | AC005899.7 | magenta |
| ENSG00000149534 | AC005332.9 | magenta |
| ENSG00000151689 | AL121890.5 | magenta |
| ENSG00000153721 | AC005225.5 | magenta |
| ENSG00000153885 | AC004232.3 | magenta |
| ENSG00000154269 | LINC01727 | magenta |
| ENSG00000157368 | AC008537.3 | magenta |
| ENSG00000158246 | AC126474.1 | magenta |
| ENSG00000158715 | AC135048.4 | magenta |
| ENSG00000160180 | AC122688.3 | magenta |
| ENSG00000161905 | AC020604.1 | magenta |
| ENSG00000162390 | AC007224.2 | magenta |
| ENSG00000163751 | AC005796.1 | magenta |
| ENSG00000167549 | AL132780.4 | magenta |
| ENSG00000168004 | AC110285.7 | magenta |
| ENSG00000179348 | AC069547.1 | magenta |
| ENSG00000179627 | AL031595.2 | magenta |
| ENSG00000182240 | AC091825.1 | magenta |
| ENSG00000182557 | AC073857.1 | magenta |
| ENSG00000183134 | AC006548.3 | magenta |
| ENSG00000184221 | AC022413.1 | magenta |
| ENSG00000196220 | AL023806.3 | magenta |
| ENSG00000198756 | AC132219.2 | magenta |
| ENSG00000204176 | AC245140.2 | magenta |
| ENSG00000204455 | LINC01163 | magenta |
| ENSG00000205927 | AC241377.3 | magenta |
| ENSG00000206531 | LINC01144 | magenta |
| ENSG00000215148 | AL627171.2 | magenta |

| | | |
|---|---|---|
| ENSG00000220349 | AC005394.2 | magenta |
| ENSG00000228549 | SLC4A1 | magenta |
| ENSG00000234493 | MPO | magenta |
| ENSG00000236204 | ITGA2B | magenta |
| ENSG00000237706 | GTF2IRD1 | magenta |
| ENSG00000248126 | NFIX | magenta |
| ENSG00000249637 | MRC2 | magenta |
| ENSG00000254226 | TSPAN9 | magenta |
| ENSG00000255587 | LTF | magenta |
| ENSG00000264695 | SLC25A39 | magenta |
| ENSG00000272040 | DDX11 | magenta |
| ENSG00000273759 | SLC38A5 | magenta |
| ENSG00000276231 | VSIG2 | magenta |
| ENSG00000282608 | RNF10 | magenta |
| ENSG00000282850 | ANK1 | magenta |
| ENSG00000282933 | EFCAB1 | magenta |
| ENSG00000283399 | MYOM2 | magenta |
| ENSG00000006704 | CDH1 | pink |
| ENSG00000041515 | MYO16 | pink |
| ENSG00000048462 | XK | pink |
| ENSG00000075035 | TNFRSF17 | pink |
| ENSG00000078900 | NEDD4L | pink |
| ENSG00000099251 | FSTL4 | pink |
| ENSG00000100473 | PITHD1 | pink |
| ENSG00000101057 | YBX3 | pink |
| ENSG00000103089 | MRPS24 | pink |
| ENSG00000105376 | HAGH | pink |
| ENSG00000110777 | TBC1D22B | pink |
| ENSG00000111291 | MYLK | pink |
| ENSG00000111665 | COL17A1 | pink |
| ENSG00000112320 | YBX1 | pink |
| ENSG00000117090 | FECH | pink |
| ENSG00000117399 | SPTB | pink |
| ENSG00000120875 | TP63 | pink |
| ENSG00000123095 | IGF2BP2 | pink |
| ENSG00000128268 | WSCD2 | pink |
| ENSG00000132465 | REXO2 | pink |
| ENSG00000133328 | MBNL3 | pink |
| ENSG00000134061 | RAP1GAP | pink |
| ENSG00000135452 | TP73 | pink |
| ENSG00000142945 | TNS1 | pink |
| ENSG00000148225 | STRADB | pink |
| ENSG00000148773 | ITGB5 | pink |
| ENSG00000153064 | CTTN | pink |
| ENSG00000156110 | TTC39A | pink |
| ENSG00000164330 | EIF2AK1 | pink |
| ENSG00000165409 | HBQ1 | pink |
| ENSG00000166435 | CEACAM6 | pink |

| | | |
|---|---|---|
| ENSG00000166819 | ACHE | pink |
| ENSG00000167476 | SMOX | pink |
| ENSG00000168070 | TESC | pink |
| ENSG00000169116 | BLVRB | pink |
| ENSG00000170476 | PTGS1 | pink |
| ENSG00000170509 | CRISP3 | pink |
| ENSG00000176658 | EFHC1 | pink |
| ENSG00000178445 | HSD17B7P2 | pink |
| ENSG00000183010 | CDC34 | pink |
| ENSG00000183605 | SEC14L3 | pink |
| ENSG00000184661 | SRRD | pink |
| ENSG00000184986 | FBXO7 | pink |
| ENSG00000186081 | ASCC2 | pink |
| ENSG00000188199 | ST13 | pink |
| ENSG00000203876 | CTSG | pink |
| ENSG00000211592 | COCH | pink |
| ENSG00000211593 | PLEK2 | pink |
| ENSG00000211594 | MYBL2 | pink |
| ENSG00000211598 | BPI | pink |
| ENSG00000211625 | TNNC2 | pink |
| ENSG00000211637 | GATA1 | pink |
| ENSG00000211639 | OLFM4 | pink |
| ENSG00000211640 | TSNAXIP1 | pink |
| ENSG00000211644 | FA2H | pink |
| ENSG00000211648 | NPRL3 | pink |
| ENSG00000211649 | SEC14L5 | pink |
| ENSG00000211651 | SLC7A5 | pink |
| ENSG00000211652 | GSPT1 | pink |
| ENSG00000211653 | TMC5 | pink |
| ENSG00000211660 | KLC3 | pink |
| ENSG00000211662 | LYL1 | pink |
| ENSG00000211663 | ICAM5 | pink |
| ENSG00000211664 | SIGLEC6 | pink |
| ENSG00000211668 | CABP5 | pink |
| ENSG00000211669 | KLF1 | pink |
| ENSG00000211670 | FKBP8 | pink |
| ENSG00000211672 | SERPINE1 | pink |
| ENSG00000211673 | BAG1 | pink |
| ENSG00000211677 | RUNDC3A | pink |
| ENSG00000211679 | SLC6A4 | pink |
| ENSG00000211890 | SH3D19 | pink |
| ENSG00000211892 | SOX6 | pink |
| ENSG00000211893 | POU2AF1 | pink |
| ENSG00000211895 | VWF | pink |
| ENSG00000211896 | ACSS3 | pink |
| ENSG00000211897 | GPRC5D | pink |
| ENSG00000211933 | CDCA3 | pink |
| ENSG00000211934 | CHPT1 | pink |

| | | |
|---|---|---|
| ENSG00000211935 | RSPH4A | pink |
| ENSG00000211937 | GCNT2 | pink |
| ENSG00000211938 | RHAG | pink |
| ENSG00000211943 | MDGA1 | pink |
| ENSG00000211945 | TSPO2 | pink |
| ENSG00000211947 | KHDRBS2 | pink |
| ENSG00000211949 | SOBP | pink |
| ENSG00000211955 | PHF1 | pink |
| ENSG00000215914 | PRSS16 | pink |
| ENSG00000224041 | SPARC | pink |
| ENSG00000224650 | HGD | pink |
| ENSG00000225986 | ODC1 | pink |
| ENSG00000228140 | GADD45A | pink |
| ENSG00000231613 | SLAMF1 | pink |
| ENSG00000231672 | SLC2A1 | pink |
| ENSG00000232727 | CDC20 | pink |
| ENSG00000236773 | MMP8 | pink |
| ENSG00000237352 | SGIP1 | pink |
| ENSG00000239264 | CTNNAL1 | pink |
| ENSG00000239571 | BBOF1 | pink |
| ENSG00000239951 | NRDE2 | pink |
| ENSG00000239975 | FKBP1B | pink |
| ENSG00000241566 | MXI1 | pink |
| ENSG00000242534 | DUSP4 | pink |
| ENSG00000243466 | CLU | pink |
| ENSG00000244437 | FAM117A | pink |
| ENSG00000246560 | KIAA1191 | pink |
| ENSG00000251546 | BHLHE41 | pink |
| ENSG00000253755 | FAM210B | pink |
| ENSG00000254709 | CEACAM8 | pink |
| ENSG00000258572 | HIST1H2BJ | pink |
| ENSG00000267150 | EMC3 | pink |
| ENSG00000267270 | TNFSF9 | pink |
| ENSG00000267309 | GNAZ | pink |
| ENSG00000269940 | MGAT3 | pink |
| ENSG00000270503 | ATP1B2 | pink |
| ENSG00000270550 | SH3BP4 | pink |
| ENSG00000272777 | XPO7 | pink |
| ENSG00000273355 | PLVAP | pink |
| ENSG00000273474 | OLFM1 | pink |
| ENSG00000273507 | HBZ | pink |
| ENSG00000273987 | SLC6A8 | pink |
| ENSG00000274576 | LLGL1 | pink |
| ENSG00000275476 | JCHAIN | pink |
| ENSG00000278196 | LPIN3 | pink |
| ENSG00000278857 | RBM38 | pink |
| ENSG00000278985 | CHIT1 | pink |
| ENSG00000005381 | TMCC2 | purple |

| | | |
|---|---|---|
| ENSG00000012223 | HRASLS2 | purple |
| ENSG00000065618 | SEC14L4 | purple |
| ENSG00000086548 | MKRN1 | purple |
| ENSG00000096006 | CA1 | purple |
| ENSG00000100448 | CD180 | purple |
| ENSG00000101425 | TPGS2 | purple |
| ENSG00000102837 | TSPAN31 | purple |
| ENSG00000112812 | GYPC | purple |
| ENSG00000118113 | C9orf78 | purple |
| ENSG00000124469 | ST6GALNAC4 | purple |
| ENSG00000131899 | TMOD1 | purple |
| ENSG00000133063 | DPM2 | purple |
| ENSG00000141161 | HEMGN | purple |
| ENSG00000142583 | PIM1 | purple |
| ENSG00000148346 | GMPR | purple |
| ENSG00000157554 | SMAD6 | purple |
| ENSG00000162877 | BCAR3 | purple |
| ENSG00000164047 | STON2 | purple |
| ENSG00000164821 | PCSK6 | purple |
| ENSG00000170801 | GID4 | purple |
| ENSG00000173391 | RANBP10 | purple |
| ENSG00000178665 | UNC45B | purple |
| ENSG00000179869 | GATA6 | purple |
| ENSG00000182551 | CARM1 | purple |
| ENSG00000187952 | SLC2A5 | purple |
| ENSG00000188599 | KIF2C | purple |
| ENSG00000188612 | PTPRF | purple |
| ENSG00000206047 | HDGF | purple |
| ENSG00000213385 | SELENBP1 | purple |
| ENSG00000224728 | DYRK3 | purple |
| ENSG00000230359 | AQP10 | purple |
| ENSG00000233968 | GUK1 | purple |
| ENSG00000234336 | SOX13 | purple |
| ENSG00000239839 | FANCD2 | purple |
| ENSG00000240247 | ACKR2 | purple |
| ENSG00000241157 | CTDSPL | purple |
| ENSG00000242852 | SNCA | purple |
| ENSG00000255733 | SFRP2 | purple |
| ENSG00000256164 | SLC25A37 | purple |
| ENSG00000258116 | ALAD | purple |
| ENSG00000267040 | WDR31 | purple |
| ENSG00000270030 | LCN2 | purple |
| ENSG00000271851 | PROSER2 | purple |
| ENSG00000272791 | PARD3 | purple |
| ENSG00000273245 | MKI67 | purple |
| ENSG00000277149 | RGS10 | purple |
| ENSG00000279332 | GLB1L2 | purple |
| ENSG00000283646 | ESAM | purple |

| | | |
|---|---|---|
| ENSG00000004939 | GPM6A | red |
| ENSG00000005961 | PIP4K2A | red |
| ENSG00000008441 | ENKUR | red |
| ENSG00000011028 | TMEM56 | red |
| ENSG00000011105 | BANK1 | red |
| ENSG00000013306 | MCOLN2 | red |
| ENSG00000013573 | NRGN | red |
| ENSG00000017483 | FAM167A | red |
| ENSG00000019102 | ADK | red |
| ENSG00000022840 | CFAP161 | red |
| ENSG00000029534 | ANKRD9 | red |
| ENSG00000034239 | NRG1 | red |
| ENSG00000036448 | ERG | red |
| ENSG00000039068 | ALAS2 | red |
| ENSG00000047597 | DMTN | red |
| ENSG00000049759 | SLC5A11 | red |
| ENSG00000053108 | ADIPOR1 | red |
| ENSG00000057757 | TREML1 | red |
| ENSG00000060138 | PDZK1IP1 | red |
| ENSG00000062582 | TAL1 | red |
| ENSG00000063854 | TRIM58 | red |
| ENSG00000065491 | PM20D1 | red |
| ENSG00000065534 | FSTL1 | red |
| ENSG00000065978 | SPTA1 | red |
| ENSG00000066926 | PF4 | red |
| ENSG00000070182 | LRRC2 | red |
| ENSG00000073282 | LIPH | red |
| ENSG00000073792 | ERMAP | red |
| ENSG00000076043 | CAMP | red |
| ENSG00000076770 | CMBL | red |
| ENSG00000076864 | EBF1 | red |
| ENSG00000079308 | DEFA4 | red |
| ENSG00000082146 | GPR146 | red |
| ENSG00000082781 | CA3 | red |
| ENSG00000085733 | ARMC3 | red |
| ENSG00000085831 | TSHR | red |
| ENSG00000086232 | FUNDC2 | red |
| ENSG00000086506 | METTL17 | red |
| ENSG00000087085 | C1QL3 | red |
| ENSG00000088826 | AVPR1A | red |
| ENSG00000088992 | POLL | red |
| ENSG00000090013 | XRRA1 | red |
| ENSG00000095303 | YPEL4 | red |
| ENSG00000096093 | PLIN1 | red |
| ENSG00000099804 | EPB42 | red |
| ENSG00000100012 | JSRP1 | red |
| ENSG00000100104 | UBXN6 | red |
| ENSG00000100225 | KRT1 | red |

| | | |
|---|---|---|
| ENSG00000100325 | RAB3IL1 | red |
| ENSG00000100380 | MAJIN | red |
| ENSG00000100558 | DDIT4 | red |
| ENSG00000101470 | TSPAN5 | red |
| ENSG00000102145 | C8orf46 | red |
| ENSG00000102904 | PARM1 | red |
| ENSG00000103148 | CPT1C | red |
| ENSG00000103184 | SH3TC2 | red |
| ENSG00000103257 | GP9 | red |
| ENSG00000103342 | AHSP | red |
| ENSG00000103534 | GYPA | red |
| ENSG00000104892 | FAXDC2 | red |
| ENSG00000104903 | UBB | red |
| ENSG00000105492 | MZB1 | red |
| ENSG00000105507 | HSD17B13 | red |
| ENSG00000105610 | HTRA3 | red |
| ENSG00000105701 | ZNF415 | red |
| ENSG00000106366 | BCL2L1 | red |
| ENSG00000107262 | BPGM | red |
| ENSG00000108309 | PRSS30P | red |
| ENSG00000108576 | XKR3 | red |
| ENSG00000109686 | ABLIM3 | red |
| ENSG00000110693 | OLR1 | red |
| ENSG00000110799 | 3-Mar | red |
| ENSG00000111058 | PPM1E | red |
| ENSG00000111666 | AP002986.1 | red |
| ENSG00000111834 | PLEKHD1 | red |
| ENSG00000111846 | RNF152 | red |
| ENSG00000112077 | MYO1D | red |
| ENSG00000112139 | ZNF620 | red |
| ENSG00000112212 | GLDC | red |
| ENSG00000112232 | ZNF713 | red |
| ENSG00000112511 | ABCA13 | red |
| ENSG00000113140 | ITLN1 | red |
| ENSG00000113924 | FAHD1 | red |
| ENSG00000115758 | MTURN | red |
| ENSG00000116717 | BHLHA15 | red |
| ENSG00000117394 | RNF182 | red |
| ENSG00000118473 | PCGF5 | red |
| ENSG00000119326 | HIST3H2A | red |
| ENSG00000119636 | SIAH2 | red |
| ENSG00000119720 | SLC2A4 | red |
| ENSG00000119782 | GLRX5 | red |
| ENSG00000119950 | ADI1 | red |
| ENSG00000120885 | RGS6 | red |
| ENSG00000121104 | C1orf116 | red |
| ENSG00000122203 | RGS7 | red |
| ENSG00000124098 | PYCR1 | red |

| | | |
|---|---|---|
| ENSG00000124635 | ARHGEF37 | red |
| ENSG00000125037 | FAM46C | red |
| ENSG00000125657 | SFXN4 | red |
| ENSG00000128266 | CLDN5 | red |
| ENSG00000129244 | FOXO4 | red |
| ENSG00000130147 | CDCA2 | red |
| ENSG00000130227 | OSBP2 | red |
| ENSG00000130300 | TMEM121 | red |
| ENSG00000130558 | PBX1 | red |
| ENSG00000130656 | KRT5 | red |
| ENSG00000130821 | EDARADD | red |
| ENSG00000132793 | UBE2H | red |
| ENSG00000132819 | BCAM | red |
| ENSG00000133069 | PEAR1 | red |
| ENSG00000133488 | SHISA7 | red |
| ENSG00000133606 | HS6ST1P1 | red |
| ENSG00000133742 | C17orf99 | red |
| ENSG00000134779 | HEPACAM2 | red |
| ENSG00000136732 | NUTM2B | red |
| ENSG00000136819 | JAKMIP3 | red |
| ENSG00000136840 | HBA2 | red |
| ENSG00000136842 | NPIPP1 | red |
| ENSG00000136908 | SUMO2 | red |
| ENSG00000136929 | RHCE | red |
| ENSG00000137193 | TMSB4XP2 | red |
| ENSG00000137198 | RPSAP47 | red |
| ENSG00000137834 | LRRC66 | red |
| ENSG00000137936 | PAX8-AS1 | red |
| ENSG00000140022 | CTSE | red |
| ENSG00000140479 | SLC6A9 | red |
| ENSG00000141034 | HBG2 | red |
| ENSG00000141084 | ARHGEF12 | red |
| ENSG00000141448 | KANK2 | red |
| ENSG00000142453 | VEPH1 | red |
| ENSG00000142949 | CR1L | red |
| ENSG00000143321 | KEL | red |
| ENSG00000143416 | MYL4 | red |
| ENSG00000143479 | FLVCR1-AS1 | red |
| ENSG00000143595 | HLA-DRB5 | red |
| ENSG00000143774 | DCAF12 | red |
| ENSG00000143842 | TGM2 | red |
| ENSG00000144554 | AL355802.1 | red |
| ENSG00000144648 | ADD3-AS1 | red |
| ENSG00000144677 | IFIT1B | red |
| ENSG00000145335 | GGTA1P | red |
| ENSG00000145423 | C10orf128 | red |
| ENSG00000147454 | TMEM57 | red |
| ENSG00000148218 | HLA-DMA | red |

| | | |
|---|---|---|
| ENSG00000148426 | VARS | red |
| ENSG00000148498 | TTC25 | red |
| ENSG00000148908 | NAT8B | red |
| ENSG00000149328 | MFSD2B | red |
| ENSG00000149564 | DEFA1 | red |
| ENSG00000150625 | HBA1 | red |
| ENSG00000150867 | HBM | red |
| ENSG00000151023 | ATP10A | red |
| ENSG00000152078 | HLA-H | red |
| ENSG00000153898 | IGKC | red |
| ENSG00000154146 | IGKJ5 | red |
| ENSG00000154319 | IGKJ4 | red |
| ENSG00000156206 | IGKV4-1 | red |
| ENSG00000156381 | IGKV3D-20 | red |
| ENSG00000157168 | IGLV4-69 | red |
| ENSG00000158578 | IGLV4-60 | red |
| ENSG00000158856 | IGLV6-57 | red |
| ENSG00000158865 | IGLV1-51 | red |
| ENSG00000159346 | IGLV1-47 | red |
| ENSG00000161911 | IGLV7-46 | red |
| ENSG00000162366 | IGLV1-44 | red |
| ENSG00000162367 | IGLV7-43 | red |
| ENSG00000162722 | IGLV1-40 | red |
| ENSG00000163430 | IGLV2-23 | red |
| ENSG00000163554 | IGLV3-21 | red |
| ENSG00000163737 | IGLV3-19 | red |
| ENSG00000163827 | IGLV2-18 | red |
| ENSG00000163898 | IGLV3-16 | red |
| ENSG00000164010 | IGLV2-11 | red |
| ENSG00000164237 | IGLV3-10 | red |
| ENSG00000164849 | IGLV3-9 | red |
| ENSG00000164879 | IGLV4-3 | red |
| ENSG00000165309 | IGLV3-1 | red |
| ENSG00000165775 | IGLC2 | red |
| ENSG00000165792 | IGLC3 | red |
| ENSG00000165985 | IGHA2 | red |
| ENSG00000166148 | IGHG4 | red |
| ENSG00000166169 | IGHG2 | red |
| ENSG00000166793 | IGHA1 | red |
| ENSG00000166947 | IGHG1 | red |
| ENSG00000167671 | IGHG3 | red |
| ENSG00000167768 | IGHV6-1 | red |
| ENSG00000167994 | IGHV1-2 | red |
| ENSG00000168209 | IGHV1-3 | red |
| ENSG00000168785 | IGHV2-5 | red |
| ENSG00000169085 | IGHV3-7 | red |
| ENSG00000169169 | IGHV3-15 | red |
| ENSG00000169247 | IGHV1-18 | red |

| | | |
|---|---|---|
| ENSG00000169704 | IGHV3-21 | red |
| ENSG00000169877 | IGHV3-23 | red |
| ENSG00000170180 | IGHV3-33 | red |
| ENSG00000170271 | ACKR1 | red |
| ENSG00000170315 | AC105052.2 | red |
| ENSG00000170954 | AL356317.1 | red |
| ENSG00000171552 | YBX1P10 | red |
| ENSG00000172331 | HBG1 | red |
| ENSG00000172460 | RPS4XP1 | red |
| ENSG00000172967 | NEURL1B | red |
| ENSG00000173210 | BTNL10 | red |
| ENSG00000173926 | MMP23A | red |
| ENSG00000175175 | GLRX3P2 | red |
| ENSG00000175773 | CCNL2 | red |
| ENSG00000175985 | UBA52 | red |
| ENSG00000176641 | RNY3P8 | red |
| ENSG00000177842 | KIF28P | red |
| ENSG00000179914 | HBD | red |
| ENSG00000180185 | HRAT92 | red |
| ENSG00000180354 | IGKV3D-15 | red |
| ENSG00000180535 | CCNT2-AS1 | red |
| ENSG00000180537 | LINC00570 | red |
| ENSG00000180628 | RTCA-AS1 | red |
| ENSG00000181218 | IGHV3-74 | red |
| ENSG00000181788 | IMPDH1P8 | red |
| ENSG00000181856 | YBX1P1 | red |
| ENSG00000182512 | ST13P6 | red |
| ENSG00000182732 | Z82206.1 | red |
| ENSG00000182795 | UBXN10-AS1 | red |
| ENSG00000182901 | LINC01535 | red |
| ENSG00000183111 | AC005154.2 | red |
| ENSG00000183508 | WDR11-AS1 | red |
| ENSG00000184113 | AL359644.1 | red |
| ENSG00000184481 | FAM212B-AS1 | red |
| ENSG00000184792 | AC108463.1 | red |
| ENSG00000185630 | AL031283.1 | red |
| ENSG00000186197 | COL6A4P2 | red |
| ENSG00000186591 | BX284668.2 | red |
| ENSG00000187244 | SLC25A38P1 | red |
| ENSG00000187800 | TPI1P2 | red |
| ENSG00000187902 | AL139220.2 | red |
| ENSG00000187997 | AC099568.1 | red |
| ENSG00000188175 | DIRC3 | red |
| ENSG00000188385 | AC010733.1 | red |
| ENSG00000188536 | YWHAEP1 | red |
| ENSG00000188672 | AC231533.2 | red |
| ENSG00000188765 | AL157895.1 | red |
| ENSG00000188856 | JAZF1-AS1 | red |

| | | |
|---|---|---|
| ENSG00000188993 | RHOXF1P1 | red |
| ENSG00000189223 | LINC01376 | red |
| ENSG00000196188 | AC092809.3 | red |
| ENSG00000196517 | LINC01358 | red |
| ENSG00000196565 | TRIM51EP | red |
| ENSG00000196914 | TXNDC5 | red |
| ENSG00000197256 | IGKV2D-30 | red |
| ENSG00000197415 | DEFA3 | red |
| ENSG00000197721 | IGKV3-20 | red |
| ENSG00000197993 | IGKV1D-33 | red |
| ENSG00000198336 | DEFA1B | red |
| ENSG00000198468 | AC104763.1 | red |
| ENSG00000198502 | IGKV2D-24 | red |
| ENSG00000198876 | IGKV2D-28 | red |
| ENSG00000198959 | ZNF709 | red |
| ENSG00000203362 | IGKV1-5 | red |
| ENSG00000204010 | IGKV3-15 | red |
| ENSG00000204136 | AC018797.2 | red |
| ENSG00000204161 | AC091849.1 | red |
| ENSG00000204178 | AC008438.1 | red |
| ENSG00000204257 | IGKV1D-39 | red |
| ENSG00000204394 | IGHGP | red |
| ENSG00000204815 | LINC01933 | red |
| ENSG00000204872 | IGLL5 | red |
| ENSG00000205639 | RAB44 | red |
| ENSG00000206172 | IFNG-AS1 | red |
| ENSG00000206177 | CCND2-AS1 | red |
| ENSG00000206190 | AC008083.2 | red |
| ENSG00000206341 | AL133467.1 | red |
| ENSG00000211665 | AC007922.1 | red |
| ENSG00000213088 | AC027097.1 | red |
| ENSG00000213754 | AC006557.1 | red |
| ENSG00000213866 | PARD6G-AS1 | red |
| ENSG00000213934 | AC092295.2 | red |
| ENSG00000214203 | AL049840.3 | red |
| ENSG00000214357 | AC136475.7 | red |
| ENSG00000215811 | YTHDF2P1 | red |
| ENSG00000216657 | IGHV3-30 | red |
| ENSG00000221978 | AC087501.5 | red |
| ENSG00000221983 | AC010245.2 | red |
| ENSG00000223298 | AC019131.2 | red |
| ENSG00000223519 | AC073389.3 | red |
| ENSG00000223609 | AC092653.1 | red |
| ENSG00000223855 | AP000894.4 | red |
| ENSG00000224043 | AL157392.4 | red |
| ENSG00000224177 | AL354809.1 | red |
| ENSG00000224616 | AL117379.1 | red |
| ENSG00000224861 | AC121761.2 | red |

| | | |
|---|---|---|
| ENSG00000225259 | IGHV2-70 | red |
| ENSG00000225528 | AC009318.4 | red |
| ENSG00000226686 | PIK3R6 | red |
| ENSG00000226874 | TYW1B | red |
| ENSG00000227165 | IGLV2-8 | red |
| ENSG00000227355 | IGKV1D-12 | red |
| ENSG00000227811 | AC092718.7 | red |
| ENSG00000227992 | AC090772.4 | red |
| ENSG00000228252 | ADORA3 | red |
| ENSG00000229785 | RHOXF1P2 | red |
| ENSG00000230615 | RHOXF1P3 | red |
| ENSG00000232713 | AC004381.2 | red |
| ENSG00000233585 | LINC02009 | red |
| ENSG00000233868 | DPM1 | red |
| ENSG00000234449 | SCYL3 | red |
| ENSG00000234572 | C1orf112 | red |
| ENSG00000235105 | CFH | red |
| ENSG00000235272 | GCLC | red |
| ENSG00000236279 | NFYA | red |
| ENSG00000236493 | LAS1L | red |
| ENSG00000236723 | ENPP4 | red |
| ENSG00000238039 | ANKIB1 | red |
| ENSG00000238164 | KRIT1 | red |
| ENSG00000238243 | BAD | red |
| ENSG00000239219 | HS3ST1 | red |
| ENSG00000240583 | MAD1L1 | red |
| ENSG00000240875 | M6PR | red |
| ENSG00000243064 | KLHL13 | red |
| ENSG00000243317 | ICA1 | red |
| ENSG00000244649 | ALS2 | red |
| ENSG00000244734 | NDUFAF7 | red |
| ENSG00000246528 | MTMR7 | red |
| ENSG00000248445 | ARF5 | red |
| ENSG00000250361 | PLXND1 | red |
| ENSG00000250966 | CAMKK1 | red |
| ENSG00000253305 | RECQL | red |
| ENSG00000253549 | VPS50 | red |
| ENSG00000253986 | ARHGAP33 | red |
| ENSG00000254415 | PDK4 | red |
| ENSG00000254481 | ZMYND10 | red |
| ENSG00000254614 | SLC25A13 | red |
| ENSG00000254717 | CDC27 | red |
| ENSG00000256020 | DVL2 | red |
| ENSG00000256312 | SKAP2 | red |
| ENSG00000257052 | MCUB | red |
| ENSG00000257851 | POLR2J | red |
| ENSG00000258469 | DHX33 | red |
| ENSG00000258769 | LIG3 | red |

| | | |
|---|---|---|
| ENSG00000259207 | RPAP3 | red |
| ENSG00000260188 | ACSM3 | red |
| ENSG00000260231 | SPPL2B | red |
| ENSG00000260461 | PRKAR2B | red |
| ENSG00000260592 | MSL3 | red |
| ENSG00000261614 | GCFC2 | red |
| ENSG00000264968 | CROT | red |
| ENSG00000265612 | RHBDD2 | red |
| ENSG00000266401 | ZNF195 | red |
| ENSG00000268119 | MYCBP2 | red |
| ENSG00000269887 | FBXL3 | red |
| ENSG00000270558 | ITGAL | red |
| ENSG00000271736 | PDK2 | red |
| ENSG00000271918 | ITGA3 | red |
| ENSG00000272716 | ZFX | red |
| ENSG00000274628 | LAMP2 | red |
| ENSG00000274776 | GDE1 | red |
| ENSG00000275111 | CRLF1 | red |
| ENSG00000275294 | OSBPL7 | red |
| ENSG00000277782 | MAP3K14 | red |
| ENSG00000278030 | ZNF263 | red |
| ENSG00000278828 | SPATA20 | red |
| ENSG00000279529 | TNFRSF12A | red |
| ENSG00000279565 | MAP3K9 | red |
| ENSG00000279841 | RALA | red |
| ENSG00000279928 | KDM7A | red |
| ENSG00000279943 | AGK | red |
| ENSG00000280212 | ALDH3B1 | red |
| ENSG00000281376 | TTC22 | red |
| ENSG00000282024 | PHTF2 | red |
| ENSG00000283189 | GGCT | red |
| ENSG00000284138 | DBF4 | red |
| ENSG00000000419 | IFRD1 | turquoise |
| ENSG00000000457 | COX10 | turquoise |
| ENSG00000000460 | VPS41 | turquoise |
| ENSG00000000971 | ARHGAP44 | turquoise |
| ENSG00000001084 | ARSD | turquoise |
| ENSG00000001167 | CDKL3 | turquoise |
| ENSG00000001497 | MARK4 | turquoise |
| ENSG00000001561 | CEACAM21 | turquoise |
| ENSG00000001629 | PAFAH1B1 | turquoise |
| ENSG00000001631 | KIAA0100 | turquoise |
| ENSG00000002330 | SCN4A | turquoise |
| ENSG00000002587 | RPUSD1 | turquoise |
| ENSG00000002822 | CACNA2D2 | turquoise |
| ENSG00000003056 | BAIAP3 | turquoise |
| ENSG00000003096 | PIGQ | turquoise |
| ENSG00000003147 | CRAMP1 | turquoise |

| | | |
|---|---|---|
| ENSG00000003393 | DNAJC11 | turquoise |
| ENSG00000003509 | MYLIP | turquoise |
| ENSG00000003987 | NOX1 | turquoise |
| ENSG00000004059 | JARID2 | turquoise |
| ENSG00000004399 | CYTH3 | turquoise |
| ENSG00000004660 | ADAM22 | turquoise |
| ENSG00000004700 | SYPL1 | turquoise |
| ENSG00000004766 | SPAG9 | turquoise |
| ENSG00000004777 | AASS | turquoise |
| ENSG00000004799 | SS18L2 | turquoise |
| ENSG00000004838 | CRY1 | turquoise |
| ENSG00000004864 | ST3GAL1 | turquoise |
| ENSG00000004897 | PKD1 | turquoise |
| ENSG00000004975 | RHOBTB2 | turquoise |
| ENSG00000005020 | HEATR5B | turquoise |
| ENSG00000005059 | SEC62 | turquoise |
| ENSG00000005075 | CSDE1 | turquoise |
| ENSG00000005100 | UBE3C | turquoise |
| ENSG00000005156 | REV3L | turquoise |
| ENSG00000005175 | FAM76A | turquoise |
| ENSG00000005187 | POMT2 | turquoise |
| ENSG00000005206 | VTA1 | turquoise |
| ENSG00000005249 | BAZ1B | turquoise |
| ENSG00000005302 | RANBP9 | turquoise |
| ENSG00000005436 | SPRTN | turquoise |
| ENSG00000005469 | ZNF207 | turquoise |
| ENSG00000005486 | STARD3NL | turquoise |
| ENSG00000005801 | CD9 | turquoise |
| ENSG00000005810 | IFFO1 | turquoise |
| ENSG00000005812 | PHF7 | turquoise |
| ENSG00000005844 | SEMA3G | turquoise |
| ENSG00000005882 | NISCH | turquoise |
| ENSG00000005884 | STAB1 | turquoise |
| ENSG00000005889 | FUZ | turquoise |
| ENSG00000005893 | SLC6A13 | turquoise |
| ENSG00000006007 | ZNF200 | turquoise |
| ENSG00000006016 | LRRC23 | turquoise |
| ENSG00000006025 | BTK | turquoise |
| ENSG00000006062 | SCMH1 | turquoise |
| ENSG00000006194 | HIVEP2 | turquoise |
| ENSG00000006282 | ELOA | turquoise |
| ENSG00000006327 | APBA3 | turquoise |
| ENSG00000006432 | MKS1 | turquoise |
| ENSG00000006451 | AKAP8L | turquoise |
| ENSG00000006459 | MBTD1 | turquoise |
| ENSG00000006530 | RNF216 | turquoise |
| ENSG00000006534 | TTC19 | turquoise |
| ENSG00000006555 | LARS2 | turquoise |

| | | |
|---|---|---|
| ENSG00000006576 | PIK3C2A | turquoise |
| ENSG00000006625 | ANLN | turquoise |
| ENSG00000006634 | WIZ | turquoise |
| ENSG00000006652 | RABGAP1 | turquoise |
| ENSG00000006695 | QPCTL | turquoise |
| ENSG00000006715 | PPP5C | turquoise |
| ENSG00000006740 | MAP4K3 | turquoise |
| ENSG00000006756 | ZBTB32 | turquoise |
| ENSG00000006837 | TYROBP | turquoise |
| ENSG00000007047 | BRCA1 | turquoise |
| ENSG00000007129 | ERCC1 | turquoise |
| ENSG00000007168 | SEMA3B | turquoise |
| ENSG00000007202 | MBTPS2 | turquoise |
| ENSG00000007314 | PRICKLE3 | turquoise |
| ENSG00000007376 | ELOVL5 | turquoise |
| ENSG00000007402 | KDM5D | turquoise |
| ENSG00000007516 | UBR7 | turquoise |
| ENSG00000007541 | MAP4K5 | turquoise |
| ENSG00000007545 | PSMC4 | turquoise |
| ENSG00000007923 | PGM3 | turquoise |
| ENSG00000007944 | RWDD2A | turquoise |
| ENSG00000007952 | CLK1 | turquoise |
| ENSG00000008083 | RNF14 | turquoise |
| ENSG00000008256 | DNASE1L1 | turquoise |
| ENSG00000008277 | MAMLD1 | turquoise |
| ENSG00000008282 | TACC3 | turquoise |
| ENSG00000008294 | UFL1 | turquoise |
| ENSG00000008311 | POLA2 | turquoise |
| ENSG00000008324 | ZC3H3 | turquoise |
| ENSG00000008405 | CAPN1 | turquoise |
| ENSG00000008513 | MDH1 | turquoise |
| ENSG00000008710 | SLC30A9 | turquoise |
| ENSG00000008853 | YAF2 | turquoise |
| ENSG00000008869 | ZMYND11 | turquoise |
| ENSG00000008952 | GLT8D1 | turquoise |
| ENSG00000009307 | ATP2C1 | turquoise |
| ENSG00000009335 | RALBP1 | turquoise |
| ENSG00000009413 | AGPS | turquoise |
| ENSG00000009780 | CXorf56 | turquoise |
| ENSG00000009830 | TTC27 | turquoise |
| ENSG00000009844 | ZNF582 | turquoise |
| ENSG00000009954 | PHLDB1 | turquoise |
| ENSG00000010017 | HGF | turquoise |
| ENSG00000010072 | ZRANB1 | turquoise |
| ENSG00000010244 | NCDN | turquoise |
| ENSG00000010270 | ADGRA2 | turquoise |
| ENSG00000010278 | ZFP64 | turquoise |
| ENSG00000010295 | MNAT1 | turquoise |

| | | |
|---|---|---|
| ENSG00000010318 | MRE11 | turquoise |
| ENSG00000010319 | SPAST | turquoise |
| ENSG00000010322 | AQR | turquoise |
| ENSG00000010327 | CPS1 | turquoise |
| ENSG00000010361 | FHL1 | turquoise |
| ENSG00000010379 | ZDHHC6 | turquoise |
| ENSG00000010539 | RNH1 | turquoise |
| ENSG00000010626 | RB1CC1 | turquoise |
| ENSG00000010671 | BIRC3 | turquoise |
| ENSG00000010803 | AKAP11 | turquoise |
| ENSG00000010818 | DERA | turquoise |
| ENSG00000011007 | STRAP | turquoise |
| ENSG00000011132 | DEF6 | turquoise |
| ENSG00000011143 | GCLM | turquoise |
| ENSG00000011243 | UBR2 | turquoise |
| ENSG00000011258 | EHD2 | turquoise |
| ENSG00000011275 | CCDC28A | turquoise |
| ENSG00000011295 | RRAGD | turquoise |
| ENSG00000011376 | HSF2 | turquoise |
| ENSG00000011405 | PHF20 | turquoise |
| ENSG00000011426 | NR1H3 | turquoise |
| ENSG00000011451 | SEC63 | turquoise |
| ENSG00000011454 | VIM | turquoise |
| ENSG00000011478 | FAS | turquoise |
| ENSG00000011485 | RNASET2 | turquoise |
| ENSG00000011566 | CD44 | turquoise |
| ENSG00000011590 | AGPAT4 | turquoise |
| ENSG00000011600 | PRKCH | turquoise |
| ENSG00000012048 | IFNGR1 | turquoise |
| ENSG00000012061 | VRK2 | turquoise |
| ENSG00000012171 | VEZT | turquoise |
| ENSG00000012174 | POU2F2 | turquoise |
| ENSG00000012211 | BRD9 | turquoise |
| ENSG00000012660 | TBPL1 | turquoise |
| ENSG00000012817 | BCLAF1 | turquoise |
| ENSG00000012963 | RABEP1 | turquoise |
| ENSG00000012983 | NUP160 | turquoise |
| ENSG00000013275 | IKZF2 | turquoise |
| ENSG00000013375 | FAM13B | turquoise |
| ENSG00000013392 | CENPQ | turquoise |
| ENSG00000013441 | RANBP3 | turquoise |
| ENSG00000013561 | ARID4A | turquoise |
| ENSG00000013563 | PNPLA6 | turquoise |
| ENSG00000013619 | IFT88 | turquoise |
| ENSG00000013810 | ZCCHC8 | turquoise |
| ENSG00000014123 | LRRC7 | turquoise |
| ENSG00000014138 | FUT8 | turquoise |
| ENSG00000014164 | UBA6 | turquoise |

| | | |
|---|---|---|
| ENSG00000014216 | ATP6V0A1 | turquoise |
| ENSG00000014641 | SLC4A7 | turquoise |
| ENSG00000014824 | MAP2K3 | turquoise |
| ENSG00000015153 | RNF19A | turquoise |
| ENSG00000015171 | GABARAPL2 | turquoise |
| ENSG00000016864 | FAM136A | turquoise |
| ENSG00000017260 | VCL | turquoise |
| ENSG00000017797 | DEPDC1B | turquoise |
| ENSG00000018510 | NSMAF | turquoise |
| ENSG00000018610 | ADSS | turquoise |
| ENSG00000018699 | STAP1 | turquoise |
| ENSG00000018869 | RFC1 | turquoise |
| ENSG00000019144 | TBC1D23 | turquoise |
| ENSG00000019991 | CUL3 | turquoise |
| ENSG00000019995 | CYP46A1 | turquoise |
| ENSG00000020129 | ZZZ3 | turquoise |
| ENSG00000020181 | TUBG2 | turquoise |
| ENSG00000020256 | RPL26L1 | turquoise |
| ENSG00000020426 | NSUN2 | turquoise |
| ENSG00000020922 | AGA | turquoise |
| ENSG00000021574 | PI4K2B | turquoise |
| ENSG00000021776 | BOD1L1 | turquoise |
| ENSG00000021826 | MAT2B | turquoise |
| ENSG00000022267 | EDC4 | turquoise |
| ENSG00000023041 | TRIO | turquoise |
| ENSG00000023191 | VCAN | turquoise |
| ENSG00000023287 | CLEC16A | turquoise |
| ENSG00000023445 | SKIV2L2 | turquoise |
| ENSG00000023516 | ZFYVE16 | turquoise |
| ENSG00000023697 | RIPOR1 | turquoise |
| ENSG00000023734 | RAI14 | turquoise |
| ENSG00000023892 | SOX30 | turquoise |
| ENSG00000023909 | PNKP | turquoise |
| ENSG00000024048 | PHLPP2 | turquoise |
| ENSG00000024422 | SPDL1 | turquoise |
| ENSG00000024862 | STAU2 | turquoise |
| ENSG00000025039 | PQLC2 | turquoise |
| ENSG00000025156 | PHF23 | turquoise |
| ENSG00000025293 | RAB27B | turquoise |
| ENSG00000025434 | PSMA4 | turquoise |
| ENSG00000025796 | LSG1 | turquoise |
| ENSG00000026025 | AIFM2 | turquoise |
| ENSG00000026103 | SPATA7 | turquoise |
| ENSG00000026297 | CAPG | turquoise |
| ENSG00000026508 | DCUN1D1 | turquoise |
| ENSG00000026652 | ADRB1 | turquoise |
| ENSG00000027075 | CUL7 | turquoise |
| ENSG00000027697 | HSPA5 | turquoise |

| | | |
|---|---|---|
| ENSG00000028116 | GEMIN8 | turquoise |
| ENSG00000028203 | OFD1 | turquoise |
| ENSG00000028277 | WDR37 | turquoise |
| ENSG00000028310 | YTHDC2 | turquoise |
| ENSG00000028839 | CTPS2 | turquoise |
| ENSG00000029363 | ATP6V1H | turquoise |
| ENSG00000029725 | POLR2B | turquoise |
| ENSG00000030066 | FAM214A | turquoise |
| ENSG00000030419 | ARAP2 | turquoise |
| ENSG00000031003 | TPR | turquoise |
| ENSG00000031691 | C12orf4 | turquoise |
| ENSG00000031823 | SCML1 | turquoise |
| ENSG00000032219 | ARHGAP6 | turquoise |
| ENSG00000032444 | FAM184B | turquoise |
| ENSG00000032742 | GOPC | turquoise |
| ENSG00000033030 | USP28 | turquoise |
| ENSG00000033122 | HDAC9 | turquoise |
| ENSG00000033170 | NOP16 | turquoise |
| ENSG00000033178 | RRM2B | turquoise |
| ENSG00000033627 | ZNF800 | turquoise |
| ENSG00000033867 | SNX29 | turquoise |
| ENSG00000034152 | RSF1 | turquoise |
| ENSG00000034677 | VPS13D | turquoise |
| ENSG00000034713 | FAM120A | turquoise |
| ENSG00000035141 | R3HDM1 | turquoise |
| ENSG00000035403 | COL9A2 | turquoise |
| ENSG00000035499 | ERCC8 | turquoise |
| ENSG00000035681 | PER3 | turquoise |
| ENSG00000035687 | LTBP1 | turquoise |
| ENSG00000035720 | RCN1 | turquoise |
| ENSG00000035928 | RFC2 | turquoise |
| ENSG00000036054 | ARID1B | turquoise |
| ENSG00000036257 | FOXP3 | turquoise |
| ENSG00000036530 | HEXB | turquoise |
| ENSG00000036549 | NEXMIF | turquoise |
| ENSG00000037042 | JKAMP | turquoise |
| ENSG00000037241 | ARHGEF5 | turquoise |
| ENSG00000037474 | NFE2L3 | turquoise |
| ENSG00000038002 | MCUR1 | turquoise |
| ENSG00000038210 | LIMA1 | turquoise |
| ENSG00000038219 | LAMC3 | turquoise |
| ENSG00000038274 | MAPK9 | turquoise |
| ENSG00000038358 | COL23A1 | turquoise |
| ENSG00000038382 | HOMER3 | turquoise |
| ENSG00000038427 | POLQ | turquoise |
| ENSG00000038532 | PIK3CB | turquoise |
| ENSG00000039123 | CYBA | turquoise |
| ENSG00000039319 | MPHOSPH9 | turquoise |

| | | |
|---|---|---|
| ENSG00000039523 | PLEKHA5 | turquoise |
| ENSG00000039560 | SIKE1 | turquoise |
| ENSG00000039600 | FNIP2 | turquoise |
| ENSG00000039650 | MSMO1 | turquoise |
| ENSG00000040199 | TTC17 | turquoise |
| ENSG00000040275 | AP5M1 | turquoise |
| ENSG00000040341 | ANAPC4 | turquoise |
| ENSG00000040487 | ENTPD2 | turquoise |
| ENSG00000040633 | LY75 | turquoise |
| ENSG00000041353 | ARID4B | turquoise |
| ENSG00000041357 | OPN3 | turquoise |
| ENSG00000041802 | TBC1D22A | turquoise |
| ENSG00000042286 | SYNE2 | turquoise |
| ENSG00000042317 | GALC | turquoise |
| ENSG00000042493 | NOP58 | turquoise |
| ENSG00000043093 | FAM114A2 | turquoise |
| ENSG00000043591 | TAB2 | turquoise |
| ENSG00000044090 | GINM1 | turquoise |
| ENSG00000044574 | KMT2C | turquoise |
| ENSG00000046647 | PUM2 | turquoise |
| ENSG00000046651 | ITIH4 | turquoise |
| ENSG00000047056 | ITIH1 | turquoise |
| ENSG00000047188 | HPF1 | turquoise |
| ENSG00000047230 | ZFR | turquoise |
| ENSG00000047249 | ZNF280C | turquoise |
| ENSG00000047315 | TRAF1 | turquoise |
| ENSG00000047346 | RC3H2 | turquoise |
| ENSG00000047365 | DCBLD2 | turquoise |
| ENSG00000047410 | SOAT1 | turquoise |
| ENSG00000047621 | PKP2 | turquoise |
| ENSG00000047634 | GDI2 | turquoise |
| ENSG00000047648 | PRDM1 | turquoise |
| ENSG00000047662 | ATG5 | turquoise |
| ENSG00000047932 | USP13 | turquoise |
| ENSG00000048028 | ATP11B | turquoise |
| ENSG00000048052 | CDK14 | turquoise |
| ENSG00000048162 | PPP1R12A | turquoise |
| ENSG00000048392 | RASGRF1 | turquoise |
| ENSG00000048405 | CROCC | turquoise |
| ENSG00000048471 | POLR3E | turquoise |
| ENSG00000048649 | ATP2B4 | turquoise |
| ENSG00000048707 | RIOK2 | turquoise |
| ENSG00000048828 | NDC1 | turquoise |
| ENSG00000048991 | FLYWCH1 | turquoise |
| ENSG00000049089 | TARBP1 | turquoise |
| ENSG00000049167 | MXD1 | turquoise |
| ENSG00000049246 | CDK17 | turquoise |
| ENSG00000049323 | DNAJC25 | turquoise |

| | | |
|---|---|---|
| ENSG00000049449 | CTDP1 | turquoise |
| ENSG00000049541 | WNK1 | turquoise |
| ENSG00000049618 | CCAR1 | turquoise |
| ENSG00000049768 | PIGV | turquoise |
| ENSG00000049860 | QSER1 | turquoise |
| ENSG00000050030 | MPC1 | turquoise |
| ENSG00000050130 | BCAT1 | turquoise |
| ENSG00000050327 | SPAG4 | turquoise |
| ENSG00000050344 | NCKAP1 | turquoise |
| ENSG00000050393 | GUCY1B3 | turquoise |
| ENSG00000050405 | SFSWAP | turquoise |
| ENSG00000050555 | MON2 | turquoise |
| ENSG00000050748 | GPBP1 | turquoise |
| ENSG00000050767 | ZNF112 | turquoise |
| ENSG00000051128 | LTK | turquoise |
| ENSG00000051341 | ELMO2 | turquoise |
| ENSG00000051382 | WAPL | turquoise |
| ENSG00000051523 | VMP1 | turquoise |
| ENSG00000051825 | APPBP2 | turquoise |
| ENSG00000052126 | POLD1 | turquoise |
| ENSG00000052723 | SLC6A16 | turquoise |
| ENSG00000052795 | BICRA | turquoise |
| ENSG00000052802 | SPHK2 | turquoise |
| ENSG00000052841 | U2AF2 | turquoise |
| ENSG00000053770 | EPN1 | turquoise |
| ENSG00000053900 | MED29 | turquoise |
| ENSG00000054179 | MTMR1 | turquoise |
| ENSG00000054219 | GPC1 | turquoise |
| ENSG00000054267 | ADCK1 | turquoise |
| ENSG00000054277 | CASP8 | turquoise |
| ENSG00000054611 | INTS13 | turquoise |
| ENSG00000054654 | TM7SF3 | turquoise |
| ENSG00000054983 | TSPAN32 | turquoise |
| ENSG00000055044 | NGFR | turquoise |
| ENSG00000055147 | TAF2 | turquoise |
| ENSG00000055208 | HIPK2 | turquoise |
| ENSG00000055211 | TNPO3 | turquoise |
| ENSG00000055609 | BORCS8-MEF2B | turquoise |
| ENSG00000055917 | SLC12A2 | turquoise |
| ENSG00000055955 | SNX24 | turquoise |
| ENSG00000055957 | CNN2 | turquoise |
| ENSG00000056050 | ABCA7 | turquoise |
| ENSG00000056097 | DDX20 | turquoise |
| ENSG00000056277 | BTBD1 | turquoise |
| ENSG00000056558 | PMS1 | turquoise |
| ENSG00000056586 | HMG20B | turquoise |
| ENSG00000057019 | CALCRL | turquoise |
| ENSG00000057252 | TAF11 | turquoise |

| | | |
|---|---|---|
| ENSG00000057294 | ZNF76 | turquoise |
| ENSG00000057608 | SLC9A3R2 | turquoise |
| ENSG00000057657 | GNAI3 | turquoise |
| ENSG00000057663 | IPO5 | turquoise |
| ENSG00000058056 | OAT | turquoise |
| ENSG00000058063 | WDR3 | turquoise |
| ENSG00000058091 | AC009302.1 | turquoise |
| ENSG00000058272 | FAM239A | turquoise |
| ENSG00000058335 | LINC01800 | turquoise |
| ENSG00000058453 | AL356968.2 | turquoise |
| ENSG00000058600 | FAM103A2P | turquoise |
| ENSG00000058668 | CLEC2L | turquoise |
| ENSG00000058729 | EIF2S2P3 | turquoise |
| ENSG00000058804 | AL606760.2 | turquoise |
| ENSG00000059122 | AC244197.2 | turquoise |
| ENSG00000059588 | TNFRSF14-AS1 | turquoise |
| ENSG00000059728 | OR2W3 | turquoise |
| ENSG00000059758 | AC008040.1 | turquoise |
| ENSG00000059769 | AQP1 | turquoise |
| ENSG00000060069 | LINC00886 | turquoise |
| ENSG00000060237 | ABCC13 | turquoise |
| ENSG00000060339 | C7orf73 | turquoise |
| ENSG00000060642 | LINC02086 | turquoise |
| ENSG00000060749 | HBB | turquoise |
| ENSG00000060762 | AC079089.1 | turquoise |
| ENSG00000060982 | SEMA6A-AS1 | turquoise |
| ENSG00000061656 | GYPB | turquoise |
| ENSG00000061676 | AC023886.2 | turquoise |
| ENSG00000061918 | PCDHGB6 | turquoise |
| ENSG00000061936 | CA3-AS1 | turquoise |
| ENSG00000061987 | AC104561.3 | turquoise |
| ENSG00000062194 | SIGLEC14 | turquoise |
| ENSG00000062370 | AP000911.2 | turquoise |
| ENSG00000062524 | AP003068.2 | turquoise |
| ENSG00000062598 | GLYATL1P2 | turquoise |
| ENSG00000062650 | AC006205.2 | turquoise |
| ENSG00000062716 | AC138466.1 | turquoise |
| ENSG00000062725 | AP003721.4 | turquoise |
| ENSG00000062822 | HNRNPA3P10 | turquoise |
| ENSG00000063127 | CHMP4BP1 | turquoise |
| ENSG00000063169 | AC005479.1 | turquoise |
| ENSG00000063176 | ITGB3 | turquoise |
| ENSG00000063244 | AC002464.1 | turquoise |
| ENSG00000063245 | JHDM1D-AS1 | turquoise |
| ENSG00000063322 | AL133355.1 | turquoise |
| ENSG00000063601 | AC130456.3 | turquoise |
| ENSG00000063660 | AC106730.1 | turquoise |
| ENSG00000063761 | AC090844.2 | turquoise |

| | | |
|---|---|---|
| ENSG00000064012 | MIR4539 | turquoise |
| ENSG00000064102 | AP002478.1 | turquoise |
| ENSG00000064115 | AC010615.2 | turquoise |
| ENSG00000064201 | AL391001.1 | turquoise |
| ENSG00000064300 | AC025449.1 | turquoise |
| ENSG00000064313 | AL138900.3 | turquoise |
| ENSG00000064393 | AC034236.2 | turquoise |
| ENSG00000064419 | AL121658.1 | turquoise |
| ENSG00000064489 | AL669942.1 | turquoise |
| ENSG00000064651 | AC090241.3 | turquoise |
| ENSG00000064652 | ZNF2 | turquoise |
| ENSG00000064666 | LINC02340 | turquoise |
| ENSG00000064687 | AC068870.2 | turquoise |
| ENSG00000064703 | TRBV7-9 | turquoise |
| ENSG00000064726 | HIST1H3H | turquoise |
| ENSG00000064933 | AC008764.8 | turquoise |
| ENSG00000064961 | AL121835.2 | turquoise |
| ENSG00000064989 | AC092135.3 | turquoise |
| ENSG00000064995 | FO538757.2 | turquoise |
| ENSG00000065029 | FLJ38576 | turquoise |
| ENSG00000065054 | AC012254.5 | turquoise |
| ENSG00000065135 | ABALON | turquoise |
| ENSG00000065150 | AL009031.1 | turquoise |
| ENSG00000065154 | AC104452.1 | turquoise |
| ENSG00000065183 | AC099795.2 | turquoise |
| ENSG00000065243 | PKN2 | turquoise |
| ENSG00000065308 | TRAM2 | turquoise |
| ENSG00000065357 | DGKA | turquoise |
| ENSG00000065548 | ZC3H15 | turquoise |
| ENSG00000065559 | MAP2K4 | turquoise |
| ENSG00000065600 | TMEM206 | turquoise |
| ENSG00000065613 | SLK | turquoise |
| ENSG00000065615 | CYB5R4 | turquoise |
| ENSG00000065665 | SEC61A2 | turquoise |
| ENSG00000065675 | PRKCQ | turquoise |
| ENSG00000065802 | ASB1 | turquoise |
| ENSG00000065809 | FAM107B | turquoise |
| ENSG00000065911 | MTHFD2 | turquoise |
| ENSG00000065923 | SLC9A7 | turquoise |
| ENSG00000065989 | PDE4A | turquoise |
| ENSG00000066027 | PPP2R5A | turquoise |
| ENSG00000066084 | DIP2B | turquoise |
| ENSG00000066117 | SMARCD1 | turquoise |
| ENSG00000066135 | KDM4A | turquoise |
| ENSG00000066185 | ZMYND12 | turquoise |
| ENSG00000066230 | SLC9A3 | turquoise |
| ENSG00000066279 | ASPM | turquoise |
| ENSG00000066322 | ELOVL1 | turquoise |

| | | |
|---|---|---|
| ENSG00000066382 | MPPED2 | turquoise |
| ENSG00000066422 | ZBTB11 | turquoise |
| ENSG00000066455 | GOLGA5 | turquoise |
| ENSG00000066468 | FGFR2 | turquoise |
| ENSG00000066557 | LRRC40 | turquoise |
| ENSG00000066583 | ISOC1 | turquoise |
| ENSG00000066651 | TRMT11 | turquoise |
| ENSG00000066654 | THUMPD1 | turquoise |
| ENSG00000066697 | MSANTD3 | turquoise |
| ENSG00000066739 | ATG2B | turquoise |
| ENSG00000066777 | ARFGEF1 | turquoise |
| ENSG00000066827 | ZFAT | turquoise |
| ENSG00000067064 | IDI1 | turquoise |
| ENSG00000067082 | KLF6 | turquoise |
| ENSG00000067167 | TRAM1 | turquoise |
| ENSG00000067191 | CACNB1 | turquoise |
| ENSG00000067208 | EVI5 | turquoise |
| ENSG00000067248 | DHX29 | turquoise |
| ENSG00000067334 | DNTTIP2 | turquoise |
| ENSG00000067369 | TP53BP1 | turquoise |
| ENSG00000067601 | PMS2P4 | turquoise |
| ENSG00000067606 | PRKCZ | turquoise |
| ENSG00000067704 | IARS2 | turquoise |
| ENSG00000067798 | NAV3 | turquoise |
| ENSG00000067836 | ROGDI | turquoise |
| ENSG00000067900 | ROCK1 | turquoise |
| ENSG00000067955 | CBFB | turquoise |
| ENSG00000068001 | HYAL2 | turquoise |
| ENSG00000068028 | RASSF1 | turquoise |
| ENSG00000068097 | HEATR6 | turquoise |
| ENSG00000068120 | COASY | turquoise |
| ENSG00000068354 | TBC1D25 | turquoise |
| ENSG00000068366 | ACSL4 | turquoise |
| ENSG00000068489 | PRR11 | turquoise |
| ENSG00000068745 | IP6K2 | turquoise |
| ENSG00000068784 | SRBD1 | turquoise |
| ENSG00000068796 | KIF2A | turquoise |
| ENSG00000068831 | RASGRP2 | turquoise |
| ENSG00000068878 | PSME4 | turquoise |
| ENSG00000068885 | IFT80 | turquoise |
| ENSG00000068912 | ERLEC1 | turquoise |
| ENSG00000068971 | PPP2R5B | turquoise |
| ENSG00000068976 | PYGM | turquoise |
| ENSG00000069020 | MAST4 | turquoise |
| ENSG00000069248 | NUP133 | turquoise |
| ENSG00000069275 | NUCKS1 | turquoise |
| ENSG00000069345 | DNAJA2 | turquoise |
| ENSG00000069424 | KCNAB2 | turquoise |

| | | |
|---|---|---|
| ENSG00000069493 | CLEC2D | turquoise |
| ENSG00000069509 | FUNDC1 | turquoise |
| ENSG00000069667 | RORA | turquoise |
| ENSG00000069702 | TGFBR3 | turquoise |
| ENSG00000069869 | NEDD4 | turquoise |
| ENSG00000069943 | PIGB | turquoise |
| ENSG00000069956 | MAPK6 | turquoise |
| ENSG00000069974 | RAB27A | turquoise |
| ENSG00000070010 | UFD1 | turquoise |
| ENSG00000070061 | ELP1 | turquoise |
| ENSG00000070081 | NUCB2 | turquoise |
| ENSG00000070087 | PFN2 | turquoise |
| ENSG00000070190 | DAPP1 | turquoise |
| ENSG00000070214 | SLC44A1 | turquoise |
| ENSG00000070366 | SMG6 | turquoise |
| ENSG00000070367 | EXOC5 | turquoise |
| ENSG00000070371 | CLTCL1 | turquoise |
| ENSG00000070388 | FGF22 | turquoise |
| ENSG00000070404 | FSTL3 | turquoise |
| ENSG00000070495 | JMJD6 | turquoise |
| ENSG00000070756 | PABPC1 | turquoise |
| ENSG00000070770 | CSNK2A2 | turquoise |
| ENSG00000070831 | CDC42 | turquoise |
| ENSG00000070882 | OSBPL3 | turquoise |
| ENSG00000070915 | SLC12A3 | turquoise |
| ENSG00000070961 | ATP2B1 | turquoise |
| ENSG00000070985 | TRPM5 | turquoise |
| ENSG00000071051 | NCK2 | turquoise |
| ENSG00000071073 | MGAT4A | turquoise |
| ENSG00000071082 | RPL31 | turquoise |
| ENSG00000071189 | SNX13 | turquoise |
| ENSG00000071242 | RPS6KA2 | turquoise |
| ENSG00000071243 | ING3 | turquoise |
| ENSG00000071246 | VASH1 | turquoise |
| ENSG00000071282 | LMCD1 | turquoise |
| ENSG00000071537 | SEL1L | turquoise |
| ENSG00000071553 | ATP6AP1 | turquoise |
| ENSG00000071575 | TRIB2 | turquoise |
| ENSG00000071626 | DAZAP1 | turquoise |
| ENSG00000071794 | HLTF | turquoise |
| ENSG00000071894 | CPSF1 | turquoise |
| ENSG00000071909 | MYO3B | turquoise |
| ENSG00000071967 | CYBRD1 | turquoise |
| ENSG00000071994 | PDCD2 | turquoise |
| ENSG00000072042 | RDH11 | turquoise |
| ENSG00000072071 | ADGRL1 | turquoise |
| ENSG00000072135 | PTPN18 | turquoise |
| ENSG00000072201 | LNX1 | turquoise |

| | | |
|---|---|---|
| ENSG00000072210 | ALDH3A2 | turquoise |
| ENSG00000072274 | TFRC | turquoise |
| ENSG00000072364 | AFF4 | turquoise |
| ENSG00000072401 | UBE2D1 | turquoise |
| ENSG00000072415 | MPP5 | turquoise |
| ENSG00000072422 | RHOBTB1 | turquoise |
| ENSG00000072501 | SMC1A | turquoise |
| ENSG00000072736 | NFATC3 | turquoise |
| ENSG00000072756 | TRNT1 | turquoise |
| ENSG00000072778 | ACADVL | turquoise |
| ENSG00000072786 | STK10 | turquoise |
| ENSG00000072803 | FBXW11 | turquoise |
| ENSG00000072818 | ACAP1 | turquoise |
| ENSG00000072832 | CRMP1 | turquoise |
| ENSG00000072840 | EVC | turquoise |
| ENSG00000072952 | MRVI1 | turquoise |
| ENSG00000072954 | TMEM38A | turquoise |
| ENSG00000073008 | PVR | turquoise |
| ENSG00000073060 | SCARB1 | turquoise |
| ENSG00000073169 | SELENOO | turquoise |
| ENSG00000073350 | LLGL2 | turquoise |
| ENSG00000073584 | SMARCE1 | turquoise |
| ENSG00000073605 | GSDMB | turquoise |
| ENSG00000073614 | KDM5A | turquoise |
| ENSG00000073756 | PTGS2 | turquoise |
| ENSG00000073803 | MAP3K13 | turquoise |
| ENSG00000073849 | ST6GAL1 | turquoise |
| ENSG00000073905 | VDAC1P1 | turquoise |
| ENSG00000073910 | FRY | turquoise |
| ENSG00000073921 | PICALM | turquoise |
| ENSG00000073969 | NSF | turquoise |
| ENSG00000074054 | CLASP1 | turquoise |
| ENSG00000074201 | CLNS1A | turquoise |
| ENSG00000074219 | TEAD2 | turquoise |
| ENSG00000074266 | EED | turquoise |
| ENSG00000074276 | CDHR2 | turquoise |
| ENSG00000074370 | ATP2A3 | turquoise |
| ENSG00000074410 | CA12 | turquoise |
| ENSG00000074416 | MGLL | turquoise |
| ENSG00000074527 | NTN4 | turquoise |
| ENSG00000074582 | BCS1L | turquoise |
| ENSG00000074603 | DPP8 | turquoise |
| ENSG00000074657 | ZNF532 | turquoise |
| ENSG00000074695 | LMAN1 | turquoise |
| ENSG00000074696 | HACD3 | turquoise |
| ENSG00000074706 | IPCEF1 | turquoise |
| ENSG00000074855 | ANO8 | turquoise |
| ENSG00000074935 | TUBE1 | turquoise |

| | | |
|---|---|---|
| ENSG00000074966 | TXK | turquoise |
| ENSG00000075089 | ACTR6 | turquoise |
| ENSG00000075131 | TIPIN | turquoise |
| ENSG00000075151 | EIF4G3 | turquoise |
| ENSG00000075188 | NUP37 | turquoise |
| ENSG00000075213 | SEMA3A | turquoise |
| ENSG00000075223 | SEMA3C | turquoise |
| ENSG00000075239 | ACAT1 | turquoise |
| ENSG00000075240 | GRAMD4 | turquoise |
| ENSG00000075290 | WNT8B | turquoise |
| ENSG00000075292 | ZNF638 | turquoise |
| ENSG00000075303 | SLC25A40 | turquoise |
| ENSG00000075391 | RASAL2 | turquoise |
| ENSG00000075407 | ZNF37A | turquoise |
| ENSG00000075420 | FNDC3B | turquoise |
| ENSG00000075539 | FRYL | turquoise |
| ENSG00000075568 | TMEM131 | turquoise |
| ENSG00000075651 | PLD1 | turquoise |
| ENSG00000075702 | WDR62 | turquoise |
| ENSG00000075711 | DLG1 | turquoise |
| ENSG00000075826 | SEC31B | turquoise |
| ENSG00000075856 | SART3 | turquoise |
| ENSG00000075945 | KIFAP3 | turquoise |
| ENSG00000075975 | MKRN2 | turquoise |
| ENSG00000076053 | RBM7 | turquoise |
| ENSG00000076067 | RBMS2 | turquoise |
| ENSG00000076201 | PTPN23 | turquoise |
| ENSG00000076258 | FMO4 | turquoise |
| ENSG00000076351 | SLC46A1 | turquoise |
| ENSG00000076554 | TPD52 | turquoise |
| ENSG00000076604 | TRAF4 | turquoise |
| ENSG00000076641 | PAG1 | turquoise |
| ENSG00000076706 | MCAM | turquoise |
| ENSG00000076716 | GPC4 | turquoise |
| ENSG00000076826 | CAMSAP3 | turquoise |
| ENSG00000076928 | ARHGEF1 | turquoise |
| ENSG00000076944 | STXBP2 | turquoise |
| ENSG00000076984 | MAP2K7 | turquoise |
| ENSG00000077092 | RARB | turquoise |
| ENSG00000077097 | TOP2B | turquoise |
| ENSG00000077147 | TM9SF3 | turquoise |
| ENSG00000077150 | NFKB2 | turquoise |
| ENSG00000077232 | DNAJC10 | turquoise |
| ENSG00000077254 | USP33 | turquoise |
| ENSG00000077380 | DYNC1I2 | turquoise |
| ENSG00000077454 | LRCH4 | turquoise |
| ENSG00000077458 | FAM76B | turquoise |
| ENSG00000077514 | POLD3 | turquoise |

| | | |
|---|---|---|
| ENSG00000077585 | GPR137B | turquoise |
| ENSG00000077616 | NAALAD2 | turquoise |
| ENSG00000077684 | JADE1 | turquoise |
| ENSG00000077713 | SLC25A43 | turquoise |
| ENSG00000077721 | UBE2A | turquoise |
| ENSG00000077782 | FGFR1 | turquoise |
| ENSG00000077984 | CST7 | turquoise |
| ENSG00000078018 | MAP2 | turquoise |
| ENSG00000078043 | PIAS2 | turquoise |
| ENSG00000078061 | ARAF | turquoise |
| ENSG00000078070 | MCCC1 | turquoise |
| ENSG00000078114 | NEBL | turquoise |
| ENSG00000078140 | UBE2K | turquoise |
| ENSG00000078142 | PIK3C3 | turquoise |
| ENSG00000078177 | N4BP2 | turquoise |
| ENSG00000078237 | TIGAR | turquoise |
| ENSG00000078246 | TULP3 | turquoise |
| ENSG00000078269 | SYNJ2 | turquoise |
| ENSG00000078304 | PPP2R5C | turquoise |
| ENSG00000078399 | HOXA9 | turquoise |
| ENSG00000078401 | EDN1 | turquoise |
| ENSG00000078403 | MLLT10 | turquoise |
| ENSG00000078589 | P2RY10 | turquoise |
| ENSG00000078596 | ITM2A | turquoise |
| ENSG00000078674 | PCM1 | turquoise |
| ENSG00000078687 | TNRC6C | turquoise |
| ENSG00000078747 | ITCH | turquoise |
| ENSG00000078808 | SDF4 | turquoise |
| ENSG00000078902 | TOLLIP | turquoise |
| ENSG00000078967 | UBE2D4 | turquoise |
| ENSG00000079134 | THOC1 | turquoise |
| ENSG00000079150 | FKBP7 | turquoise |
| ENSG00000079215 | SLC1A3 | turquoise |
| ENSG00000079246 | XRCC5 | turquoise |
| ENSG00000079257 | LXN | turquoise |
| ENSG00000079313 | REXO1 | turquoise |
| ENSG00000079332 | SAR1A | turquoise |
| ENSG00000079335 | CDC14A | turquoise |
| ENSG00000079337 | RAPGEF3 | turquoise |
| ENSG00000079387 | SENP1 | turquoise |
| ENSG00000079432 | CIC | turquoise |
| ENSG00000079435 | LIPE | turquoise |
| ENSG00000079482 | OPHN1 | turquoise |
| ENSG00000079616 | KIF22 | turquoise |
| ENSG00000079739 | PGM1 | turquoise |
| ENSG00000079785 | DDX1 | turquoise |
| ENSG00000079819 | EPB41L2 | turquoise |
| ENSG00000079931 | MOXD1 | turquoise |

| | | |
|---|---|---|
| ENSG00000079950 | STX7 | turquoise |
| ENSG00000079974 | RABL2B | turquoise |
| ENSG00000080166 | DCT | turquoise |
| ENSG00000080298 | RFX3 | turquoise |
| ENSG00000080345 | RIF1 | turquoise |
| ENSG00000080371 | RAB21 | turquoise |
| ENSG00000080493 | SLC4A4 | turquoise |
| ENSG00000080503 | SMARCA2 | turquoise |
| ENSG00000080546 | SESN1 | turquoise |
| ENSG00000080561 | MID2 | turquoise |
| ENSG00000080608 | PUM3 | turquoise |
| ENSG00000080802 | CNOT4 | turquoise |
| ENSG00000080819 | CPOX | turquoise |
| ENSG00000080822 | CLDND1 | turquoise |
| ENSG00000080823 | MOK | turquoise |
| ENSG00000080824 | HSP90AA1 | turquoise |
| ENSG00000080839 | RBL1 | turquoise |
| ENSG00000080845 | DLGAP4 | turquoise |
| ENSG00000080986 | NDC80 | turquoise |
| ENSG00000081014 | AP4E1 | turquoise |
| ENSG00000081019 | RSBN1 | turquoise |
| ENSG00000081087 | OSTM1 | turquoise |
| ENSG00000081154 | PCNP | turquoise |
| ENSG00000081177 | EXD2 | turquoise |
| ENSG00000081181 | ARG2 | turquoise |
| ENSG00000081189 | MEF2C | turquoise |
| ENSG00000081237 | PTPRC | turquoise |
| ENSG00000081307 | UBA5 | turquoise |
| ENSG00000081320 | STK17B | turquoise |
| ENSG00000081377 | CDC14B | turquoise |
| ENSG00000081386 | ZNF510 | turquoise |
| ENSG00000081665 | ZNF506 | turquoise |
| ENSG00000081692 | JMJD4 | turquoise |
| ENSG00000081721 | DUSP12 | turquoise |
| ENSG00000081791 | KIAA0141 | turquoise |
| ENSG00000081870 | HSPB11 | turquoise |
| ENSG00000082074 | FYB1 | turquoise |
| ENSG00000082153 | BZW1 | turquoise |
| ENSG00000082196 | C1QTNF3 | turquoise |
| ENSG00000082212 | ME2 | turquoise |
| ENSG00000082213 | C5orf22 | turquoise |
| ENSG00000082258 | CCNT2 | turquoise |
| ENSG00000082269 | FAM135A | turquoise |
| ENSG00000082438 | COBLL1 | turquoise |
| ENSG00000082458 | DLG3 | turquoise |
| ENSG00000082512 | TRAF5 | turquoise |
| ENSG00000082515 | MRPL22 | turquoise |
| ENSG00000082516 | GEMIN5 | turquoise |

| | | |
|---|---|---|
| ENSG00000082701 | GSK3B | turquoise |
| ENSG00000082898 | XPO1 | turquoise |
| ENSG00000082996 | RNF13 | turquoise |
| ENSG00000083093 | PALB2 | turquoise |
| ENSG00000083097 | DOPEY1 | turquoise |
| ENSG00000083099 | LYRM2 | turquoise |
| ENSG00000083123 | BCKDHB | turquoise |
| ENSG00000083168 | KAT6A | turquoise |
| ENSG00000083223 | ZCCHC6 | turquoise |
| ENSG00000083312 | TNPO1 | turquoise |
| ENSG00000083444 | PLOD1 | turquoise |
| ENSG00000083454 | P2RX5 | turquoise |
| ENSG00000083520 | DIS3 | turquoise |
| ENSG00000083535 | PIBF1 | turquoise |
| ENSG00000083544 | TDRD3 | turquoise |
| ENSG00000083642 | PDS5B | turquoise |
| ENSG00000083720 | OXCT1 | turquoise |
| ENSG00000083750 | RRAGB | turquoise |
| ENSG00000083799 | CYLD | turquoise |
| ENSG00000083807 | SLC27A5 | turquoise |
| ENSG00000083812 | ZNF324 | turquoise |
| ENSG00000083838 | ZNF446 | turquoise |
| ENSG00000083896 | YTHDC1 | turquoise |
| ENSG00000083937 | CHMP2B | turquoise |
| ENSG00000084073 | ZMPSTE24 | turquoise |
| ENSG00000084093 | REST | turquoise |
| ENSG00000084112 | SSH1 | turquoise |
| ENSG00000084463 | WBP11 | turquoise |
| ENSG00000084652 | TXLNA | turquoise |
| ENSG00000084676 | NCOA1 | turquoise |
| ENSG00000084693 | AGBL5 | turquoise |
| ENSG00000084733 | RAB10 | turquoise |
| ENSG00000084764 | MAPRE3 | turquoise |
| ENSG00000085117 | CD82 | turquoise |
| ENSG00000085224 | ATRX | turquoise |
| ENSG00000085274 | MYNN | turquoise |
| ENSG00000085365 | SCAMP1 | turquoise |
| ENSG00000085377 | PREP | turquoise |
| ENSG00000085382 | HACE1 | turquoise |
| ENSG00000085415 | SEH1L | turquoise |
| ENSG00000085433 | WDR47 | turquoise |
| ENSG00000085449 | WDFY1 | turquoise |
| ENSG00000085465 | OVGP1 | turquoise |
| ENSG00000085511 | MAP3K4 | turquoise |
| ENSG00000085563 | ABCB1 | turquoise |
| ENSG00000085644 | ZNF213 | turquoise |
| ENSG00000085719 | CPNE3 | turquoise |
| ENSG00000085721 | RRN3 | turquoise |

| | | |
|---|---|---|
| ENSG00000085760 | MTIF2 | turquoise |
| ENSG00000085788 | DDHD2 | turquoise |
| ENSG00000085832 | EPS15 | turquoise |
| ENSG00000085840 | ORC1 | turquoise |
| ENSG00000085872 | CHERP | turquoise |
| ENSG00000085982 | USP40 | turquoise |
| ENSG00000085998 | POMGNT1 | turquoise |
| ENSG00000086015 | MAST2 | turquoise |
| ENSG00000086061 | DNAJA1 | turquoise |
| ENSG00000086065 | CHMP5 | turquoise |
| ENSG00000086102 | NFX1 | turquoise |
| ENSG00000086189 | DIMT1 | turquoise |
| ENSG00000086200 | IPO11 | turquoise |
| ENSG00000086289 | EPDR1 | turquoise |
| ENSG00000086300 | SNX10 | turquoise |
| ENSG00000086544 | ITPKC | turquoise |
| ENSG00000086589 | RBM22 | turquoise |
| ENSG00000086598 | TMED2 | turquoise |
| ENSG00000086619 | ERO1B | turquoise |
| ENSG00000086666 | ZFAND6 | turquoise |
| ENSG00000086712 | TXLNG | turquoise |
| ENSG00000086717 | PPEF1 | turquoise |
| ENSG00000086827 | ZW10 | turquoise |
| ENSG00000086848 | ALG9 | turquoise |
| ENSG00000087008 | ACOX3 | turquoise |
| ENSG00000087053 | MTMR2 | turquoise |
| ENSG00000087077 | TRIP6 | turquoise |
| ENSG00000087095 | NLK | turquoise |
| ENSG00000087111 | PIGS | turquoise |
| ENSG00000087152 | ATXN7L3 | turquoise |
| ENSG00000087253 | LPCAT2 | turquoise |
| ENSG00000087299 | L2HGDH | turquoise |
| ENSG00000087301 | TXNDC16 | turquoise |
| ENSG00000087302 | C14orf166 | turquoise |
| ENSG00000087338 | GMCL1 | turquoise |
| ENSG00000087448 | KLHL42 | turquoise |
| ENSG00000087470 | DNM1L | turquoise |
| ENSG00000087495 | PHACTR3 | turquoise |
| ENSG00000087502 | ERGIC2 | turquoise |
| ENSG00000087586 | AURKA | turquoise |
| ENSG00000087903 | RFX2 | turquoise |
| ENSG00000088035 | ALG6 | turquoise |
| ENSG00000088179 | PTPN4 | turquoise |
| ENSG00000088205 | DDX18 | turquoise |
| ENSG00000088256 | GNA11 | turquoise |
| ENSG00000088305 | DNMT3B | turquoise |
| ENSG00000088340 | FER1L4 | turquoise |
| ENSG00000088356 | PDRG1 | turquoise |

| | | |
|---|---|---|
| ENSG00000088367 | EPB41L1 | turquoise |
| ENSG00000088387 | DOCK9 | turquoise |
| ENSG00000088448 | ANKRD10 | turquoise |
| ENSG00000088451 | TGDS | turquoise |
| ENSG00000088543 | C3orf18 | turquoise |
| ENSG00000088727 | KIF9 | turquoise |
| ENSG00000088766 | CRLS1 | turquoise |
| ENSG00000088808 | PPP1R13B | turquoise |
| ENSG00000088812 | ATRN | turquoise |
| ENSG00000088833 | NSFL1C | turquoise |
| ENSG00000088854 | C20orf194 | turquoise |
| ENSG00000088876 | ZNF343 | turquoise |
| ENSG00000088881 | EBF4 | turquoise |
| ENSG00000088930 | XRN2 | turquoise |
| ENSG00000088970 | KIZ | turquoise |
| ENSG00000089006 | SNX5 | turquoise |
| ENSG00000089009 | RPL6 | turquoise |
| ENSG00000089022 | MAPKAPK5 | turquoise |
| ENSG00000089041 | P2RX7 | turquoise |
| ENSG00000089048 | ESF1 | turquoise |
| ENSG00000089050 | RBBP9 | turquoise |
| ENSG00000089053 | ANAPC5 | turquoise |
| ENSG00000089057 | SLC23A2 | turquoise |
| ENSG00000089060 | SLC8B1 | turquoise |
| ENSG00000089063 | TMEM230 | turquoise |
| ENSG00000089123 | TASP1 | turquoise |
| ENSG00000089169 | RPH3A | turquoise |
| ENSG00000089177 | KIF16B | turquoise |
| ENSG00000089195 | TRMT6 | turquoise |
| ENSG00000089280 | FUS | turquoise |
| ENSG00000089289 | IGBP1 | turquoise |
| ENSG00000089335 | ZNF302 | turquoise |
| ENSG00000089682 | RBM41 | turquoise |
| ENSG00000089693 | MLF2 | turquoise |
| ENSG00000089775 | ZBTB25 | turquoise |
| ENSG00000089818 | NECAP1 | turquoise |
| ENSG00000089820 | ARHGAP4 | turquoise |
| ENSG00000089847 | ANKRD24 | turquoise |
| ENSG00000089876 | DHX32 | turquoise |
| ENSG00000089902 | RCOR1 | turquoise |
| ENSG00000090006 | LTBP4 | turquoise |
| ENSG00000090054 | SPTLC1 | turquoise |
| ENSG00000090060 | PAPOLA | turquoise |
| ENSG00000090097 | PCBP4 | turquoise |
| ENSG00000090104 | RGS1 | turquoise |
| ENSG00000090238 | YPEL3 | turquoise |
| ENSG00000090263 | MRPS33 | turquoise |
| ENSG00000090372 | STRN4 | turquoise |

| | | |
|---|---|---|
| ENSG00000090376 | IRAK3 | turquoise |
| ENSG00000090382 | LYZ | turquoise |
| ENSG00000090432 | MUL1 | turquoise |
| ENSG00000090470 | PDCD7 | turquoise |
| ENSG00000090520 | DNAJB11 | turquoise |
| ENSG00000090554 | FLT3LG | turquoise |
| ENSG00000090581 | GNPTG | turquoise |
| ENSG00000090612 | ZNF268 | turquoise |
| ENSG00000090661 | CERS4 | turquoise |
| ENSG00000090674 | MCOLN1 | turquoise |
| ENSG00000090686 | USP48 | turquoise |
| ENSG00000090776 | EFNB1 | turquoise |
| ENSG00000090889 | KIF4A | turquoise |
| ENSG00000090905 | TNRC6A | turquoise |
| ENSG00000090924 | PLEKHG2 | turquoise |
| ENSG00000090989 | EXOC1 | turquoise |
| ENSG00000091009 | RBM27 | turquoise |
| ENSG00000091039 | OSBPL8 | turquoise |
| ENSG00000091106 | NLRC4 | turquoise |
| ENSG00000091127 | PUS7 | turquoise |
| ENSG00000091129 | NRCAM | turquoise |
| ENSG00000091140 | DLD | turquoise |
| ENSG00000091157 | WDR7 | turquoise |
| ENSG00000091262 | ABCC6 | turquoise |
| ENSG00000091317 | CMTM6 | turquoise |
| ENSG00000091409 | ITGA6 | turquoise |
| ENSG00000091436 | MAP3K20 | turquoise |
| ENSG00000091490 | SEL1L3 | turquoise |
| ENSG00000091527 | CDV3 | turquoise |
| ENSG00000091536 | MYO15A | turquoise |
| ENSG00000091651 | ORC6 | turquoise |
| ENSG00000091844 | RGS17 | turquoise |
| ENSG00000091972 | CD200 | turquoise |
| ENSG00000092020 | PPP2R3C | turquoise |
| ENSG00000092051 | JPH4 | turquoise |
| ENSG00000092094 | OSGEP | turquoise |
| ENSG00000092108 | SCFD1 | turquoise |
| ENSG00000092140 | G2E3 | turquoise |
| ENSG00000092148 | HECTD1 | turquoise |
| ENSG00000092199 | HNRNPC | turquoise |
| ENSG00000092201 | SUPT16H | turquoise |
| ENSG00000092208 | GEMIN2 | turquoise |
| ENSG00000092295 | TGM1 | turquoise |
| ENSG00000092439 | TRPM7 | turquoise |
| ENSG00000092470 | WDR76 | turquoise |
| ENSG00000092531 | SNAP23 | turquoise |
| ENSG00000092850 | TEKT2 | turquoise |
| ENSG00000092871 | RFFL | turquoise |

| | | |
|---|---|---|
| ENSG00000092931 | MFSD11 | turquoise |
| ENSG00000092964 | DPYSL2 | turquoise |
| ENSG00000092978 | GPATCH2 | turquoise |
| ENSG00000093000 | NUP50 | turquoise |
| ENSG00000093144 | ECHDC1 | turquoise |
| ENSG00000093183 | SEC22C | turquoise |
| ENSG00000093217 | XYLB | turquoise |
| ENSG00000094631 | HDAC6 | turquoise |
| ENSG00000094804 | CDC6 | turquoise |
| ENSG00000094841 | UPRT | turquoise |
| ENSG00000094880 | CDC23 | turquoise |
| ENSG00000094975 | SUCO | turquoise |
| ENSG00000095002 | MSH2 | turquoise |
| ENSG00000095015 | MAP3K1 | turquoise |
| ENSG00000095139 | ARCN1 | turquoise |
| ENSG00000095209 | TMEM38B | turquoise |
| ENSG00000095261 | PSMD5 | turquoise |
| ENSG00000095321 | CRAT | turquoise |
| ENSG00000095383 | TBC1D2 | turquoise |
| ENSG00000095397 | WHRN | turquoise |
| ENSG00000095485 | CWF19L1 | turquoise |
| ENSG00000095564 | BTAF1 | turquoise |
| ENSG00000095574 | IKZF5 | turquoise |
| ENSG00000095637 | SORBS1 | turquoise |
| ENSG00000095739 | BAMBI | turquoise |
| ENSG00000095787 | WAC | turquoise |
| ENSG00000095794 | CREM | turquoise |
| ENSG00000095951 | HIVEP1 | turquoise |
| ENSG00000095970 | TREM2 | turquoise |
| ENSG00000096060 | FKBP5 | turquoise |
| ENSG00000096063 | SRPK1 | turquoise |
| ENSG00000096080 | MRPS18A | turquoise |
| ENSG00000096092 | TMEM14A | turquoise |
| ENSG00000096401 | CDC5L | turquoise |
| ENSG00000096654 | ZNF184 | turquoise |
| ENSG00000096696 | DSP | turquoise |
| ENSG00000096717 | SIRT1 | turquoise |
| ENSG00000096746 | HNRNPH3 | turquoise |
| ENSG00000096872 | IFT74 | turquoise |
| ENSG00000096968 | JAK2 | turquoise |
| ENSG00000096996 | IL12RB1 | turquoise |
| ENSG00000097033 | SH3GLB1 | turquoise |
| ENSG00000097046 | CDC7 | turquoise |
| ENSG00000099139 | PCSK5 | turquoise |
| ENSG00000099194 | SCD | turquoise |
| ENSG00000099203 | TMED1 | turquoise |
| ENSG00000099219 | ERMP1 | turquoise |
| ENSG00000099246 | RAB18 | turquoise |

| | | |
|---|---|---|
| ENSG00000099256 | PRTFDC1 | turquoise |
| ENSG00000099282 | TSPAN15 | turquoise |
| ENSG00000099290 | WASHC2A | turquoise |
| ENSG00000099326 | MZF1 | turquoise |
| ENSG00000099337 | KCNK6 | turquoise |
| ENSG00000099338 | CATSPERG | turquoise |
| ENSG00000099364 | FBXL19 | turquoise |
| ENSG00000099365 | STX1B | turquoise |
| ENSG00000099622 | CIRBP | turquoise |
| ENSG00000099725 | PRKY | turquoise |
| ENSG00000099769 | IGFALS | turquoise |
| ENSG00000099785 | 2-Mar | turquoise |
| ENSG00000099810 | MTAP | turquoise |
| ENSG00000099822 | HCN2 | turquoise |
| ENSG00000099840 | IZUMO4 | turquoise |
| ENSG00000099849 | RASSF7 | turquoise |
| ENSG00000099864 | PALM | turquoise |
| ENSG00000099866 | MADCAM1 | turquoise |
| ENSG00000099899 | TRMT2A | turquoise |
| ENSG00000099904 | ZDHHC8 | turquoise |
| ENSG00000099910 | KLHL22 | turquoise |
| ENSG00000099917 | MED15 | turquoise |
| ENSG00000099942 | CRKL | turquoise |
| ENSG00000099949 | LZTR1 | turquoise |
| ENSG00000099954 | CECR2 | turquoise |
| ENSG00000099968 | BCL2L13 | turquoise |
| ENSG00000099985 | OSM | turquoise |
| ENSG00000099991 | CABIN1 | turquoise |
| ENSG00000099992 | TBC1D10A | turquoise |
| ENSG00000099994 | SUSD2 | turquoise |
| ENSG00000099999 | RNF215 | turquoise |
| ENSG00000100030 | MAPK1 | turquoise |
| ENSG00000100031 | GGT1 | turquoise |
| ENSG00000100056 | ESS2 | turquoise |
| ENSG00000100058 | CRYBB2P1 | turquoise |
| ENSG00000100068 | LRP5L | turquoise |
| ENSG00000100083 | GGA1 | turquoise |
| ENSG00000100092 | SH3BP1 | turquoise |
| ENSG00000100099 | HPS4 | turquoise |
| ENSG00000100106 | TRIOBP | turquoise |
| ENSG00000100109 | TFIP11 | turquoise |
| ENSG00000100139 | MICALL1 | turquoise |
| ENSG00000100150 | DEPDC5 | turquoise |
| ENSG00000100151 | PICK1 | turquoise |
| ENSG00000100154 | TTC28 | turquoise |
| ENSG00000100156 | SLC16A8 | turquoise |
| ENSG00000100167 | 3-Sep | turquoise |
| ENSG00000100181 | TPTEP1 | turquoise |

| | | |
|---|---|---|
| ENSG00000100197 | CYP2D6 | turquoise |
| ENSG00000100218 | RSPH14 | turquoise |
| ENSG00000100221 | JOSD1 | turquoise |
| ENSG00000100228 | RAB36 | turquoise |
| ENSG00000100239 | PPP6R2 | turquoise |
| ENSG00000100241 | SBF1 | turquoise |
| ENSG00000100246 | DNAL4 | turquoise |
| ENSG00000100258 | LMF2 | turquoise |
| ENSG00000100263 | RHBDD3 | turquoise |
| ENSG00000100271 | TTLL1 | turquoise |
| ENSG00000100276 | RASL10A | turquoise |
| ENSG00000100281 | HMGXB4 | turquoise |
| ENSG00000100284 | TOM1 | turquoise |
| ENSG00000100288 | CHKB | turquoise |
| ENSG00000100294 | MCAT | turquoise |
| ENSG00000100299 | ARSA | turquoise |
| ENSG00000100300 | TSPO | turquoise |
| ENSG00000100304 | TTLL12 | turquoise |
| ENSG00000100307 | CBX7 | turquoise |
| ENSG00000100314 | CABP7 | turquoise |
| ENSG00000100319 | ZMAT5 | turquoise |
| ENSG00000100321 | SYNGR1 | turquoise |
| ENSG00000100335 | MIEF1 | turquoise |
| ENSG00000100350 | FOXRED2 | turquoise |
| ENSG00000100359 | SGSM3 | turquoise |
| ENSG00000100360 | IFT27 | turquoise |
| ENSG00000100368 | CSF2RB | turquoise |
| ENSG00000100379 | KCTD17 | turquoise |
| ENSG00000100387 | RBX1 | turquoise |
| ENSG00000100399 | CHADL | turquoise |
| ENSG00000100403 | ZC3H7B | turquoise |
| ENSG00000100410 | PHF5A | turquoise |
| ENSG00000100413 | POLR3H | turquoise |
| ENSG00000100416 | TRMU | turquoise |
| ENSG00000100417 | PMM1 | turquoise |
| ENSG00000100418 | DESI1 | turquoise |
| ENSG00000100425 | BRD1 | turquoise |
| ENSG00000100426 | ZBED4 | turquoise |
| ENSG00000100433 | KCNK10 | turquoise |
| ENSG00000100439 | ABHD4 | turquoise |
| ENSG00000100442 | FKBP3 | turquoise |
| ENSG00000100445 | SDR39U1 | turquoise |
| ENSG00000100462 | PRMT5 | turquoise |
| ENSG00000100478 | AP4S1 | turquoise |
| ENSG00000100483 | VCPKMT | turquoise |
| ENSG00000100485 | SOS2 | turquoise |
| ENSG00000100503 | NIN | turquoise |
| ENSG00000100519 | PSMC6 | turquoise |

| | | |
|---|---|---|
| ENSG00000100522 | GNPNAT1 | turquoise |
| ENSG00000100523 | DDHD1 | turquoise |
| ENSG00000100528 | CNIH1 | turquoise |
| ENSG00000100532 | CGRRF1 | turquoise |
| ENSG00000100554 | ATP6V1D | turquoise |
| ENSG00000100564 | PIGH | turquoise |
| ENSG00000100567 | PSMA3 | turquoise |
| ENSG00000100568 | VTI1B | turquoise |
| ENSG00000100575 | TIMM9 | turquoise |
| ENSG00000100577 | GSTZ1 | turquoise |
| ENSG00000100578 | KIAA0586 | turquoise |
| ENSG00000100580 | TMED8 | turquoise |
| ENSG00000100592 | DAAM1 | turquoise |
| ENSG00000100599 | RIN3 | turquoise |
| ENSG00000100603 | SNW1 | turquoise |
| ENSG00000100605 | ITPK1 | turquoise |
| ENSG00000100614 | PPM1A | turquoise |
| ENSG00000100628 | ASB2 | turquoise |
| ENSG00000100629 | CEP128 | turquoise |
| ENSG00000100632 | ERH | turquoise |
| ENSG00000100644 | HIF1A | turquoise |
| ENSG00000100647 | SUSD6 | turquoise |
| ENSG00000100650 | SRSF5 | turquoise |
| ENSG00000100664 | EIF5 | turquoise |
| ENSG00000100678 | SLC8A3 | turquoise |
| ENSG00000100714 | MTHFD1 | turquoise |
| ENSG00000100726 | TELO2 | turquoise |
| ENSG00000100744 | GSKIP | turquoise |
| ENSG00000100749 | VRK1 | turquoise |
| ENSG00000100764 | PSMC1 | turquoise |
| ENSG00000100767 | PAPLN | turquoise |
| ENSG00000100796 | PPP4R3A | turquoise |
| ENSG00000100811 | YY1 | turquoise |
| ENSG00000100813 | ACIN1 | turquoise |
| ENSG00000100815 | TRIP11 | turquoise |
| ENSG00000100852 | ARHGAP5 | turquoise |
| ENSG00000100865 | CINP | turquoise |
| ENSG00000100883 | SRP54 | turquoise |
| ENSG00000100906 | NFKBIA | turquoise |
| ENSG00000100916 | BRMS1L | turquoise |
| ENSG00000100934 | SEC23A | turquoise |
| ENSG00000100941 | PNN | turquoise |
| ENSG00000100949 | RABGGTA | turquoise |
| ENSG00000100979 | PLTP | turquoise |
| ENSG00000100982 | PCIF1 | turquoise |
| ENSG00000100994 | PYGB | turquoise |
| ENSG00000101000 | PROCR | turquoise |
| ENSG00000101003 | GINS1 | turquoise |

| | | |
|---|---|---|
| ENSG00000101004 | NINL | turquoise |
| ENSG00000101019 | UQCC1 | turquoise |
| ENSG00000101040 | ZMYND8 | turquoise |
| ENSG00000101049 | SGK2 | turquoise |
| ENSG00000101084 | C20orf24 | turquoise |
| ENSG00000101096 | NFATC2 | turquoise |
| ENSG00000101109 | STK4 | turquoise |
| ENSG00000101115 | SALL4 | turquoise |
| ENSG00000101126 | ADNP | turquoise |
| ENSG00000101132 | PFDN4 | turquoise |
| ENSG00000101150 | TPD52L2 | turquoise |
| ENSG00000101162 | TUBB1 | turquoise |
| ENSG00000101166 | PRELID3B | turquoise |
| ENSG00000101189 | MRGBP | turquoise |
| ENSG00000101190 | TCFL5 | turquoise |
| ENSG00000101194 | SLC17A9 | turquoise |
| ENSG00000101199 | ARFGAP1 | turquoise |
| ENSG00000101213 | PTK6 | turquoise |
| ENSG00000101216 | GMEB2 | turquoise |
| ENSG00000101230 | ISM1 | turquoise |
| ENSG00000101247 | NDUFAF5 | turquoise |
| ENSG00000101255 | TRIB3 | turquoise |
| ENSG00000101266 | CSNK2A1 | turquoise |
| ENSG00000101282 | RSPO4 | turquoise |
| ENSG00000101294 | HM13 | turquoise |
| ENSG00000101307 | SIRPB1 | turquoise |
| ENSG00000101310 | SEC23B | turquoise |
| ENSG00000101335 | MYL9 | turquoise |
| ENSG00000101343 | CRNKL1 | turquoise |
| ENSG00000101346 | POFUT1 | turquoise |
| ENSG00000101350 | KIF3B | turquoise |
| ENSG00000101361 | NOP56 | turquoise |
| ENSG00000101363 | MANBAL | turquoise |
| ENSG00000101367 | MAPRE1 | turquoise |
| ENSG00000101384 | JAG1 | turquoise |
| ENSG00000101391 | CDK5RAP1 | turquoise |
| ENSG00000101400 | SNTA1 | turquoise |
| ENSG00000101405 | OXT | turquoise |
| ENSG00000101407 | TTI1 | turquoise |
| ENSG00000101413 | RPRD1B | turquoise |
| ENSG00000101417 | PXMP4 | turquoise |
| ENSG00000101442 | ACTR5 | turquoise |
| ENSG00000101445 | PPP1R16B | turquoise |
| ENSG00000101447 | FAM83D | turquoise |
| ENSG00000101452 | DHX35 | turquoise |
| ENSG00000101473 | ACOT8 | turquoise |
| ENSG00000101544 | ADNP2 | turquoise |
| ENSG00000101546 | RBFA | turquoise |

| | | |
|---|---|---|
| ENSG00000101557 | USP14 | turquoise |
| ENSG00000101558 | VAPA | turquoise |
| ENSG00000101574 | METTL4 | turquoise |
| ENSG00000101577 | LPIN2 | turquoise |
| ENSG00000101596 | SMCHD1 | turquoise |
| ENSG00000101624 | CEP76 | turquoise |
| ENSG00000101639 | CEP192 | turquoise |
| ENSG00000101665 | SMAD7 | turquoise |
| ENSG00000101695 | RNF125 | turquoise |
| ENSG00000101745 | ANKRD12 | turquoise |
| ENSG00000101751 | POLI | turquoise |
| ENSG00000101752 | MIB1 | turquoise |
| ENSG00000101773 | RBBP8 | turquoise |
| ENSG00000101782 | RIOK3 | turquoise |
| ENSG00000101843 | PSMD10 | turquoise |
| ENSG00000101844 | ATG4A | turquoise |
| ENSG00000101856 | PGRMC1 | turquoise |
| ENSG00000101868 | POLA1 | turquoise |
| ENSG00000101882 | NKAP | turquoise |
| ENSG00000101888 | NXT2 | turquoise |
| ENSG00000101901 | ALG13 | turquoise |
| ENSG00000101911 | PRPS2 | turquoise |
| ENSG00000101916 | TLR8 | turquoise |
| ENSG00000101928 | MOSPD1 | turquoise |
| ENSG00000101935 | AMMECR1 | turquoise |
| ENSG00000101940 | WDR13 | turquoise |
| ENSG00000101966 | XIAP | turquoise |
| ENSG00000101972 | STAG2 | turquoise |
| ENSG00000101974 | ATP11C | turquoise |
| ENSG00000101977 | MCF2 | turquoise |
| ENSG00000101986 | ABCD1 | turquoise |
| ENSG00000102001 | CACNA1F | turquoise |
| ENSG00000102003 | SYP | turquoise |
| ENSG00000102007 | PLP2 | turquoise |
| ENSG00000102024 | PLS3 | turquoise |
| ENSG00000102030 | NAA10 | turquoise |
| ENSG00000102032 | RENBP | turquoise |
| ENSG00000102038 | SMARCA1 | turquoise |
| ENSG00000102043 | MTMR8 | turquoise |
| ENSG00000102048 | ASB9 | turquoise |
| ENSG00000102053 | ZC3H12B | turquoise |
| ENSG00000102078 | SLC25A14 | turquoise |
| ENSG00000102081 | FMR1 | turquoise |
| ENSG00000102119 | EMD | turquoise |
| ENSG00000102125 | TAZ | turquoise |
| ENSG00000102158 | MAGT1 | turquoise |
| ENSG00000102172 | SMS | turquoise |
| ENSG00000102174 | PHEX | turquoise |

| | | |
|---|---|---|
| ENSG00000102189 | EEA1 | turquoise |
| ENSG00000102218 | RP2 | turquoise |
| ENSG00000102221 | JADE3 | turquoise |
| ENSG00000102225 | CDK16 | turquoise |
| ENSG00000102241 | HTATSF1 | turquoise |
| ENSG00000102265 | TIMP1 | turquoise |
| ENSG00000102309 | PIN4 | turquoise |
| ENSG00000102312 | PORCN | turquoise |
| ENSG00000102317 | RBM3 | turquoise |
| ENSG00000102362 | SYTL4 | turquoise |
| ENSG00000102383 | ZDHHC15 | turquoise |
| ENSG00000102384 | CENPI | turquoise |
| ENSG00000102401 | ARMCX3 | turquoise |
| ENSG00000102445 | RUBCNL | turquoise |
| ENSG00000102471 | NDFIP2 | turquoise |
| ENSG00000102531 | FNDC3A | turquoise |
| ENSG00000102543 | CDADC1 | turquoise |
| ENSG00000102547 | CAB39L | turquoise |
| ENSG00000102554 | KLF5 | turquoise |
| ENSG00000102572 | STK24 | turquoise |
| ENSG00000102580 | DNAJC3 | turquoise |
| ENSG00000102595 | UGGT2 | turquoise |
| ENSG00000102606 | ARHGEF7 | turquoise |
| ENSG00000102738 | MRPS31 | turquoise |
| ENSG00000102753 | KPNA3 | turquoise |
| ENSG00000102763 | VWA8 | turquoise |
| ENSG00000102781 | KATNAL1 | turquoise |
| ENSG00000102804 | TSC22D1 | turquoise |
| ENSG00000102871 | TRADD | turquoise |
| ENSG00000102878 | HSF4 | turquoise |
| ENSG00000102879 | CORO1A | turquoise |
| ENSG00000102882 | MAPK3 | turquoise |
| ENSG00000102886 | GDPD3 | turquoise |
| ENSG00000102890 | ELMO3 | turquoise |
| ENSG00000102893 | PHKB | turquoise |
| ENSG00000102900 | NUP93 | turquoise |
| ENSG00000102974 | CTCF | turquoise |
| ENSG00000102978 | POLR2C | turquoise |
| ENSG00000102981 | PARD6A | turquoise |
| ENSG00000102984 | ZNF821 | turquoise |
| ENSG00000103034 | NDRG4 | turquoise |
| ENSG00000103042 | SLC38A7 | turquoise |
| ENSG00000103043 | VAC14 | turquoise |
| ENSG00000103047 | TANGO6 | turquoise |
| ENSG00000103051 | COG4 | turquoise |
| ENSG00000103066 | PLA2G15 | turquoise |
| ENSG00000103091 | WDR59 | turquoise |
| ENSG00000103121 | CMC2 | turquoise |

| | | |
|---|---|---|
| ENSG00000103126 | AXIN1 | turquoise |
| ENSG00000103150 | MLYCD | turquoise |
| ENSG00000103154 | NECAB2 | turquoise |
| ENSG00000103160 | HSDL1 | turquoise |
| ENSG00000103168 | TAF1C | turquoise |
| ENSG00000103197 | TSC2 | turquoise |
| ENSG00000103199 | ZNF500 | turquoise |
| ENSG00000103202 | NME4 | turquoise |
| ENSG00000103227 | LMF1 | turquoise |
| ENSG00000103245 | NARFL | turquoise |
| ENSG00000103248 | MTHFSD | turquoise |
| ENSG00000103249 | CLCN7 | turquoise |
| ENSG00000103253 | HAGHL | turquoise |
| ENSG00000103260 | METRN | turquoise |
| ENSG00000103269 | RHBDL1 | turquoise |
| ENSG00000103326 | CAPN15 | turquoise |
| ENSG00000103335 | PIEZO1 | turquoise |
| ENSG00000103343 | ZNF174 | turquoise |
| ENSG00000103351 | CLUAP1 | turquoise |
| ENSG00000103353 | UBFD1 | turquoise |
| ENSG00000103365 | GGA2 | turquoise |
| ENSG00000103472 | RRN3P2 | turquoise |
| ENSG00000103479 | RBL2 | turquoise |
| ENSG00000103485 | QPRT | turquoise |
| ENSG00000103489 | XYLT1 | turquoise |
| ENSG00000103490 | PYCARD | turquoise |
| ENSG00000103495 | MAZ | turquoise |
| ENSG00000103496 | STX4 | turquoise |
| ENSG00000103502 | CDIPT | turquoise |
| ENSG00000103507 | BCKDK | turquoise |
| ENSG00000103510 | KAT8 | turquoise |
| ENSG00000103540 | CCP110 | turquoise |
| ENSG00000103549 | RNF40 | turquoise |
| ENSG00000103657 | HERC1 | turquoise |
| ENSG00000103671 | TRIP4 | turquoise |
| ENSG00000103707 | MTFMT | turquoise |
| ENSG00000103723 | AP3B2 | turquoise |
| ENSG00000103742 | IGDCC4 | turquoise |
| ENSG00000103769 | RAB11A | turquoise |
| ENSG00000103811 | CTSH | turquoise |
| ENSG00000103932 | RPAP1 | turquoise |
| ENSG00000103978 | TMEM87A | turquoise |
| ENSG00000103995 | CEP152 | turquoise |
| ENSG00000104043 | ATP8B4 | turquoise |
| ENSG00000104047 | DTWD1 | turquoise |
| ENSG00000104064 | GABPB1 | turquoise |
| ENSG00000104093 | DMXL2 | turquoise |
| ENSG00000104131 | EIF3J | turquoise |

| | | |
|---|---|---|
| ENSG00000104142 | VPS18 | turquoise |
| ENSG00000104147 | OIP5 | turquoise |
| ENSG00000104154 | SLC30A4 | turquoise |
| ENSG00000104164 | BLOC1S6 | turquoise |
| ENSG00000104205 | SGK3 | turquoise |
| ENSG00000104218 | CSPP1 | turquoise |
| ENSG00000104219 | ZDHHC2 | turquoise |
| ENSG00000104228 | TRIM35 | turquoise |
| ENSG00000104231 | ZFAND1 | turquoise |
| ENSG00000104267 | CA2 | turquoise |
| ENSG00000104290 | FZD3 | turquoise |
| ENSG00000104312 | RIPK2 | turquoise |
| ENSG00000104320 | NBN | turquoise |
| ENSG00000104331 | IMPAD1 | turquoise |
| ENSG00000104341 | LAPTM4B | turquoise |
| ENSG00000104343 | UBE2W | turquoise |
| ENSG00000104356 | POP1 | turquoise |
| ENSG00000104368 | PLAT | turquoise |
| ENSG00000104375 | STK3 | turquoise |
| ENSG00000104381 | GDAP1 | turquoise |
| ENSG00000104388 | RAB2A | turquoise |
| ENSG00000104408 | EIF3E | turquoise |
| ENSG00000104412 | EMC2 | turquoise |
| ENSG00000104427 | ZC2HC1A | turquoise |
| ENSG00000104432 | IL7 | turquoise |
| ENSG00000104442 | ARMC1 | turquoise |
| ENSG00000104447 | TRPS1 | turquoise |
| ENSG00000104450 | SPAG1 | turquoise |
| ENSG00000104497 | SNX16 | turquoise |
| ENSG00000104517 | UBR5 | turquoise |
| ENSG00000104518 | GSDMD | turquoise |
| ENSG00000104522 | TSTA3 | turquoise |
| ENSG00000104524 | PYCR3 | turquoise |
| ENSG00000104529 | EEF1D | turquoise |
| ENSG00000104549 | SQLE | turquoise |
| ENSG00000104626 | ERI1 | turquoise |
| ENSG00000104635 | SLC39A14 | turquoise |
| ENSG00000104643 | MTMR9 | turquoise |
| ENSG00000104660 | LEPROTL1 | turquoise |
| ENSG00000104671 | DCTN6 | turquoise |
| ENSG00000104687 | GSR | turquoise |
| ENSG00000104689 | TNFRSF10A | turquoise |
| ENSG00000104691 | UBXN8 | turquoise |
| ENSG00000104695 | PPP2CB | turquoise |
| ENSG00000104722 | NEFM | turquoise |
| ENSG00000104731 | KLHDC4 | turquoise |
| ENSG00000104738 | MCM4 | turquoise |
| ENSG00000104756 | KCTD9 | turquoise |

| | | |
|---|---|---|
| ENSG00000104763 | ASAH1 | turquoise |
| ENSG00000104765 | BNIP3L | turquoise |
| ENSG00000104774 | MAN2B1 | turquoise |
| ENSG00000104783 | KCNN4 | turquoise |
| ENSG00000104812 | GYS1 | turquoise |
| ENSG00000104852 | SNRNP70 | turquoise |
| ENSG00000104853 | CLPTM1 | turquoise |
| ENSG00000104866 | PPP1R37 | turquoise |
| ENSG00000104870 | FCGRT | turquoise |
| ENSG00000104881 | PPP1R13L | turquoise |
| ENSG00000104883 | PEX11G | turquoise |
| ENSG00000104884 | ERCC2 | turquoise |
| ENSG00000104885 | DOT1L | turquoise |
| ENSG00000104894 | CD37 | turquoise |
| ENSG00000104897 | SF3A2 | turquoise |
| ENSG00000104915 | STX10 | turquoise |
| ENSG00000104918 | RETN | turquoise |
| ENSG00000104936 | DMPK | turquoise |
| ENSG00000104946 | TBC1D17 | turquoise |
| ENSG00000104953 | TLE6 | turquoise |
| ENSG00000104957 | CCDC130 | turquoise |
| ENSG00000104960 | PTOV1 | turquoise |
| ENSG00000104969 | SGTA | turquoise |
| ENSG00000104972 | LILRB1 | turquoise |
| ENSG00000104973 | MED25 | turquoise |
| ENSG00000104980 | TIMM44 | turquoise |
| ENSG00000105011 | ASF1B | turquoise |
| ENSG00000105048 | TNNT1 | turquoise |
| ENSG00000105063 | PPP6R1 | turquoise |
| ENSG00000105072 | C19orf44 | turquoise |
| ENSG00000105085 | MED26 | turquoise |
| ENSG00000105122 | RASAL3 | turquoise |
| ENSG00000105135 | ILVBL | turquoise |
| ENSG00000105136 | ZNF419 | turquoise |
| ENSG00000105137 | SYDE1 | turquoise |
| ENSG00000105146 | AURKC | turquoise |
| ENSG00000105176 | URI1 | turquoise |
| ENSG00000105186 | ANKRD27 | turquoise |
| ENSG00000105197 | TIMM50 | turquoise |
| ENSG00000105204 | DYRK1B | turquoise |
| ENSG00000105221 | AKT2 | turquoise |
| ENSG00000105229 | PIAS4 | turquoise |
| ENSG00000105245 | NUMBL | turquoise |
| ENSG00000105255 | FSD1 | turquoise |
| ENSG00000105270 | CLIP3 | turquoise |
| ENSG00000105278 | ZFR2 | turquoise |
| ENSG00000105298 | CACTIN | turquoise |
| ENSG00000105321 | CCDC9 | turquoise |

| | | |
|---|---|---|
| ENSG00000105325 | FZR1 | turquoise |
| ENSG00000105327 | BBC3 | turquoise |
| ENSG00000105329 | TGFB1 | turquoise |
| ENSG00000105341 | DMAC2 | turquoise |
| ENSG00000105352 | CEACAM4 | turquoise |
| ENSG00000105383 | CD33 | turquoise |
| ENSG00000105397 | TYK2 | turquoise |
| ENSG00000105427 | CNFN | turquoise |
| ENSG00000105429 | MEGF8 | turquoise |
| ENSG00000105443 | CYTH2 | turquoise |
| ENSG00000105464 | GRIN2D | turquoise |
| ENSG00000105479 | CCDC114 | turquoise |
| ENSG00000105497 | ZNF175 | turquoise |
| ENSG00000105509 | HAS1 | turquoise |
| ENSG00000105519 | CAPS | turquoise |
| ENSG00000105576 | TNPO2 | turquoise |
| ENSG00000105613 | MAST1 | turquoise |
| ENSG00000105618 | PRPF31 | turquoise |
| ENSG00000105649 | RAB3A | turquoise |
| ENSG00000105662 | CRTC1 | turquoise |
| ENSG00000105700 | KXD1 | turquoise |
| ENSG00000105705 | SUGP1 | turquoise |
| ENSG00000105708 | ZNF14 | turquoise |
| ENSG00000105711 | SCN1B | turquoise |
| ENSG00000105723 | GSK3A | turquoise |
| ENSG00000105726 | ATP13A1 | turquoise |
| ENSG00000105732 | ZNF574 | turquoise |
| ENSG00000105738 | SIPA1L3 | turquoise |
| ENSG00000105767 | CADM4 | turquoise |
| ENSG00000105784 | RUNDC3B | turquoise |
| ENSG00000105793 | GTPBP10 | turquoise |
| ENSG00000105808 | RASA4 | turquoise |
| ENSG00000105810 | CDK6 | turquoise |
| ENSG00000105819 | PMPCB | turquoise |
| ENSG00000105821 | DNAJC2 | turquoise |
| ENSG00000105829 | BET1 | turquoise |
| ENSG00000105835 | NAMPT | turquoise |
| ENSG00000105849 | TWISTNB | turquoise |
| ENSG00000105854 | PON2 | turquoise |
| ENSG00000105856 | HBP1 | turquoise |
| ENSG00000105865 | DUS4L | turquoise |
| ENSG00000105866 | SP4 | turquoise |
| ENSG00000105879 | CBLL1 | turquoise |
| ENSG00000105887 | MTPN | turquoise |
| ENSG00000105963 | ADAP1 | turquoise |
| ENSG00000105967 | TFEC | turquoise |
| ENSG00000105971 | CAV2 | turquoise |
| ENSG00000105974 | CAV1 | turquoise |

| | | |
|---|---|---|
| ENSG00000105983 | LMBR1 | turquoise |
| ENSG00000105991 | HOXA1 | turquoise |
| ENSG00000105993 | DNAJB6 | turquoise |
| ENSG00000106009 | BRAT1 | turquoise |
| ENSG00000106012 | IQCE | turquoise |
| ENSG00000106034 | CPED1 | turquoise |
| ENSG00000106049 | HIBADH | turquoise |
| ENSG00000106052 | TAX1BP1 | turquoise |
| ENSG00000106069 | CHN2 | turquoise |
| ENSG00000106077 | ABHD11 | turquoise |
| ENSG00000106086 | PLEKHA8 | turquoise |
| ENSG00000106089 | STX1A | turquoise |
| ENSG00000106123 | EPHB6 | turquoise |
| ENSG00000106133 | NSUN5P2 | turquoise |
| ENSG00000106144 | CASP2 | turquoise |
| ENSG00000106244 | PDAP1 | turquoise |
| ENSG00000106246 | PTCD1 | turquoise |
| ENSG00000106258 | CYP3A5 | turquoise |
| ENSG00000106261 | ZKSCAN1 | turquoise |
| ENSG00000106290 | TAF6 | turquoise |
| ENSG00000106299 | WASL | turquoise |
| ENSG00000106327 | TFR2 | turquoise |
| ENSG00000106330 | MOSPD3 | turquoise |
| ENSG00000106333 | PCOLCE | turquoise |
| ENSG00000106346 | USP42 | turquoise |
| ENSG00000106351 | AGFG2 | turquoise |
| ENSG00000106355 | LSM5 | turquoise |
| ENSG00000106392 | C1GALT1 | turquoise |
| ENSG00000106399 | RPA3 | turquoise |
| ENSG00000106404 | CLDN15 | turquoise |
| ENSG00000106415 | GLCCI1 | turquoise |
| ENSG00000106443 | PHF14 | turquoise |
| ENSG00000106459 | NRF1 | turquoise |
| ENSG00000106460 | TMEM106B | turquoise |
| ENSG00000106479 | ZNF862 | turquoise |
| ENSG00000106524 | ANKMY2 | turquoise |
| ENSG00000106526 | ACTR3C | turquoise |
| ENSG00000106537 | TSPAN13 | turquoise |
| ENSG00000106546 | AHR | turquoise |
| ENSG00000106560 | GIMAP2 | turquoise |
| ENSG00000106588 | PSMA2 | turquoise |
| ENSG00000106591 | MRPL32 | turquoise |
| ENSG00000106610 | STAG3L4 | turquoise |
| ENSG00000106615 | RHEB | turquoise |
| ENSG00000106617 | PRKAG2 | turquoise |
| ENSG00000106633 | GCK | turquoise |
| ENSG00000106636 | YKT6 | turquoise |
| ENSG00000106665 | CLIP2 | turquoise |

| | | |
|---|---|---|
| ENSG00000106686 | SPATA6L | turquoise |
| ENSG00000106692 | FKTN | turquoise |
| ENSG00000106701 | FSD1L | turquoise |
| ENSG00000106723 | SPIN1 | turquoise |
| ENSG00000106733 | NMRK1 | turquoise |
| ENSG00000106771 | TMEM245 | turquoise |
| ENSG00000106772 | PRUNE2 | turquoise |
| ENSG00000106780 | MEGF9 | turquoise |
| ENSG00000106789 | CORO2A | turquoise |
| ENSG00000106799 | TGFBR1 | turquoise |
| ENSG00000106829 | TLE4 | turquoise |
| ENSG00000106853 | PTGR1 | turquoise |
| ENSG00000106952 | TNFSF8 | turquoise |
| ENSG00000106976 | DNM1 | turquoise |
| ENSG00000106991 | ENG | turquoise |
| ENSG00000106992 | AK1 | turquoise |
| ENSG00000106993 | CDC37L1 | turquoise |
| ENSG00000107014 | RLN2 | turquoise |
| ENSG00000107020 | PLGRKT | turquoise |
| ENSG00000107021 | TBC1D13 | turquoise |
| ENSG00000107036 | RIC1 | turquoise |
| ENSG00000107077 | KDM4C | turquoise |
| ENSG00000107099 | DOCK8 | turquoise |
| ENSG00000107104 | KANK1 | turquoise |
| ENSG00000107140 | TESK1 | turquoise |
| ENSG00000107164 | FUBP3 | turquoise |
| ENSG00000107185 | RGP1 | turquoise |
| ENSG00000107242 | PIP5K1B | turquoise |
| ENSG00000107281 | NPDC1 | turquoise |
| ENSG00000107290 | SETX | turquoise |
| ENSG00000107317 | PTGDS | turquoise |
| ENSG00000107362 | ABHD17B | turquoise |
| ENSG00000107371 | EXOSC3 | turquoise |
| ENSG00000107372 | ZFAND5 | turquoise |
| ENSG00000107404 | DVL1 | turquoise |
| ENSG00000107443 | CCNJ | turquoise |
| ENSG00000107485 | GATA3 | turquoise |
| ENSG00000107521 | HPS1 | turquoise |
| ENSG00000107560 | RAB11FIP2 | turquoise |
| ENSG00000107566 | ERLIN1 | turquoise |
| ENSG00000107581 | EIF3A | turquoise |
| ENSG00000107593 | PKD2L1 | turquoise |
| ENSG00000107614 | TRDMT1 | turquoise |
| ENSG00000107625 | DDX50 | turquoise |
| ENSG00000107643 | MAPK8 | turquoise |
| ENSG00000107651 | SEC23IP | turquoise |
| ENSG00000107669 | ATE1 | turquoise |
| ENSG00000107672 | NSMCE4A | turquoise |

| | | |
|---|---|---|
| ENSG00000107679 | PLEKHA1 | turquoise |
| ENSG00000107731 | UNC5B | turquoise |
| ENSG00000107736 | CDH23 | turquoise |
| ENSG00000107758 | PPP3CB | turquoise |
| ENSG00000107771 | CCSER2 | turquoise |
| ENSG00000107779 | BMPR1A | turquoise |
| ENSG00000107789 | MINPP1 | turquoise |
| ENSG00000107798 | LIPA | turquoise |
| ENSG00000107819 | SFXN3 | turquoise |
| ENSG00000107854 | TNKS2 | turquoise |
| ENSG00000107863 | ARHGAP21 | turquoise |
| ENSG00000107890 | ANKRD26 | turquoise |
| ENSG00000107897 | ACBD5 | turquoise |
| ENSG00000107929 | LARP4B | turquoise |
| ENSG00000107937 | GTPBP4 | turquoise |
| ENSG00000107938 | EDRF1 | turquoise |
| ENSG00000107949 | BCCIP | turquoise |
| ENSG00000107951 | MTPAP | turquoise |
| ENSG00000107954 | NEURL1 | turquoise |
| ENSG00000107957 | SH3PXD2A | turquoise |
| ENSG00000107968 | MAP3K8 | turquoise |
| ENSG00000108010 | GLRX3 | turquoise |
| ENSG00000108021 | FAM208B | turquoise |
| ENSG00000108055 | SMC3 | turquoise |
| ENSG00000108061 | SHOC2 | turquoise |
| ENSG00000108064 | TFAM | turquoise |
| ENSG00000108091 | CCDC6 | turquoise |
| ENSG00000108094 | CUL2 | turquoise |
| ENSG00000108175 | ZMIZ1 | turquoise |
| ENSG00000108239 | TBC1D12 | turquoise |
| ENSG00000108256 | NUFIP2 | turquoise |
| ENSG00000108262 | GIT1 | turquoise |
| ENSG00000108375 | RNF43 | turquoise |
| ENSG00000108384 | RAD51C | turquoise |
| ENSG00000108395 | TRIM37 | turquoise |
| ENSG00000108406 | DHX40 | turquoise |
| ENSG00000108439 | PNPO | turquoise |
| ENSG00000108443 | RPS6KB1 | turquoise |
| ENSG00000108465 | CDK5RAP3 | turquoise |
| ENSG00000108468 | CBX1 | turquoise |
| ENSG00000108469 | RECQL5 | turquoise |
| ENSG00000108479 | GALK1 | turquoise |
| ENSG00000108506 | INTS2 | turquoise |
| ENSG00000108510 | MED13 | turquoise |
| ENSG00000108515 | ENO3 | turquoise |
| ENSG00000108523 | RNF167 | turquoise |
| ENSG00000108528 | SLC25A11 | turquoise |
| ENSG00000108551 | RASD1 | turquoise |

| | | |
|---|---|---|
| ENSG00000108556 | CHRNE | turquoise |
| ENSG00000108559 | NUP88 | turquoise |
| ENSG00000108582 | CPD | turquoise |
| ENSG00000108587 | GOSR1 | turquoise |
| ENSG00000108588 | CCDC47 | turquoise |
| ENSG00000108590 | MED31 | turquoise |
| ENSG00000108591 | DRG2 | turquoise |
| ENSG00000108599 | AKAP10 | turquoise |
| ENSG00000108622 | ICAM2 | turquoise |
| ENSG00000108641 | B9D1 | turquoise |
| ENSG00000108651 | UTP6 | turquoise |
| ENSG00000108654 | DDX5 | turquoise |
| ENSG00000108666 | C17orf75 | turquoise |
| ENSG00000108669 | CYTH1 | turquoise |
| ENSG00000108733 | PEX12 | turquoise |
| ENSG00000108773 | KAT2A | turquoise |
| ENSG00000108784 | NAGLU | turquoise |
| ENSG00000108788 | MLX | turquoise |
| ENSG00000108798 | ABI3 | turquoise |
| ENSG00000108799 | EZH1 | turquoise |
| ENSG00000108813 | DLX4 | turquoise |
| ENSG00000108819 | PPP1R9B | turquoise |
| ENSG00000108823 | SGCA | turquoise |
| ENSG00000108830 | RND2 | turquoise |
| ENSG00000108839 | ALOX12 | turquoise |
| ENSG00000108840 | HDAC5 | turquoise |
| ENSG00000108854 | SMURF2 | turquoise |
| ENSG00000108932 | SLC16A6 | turquoise |
| ENSG00000108946 | PRKAR1A | turquoise |
| ENSG00000108960 | MMD | turquoise |
| ENSG00000108961 | RANGRF | turquoise |
| ENSG00000108963 | DPH1 | turquoise |
| ENSG00000109016 | DHRS7B | turquoise |
| ENSG00000109046 | WSB1 | turquoise |
| ENSG00000109062 | SLC9A3R1 | turquoise |
| ENSG00000109065 | NAT9 | turquoise |
| ENSG00000109066 | TMEM104 | turquoise |
| ENSG00000109072 | VTN | turquoise |
| ENSG00000109083 | IFT20 | turquoise |
| ENSG00000109133 | TMEM33 | turquoise |
| ENSG00000109171 | SLAIN2 | turquoise |
| ENSG00000109180 | OCIAD1 | turquoise |
| ENSG00000109184 | DCUN1D4 | turquoise |
| ENSG00000109189 | USP46 | turquoise |
| ENSG00000109265 | KIAA1211 | turquoise |
| ENSG00000109270 | LAMTOR3 | turquoise |
| ENSG00000109272 | PF4V1 | turquoise |
| ENSG00000109320 | NFKB1 | turquoise |

| | | |
|---|---|---|
| ENSG00000109323 | MANBA | turquoise |
| ENSG00000109332 | UBE2D3 | turquoise |
| ENSG00000109339 | MAPK10 | turquoise |
| ENSG00000109381 | ELF2 | turquoise |
| ENSG00000109436 | TBC1D9 | turquoise |
| ENSG00000109445 | ZNF330 | turquoise |
| ENSG00000109452 | INPP4B | turquoise |
| ENSG00000109466 | KLHL2 | turquoise |
| ENSG00000109475 | RPL34 | turquoise |
| ENSG00000109536 | FRG1 | turquoise |
| ENSG00000109572 | CLCN3 | turquoise |
| ENSG00000109586 | GALNT7 | turquoise |
| ENSG00000109606 | DHX15 | turquoise |
| ENSG00000109618 | SEPSECS | turquoise |
| ENSG00000109654 | TRIM2 | turquoise |
| ENSG00000109667 | SLC2A9 | turquoise |
| ENSG00000109670 | FBXW7 | turquoise |
| ENSG00000109674 | NEIL3 | turquoise |
| ENSG00000109680 | TBC1D19 | turquoise |
| ENSG00000109685 | NSD2 | turquoise |
| ENSG00000109689 | STIM2 | turquoise |
| ENSG00000109736 | MFSD10 | turquoise |
| ENSG00000109756 | RAPGEF2 | turquoise |
| ENSG00000109762 | SNX25 | turquoise |
| ENSG00000109787 | KLF3 | turquoise |
| ENSG00000109790 | KLHL5 | turquoise |
| ENSG00000109805 | NCAPG | turquoise |
| ENSG00000109814 | UGDH | turquoise |
| ENSG00000109911 | ELP4 | turquoise |
| ENSG00000109920 | FNBP4 | turquoise |
| ENSG00000109929 | SC5D | turquoise |
| ENSG00000109944 | C11orf63 | turquoise |
| ENSG00000109971 | HSPA8 | turquoise |
| ENSG00000110002 | VWA5A | turquoise |
| ENSG00000110013 | SIAE | turquoise |
| ENSG00000110047 | EHD1 | turquoise |
| ENSG00000110048 | OSBP | turquoise |
| ENSG00000110060 | PUS3 | turquoise |
| ENSG00000110066 | KMT5B | turquoise |
| ENSG00000110074 | FOXRED1 | turquoise |
| ENSG00000110075 | PPP6R3 | turquoise |
| ENSG00000110076 | NRXN2 | turquoise |
| ENSG00000110090 | CPT1A | turquoise |
| ENSG00000110092 | CCND1 | turquoise |
| ENSG00000110104 | CCDC86 | turquoise |
| ENSG00000110169 | HPX | turquoise |
| ENSG00000110171 | TRIM3 | turquoise |
| ENSG00000110172 | CHORDC1 | turquoise |

| | | |
|---|---|---|
| ENSG00000110200 | ANAPC15 | turquoise |
| ENSG00000110218 | PANX1 | turquoise |
| ENSG00000110274 | CEP164 | turquoise |
| ENSG00000110315 | RNF141 | turquoise |
| ENSG00000110318 | CEP126 | turquoise |
| ENSG00000110321 | EIF4G2 | turquoise |
| ENSG00000110330 | BIRC2 | turquoise |
| ENSG00000110367 | DDX6 | turquoise |
| ENSG00000110395 | CBL | turquoise |
| ENSG00000110422 | HIPK3 | turquoise |
| ENSG00000110429 | FBXO3 | turquoise |
| ENSG00000110435 | PDHX | turquoise |
| ENSG00000110619 | CARS | turquoise |
| ENSG00000110628 | SLC22A18 | turquoise |
| ENSG00000110696 | C11orf58 | turquoise |
| ENSG00000110697 | PITPNM1 | turquoise |
| ENSG00000110721 | CHKA | turquoise |
| ENSG00000110723 | EXPH5 | turquoise |
| ENSG00000110756 | HPS5 | turquoise |
| ENSG00000110768 | GTF2H1 | turquoise |
| ENSG00000110801 | PSMD9 | turquoise |
| ENSG00000110811 | P3H3 | turquoise |
| ENSG00000110841 | PPFIBP1 | turquoise |
| ENSG00000110848 | CD69 | turquoise |
| ENSG00000110851 | PRDM4 | turquoise |
| ENSG00000110852 | CLEC2B | turquoise |
| ENSG00000110871 | COQ5 | turquoise |
| ENSG00000110880 | CORO1C | turquoise |
| ENSG00000110906 | KCTD10 | turquoise |
| ENSG00000110911 | SLC11A2 | turquoise |
| ENSG00000110921 | MVK | turquoise |
| ENSG00000110925 | CSRNP2 | turquoise |
| ENSG00000110958 | PTGES3 | turquoise |
| ENSG00000111011 | RSRC2 | turquoise |
| ENSG00000111087 | GLI1 | turquoise |
| ENSG00000111110 | PPM1H | turquoise |
| ENSG00000111142 | METAP2 | turquoise |
| ENSG00000111145 | ELK3 | turquoise |
| ENSG00000111186 | WNT5B | turquoise |
| ENSG00000111199 | TRPV4 | turquoise |
| ENSG00000111203 | ITFG2 | turquoise |
| ENSG00000111224 | PARP11 | turquoise |
| ENSG00000111231 | GPN3 | turquoise |
| ENSG00000111237 | VPS29 | turquoise |
| ENSG00000111247 | RAD51AP1 | turquoise |
| ENSG00000111252 | SH2B3 | turquoise |
| ENSG00000111254 | AKAP3 | turquoise |
| ENSG00000111269 | CREBL2 | turquoise |

| | | |
|---|---|---|
| ENSG00000111271 | ACAD10 | turquoise |
| ENSG00000111275 | ALDH2 | turquoise |
| ENSG00000111276 | CDKN1B | turquoise |
| ENSG00000111300 | NAA25 | turquoise |
| ENSG00000111319 | SCNN1A | turquoise |
| ENSG00000111328 | CDK2AP1 | turquoise |
| ENSG00000111341 | MGP | turquoise |
| ENSG00000111348 | ARHGDIB | turquoise |
| ENSG00000111358 | GTF2H3 | turquoise |
| ENSG00000111371 | SLC38A1 | turquoise |
| ENSG00000111412 | C12orf49 | turquoise |
| ENSG00000111424 | VDR | turquoise |
| ENSG00000111445 | RFC5 | turquoise |
| ENSG00000111450 | STX2 | turquoise |
| ENSG00000111452 | ADGRD1 | turquoise |
| ENSG00000111530 | CAND1 | turquoise |
| ENSG00000111554 | MDM1 | turquoise |
| ENSG00000111581 | NUP107 | turquoise |
| ENSG00000111596 | CNOT2 | turquoise |
| ENSG00000111602 | TIMELESS | turquoise |
| ENSG00000111605 | CPSF6 | turquoise |
| ENSG00000111615 | KRR1 | turquoise |
| ENSG00000111641 | NOP2 | turquoise |
| ENSG00000111644 | ACRBP | turquoise |
| ENSG00000111647 | UHRF1BP1L | turquoise |
| ENSG00000111652 | COPS7A | turquoise |
| ENSG00000111664 | GNB3 | turquoise |
| ENSG00000111670 | GNPTAB | turquoise |
| ENSG00000111671 | SPSB2 | turquoise |
| ENSG00000111676 | ATN1 | turquoise |
| ENSG00000111684 | LPCAT3 | turquoise |
| ENSG00000111696 | NT5DC3 | turquoise |
| ENSG00000111707 | SUDS3 | turquoise |
| ENSG00000111711 | GOLT1B | turquoise |
| ENSG00000111716 | LDHB | turquoise |
| ENSG00000111725 | PRKAB1 | turquoise |
| ENSG00000111726 | CMAS | turquoise |
| ENSG00000111727 | HCFC2 | turquoise |
| ENSG00000111729 | CLEC4A | turquoise |
| ENSG00000111731 | C2CD5 | turquoise |
| ENSG00000111790 | FGFR1OP2 | turquoise |
| ENSG00000111796 | KLRB1 | turquoise |
| ENSG00000111802 | TDP2 | turquoise |
| ENSG00000111817 | DSE | turquoise |
| ENSG00000111832 | RWDD1 | turquoise |
| ENSG00000111843 | TMEM14C | turquoise |
| ENSG00000111845 | PAK1IP1 | turquoise |
| ENSG00000111859 | NEDD9 | turquoise |

| | | |
|---|---|---|
| ENSG00000111860 | CEP85L | turquoise |
| ENSG00000111875 | ASF1A | turquoise |
| ENSG00000111877 | MCM9 | turquoise |
| ENSG00000111879 | FAM184A | turquoise |
| ENSG00000111880 | RNGTT | turquoise |
| ENSG00000111897 | SERINC1 | turquoise |
| ENSG00000111906 | HDDC2 | turquoise |
| ENSG00000111911 | HINT3 | turquoise |
| ENSG00000111912 | NCOA7 | turquoise |
| ENSG00000111961 | SASH1 | turquoise |
| ENSG00000111962 | UST | turquoise |
| ENSG00000112029 | FBXO5 | turquoise |
| ENSG00000112031 | MTRF1L | turquoise |
| ENSG00000112033 | PPARD | turquoise |
| ENSG00000112039 | FANCE | turquoise |
| ENSG00000112062 | MAPK14 | turquoise |
| ENSG00000112078 | KCTD20 | turquoise |
| ENSG00000112079 | STK38 | turquoise |
| ENSG00000112081 | SRSF3 | turquoise |
| ENSG00000112144 | ICK | turquoise |
| ENSG00000112146 | FBXO9 | turquoise |
| ENSG00000112149 | CD83 | turquoise |
| ENSG00000112159 | MDN1 | turquoise |
| ENSG00000112182 | BACH2 | turquoise |
| ENSG00000112200 | ZNF451 | turquoise |
| ENSG00000112208 | BAG2 | turquoise |
| ENSG00000112210 | RAB23 | turquoise |
| ENSG00000112218 | GPR63 | turquoise |
| ENSG00000112234 | FBXL4 | turquoise |
| ENSG00000112237 | CCNC | turquoise |
| ENSG00000112242 | E2F3 | turquoise |
| ENSG00000112245 | PTP4A1 | turquoise |
| ENSG00000112249 | ASCC3 | turquoise |
| ENSG00000112282 | MED23 | turquoise |
| ENSG00000112290 | WASF1 | turquoise |
| ENSG00000112294 | ALDH5A1 | turquoise |
| ENSG00000112303 | VNN2 | turquoise |
| ENSG00000112304 | ACOT13 | turquoise |
| ENSG00000112305 | SMAP1 | turquoise |
| ENSG00000112308 | C6orf62 | turquoise |
| ENSG00000112312 | GMNN | turquoise |
| ENSG00000112339 | HBS1L | turquoise |
| ENSG00000112357 | PEX7 | turquoise |
| ENSG00000112365 | ZBTB24 | turquoise |
| ENSG00000112378 | PERP | turquoise |
| ENSG00000112394 | SLC16A10 | turquoise |
| ENSG00000112406 | HECA | turquoise |
| ENSG00000112414 | ADGRG6 | turquoise |

| | | |
|---|---|---|
| ENSG00000112419 | PHACTR2 | turquoise |
| ENSG00000112425 | EPM2A | turquoise |
| ENSG00000112486 | CCR6 | turquoise |
| ENSG00000112531 | QKI | turquoise |
| ENSG00000112578 | BYSL | turquoise |
| ENSG00000112592 | TBP | turquoise |
| ENSG00000112599 | GUCA1B | turquoise |
| ENSG00000112619 | PRPH2 | turquoise |
| ENSG00000112624 | BICRAL | turquoise |
| ENSG00000112658 | SRF | turquoise |
| ENSG00000112659 | CUL9 | turquoise |
| ENSG00000112685 | EXOC2 | turquoise |
| ENSG00000112697 | TMEM30A | turquoise |
| ENSG00000112701 | SENP6 | turquoise |
| ENSG00000112739 | PRPF4B | turquoise |
| ENSG00000112763 | BTN2A1 | turquoise |
| ENSG00000112782 | CLIC5 | turquoise |
| ENSG00000112787 | FBRSL1 | turquoise |
| ENSG00000112851 | ERBIN | turquoise |
| ENSG00000112874 | NUDT12 | turquoise |
| ENSG00000112877 | CEP72 | turquoise |
| ENSG00000112893 | MAN2A1 | turquoise |
| ENSG00000112902 | SEMA5A | turquoise |
| ENSG00000112941 | PAPD7 | turquoise |
| ENSG00000112972 | HMGCS1 | turquoise |
| ENSG00000112977 | DAP | turquoise |
| ENSG00000112983 | BRD8 | turquoise |
| ENSG00000112984 | KIF20A | turquoise |
| ENSG00000112992 | NNT | turquoise |
| ENSG00000112996 | MRPS30 | turquoise |
| ENSG00000113013 | HSPA9 | turquoise |
| ENSG00000113070 | HBEGF | turquoise |
| ENSG00000113083 | LOX | turquoise |
| ENSG00000113108 | APBB3 | turquoise |
| ENSG00000113161 | HMGCR | turquoise |
| ENSG00000113163 | COL4A3BP | turquoise |
| ENSG00000113194 | FAF2 | turquoise |
| ENSG00000113231 | PDE8B | turquoise |
| ENSG00000113240 | CLK4 | turquoise |
| ENSG00000113263 | ITK | turquoise |
| ENSG00000113282 | CLINT1 | turquoise |
| ENSG00000113300 | CNOT6 | turquoise |
| ENSG00000113303 | BTNL8 | turquoise |
| ENSG00000113318 | MSH3 | turquoise |
| ENSG00000113319 | RASGRF2 | turquoise |
| ENSG00000113328 | CCNG1 | turquoise |
| ENSG00000113356 | POLR3G | turquoise |
| ENSG00000113360 | DROSHA | turquoise |

| | | |
|---|---|---|
| ENSG00000113369 | ARRDC3 | turquoise |
| ENSG00000113384 | GOLPH3 | turquoise |
| ENSG00000113387 | SUB1 | turquoise |
| ENSG00000113391 | FAM172A | turquoise |
| ENSG00000113407 | TARS | turquoise |
| ENSG00000113441 | LNPEP | turquoise |
| ENSG00000113448 | PDE4D | turquoise |
| ENSG00000113456 | RAD1 | turquoise |
| ENSG00000113460 | BRIX1 | turquoise |
| ENSG00000113522 | RAD50 | turquoise |
| ENSG00000113532 | ST8SIA4 | turquoise |
| ENSG00000113555 | PCDH12 | turquoise |
| ENSG00000113569 | NUP155 | turquoise |
| ENSG00000113575 | PPP2CA | turquoise |
| ENSG00000113580 | NR3C1 | turquoise |
| ENSG00000113583 | C5orf15 | turquoise |
| ENSG00000113593 | PPWD1 | turquoise |
| ENSG00000113595 | TRIM23 | turquoise |
| ENSG00000113597 | TRAPPC13 | turquoise |
| ENSG00000113615 | SEC24A | turquoise |
| ENSG00000113621 | TXNDC15 | turquoise |
| ENSG00000113638 | TTC33 | turquoise |
| ENSG00000113643 | RARS | turquoise |
| ENSG00000113648 | H2AFY | turquoise |
| ENSG00000113649 | TCERG1 | turquoise |
| ENSG00000113657 | DPYSL3 | turquoise |
| ENSG00000113658 | SMAD5 | turquoise |
| ENSG00000113712 | CSNK1A1 | turquoise |
| ENSG00000113716 | HMGXB3 | turquoise |
| ENSG00000113742 | CPEB4 | turquoise |
| ENSG00000113758 | DBN1 | turquoise |
| ENSG00000113761 | ZNF346 | turquoise |
| ENSG00000113810 | SMC4 | turquoise |
| ENSG00000113811 | SELENOK | turquoise |
| ENSG00000113838 | TBCCD1 | turquoise |
| ENSG00000113851 | CRBN | turquoise |
| ENSG00000113916 | BCL6 | turquoise |
| ENSG00000113971 | NPHP3 | turquoise |
| ENSG00000114026 | OGG1 | turquoise |
| ENSG00000114054 | PCCB | turquoise |
| ENSG00000114062 | UBE3A | turquoise |
| ENSG00000114098 | ARMC8 | turquoise |
| ENSG00000114107 | CEP70 | turquoise |
| ENSG00000114120 | SLC25A36 | turquoise |
| ENSG00000114125 | RNF7 | turquoise |
| ENSG00000114126 | TFDP2 | turquoise |
| ENSG00000114127 | XRN1 | turquoise |
| ENSG00000114166 | KAT2B | turquoise |

| | | |
|---|---|---|
| ENSG00000114204 | SERPINI2 | turquoise |
| ENSG00000114209 | PDCD10 | turquoise |
| ENSG00000114302 | PRKAR2A | turquoise |
| ENSG00000114315 | HES1 | turquoise |
| ENSG00000114331 | ACAP2 | turquoise |
| ENSG00000114346 | ECT2 | turquoise |
| ENSG00000114354 | TFG | turquoise |
| ENSG00000114383 | TUSC2 | turquoise |
| ENSG00000114388 | NPRL2 | turquoise |
| ENSG00000114416 | FXR1 | turquoise |
| ENSG00000114423 | CBLB | turquoise |
| ENSG00000114439 | BBX | turquoise |
| ENSG00000114446 | IFT57 | turquoise |
| ENSG00000114450 | GNB4 | turquoise |
| ENSG00000114455 | HHLA2 | turquoise |
| ENSG00000114473 | IQCG | turquoise |
| ENSG00000114480 | GBE1 | turquoise |
| ENSG00000114503 | NCBP2 | turquoise |
| ENSG00000114520 | SNX4 | turquoise |
| ENSG00000114541 | FRMD4B | turquoise |
| ENSG00000114573 | ATP6V1A | turquoise |
| ENSG00000114626 | ABTB1 | turquoise |
| ENSG00000114631 | PODXL2 | turquoise |
| ENSG00000114648 | KLHL18 | turquoise |
| ENSG00000114650 | SCAP | turquoise |
| ENSG00000114686 | MRPL3 | turquoise |
| ENSG00000114735 | HEMK1 | turquoise |
| ENSG00000114737 | CISH | turquoise |
| ENSG00000114738 | MAPKAPK3 | turquoise |
| ENSG00000114739 | ACVR2B | turquoise |
| ENSG00000114742 | WDR48 | turquoise |
| ENSG00000114744 | COMMD2 | turquoise |
| ENSG00000114796 | KLHL24 | turquoise |
| ENSG00000114805 | PLCH1 | turquoise |
| ENSG00000114850 | SSR3 | turquoise |
| ENSG00000114853 | ZBTB47 | turquoise |
| ENSG00000114859 | CLCN2 | turquoise |
| ENSG00000114904 | NEK4 | turquoise |
| ENSG00000114923 | SLC4A3 | turquoise |
| ENSG00000114933 | INO80D | turquoise |
| ENSG00000114948 | ADAM23 | turquoise |
| ENSG00000114978 | MOB1A | turquoise |
| ENSG00000114988 | LMAN2L | turquoise |
| ENSG00000114999 | TTL | turquoise |
| ENSG00000115020 | PIKFYVE | turquoise |
| ENSG00000115042 | FAHD2A | turquoise |
| ENSG00000115073 | ACTR1B | turquoise |
| ENSG00000115085 | ZAP70 | turquoise |

| | | |
|---|---|---|
| ENSG00000115091 | ACTR3 | turquoise |
| ENSG00000115107 | STEAP3 | turquoise |
| ENSG00000115109 | EPB41L5 | turquoise |
| ENSG00000115112 | TFCP2L1 | turquoise |
| ENSG00000115128 | SF3B6 | turquoise |
| ENSG00000115129 | TP53I3 | turquoise |
| ENSG00000115137 | DNAJC27 | turquoise |
| ENSG00000115138 | POMC | turquoise |
| ENSG00000115159 | GPD2 | turquoise |
| ENSG00000115165 | CYTIP | turquoise |
| ENSG00000115170 | ACVR1 | turquoise |
| ENSG00000115204 | MPV17 | turquoise |
| ENSG00000115207 | GTF3C2 | turquoise |
| ENSG00000115211 | EIF2B4 | turquoise |
| ENSG00000115216 | NRBP1 | turquoise |
| ENSG00000115232 | ITGA4 | turquoise |
| ENSG00000115233 | PSMD14 | turquoise |
| ENSG00000115255 | REEP6 | turquoise |
| ENSG00000115266 | APC2 | turquoise |
| ENSG00000115271 | GCA | turquoise |
| ENSG00000115275 | MOGS | turquoise |
| ENSG00000115282 | TTC31 | turquoise |
| ENSG00000115295 | CLIP4 | turquoise |
| ENSG00000115306 | SPTBN1 | turquoise |
| ENSG00000115307 | AUP1 | turquoise |
| ENSG00000115310 | RTN4 | turquoise |
| ENSG00000115317 | HTRA2 | turquoise |
| ENSG00000115325 | DOK1 | turquoise |
| ENSG00000115339 | GALNT3 | turquoise |
| ENSG00000115355 | CCDC88A | turquoise |
| ENSG00000115364 | MRPL19 | turquoise |
| ENSG00000115365 | LANCL1 | turquoise |
| ENSG00000115368 | WDR75 | turquoise |
| ENSG00000115392 | FANCL | turquoise |
| ENSG00000115419 | GLS | turquoise |
| ENSG00000115421 | PAPOLG | turquoise |
| ENSG00000115425 | PECR | turquoise |
| ENSG00000115446 | UNC50 | turquoise |
| ENSG00000115457 | IGFBP2 | turquoise |
| ENSG00000115459 | ELMOD3 | turquoise |
| ENSG00000115486 | GGCX | turquoise |
| ENSG00000115504 | EHBP1 | turquoise |
| ENSG00000115514 | TXNDC9 | turquoise |
| ENSG00000115520 | COQ10B | turquoise |
| ENSG00000115524 | SF3B1 | turquoise |
| ENSG00000115525 | ST3GAL5 | turquoise |
| ENSG00000115540 | MOB4 | turquoise |
| ENSG00000115541 | HSPE1 | turquoise |

| | | |
|---|---|---|
| ENSG00000115548 | KDM3A | turquoise |
| ENSG00000115556 | PLCD4 | turquoise |
| ENSG00000115561 | CHMP3 | turquoise |
| ENSG00000115568 | ZNF142 | turquoise |
| ENSG00000115594 | IL1R1 | turquoise |
| ENSG00000115596 | WNT6 | turquoise |
| ENSG00000115602 | IL1RL1 | turquoise |
| ENSG00000115604 | IL18R1 | turquoise |
| ENSG00000115607 | IL18RAP | turquoise |
| ENSG00000115661 | STK16 | turquoise |
| ENSG00000115677 | HDLBP | turquoise |
| ENSG00000115694 | STK25 | turquoise |
| ENSG00000115718 | PROC | turquoise |
| ENSG00000115738 | ID2 | turquoise |
| ENSG00000115750 | TAF1B | turquoise |
| ENSG00000115756 | HPCAL1 | turquoise |
| ENSG00000115760 | BIRC6 | turquoise |
| ENSG00000115762 | PLEKHB2 | turquoise |
| ENSG00000115808 | STRN | turquoise |
| ENSG00000115816 | CEBPZ | turquoise |
| ENSG00000115825 | PRKD3 | turquoise |
| ENSG00000115827 | DCAF17 | turquoise |
| ENSG00000115839 | RAB3GAP1 | turquoise |
| ENSG00000115840 | SLC25A12 | turquoise |
| ENSG00000115841 | RMDN2 | turquoise |
| ENSG00000115866 | DARS | turquoise |
| ENSG00000115875 | SRSF7 | turquoise |
| ENSG00000115896 | PLCL1 | turquoise |
| ENSG00000115904 | SOS1 | turquoise |
| ENSG00000115935 | WIPF1 | turquoise |
| ENSG00000115942 | ORC2 | turquoise |
| ENSG00000115944 | COX7A2L | turquoise |
| ENSG00000115947 | ORC4 | turquoise |
| ENSG00000115966 | ATF2 | turquoise |
| ENSG00000115970 | THADA | turquoise |
| ENSG00000115977 | AAK1 | turquoise |
| ENSG00000115993 | TRAK2 | turquoise |
| ENSG00000116001 | TIA1 | turquoise |
| ENSG00000116005 | PCYOX1 | turquoise |
| ENSG00000116014 | KISS1R | turquoise |
| ENSG00000116016 | EPAS1 | turquoise |
| ENSG00000116030 | SUMO1 | turquoise |
| ENSG00000116044 | NFE2L2 | turquoise |
| ENSG00000116062 | MSH6 | turquoise |
| ENSG00000116095 | PLEKHA3 | turquoise |
| ENSG00000116096 | SPR | turquoise |
| ENSG00000116106 | EPHA4 | turquoise |
| ENSG00000116127 | ALMS1 | turquoise |

| | | |
|---|---|---|
| ENSG00000116138 | DNAJC16 | turquoise |
| ENSG00000116151 | MORN1 | turquoise |
| ENSG00000116161 | CACYBP | turquoise |
| ENSG00000116171 | SCP2 | turquoise |
| ENSG00000116191 | RALGPS2 | turquoise |
| ENSG00000116198 | CEP104 | turquoise |
| ENSG00000116199 | FAM20B | turquoise |
| ENSG00000116209 | TMEM59 | turquoise |
| ENSG00000116213 | WRAP73 | turquoise |
| ENSG00000116266 | STXBP3 | turquoise |
| ENSG00000116273 | PHF13 | turquoise |
| ENSG00000116285 | ERRFI1 | turquoise |
| ENSG00000116299 | KIAA1324 | turquoise |
| ENSG00000116406 | EDEM3 | turquoise |
| ENSG00000116459 | ATP5F1 | turquoise |
| ENSG00000116473 | RAP1A | turquoise |
| ENSG00000116489 | CAPZA1 | turquoise |
| ENSG00000116497 | S100PBP | turquoise |
| ENSG00000116539 | ASH1L | turquoise |
| ENSG00000116544 | DLGAP3 | turquoise |
| ENSG00000116560 | SFPQ | turquoise |
| ENSG00000116574 | RHOU | turquoise |
| ENSG00000116641 | DOCK7 | turquoise |
| ENSG00000116668 | SWT1 | turquoise |
| ENSG00000116675 | DNAJC6 | turquoise |
| ENSG00000116678 | LEPR | turquoise |
| ENSG00000116679 | IVNS1ABP | turquoise |
| ENSG00000116685 | KIAA2013 | turquoise |
| ENSG00000116688 | MFN2 | turquoise |
| ENSG00000116691 | MIIP | turquoise |
| ENSG00000116704 | SLC35D1 | turquoise |
| ENSG00000116711 | PLA2G4A | turquoise |
| ENSG00000116729 | WLS | turquoise |
| ENSG00000116741 | RGS2 | turquoise |
| ENSG00000116747 | TROVE2 | turquoise |
| ENSG00000116750 | UCHL5 | turquoise |
| ENSG00000116752 | BCAS2 | turquoise |
| ENSG00000116754 | SRSF11 | turquoise |
| ENSG00000116786 | PLEKHM2 | turquoise |
| ENSG00000116791 | CRYZ | turquoise |
| ENSG00000116793 | PHTF1 | turquoise |
| ENSG00000116809 | ZBTB17 | turquoise |
| ENSG00000116815 | CD58 | turquoise |
| ENSG00000116830 | TTF2 | turquoise |
| ENSG00000116874 | WARS2 | turquoise |
| ENSG00000116885 | OSCP1 | turquoise |
| ENSG00000116898 | MRPS15 | turquoise |
| ENSG00000116903 | EXOC8 | turquoise |

| | | |
|---|---|---|
| ENSG00000116906 | GNPAT | turquoise |
| ENSG00000116918 | TSNAX | turquoise |
| ENSG00000116922 | C1orf109 | turquoise |
| ENSG00000116954 | RRAGC | turquoise |
| ENSG00000116957 | TBCE | turquoise |
| ENSG00000116962 | NID1 | turquoise |
| ENSG00000116977 | LGALS8 | turquoise |
| ENSG00000116984 | MTR | turquoise |
| ENSG00000116985 | BMP8B | turquoise |
| ENSG00000116990 | MYCL | turquoise |
| ENSG00000116991 | SIPA1L2 | turquoise |
| ENSG00000117000 | RLF | turquoise |
| ENSG00000117009 | KMO | turquoise |
| ENSG00000117010 | ZNF684 | turquoise |
| ENSG00000117013 | KCNQ4 | turquoise |
| ENSG00000117016 | RIMS3 | turquoise |
| ENSG00000117020 | AKT3 | turquoise |
| ENSG00000117036 | ETV3 | turquoise |
| ENSG00000117054 | ACADM | turquoise |
| ENSG00000117091 | CD48 | turquoise |
| ENSG00000117133 | RPF1 | turquoise |
| ENSG00000117143 | UAP1 | turquoise |
| ENSG00000117151 | CTBS | turquoise |
| ENSG00000117153 | KLHL12 | turquoise |
| ENSG00000117155 | SSX2IP | turquoise |
| ENSG00000117174 | ZNHIT6 | turquoise |
| ENSG00000117215 | PLA2G2D | turquoise |
| ENSG00000117222 | RBBP5 | turquoise |
| ENSG00000117226 | GBP3 | turquoise |
| ENSG00000117245 | KIF17 | turquoise |
| ENSG00000117262 | GPR89A | turquoise |
| ENSG00000117266 | CDK18 | turquoise |
| ENSG00000117280 | RAB29 | turquoise |
| ENSG00000117308 | GALE | turquoise |
| ENSG00000117322 | CR2 | turquoise |
| ENSG00000117335 | CD46 | turquoise |
| ENSG00000117360 | PRPF3 | turquoise |
| ENSG00000117385 | P3H1 | turquoise |
| ENSG00000117410 | ATP6V0B | turquoise |
| ENSG00000117411 | B4GALT2 | turquoise |
| ENSG00000117419 | ERI3 | turquoise |
| ENSG00000117425 | PTCH2 | turquoise |
| ENSG00000117461 | PIK3R3 | turquoise |
| ENSG00000117475 | BLZF1 | turquoise |
| ENSG00000117477 | CCDC181 | turquoise |
| ENSG00000117479 | SLC19A2 | turquoise |
| ENSG00000117480 | FAAH | turquoise |
| ENSG00000117481 | NSUN4 | turquoise |

| | | |
|---|---|---|
| ENSG00000117500 | TMED5 | turquoise |
| ENSG00000117505 | DR1 | turquoise |
| ENSG00000117519 | CNN3 | turquoise |
| ENSG00000117523 | PRRC2C | turquoise |
| ENSG00000117528 | ABCD3 | turquoise |
| ENSG00000117533 | VAMP4 | turquoise |
| ENSG00000117543 | DPH5 | turquoise |
| ENSG00000117569 | PTBP2 | turquoise |
| ENSG00000117586 | TNFSF4 | turquoise |
| ENSG00000117593 | DARS2 | turquoise |
| ENSG00000117597 | DIEXF | turquoise |
| ENSG00000117601 | SERPINC1 | turquoise |
| ENSG00000117602 | RCAN3 | turquoise |
| ENSG00000117614 | SYF2 | turquoise |
| ENSG00000117616 | RSRP1 | turquoise |
| ENSG00000117620 | SLC35A3 | turquoise |
| ENSG00000117625 | RCOR3 | turquoise |
| ENSG00000117640 | MTFR1L | turquoise |
| ENSG00000117682 | DHDDS | turquoise |
| ENSG00000117697 | NSL1 | turquoise |
| ENSG00000117713 | ARID1A | turquoise |
| ENSG00000117724 | CENPF | turquoise |
| ENSG00000117748 | RPA2 | turquoise |
| ENSG00000117751 | PPP1R8 | turquoise |
| ENSG00000117859 | OSBPL9 | turquoise |
| ENSG00000117862 | TXNDC12 | turquoise |
| ENSG00000117877 | CD3EAP | turquoise |
| ENSG00000117906 | RCN2 | turquoise |
| ENSG00000117983 | MUC5B | turquoise |
| ENSG00000117984 | CTSD | turquoise |
| ENSG00000118007 | STAG1 | turquoise |
| ENSG00000118046 | STK11 | turquoise |
| ENSG00000118058 | KMT2A | turquoise |
| ENSG00000118156 | ZNF541 | turquoise |
| ENSG00000118193 | KIF14 | turquoise |
| ENSG00000118197 | DDX59 | turquoise |
| ENSG00000118200 | CAMSAP2 | turquoise |
| ENSG00000118242 | MREG | turquoise |
| ENSG00000118246 | FASTKD2 | turquoise |
| ENSG00000118260 | CREB1 | turquoise |
| ENSG00000118276 | B4GALT6 | turquoise |
| ENSG00000118308 | LRMP | turquoise |
| ENSG00000118402 | ELOVL4 | turquoise |
| ENSG00000118412 | CASP8AP2 | turquoise |
| ENSG00000118418 | HMGN3 | turquoise |
| ENSG00000118432 | CNR1 | turquoise |
| ENSG00000118454 | ANKRD13C | turquoise |
| ENSG00000118482 | PHF3 | turquoise |

| | | |
|---|---|---|
| ENSG00000118495 | PLAGL1 | turquoise |
| ENSG00000118496 | FBXO30 | turquoise |
| ENSG00000118503 | TNFAIP3 | turquoise |
| ENSG00000118507 | AKAP7 | turquoise |
| ENSG00000118508 | RAB32 | turquoise |
| ENSG00000118513 | MYB | turquoise |
| ENSG00000118514 | ALDH8A1 | turquoise |
| ENSG00000118515 | SGK1 | turquoise |
| ENSG00000118518 | RNF146 | turquoise |
| ENSG00000118557 | PMFBP1 | turquoise |
| ENSG00000118564 | FBXL5 | turquoise |
| ENSG00000118579 | MED28 | turquoise |
| ENSG00000118596 | SLC16A7 | turquoise |
| ENSG00000118600 | TMEM5 | turquoise |
| ENSG00000118620 | ZNF430 | turquoise |
| ENSG00000118655 | DCLRE1B | turquoise |
| ENSG00000118680 | MYL12B | turquoise |
| ENSG00000118689 | FOXO3 | turquoise |
| ENSG00000118762 | PKD2 | turquoise |
| ENSG00000118855 | MFSD1 | turquoise |
| ENSG00000118873 | RAB3GAP2 | turquoise |
| ENSG00000118894 | EEF2KMT | turquoise |
| ENSG00000118922 | KLF12 | turquoise |
| ENSG00000118960 | HS1BP3 | turquoise |
| ENSG00000118961 | LDAH | turquoise |
| ENSG00000118965 | WDR35 | turquoise |
| ENSG00000118971 | CCND2 | turquoise |
| ENSG00000118985 | ELL2 | turquoise |
| ENSG00000119004 | CYP20A1 | turquoise |
| ENSG00000119013 | NDUFB3 | turquoise |
| ENSG00000119041 | GTF3C3 | turquoise |
| ENSG00000119042 | SATB2 | turquoise |
| ENSG00000119048 | UBE2B | turquoise |
| ENSG00000119138 | KLF9 | turquoise |
| ENSG00000119285 | HEATR1 | turquoise |
| ENSG00000119314 | PTBP3 | turquoise |
| ENSG00000119318 | RAD23B | turquoise |
| ENSG00000119321 | FKBP15 | turquoise |
| ENSG00000119328 | FAM206A | turquoise |
| ENSG00000119335 | SET | turquoise |
| ENSG00000119392 | GLE1 | turquoise |
| ENSG00000119396 | RAB14 | turquoise |
| ENSG00000119397 | CNTRL | turquoise |
| ENSG00000119401 | TRIM32 | turquoise |
| ENSG00000119402 | FBXW2 | turquoise |
| ENSG00000119414 | PPP6C | turquoise |
| ENSG00000119457 | SLC46A2 | turquoise |
| ENSG00000119471 | HSDL2 | turquoise |

| | | |
|---|---|---|
| ENSG00000119508 | NR4A3 | turquoise |
| ENSG00000119514 | GALNT12 | turquoise |
| ENSG00000119523 | ALG2 | turquoise |
| ENSG00000119537 | KDSR | turquoise |
| ENSG00000119541 | VPS4B | turquoise |
| ENSG00000119559 | C19orf25 | turquoise |
| ENSG00000119596 | YLPM1 | turquoise |
| ENSG00000119599 | DCAF4 | turquoise |
| ENSG00000119616 | FCF1 | turquoise |
| ENSG00000119630 | PGF | turquoise |
| ENSG00000119638 | NEK9 | turquoise |
| ENSG00000119640 | ACYP1 | turquoise |
| ENSG00000119650 | IFT43 | turquoise |
| ENSG00000119661 | DNAL1 | turquoise |
| ENSG00000119669 | IRF2BPL | turquoise |
| ENSG00000119673 | ACOT2 | turquoise |
| ENSG00000119682 | AREL1 | turquoise |
| ENSG00000119684 | MLH3 | turquoise |
| ENSG00000119685 | TTLL5 | turquoise |
| ENSG00000119689 | DLST | turquoise |
| ENSG00000119707 | RBM25 | turquoise |
| ENSG00000119729 | RHOQ | turquoise |
| ENSG00000119760 | SUPT7L | turquoise |
| ENSG00000119777 | TMEM214 | turquoise |
| ENSG00000119778 | ATAD2B | turquoise |
| ENSG00000119787 | ATL2 | turquoise |
| ENSG00000119801 | YPEL5 | turquoise |
| ENSG00000119812 | FAM98A | turquoise |
| ENSG00000119820 | YIPF4 | turquoise |
| ENSG00000119844 | AFTPH | turquoise |
| ENSG00000119865 | CNRIP1 | turquoise |
| ENSG00000119878 | CRIPT | turquoise |
| ENSG00000119899 | SLC17A5 | turquoise |
| ENSG00000119900 | OGFRL1 | turquoise |
| ENSG00000119906 | SLF2 | turquoise |
| ENSG00000119912 | IDE | turquoise |
| ENSG00000119915 | ELOVL3 | turquoise |
| ENSG00000119927 | GPAM | turquoise |
| ENSG00000119929 | CUTC | turquoise |
| ENSG00000119953 | SMNDC1 | turquoise |
| ENSG00000119965 | C10orf88 | turquoise |
| ENSG00000119969 | HELLS | turquoise |
| ENSG00000119977 | TCTN3 | turquoise |
| ENSG00000119979 | FAM45A | turquoise |
| ENSG00000120008 | WDR11 | turquoise |
| ENSG00000120049 | KCNIP2 | turquoise |
| ENSG00000120051 | CFAP58 | turquoise |
| ENSG00000120063 | GNA13 | turquoise |

| | | |
|---|---|---|
| ENSG00000120071 | KANSL1 | turquoise |
| ENSG00000120129 | DUSP1 | turquoise |
| ENSG00000120137 | PANK3 | turquoise |
| ENSG00000120158 | RCL1 | turquoise |
| ENSG00000120159 | CAAP1 | turquoise |
| ENSG00000120162 | MOB3B | turquoise |
| ENSG00000120254 | MTHFD1L | turquoise |
| ENSG00000120256 | LRP11 | turquoise |
| ENSG00000120262 | CCDC170 | turquoise |
| ENSG00000120265 | PCMT1 | turquoise |
| ENSG00000120278 | PLEKHG1 | turquoise |
| ENSG00000120279 | MYCT1 | turquoise |
| ENSG00000120280 | CXorf21 | turquoise |
| ENSG00000120314 | WDR55 | turquoise |
| ENSG00000120370 | GORAB | turquoise |
| ENSG00000120438 | TCP1 | turquoise |
| ENSG00000120451 | SNX19 | turquoise |
| ENSG00000120500 | ARR3 | turquoise |
| ENSG00000120519 | SLC10A7 | turquoise |
| ENSG00000120526 | NUDCD1 | turquoise |
| ENSG00000120533 | ENY2 | turquoise |
| ENSG00000120539 | MASTL | turquoise |
| ENSG00000120616 | EPC1 | turquoise |
| ENSG00000120647 | CCDC77 | turquoise |
| ENSG00000120675 | DNAJC15 | turquoise |
| ENSG00000120685 | PROSER1 | turquoise |
| ENSG00000120686 | UFM1 | turquoise |
| ENSG00000120688 | WBP4 | turquoise |
| ENSG00000120690 | ELF1 | turquoise |
| ENSG00000120693 | SMAD9 | turquoise |
| ENSG00000120694 | HSPH1 | turquoise |
| ENSG00000120696 | KBTBD7 | turquoise |
| ENSG00000120697 | ALG5 | turquoise |
| ENSG00000120699 | EXOSC8 | turquoise |
| ENSG00000120705 | ETF1 | turquoise |
| ENSG00000120727 | PAIP2 | turquoise |
| ENSG00000120729 | MYOT | turquoise |
| ENSG00000120756 | PLS1 | turquoise |
| ENSG00000120784 | ZFP30 | turquoise |
| ENSG00000120798 | NR2C1 | turquoise |
| ENSG00000120800 | UTP20 | turquoise |
| ENSG00000120802 | TMPO | turquoise |
| ENSG00000120805 | ARL1 | turquoise |
| ENSG00000120820 | GLT8D2 | turquoise |
| ENSG00000120837 | NFYB | turquoise |
| ENSG00000120860 | WASHC3 | turquoise |
| ENSG00000120889 | TNFRSF10B | turquoise |
| ENSG00000120910 | PPP3CC | turquoise |

| | | |
|---|---|---|
| ENSG00000120925 | RNF170 | turquoise |
| ENSG00000120948 | TARDBP | turquoise |
| ENSG00000120949 | TNFRSF8 | turquoise |
| ENSG00000120992 | LYPLA1 | turquoise |
| ENSG00000121022 | COPS5 | turquoise |
| ENSG00000121039 | RDH10 | turquoise |
| ENSG00000121057 | AKAP1 | turquoise |
| ENSG00000121058 | COIL | turquoise |
| ENSG00000121067 | SPOP | turquoise |
| ENSG00000121210 | TMEM131L | turquoise |
| ENSG00000121274 | PAPD5 | turquoise |
| ENSG00000121289 | CEP89 | turquoise |
| ENSG00000121310 | ECHDC2 | turquoise |
| ENSG00000121350 | PYROXD1 | turquoise |
| ENSG00000121390 | PSPC1 | turquoise |
| ENSG00000121410 | A1BG | turquoise |
| ENSG00000121481 | RNF2 | turquoise |
| ENSG00000121486 | TRMT1L | turquoise |
| ENSG00000121552 | CSTA | turquoise |
| ENSG00000121577 | POPDC2 | turquoise |
| ENSG00000121578 | B4GALT4 | turquoise |
| ENSG00000121579 | NAA50 | turquoise |
| ENSG00000121621 | KIF18A | turquoise |
| ENSG00000121644 | DESI2 | turquoise |
| ENSG00000121653 | MAPK8IP1 | turquoise |
| ENSG00000121671 | CRY2 | turquoise |
| ENSG00000121716 | PILRB | turquoise |
| ENSG00000121741 | ZMYM2 | turquoise |
| ENSG00000121743 | GJA3 | turquoise |
| ENSG00000121749 | TBC1D15 | turquoise |
| ENSG00000121766 | ZCCHC17 | turquoise |
| ENSG00000121774 | KHDRBS1 | turquoise |
| ENSG00000121807 | CCR2 | turquoise |
| ENSG00000121851 | POLR3GL | turquoise |
| ENSG00000121864 | ZNF639 | turquoise |
| ENSG00000121879 | PIK3CA | turquoise |
| ENSG00000121892 | PDS5A | turquoise |
| ENSG00000121895 | TMEM156 | turquoise |
| ENSG00000121897 | LIAS | turquoise |
| ENSG00000121931 | LRIF1 | turquoise |
| ENSG00000121964 | GTDC1 | turquoise |
| ENSG00000121966 | CXCR4 | turquoise |
| ENSG00000121988 | ZRANB3 | turquoise |
| ENSG00000121989 | ACVR2A | turquoise |
| ENSG00000122026 | RPL21 | turquoise |
| ENSG00000122033 | MTIF3 | turquoise |
| ENSG00000122035 | RASL11A | turquoise |
| ENSG00000122042 | UBL3 | turquoise |

| | | |
|---|---|---|
| ENSG00000122043 | LINC00544 | turquoise |
| ENSG00000122068 | FYTTD1 | turquoise |
| ENSG00000122126 | OCRL | turquoise |
| ENSG00000122257 | RBBP6 | turquoise |
| ENSG00000122299 | ZC3H7A | turquoise |
| ENSG00000122335 | SERAC1 | turquoise |
| ENSG00000122359 | ANXA11 | turquoise |
| ENSG00000122376 | FAM35A | turquoise |
| ENSG00000122386 | ZNF205 | turquoise |
| ENSG00000122390 | NAA60 | turquoise |
| ENSG00000122406 | RPL5 | turquoise |
| ENSG00000122417 | ODF2L | turquoise |
| ENSG00000122432 | SPATA1 | turquoise |
| ENSG00000122435 | TRMT13 | turquoise |
| ENSG00000122477 | LRRC39 | turquoise |
| ENSG00000122481 | RWDD3 | turquoise |
| ENSG00000122482 | ZNF644 | turquoise |
| ENSG00000122483 | CCDC18 | turquoise |
| ENSG00000122484 | RPAP2 | turquoise |
| ENSG00000122507 | BBS9 | turquoise |
| ENSG00000122515 | ZMIZ2 | turquoise |
| ENSG00000122543 | OCM | turquoise |
| ENSG00000122545 | 7-Sep | turquoise |
| ENSG00000122547 | EEPD1 | turquoise |
| ENSG00000122550 | KLHL7 | turquoise |
| ENSG00000122557 | HERPUD2 | turquoise |
| ENSG00000122565 | CBX3 | turquoise |
| ENSG00000122566 | HNRNPA2B1 | turquoise |
| ENSG00000122591 | FAM126A | turquoise |
| ENSG00000122643 | NT5C3A | turquoise |
| ENSG00000122644 | ARL4A | turquoise |
| ENSG00000122674 | CCZ1 | turquoise |
| ENSG00000122678 | POLM | turquoise |
| ENSG00000122679 | RAMP3 | turquoise |
| ENSG00000122692 | SMU1 | turquoise |
| ENSG00000122696 | SLC25A51 | turquoise |
| ENSG00000122707 | RECK | turquoise |
| ENSG00000122733 | PHF24 | turquoise |
| ENSG00000122741 | DCAF10 | turquoise |
| ENSG00000122779 | TRIM24 | turquoise |
| ENSG00000122786 | CALD1 | turquoise |
| ENSG00000122862 | SRGN | turquoise |
| ENSG00000122870 | BICC1 | turquoise |
| ENSG00000122873 | CISD1 | turquoise |
| ENSG00000122877 | EGR2 | turquoise |
| ENSG00000122884 | P4HA1 | turquoise |
| ENSG00000122958 | VPS26A | turquoise |
| ENSG00000122965 | RBM19 | turquoise |

| | | |
|---|---|---|
| ENSG00000122966 | CIT | turquoise |
| ENSG00000122986 | HVCN1 | turquoise |
| ENSG00000123009 | NME2P1 | turquoise |
| ENSG00000123080 | CDKN2C | turquoise |
| ENSG00000123091 | RNF11 | turquoise |
| ENSG00000123106 | CCDC91 | turquoise |
| ENSG00000123124 | WWP1 | turquoise |
| ENSG00000123136 | DDX39A | turquoise |
| ENSG00000123154 | WDR83 | turquoise |
| ENSG00000123178 | SPRYD7 | turquoise |
| ENSG00000123200 | ZC3H13 | turquoise |
| ENSG00000123219 | CENPK | turquoise |
| ENSG00000123240 | OPTN | turquoise |
| ENSG00000123268 | ATF1 | turquoise |
| ENSG00000123349 | PFDN5 | turquoise |
| ENSG00000123353 | ORMDL2 | turquoise |
| ENSG00000123358 | NR4A1 | turquoise |
| ENSG00000123360 | PDE1B | turquoise |
| ENSG00000123374 | CDK2 | turquoise |
| ENSG00000123411 | IKZF4 | turquoise |
| ENSG00000123415 | SMUG1 | turquoise |
| ENSG00000123416 | TUBA1B | turquoise |
| ENSG00000123473 | STIL | turquoise |
| ENSG00000123505 | AMD1 | turquoise |
| ENSG00000123545 | NDUFAF4 | turquoise |
| ENSG00000123552 | USP45 | turquoise |
| ENSG00000123562 | MORF4L2 | turquoise |
| ENSG00000123570 | RAB9B | turquoise |
| ENSG00000123575 | FAM199X | turquoise |
| ENSG00000123595 | RAB9A | turquoise |
| ENSG00000123600 | METTL8 | turquoise |
| ENSG00000123607 | TTC21B | turquoise |
| ENSG00000123612 | ACVR1C | turquoise |
| ENSG00000123636 | BAZ2B | turquoise |
| ENSG00000123684 | LPGAT1 | turquoise |
| ENSG00000123685 | BATF3 | turquoise |
| ENSG00000123700 | KCNJ2 | turquoise |
| ENSG00000123728 | RAP2C | turquoise |
| ENSG00000123737 | EXOSC9 | turquoise |
| ENSG00000123739 | PLA2G12A | turquoise |
| ENSG00000123810 | B9D2 | turquoise |
| ENSG00000123815 | COQ8B | turquoise |
| ENSG00000123836 | PFKFB2 | turquoise |
| ENSG00000123838 | C4BPA | turquoise |
| ENSG00000123901 | GPR83 | turquoise |
| ENSG00000123975 | CKS2 | turquoise |
| ENSG00000123983 | ACSL3 | turquoise |
| ENSG00000124006 | OBSL1 | turquoise |

| | | |
|---|---|---|
| ENSG00000124019 | FAM124B | turquoise |
| ENSG00000124074 | ENKD1 | turquoise |
| ENSG00000124102 | PI3 | turquoise |
| ENSG00000124104 | SNX21 | turquoise |
| ENSG00000124155 | PIGT | turquoise |
| ENSG00000124164 | VAPB | turquoise |
| ENSG00000124171 | PARD6B | turquoise |
| ENSG00000124172 | ATP5E | turquoise |
| ENSG00000124193 | SRSF6 | turquoise |
| ENSG00000124198 | ARFGEF2 | turquoise |
| ENSG00000124207 | CSE1L | turquoise |
| ENSG00000124209 | RAB22A | turquoise |
| ENSG00000124214 | STAU1 | turquoise |
| ENSG00000124217 | MOCS3 | turquoise |
| ENSG00000124224 | PPP4R1L | turquoise |
| ENSG00000124257 | NEURL2 | turquoise |
| ENSG00000124275 | MTRR | turquoise |
| ENSG00000124279 | FASTKD3 | turquoise |
| ENSG00000124302 | CHST8 | turquoise |
| ENSG00000124313 | IQSEC2 | turquoise |
| ENSG00000124333 | VAMP7 | turquoise |
| ENSG00000124334 | IL9R | turquoise |
| ENSG00000124370 | MCEE | turquoise |
| ENSG00000124380 | SNRNP27 | turquoise |
| ENSG00000124383 | MPHOSPH10 | turquoise |
| ENSG00000124399 | NDUFB4P12 | turquoise |
| ENSG00000124406 | ATP8A1 | turquoise |
| ENSG00000124422 | USP22 | turquoise |
| ENSG00000124429 | POF1B | turquoise |
| ENSG00000124459 | ZNF45 | turquoise |
| ENSG00000124486 | USP9X | turquoise |
| ENSG00000124491 | F13A1 | turquoise |
| ENSG00000124508 | BTN2A2 | turquoise |
| ENSG00000124523 | SIRT5 | turquoise |
| ENSG00000124532 | MRS2 | turquoise |
| ENSG00000124535 | WRNIP1 | turquoise |
| ENSG00000124541 | RRP36 | turquoise |
| ENSG00000124587 | PEX6 | turquoise |
| ENSG00000124588 | NQO2 | turquoise |
| ENSG00000124593 | AL365205.1 | turquoise |
| ENSG00000124608 | AARS2 | turquoise |
| ENSG00000124613 | ZNF391 | turquoise |
| ENSG00000124641 | MED20 | turquoise |
| ENSG00000124659 | TBCC | turquoise |
| ENSG00000124713 | GNMT | turquoise |
| ENSG00000124766 | SOX4 | turquoise |
| ENSG00000124767 | GLO1 | turquoise |
| ENSG00000124772 | CPNE5 | turquoise |

| | | |
|---|---|---|
| ENSG00000124783 | SSR1 | turquoise |
| ENSG00000124784 | RIOK1 | turquoise |
| ENSG00000124785 | NRN1 | turquoise |
| ENSG00000124786 | SLC35B3 | turquoise |
| ENSG00000124789 | NUP153 | turquoise |
| ENSG00000124795 | DEK | turquoise |
| ENSG00000124802 | EEF1E1 | turquoise |
| ENSG00000124813 | RUNX2 | turquoise |
| ENSG00000124882 | EREG | turquoise |
| ENSG00000124942 | AHNAK | turquoise |
| ENSG00000125089 | SH3TC1 | turquoise |
| ENSG00000125107 | CNOT1 | turquoise |
| ENSG00000125122 | LRRC29 | turquoise |
| ENSG00000125149 | C16orf70 | turquoise |
| ENSG00000125245 | GPR18 | turquoise |
| ENSG00000125246 | CLYBL | turquoise |
| ENSG00000125247 | TMTC4 | turquoise |
| ENSG00000125249 | RAP2A | turquoise |
| ENSG00000125257 | ABCC4 | turquoise |
| ENSG00000125266 | EFNB2 | turquoise |
| ENSG00000125304 | TM9SF2 | turquoise |
| ENSG00000125319 | C17orf53 | turquoise |
| ENSG00000125351 | UPF3B | turquoise |
| ENSG00000125354 | 6-Sep | turquoise |
| ENSG00000125384 | PTGER2 | turquoise |
| ENSG00000125388 | GRK4 | turquoise |
| ENSG00000125430 | HS3ST3B1 | turquoise |
| ENSG00000125434 | SLC25A35 | turquoise |
| ENSG00000125449 | ARMC7 | turquoise |
| ENSG00000125450 | NUP85 | turquoise |
| ENSG00000125454 | SLC25A19 | turquoise |
| ENSG00000125459 | MSTO1 | turquoise |
| ENSG00000125482 | TTF1 | turquoise |
| ENSG00000125484 | GTF3C4 | turquoise |
| ENSG00000125503 | PPP1R12C | turquoise |
| ENSG00000125505 | MBOAT7 | turquoise |
| ENSG00000125531 | FNDC11 | turquoise |
| ENSG00000125611 | CHCHD5 | turquoise |
| ENSG00000125629 | INSIG2 | turquoise |
| ENSG00000125630 | POLR1B | turquoise |
| ENSG00000125633 | CCDC93 | turquoise |
| ENSG00000125637 | PSD4 | turquoise |
| ENSG00000125650 | PSPN | turquoise |
| ENSG00000125676 | THOC2 | turquoise |
| ENSG00000125686 | MED1 | turquoise |
| ENSG00000125691 | RPL23 | turquoise |
| ENSG00000125703 | ATG4C | turquoise |
| ENSG00000125726 | CD70 | turquoise |

| | | |
|---|---|---|
| ENSG00000125730 | C3 | turquoise |
| ENSG00000125731 | SH2D3A | turquoise |
| ENSG00000125733 | TRIP10 | turquoise |
| ENSG00000125734 | GPR108 | turquoise |
| ENSG00000125741 | OPA3 | turquoise |
| ENSG00000125744 | RTN2 | turquoise |
| ENSG00000125755 | SYMPK | turquoise |
| ENSG00000125775 | SDCBP2 | turquoise |
| ENSG00000125780 | TGM3 | turquoise |
| ENSG00000125814 | NAPB | turquoise |
| ENSG00000125817 | CENPB | turquoise |
| ENSG00000125827 | TMX4 | turquoise |
| ENSG00000125843 | AP5S1 | turquoise |
| ENSG00000125845 | BMP2 | turquoise |
| ENSG00000125846 | ZNF133 | turquoise |
| ENSG00000125863 | MKKS | turquoise |
| ENSG00000125868 | DSTN | turquoise |
| ENSG00000125869 | LAMP5 | turquoise |
| ENSG00000125870 | SNRPB2 | turquoise |
| ENSG00000125871 | MGME1 | turquoise |
| ENSG00000125898 | FAM110A | turquoise |
| ENSG00000125900 | SIRPD | turquoise |
| ENSG00000125910 | S1PR4 | turquoise |
| ENSG00000125912 | NCLN | turquoise |
| ENSG00000125962 | ARMCX5 | turquoise |
| ENSG00000125967 | NECAB3 | turquoise |
| ENSG00000125970 | RALY | turquoise |
| ENSG00000125977 | EIF2S2 | turquoise |
| ENSG00000126001 | CEP250 | turquoise |
| ENSG00000126091 | ST3GAL3 | turquoise |
| ENSG00000126107 | HECTD3 | turquoise |
| ENSG00000126214 | KLC1 | turquoise |
| ENSG00000126215 | XRCC3 | turquoise |
| ENSG00000126216 | TUBGCP3 | turquoise |
| ENSG00000126217 | MCF2L | turquoise |
| ENSG00000126226 | PCID2 | turquoise |
| ENSG00000126231 | PROZ | turquoise |
| ENSG00000126246 | IGFLR1 | turquoise |
| ENSG00000126261 | UBA2 | turquoise |
| ENSG00000126368 | NR1D1 | turquoise |
| ENSG00000126460 | PRRG2 | turquoise |
| ENSG00000126461 | SCAF1 | turquoise |
| ENSG00000126464 | PRR12 | turquoise |
| ENSG00000126522 | ASL | turquoise |
| ENSG00000126524 | SBDS | turquoise |
| ENSG00000126562 | WNK4 | turquoise |
| ENSG00000126581 | BECN1 | turquoise |
| ENSG00000126602 | TRAP1 | turquoise |

| | | |
|---|---|---|
| ENSG00000126653 | NSRP1 | turquoise |
| ENSG00000126749 | EMG1 | turquoise |
| ENSG00000126759 | CFP | turquoise |
| ENSG00000126773 | PCNX4 | turquoise |
| ENSG00000126775 | ATG14 | turquoise |
| ENSG00000126777 | KTN1 | turquoise |
| ENSG00000126787 | DLGAP5 | turquoise |
| ENSG00000126803 | HSPA2 | turquoise |
| ENSG00000126804 | ZBTB1 | turquoise |
| ENSG00000126814 | TRMT5 | turquoise |
| ENSG00000126821 | SGPP1 | turquoise |
| ENSG00000126822 | PLEKHG3 | turquoise |
| ENSG00000126858 | RHOT1 | turquoise |
| ENSG00000126860 | EVI2A | turquoise |
| ENSG00000126861 | OMG | turquoise |
| ENSG00000126870 | WDR60 | turquoise |
| ENSG00000126882 | FAM78A | turquoise |
| ENSG00000126883 | NUP214 | turquoise |
| ENSG00000126903 | SLC10A3 | turquoise |
| ENSG00000126934 | MAP2K2 | turquoise |
| ENSG00000126945 | HNRNPH2 | turquoise |
| ENSG00000126947 | ARMCX1 | turquoise |
| ENSG00000127054 | INTS11 | turquoise |
| ENSG00000127081 | ZNF484 | turquoise |
| ENSG00000127124 | HIVEP3 | turquoise |
| ENSG00000127125 | PPCS | turquoise |
| ENSG00000127152 | BCL11B | turquoise |
| ENSG00000127184 | COX7C | turquoise |
| ENSG00000127249 | ATP13A4 | turquoise |
| ENSG00000127314 | RAP1B | turquoise |
| ENSG00000127328 | RAB3IP | turquoise |
| ENSG00000127329 | PTPRB | turquoise |
| ENSG00000127334 | DYRK2 | turquoise |
| ENSG00000127337 | YEATS4 | turquoise |
| ENSG00000127364 | TAS2R4 | turquoise |
| ENSG00000127399 | LRRC61 | turquoise |
| ENSG00000127415 | IDUA | turquoise |
| ENSG00000127419 | TMEM175 | turquoise |
| ENSG00000127423 | AUNIP | turquoise |
| ENSG00000127445 | PIN1 | turquoise |
| ENSG00000127481 | UBR4 | turquoise |
| ENSG00000127483 | HP1BP3 | turquoise |
| ENSG00000127511 | SIN3B | turquoise |
| ENSG00000127526 | SLC35E1 | turquoise |
| ENSG00000127533 | F2RL3 | turquoise |
| ENSG00000127540 | UQCR11 | turquoise |
| ENSG00000127564 | PKMYT1 | turquoise |
| ENSG00000127578 | WFIKKN1 | turquoise |

| | | |
|---|---|---|
| ENSG00000127586 | CHTF18 | turquoise |
| ENSG00000127603 | MACF1 | turquoise |
| ENSG00000127720 | METTL25 | turquoise |
| ENSG00000127743 | IL17B | turquoise |
| ENSG00000127774 | EMC6 | turquoise |
| ENSG00000127824 | TUBA4A | turquoise |
| ENSG00000127831 | VIL1 | turquoise |
| ENSG00000127870 | RNF6 | turquoise |
| ENSG00000127914 | AKAP9 | turquoise |
| ENSG00000127920 | GNG11 | turquoise |
| ENSG00000127947 | PTPN12 | turquoise |
| ENSG00000127948 | POR | turquoise |
| ENSG00000127951 | FGL2 | turquoise |
| ENSG00000127952 | STYXL1 | turquoise |
| ENSG00000127954 | STEAP4 | turquoise |
| ENSG00000127980 | PEX1 | turquoise |
| ENSG00000127989 | MTERF1 | turquoise |
| ENSG00000127993 | RBM48 | turquoise |
| ENSG00000127995 | CASD1 | turquoise |
| ENSG00000128000 | ZNF780B | turquoise |
| ENSG00000128011 | LRFN1 | turquoise |
| ENSG00000128039 | SRD5A3 | turquoise |
| ENSG00000128040 | SPINK2 | turquoise |
| ENSG00000128059 | PPAT | turquoise |
| ENSG00000128159 | TUBGCP6 | turquoise |
| ENSG00000128165 | ADM2 | turquoise |
| ENSG00000128253 | RFPL2 | turquoise |
| ENSG00000128271 | ADORA2A | turquoise |
| ENSG00000128283 | CDC42EP1 | turquoise |
| ENSG00000128294 | TPST2 | turquoise |
| ENSG00000128298 | BAIAP2L2 | turquoise |
| ENSG00000128309 | MPST | turquoise |
| ENSG00000128311 | TST | turquoise |
| ENSG00000128340 | RAC2 | turquoise |
| ENSG00000128394 | APOBEC3F | turquoise |
| ENSG00000128438 | TBC1D27 | turquoise |
| ENSG00000128482 | RNF112 | turquoise |
| ENSG00000128513 | POT1 | turquoise |
| ENSG00000128534 | LSM8 | turquoise |
| ENSG00000128536 | CDHR3 | turquoise |
| ENSG00000128590 | DNAJB9 | turquoise |
| ENSG00000128595 | CALU | turquoise |
| ENSG00000128607 | KLHDC10 | turquoise |
| ENSG00000128609 | NDUFA5 | turquoise |
| ENSG00000128641 | MYO1B | turquoise |
| ENSG00000128654 | MTX2 | turquoise |
| ENSG00000128656 | CHN1 | turquoise |
| ENSG00000128699 | ORMDL1 | turquoise |

| | | |
|---|---|---|
| ENSG00000128708 | HAT1 | turquoise |
| ENSG00000128791 | TWSG1 | turquoise |
| ENSG00000128815 | WDFY4 | turquoise |
| ENSG00000128829 | EIF2AK4 | turquoise |
| ENSG00000128891 | CCDC32 | turquoise |
| ENSG00000128908 | INO80 | turquoise |
| ENSG00000128915 | ICE2 | turquoise |
| ENSG00000128928 | IVD | turquoise |
| ENSG00000128944 | KNSTRN | turquoise |
| ENSG00000128951 | DUT | turquoise |
| ENSG00000128973 | CLN6 | turquoise |
| ENSG00000128989 | ARPP19 | turquoise |
| ENSG00000129003 | VPS13C | turquoise |
| ENSG00000129038 | LOXL1 | turquoise |
| ENSG00000129048 | ACKR4 | turquoise |
| ENSG00000129055 | ANAPC13 | turquoise |
| ENSG00000129071 | MBD4 | turquoise |
| ENSG00000129083 | COPB1 | turquoise |
| ENSG00000129084 | PSMA1 | turquoise |
| ENSG00000129116 | PALLD | turquoise |
| ENSG00000129128 | SPCS3 | turquoise |
| ENSG00000129173 | E2F8 | turquoise |
| ENSG00000129194 | SOX15 | turquoise |
| ENSG00000129195 | PIMREG | turquoise |
| ENSG00000129219 | PLD2 | turquoise |
| ENSG00000129226 | CD68 | turquoise |
| ENSG00000129235 | TXNDC17 | turquoise |
| ENSG00000129245 | FXR2 | turquoise |
| ENSG00000129292 | PHF20L1 | turquoise |
| ENSG00000129315 | CCNT1 | turquoise |
| ENSG00000129317 | PUS7L | turquoise |
| ENSG00000129347 | KRI1 | turquoise |
| ENSG00000129422 | MTUS1 | turquoise |
| ENSG00000129437 | KLK14 | turquoise |
| ENSG00000129460 | NGDN | turquoise |
| ENSG00000129465 | RIPK3 | turquoise |
| ENSG00000129472 | RAB2B | turquoise |
| ENSG00000129473 | BCL2L2 | turquoise |
| ENSG00000129480 | DTD2 | turquoise |
| ENSG00000129515 | SNX6 | turquoise |
| ENSG00000129518 | EAPP | turquoise |
| ENSG00000129534 | MIS18BP1 | turquoise |
| ENSG00000129625 | REEP5 | turquoise |
| ENSG00000129636 | ITFG1 | turquoise |
| ENSG00000129646 | QRICH2 | turquoise |
| ENSG00000129673 | AANAT | turquoise |
| ENSG00000129675 | ARHGEF6 | turquoise |
| ENSG00000129680 | MAP7D3 | turquoise |

| | | |
|---|---|---|
| ENSG00000129749 | CHRNA10 | turquoise |
| ENSG00000129810 | SGO1 | turquoise |
| ENSG00000129824 | RPS4Y1 | turquoise |
| ENSG00000129911 | KLF16 | turquoise |
| ENSG00000129925 | TMEM8A | turquoise |
| ENSG00000129968 | ABHD17A | turquoise |
| ENSG00000130021 | PUDP | turquoise |
| ENSG00000130023 | ERMARD | turquoise |
| ENSG00000130024 | PHF10 | turquoise |
| ENSG00000130037 | KCNA5 | turquoise |
| ENSG00000130150 | MOSPD2 | turquoise |
| ENSG00000130164 | LDLR | turquoise |
| ENSG00000130175 | PRKCSH | turquoise |
| ENSG00000130182 | ZSCAN10 | turquoise |
| ENSG00000130202 | NECTIN2 | turquoise |
| ENSG00000130203 | APOE | turquoise |
| ENSG00000130222 | GADD45G | turquoise |
| ENSG00000130244 | FAM98C | turquoise |
| ENSG00000130254 | SAFB2 | turquoise |
| ENSG00000130270 | ATP8B3 | turquoise |
| ENSG00000130304 | SLC27A1 | turquoise |
| ENSG00000130309 | COLGALT1 | turquoise |
| ENSG00000130311 | DDA1 | turquoise |
| ENSG00000130313 | PGLS | turquoise |
| ENSG00000130338 | TULP4 | turquoise |
| ENSG00000130349 | C6orf203 | turquoise |
| ENSG00000130382 | MLLT1 | turquoise |
| ENSG00000130414 | NDUFA10 | turquoise |
| ENSG00000130429 | ARPC1B | turquoise |
| ENSG00000130449 | ZSWIM6 | turquoise |
| ENSG00000130475 | FCHO1 | turquoise |
| ENSG00000130479 | MAP1S | turquoise |
| ENSG00000130511 | SSBP4 | turquoise |
| ENSG00000130513 | GDF15 | turquoise |
| ENSG00000130517 | PGPEP1 | turquoise |
| ENSG00000130518 | KIAA1683 | turquoise |
| ENSG00000130529 | TRPM4 | turquoise |
| ENSG00000130559 | CAMSAP1 | turquoise |
| ENSG00000130584 | ZBTB46 | turquoise |
| ENSG00000130590 | SAMD10 | turquoise |
| ENSG00000130592 | LSP1 | turquoise |
| ENSG00000130595 | TNNT3 | turquoise |
| ENSG00000130598 | TNNI2 | turquoise |
| ENSG00000130635 | COL5A1 | turquoise |
| ENSG00000130640 | TUBGCP2 | turquoise |
| ENSG00000130649 | CYP2E1 | turquoise |
| ENSG00000130669 | PAK4 | turquoise |
| ENSG00000130695 | CEP85 | turquoise |

| | | |
|---|---|---|
| ENSG00000130702 | LAMA5 | turquoise |
| ENSG00000130713 | EXOSC2 | turquoise |
| ENSG00000130714 | POMT1 | turquoise |
| ENSG00000130717 | UCK1 | turquoise |
| ENSG00000130723 | PRRC2B | turquoise |
| ENSG00000130733 | YIPF2 | turquoise |
| ENSG00000130734 | ATG4D | turquoise |
| ENSG00000130741 | EIF2S3 | turquoise |
| ENSG00000130749 | ZC3H4 | turquoise |
| ENSG00000130755 | GMFG | turquoise |
| ENSG00000130758 | MAP3K10 | turquoise |
| ENSG00000130766 | SESN2 | turquoise |
| ENSG00000130768 | SMPDL3B | turquoise |
| ENSG00000130772 | MED18 | turquoise |
| ENSG00000130787 | HIP1R | turquoise |
| ENSG00000130810 | PPAN | turquoise |
| ENSG00000130812 | ANGPTL6 | turquoise |
| ENSG00000130816 | DNMT1 | turquoise |
| ENSG00000130818 | ZNF426 | turquoise |
| ENSG00000130826 | DKC1 | turquoise |
| ENSG00000130827 | PLXNA3 | turquoise |
| ENSG00000130830 | MPP1 | turquoise |
| ENSG00000130856 | ZNF236 | turquoise |
| ENSG00000130881 | LRP3 | turquoise |
| ENSG00000130935 | NOL11 | turquoise |
| ENSG00000130962 | PRRG1 | turquoise |
| ENSG00000130997 | POLN | turquoise |
| ENSG00000131013 | PPIL4 | turquoise |
| ENSG00000131018 | SYNE1 | turquoise |
| ENSG00000131023 | LATS1 | turquoise |
| ENSG00000131037 | EPS8L1 | turquoise |
| ENSG00000131051 | RBM39 | turquoise |
| ENSG00000131061 | ZNF341 | turquoise |
| ENSG00000131067 | GGT7 | turquoise |
| ENSG00000131069 | ACSS2 | turquoise |
| ENSG00000131080 | EDA2R | turquoise |
| ENSG00000131115 | ZNF227 | turquoise |
| ENSG00000131165 | CHMP1A | turquoise |
| ENSG00000131171 | SH3BGRL | turquoise |
| ENSG00000131174 | COX7B | turquoise |
| ENSG00000131187 | F12 | turquoise |
| ENSG00000131188 | PRR7 | turquoise |
| ENSG00000131203 | IDO1 | turquoise |
| ENSG00000131238 | PPT1 | turquoise |
| ENSG00000131263 | RLIM | turquoise |
| ENSG00000131323 | TRAF3 | turquoise |
| ENSG00000131370 | SH3BP5 | turquoise |
| ENSG00000131374 | TBC1D5 | turquoise |

| | | |
|---|---|---|
| ENSG00000131375 | CAPN7 | turquoise |
| ENSG00000131379 | C3orf20 | turquoise |
| ENSG00000131398 | KCNC3 | turquoise |
| ENSG00000131400 | NAPSA | turquoise |
| ENSG00000131408 | NR1H2 | turquoise |
| ENSG00000131409 | LRRC4B | turquoise |
| ENSG00000131437 | KIF3A | turquoise |
| ENSG00000131446 | MGAT1 | turquoise |
| ENSG00000131462 | TUBG1 | turquoise |
| ENSG00000131473 | ACLY | turquoise |
| ENSG00000131507 | NDFIP1 | turquoise |
| ENSG00000131558 | EXOC4 | turquoise |
| ENSG00000131584 | ACAP3 | turquoise |
| ENSG00000131591 | C1orf159 | turquoise |
| ENSG00000131626 | PPFIA1 | turquoise |
| ENSG00000131650 | KREMEN2 | turquoise |
| ENSG00000131653 | TRAF7 | turquoise |
| ENSG00000131669 | NINJ1 | turquoise |
| ENSG00000131725 | WDR44 | turquoise |
| ENSG00000131732 | ZCCHC9 | turquoise |
| ENSG00000131747 | TOP2A | turquoise |
| ENSG00000131748 | STARD3 | turquoise |
| ENSG00000131773 | KHDRBS3 | turquoise |
| ENSG00000131781 | FMO5 | turquoise |
| ENSG00000131788 | PIAS3 | turquoise |
| ENSG00000131791 | PRKAB2 | turquoise |
| ENSG00000131844 | MCCC2 | turquoise |
| ENSG00000131845 | ZNF304 | turquoise |
| ENSG00000131848 | ZSCAN5A | turquoise |
| ENSG00000131849 | ZNF132 | turquoise |
| ENSG00000131871 | SELENOS | turquoise |
| ENSG00000131873 | CHSY1 | turquoise |
| ENSG00000131885 | KRT17P1 | turquoise |
| ENSG00000131931 | THAP1 | turquoise |
| ENSG00000131943 | C19orf12 | turquoise |
| ENSG00000131966 | ACTR10 | turquoise |
| ENSG00000131979 | GCH1 | turquoise |
| ENSG00000132000 | PODNL1 | turquoise |
| ENSG00000132004 | FBXW9 | turquoise |
| ENSG00000132005 | RFX1 | turquoise |
| ENSG00000132016 | C19orf57 | turquoise |
| ENSG00000132017 | DCAF15 | turquoise |
| ENSG00000132024 | CC2D1A | turquoise |
| ENSG00000132128 | LRRC41 | turquoise |
| ENSG00000132141 | CCT6B | turquoise |
| ENSG00000132170 | PPARG | turquoise |
| ENSG00000132196 | HSD17B7 | turquoise |
| ENSG00000132254 | ARFIP2 | turquoise |

| | | |
|---|---|---|
| ENSG00000132275 | RRP8 | turquoise |
| ENSG00000132286 | TIMM10B | turquoise |
| ENSG00000132294 | EFR3A | turquoise |
| ENSG00000132300 | PTCD3 | turquoise |
| ENSG00000132313 | MRPL35 | turquoise |
| ENSG00000132321 | IQCA1 | turquoise |
| ENSG00000132326 | PER2 | turquoise |
| ENSG00000132334 | PTPRE | turquoise |
| ENSG00000132356 | PRKAA1 | turquoise |
| ENSG00000132376 | INPP5K | turquoise |
| ENSG00000132386 | SERPINF1 | turquoise |
| ENSG00000132388 | UBE2G1 | turquoise |
| ENSG00000132406 | TMEM128 | turquoise |
| ENSG00000132424 | PNISR | turquoise |
| ENSG00000132434 | LANCL2 | turquoise |
| ENSG00000132436 | FIGNL1 | turquoise |
| ENSG00000132463 | GRSF1 | turquoise |
| ENSG00000132466 | ANKRD17 | turquoise |
| ENSG00000132467 | UTP3 | turquoise |
| ENSG00000132478 | UNK | turquoise |
| ENSG00000132481 | TRIM47 | turquoise |
| ENSG00000132485 | ZRANB2 | turquoise |
| ENSG00000132518 | GUCY2D | turquoise |
| ENSG00000132522 | GPS2 | turquoise |
| ENSG00000132535 | DLG4 | turquoise |
| ENSG00000132541 | RIDA | turquoise |
| ENSG00000132561 | MATN2 | turquoise |
| ENSG00000132563 | REEP2 | turquoise |
| ENSG00000132581 | SDF2 | turquoise |
| ENSG00000132600 | PRMT7 | turquoise |
| ENSG00000132603 | NIP7 | turquoise |
| ENSG00000132612 | VPS4A | turquoise |
| ENSG00000132613 | MTSS1L | turquoise |
| ENSG00000132623 | ANKEF1 | turquoise |
| ENSG00000132635 | PCED1A | turquoise |
| ENSG00000132640 | BTBD3 | turquoise |
| ENSG00000132646 | PCNA | turquoise |
| ENSG00000132664 | POLR3F | turquoise |
| ENSG00000132669 | RIN2 | turquoise |
| ENSG00000132670 | PTPRA | turquoise |
| ENSG00000132680 | KIAA0907 | turquoise |
| ENSG00000132716 | DCAF8 | turquoise |
| ENSG00000132740 | IGHMBP2 | turquoise |
| ENSG00000132749 | TESMIN | turquoise |
| ENSG00000132763 | MMACHC | turquoise |
| ENSG00000132768 | DPH2 | turquoise |
| ENSG00000132824 | SERINC3 | turquoise |
| ENSG00000132825 | PPP1R3D | turquoise |

| | | |
|---|---|---|
| ENSG00000132842 | AP3B1 | turquoise |
| ENSG00000132846 | ZBED3 | turquoise |
| ENSG00000132849 | PATJ | turquoise |
| ENSG00000132881 | RSG1 | turquoise |
| ENSG00000132906 | CASP9 | turquoise |
| ENSG00000132912 | DCTN4 | turquoise |
| ENSG00000132932 | ATP8A2 | turquoise |
| ENSG00000132938 | MTUS2 | turquoise |
| ENSG00000132950 | ZMYM5 | turquoise |
| ENSG00000132952 | USPL1 | turquoise |
| ENSG00000132953 | XPO4 | turquoise |
| ENSG00000132964 | CDK8 | turquoise |
| ENSG00000132965 | ALOX5AP | turquoise |
| ENSG00000132967 | HMGB1P5 | turquoise |
| ENSG00000133027 | PEMT | turquoise |
| ENSG00000133030 | MPRIP | turquoise |
| ENSG00000133048 | CHI3L1 | turquoise |
| ENSG00000133055 | MYBPH | turquoise |
| ENSG00000133056 | PIK3C2B | turquoise |
| ENSG00000133065 | SLC41A1 | turquoise |
| ENSG00000133101 | CCNA1 | turquoise |
| ENSG00000133103 | COG6 | turquoise |
| ENSG00000133104 | SPART | turquoise |
| ENSG00000133105 | RXFP2 | turquoise |
| ENSG00000133111 | RFXAP | turquoise |
| ENSG00000133112 | TPT1 | turquoise |
| ENSG00000133114 | GPALPP1 | turquoise |
| ENSG00000133116 | KL | turquoise |
| ENSG00000133119 | RFC3 | turquoise |
| ENSG00000133131 | MORC4 | turquoise |
| ENSG00000133136 | GNG5P2 | turquoise |
| ENSG00000133138 | TBC1D8B | turquoise |
| ENSG00000133169 | BEX1 | turquoise |
| ENSG00000133193 | FAM104A | turquoise |
| ENSG00000133195 | SLC39A11 | turquoise |
| ENSG00000133226 | SRRM1 | turquoise |
| ENSG00000133243 | BTBD2 | turquoise |
| ENSG00000133246 | PRAM1 | turquoise |
| ENSG00000133247 | KMT5C | turquoise |
| ENSG00000133275 | CSNK1G2 | turquoise |
| ENSG00000133302 | SLF1 | turquoise |
| ENSG00000133393 | FOPNL | turquoise |
| ENSG00000133398 | MED10 | turquoise |
| ENSG00000133424 | LARGE1 | turquoise |
| ENSG00000133466 | C1QTNF6 | turquoise |
| ENSG00000133477 | FAM83F | turquoise |
| ENSG00000133561 | GIMAP6 | turquoise |
| ENSG00000133612 | AGAP3 | turquoise |

| | | |
|---|---|---|
| ENSG00000133619 | KRBA1 | turquoise |
| ENSG00000133624 | ZNF767P | turquoise |
| ENSG00000133639 | BTG1 | turquoise |
| ENSG00000133641 | C12orf29 | turquoise |
| ENSG00000133657 | ATP13A3 | turquoise |
| ENSG00000133661 | SFTPD | turquoise |
| ENSG00000133703 | KRAS | turquoise |
| ENSG00000133704 | IPO8 | turquoise |
| ENSG00000133706 | LARS | turquoise |
| ENSG00000133731 | IMPA1 | turquoise |
| ENSG00000133739 | LRRCC1 | turquoise |
| ENSG00000133740 | E2F5 | turquoise |
| ENSG00000133773 | CCDC59 | turquoise |
| ENSG00000133789 | SWAP70 | turquoise |
| ENSG00000133808 | MICALCL | turquoise |
| ENSG00000133812 | SBF2 | turquoise |
| ENSG00000133816 | MICAL2 | turquoise |
| ENSG00000133818 | RRAS2 | turquoise |
| ENSG00000133835 | HSD17B4 | turquoise |
| ENSG00000133858 | ZFC3H1 | turquoise |
| ENSG00000133872 | SARAF | turquoise |
| ENSG00000133895 | MEN1 | turquoise |
| ENSG00000133943 | C14orf159 | turquoise |
| ENSG00000133961 | NUMB | turquoise |
| ENSG00000133985 | TTC9 | turquoise |
| ENSG00000134001 | EIF2S1 | turquoise |
| ENSG00000134046 | MBD2 | turquoise |
| ENSG00000134049 | IER3IP1 | turquoise |
| ENSG00000134058 | CDK7 | turquoise |
| ENSG00000134070 | IRAK2 | turquoise |
| ENSG00000134072 | CAMK1 | turquoise |
| ENSG00000134077 | THUMPD3 | turquoise |
| ENSG00000134107 | BHLHE40 | turquoise |
| ENSG00000134108 | ARL8B | turquoise |
| ENSG00000134109 | EDEM1 | turquoise |
| ENSG00000134146 | DPH6 | turquoise |
| ENSG00000134152 | KATNBL1 | turquoise |
| ENSG00000134186 | PRPF38B | turquoise |
| ENSG00000134215 | VAV3 | turquoise |
| ENSG00000134222 | PSRC1 | turquoise |
| ENSG00000134242 | PTPN22 | turquoise |
| ENSG00000134245 | WNT2B | turquoise |
| ENSG00000134248 | LAMTOR5 | turquoise |
| ENSG00000134250 | NOTCH2 | turquoise |
| ENSG00000134255 | CEPT1 | turquoise |
| ENSG00000134265 | NAPG | turquoise |
| ENSG00000134283 | PPHLN1 | turquoise |
| ENSG00000134285 | FKBP11 | turquoise |

| | | |
|---|---|---|
| ENSG00000134294 | SLC38A2 | turquoise |
| ENSG00000134297 | PLEKHA8P1 | turquoise |
| ENSG00000134308 | YWHAQ | turquoise |
| ENSG00000134313 | KIDINS220 | turquoise |
| ENSG00000134317 | GRHL1 | turquoise |
| ENSG00000134318 | ROCK2 | turquoise |
| ENSG00000134324 | LPIN1 | turquoise |
| ENSG00000134330 | IAH1 | turquoise |
| ENSG00000134352 | IL6ST | turquoise |
| ENSG00000134369 | NAV1 | turquoise |
| ENSG00000134371 | CDC73 | turquoise |
| ENSG00000134375 | TIMM17A | turquoise |
| ENSG00000134419 | RPS15A | turquoise |
| ENSG00000134440 | NARS | turquoise |
| ENSG00000134444 | KIAA1468 | turquoise |
| ENSG00000134452 | FBXO18 | turquoise |
| ENSG00000134453 | RBM17 | turquoise |
| ENSG00000134460 | IL2RA | turquoise |
| ENSG00000134461 | ANKRD16 | turquoise |
| ENSG00000134463 | ECHDC3 | turquoise |
| ENSG00000134480 | CCNH | turquoise |
| ENSG00000134489 | HRH4 | turquoise |
| ENSG00000134504 | KCTD1 | turquoise |
| ENSG00000134532 | SOX5 | turquoise |
| ENSG00000134545 | KLRC1 | turquoise |
| ENSG00000134548 | SPX | turquoise |
| ENSG00000134571 | MYBPC3 | turquoise |
| ENSG00000134574 | DDB2 | turquoise |
| ENSG00000134602 | STK26 | turquoise |
| ENSG00000134627 | PIWIL4 | turquoise |
| ENSG00000134644 | PUM1 | turquoise |
| ENSG00000134697 | GNL2 | turquoise |
| ENSG00000134698 | AGO4 | turquoise |
| ENSG00000134709 | HOOK1 | turquoise |
| ENSG00000134717 | BTF3L4 | turquoise |
| ENSG00000134744 | ZCCHC11 | turquoise |
| ENSG00000134748 | PRPF38A | turquoise |
| ENSG00000134755 | DSC2 | turquoise |
| ENSG00000134758 | RNF138 | turquoise |
| ENSG00000134759 | ELP2 | turquoise |
| ENSG00000134765 | DSC1 | turquoise |
| ENSG00000134769 | DTNA | turquoise |
| ENSG00000134780 | DAGLA | turquoise |
| ENSG00000134827 | TCN1 | turquoise |
| ENSG00000134851 | TMEM165 | turquoise |
| ENSG00000134852 | CLOCK | turquoise |
| ENSG00000134884 | ARGLU1 | turquoise |
| ENSG00000134900 | TPP2 | turquoise |

| | | |
|---|---|---|
| ENSG00000134909 | ARHGAP32 | turquoise |
| ENSG00000134954 | ETS1 | turquoise |
| ENSG00000134955 | SLC37A2 | turquoise |
| ENSG00000134970 | TMED7 | turquoise |
| ENSG00000134982 | APC | turquoise |
| ENSG00000134987 | WDR36 | turquoise |
| ENSG00000135002 | RFK | turquoise |
| ENSG00000135018 | UBQLN1 | turquoise |
| ENSG00000135040 | NAA35 | turquoise |
| ENSG00000135045 | C9orf40 | turquoise |
| ENSG00000135046 | ANXA1 | turquoise |
| ENSG00000135048 | TMEM2 | turquoise |
| ENSG00000135049 | AGTPBP1 | turquoise |
| ENSG00000135052 | GOLM1 | turquoise |
| ENSG00000135069 | PSAT1 | turquoise |
| ENSG00000135070 | ISCA1 | turquoise |
| ENSG00000135090 | TAOK3 | turquoise |
| ENSG00000135093 | USP30 | turquoise |
| ENSG00000135094 | SDS | turquoise |
| ENSG00000135108 | FBXO21 | turquoise |
| ENSG00000135119 | RNFT2 | turquoise |
| ENSG00000135124 | P2RX4 | turquoise |
| ENSG00000135164 | DMTF1 | turquoise |
| ENSG00000135185 | TMEM243 | turquoise |
| ENSG00000135205 | CCDC146 | turquoise |
| ENSG00000135241 | PNPLA8 | turquoise |
| ENSG00000135249 | RINT1 | turquoise |
| ENSG00000135250 | SRPK2 | turquoise |
| ENSG00000135253 | KCP | turquoise |
| ENSG00000135269 | TES | turquoise |
| ENSG00000135272 | MDFIC | turquoise |
| ENSG00000135297 | MTO1 | turquoise |
| ENSG00000135314 | KHDC1 | turquoise |
| ENSG00000135315 | CEP162 | turquoise |
| ENSG00000135316 | SYNCRIP | turquoise |
| ENSG00000135317 | SNX14 | turquoise |
| ENSG00000135334 | AKIRIN2 | turquoise |
| ENSG00000135336 | ORC3 | turquoise |
| ENSG00000135341 | MAP3K7 | turquoise |
| ENSG00000135387 | CAPRIN1 | turquoise |
| ENSG00000135392 | DNAJC14 | turquoise |
| ENSG00000135424 | ITGA7 | turquoise |
| ENSG00000135439 | AGAP2 | turquoise |
| ENSG00000135441 | BLOC1S1 | turquoise |
| ENSG00000135457 | TFCP2 | turquoise |
| ENSG00000135482 | ZC3H10 | turquoise |
| ENSG00000135503 | ACVR1B | turquoise |
| ENSG00000135519 | KCNH3 | turquoise |

| | | |
|---|---|---|
| ENSG00000135521 | LTV1 | turquoise |
| ENSG00000135525 | MAP7 | turquoise |
| ENSG00000135535 | CD164 | turquoise |
| ENSG00000135537 | AFG1L | turquoise |
| ENSG00000135541 | AHI1 | turquoise |
| ENSG00000135549 | PKIB | turquoise |
| ENSG00000135587 | SMPD2 | turquoise |
| ENSG00000135596 | MICAL1 | turquoise |
| ENSG00000135605 | TEC | turquoise |
| ENSG00000135617 | PRADC1 | turquoise |
| ENSG00000135632 | SMYD5 | turquoise |
| ENSG00000135637 | CCDC142 | turquoise |
| ENSG00000135655 | USP15 | turquoise |
| ENSG00000135678 | CPM | turquoise |
| ENSG00000135679 | MDM2 | turquoise |
| ENSG00000135698 | MPHOSPH6 | turquoise |
| ENSG00000135722 | FBXL8 | turquoise |
| ENSG00000135723 | FHOD1 | turquoise |
| ENSG00000135736 | CCDC102A | turquoise |
| ENSG00000135740 | SLC9A5 | turquoise |
| ENSG00000135763 | URB2 | turquoise |
| ENSG00000135766 | EGLN1 | turquoise |
| ENSG00000135776 | ABCB10 | turquoise |
| ENSG00000135778 | NTPCR | turquoise |
| ENSG00000135801 | TAF5L | turquoise |
| ENSG00000135823 | STX6 | turquoise |
| ENSG00000135828 | RNASEL | turquoise |
| ENSG00000135829 | DHX9 | turquoise |
| ENSG00000135837 | CEP350 | turquoise |
| ENSG00000135870 | RC3H1 | turquoise |
| ENSG00000135905 | DOCK10 | turquoise |
| ENSG00000135912 | TTLL4 | turquoise |
| ENSG00000135916 | ITM2C | turquoise |
| ENSG00000135929 | CYP27A1 | turquoise |
| ENSG00000135932 | CAB39 | turquoise |
| ENSG00000135945 | REV1 | turquoise |
| ENSG00000135951 | TSGA10 | turquoise |
| ENSG00000135960 | EDAR | turquoise |
| ENSG00000135966 | TGFBRAP1 | turquoise |
| ENSG00000135968 | GCC2 | turquoise |
| ENSG00000135974 | C2orf49 | turquoise |
| ENSG00000135999 | EPC2 | turquoise |
| ENSG00000136002 | ARHGEF4 | turquoise |
| ENSG00000136014 | USP44 | turquoise |
| ENSG00000136021 | SCYL2 | turquoise |
| ENSG00000136040 | PLXNC1 | turquoise |
| ENSG00000136048 | DRAM1 | turquoise |
| ENSG00000136051 | WASHC4 | turquoise |

| | | |
|---|---|---|
| ENSG00000136059 | VILL | turquoise |
| ENSG00000136068 | FLNB | turquoise |
| ENSG00000136100 | VPS36 | turquoise |
| ENSG00000136108 | CKAP2 | turquoise |
| ENSG00000136111 | TBC1D4 | turquoise |
| ENSG00000136114 | THSD1 | turquoise |
| ENSG00000136122 | BORA | turquoise |
| ENSG00000136141 | LRCH1 | turquoise |
| ENSG00000136143 | SUCLA2 | turquoise |
| ENSG00000136144 | RCBTB1 | turquoise |
| ENSG00000136146 | MED4 | turquoise |
| ENSG00000136152 | COG3 | turquoise |
| ENSG00000136153 | LMO7 | turquoise |
| ENSG00000136158 | SPRY2 | turquoise |
| ENSG00000136161 | RCBTB2 | turquoise |
| ENSG00000136169 | SETDB2 | turquoise |
| ENSG00000136237 | RAPGEF5 | turquoise |
| ENSG00000136243 | NUPL2 | turquoise |
| ENSG00000136247 | ZDHHC4 | turquoise |
| ENSG00000136270 | TBRG4 | turquoise |
| ENSG00000136271 | DDX56 | turquoise |
| ENSG00000136273 | HUS1 | turquoise |
| ENSG00000136279 | DBNL | turquoise |
| ENSG00000136280 | CCM2 | turquoise |
| ENSG00000136286 | MYO1G | turquoise |
| ENSG00000136315 | AL355922.1 | turquoise |
| ENSG00000136379 | ABHD17C | turquoise |
| ENSG00000136381 | IREB2 | turquoise |
| ENSG00000136425 | CIB2 | turquoise |
| ENSG00000136448 | NMT1 | turquoise |
| ENSG00000136450 | SRSF1 | turquoise |
| ENSG00000136451 | VEZF1 | turquoise |
| ENSG00000136457 | CHAD | turquoise |
| ENSG00000136478 | TEX2 | turquoise |
| ENSG00000136485 | DCAF7 | turquoise |
| ENSG00000136490 | LIMD2 | turquoise |
| ENSG00000136504 | KAT7 | turquoise |
| ENSG00000136521 | NDUFB5 | turquoise |
| ENSG00000136522 | MRPL47 | turquoise |
| ENSG00000136527 | TRA2B | turquoise |
| ENSG00000136536 | 7-Mar | turquoise |
| ENSG00000136560 | TANK | turquoise |
| ENSG00000136573 | BLK | turquoise |
| ENSG00000136603 | SKIL | turquoise |
| ENSG00000136628 | EPRS | turquoise |
| ENSG00000136636 | KCTD3 | turquoise |
| ENSG00000136643 | RPS6KC1 | turquoise |
| ENSG00000136682 | CBWD2 | turquoise |

| | | |
|---|---|---|
| ENSG00000136699 | SMPD4 | turquoise |
| ENSG00000136709 | WDR33 | turquoise |
| ENSG00000136720 | HS6ST1 | turquoise |
| ENSG00000136738 | STAM | turquoise |
| ENSG00000136754 | ABI1 | turquoise |
| ENSG00000136758 | YME1L1 | turquoise |
| ENSG00000136783 | NIPSNAP3A | turquoise |
| ENSG00000136802 | LRRC8A | turquoise |
| ENSG00000136807 | CDK9 | turquoise |
| ENSG00000136810 | TXN | turquoise |
| ENSG00000136813 | KIAA0368 | turquoise |
| ENSG00000136824 | SMC2 | turquoise |
| ENSG00000136854 | STXBP1 | turquoise |
| ENSG00000136861 | CDK5RAP2 | turquoise |
| ENSG00000136866 | ZFP37 | turquoise |
| ENSG00000136869 | TLR4 | turquoise |
| ENSG00000136870 | ZNF189 | turquoise |
| ENSG00000136875 | PRPF4 | turquoise |
| ENSG00000136888 | ATP6V1G1 | turquoise |
| ENSG00000136897 | MRPL50 | turquoise |
| ENSG00000136933 | RABEPK | turquoise |
| ENSG00000136936 | XPA | turquoise |
| ENSG00000136937 | NCBP1 | turquoise |
| ENSG00000136940 | PDCL | turquoise |
| ENSG00000136960 | ENPP2 | turquoise |
| ENSG00000136982 | DSCC1 | turquoise |
| ENSG00000136986 | DERL1 | turquoise |
| ENSG00000136999 | NOV | turquoise |
| ENSG00000137038 | DMAC1 | turquoise |
| ENSG00000137040 | RANBP6 | turquoise |
| ENSG00000137054 | POLR1E | turquoise |
| ENSG00000137055 | PLAA | turquoise |
| ENSG00000137070 | IL11RA | turquoise |
| ENSG00000137075 | RNF38 | turquoise |
| ENSG00000137098 | SPAG8 | turquoise |
| ENSG00000137124 | ALDH1B1 | turquoise |
| ENSG00000137135 | ARHGEF39 | turquoise |
| ENSG00000137145 | DENND4C | turquoise |
| ENSG00000137161 | CNPY3 | turquoise |
| ENSG00000137166 | FOXP4 | turquoise |
| ENSG00000137168 | PPIL1 | turquoise |
| ENSG00000137171 | KLC4 | turquoise |
| ENSG00000137177 | KIF13A | turquoise |
| ENSG00000137210 | TMEM14B | turquoise |
| ENSG00000137216 | TMEM63B | turquoise |
| ENSG00000137221 | TJAP1 | turquoise |
| ENSG00000137261 | KIAA0319 | turquoise |
| ENSG00000137269 | LRRC1 | turquoise |

| | | |
|---|---|---|
| ENSG00000137274 | BPHL | turquoise |
| ENSG00000137285 | TUBB2B | turquoise |
| ENSG00000137288 | UQCC2 | turquoise |
| ENSG00000137338 | PGBD1 | turquoise |
| ENSG00000137364 | TPMT | turquoise |
| ENSG00000137393 | RNF144B | turquoise |
| ENSG00000137409 | MTCH1 | turquoise |
| ENSG00000137413 | TAF8 | turquoise |
| ENSG00000137414 | FAM8A1 | turquoise |
| ENSG00000137449 | CPEB2 | turquoise |
| ENSG00000137460 | FHDC1 | turquoise |
| ENSG00000137474 | MYO7A | turquoise |
| ENSG00000137478 | FCHSD2 | turquoise |
| ENSG00000137486 | ARRB1 | turquoise |
| ENSG00000137492 | THAP12 | turquoise |
| ENSG00000137494 | ANKRD42 | turquoise |
| ENSG00000137496 | IL18BP | turquoise |
| ENSG00000137497 | NUMA1 | turquoise |
| ENSG00000137500 | CCDC90B | turquoise |
| ENSG00000137501 | SYTL2 | turquoise |
| ENSG00000137502 | RAB30 | turquoise |
| ENSG00000137504 | CREBZF | turquoise |
| ENSG00000137513 | NARS2 | turquoise |
| ENSG00000137522 | RNF121 | turquoise |
| ENSG00000137547 | MRPL15 | turquoise |
| ENSG00000137574 | TGS1 | turquoise |
| ENSG00000137575 | SDCBP | turquoise |
| ENSG00000137601 | NEK1 | turquoise |
| ENSG00000137642 | SORL1 | turquoise |
| ENSG00000137672 | TRPC6 | turquoise |
| ENSG00000137692 | DCUN1D5 | turquoise |
| ENSG00000137710 | RDX | turquoise |
| ENSG00000137713 | PPP2R1B | turquoise |
| ENSG00000137714 | FDX1 | turquoise |
| ENSG00000137720 | C11orf1 | turquoise |
| ENSG00000137726 | FXYD6 | turquoise |
| ENSG00000137747 | TMPRSS13 | turquoise |
| ENSG00000137760 | ALKBH8 | turquoise |
| ENSG00000137770 | CTDSPL2 | turquoise |
| ENSG00000137776 | SLTM | turquoise |
| ENSG00000137802 | MAPKBP1 | turquoise |
| ENSG00000137807 | KIF23 | turquoise |
| ENSG00000137812 | KNL1 | turquoise |
| ENSG00000137814 | HAUS2 | turquoise |
| ENSG00000137815 | RTF1 | turquoise |
| ENSG00000137817 | PARP6 | turquoise |
| ENSG00000137831 | UACA | turquoise |
| ENSG00000137841 | PLCB2 | turquoise |

| | | |
|---|---|---|
| ENSG00000137845 | ADAM10 | turquoise |
| ENSG00000137871 | ZNF280D | turquoise |
| ENSG00000137876 | RSL24D1 | turquoise |
| ENSG00000137877 | SPTBN5 | turquoise |
| ENSG00000137880 | GCHFR | turquoise |
| ENSG00000137941 | TTLL7 | turquoise |
| ENSG00000137942 | FNBP1L | turquoise |
| ENSG00000137944 | KYAT3 | turquoise |
| ENSG00000137947 | GTF2B | turquoise |
| ENSG00000137955 | RABGGTB | turquoise |
| ENSG00000137962 | ARHGAP29 | turquoise |
| ENSG00000137970 | RPL7P9 | turquoise |
| ENSG00000137992 | DBT | turquoise |
| ENSG00000137996 | RTCA | turquoise |
| ENSG00000138002 | IFT172 | turquoise |
| ENSG00000138018 | SELENOI | turquoise |
| ENSG00000138030 | KHK | turquoise |
| ENSG00000138032 | PPM1B | turquoise |
| ENSG00000138036 | DYNC2LI1 | turquoise |
| ENSG00000138050 | THUMPD2 | turquoise |
| ENSG00000138061 | CYP1B1 | turquoise |
| ENSG00000138069 | RAB1A | turquoise |
| ENSG00000138071 | ACTR2 | turquoise |
| ENSG00000138074 | SLC5A6 | turquoise |
| ENSG00000138078 | PREPL | turquoise |
| ENSG00000138079 | SLC3A1 | turquoise |
| ENSG00000138080 | EMILIN1 | turquoise |
| ENSG00000138092 | CENPO | turquoise |
| ENSG00000138095 | LRPPRC | turquoise |
| ENSG00000138101 | DTNB | turquoise |
| ENSG00000138107 | ACTR1A | turquoise |
| ENSG00000138111 | MFSD13A | turquoise |
| ENSG00000138134 | STAMBPL1 | turquoise |
| ENSG00000138138 | ATAD1 | turquoise |
| ENSG00000138160 | KIF11 | turquoise |
| ENSG00000138166 | DUSP5 | turquoise |
| ENSG00000138172 | CALHM2 | turquoise |
| ENSG00000138175 | ARL3 | turquoise |
| ENSG00000138180 | CEP55 | turquoise |
| ENSG00000138182 | KIF20B | turquoise |
| ENSG00000138190 | EXOC6 | turquoise |
| ENSG00000138193 | PLCE1 | turquoise |
| ENSG00000138231 | DBR1 | turquoise |
| ENSG00000138246 | DNAJC13 | turquoise |
| ENSG00000138286 | FAM149B1 | turquoise |
| ENSG00000138303 | ASCC1 | turquoise |
| ENSG00000138316 | ADAMTS14 | turquoise |
| ENSG00000138326 | RPS24 | turquoise |

| | | |
|---|---|---|
| ENSG00000138336 | TET1 | turquoise |
| ENSG00000138346 | DNA2 | turquoise |
| ENSG00000138376 | BARD1 | turquoise |
| ENSG00000138378 | STAT4 | turquoise |
| ENSG00000138380 | CARF | turquoise |
| ENSG00000138381 | ASNSD1 | turquoise |
| ENSG00000138382 | METTL5 | turquoise |
| ENSG00000138385 | SSB | turquoise |
| ENSG00000138386 | NAB1 | turquoise |
| ENSG00000138398 | PPIG | turquoise |
| ENSG00000138399 | FASTKD1 | turquoise |
| ENSG00000138411 | HECW2 | turquoise |
| ENSG00000138413 | IDH1 | turquoise |
| ENSG00000138430 | OLA1 | turquoise |
| ENSG00000138434 | SSFA2 | turquoise |
| ENSG00000138439 | FAM117B | turquoise |
| ENSG00000138443 | ABI2 | turquoise |
| ENSG00000138448 | ITGAV | turquoise |
| ENSG00000138449 | SLC40A1 | turquoise |
| ENSG00000138459 | SLC35A5 | turquoise |
| ENSG00000138468 | SENP7 | turquoise |
| ENSG00000138593 | SECISBP2L | turquoise |
| ENSG00000138594 | TMOD3 | turquoise |
| ENSG00000138600 | SPPL2A | turquoise |
| ENSG00000138604 | GLCE | turquoise |
| ENSG00000138606 | SHF | turquoise |
| ENSG00000138658 | ZGRF1 | turquoise |
| ENSG00000138660 | AP1AR | turquoise |
| ENSG00000138663 | COPS4 | turquoise |
| ENSG00000138668 | HNRNPD | turquoise |
| ENSG00000138669 | PRKG2 | turquoise |
| ENSG00000138670 | RASGEF1B | turquoise |
| ENSG00000138678 | GPAT3 | turquoise |
| ENSG00000138686 | BBS7 | turquoise |
| ENSG00000138688 | KIAA1109 | turquoise |
| ENSG00000138698 | RAP1GDS1 | turquoise |
| ENSG00000138709 | LARP1B | turquoise |
| ENSG00000138735 | PDE5A | turquoise |
| ENSG00000138738 | PRDM5 | turquoise |
| ENSG00000138750 | NUP54 | turquoise |
| ENSG00000138756 | BMP2K | turquoise |
| ENSG00000138757 | G3BP2 | turquoise |
| ENSG00000138758 | 11-Sep | turquoise |
| ENSG00000138764 | CCNG2 | turquoise |
| ENSG00000138767 | CNOT6L | turquoise |
| ENSG00000138768 | USO1 | turquoise |
| ENSG00000138772 | ANXA3 | turquoise |
| ENSG00000138777 | PPA2 | turquoise |

| | | |
|---|---|---|
| ENSG00000138778 | CENPE | turquoise |
| ENSG00000138780 | GSTCD | turquoise |
| ENSG00000138798 | EGF | turquoise |
| ENSG00000138801 | PAPSS1 | turquoise |
| ENSG00000138802 | SEC24B | turquoise |
| ENSG00000138814 | PPP3CA | turquoise |
| ENSG00000138821 | SLC39A8 | turquoise |
| ENSG00000138867 | GUCD1 | turquoise |
| ENSG00000138964 | PARVG | turquoise |
| ENSG00000139055 | ERP27 | turquoise |
| ENSG00000139112 | GABARAPL1 | turquoise |
| ENSG00000139116 | KIF21A | turquoise |
| ENSG00000139117 | CPNE8 | turquoise |
| ENSG00000139131 | YARS2 | turquoise |
| ENSG00000139133 | ALG10 | turquoise |
| ENSG00000139146 | FAM60A | turquoise |
| ENSG00000139154 | AEBP2 | turquoise |
| ENSG00000139163 | ETNK1 | turquoise |
| ENSG00000139168 | ZCRB1 | turquoise |
| ENSG00000139173 | TMEM117 | turquoise |
| ENSG00000139182 | CLSTN3 | turquoise |
| ENSG00000139187 | KLRG1 | turquoise |
| ENSG00000139190 | VAMP1 | turquoise |
| ENSG00000139192 | TAPBPL | turquoise |
| ENSG00000139197 | PEX5 | turquoise |
| ENSG00000139200 | PIANP | turquoise |
| ENSG00000139211 | AMIGO2 | turquoise |
| ENSG00000139218 | SCAF11 | turquoise |
| ENSG00000139233 | LLPH | turquoise |
| ENSG00000139269 | INHBE | turquoise |
| ENSG00000139289 | PHLDA1 | turquoise |
| ENSG00000139291 | TMEM19 | turquoise |
| ENSG00000139318 | DUSP6 | turquoise |
| ENSG00000139323 | POC1B | turquoise |
| ENSG00000139324 | TMTC3 | turquoise |
| ENSG00000139350 | NEDD1 | turquoise |
| ENSG00000139354 | GAS2L3 | turquoise |
| ENSG00000139370 | SLC15A4 | turquoise |
| ENSG00000139372 | TDG | turquoise |
| ENSG00000139410 | SDSL | turquoise |
| ENSG00000139428 | MMAB | turquoise |
| ENSG00000139437 | TCHP | turquoise |
| ENSG00000139496 | NUP58 | turquoise |
| ENSG00000139505 | MTMR6 | turquoise |
| ENSG00000139508 | SLC46A3 | turquoise |
| ENSG00000139514 | SLC7A1 | turquoise |
| ENSG00000139517 | LNX2 | turquoise |
| ENSG00000139531 | SUOX | turquoise |

| | | |
|---|---|---|
| ENSG00000139537 | CCDC65 | turquoise |
| ENSG00000139547 | RDH16 | turquoise |
| ENSG00000139567 | ACVRL1 | turquoise |
| ENSG00000139597 | N4BP2L1 | turquoise |
| ENSG00000139613 | SMARCC2 | turquoise |
| ENSG00000139620 | KANSL2 | turquoise |
| ENSG00000139626 | ITGB7 | turquoise |
| ENSG00000139629 | GALNT6 | turquoise |
| ENSG00000139636 | LMBR1L | turquoise |
| ENSG00000139645 | ANKRD52 | turquoise |
| ENSG00000139651 | ZNF740 | turquoise |
| ENSG00000139675 | HNRNPA1L2 | turquoise |
| ENSG00000139679 | LPAR6 | turquoise |
| ENSG00000139687 | RB1 | turquoise |
| ENSG00000139697 | SBNO1 | turquoise |
| ENSG00000139714 | MORN3 | turquoise |
| ENSG00000139719 | VPS33A | turquoise |
| ENSG00000139722 | VPS37B | turquoise |
| ENSG00000139725 | RHOF | turquoise |
| ENSG00000139726 | DENR | turquoise |
| ENSG00000139737 | SLAIN1 | turquoise |
| ENSG00000139746 | RBM26 | turquoise |
| ENSG00000139793 | MBNL2 | turquoise |
| ENSG00000139826 | ABHD13 | turquoise |
| ENSG00000139835 | GRTP1 | turquoise |
| ENSG00000139842 | CUL4A | turquoise |
| ENSG00000139921 | TMX1 | turquoise |
| ENSG00000139926 | FRMD6 | turquoise |
| ENSG00000139946 | PELI2 | turquoise |
| ENSG00000139970 | RTN1 | turquoise |
| ENSG00000139974 | SLC38A6 | turquoise |
| ENSG00000139977 | NAA30 | turquoise |
| ENSG00000139998 | RAB15 | turquoise |
| ENSG00000140006 | WDR89 | turquoise |
| ENSG00000140030 | GPR65 | turquoise |
| ENSG00000140044 | JDP2 | turquoise |
| ENSG00000140090 | SLC24A4 | turquoise |
| ENSG00000140104 | C14orf79 | turquoise |
| ENSG00000140153 | WDR20 | turquoise |
| ENSG00000140157 | NIPA2 | turquoise |
| ENSG00000140199 | SLC12A6 | turquoise |
| ENSG00000140259 | MFAP1 | turquoise |
| ENSG00000140262 | TCF12 | turquoise |
| ENSG00000140265 | ZSCAN29 | turquoise |
| ENSG00000140299 | BNIP2 | turquoise |
| ENSG00000140319 | SRP14 | turquoise |
| ENSG00000140320 | BAHD1 | turquoise |
| ENSG00000140326 | CDAN1 | turquoise |

| | | |
|---|---|---|
| ENSG00000140367 | UBE2Q2 | turquoise |
| ENSG00000140368 | PSTPIP1 | turquoise |
| ENSG00000140379 | BCL2A1 | turquoise |
| ENSG00000140386 | SCAPER | turquoise |
| ENSG00000140396 | NCOA2 | turquoise |
| ENSG00000140398 | NEIL1 | turquoise |
| ENSG00000140400 | MAN2C1 | turquoise |
| ENSG00000140403 | DNAJA4 | turquoise |
| ENSG00000140406 | TLNRD1 | turquoise |
| ENSG00000140450 | ARRDC4 | turquoise |
| ENSG00000140463 | BBS4 | turquoise |
| ENSG00000140471 | LINS1 | turquoise |
| ENSG00000140474 | ULK3 | turquoise |
| ENSG00000140521 | POLG | turquoise |
| ENSG00000140525 | FANCI | turquoise |
| ENSG00000140543 | DET1 | turquoise |
| ENSG00000140575 | IQGAP1 | turquoise |
| ENSG00000140598 | EFL1 | turquoise |
| ENSG00000140612 | SEC11A | turquoise |
| ENSG00000140632 | GLYR1 | turquoise |
| ENSG00000140682 | TGFB1I1 | turquoise |
| ENSG00000140694 | PARN | turquoise |
| ENSG00000140743 | CDR2 | turquoise |
| ENSG00000140829 | DHX38 | turquoise |
| ENSG00000140839 | CLEC18B | turquoise |
| ENSG00000140848 | CPNE2 | turquoise |
| ENSG00000140854 | KATNB1 | turquoise |
| ENSG00000140859 | KIFC3 | turquoise |
| ENSG00000140905 | GCSH | turquoise |
| ENSG00000140932 | CMTM2 | turquoise |
| ENSG00000140939 | NOL3 | turquoise |
| ENSG00000140943 | MBTPS1 | turquoise |
| ENSG00000140948 | ZCCHC14 | turquoise |
| ENSG00000140961 | OSGIN1 | turquoise |
| ENSG00000140983 | RHOT2 | turquoise |
| ENSG00000140986 | RPL3L | turquoise |
| ENSG00000140992 | PDPK1 | turquoise |
| ENSG00000141002 | TCF25 | turquoise |
| ENSG00000141027 | NCOR1 | turquoise |
| ENSG00000141096 | DPEP3 | turquoise |
| ENSG00000141098 | GFOD2 | turquoise |
| ENSG00000141179 | PCTP | turquoise |
| ENSG00000141194 | OR4D1 | turquoise |
| ENSG00000141219 | C17orf80 | turquoise |
| ENSG00000141232 | TOB1 | turquoise |
| ENSG00000141252 | VPS53 | turquoise |
| ENSG00000141258 | SGSM2 | turquoise |
| ENSG00000141279 | NPEPPS | turquoise |

| | | |
|---|---|---|
| ENSG00000141294 | LRRC46 | turquoise |
| ENSG00000141295 | SCRN2 | turquoise |
| ENSG00000141367 | CLTC | turquoise |
| ENSG00000141376 | BCAS3 | turquoise |
| ENSG00000141378 | PTRH2 | turquoise |
| ENSG00000141380 | SS18 | turquoise |
| ENSG00000141384 | TAF4B | turquoise |
| ENSG00000141385 | AFG3L2 | turquoise |
| ENSG00000141391 | PRELID3A | turquoise |
| ENSG00000141401 | IMPA2 | turquoise |
| ENSG00000141424 | SLC39A6 | turquoise |
| ENSG00000141425 | RPRD1A | turquoise |
| ENSG00000141428 | C18orf21 | turquoise |
| ENSG00000141429 | GALNT1 | turquoise |
| ENSG00000141446 | ESCO1 | turquoise |
| ENSG00000141447 | OSBPL1A | turquoise |
| ENSG00000141458 | NPC1 | turquoise |
| ENSG00000141469 | SLC14A1 | turquoise |
| ENSG00000141497 | ZMYND15 | turquoise |
| ENSG00000141503 | MINK1 | turquoise |
| ENSG00000141524 | TMC6 | turquoise |
| ENSG00000141540 | TTYH2 | turquoise |
| ENSG00000141568 | FOXK2 | turquoise |
| ENSG00000141570 | CBX8 | turquoise |
| ENSG00000141577 | CEP131 | turquoise |
| ENSG00000141580 | WDR45B | turquoise |
| ENSG00000141582 | CBX4 | turquoise |
| ENSG00000141642 | ELAC1 | turquoise |
| ENSG00000141646 | SMAD4 | turquoise |
| ENSG00000141682 | PMAIP1 | turquoise |
| ENSG00000141696 | P3H4 | turquoise |
| ENSG00000141698 | NT5C3B | turquoise |
| ENSG00000141744 | PNMT | turquoise |
| ENSG00000141873 | SLC39A3 | turquoise |
| ENSG00000141905 | NFIC | turquoise |
| ENSG00000141933 | TPGS1 | turquoise |
| ENSG00000141965 | FEM1A | turquoise |
| ENSG00000141971 | MVB12A | turquoise |
| ENSG00000141985 | SH3GL1 | turquoise |
| ENSG00000141994 | DUS3L | turquoise |
| ENSG00000142002 | DPP9 | turquoise |
| ENSG00000142025 | DMRTC2 | turquoise |
| ENSG00000142039 | CCDC97 | turquoise |
| ENSG00000142046 | TMEM91 | turquoise |
| ENSG00000142065 | ZFP14 | turquoise |
| ENSG00000142082 | SIRT3 | turquoise |
| ENSG00000142102 | PGGHG | turquoise |
| ENSG00000142166 | IFNAR1 | turquoise |

| | | |
|---|---|---|
| ENSG00000142173 | COL6A2 | turquoise |
| ENSG00000142178 | SIK1 | turquoise |
| ENSG00000142186 | SCYL1 | turquoise |
| ENSG00000142188 | TMEM50B | turquoise |
| ENSG00000142192 | APP | turquoise |
| ENSG00000142197 | DOPEY2 | turquoise |
| ENSG00000142207 | URB1 | turquoise |
| ENSG00000142279 | WTIP | turquoise |
| ENSG00000142303 | ADAMTS10 | turquoise |
| ENSG00000142330 | CAPN10 | turquoise |
| ENSG00000142396 | AC020915.1 | turquoise |
| ENSG00000142409 | ZNF787 | turquoise |
| ENSG00000142459 | EVI5L | turquoise |
| ENSG00000142512 | SIGLEC10 | turquoise |
| ENSG00000142528 | ZNF473 | turquoise |
| ENSG00000142544 | CTU1 | turquoise |
| ENSG00000142552 | RCN3 | turquoise |
| ENSG00000142556 | ZNF614 | turquoise |
| ENSG00000142621 | FHAD1 | turquoise |
| ENSG00000142627 | EPHA2 | turquoise |
| ENSG00000142632 | ARHGEF19 | turquoise |
| ENSG00000142669 | SH3BGRL3 | turquoise |
| ENSG00000142675 | CNKSR1 | turquoise |
| ENSG00000142676 | RPL11 | turquoise |
| ENSG00000142694 | EVA1B | turquoise |
| ENSG00000142731 | PLK4 | turquoise |
| ENSG00000142733 | MAP3K6 | turquoise |
| ENSG00000142751 | GPN2 | turquoise |
| ENSG00000142765 | SYTL1 | turquoise |
| ENSG00000142784 | WDTC1 | turquoise |
| ENSG00000142794 | NBPF3 | turquoise |
| ENSG00000142856 | ITGB3BP | turquoise |
| ENSG00000142867 | BCL10 | turquoise |
| ENSG00000142875 | PRKACB | turquoise |
| ENSG00000142892 | PIGK | turquoise |
| ENSG00000142959 | BEST4 | turquoise |
| ENSG00000143013 | LMO4 | turquoise |
| ENSG00000143033 | MTF2 | turquoise |
| ENSG00000143036 | SLC44A3 | turquoise |
| ENSG00000143067 | ZNF697 | turquoise |
| ENSG00000143079 | CTTNBP2NL | turquoise |
| ENSG00000143093 | STRIP1 | turquoise |
| ENSG00000143126 | CELSR2 | turquoise |
| ENSG00000143149 | ALDH9A1 | turquoise |
| ENSG00000143153 | ATP1B1 | turquoise |
| ENSG00000143155 | TIPRL | turquoise |
| ENSG00000143162 | CREG1 | turquoise |
| ENSG00000143164 | DCAF6 | turquoise |

| | | |
|---|---|---|
| ENSG00000143178 | TBX19 | turquoise |
| ENSG00000143183 | TMCO1 | turquoise |
| ENSG00000143184 | XCL1 | turquoise |
| ENSG00000143190 | POU2F1 | turquoise |
| ENSG00000143195 | ILDR2 | turquoise |
| ENSG00000143207 | RFWD2 | turquoise |
| ENSG00000143224 | PPOX | turquoise |
| ENSG00000143228 | NUF2 | turquoise |
| ENSG00000143248 | RGS5 | turquoise |
| ENSG00000143252 | SDHC | turquoise |
| ENSG00000143258 | USP21 | turquoise |
| ENSG00000143294 | PRCC | turquoise |
| ENSG00000143297 | FCRL5 | turquoise |
| ENSG00000143303 | RRNAD1 | turquoise |
| ENSG00000143318 | CASQ1 | turquoise |
| ENSG00000143320 | CRABP2 | turquoise |
| ENSG00000143322 | ABL2 | turquoise |
| ENSG00000143324 | XPR1 | turquoise |
| ENSG00000143337 | TOR1AIP1 | turquoise |
| ENSG00000143353 | LYPLAL1 | turquoise |
| ENSG00000143363 | PRUNE1 | turquoise |
| ENSG00000143365 | RORC | turquoise |
| ENSG00000143368 | SF3B4 | turquoise |
| ENSG00000143369 | ECM1 | turquoise |
| ENSG00000143374 | TARS2 | turquoise |
| ENSG00000143375 | CGN | turquoise |
| ENSG00000143382 | ADAMTSL4 | turquoise |
| ENSG00000143384 | MCL1 | turquoise |
| ENSG00000143387 | CTSK | turquoise |
| ENSG00000143393 | PI4KB | turquoise |
| ENSG00000143398 | PIP5K1A | turquoise |
| ENSG00000143401 | ANP32E | turquoise |
| ENSG00000143434 | SEMA6C | turquoise |
| ENSG00000143437 | ARNT | turquoise |
| ENSG00000143442 | POGZ | turquoise |
| ENSG00000143443 | C1orf56 | turquoise |
| ENSG00000143457 | GOLPH3L | turquoise |
| ENSG00000143458 | GABPB2 | turquoise |
| ENSG00000143493 | INTS7 | turquoise |
| ENSG00000143494 | VASH2 | turquoise |
| ENSG00000143498 | TAF1A | turquoise |
| ENSG00000143499 | SMYD2 | turquoise |
| ENSG00000143502 | SUSD4 | turquoise |
| ENSG00000143512 | HHIPL2 | turquoise |
| ENSG00000143514 | TP53BP2 | turquoise |
| ENSG00000143515 | ATP8B2 | turquoise |
| ENSG00000143537 | ADAM15 | turquoise |
| ENSG00000143546 | S100A8 | turquoise |

| | | |
|---|---|---|
| ENSG00000143578 | CREB3L4 | turquoise |
| ENSG00000143612 | C1orf43 | turquoise |
| ENSG00000143622 | RIT1 | turquoise |
| ENSG00000143624 | INTS3 | turquoise |
| ENSG00000143633 | C1orf131 | turquoise |
| ENSG00000143643 | TTC13 | turquoise |
| ENSG00000143669 | LYST | turquoise |
| ENSG00000143674 | MAP3K21 | turquoise |
| ENSG00000143727 | ACP1 | turquoise |
| ENSG00000143740 | SNAP47 | turquoise |
| ENSG00000143742 | SRP9 | turquoise |
| ENSG00000143748 | NVL | turquoise |
| ENSG00000143751 | SDE2 | turquoise |
| ENSG00000143756 | FBXO28 | turquoise |
| ENSG00000143771 | CNIH4 | turquoise |
| ENSG00000143776 | CDC42BPA | turquoise |
| ENSG00000143797 | MBOAT2 | turquoise |
| ENSG00000143815 | LBR | turquoise |
| ENSG00000143819 | EPHX1 | turquoise |
| ENSG00000143845 | ETNK2 | turquoise |
| ENSG00000143851 | PTPN7 | turquoise |
| ENSG00000143870 | PDIA6 | turquoise |
| ENSG00000143882 | ATP6V1C2 | turquoise |
| ENSG00000143889 | HNRNPLL | turquoise |
| ENSG00000143924 | EML4 | turquoise |
| ENSG00000143933 | CALM2 | turquoise |
| ENSG00000143942 | CHAC2 | turquoise |
| ENSG00000143952 | VPS54 | turquoise |
| ENSG00000143970 | ASXL2 | turquoise |
| ENSG00000143971 | ETAA1 | turquoise |
| ENSG00000143977 | SNRPG | turquoise |
| ENSG00000143994 | ABHD1 | turquoise |
| ENSG00000143995 | MEIS1 | turquoise |
| ENSG00000144036 | EXOC6B | turquoise |
| ENSG00000144040 | SFXN5 | turquoise |
| ENSG00000144043 | TEX261 | turquoise |
| ENSG00000144045 | DQX1 | turquoise |
| ENSG00000144048 | DUSP11 | turquoise |
| ENSG00000144115 | THNSL2 | turquoise |
| ENSG00000144134 | RABL2A | turquoise |
| ENSG00000144136 | SLC20A1 | turquoise |
| ENSG00000144161 | ZC3H8 | turquoise |
| ENSG00000144182 | LIPT1 | turquoise |
| ENSG00000144199 | FAHD2B | turquoise |
| ENSG00000144214 | LYG1 | turquoise |
| ENSG00000144218 | AFF3 | turquoise |
| ENSG00000144224 | UBXN4 | turquoise |
| ENSG00000144228 | SPOPL | turquoise |

| | | |
|---|---|---|
| ENSG00000144233 | AMMECR1L | turquoise |
| ENSG00000144306 | SCRN3 | turquoise |
| ENSG00000144320 | LNPK | turquoise |
| ENSG00000144354 | CDCA7 | turquoise |
| ENSG00000144357 | UBR3 | turquoise |
| ENSG00000144381 | HSPD1 | turquoise |
| ENSG00000144395 | CCDC150 | turquoise |
| ENSG00000144407 | PTH2R | turquoise |
| ENSG00000144451 | SPAG16 | turquoise |
| ENSG00000144476 | ACKR3 | turquoise |
| ENSG00000144488 | ESPNL | turquoise |
| ENSG00000144504 | ANKMY1 | turquoise |
| ENSG00000144535 | DIS3L2 | turquoise |
| ENSG00000144550 | CPNE9 | turquoise |
| ENSG00000144560 | VGLL4 | turquoise |
| ENSG00000144566 | RAB5A | turquoise |
| ENSG00000144579 | CTDSP1 | turquoise |
| ENSG00000144589 | STK11IP | turquoise |
| ENSG00000144597 | EAF1 | turquoise |
| ENSG00000144642 | RBMS3 | turquoise |
| ENSG00000144649 | FAM198A | turquoise |
| ENSG00000144668 | ITGA9 | turquoise |
| ENSG00000144671 | SLC22A14 | turquoise |
| ENSG00000144674 | GOLGA4 | turquoise |
| ENSG00000144712 | CAND2 | turquoise |
| ENSG00000144736 | SHQ1 | turquoise |
| ENSG00000144744 | UBA3 | turquoise |
| ENSG00000144746 | ARL6IP5 | turquoise |
| ENSG00000144747 | TMF1 | turquoise |
| ENSG00000144749 | LRIG1 | turquoise |
| ENSG00000144802 | NFKBIZ | turquoise |
| ENSG00000144815 | NXPE3 | turquoise |
| ENSG00000144824 | PHLDB2 | turquoise |
| ENSG00000144827 | ABHD10 | turquoise |
| ENSG00000144840 | RABL3 | turquoise |
| ENSG00000144848 | ATG3 | turquoise |
| ENSG00000144852 | NR1I2 | turquoise |
| ENSG00000144868 | TMEM108 | turquoise |
| ENSG00000144893 | MED12L | turquoise |
| ENSG00000144895 | EIF2A | turquoise |
| ENSG00000144909 | OSBPL11 | turquoise |
| ENSG00000144935 | TRPC1 | turquoise |
| ENSG00000144959 | NCEH1 | turquoise |
| ENSG00000145002 | FAM86B2 | turquoise |
| ENSG00000145014 | TMEM44 | turquoise |
| ENSG00000145020 | AMT | turquoise |
| ENSG00000145022 | TCTA | turquoise |
| ENSG00000145041 | DCAF1 | turquoise |

| | | |
|---|---|---|
| ENSG00000145075 | CCDC39 | turquoise |
| ENSG00000145088 | EAF2 | turquoise |
| ENSG00000145241 | CENPC | turquoise |
| ENSG00000145244 | CORIN | turquoise |
| ENSG00000145246 | ATP10D | turquoise |
| ENSG00000145284 | SCD5 | turquoise |
| ENSG00000145293 | ENOPH1 | turquoise |
| ENSG00000145331 | TRMT10A | turquoise |
| ENSG00000145332 | KLHL8 | turquoise |
| ENSG00000145348 | TBCK | turquoise |
| ENSG00000145349 | CAMK2D | turquoise |
| ENSG00000145354 | CISD2 | turquoise |
| ENSG00000145365 | TIFA | turquoise |
| ENSG00000145375 | SPATA5 | turquoise |
| ENSG00000145386 | CCNA2 | turquoise |
| ENSG00000145388 | METTL14 | turquoise |
| ENSG00000145390 | USP53 | turquoise |
| ENSG00000145391 | SETD7 | turquoise |
| ENSG00000145414 | NAF1 | turquoise |
| ENSG00000145416 | 1-Mar | turquoise |
| ENSG00000145425 | RPS3A | turquoise |
| ENSG00000145428 | RNF175 | turquoise |
| ENSG00000145431 | PDGFC | turquoise |
| ENSG00000145439 | CBR4 | turquoise |
| ENSG00000145476 | CYP4V2 | turquoise |
| ENSG00000145495 | 6-Mar | turquoise |
| ENSG00000145569 | FAM105A | turquoise |
| ENSG00000145604 | SKP2 | turquoise |
| ENSG00000145632 | PLK2 | turquoise |
| ENSG00000145675 | PIK3R1 | turquoise |
| ENSG00000145687 | SSBP2 | turquoise |
| ENSG00000145700 | ANKRD31 | turquoise |
| ENSG00000145703 | IQGAP2 | turquoise |
| ENSG00000145708 | CRHBP | turquoise |
| ENSG00000145715 | RASA1 | turquoise |
| ENSG00000145721 | LIX1 | turquoise |
| ENSG00000145723 | GIN1 | turquoise |
| ENSG00000145725 | PPIP5K2 | turquoise |
| ENSG00000145730 | PAM | turquoise |
| ENSG00000145736 | GTF2H2 | turquoise |
| ENSG00000145740 | SLC30A5 | turquoise |
| ENSG00000145741 | BTF3 | turquoise |
| ENSG00000145743 | FBXL17 | turquoise |
| ENSG00000145779 | TNFAIP8 | turquoise |
| ENSG00000145780 | FEM1C | turquoise |
| ENSG00000145781 | COMMD10 | turquoise |
| ENSG00000145782 | ATG12 | turquoise |
| ENSG00000145817 | YIPF5 | turquoise |

| | | |
|---|---|---|
| ENSG00000145833 | DDX46 | turquoise |
| ENSG00000145860 | RNF145 | turquoise |
| ENSG00000145868 | FBXO38 | turquoise |
| ENSG00000145882 | PCYOX1L | turquoise |
| ENSG00000145901 | TNIP1 | turquoise |
| ENSG00000145907 | G3BP1 | turquoise |
| ENSG00000145916 | RMND5B | turquoise |
| ENSG00000145919 | BOD1 | turquoise |
| ENSG00000145936 | KCNMB1 | turquoise |
| ENSG00000145949 | MYLK4 | turquoise |
| ENSG00000145979 | TBC1D7 | turquoise |
| ENSG00000146007 | ZMAT2 | turquoise |
| ENSG00000146021 | KLHL3 | turquoise |
| ENSG00000146054 | TRIM7 | turquoise |
| ENSG00000146063 | TRIM41 | turquoise |
| ENSG00000146067 | FAM193B | turquoise |
| ENSG00000146083 | RNF44 | turquoise |
| ENSG00000146085 | MUT | turquoise |
| ENSG00000146122 | DAAM2 | turquoise |
| ENSG00000146166 | LGSN | turquoise |
| ENSG00000146192 | FGD2 | turquoise |
| ENSG00000146223 | RPL7L1 | turquoise |
| ENSG00000146247 | PHIP | turquoise |
| ENSG00000146250 | PRSS35 | turquoise |
| ENSG00000146263 | MMS22L | turquoise |
| ENSG00000146278 | PNRC1 | turquoise |
| ENSG00000146281 | PM20D2 | turquoise |
| ENSG00000146282 | RARS2 | turquoise |
| ENSG00000146285 | SCML4 | turquoise |
| ENSG00000146350 | TBC1D32 | turquoise |
| ENSG00000146373 | RNF217 | turquoise |
| ENSG00000146376 | ARHGAP18 | turquoise |
| ENSG00000146386 | ABRACL | turquoise |
| ENSG00000146409 | SLC18B1 | turquoise |
| ENSG00000146410 | MTFR2 | turquoise |
| ENSG00000146414 | SHPRH | turquoise |
| ENSG00000146426 | TIAM2 | turquoise |
| ENSG00000146433 | TMEM181 | turquoise |
| ENSG00000146453 | PNLDC1 | turquoise |
| ENSG00000146457 | WTAP | turquoise |
| ENSG00000146463 | ZMYM4 | turquoise |
| ENSG00000146476 | ARMT1 | turquoise |
| ENSG00000146540 | C7orf50 | turquoise |
| ENSG00000146556 | WASH2P | turquoise |
| ENSG00000146574 | CCZ1B | turquoise |
| ENSG00000146576 | C7orf26 | turquoise |
| ENSG00000146587 | RBAK | turquoise |
| ENSG00000146592 | CREB5 | turquoise |

| | | |
|---|---|---|
| ENSG00000146674 | IGFBP3 | turquoise |
| ENSG00000146677 | AC004453.1 | turquoise |
| ENSG00000146700 | SSC4D | turquoise |
| ENSG00000146729 | NIPSNAP2 | turquoise |
| ENSG00000146731 | CCT6A | turquoise |
| ENSG00000146757 | ZNF92 | turquoise |
| ENSG00000146802 | TMEM168 | turquoise |
| ENSG00000146826 | C7orf43 | turquoise |
| ENSG00000146830 | GIGYF1 | turquoise |
| ENSG00000146833 | TRIM4 | turquoise |
| ENSG00000146842 | TMEM209 | turquoise |
| ENSG00000146856 | AGBL3 | turquoise |
| ENSG00000146872 | TLK2 | turquoise |
| ENSG00000146909 | NOM1 | turquoise |
| ENSG00000146918 | NCAPG2 | turquoise |
| ENSG00000146963 | LUC7L2 | turquoise |
| ENSG00000147036 | LANCL3 | turquoise |
| ENSG00000147050 | KDM6A | turquoise |
| ENSG00000147082 | CCNB3 | turquoise |
| ENSG00000147118 | ZNF182 | turquoise |
| ENSG00000147121 | KRBOX4 | turquoise |
| ENSG00000147124 | ZNF41 | turquoise |
| ENSG00000147133 | TAF1 | turquoise |
| ENSG00000147138 | GPR174 | turquoise |
| ENSG00000147162 | OGT | turquoise |
| ENSG00000147166 | ITGB1BP2 | turquoise |
| ENSG00000147174 | GCNA | turquoise |
| ENSG00000147180 | ZNF711 | turquoise |
| ENSG00000147231 | CXorf57 | turquoise |
| ENSG00000147234 | FRMPD3 | turquoise |
| ENSG00000147251 | DOCK11 | turquoise |
| ENSG00000147274 | RBMX | turquoise |
| ENSG00000147316 | MCPH1 | turquoise |
| ENSG00000147394 | ZNF185 | turquoise |
| ENSG00000147408 | CSGALNACT1 | turquoise |
| ENSG00000147419 | CCDC25 | turquoise |
| ENSG00000147421 | HMBOX1 | turquoise |
| ENSG00000147437 | GNRH1 | turquoise |
| ENSG00000147459 | DOCK5 | turquoise |
| ENSG00000147471 | PLPBP | turquoise |
| ENSG00000147475 | ERLIN2 | turquoise |
| ENSG00000147533 | GOLGA7 | turquoise |
| ENSG00000147535 | PLPP5 | turquoise |
| ENSG00000147548 | NSD3 | turquoise |
| ENSG00000147570 | DNAJC5B | turquoise |
| ENSG00000147576 | ADHFE1 | turquoise |
| ENSG00000147592 | LACTB2 | turquoise |
| ENSG00000147601 | TERF1 | turquoise |

| | | |
|---|---|---|
| ENSG00000147604 | RPL7 | turquoise |
| ENSG00000147649 | MTDH | turquoise |
| ENSG00000147650 | LRP12 | turquoise |
| ENSG00000147654 | EBAG9 | turquoise |
| ENSG00000147669 | POLR2K | turquoise |
| ENSG00000147679 | UTP23 | turquoise |
| ENSG00000147687 | TATDN1 | turquoise |
| ENSG00000147789 | ZNF7 | turquoise |
| ENSG00000147804 | SLC39A4 | turquoise |
| ENSG00000147813 | NAPRT | turquoise |
| ENSG00000147852 | VLDLR | turquoise |
| ENSG00000147853 | AK3 | turquoise |
| ENSG00000147854 | UHRF2 | turquoise |
| ENSG00000147874 | HAUS6 | turquoise |
| ENSG00000147883 | CDKN2B | turquoise |
| ENSG00000147889 | CDKN2A | turquoise |
| ENSG00000147894 | C9orf72 | turquoise |
| ENSG00000147905 | ZCCHC7 | turquoise |
| ENSG00000147912 | FBXO10 | turquoise |
| ENSG00000147955 | SIGMAR1 | turquoise |
| ENSG00000147996 | CBWD5 | turquoise |
| ENSG00000148082 | SHC3 | turquoise |
| ENSG00000148090 | AUH | turquoise |
| ENSG00000148110 | MFSD14B | turquoise |
| ENSG00000148153 | INIP | turquoise |
| ENSG00000148154 | UGCG | turquoise |
| ENSG00000148204 | CRB2 | turquoise |
| ENSG00000148288 | GBGT1 | turquoise |
| ENSG00000148331 | ASB6 | turquoise |
| ENSG00000148334 | PTGES2 | turquoise |
| ENSG00000148335 | NTMT1 | turquoise |
| ENSG00000148337 | CIZ1 | turquoise |
| ENSG00000148339 | SLC25A25 | turquoise |
| ENSG00000148341 | SH3GLB2 | turquoise |
| ENSG00000148343 | MIGA2 | turquoise |
| ENSG00000148344 | PTGES | turquoise |
| ENSG00000148356 | LRSAM1 | turquoise |
| ENSG00000148358 | GPR107 | turquoise |
| ENSG00000148399 | DPH7 | turquoise |
| ENSG00000148429 | USP6NL | turquoise |
| ENSG00000148444 | COMMD3 | turquoise |
| ENSG00000148459 | PDSS1 | turquoise |
| ENSG00000148481 | MINDY3 | turquoise |
| ENSG00000148483 | TMEM236 | turquoise |
| ENSG00000148488 | ST8SIA6 | turquoise |
| ENSG00000148572 | NRBF2 | turquoise |
| ENSG00000148634 | HERC4 | turquoise |
| ENSG00000148655 | LRMDA | turquoise |

| | | |
|---|---|---|
| ENSG00000148672 | GLUD1 | turquoise |
| ENSG00000148680 | HTR7 | turquoise |
| ENSG00000148690 | FRA10AC1 | turquoise |
| ENSG00000148700 | ADD3 | turquoise |
| ENSG00000148719 | DNAJB12 | turquoise |
| ENSG00000148737 | TCF7L2 | turquoise |
| ENSG00000148814 | LRRC27 | turquoise |
| ENSG00000148824 | MTG1 | turquoise |
| ENSG00000148832 | PAOX | turquoise |
| ENSG00000148835 | TAF5 | turquoise |
| ENSG00000148842 | CNNM2 | turquoise |
| ENSG00000148925 | BTBD10 | turquoise |
| ENSG00000148926 | ADM | turquoise |
| ENSG00000148935 | GAS2 | turquoise |
| ENSG00000148943 | LIN7C | turquoise |
| ENSG00000148950 | IMMP1L | turquoise |
| ENSG00000148985 | PGAP2 | turquoise |
| ENSG00000149084 | HSD17B12 | turquoise |
| ENSG00000149089 | APIP | turquoise |
| ENSG00000149091 | DGKZ | turquoise |
| ENSG00000149179 | C11orf49 | turquoise |
| ENSG00000149212 | SESN3 | turquoise |
| ENSG00000149218 | ENDOD1 | turquoise |
| ENSG00000149231 | CCDC82 | turquoise |
| ENSG00000149243 | KLHL35 | turquoise |
| ENSG00000149257 | SERPINH1 | turquoise |
| ENSG00000149262 | INTS4 | turquoise |
| ENSG00000149308 | NPAT | turquoise |
| ENSG00000149311 | ATM | turquoise |
| ENSG00000149313 | AASDHPPT | turquoise |
| ENSG00000149346 | SLX4IP | turquoise |
| ENSG00000149403 | GRIK4 | turquoise |
| ENSG00000149483 | TMEM138 | turquoise |
| ENSG00000149488 | TMC2 | turquoise |
| ENSG00000149489 | ROM1 | turquoise |
| ENSG00000149499 | EML3 | turquoise |
| ENSG00000149503 | INCENP | turquoise |
| ENSG00000149506 | ZP1 | turquoise |
| ENSG00000149516 | MS4A3 | turquoise |
| ENSG00000149548 | CCDC15 | turquoise |
| ENSG00000149573 | MPZL2 | turquoise |
| ENSG00000149591 | TAGLN | turquoise |
| ENSG00000149636 | DSN1 | turquoise |
| ENSG00000149639 | SOGA1 | turquoise |
| ENSG00000149657 | LSM14B | turquoise |
| ENSG00000149658 | YTHDF1 | turquoise |
| ENSG00000149716 | ORAOV1 | turquoise |
| ENSG00000149743 | TRPT1 | turquoise |

| | | |
|---|---|---|
| ENSG00000149761 | NUDT22 | turquoise |
| ENSG00000149782 | PLCB3 | turquoise |
| ENSG00000149798 | CDC42EP2 | turquoise |
| ENSG00000149922 | TBX6 | turquoise |
| ENSG00000149923 | PPP4C | turquoise |
| ENSG00000149925 | ALDOA | turquoise |
| ENSG00000149927 | DOC2A | turquoise |
| ENSG00000149930 | TAOK2 | turquoise |
| ENSG00000149970 | CNKSR2 | turquoise |
| ENSG00000150054 | MPP7 | turquoise |
| ENSG00000150093 | ITGB1 | turquoise |
| ENSG00000150281 | CTF1 | turquoise |
| ENSG00000150316 | CWC15 | turquoise |
| ENSG00000150347 | ARID5B | turquoise |
| ENSG00000150433 | TMEM218 | turquoise |
| ENSG00000150457 | LATS2 | turquoise |
| ENSG00000150540 | HNMT | turquoise |
| ENSG00000150593 | PDCD4 | turquoise |
| ENSG00000150627 | WDR17 | turquoise |
| ENSG00000150636 | CCDC102B | turquoise |
| ENSG00000150637 | CD226 | turquoise |
| ENSG00000150667 | FSIP1 | turquoise |
| ENSG00000150672 | DLG2 | turquoise |
| ENSG00000150681 | RGS18 | turquoise |
| ENSG00000150712 | MTMR12 | turquoise |
| ENSG00000150756 | FAM173B | turquoise |
| ENSG00000150760 | DOCK1 | turquoise |
| ENSG00000150764 | DIXDC1 | turquoise |
| ENSG00000150768 | DLAT | turquoise |
| ENSG00000150776 | C11orf57 | turquoise |
| ENSG00000150779 | TIMM8B | turquoise |
| ENSG00000150782 | IL18 | turquoise |
| ENSG00000150787 | PTS | turquoise |
| ENSG00000150907 | FOXO1 | turquoise |
| ENSG00000150938 | CRIM1 | turquoise |
| ENSG00000150967 | ABCB9 | turquoise |
| ENSG00000150977 | RILPL2 | turquoise |
| ENSG00000150991 | UBC | turquoise |
| ENSG00000150995 | ITPR1 | turquoise |
| ENSG00000151012 | SLC7A11 | turquoise |
| ENSG00000151014 | NOCT | turquoise |
| ENSG00000151092 | NGLY1 | turquoise |
| ENSG00000151093 | OXSM | turquoise |
| ENSG00000151116 | UEVLD | turquoise |
| ENSG00000151117 | TMEM86A | turquoise |
| ENSG00000151131 | C12orf45 | turquoise |
| ENSG00000151135 | TMEM263 | turquoise |
| ENSG00000151136 | BTBD11 | turquoise |

| | | |
|---|---|---|
| ENSG00000151150 | ANK3 | turquoise |
| ENSG00000151151 | IPMK | turquoise |
| ENSG00000151164 | RAD9B | turquoise |
| ENSG00000151176 | PLBD2 | turquoise |
| ENSG00000151229 | SLC2A13 | turquoise |
| ENSG00000151233 | GXYLT1 | turquoise |
| ENSG00000151239 | TWF1 | turquoise |
| ENSG00000151247 | EIF4E | turquoise |
| ENSG00000151287 | TEX30 | turquoise |
| ENSG00000151292 | CSNK1G3 | turquoise |
| ENSG00000151304 | SRFBP1 | turquoise |
| ENSG00000151332 | MBIP | turquoise |
| ENSG00000151348 | EXT2 | turquoise |
| ENSG00000151353 | TMEM18 | turquoise |
| ENSG00000151376 | ME3 | turquoise |
| ENSG00000151413 | NUBPL | turquoise |
| ENSG00000151414 | NEK7 | turquoise |
| ENSG00000151422 | FER | turquoise |
| ENSG00000151445 | VIPAS39 | turquoise |
| ENSG00000151458 | ANKRD50 | turquoise |
| ENSG00000151461 | UPF2 | turquoise |
| ENSG00000151465 | CDC123 | turquoise |
| ENSG00000151466 | SCLT1 | turquoise |
| ENSG00000151470 | C4orf33 | turquoise |
| ENSG00000151490 | PTPRO | turquoise |
| ENSG00000151491 | EPS8 | turquoise |
| ENSG00000151502 | VPS26B | turquoise |
| ENSG00000151503 | NCAPD3 | turquoise |
| ENSG00000151553 | FAM160B1 | turquoise |
| ENSG00000151576 | QTRT2 | turquoise |
| ENSG00000151611 | MMAA | turquoise |
| ENSG00000151655 | ITIH2 | turquoise |
| ENSG00000151657 | KIN | turquoise |
| ENSG00000151665 | PIGF | turquoise |
| ENSG00000151687 | ANKAR | turquoise |
| ENSG00000151690 | MFSD6 | turquoise |
| ENSG00000151692 | RNF144A | turquoise |
| ENSG00000151702 | FLI1 | turquoise |
| ENSG00000151718 | WWC2 | turquoise |
| ENSG00000151725 | CENPU | turquoise |
| ENSG00000151726 | ACSL1 | turquoise |
| ENSG00000151746 | BICD1 | turquoise |
| ENSG00000151748 | SAV1 | turquoise |
| ENSG00000151835 | SACS | turquoise |
| ENSG00000151846 | PABPC3 | turquoise |
| ENSG00000151849 | CENPJ | turquoise |
| ENSG00000151881 | TMEM267 | turquoise |
| ENSG00000151883 | PARP8 | turquoise |

| | | |
|---|---|---|
| ENSG00000151893 | CACUL1 | turquoise |
| ENSG00000151914 | DST | turquoise |
| ENSG00000151923 | TIAL1 | turquoise |
| ENSG00000151952 | TMEM132D | turquoise |
| ENSG00000152061 | RABGAP1L | turquoise |
| ENSG00000152102 | FAM168B | turquoise |
| ENSG00000152117 | AC073869.1 | turquoise |
| ENSG00000152133 | GPATCH11 | turquoise |
| ENSG00000152193 | RNF219 | turquoise |
| ENSG00000152207 | CYSLTR2 | turquoise |
| ENSG00000152217 | SETBP1 | turquoise |
| ENSG00000152219 | ARL14EP | turquoise |
| ENSG00000152223 | EPG5 | turquoise |
| ENSG00000152240 | HAUS1 | turquoise |
| ENSG00000152270 | PDE3B | turquoise |
| ENSG00000152284 | TCF7L1 | turquoise |
| ENSG00000152291 | TGOLN2 | turquoise |
| ENSG00000152332 | UHMK1 | turquoise |
| ENSG00000152348 | ATG10 | turquoise |
| ENSG00000152359 | POC5 | turquoise |
| ENSG00000152380 | FAM151B | turquoise |
| ENSG00000152382 | TADA1 | turquoise |
| ENSG00000152404 | CWF19L2 | turquoise |
| ENSG00000152409 | JMY | turquoise |
| ENSG00000152422 | XRCC4 | turquoise |
| ENSG00000152454 | ZNF256 | turquoise |
| ENSG00000152464 | RPP38 | turquoise |
| ENSG00000152465 | NMT2 | turquoise |
| ENSG00000152475 | ZNF837 | turquoise |
| ENSG00000152484 | USP12 | turquoise |
| ENSG00000152492 | CCDC50 | turquoise |
| ENSG00000152495 | CAMK4 | turquoise |
| ENSG00000152518 | ZFP36L2 | turquoise |
| ENSG00000152520 | PAN3 | turquoise |
| ENSG00000152556 | PFKM | turquoise |
| ENSG00000152558 | TMEM123 | turquoise |
| ENSG00000152582 | SPEF2 | turquoise |
| ENSG00000152601 | MBNL1 | turquoise |
| ENSG00000152620 | NADK2 | turquoise |
| ENSG00000152642 | GPD1L | turquoise |
| ENSG00000152672 | CLEC4F | turquoise |
| ENSG00000152683 | SLC30A6 | turquoise |
| ENSG00000152684 | PELO | turquoise |
| ENSG00000152689 | RASGRP3 | turquoise |
| ENSG00000152700 | SAR1B | turquoise |
| ENSG00000152705 | CATSPER3 | turquoise |
| ENSG00000152749 | GPR180 | turquoise |
| ENSG00000152763 | WDR78 | turquoise |

| | | |
|---|---|---|
| ENSG00000152795 | HNRNPDL | turquoise |
| ENSG00000152804 | HHEX | turquoise |
| ENSG00000152818 | UTRN | turquoise |
| ENSG00000152904 | GGPS1 | turquoise |
| ENSG00000152926 | ZNF117 | turquoise |
| ENSG00000152932 | RAB3C | turquoise |
| ENSG00000152942 | RAD17 | turquoise |
| ENSG00000152944 | MED21 | turquoise |
| ENSG00000152969 | JAKMIP1 | turquoise |
| ENSG00000152990 | ADGRA3 | turquoise |
| ENSG00000153006 | SREK1IP1 | turquoise |
| ENSG00000153015 | CWC27 | turquoise |
| ENSG00000153037 | SRP19 | turquoise |
| ENSG00000153046 | CDYL | turquoise |
| ENSG00000153048 | CARHSP1 | turquoise |
| ENSG00000153094 | BCL2L11 | turquoise |
| ENSG00000153107 | ANAPC1 | turquoise |
| ENSG00000153113 | CAST | turquoise |
| ENSG00000153130 | SCOC | turquoise |
| ENSG00000153140 | CETN3 | turquoise |
| ENSG00000153147 | SMARCA5 | turquoise |
| ENSG00000153162 | BMP6 | turquoise |
| ENSG00000153165 | RGPD3 | turquoise |
| ENSG00000153179 | RASSF3 | turquoise |
| ENSG00000153187 | HNRNPU | turquoise |
| ENSG00000153201 | RANBP2 | turquoise |
| ENSG00000153207 | AHCTF1 | turquoise |
| ENSG00000153214 | TMEM87B | turquoise |
| ENSG00000153250 | RBMS1 | turquoise |
| ENSG00000153253 | SCN3A | turquoise |
| ENSG00000153291 | SLC25A27 | turquoise |
| ENSG00000153310 | FAM49B | turquoise |
| ENSG00000153317 | ASAP1 | turquoise |
| ENSG00000153339 | TRAPPC8 | turquoise |
| ENSG00000153363 | LINC00467 | turquoise |
| ENSG00000153443 | UBALD1 | turquoise |
| ENSG00000153487 | ING1 | turquoise |
| ENSG00000153495 | TEX29 | turquoise |
| ENSG00000153531 | ADPRHL1 | turquoise |
| ENSG00000153560 | UBP1 | turquoise |
| ENSG00000153561 | RMND5A | turquoise |
| ENSG00000153767 | GTF2E1 | turquoise |
| ENSG00000153774 | CFDP1 | turquoise |
| ENSG00000153790 | C7orf31 | turquoise |
| ENSG00000153814 | JAZF1 | turquoise |
| ENSG00000153823 | PID1 | turquoise |
| ENSG00000153827 | TRIP12 | turquoise |
| ENSG00000153832 | FBXO36 | turquoise |

| | | |
|---|---|---|
| ENSG00000153879 | CEBPG | turquoise |
| ENSG00000153902 | LGI4 | turquoise |
| ENSG00000153914 | SREK1 | turquoise |
| ENSG00000153922 | CHD1 | turquoise |
| ENSG00000153936 | HS2ST1 | turquoise |
| ENSG00000153944 | MSI2 | turquoise |
| ENSG00000153975 | ZUFSP | turquoise |
| ENSG00000153982 | GDPD1 | turquoise |
| ENSG00000153989 | NUS1 | turquoise |
| ENSG00000154001 | PPP2R5E | turquoise |
| ENSG00000154025 | SLC5A10 | turquoise |
| ENSG00000154059 | IMPACT | turquoise |
| ENSG00000154099 | DNAAF1 | turquoise |
| ENSG00000154102 | C16orf74 | turquoise |
| ENSG00000154114 | TBCEL | turquoise |
| ENSG00000154127 | UBASH3B | turquoise |
| ENSG00000154134 | ROBO3 | turquoise |
| ENSG00000154153 | RETREG1 | turquoise |
| ENSG00000154165 | GPR15 | turquoise |
| ENSG00000154174 | TOMM70 | turquoise |
| ENSG00000154175 | ABI3BP | turquoise |
| ENSG00000154217 | PITPNC1 | turquoise |
| ENSG00000154222 | CC2D1B | turquoise |
| ENSG00000154229 | PRKCA | turquoise |
| ENSG00000154237 | LRRK1 | turquoise |
| ENSG00000154262 | ABCA6 | turquoise |
| ENSG00000154263 | ABCA10 | turquoise |
| ENSG00000154265 | ABCA5 | turquoise |
| ENSG00000154310 | TNIK | turquoise |
| ENSG00000154316 | TDH | turquoise |
| ENSG00000154330 | PGM5 | turquoise |
| ENSG00000154359 | LONRF1 | turquoise |
| ENSG00000154370 | TRIM11 | turquoise |
| ENSG00000154380 | ENAH | turquoise |
| ENSG00000154429 | CCSAP | turquoise |
| ENSG00000154447 | SH3RF1 | turquoise |
| ENSG00000154511 | FAM69A | turquoise |
| ENSG00000154589 | LY96 | turquoise |
| ENSG00000154611 | PSMA8 | turquoise |
| ENSG00000154640 | BTG3 | turquoise |
| ENSG00000154642 | C21orf91 | turquoise |
| ENSG00000154655 | L3MBTL4 | turquoise |
| ENSG00000154719 | MRPL39 | turquoise |
| ENSG00000154721 | JAM2 | turquoise |
| ENSG00000154723 | ATP5J | turquoise |
| ENSG00000154727 | GABPA | turquoise |
| ENSG00000154736 | ADAMTS5 | turquoise |
| ENSG00000154767 | XPC | turquoise |

| | | |
|---|---|---|
| ENSG00000154813 | DPH3 | turquoise |
| ENSG00000154814 | OXNAD1 | turquoise |
| ENSG00000154845 | PPP4R1 | turquoise |
| ENSG00000154914 | USP43 | turquoise |
| ENSG00000154917 | RAB6B | turquoise |
| ENSG00000154920 | EME1 | turquoise |
| ENSG00000154945 | ANKRD40 | turquoise |
| ENSG00000155008 | APOOL | turquoise |
| ENSG00000155016 | CYP2U1 | turquoise |
| ENSG00000155034 | FBXL18 | turquoise |
| ENSG00000155066 | PROM2 | turquoise |
| ENSG00000155070 | UNC93B2 | turquoise |
| ENSG00000155090 | KLF10 | turquoise |
| ENSG00000155096 | AZIN1 | turquoise |
| ENSG00000155097 | ATP6V1C1 | turquoise |
| ENSG00000155099 | TMEM55A | turquoise |
| ENSG00000155100 | OTUD6B | turquoise |
| ENSG00000155111 | CDK19 | turquoise |
| ENSG00000155158 | TTC39B | turquoise |
| ENSG00000155189 | AGPAT5 | turquoise |
| ENSG00000155229 | MMS19 | turquoise |
| ENSG00000155254 | MARVELD1 | turquoise |
| ENSG00000155256 | ZFYVE27 | turquoise |
| ENSG00000155275 | TRMT44 | turquoise |
| ENSG00000155287 | SLC25A28 | turquoise |
| ENSG00000155304 | HSPA13 | turquoise |
| ENSG00000155307 | SAMSN1 | turquoise |
| ENSG00000155313 | USP25 | turquoise |
| ENSG00000155324 | GRAMD2B | turquoise |
| ENSG00000155329 | ZCCHC10 | turquoise |
| ENSG00000155330 | C16orf87 | turquoise |
| ENSG00000155366 | RHOC | turquoise |
| ENSG00000155367 | PPM1J | turquoise |
| ENSG00000155368 | DBI | turquoise |
| ENSG00000155393 | HEATR3 | turquoise |
| ENSG00000155428 | TRIM74 | turquoise |
| ENSG00000155438 | NIFK | turquoise |
| ENSG00000155506 | LARP1 | turquoise |
| ENSG00000155508 | CNOT8 | turquoise |
| ENSG00000155530 | LRGUK | turquoise |
| ENSG00000155542 | SETD9 | turquoise |
| ENSG00000155545 | MIER3 | turquoise |
| ENSG00000155561 | NUP205 | turquoise |
| ENSG00000155592 | ZKSCAN2 | turquoise |
| ENSG00000155621 | C9orf85 | turquoise |
| ENSG00000155636 | RBM45 | turquoise |
| ENSG00000155659 | VSIG4 | turquoise |
| ENSG00000155666 | KDM8 | turquoise |

| | | |
|---|---|---|
| ENSG00000155729 | KCTD18 | turquoise |
| ENSG00000155755 | TMEM237 | turquoise |
| ENSG00000155760 | FZD7 | turquoise |
| ENSG00000155792 | DEPTOR | turquoise |
| ENSG00000155827 | RNF20 | turquoise |
| ENSG00000155846 | PPARGC1B | turquoise |
| ENSG00000155850 | SLC26A2 | turquoise |
| ENSG00000155858 | LSM11 | turquoise |
| ENSG00000155868 | MED7 | turquoise |
| ENSG00000155893 | PXYLP1 | turquoise |
| ENSG00000155903 | RASA2 | turquoise |
| ENSG00000155926 | SLA | turquoise |
| ENSG00000155957 | TMBIM4 | turquoise |
| ENSG00000155959 | VBP1 | turquoise |
| ENSG00000155961 | RAB39B | turquoise |
| ENSG00000155962 | CLIC2 | turquoise |
| ENSG00000155970 | MICU3 | turquoise |
| ENSG00000155975 | VPS37A | turquoise |
| ENSG00000156011 | PSD3 | turquoise |
| ENSG00000156017 | CARNMT1 | turquoise |
| ENSG00000156026 | MCU | turquoise |
| ENSG00000156050 | FAM161B | turquoise |
| ENSG00000156052 | GNAQ | turquoise |
| ENSG00000156097 | GPR61 | turquoise |
| ENSG00000156136 | DCK | turquoise |
| ENSG00000156162 | DPY19L4 | turquoise |
| ENSG00000156170 | NDUFAF6 | turquoise |
| ENSG00000156171 | DRAM2 | turquoise |
| ENSG00000156172 | C8orf37 | turquoise |
| ENSG00000156194 | PPEF2 | turquoise |
| ENSG00000156232 | WHAMM | turquoise |
| ENSG00000156253 | RWDD2B | turquoise |
| ENSG00000156256 | USP16 | turquoise |
| ENSG00000156265 | MAP3K7CL | turquoise |
| ENSG00000156273 | BACH1 | turquoise |
| ENSG00000156298 | TSPAN7 | turquoise |
| ENSG00000156299 | TIAM1 | turquoise |
| ENSG00000156313 | RPGR | turquoise |
| ENSG00000156345 | CDK20 | turquoise |
| ENSG00000156374 | PCGF6 | turquoise |
| ENSG00000156384 | SFR1 | turquoise |
| ENSG00000156395 | SORCS3 | turquoise |
| ENSG00000156411 | C14orf2 | turquoise |
| ENSG00000156467 | UQCRB | turquoise |
| ENSG00000156469 | MTERF3 | turquoise |
| ENSG00000156482 | RPL30 | turquoise |
| ENSG00000156504 | FAM122B | turquoise |
| ENSG00000156508 | EEF1A1 | turquoise |

| | | |
|---|---|---|
| ENSG00000156521 | TYSND1 | turquoise |
| ENSG00000156531 | PHF6 | turquoise |
| ENSG00000156535 | CD109 | turquoise |
| ENSG00000156603 | MED19 | turquoise |
| ENSG00000156642 | NPTN | turquoise |
| ENSG00000156671 | SAMD8 | turquoise |
| ENSG00000156735 | BAG4 | turquoise |
| ENSG00000156738 | MS4A1 | turquoise |
| ENSG00000156787 | TBC1D31 | turquoise |
| ENSG00000156795 | WDYHV1 | turquoise |
| ENSG00000156802 | ATAD2 | turquoise |
| ENSG00000156804 | FBXO32 | turquoise |
| ENSG00000156831 | NSMCE2 | turquoise |
| ENSG00000156858 | PRR14 | turquoise |
| ENSG00000156860 | FBRS | turquoise |
| ENSG00000156869 | FRRS1 | turquoise |
| ENSG00000156875 | MFSD14A | turquoise |
| ENSG00000156876 | SASS6 | turquoise |
| ENSG00000156886 | ITGAD | turquoise |
| ENSG00000156931 | VPS8 | turquoise |
| ENSG00000156958 | GALK2 | turquoise |
| ENSG00000156970 | BUB1B | turquoise |
| ENSG00000156973 | PDE6D | turquoise |
| ENSG00000156976 | EIF4A2 | turquoise |
| ENSG00000156990 | RPUSD3 | turquoise |
| ENSG00000157017 | GHRL | turquoise |
| ENSG00000157045 | NTAN1 | turquoise |
| ENSG00000157077 | ZFYVE9 | turquoise |
| ENSG00000157106 | SMG1 | turquoise |
| ENSG00000157107 | FCHO2 | turquoise |
| ENSG00000157181 | C1orf27 | turquoise |
| ENSG00000157193 | LRP8 | turquoise |
| ENSG00000157212 | PAXIP1 | turquoise |
| ENSG00000157224 | CLDN12 | turquoise |
| ENSG00000157240 | FZD1 | turquoise |
| ENSG00000157259 | GATAD1 | turquoise |
| ENSG00000157306 | ZFHX2-AS1 | turquoise |
| ENSG00000157322 | CLEC18A | turquoise |
| ENSG00000157343 | ARMC12 | turquoise |
| ENSG00000157353 | FUK | turquoise |
| ENSG00000157404 | KIT | turquoise |
| ENSG00000157426 | AASDH | turquoise |
| ENSG00000157450 | RNF111 | turquoise |
| ENSG00000157500 | APPL1 | turquoise |
| ENSG00000157514 | TSC22D3 | turquoise |
| ENSG00000157540 | DYRK1A | turquoise |
| ENSG00000157551 | KCNJ15 | turquoise |
| ENSG00000157557 | ETS2 | turquoise |

| | | |
|---|---|---|
| ENSG00000157600 | TMEM164 | turquoise |
| ENSG00000157617 | C2CD2 | turquoise |
| ENSG00000157625 | TAB3 | turquoise |
| ENSG00000157653 | C9orf43 | turquoise |
| ENSG00000157657 | ZNF618 | turquoise |
| ENSG00000157734 | SNX22 | turquoise |
| ENSG00000157741 | UBN2 | turquoise |
| ENSG00000157764 | BRAF | turquoise |
| ENSG00000157796 | WDR19 | turquoise |
| ENSG00000157800 | SLC37A3 | turquoise |
| ENSG00000157823 | AP3S2 | turquoise |
| ENSG00000157833 | GAREM2 | turquoise |
| ENSG00000157869 | RAB28 | turquoise |
| ENSG00000157870 | FAM213B | turquoise |
| ENSG00000157873 | TNFRSF14 | turquoise |
| ENSG00000157911 | PEX10 | turquoise |
| ENSG00000157916 | RER1 | turquoise |
| ENSG00000157954 | WIPI2 | turquoise |
| ENSG00000157985 | AGAP1 | turquoise |
| ENSG00000157992 | KRTCAP3 | turquoise |
| ENSG00000158006 | PAFAH2 | turquoise |
| ENSG00000158019 | BABAM2 | turquoise |
| ENSG00000158023 | WDR66 | turquoise |
| ENSG00000158062 | UBXN11 | turquoise |
| ENSG00000158079 | PTPDC1 | turquoise |
| ENSG00000158092 | NCK1 | turquoise |
| ENSG00000158104 | HPD | turquoise |
| ENSG00000158106 | RHPN1 | turquoise |
| ENSG00000158109 | TPRG1L | turquoise |
| ENSG00000158113 | LRRC43 | turquoise |
| ENSG00000158122 | AAED1 | turquoise |
| ENSG00000158158 | CNNM4 | turquoise |
| ENSG00000158161 | EYA3 | turquoise |
| ENSG00000158195 | WASF2 | turquoise |
| ENSG00000158201 | ABHD3 | turquoise |
| ENSG00000158290 | CUL4B | turquoise |
| ENSG00000158301 | GPRASP2 | turquoise |
| ENSG00000158321 | AUTS2 | turquoise |
| ENSG00000158352 | SHROOM4 | turquoise |
| ENSG00000158411 | MITD1 | turquoise |
| ENSG00000158417 | EIF5B | turquoise |
| ENSG00000158423 | RIBC1 | turquoise |
| ENSG00000158428 | CATIP | turquoise |
| ENSG00000158435 | CNOT11 | turquoise |
| ENSG00000158445 | KCNB1 | turquoise |
| ENSG00000158470 | B4GALT5 | turquoise |
| ENSG00000158477 | CD1A | turquoise |
| ENSG00000158480 | SPATA2 | turquoise |

| | | |
|---|---|---|
| ENSG00000158481 | CD1C | turquoise |
| ENSG00000158552 | ZFAND2B | turquoise |
| ENSG00000158555 | GDPD5 | turquoise |
| ENSG00000158571 | PFKFB1 | turquoise |
| ENSG00000158615 | PPP1R15B | turquoise |
| ENSG00000158636 | EMSY | turquoise |
| ENSG00000158669 | GPAT4 | turquoise |
| ENSG00000158691 | ZSCAN12 | turquoise |
| ENSG00000158711 | ELK4 | turquoise |
| ENSG00000158716 | DUSP23 | turquoise |
| ENSG00000158717 | RNF166 | turquoise |
| ENSG00000158748 | HTR6 | turquoise |
| ENSG00000158792 | SPATA2L | turquoise |
| ENSG00000158815 | FGF17 | turquoise |
| ENSG00000158828 | PINK1 | turquoise |
| ENSG00000158863 | FAM160B2 | turquoise |
| ENSG00000158882 | TOMM40L | turquoise |
| ENSG00000158966 | CACHD1 | turquoise |
| ENSG00000158985 | CDC42SE2 | turquoise |
| ENSG00000158987 | RAPGEF6 | turquoise |
| ENSG00000159023 | EPB41 | turquoise |
| ENSG00000159055 | MIS18A | turquoise |
| ENSG00000159069 | FBXW5 | turquoise |
| ENSG00000159082 | SYNJ1 | turquoise |
| ENSG00000159086 | PAXBP1 | turquoise |
| ENSG00000159128 | IFNGR2 | turquoise |
| ENSG00000159131 | GART | turquoise |
| ENSG00000159140 | SON | turquoise |
| ENSG00000159147 | DONSON | turquoise |
| ENSG00000159176 | CSRP1 | turquoise |
| ENSG00000159200 | RCAN1 | turquoise |
| ENSG00000159202 | UBE2Z | turquoise |
| ENSG00000159212 | CLIC6 | turquoise |
| ENSG00000159214 | CCDC24 | turquoise |
| ENSG00000159216 | RUNX1 | turquoise |
| ENSG00000159256 | MORC3 | turquoise |
| ENSG00000159263 | SIM2 | turquoise |
| ENSG00000159307 | SCUBE1 | turquoise |
| ENSG00000159322 | ADPGK | turquoise |
| ENSG00000159363 | ATP13A2 | turquoise |
| ENSG00000159374 | M1AP | turquoise |
| ENSG00000159388 | BTG2 | turquoise |
| ENSG00000159399 | HK2 | turquoise |
| ENSG00000159403 | C1R | turquoise |
| ENSG00000159459 | UBR1 | turquoise |
| ENSG00000159461 | AMFR | turquoise |
| ENSG00000159556 | ISL2 | turquoise |
| ENSG00000159579 | RSPRY1 | turquoise |

| | | |
|---|---|---|
| ENSG00000159592 | GPBP1L1 | turquoise |
| ENSG00000159593 | NAE1 | turquoise |
| ENSG00000159596 | TMEM69 | turquoise |
| ENSG00000159658 | EFCAB14 | turquoise |
| ENSG00000159708 | LRRC36 | turquoise |
| ENSG00000159713 | TPPP3 | turquoise |
| ENSG00000159714 | ZDHHC1 | turquoise |
| ENSG00000159761 | C16orf86 | turquoise |
| ENSG00000159788 | RGS12 | turquoise |
| ENSG00000159792 | PSKH1 | turquoise |
| ENSG00000159842 | ABR | turquoise |
| ENSG00000159860 | TCAF2P1 | turquoise |
| ENSG00000159873 | CCDC117 | turquoise |
| ENSG00000159882 | ZNF230 | turquoise |
| ENSG00000159884 | CCDC107 | turquoise |
| ENSG00000159885 | ZNF222 | turquoise |
| ENSG00000159905 | ZNF221 | turquoise |
| ENSG00000159915 | ZNF233 | turquoise |
| ENSG00000159921 | GNE | turquoise |
| ENSG00000160007 | ARHGAP35 | turquoise |
| ENSG00000160014 | CALM3 | turquoise |
| ENSG00000160050 | CCDC28B | turquoise |
| ENSG00000160062 | ZBTB8A | turquoise |
| ENSG00000160072 | ATAD3B | turquoise |
| ENSG00000160087 | UBE2J2 | turquoise |
| ENSG00000160097 | FNDC5 | turquoise |
| ENSG00000160111 | CPAMD8 | turquoise |
| ENSG00000160113 | NR2F6 | turquoise |
| ENSG00000160124 | CCDC58 | turquoise |
| ENSG00000160131 | VMA21 | turquoise |
| ENSG00000160145 | KALRN | turquoise |
| ENSG00000160172 | FAM86C2P | turquoise |
| ENSG00000160179 | ABCG1 | turquoise |
| ENSG00000160191 | PDE9A | turquoise |
| ENSG00000160194 | NDUFV3 | turquoise |
| ENSG00000160208 | RRP1B | turquoise |
| ENSG00000160209 | PDXK | turquoise |
| ENSG00000160219 | GAB3 | turquoise |
| ENSG00000160221 | C21orf33 | turquoise |
| ENSG00000160226 | C21orf2 | turquoise |
| ENSG00000160298 | C21orf58 | turquoise |
| ENSG00000160299 | PCNT | turquoise |
| ENSG00000160307 | S100B | turquoise |
| ENSG00000160336 | ZNF761 | turquoise |
| ENSG00000160360 | GPSM1 | turquoise |
| ENSG00000160392 | C19orf47 | turquoise |
| ENSG00000160404 | TOR2A | turquoise |
| ENSG00000160408 | ST6GALNAC6 | turquoise |

| | | |
|---|---|---|
| ENSG00000160410 | SHKBP1 | turquoise |
| ENSG00000160445 | ZER1 | turquoise |
| ENSG00000160446 | ZDHHC12 | turquoise |
| ENSG00000160447 | PKN3 | turquoise |
| ENSG00000160539 | PLPP7 | turquoise |
| ENSG00000160551 | TAOK1 | turquoise |
| ENSG00000160588 | MPZL3 | turquoise |
| ENSG00000160602 | NEK8 | turquoise |
| ENSG00000160678 | S100A1 | turquoise |
| ENSG00000160679 | CHTOP | turquoise |
| ENSG00000160691 | SHC1 | turquoise |
| ENSG00000160703 | NLRX1 | turquoise |
| ENSG00000160712 | IL6R | turquoise |
| ENSG00000160741 | CRTC2 | turquoise |
| ENSG00000160746 | ANO10 | turquoise |
| ENSG00000160753 | RUSC1 | turquoise |
| ENSG00000160766 | GBAP1 | turquoise |
| ENSG00000160781 | PAQR6 | turquoise |
| ENSG00000160791 | CCR5 | turquoise |
| ENSG00000160867 | FGFR4 | turquoise |
| ENSG00000160877 | NACC1 | turquoise |
| ENSG00000160908 | ZNF394 | turquoise |
| ENSG00000160948 | VPS28 | turquoise |
| ENSG00000160949 | TONSL | turquoise |
| ENSG00000160953 | MUM1 | turquoise |
| ENSG00000160957 | RECQL4 | turquoise |
| ENSG00000160959 | LRRC14 | turquoise |
| ENSG00000160972 | PPP1R16A | turquoise |
| ENSG00000160993 | ALKBH4 | turquoise |
| ENSG00000160999 | SH2B2 | turquoise |
| ENSG00000161011 | SQSTM1 | turquoise |
| ENSG00000161031 | PGLYRP2 | turquoise |
| ENSG00000161036 | LRWD1 | turquoise |
| ENSG00000161055 | SCGB3A1 | turquoise |
| ENSG00000161057 | PSMC2 | turquoise |
| ENSG00000161091 | MFSD12 | turquoise |
| ENSG00000161180 | CCDC116 | turquoise |
| ENSG00000161202 | DVL3 | turquoise |
| ENSG00000161204 | ABCF3 | turquoise |
| ENSG00000161265 | U2AF1L4 | turquoise |
| ENSG00000161277 | THAP8 | turquoise |
| ENSG00000161298 | ZNF382 | turquoise |
| ENSG00000161328 | LRRC56 | turquoise |
| ENSG00000161509 | GRIN2C | turquoise |
| ENSG00000161526 | SAP30BP | turquoise |
| ENSG00000161544 | CYGB | turquoise |
| ENSG00000161547 | SRSF2 | turquoise |
| ENSG00000161558 | TMEM143 | turquoise |

| | | |
|---|---|---|
| ENSG00000161638 | ITGA5 | turquoise |
| ENSG00000161642 | ZNF385A | turquoise |
| ENSG00000161643 | SIGLEC16 | turquoise |
| ENSG00000161653 | NAGS | turquoise |
| ENSG00000161654 | LSM12 | turquoise |
| ENSG00000161681 | SHANK1 | turquoise |
| ENSG00000161682 | FAM171A2 | turquoise |
| ENSG00000161692 | DBF4B | turquoise |
| ENSG00000161714 | PLCD3 | turquoise |
| ENSG00000161791 | FMNL3 | turquoise |
| ENSG00000161800 | RACGAP1 | turquoise |
| ENSG00000161813 | LARP4 | turquoise |
| ENSG00000161835 | GRASP | turquoise |
| ENSG00000161847 | RAVER1 | turquoise |
| ENSG00000161860 | SYCE2 | turquoise |
| ENSG00000161904 | LEMD2 | turquoise |
| ENSG00000161914 | ZNF653 | turquoise |
| ENSG00000161920 | MED11 | turquoise |
| ENSG00000161944 | ASGR2 | turquoise |
| ENSG00000161970 | RPL26 | turquoise |
| ENSG00000161981 | SNRNP25 | turquoise |
| ENSG00000161996 | WDR90 | turquoise |
| ENSG00000161999 | JMJD8 | turquoise |
| ENSG00000162004 | CCDC78 | turquoise |
| ENSG00000162066 | AMDHD2 | turquoise |
| ENSG00000162069 | BICDL2 | turquoise |
| ENSG00000162073 | PAQR4 | turquoise |
| ENSG00000162086 | ZNF75A | turquoise |
| ENSG00000162129 | CLPB | turquoise |
| ENSG00000162139 | NEU3 | turquoise |
| ENSG00000162144 | CYB561A3 | turquoise |
| ENSG00000162148 | PPP1R32 | turquoise |
| ENSG00000162194 | LBHD1 | turquoise |
| ENSG00000162222 | TTC9C | turquoise |
| ENSG00000162227 | TAF6L | turquoise |
| ENSG00000162231 | NXF1 | turquoise |
| ENSG00000162236 | STX5 | turquoise |
| ENSG00000162241 | SLC25A45 | turquoise |
| ENSG00000162267 | ITIH3 | turquoise |
| ENSG00000162298 | SYVN1 | turquoise |
| ENSG00000162300 | ZFPL1 | turquoise |
| ENSG00000162302 | RPS6KA4 | turquoise |
| ENSG00000162368 | CMPK1 | turquoise |
| ENSG00000162377 | COA7 | turquoise |
| ENSG00000162378 | ZYG11B | turquoise |
| ENSG00000162384 | C1orf123 | turquoise |
| ENSG00000162402 | USP24 | turquoise |
| ENSG00000162407 | PLPP3 | turquoise |

| | | |
|---|---|---|
| ENSG00000162430 | SELENON | turquoise |
| ENSG00000162434 | JAK1 | turquoise |
| ENSG00000162438 | CTRC | turquoise |
| ENSG00000162441 | LZIC | turquoise |
| ENSG00000162444 | RBP7 | turquoise |
| ENSG00000162461 | SLC25A34 | turquoise |
| ENSG00000162482 | AKR7A3 | turquoise |
| ENSG00000162510 | MATN1 | turquoise |
| ENSG00000162511 | LAPTM5 | turquoise |
| ENSG00000162521 | RBBP4 | turquoise |
| ENSG00000162522 | KIAA1522 | turquoise |
| ENSG00000162542 | TMCO4 | turquoise |
| ENSG00000162571 | TTLL10 | turquoise |
| ENSG00000162572 | SCNN1D | turquoise |
| ENSG00000162576 | MXRA8 | turquoise |
| ENSG00000162591 | MEGF6 | turquoise |
| ENSG00000162600 | OMA1 | turquoise |
| ENSG00000162601 | MYSM1 | turquoise |
| ENSG00000162604 | TM2D1 | turquoise |
| ENSG00000162607 | USP1 | turquoise |
| ENSG00000162613 | FUBP1 | turquoise |
| ENSG00000162616 | DNAJB4 | turquoise |
| ENSG00000162620 | LRRIQ3 | turquoise |
| ENSG00000162623 | TYW3 | turquoise |
| ENSG00000162630 | B3GALT2 | turquoise |
| ENSG00000162636 | FAM102B | turquoise |
| ENSG00000162639 | HENMT1 | turquoise |
| ENSG00000162642 | C1orf52 | turquoise |
| ENSG00000162650 | ATXN7L2 | turquoise |
| ENSG00000162664 | ZNF326 | turquoise |
| ENSG00000162676 | GFI1 | turquoise |
| ENSG00000162688 | AGL | turquoise |
| ENSG00000162694 | EXTL2 | turquoise |
| ENSG00000162695 | SLC30A7 | turquoise |
| ENSG00000162702 | ZNF281 | turquoise |
| ENSG00000162704 | ARPC5 | turquoise |
| ENSG00000162733 | DDR2 | turquoise |
| ENSG00000162769 | FLVCR1 | turquoise |
| ENSG00000162775 | RBM15 | turquoise |
| ENSG00000162783 | IER5 | turquoise |
| ENSG00000162813 | BPNT1 | turquoise |
| ENSG00000162819 | BROX | turquoise |
| ENSG00000162825 | NBPF20 | turquoise |
| ENSG00000162836 | ACP6 | turquoise |
| ENSG00000162851 | TFB2M | turquoise |
| ENSG00000162852 | CNST | turquoise |
| ENSG00000162869 | PPP1R21 | turquoise |
| ENSG00000162878 | PKDCC | turquoise |

| | | |
|---|---|---|
| ENSG00000162881 | OXER1 | turquoise |
| ENSG00000162882 | HAAO | turquoise |
| ENSG00000162885 | B3GALNT2 | turquoise |
| ENSG00000162923 | WDR26 | turquoise |
| ENSG00000162927 | PUS10 | turquoise |
| ENSG00000162928 | PEX13 | turquoise |
| ENSG00000162944 | RFTN2 | turquoise |
| ENSG00000162949 | CAPN13 | turquoise |
| ENSG00000162961 | DPY30 | turquoise |
| ENSG00000162971 | TYW5 | turquoise |
| ENSG00000162972 | MAIP1 | turquoise |
| ENSG00000162976 | PQLC3 | turquoise |
| ENSG00000162980 | ARL5A | turquoise |
| ENSG00000162999 | DUSP19 | turquoise |
| ENSG00000163001 | CFAP36 | turquoise |
| ENSG00000163002 | NUP35 | turquoise |
| ENSG00000163006 | CCDC138 | turquoise |
| ENSG00000163009 | C2orf48 | turquoise |
| ENSG00000163013 | FBXO41 | turquoise |
| ENSG00000163029 | SMC6 | turquoise |
| ENSG00000163053 | SLC16A14 | turquoise |
| ENSG00000163069 | SGCB | turquoise |
| ENSG00000163082 | SGPP2 | turquoise |
| ENSG00000163093 | BBS5 | turquoise |
| ENSG00000163104 | SMARCAD1 | turquoise |
| ENSG00000163110 | PDLIM5 | turquoise |
| ENSG00000163125 | RPRD2 | turquoise |
| ENSG00000163126 | ANKRD23 | turquoise |
| ENSG00000163138 | PACRGL | turquoise |
| ENSG00000163141 | BNIPL | turquoise |
| ENSG00000163154 | TNFAIP8L2 | turquoise |
| ENSG00000163155 | LYSMD1 | turquoise |
| ENSG00000163156 | SCNM1 | turquoise |
| ENSG00000163162 | RNF149 | turquoise |
| ENSG00000163166 | IWS1 | turquoise |
| ENSG00000163171 | CDC42EP3 | turquoise |
| ENSG00000163214 | DHX57 | turquoise |
| ENSG00000163220 | S100A9 | turquoise |
| ENSG00000163235 | TGFA | turquoise |
| ENSG00000163249 | CCNYL1 | turquoise |
| ENSG00000163257 | DCAF16 | turquoise |
| ENSG00000163281 | GNPDA2 | turquoise |
| ENSG00000163291 | PAQR3 | turquoise |
| ENSG00000163293 | NIPAL1 | turquoise |
| ENSG00000163297 | ANTXR2 | turquoise |
| ENSG00000163312 | HELQ | turquoise |
| ENSG00000163319 | MRPS18C | turquoise |
| ENSG00000163320 | CGGBP1 | turquoise |

| | | |
|---|---|---|
| ENSG00000163322 | ABRAXAS1 | turquoise |
| ENSG00000163346 | PBXIP1 | turquoise |
| ENSG00000163348 | PYGO2 | turquoise |
| ENSG00000163349 | HIPK1 | turquoise |
| ENSG00000163354 | DCST2 | turquoise |
| ENSG00000163357 | DCST1 | turquoise |
| ENSG00000163374 | YY1AP1 | turquoise |
| ENSG00000163376 | KBTBD8 | turquoise |
| ENSG00000163378 | EOGT | turquoise |
| ENSG00000163389 | POGLUT1 | turquoise |
| ENSG00000163393 | SLC22A15 | turquoise |
| ENSG00000163406 | SLC15A2 | turquoise |
| ENSG00000163412 | EIF4E3 | turquoise |
| ENSG00000163421 | PROK2 | turquoise |
| ENSG00000163428 | LRRC58 | turquoise |
| ENSG00000163431 | LMOD1 | turquoise |
| ENSG00000163435 | ELF3 | turquoise |
| ENSG00000163444 | TMEM183A | turquoise |
| ENSG00000163462 | TRIM46 | turquoise |
| ENSG00000163463 | KRTCAP2 | turquoise |
| ENSG00000163472 | TMEM79 | turquoise |
| ENSG00000163482 | STK36 | turquoise |
| ENSG00000163507 | KIAA1524 | turquoise |
| ENSG00000163510 | CWC22 | turquoise |
| ENSG00000163512 | AZI2 | turquoise |
| ENSG00000163513 | TGFBR2 | turquoise |
| ENSG00000163516 | ANKZF1 | turquoise |
| ENSG00000163517 | HDAC11 | turquoise |
| ENSG00000163519 | TRAT1 | turquoise |
| ENSG00000163521 | GLB1L | turquoise |
| ENSG00000163527 | STT3B | turquoise |
| ENSG00000163531 | NFASC | turquoise |
| ENSG00000163535 | SGO2 | turquoise |
| ENSG00000163536 | SERPINI1 | turquoise |
| ENSG00000163539 | CLASP2 | turquoise |
| ENSG00000163558 | PRKCI | turquoise |
| ENSG00000163577 | EIF5A2 | turquoise |
| ENSG00000163584 | RPL22L1 | turquoise |
| ENSG00000163590 | PPM1L | turquoise |
| ENSG00000163597 | SNHG16 | turquoise |
| ENSG00000163599 | CTLA4 | turquoise |
| ENSG00000163600 | ICOS | turquoise |
| ENSG00000163602 | RYBP | turquoise |
| ENSG00000163605 | PPP4R2 | turquoise |
| ENSG00000163606 | CD200R1 | turquoise |
| ENSG00000163607 | GTPBP8 | turquoise |
| ENSG00000163608 | NEPRO | turquoise |
| ENSG00000163611 | SPICE1 | turquoise |

| | | |
|---|---|---|
| ENSG00000163617 | CCDC191 | turquoise |
| ENSG00000163625 | WDFY3 | turquoise |
| ENSG00000163626 | COX18 | turquoise |
| ENSG00000163629 | PTPN13 | turquoise |
| ENSG00000163634 | THOC7 | turquoise |
| ENSG00000163635 | ATXN7 | turquoise |
| ENSG00000163636 | PSMD6 | turquoise |
| ENSG00000163637 | PRICKLE2 | turquoise |
| ENSG00000163644 | PPM1K | turquoise |
| ENSG00000163655 | GMPS | turquoise |
| ENSG00000163659 | TIPARP | turquoise |
| ENSG00000163660 | CCNL1 | turquoise |
| ENSG00000163661 | PTX3 | turquoise |
| ENSG00000163681 | SLMAP | turquoise |
| ENSG00000163682 | RPL9 | turquoise |
| ENSG00000163683 | SMIM14 | turquoise |
| ENSG00000163684 | RPP14 | turquoise |
| ENSG00000163686 | ABHD6 | turquoise |
| ENSG00000163687 | DNASE1L3 | turquoise |
| ENSG00000163694 | RBM47 | turquoise |
| ENSG00000163701 | IL17RE | turquoise |
| ENSG00000163702 | IL17RC | turquoise |
| ENSG00000163703 | CRELD1 | turquoise |
| ENSG00000163704 | PRRT3 | turquoise |
| ENSG00000163714 | U2SURP | turquoise |
| ENSG00000163719 | MTMR14 | turquoise |
| ENSG00000163728 | TTC14 | turquoise |
| ENSG00000163735 | CXCL5 | turquoise |
| ENSG00000163736 | PPBP | turquoise |
| ENSG00000163738 | MTHFD2L | turquoise |
| ENSG00000163739 | CXCL1 | turquoise |
| ENSG00000163743 | RCHY1 | turquoise |
| ENSG00000163755 | HPS3 | turquoise |
| ENSG00000163781 | TOPBP1 | turquoise |
| ENSG00000163785 | RYK | turquoise |
| ENSG00000163788 | SNRK | turquoise |
| ENSG00000163795 | ZNF513 | turquoise |
| ENSG00000163798 | SLC4A1AP | turquoise |
| ENSG00000163807 | KIAA1143 | turquoise |
| ENSG00000163811 | WDR43 | turquoise |
| ENSG00000163814 | CDCP1 | turquoise |
| ENSG00000163815 | CLEC3B | turquoise |
| ENSG00000163818 | LZTFL1 | turquoise |
| ENSG00000163820 | FYCO1 | turquoise |
| ENSG00000163833 | FBXO40 | turquoise |
| ENSG00000163848 | ZNF148 | turquoise |
| ENSG00000163866 | SMIM12 | turquoise |
| ENSG00000163867 | ZMYM6 | turquoise |

| | | |
|---|---|---|
| ENSG00000163870 | TPRA1 | turquoise |
| ENSG00000163872 | YEATS2 | turquoise |
| ENSG00000163874 | ZC3H12A | turquoise |
| ENSG00000163877 | SNIP1 | turquoise |
| ENSG00000163902 | RPN1 | turquoise |
| ENSG00000163904 | SENP2 | turquoise |
| ENSG00000163913 | IFT122 | turquoise |
| ENSG00000163918 | RFC4 | turquoise |
| ENSG00000163923 | RPL39L | turquoise |
| ENSG00000163930 | BAP1 | turquoise |
| ENSG00000163931 | TKT | turquoise |
| ENSG00000163933 | RFT1 | turquoise |
| ENSG00000163938 | GNL3 | turquoise |
| ENSG00000163939 | PBRM1 | turquoise |
| ENSG00000163946 | FAM208A | turquoise |
| ENSG00000163947 | ARHGEF3 | turquoise |
| ENSG00000163950 | SLBP | turquoise |
| ENSG00000163956 | LRPAP1 | turquoise |
| ENSG00000163958 | ZDHHC19 | turquoise |
| ENSG00000163959 | SLC51A | turquoise |
| ENSG00000163961 | RNF168 | turquoise |
| ENSG00000163964 | PIGX | turquoise |
| ENSG00000163975 | MELTF | turquoise |
| ENSG00000163995 | ABLIM2 | turquoise |
| ENSG00000164008 | C1orf50 | turquoise |
| ENSG00000164011 | ZNF691 | turquoise |
| ENSG00000164022 | AIMP1 | turquoise |
| ENSG00000164023 | SGMS2 | turquoise |
| ENSG00000164024 | METAP1 | turquoise |
| ENSG00000164031 | DNAJB14 | turquoise |
| ENSG00000164037 | SLC9B1 | turquoise |
| ENSG00000164039 | BDH2 | turquoise |
| ENSG00000164040 | PGRMC2 | turquoise |
| ENSG00000164045 | CDC25A | turquoise |
| ENSG00000164048 | ZNF589 | turquoise |
| ENSG00000164050 | PLXNB1 | turquoise |
| ENSG00000164056 | SPRY1 | turquoise |
| ENSG00000164068 | RNF123 | turquoise |
| ENSG00000164073 | MFSD8 | turquoise |
| ENSG00000164074 | ABHD18 | turquoise |
| ENSG00000164077 | MON1A | turquoise |
| ENSG00000164078 | MST1R | turquoise |
| ENSG00000164080 | RAD54L2 | turquoise |
| ENSG00000164081 | TEX264 | turquoise |
| ENSG00000164086 | DUSP7 | turquoise |
| ENSG00000164087 | POC1A | turquoise |
| ENSG00000164088 | PPM1M | turquoise |
| ENSG00000164091 | WDR82 | turquoise |

| | | |
|---|---|---|
| ENSG00000164104 | HMGB2 | turquoise |
| ENSG00000164109 | MAD2L1 | turquoise |
| ENSG00000164114 | MAP9 | turquoise |
| ENSG00000164116 | GUCY1A3 | turquoise |
| ENSG00000164117 | FBXO8 | turquoise |
| ENSG00000164118 | CEP44 | turquoise |
| ENSG00000164120 | HPGD | turquoise |
| ENSG00000164124 | TMEM144 | turquoise |
| ENSG00000164125 | FAM198B | turquoise |
| ENSG00000164134 | NAA15 | turquoise |
| ENSG00000164136 | IL15 | turquoise |
| ENSG00000164144 | ARFIP1 | turquoise |
| ENSG00000164151 | ICE1 | turquoise |
| ENSG00000164162 | ANAPC10 | turquoise |
| ENSG00000164163 | ABCE1 | turquoise |
| ENSG00000164164 | OTUD4 | turquoise |
| ENSG00000164168 | TMEM184C | turquoise |
| ENSG00000164169 | PRMT9 | turquoise |
| ENSG00000164171 | ITGA2 | turquoise |
| ENSG00000164172 | MOCS2 | turquoise |
| ENSG00000164180 | TMEM161B | turquoise |
| ENSG00000164181 | ELOVL7 | turquoise |
| ENSG00000164187 | LMBRD2 | turquoise |
| ENSG00000164190 | NIPBL | turquoise |
| ENSG00000164199 | ADGRV1 | turquoise |
| ENSG00000164209 | SLC25A46 | turquoise |
| ENSG00000164211 | STARD4 | turquoise |
| ENSG00000164219 | PGGT1B | turquoise |
| ENSG00000164221 | CCDC112 | turquoise |
| ENSG00000164236 | ANKRD33B | turquoise |
| ENSG00000164241 | C5orf63 | turquoise |
| ENSG00000164244 | PRRC1 | turquoise |
| ENSG00000164251 | F2RL1 | turquoise |
| ENSG00000164252 | AGGF1 | turquoise |
| ENSG00000164253 | WDR41 | turquoise |
| ENSG00000164258 | NDUFS4 | turquoise |
| ENSG00000164283 | ESM1 | turquoise |
| ENSG00000164284 | GRPEL2 | turquoise |
| ENSG00000164291 | ARSK | turquoise |
| ENSG00000164292 | RHOBTB3 | turquoise |
| ENSG00000164305 | CASP3 | turquoise |
| ENSG00000164306 | PRIMPOL | turquoise |
| ENSG00000164307 | ERAP1 | turquoise |
| ENSG00000164323 | CFAP97 | turquoise |
| ENSG00000164327 | RICTOR | turquoise |
| ENSG00000164329 | PAPD4 | turquoise |
| ENSG00000164331 | ANKRA2 | turquoise |
| ENSG00000164332 | UBLCP1 | turquoise |

| | | |
|---|---|---|
| ENSG00000164338 | UTP15 | turquoise |
| ENSG00000164342 | TLR3 | turquoise |
| ENSG00000164344 | KLKB1 | turquoise |
| ENSG00000164346 | NSA2 | turquoise |
| ENSG00000164347 | GFM2 | turquoise |
| ENSG00000164366 | CCDC127 | turquoise |
| ENSG00000164398 | ACSL6 | turquoise |
| ENSG00000164405 | UQCRQ | turquoise |
| ENSG00000164406 | LEAP2 | turquoise |
| ENSG00000164414 | SLC35A1 | turquoise |
| ENSG00000164430 | MB21D1 | turquoise |
| ENSG00000164440 | TXLNB | turquoise |
| ENSG00000164442 | CITED2 | turquoise |
| ENSG00000164463 | CREBRF | turquoise |
| ENSG00000164465 | DCBLD1 | turquoise |
| ENSG00000164484 | TMEM200A | turquoise |
| ENSG00000164494 | PDSS2 | turquoise |
| ENSG00000164506 | STXBP5 | turquoise |
| ENSG00000164535 | DAGLB | turquoise |
| ENSG00000164542 | KIAA0895 | turquoise |
| ENSG00000164543 | STK17A | turquoise |
| ENSG00000164548 | TRA2A | turquoise |
| ENSG00000164576 | SAP30L | turquoise |
| ENSG00000164603 | BMT2 | turquoise |
| ENSG00000164609 | SLU7 | turquoise |
| ENSG00000164615 | CAMLG | turquoise |
| ENSG00000164620 | RELL2 | turquoise |
| ENSG00000164626 | KCNK5 | turquoise |
| ENSG00000164631 | ZNF12 | turquoise |
| ENSG00000164649 | CDCA7L | turquoise |
| ENSG00000164654 | MIOS | turquoise |
| ENSG00000164674 | SYTL3 | turquoise |
| ENSG00000164684 | ZNF704 | turquoise |
| ENSG00000164691 | TAGAP | turquoise |
| ENSG00000164707 | SLC13A4 | turquoise |
| ENSG00000164715 | LMTK2 | turquoise |
| ENSG00000164741 | DLC1 | turquoise |
| ENSG00000164751 | PEX2 | turquoise |
| ENSG00000164754 | RAD21 | turquoise |
| ENSG00000164758 | MED30 | turquoise |
| ENSG00000164764 | SBSPON | turquoise |
| ENSG00000164808 | SPIDR | turquoise |
| ENSG00000164815 | ORC5 | turquoise |
| ENSG00000164823 | OSGIN2 | turquoise |
| ENSG00000164828 | SUN1 | turquoise |
| ENSG00000164830 | OXR1 | turquoise |
| ENSG00000164845 | FAM86FP | turquoise |
| ENSG00000164850 | GPER1 | turquoise |

| | | |
|---|---|---|
| ENSG00000164877 | MICALL2 | turquoise |
| ENSG00000164880 | INTS1 | turquoise |
| ENSG00000164889 | SLC4A2 | turquoise |
| ENSG00000164898 | FMC1 | turquoise |
| ENSG00000164902 | PHAX | turquoise |
| ENSG00000164904 | ALDH7A1 | turquoise |
| ENSG00000164916 | FOXK1 | turquoise |
| ENSG00000164919 | COX6C | turquoise |
| ENSG00000164924 | YWHAZ | turquoise |
| ENSG00000164930 | FZD6 | turquoise |
| ENSG00000164933 | SLC25A32 | turquoise |
| ENSG00000164934 | DCAF13 | turquoise |
| ENSG00000164938 | TP53INP1 | turquoise |
| ENSG00000164941 | INTS8 | turquoise |
| ENSG00000164944 | VIRMA | turquoise |
| ENSG00000164951 | PDP1 | turquoise |
| ENSG00000164961 | WASHC5 | turquoise |
| ENSG00000164976 | KIAA1161 | turquoise |
| ENSG00000164978 | NUDT2 | turquoise |
| ENSG00000164983 | TMEM65 | turquoise |
| ENSG00000164985 | PSIP1 | turquoise |
| ENSG00000165029 | ABCA1 | turquoise |
| ENSG00000165055 | METTL2B | turquoise |
| ENSG00000165071 | TMEM71 | turquoise |
| ENSG00000165097 | KDM1B | turquoise |
| ENSG00000165102 | HGSNAT | turquoise |
| ENSG00000165113 | GKAP1 | turquoise |
| ENSG00000165118 | C9orf64 | turquoise |
| ENSG00000165121 | AL353743.1 | turquoise |
| ENSG00000165125 | TRPV6 | turquoise |
| ENSG00000165138 | ANKS6 | turquoise |
| ENSG00000165156 | ZHX1 | turquoise |
| ENSG00000165169 | DYNLT3 | turquoise |
| ENSG00000165195 | PIGA | turquoise |
| ENSG00000165209 | STRBP | turquoise |
| ENSG00000165219 | GAPVD1 | turquoise |
| ENSG00000165233 | CARD19 | turquoise |
| ENSG00000165238 | WNK2 | turquoise |
| ENSG00000165240 | ATP7A | turquoise |
| ENSG00000165244 | ZNF367 | turquoise |
| ENSG00000165259 | HDX | turquoise |
| ENSG00000165282 | PIGO | turquoise |
| ENSG00000165300 | SLITRK5 | turquoise |
| ENSG00000165312 | OTUD1 | turquoise |
| ENSG00000165322 | ARHGAP12 | turquoise |
| ENSG00000165338 | HECTD2 | turquoise |
| ENSG00000165355 | FBXO33 | turquoise |
| ENSG00000165359 | INTS6L | turquoise |

| | | |
|---|---|---|
| ENSG00000165389 | SPTSSA | turquoise |
| ENSG00000165406 | 8-Mar | turquoise |
| ENSG00000165410 | CFL2 | turquoise |
| ENSG00000165416 | SUGT1 | turquoise |
| ENSG00000165417 | GTF2A1 | turquoise |
| ENSG00000165424 | ZCCHC24 | turquoise |
| ENSG00000165434 | PGM2L1 | turquoise |
| ENSG00000165458 | INPPL1 | turquoise |
| ENSG00000165476 | REEP3 | turquoise |
| ENSG00000165487 | MICU2 | turquoise |
| ENSG00000165490 | DDIAS | turquoise |
| ENSG00000165501 | LRR1 | turquoise |
| ENSG00000165506 | DNAAF2 | turquoise |
| ENSG00000165512 | ZNF22 | turquoise |
| ENSG00000165516 | KLHDC2 | turquoise |
| ENSG00000165521 | EML5 | turquoise |
| ENSG00000165527 | ARF6 | turquoise |
| ENSG00000165533 | TTC8 | turquoise |
| ENSG00000165572 | KBTBD6 | turquoise |
| ENSG00000165591 | FAAH2 | turquoise |
| ENSG00000165609 | NUDT5 | turquoise |
| ENSG00000165617 | DACT1 | turquoise |
| ENSG00000165626 | BEND7 | turquoise |
| ENSG00000165629 | ATP5C1 | turquoise |
| ENSG00000165630 | PRPF18 | turquoise |
| ENSG00000165632 | TAF3 | turquoise |
| ENSG00000165644 | COMTD1 | turquoise |
| ENSG00000165650 | PDZD8 | turquoise |
| ENSG00000165655 | ZNF503 | turquoise |
| ENSG00000165660 | ABRAXAS2 | turquoise |
| ENSG00000165661 | QSOX2 | turquoise |
| ENSG00000165669 | FAM204A | turquoise |
| ENSG00000165671 | NSD1 | turquoise |
| ENSG00000165672 | PRDX3 | turquoise |
| ENSG00000165675 | ENOX2 | turquoise |
| ENSG00000165682 | CLEC1B | turquoise |
| ENSG00000165684 | SNAPC4 | turquoise |
| ENSG00000165689 | SDCCAG3 | turquoise |
| ENSG00000165695 | AK8 | turquoise |
| ENSG00000165698 | SPACA9 | turquoise |
| ENSG00000165704 | HPRT1 | turquoise |
| ENSG00000165716 | FAM69B | turquoise |
| ENSG00000165731 | RET | turquoise |
| ENSG00000165732 | DDX21 | turquoise |
| ENSG00000165752 | STK32C | turquoise |
| ENSG00000165782 | TMEM55B | turquoise |
| ENSG00000165795 | NDRG2 | turquoise |
| ENSG00000165802 | NSMF | turquoise |

| | | |
|---|---|---|
| ENSG00000165804 | ZNF219 | turquoise |
| ENSG00000165806 | CASP7 | turquoise |
| ENSG00000165810 | BTNL9 | turquoise |
| ENSG00000165813 | CCDC186 | turquoise |
| ENSG00000165832 | TRUB1 | turquoise |
| ENSG00000165837 | ERICH6B | turquoise |
| ENSG00000165861 | ZFYVE1 | turquoise |
| ENSG00000165874 | FAM35BP | turquoise |
| ENSG00000165886 | UBTD1 | turquoise |
| ENSG00000165891 | E2F7 | turquoise |
| ENSG00000165895 | ARHGAP42 | turquoise |
| ENSG00000165914 | TTC7B | turquoise |
| ENSG00000165915 | SLC39A13 | turquoise |
| ENSG00000165917 | RAPSN | turquoise |
| ENSG00000165929 | TC2N | turquoise |
| ENSG00000165934 | CPSF2 | turquoise |
| ENSG00000165948 | IFI27L1 | turquoise |
| ENSG00000165959 | CLMN | turquoise |
| ENSG00000165983 | PTER | turquoise |
| ENSG00000165997 | ARL5B | turquoise |
| ENSG00000166012 | TAF1D | turquoise |
| ENSG00000166024 | R3HCC1L | turquoise |
| ENSG00000166037 | CEP57 | turquoise |
| ENSG00000166068 | SPRED1 | turquoise |
| ENSG00000166091 | CMTM5 | turquoise |
| ENSG00000166105 | GLB1L3 | turquoise |
| ENSG00000166123 | GPT2 | turquoise |
| ENSG00000166126 | AMN | turquoise |
| ENSG00000166128 | RAB8B | turquoise |
| ENSG00000166130 | IKBIP | turquoise |
| ENSG00000166135 | HIF1AN | turquoise |
| ENSG00000166140 | ZFYVE19 | turquoise |
| ENSG00000166145 | SPINT1 | turquoise |
| ENSG00000166147 | FBN1 | turquoise |
| ENSG00000166153 | DEPDC4 | turquoise |
| ENSG00000166164 | BRD7 | turquoise |
| ENSG00000166167 | BTRC | turquoise |
| ENSG00000166170 | BAG5 | turquoise |
| ENSG00000166181 | API5 | turquoise |
| ENSG00000166188 | ZNF319 | turquoise |
| ENSG00000166192 | SENP8 | turquoise |
| ENSG00000166197 | NOLC1 | turquoise |
| ENSG00000166200 | COPS2 | turquoise |
| ENSG00000166225 | FRS2 | turquoise |
| ENSG00000166226 | CCT2 | turquoise |
| ENSG00000166233 | ARIH1 | turquoise |
| ENSG00000166250 | CLMP | turquoise |
| ENSG00000166261 | ZNF202 | turquoise |

| | | |
|---|---|---|
| ENSG00000166262 | FAM227B | turquoise |
| ENSG00000166263 | STXBP4 | turquoise |
| ENSG00000166266 | CUL5 | turquoise |
| ENSG00000166275 | BORCS7 | turquoise |
| ENSG00000166295 | ANAPC16 | turquoise |
| ENSG00000166326 | TRIM44 | turquoise |
| ENSG00000166341 | DCHS1 | turquoise |
| ENSG00000166349 | RAG1 | turquoise |
| ENSG00000166352 | C11orf74 | turquoise |
| ENSG00000166377 | ATP9B | turquoise |
| ENSG00000166401 | SERPINB8 | turquoise |
| ENSG00000166411 | IDH3A | turquoise |
| ENSG00000166432 | ZMAT1 | turquoise |
| ENSG00000166436 | TRIM66 | turquoise |
| ENSG00000166444 | ST5 | turquoise |
| ENSG00000166446 | CDYL2 | turquoise |
| ENSG00000166451 | CENPN | turquoise |
| ENSG00000166454 | ATMIN | turquoise |
| ENSG00000166471 | TMEM41B | turquoise |
| ENSG00000166477 | LEO1 | turquoise |
| ENSG00000166478 | ZNF143 | turquoise |
| ENSG00000166479 | TMX3 | turquoise |
| ENSG00000166483 | WEE1 | turquoise |
| ENSG00000166484 | MAPK7 | turquoise |
| ENSG00000166501 | PRKCB | turquoise |
| ENSG00000166507 | NDST2 | turquoise |
| ENSG00000166527 | CLEC4D | turquoise |
| ENSG00000166529 | ZSCAN21 | turquoise |
| ENSG00000166532 | RIMKLB | turquoise |
| ENSG00000166548 | TK2 | turquoise |
| ENSG00000166562 | SEC11C | turquoise |
| ENSG00000166575 | TMEM135 | turquoise |
| ENSG00000166578 | IQCD | turquoise |
| ENSG00000166598 | HSP90B1 | turquoise |
| ENSG00000166669 | ATF7IP2 | turquoise |
| ENSG00000166676 | TVP23A | turquoise |
| ENSG00000166685 | COG1 | turquoise |
| ENSG00000166710 | B2M | turquoise |
| ENSG00000166734 | CASC4 | turquoise |
| ENSG00000166736 | HTR3A | turquoise |
| ENSG00000166747 | AP1G1 | turquoise |
| ENSG00000166750 | SLFN5 | turquoise |
| ENSG00000166762 | CATSPER2 | turquoise |
| ENSG00000166783 | MARF1 | turquoise |
| ENSG00000166797 | FAM96A | turquoise |
| ENSG00000166801 | FAM111A | turquoise |
| ENSG00000166803 | PCLAF | turquoise |
| ENSG00000166816 | LDHD | turquoise |

| | | |
|---|---|---|
| ENSG00000166821 | PEX11A | turquoise |
| ENSG00000166822 | TMEM170A | turquoise |
| ENSG00000166833 | NAV2 | turquoise |
| ENSG00000166845 | C18orf54 | turquoise |
| ENSG00000166848 | TERF2IP | turquoise |
| ENSG00000166855 | CLPX | turquoise |
| ENSG00000166860 | ZBTB39 | turquoise |
| ENSG00000166866 | MYO1A | turquoise |
| ENSG00000166881 | NEMP1 | turquoise |
| ENSG00000166887 | VPS39 | turquoise |
| ENSG00000166897 | ELFN2 | turquoise |
| ENSG00000166900 | STX3 | turquoise |
| ENSG00000166908 | PIP4K2C | turquoise |
| ENSG00000166927 | MS4A7 | turquoise |
| ENSG00000166928 | MS4A14 | turquoise |
| ENSG00000166938 | DIS3L | turquoise |
| ENSG00000166949 | SMAD3 | turquoise |
| ENSG00000166971 | AKTIP | turquoise |
| ENSG00000166974 | MAPRE2 | turquoise |
| ENSG00000166979 | EVA1C | turquoise |
| ENSG00000167004 | PDIA3 | turquoise |
| ENSG00000167005 | NUDT21 | turquoise |
| ENSG00000167034 | NKX3-1 | turquoise |
| ENSG00000167037 | SGSM1 | turquoise |
| ENSG00000167065 | DUSP18 | turquoise |
| ENSG00000167074 | TEF | turquoise |
| ENSG00000167077 | MEI1 | turquoise |
| ENSG00000167081 | PBX3 | turquoise |
| ENSG00000167088 | SNRPD1 | turquoise |
| ENSG00000167094 | TTC16 | turquoise |
| ENSG00000167100 | SAMD14 | turquoise |
| ENSG00000167103 | PIP5KL1 | turquoise |
| ENSG00000167105 | TMEM92 | turquoise |
| ENSG00000167107 | ACSF2 | turquoise |
| ENSG00000167110 | GOLGA2 | turquoise |
| ENSG00000167113 | COQ4 | turquoise |
| ENSG00000167114 | SLC27A4 | turquoise |
| ENSG00000167123 | CERCAM | turquoise |
| ENSG00000167196 | FBXO22 | turquoise |
| ENSG00000167216 | KATNAL2 | turquoise |
| ENSG00000167232 | ZNF91 | turquoise |
| ENSG00000167258 | CDK12 | turquoise |
| ENSG00000167261 | DPEP2 | turquoise |
| ENSG00000167264 | DUS2 | turquoise |
| ENSG00000167280 | ENGASE | turquoise |
| ENSG00000167281 | RBFOX3 | turquoise |
| ENSG00000167283 | ATP5L | turquoise |
| ENSG00000167291 | TBC1D16 | turquoise |

| | | |
|---|---|---|
| ENSG00000167302 | TEPSIN | turquoise |
| ENSG00000167306 | MYO5B | turquoise |
| ENSG00000167323 | STIM1 | turquoise |
| ENSG00000167325 | RRM1 | turquoise |
| ENSG00000167333 | TRIM68 | turquoise |
| ENSG00000167363 | FN3K | turquoise |
| ENSG00000167378 | IRGQ | turquoise |
| ENSG00000167380 | ZNF226 | turquoise |
| ENSG00000167384 | ZNF180 | turquoise |
| ENSG00000167393 | PPP2R3B | turquoise |
| ENSG00000167394 | ZNF668 | turquoise |
| ENSG00000167447 | SMG8 | turquoise |
| ENSG00000167461 | RAB8A | turquoise |
| ENSG00000167483 | FAM129C | turquoise |
| ENSG00000167487 | KLHL26 | turquoise |
| ENSG00000167513 | CDT1 | turquoise |
| ENSG00000167522 | ANKRD11 | turquoise |
| ENSG00000167525 | PROCA1 | turquoise |
| ENSG00000167535 | CACNB3 | turquoise |
| ENSG00000167543 | TP53I13 | turquoise |
| ENSG00000167553 | TUBA1C | turquoise |
| ENSG00000167555 | ZNF528 | turquoise |
| ENSG00000167566 | NCKAP5L | turquoise |
| ENSG00000167578 | RAB4B | turquoise |
| ENSG00000167588 | GPD1 | turquoise |
| ENSG00000167600 | CYP2S1 | turquoise |
| ENSG00000167604 | NFKBID | turquoise |
| ENSG00000167613 | LAIR1 | turquoise |
| ENSG00000167625 | ZNF526 | turquoise |
| ENSG00000167635 | ZNF146 | turquoise |
| ENSG00000167637 | ZNF283 | turquoise |
| ENSG00000167657 | DAPK3 | turquoise |
| ENSG00000167693 | NXN | turquoise |
| ENSG00000167701 | GPT | turquoise |
| ENSG00000167702 | KIFC2 | turquoise |
| ENSG00000167703 | SLC43A2 | turquoise |
| ENSG00000167716 | WDR81 | turquoise |
| ENSG00000167721 | TSR1 | turquoise |
| ENSG00000167733 | HSD11B1L | turquoise |
| ENSG00000167740 | CYB5D2 | turquoise |
| ENSG00000167748 | KLK1 | turquoise |
| ENSG00000167769 | ACER1 | turquoise |
| ENSG00000167772 | ANGPTL4 | turquoise |
| ENSG00000167778 | SPRYD3 | turquoise |
| ENSG00000167779 | IGFBP6 | turquoise |
| ENSG00000167780 | SOAT2 | turquoise |
| ENSG00000167785 | ZNF558 | turquoise |
| ENSG00000167842 | MIS12 | turquoise |

| | | |
|---|---|---|
| ENSG00000167874 | TMEM88 | turquoise |
| ENSG00000167895 | TMC8 | turquoise |
| ENSG00000167900 | TK1 | turquoise |
| ENSG00000167904 | TMEM68 | turquoise |
| ENSG00000167945 | PRR25 | turquoise |
| ENSG00000167962 | ZNF598 | turquoise |
| ENSG00000167967 | E4F1 | turquoise |
| ENSG00000167968 | DNASE1L2 | turquoise |
| ENSG00000167972 | ABCA3 | turquoise |
| ENSG00000167977 | KCTD5 | turquoise |
| ENSG00000167978 | SRRM2 | turquoise |
| ENSG00000167981 | ZNF597 | turquoise |
| ENSG00000167996 | FTH1 | turquoise |
| ENSG00000168000 | BSCL2 | turquoise |
| ENSG00000168036 | CTNNB1 | turquoise |
| ENSG00000168040 | FADD | turquoise |
| ENSG00000168056 | LTBP3 | turquoise |
| ENSG00000168060 | NAALADL1 | turquoise |
| ENSG00000168066 | SF1 | turquoise |
| ENSG00000168067 | MAP4K2 | turquoise |
| ENSG00000168071 | CCDC88B | turquoise |
| ENSG00000168092 | PAFAH1B2 | turquoise |
| ENSG00000168116 | KIAA1586 | turquoise |
| ENSG00000168118 | RAB4A | turquoise |
| ENSG00000168137 | SETD5 | turquoise |
| ENSG00000168152 | THAP9 | turquoise |
| ENSG00000168172 | HOOK3 | turquoise |
| ENSG00000168175 | MAPK1IP1L | turquoise |
| ENSG00000168216 | LMBRD1 | turquoise |
| ENSG00000168228 | ZCCHC4 | turquoise |
| ENSG00000168237 | GLYCTK | turquoise |
| ENSG00000168246 | UBTD2 | turquoise |
| ENSG00000168255 | POLR2J3 | turquoise |
| ENSG00000168264 | IRF2BP2 | turquoise |
| ENSG00000168268 | NT5DC2 | turquoise |
| ENSG00000168280 | KIF5C | turquoise |
| ENSG00000168282 | MGAT2 | turquoise |
| ENSG00000168283 | BMI1 | turquoise |
| ENSG00000168288 | MMADHC | turquoise |
| ENSG00000168300 | PCMTD1 | turquoise |
| ENSG00000168301 | KCTD6 | turquoise |
| ENSG00000168303 | MPLKIP | turquoise |
| ENSG00000168306 | ACOX2 | turquoise |
| ENSG00000168350 | DEGS2 | turquoise |
| ENSG00000168374 | ARF4 | turquoise |
| ENSG00000168385 | 2-Sep | turquoise |
| ENSG00000168389 | MFSD2A | turquoise |
| ENSG00000168397 | ATG4B | turquoise |

| | | |
|---|---|---|
| ENSG00000168405 | CMAHP | turquoise |
| ENSG00000168411 | RFWD3 | turquoise |
| ENSG00000168438 | CDC40 | turquoise |
| ENSG00000168487 | BMP1 | turquoise |
| ENSG00000168488 | ATXN2L | turquoise |
| ENSG00000168495 | POLR3D | turquoise |
| ENSG00000168497 | CAVIN2 | turquoise |
| ENSG00000168502 | MTCL1 | turquoise |
| ENSG00000168517 | HEXIM2 | turquoise |
| ENSG00000168522 | FNTA | turquoise |
| ENSG00000168528 | SERINC2 | turquoise |
| ENSG00000168538 | TRAPPC11 | turquoise |
| ENSG00000168546 | GFRA2 | turquoise |
| ENSG00000168556 | ING2 | turquoise |
| ENSG00000168564 | CDKN2AIP | turquoise |
| ENSG00000168566 | SNRNP48 | turquoise |
| ENSG00000168569 | TMEM223 | turquoise |
| ENSG00000168612 | ZSWIM1 | turquoise |
| ENSG00000168646 | AXIN2 | turquoise |
| ENSG00000168661 | ZNF30 | turquoise |
| ENSG00000168685 | IL7R | turquoise |
| ENSG00000168710 | AHCYL1 | turquoise |
| ENSG00000168724 | DNAJC21 | turquoise |
| ENSG00000168754 | FAM178B | turquoise |
| ENSG00000168758 | SEMA4C | turquoise |
| ENSG00000168765 | GSTM4 | turquoise |
| ENSG00000168769 | TET2 | turquoise |
| ENSG00000168795 | ZBTB5 | turquoise |
| ENSG00000168802 | CHTF8 | turquoise |
| ENSG00000168803 | ADAL | turquoise |
| ENSG00000168806 | LCMT2 | turquoise |
| ENSG00000168813 | ZNF507 | turquoise |
| ENSG00000168826 | ZBTB49 | turquoise |
| ENSG00000168827 | GFM1 | turquoise |
| ENSG00000168876 | ANKRD49 | turquoise |
| ENSG00000168884 | TNIP2 | turquoise |
| ENSG00000168887 | C2orf68 | turquoise |
| ENSG00000168890 | TMEM150A | turquoise |
| ENSG00000168904 | LRRC28 | turquoise |
| ENSG00000168913 | ENHO | turquoise |
| ENSG00000168924 | LETM1 | turquoise |
| ENSG00000168944 | CEP120 | turquoise |
| ENSG00000168958 | MFF | turquoise |
| ENSG00000168993 | CPLX1 | turquoise |
| ENSG00000168994 | PXDC1 | turquoise |
| ENSG00000169016 | E2F6 | turquoise |
| ENSG00000169018 | FEM1B | turquoise |
| ENSG00000169019 | COMMD8 | turquoise |

| | | |
|---|---|---|
| ENSG00000169032 | MAP2K1 | turquoise |
| ENSG00000169045 | HNRNPH1 | turquoise |
| ENSG00000169047 | IRS1 | turquoise |
| ENSG00000169057 | MECP2 | turquoise |
| ENSG00000169062 | UPF3A | turquoise |
| ENSG00000169093 | ASMTL | turquoise |
| ENSG00000169118 | CSNK1G1 | turquoise |
| ENSG00000169129 | AFAP1L2 | turquoise |
| ENSG00000169131 | ZNF354A | turquoise |
| ENSG00000169139 | UBE2V2 | turquoise |
| ENSG00000169155 | ZBTB43 | turquoise |
| ENSG00000169184 | MN1 | turquoise |
| ENSG00000169193 | CCDC126 | turquoise |
| ENSG00000169203 | NPIPB12 | turquoise |
| ENSG00000169217 | CD2BP2 | turquoise |
| ENSG00000169220 | RGS14 | turquoise |
| ENSG00000169221 | TBC1D10B | turquoise |
| ENSG00000169223 | LMAN2 | turquoise |
| ENSG00000169224 | GCSAML | turquoise |
| ENSG00000169231 | THBS3 | turquoise |
| ENSG00000169246 | NPIPB3 | turquoise |
| ENSG00000169251 | NMD3 | turquoise |
| ENSG00000169252 | ADRB2 | turquoise |
| ENSG00000169255 | B3GALNT1 | turquoise |
| ENSG00000169258 | GPRIN1 | turquoise |
| ENSG00000169282 | KCNAB1 | turquoise |
| ENSG00000169288 | MRPL1 | turquoise |
| ENSG00000169299 | PGM2 | turquoise |
| ENSG00000169313 | P2RY12 | turquoise |
| ENSG00000169330 | KIAA1024 | turquoise |
| ENSG00000169359 | SLC33A1 | turquoise |
| ENSG00000169375 | SIN3A | turquoise |
| ENSG00000169379 | ARL13B | turquoise |
| ENSG00000169398 | PTK2 | turquoise |
| ENSG00000169410 | PTPN9 | turquoise |
| ENSG00000169429 | CXCL8 | turquoise |
| ENSG00000169442 | CD52 | turquoise |
| ENSG00000169446 | MMGT1 | turquoise |
| ENSG00000169490 | TM2D2 | turquoise |
| ENSG00000169499 | PLEKHA2 | turquoise |
| ENSG00000169504 | CLIC4 | turquoise |
| ENSG00000169507 | SLC38A11 | turquoise |
| ENSG00000169519 | METTL15 | turquoise |
| ENSG00000169554 | ZEB2 | turquoise |
| ENSG00000169567 | HINT1 | turquoise |
| ENSG00000169570 | DTWD2 | turquoise |
| ENSG00000169592 | INO80E | turquoise |
| ENSG00000169598 | DFFB | turquoise |

| | | |
|---|---|---|
| ENSG00000169599 | NFU1 | turquoise |
| ENSG00000169612 | FAM103A1 | turquoise |
| ENSG00000169621 | APLF | turquoise |
| ENSG00000169641 | LUZP1 | turquoise |
| ENSG00000169660 | HEXDC | turquoise |
| ENSG00000169679 | BUB1 | turquoise |
| ENSG00000169692 | AGPAT2 | turquoise |
| ENSG00000169714 | CNBP | turquoise |
| ENSG00000169740 | ZNF32 | turquoise |
| ENSG00000169750 | RAC3 | turquoise |
| ENSG00000169752 | NRG4 | turquoise |
| ENSG00000169756 | LIMS1 | turquoise |
| ENSG00000169762 | TAPT1 | turquoise |
| ENSG00000169764 | UGP2 | turquoise |
| ENSG00000169813 | HNRNPF | turquoise |
| ENSG00000169814 | BTD | turquoise |
| ENSG00000169826 | CSGALNACT2 | turquoise |
| ENSG00000169855 | ROBO1 | turquoise |
| ENSG00000169860 | P2RY1 | turquoise |
| ENSG00000169885 | CALML6 | turquoise |
| ENSG00000169895 | SYAP1 | turquoise |
| ENSG00000169914 | OTUD3 | turquoise |
| ENSG00000169925 | BRD3 | turquoise |
| ENSG00000169926 | KLF13 | turquoise |
| ENSG00000169957 | ZNF768 | turquoise |
| ENSG00000169962 | TAS1R3 | turquoise |
| ENSG00000169967 | MAP3K2 | turquoise |
| ENSG00000169972 | PUSL1 | turquoise |
| ENSG00000169994 | MYO7B | turquoise |
| ENSG00000170017 | ALCAM | turquoise |
| ENSG00000170027 | YWHAG | turquoise |
| ENSG00000170035 | UBE2E3 | turquoise |
| ENSG00000170049 | KCNAB3 | turquoise |
| ENSG00000170074 | FAM153A | turquoise |
| ENSG00000170085 | SIMC1 | turquoise |
| ENSG00000170088 | TMEM192 | turquoise |
| ENSG00000170113 | NIPA1 | turquoise |
| ENSG00000170128 | GPR25 | turquoise |
| ENSG00000170142 | UBE2E1 | turquoise |
| ENSG00000170144 | HNRNPA3 | turquoise |
| ENSG00000170145 | SIK2 | turquoise |
| ENSG00000170185 | USP38 | turquoise |
| ENSG00000170190 | SLC16A5 | turquoise |
| ENSG00000170191 | NANP | turquoise |
| ENSG00000170209 | ANKK1 | turquoise |
| ENSG00000170222 | ADPRM | turquoise |
| ENSG00000170234 | PWWP2A | turquoise |
| ENSG00000170242 | USP47 | turquoise |

| | | |
|---|---|---|
| ENSG00000170248 | PDCD6IP | turquoise |
| ENSG00000170264 | FAM161A | turquoise |
| ENSG00000170265 | ZNF282 | turquoise |
| ENSG00000170293 | CMTM8 | turquoise |
| ENSG00000170296 | GABARAP | turquoise |
| ENSG00000170312 | CDK1 | turquoise |
| ENSG00000170322 | NFRKB | turquoise |
| ENSG00000170325 | PRDM10 | turquoise |
| ENSG00000170340 | B3GNT2 | turquoise |
| ENSG00000170345 | FOS | turquoise |
| ENSG00000170348 | TMED10 | turquoise |
| ENSG00000170364 | SETMAR | turquoise |
| ENSG00000170365 | SMAD1 | turquoise |
| ENSG00000170379 | TCAF2 | turquoise |
| ENSG00000170385 | SLC30A1 | turquoise |
| ENSG00000170417 | TMEM182 | turquoise |
| ENSG00000170425 | ADORA2B | turquoise |
| ENSG00000170445 | HARS | turquoise |
| ENSG00000170448 | NFXL1 | turquoise |
| ENSG00000170456 | DENND5B | turquoise |
| ENSG00000170469 | SPATA24 | turquoise |
| ENSG00000170471 | RALGAPB | turquoise |
| ENSG00000170502 | NUDT9 | turquoise |
| ENSG00000170522 | ELOVL6 | turquoise |
| ENSG00000170525 | PFKFB3 | turquoise |
| ENSG00000170540 | ARL6IP1 | turquoise |
| ENSG00000170542 | SERPINB9 | turquoise |
| ENSG00000170545 | SMAGP | turquoise |
| ENSG00000170558 | CDH2 | turquoise |
| ENSG00000170584 | NUDCD2 | turquoise |
| ENSG00000170604 | IRF2BP1 | turquoise |
| ENSG00000170606 | HSPA4 | turquoise |
| ENSG00000170619 | COMMD5 | turquoise |
| ENSG00000170632 | ARMC10 | turquoise |
| ENSG00000170633 | RNF34 | turquoise |
| ENSG00000170638 | TRABD | turquoise |
| ENSG00000170647 | TMEM133 | turquoise |
| ENSG00000170653 | ATF7 | turquoise |
| ENSG00000170677 | SOCS6 | turquoise |
| ENSG00000170684 | ZNF296 | turquoise |
| ENSG00000170759 | KIF5B | turquoise |
| ENSG00000170776 | AKAP13 | turquoise |
| ENSG00000170779 | CDCA4 | turquoise |
| ENSG00000170791 | CHCHD7 | turquoise |
| ENSG00000170802 | FOXN2 | turquoise |
| ENSG00000170832 | USP32 | turquoise |
| ENSG00000170835 | CEL | turquoise |
| ENSG00000170836 | PPM1D | turquoise |

| | | |
|---|---|---|
| ENSG00000170837 | GPR27 | turquoise |
| ENSG00000170846 | AC093323.1 | turquoise |
| ENSG00000170852 | KBTBD2 | turquoise |
| ENSG00000170854 | RIOX2 | turquoise |
| ENSG00000170855 | TRIAP1 | turquoise |
| ENSG00000170860 | LSM3 | turquoise |
| ENSG00000170871 | KIAA0232 | turquoise |
| ENSG00000170873 | MTSS1 | turquoise |
| ENSG00000170876 | TMEM43 | turquoise |
| ENSG00000170881 | RNF139 | turquoise |
| ENSG00000170903 | MSANTD4 | turquoise |
| ENSG00000170906 | NDUFA3 | turquoise |
| ENSG00000170909 | OSCAR | turquoise |
| ENSG00000170915 | PAQR8 | turquoise |
| ENSG00000170919 | TPT1-AS1 | turquoise |
| ENSG00000170946 | DNAJC24 | turquoise |
| ENSG00000170949 | ZNF160 | turquoise |
| ENSG00000170989 | S1PR1 | turquoise |
| ENSG00000171033 | PKIA | turquoise |
| ENSG00000171045 | TSNARE1 | turquoise |
| ENSG00000171049 | FPR2 | turquoise |
| ENSG00000171055 | FEZ2 | turquoise |
| ENSG00000171067 | C11orf24 | turquoise |
| ENSG00000171084 | FAM86JP | turquoise |
| ENSG00000171097 | KYAT1 | turquoise |
| ENSG00000171100 | MTM1 | turquoise |
| ENSG00000171103 | TRMT61B | turquoise |
| ENSG00000171109 | MFN1 | turquoise |
| ENSG00000171121 | KCNMB3 | turquoise |
| ENSG00000171132 | PRKCE | turquoise |
| ENSG00000171148 | TADA3 | turquoise |
| ENSG00000171150 | SOCS5 | turquoise |
| ENSG00000171155 | C1GALT1C1 | turquoise |
| ENSG00000171160 | MORN4 | turquoise |
| ENSG00000171161 | ZNF672 | turquoise |
| ENSG00000171163 | ZNF692 | turquoise |
| ENSG00000171169 | NAIF1 | turquoise |
| ENSG00000171174 | RBKS | turquoise |
| ENSG00000171202 | TMEM126A | turquoise |
| ENSG00000171204 | TMEM126B | turquoise |
| ENSG00000171208 | NETO2 | turquoise |
| ENSG00000171241 | SHCBP1 | turquoise |
| ENSG00000171246 | NPTX1 | turquoise |
| ENSG00000171262 | FAM98B | turquoise |
| ENSG00000171291 | ZNF439 | turquoise |
| ENSG00000171298 | GAA | turquoise |
| ENSG00000171307 | ZDHHC16 | turquoise |
| ENSG00000171316 | CHD7 | turquoise |

| | | |
|---|---|---|
| ENSG00000171320 | ESCO2 | turquoise |
| ENSG00000171428 | NAT1 | turquoise |
| ENSG00000171448 | ZBTB26 | turquoise |
| ENSG00000171462 | DLK2 | turquoise |
| ENSG00000171467 | ZNF318 | turquoise |
| ENSG00000171469 | ZNF561 | turquoise |
| ENSG00000171488 | LRRC8C | turquoise |
| ENSG00000171492 | LRRC8D | turquoise |
| ENSG00000171497 | PPID | turquoise |
| ENSG00000171502 | COL24A1 | turquoise |
| ENSG00000171503 | ETFDH | turquoise |
| ENSG00000171517 | LPAR3 | turquoise |
| ENSG00000171522 | PTGER4 | turquoise |
| ENSG00000171530 | TBCA | turquoise |
| ENSG00000171566 | PLRG1 | turquoise |
| ENSG00000171574 | ZNF584 | turquoise |
| ENSG00000171611 | PTCRA | turquoise |
| ENSG00000171617 | ENC1 | turquoise |
| ENSG00000171634 | BPTF | turquoise |
| ENSG00000171657 | GPR82 | turquoise |
| ENSG00000171703 | TCEA2 | turquoise |
| ENSG00000171714 | ANO5 | turquoise |
| ENSG00000171723 | GPHN | turquoise |
| ENSG00000171763 | SPATA5L1 | turquoise |
| ENSG00000171786 | NHLH1 | turquoise |
| ENSG00000171791 | BCL2 | turquoise |
| ENSG00000171792 | RHNO1 | turquoise |
| ENSG00000171794 | UTF1 | turquoise |
| ENSG00000171798 | KNDC1 | turquoise |
| ENSG00000171806 | METTL18 | turquoise |
| ENSG00000171813 | PWWP2B | turquoise |
| ENSG00000171817 | ZNF540 | turquoise |
| ENSG00000171824 | EXOSC10 | turquoise |
| ENSG00000171827 | ZNF570 | turquoise |
| ENSG00000171840 | NINJ2 | turquoise |
| ENSG00000171843 | MLLT3 | turquoise |
| ENSG00000171853 | TRAPPC12 | turquoise |
| ENSG00000171861 | MRM3 | turquoise |
| ENSG00000171862 | PTEN | turquoise |
| ENSG00000171863 | RPS7 | turquoise |
| ENSG00000171867 | PRNP | turquoise |
| ENSG00000171928 | TVP23B | turquoise |
| ENSG00000171940 | ZNF217 | turquoise |
| ENSG00000171943 | SRGAP2C | turquoise |
| ENSG00000171953 | ATPAF2 | turquoise |
| ENSG00000171970 | ZNF57 | turquoise |
| ENSG00000171988 | JMJD1C | turquoise |
| ENSG00000171992 | SYNPO | turquoise |

| | | |
|---|---|---|
| ENSG00000172007 | RAB33B | turquoise |
| ENSG00000172009 | THOP1 | turquoise |
| ENSG00000172037 | LAMB2 | turquoise |
| ENSG00000172059 | KLF11 | turquoise |
| ENSG00000172071 | EIF2AK3 | turquoise |
| ENSG00000172086 | KRCC1 | turquoise |
| ENSG00000172113 | NME6 | turquoise |
| ENSG00000172115 | CYCS | turquoise |
| ENSG00000172116 | CD8B | turquoise |
| ENSG00000172123 | SLFN12 | turquoise |
| ENSG00000172164 | SNTB1 | turquoise |
| ENSG00000172167 | MTBP | turquoise |
| ENSG00000172172 | MRPL13 | turquoise |
| ENSG00000172175 | MALT1 | turquoise |
| ENSG00000172197 | MBOAT1 | turquoise |
| ENSG00000172236 | TPSAB1 | turquoise |
| ENSG00000172239 | PAIP1 | turquoise |
| ENSG00000172243 | CLEC7A | turquoise |
| ENSG00000172247 | C1QTNF4 | turquoise |
| ENSG00000172250 | SERHL | turquoise |
| ENSG00000172262 | ZNF131 | turquoise |
| ENSG00000172264 | MACROD2 | turquoise |
| ENSG00000172273 | HINFP | turquoise |
| ENSG00000172292 | CERS6 | turquoise |
| ENSG00000172296 | SPTLC3 | turquoise |
| ENSG00000172322 | CLEC12A | turquoise |
| ENSG00000172339 | ALG14 | turquoise |
| ENSG00000172340 | SUCLG2 | turquoise |
| ENSG00000172345 | STARD5 | turquoise |
| ENSG00000172348 | RCAN2 | turquoise |
| ENSG00000172349 | IL16 | turquoise |
| ENSG00000172366 | MCRIP2 | turquoise |
| ENSG00000172367 | PDZD3 | turquoise |
| ENSG00000172375 | C2CD2L | turquoise |
| ENSG00000172382 | PRSS27 | turquoise |
| ENSG00000172403 | SYNPO2 | turquoise |
| ENSG00000172456 | FGGY | turquoise |
| ENSG00000172458 | IL17D | turquoise |
| ENSG00000172465 | TCEAL1 | turquoise |
| ENSG00000172466 | ZNF24 | turquoise |
| ENSG00000172469 | MANEA | turquoise |
| ENSG00000172493 | AFF1 | turquoise |
| ENSG00000172508 | CARNS1 | turquoise |
| ENSG00000172530 | BANP | turquoise |
| ENSG00000172548 | NIPAL4 | turquoise |
| ENSG00000172575 | RASGRP1 | turquoise |
| ENSG00000172578 | KLHL6 | turquoise |
| ENSG00000172594 | SMPDL3A | turquoise |

| | | |
|---|---|---|
| ENSG00000172602 | RND1 | turquoise |
| ENSG00000172613 | RAD9A | turquoise |
| ENSG00000172638 | EFEMP2 | turquoise |
| ENSG00000172661 | WASHC2C | turquoise |
| ENSG00000172663 | TMEM134 | turquoise |
| ENSG00000172667 | ZMAT3 | turquoise |
| ENSG00000172671 | ZFAND4 | turquoise |
| ENSG00000172716 | SLFN11 | turquoise |
| ENSG00000172725 | CORO1B | turquoise |
| ENSG00000172731 | LRRC20 | turquoise |
| ENSG00000172732 | MUS81 | turquoise |
| ENSG00000172748 | ZNF596 | turquoise |
| ENSG00000172757 | CFL1 | turquoise |
| ENSG00000172765 | TMCC1 | turquoise |
| ENSG00000172766 | NAA16 | turquoise |
| ENSG00000172775 | FAM192A | turquoise |
| ENSG00000172780 | RAB43 | turquoise |
| ENSG00000172785 | CBWD1 | turquoise |
| ENSG00000172794 | RAB37 | turquoise |
| ENSG00000172795 | DCP2 | turquoise |
| ENSG00000172819 | RARG | turquoise |
| ENSG00000172824 | CES4A | turquoise |
| ENSG00000172831 | CES2 | turquoise |
| ENSG00000172845 | SP3 | turquoise |
| ENSG00000172869 | DMXL1 | turquoise |
| ENSG00000172888 | ZNF621 | turquoise |
| ENSG00000172889 | EGFL7 | turquoise |
| ENSG00000172890 | NADSYN1 | turquoise |
| ENSG00000172915 | NBEA | turquoise |
| ENSG00000172927 | MYEOV | turquoise |
| ENSG00000172932 | ANKRD13D | turquoise |
| ENSG00000172939 | OXSR1 | turquoise |
| ENSG00000172954 | LCLAT1 | turquoise |
| ENSG00000172977 | KAT5 | turquoise |
| ENSG00000173011 | TADA2B | turquoise |
| ENSG00000173013 | CCDC96 | turquoise |
| ENSG00000173020 | GRK2 | turquoise |
| ENSG00000173039 | RELA | turquoise |
| ENSG00000173040 | EVC2 | turquoise |
| ENSG00000173041 | ZNF680 | turquoise |
| ENSG00000173085 | COQ2 | turquoise |
| ENSG00000173114 | LRRN3 | turquoise |
| ENSG00000173120 | KDM2A | turquoise |
| ENSG00000173137 | ADCK5 | turquoise |
| ENSG00000173145 | NOC3L | turquoise |
| ENSG00000173153 | ESRRA | turquoise |
| ENSG00000173171 | MTX1 | turquoise |
| ENSG00000173175 | ADCY5 | turquoise |

| | | |
|---|---|---|
| ENSG00000173198 | CYSLTR1 | turquoise |
| ENSG00000173200 | PARP15 | turquoise |
| ENSG00000173207 | CKS1B | turquoise |
| ENSG00000173208 | ABCD2 | turquoise |
| ENSG00000173218 | VANGL1 | turquoise |
| ENSG00000173221 | GLRX | turquoise |
| ENSG00000173226 | IQCB1 | turquoise |
| ENSG00000173230 | GOLGB1 | turquoise |
| ENSG00000173239 | LIPM | turquoise |
| ENSG00000173258 | ZNF483 | turquoise |
| ENSG00000173264 | GPR137 | turquoise |
| ENSG00000173273 | TNKS | turquoise |
| ENSG00000173276 | ZBTB21 | turquoise |
| ENSG00000173281 | PPP1R3B | turquoise |
| ENSG00000173295 | FAM86B3P | turquoise |
| ENSG00000173327 | MAP3K11 | turquoise |
| ENSG00000173334 | TRIB1 | turquoise |
| ENSG00000173338 | KCNK7 | turquoise |
| ENSG00000173401 | GLIPR1L1 | turquoise |
| ENSG00000173406 | DAB1 | turquoise |
| ENSG00000173409 | ARV1 | turquoise |
| ENSG00000173418 | NAA20 | turquoise |
| ENSG00000173451 | THAP2 | turquoise |
| ENSG00000173456 | RNF26 | turquoise |
| ENSG00000173473 | SMARCC1 | turquoise |
| ENSG00000173482 | PTPRM | turquoise |
| ENSG00000173517 | PEAK1 | turquoise |
| ENSG00000173531 | MST1 | turquoise |
| ENSG00000173540 | GMPPB | turquoise |
| ENSG00000173542 | MOB1B | turquoise |
| ENSG00000173548 | SNX33 | turquoise |
| ENSG00000173575 | CHD2 | turquoise |
| ENSG00000173578 | XCR1 | turquoise |
| ENSG00000173585 | CCR9 | turquoise |
| ENSG00000173597 | SULT1B1 | turquoise |
| ENSG00000173598 | NUDT4 | turquoise |
| ENSG00000173599 | PC | turquoise |
| ENSG00000173621 | LRFN4 | turquoise |
| ENSG00000173626 | TRAPPC3L | turquoise |
| ENSG00000173674 | EIF1AX | turquoise |
| ENSG00000173706 | HEG1 | turquoise |
| ENSG00000173715 | C11orf80 | turquoise |
| ENSG00000173726 | TOMM20 | turquoise |
| ENSG00000173744 | AGFG1 | turquoise |
| ENSG00000173809 | TDRD12 | turquoise |
| ENSG00000173811 | CCDC13-AS1 | turquoise |
| ENSG00000173818 | ENDOV | turquoise |
| ENSG00000173825 | TIGD3 | turquoise |

| | | |
|---|---|---|
| ENSG00000173838 | 10-Mar | turquoise |
| ENSG00000173846 | PLK3 | turquoise |
| ENSG00000173848 | NET1 | turquoise |
| ENSG00000173852 | DPY19L1 | turquoise |
| ENSG00000173868 | PHOSPHO1 | turquoise |
| ENSG00000173890 | GPR160 | turquoise |
| ENSG00000173894 | CBX2 | turquoise |
| ENSG00000173898 | SPTBN2 | turquoise |
| ENSG00000173905 | GOLIM4 | turquoise |
| ENSG00000173914 | RBM4B | turquoise |
| ENSG00000173915 | USMG5 | turquoise |
| ENSG00000173930 | SLCO4C1 | turquoise |
| ENSG00000173947 | PIFO | turquoise |
| ENSG00000173950 | XXYLT1 | turquoise |
| ENSG00000173960 | UBXN2A | turquoise |
| ENSG00000173991 | TCAP | turquoise |
| ENSG00000174007 | CEP19 | turquoise |
| ENSG00000174010 | KLHL15 | turquoise |
| ENSG00000174013 | FBXO45 | turquoise |
| ENSG00000174032 | SLC25A30 | turquoise |
| ENSG00000174059 | CD34 | turquoise |
| ENSG00000174080 | CTSF | turquoise |
| ENSG00000174099 | MSRB3 | turquoise |
| ENSG00000174106 | LEMD3 | turquoise |
| ENSG00000174123 | TLR10 | turquoise |
| ENSG00000174132 | FAM174A | turquoise |
| ENSG00000174165 | ZDHHC24 | turquoise |
| ENSG00000174171 | AC020659.1 | turquoise |
| ENSG00000174173 | TRMT10C | turquoise |
| ENSG00000174175 | SELP | turquoise |
| ENSG00000174197 | MGA | turquoise |
| ENSG00000174206 | C12orf66 | turquoise |
| ENSG00000174233 | ADCY6 | turquoise |
| ENSG00000174326 | SLC16A11 | turquoise |
| ENSG00000174327 | SLC16A13 | turquoise |
| ENSG00000174365 | SNHG11 | turquoise |
| ENSG00000174405 | LIG4 | turquoise |
| ENSG00000174437 | ATP2A2 | turquoise |
| ENSG00000174442 | ZWILCH | turquoise |
| ENSG00000174446 | SNAPC5 | turquoise |
| ENSG00000174456 | C12orf76 | turquoise |
| ENSG00000174485 | DENND4A | turquoise |
| ENSG00000174500 | GCSAM | turquoise |
| ENSG00000174514 | MFSD4A | turquoise |
| ENSG00000174516 | PELI3 | turquoise |
| ENSG00000174527 | MYO1H | turquoise |
| ENSG00000174564 | IL20RB | turquoise |
| ENSG00000174574 | AKIRIN1 | turquoise |

| | | |
|---|---|---|
| ENSG00000174579 | MSL2 | turquoise |
| ENSG00000174606 | ANGEL2 | turquoise |
| ENSG00000174607 | UGT8 | turquoise |
| ENSG00000174652 | ZNF266 | turquoise |
| ENSG00000174669 | SLC29A2 | turquoise |
| ENSG00000174695 | TMEM167A | turquoise |
| ENSG00000174718 | KIAA1551 | turquoise |
| ENSG00000174720 | LARP7 | turquoise |
| ENSG00000174738 | NR1D2 | turquoise |
| ENSG00000174744 | BRMS1 | turquoise |
| ENSG00000174749 | C4orf32 | turquoise |
| ENSG00000174780 | SRP72 | turquoise |
| ENSG00000174788 | PCP2 | turquoise |
| ENSG00000174799 | CEP135 | turquoise |
| ENSG00000174839 | DENND6A | turquoise |
| ENSG00000174840 | PDE12 | turquoise |
| ENSG00000174842 | GLMN | turquoise |
| ENSG00000174871 | CNIH2 | turquoise |
| ENSG00000174886 | NDUFA11 | turquoise |
| ENSG00000174891 | RSRC1 | turquoise |
| ENSG00000174903 | RAB1B | turquoise |
| ENSG00000174912 | METTL15P1 | turquoise |
| ENSG00000174915 | PTDSS2 | turquoise |
| ENSG00000174928 | C3orf33 | turquoise |
| ENSG00000174943 | KCTD13 | turquoise |
| ENSG00000174944 | P2RY14 | turquoise |
| ENSG00000174946 | GPR171 | turquoise |
| ENSG00000174953 | DHX36 | turquoise |
| ENSG00000174977 | AC026271.1 | turquoise |
| ENSG00000174996 | KLC2 | turquoise |
| ENSG00000175029 | CTBP2 | turquoise |
| ENSG00000175048 | ZDHHC14 | turquoise |
| ENSG00000175054 | ATR | turquoise |
| ENSG00000175061 | LRRC75A-AS1 | turquoise |
| ENSG00000175063 | UBE2C | turquoise |
| ENSG00000175073 | VCPIP1 | turquoise |
| ENSG00000175087 | PDIK1L | turquoise |
| ENSG00000175104 | TRAF6 | turquoise |
| ENSG00000175105 | ZNF654 | turquoise |
| ENSG00000175110 | MRPS22 | turquoise |
| ENSG00000175115 | PACS1 | turquoise |
| ENSG00000175155 | YPEL2 | turquoise |
| ENSG00000175164 | ABO | turquoise |
| ENSG00000175170 | FAM182B | turquoise |
| ENSG00000175197 | DDIT3 | turquoise |
| ENSG00000175203 | DCTN2 | turquoise |
| ENSG00000175206 | NPPA | turquoise |
| ENSG00000175213 | ZNF408 | turquoise |

| | | |
|---|---|---|
| ENSG00000175216 | CKAP5 | turquoise |
| ENSG00000175221 | MED16 | turquoise |
| ENSG00000175224 | ATG13 | turquoise |
| ENSG00000175262 | C1orf127 | turquoise |
| ENSG00000175265 | GOLGA8A | turquoise |
| ENSG00000175279 | CENPS | turquoise |
| ENSG00000175287 | PHYHD1 | turquoise |
| ENSG00000175309 | PHYKPL | turquoise |
| ENSG00000175322 | ZNF519 | turquoise |
| ENSG00000175324 | LSM1 | turquoise |
| ENSG00000175348 | TMEM9B | turquoise |
| ENSG00000175354 | PTPN2 | turquoise |
| ENSG00000175387 | SMAD2 | turquoise |
| ENSG00000175395 | ZNF25 | turquoise |
| ENSG00000175449 | RFESD | turquoise |
| ENSG00000175455 | CCDC14 | turquoise |
| ENSG00000175463 | TBC1D10C | turquoise |
| ENSG00000175470 | PPP2R2D | turquoise |
| ENSG00000175471 | MCTP1 | turquoise |
| ENSG00000175482 | POLD4 | turquoise |
| ENSG00000175513 | TSGA10IP | turquoise |
| ENSG00000175536 | LIPT2 | turquoise |
| ENSG00000175538 | KCNE3 | turquoise |
| ENSG00000175548 | ALG10B | turquoise |
| ENSG00000175564 | UCP3 | turquoise |
| ENSG00000175573 | C11orf68 | turquoise |
| ENSG00000175581 | MRPL48 | turquoise |
| ENSG00000175582 | RAB6A | turquoise |
| ENSG00000175591 | P2RY2 | turquoise |
| ENSG00000175595 | ERCC4 | turquoise |
| ENSG00000175602 | CCDC85B | turquoise |
| ENSG00000175606 | TMEM70 | turquoise |
| ENSG00000175634 | RPS6KB2 | turquoise |
| ENSG00000175662 | TOM1L2 | turquoise |
| ENSG00000175691 | ZNF77 | turquoise |
| ENSG00000175711 | B3GNTL1 | turquoise |
| ENSG00000175730 | BAK1P1 | turquoise |
| ENSG00000175741 | RWDD4P2 | turquoise |
| ENSG00000175768 | TOMM5 | turquoise |
| ENSG00000175793 | SFN | turquoise |
| ENSG00000175800 | OR52B3P | turquoise |
| ENSG00000175857 | GAPT | turquoise |
| ENSG00000175866 | BAIAP2 | turquoise |
| ENSG00000175874 | CREG2 | turquoise |
| ENSG00000175893 | ZDHHC21 | turquoise |
| ENSG00000175895 | PLEKHF2 | turquoise |
| ENSG00000175906 | ARL4D | turquoise |
| ENSG00000175920 | DOK7 | turquoise |

| | | |
|---|---|---|
| ENSG00000175928 | LRRN1 | turquoise |
| ENSG00000175931 | UBE2O | turquoise |
| ENSG00000175938 | ORAI3 | turquoise |
| ENSG00000176018 | LYSMD3 | turquoise |
| ENSG00000176024 | ZNF613 | turquoise |
| ENSG00000176049 | JAKMIP2 | turquoise |
| ENSG00000176054 | RPL23P2 | turquoise |
| ENSG00000176055 | MBLAC2 | turquoise |
| ENSG00000176092 | CRYBG2 | turquoise |
| ENSG00000176102 | CSTF3 | turquoise |
| ENSG00000176105 | YES1 | turquoise |
| ENSG00000176108 | CHMP6 | turquoise |
| ENSG00000176125 | UFSP1 | turquoise |
| ENSG00000176142 | TMEM39A | turquoise |
| ENSG00000176155 | CCDC57 | turquoise |
| ENSG00000176160 | HSF5 | turquoise |
| ENSG00000176170 | SPHK1 | turquoise |
| ENSG00000176171 | BNIP3 | turquoise |
| ENSG00000176182 | MYPOP | turquoise |
| ENSG00000176209 | SMIM19 | turquoise |
| ENSG00000176222 | ZNF404 | turquoise |
| ENSG00000176225 | RTTN | turquoise |
| ENSG00000176248 | ANAPC2 | turquoise |
| ENSG00000176261 | ZBTB8OS | turquoise |
| ENSG00000176268 | CYCSP34 | turquoise |
| ENSG00000176273 | SLC35G1 | turquoise |
| ENSG00000176293 | ZNF135 | turquoise |
| ENSG00000176383 | B3GNT4 | turquoise |
| ENSG00000176386 | CDC26 | turquoise |
| ENSG00000176390 | CRLF3 | turquoise |
| ENSG00000176410 | DNAJC30 | turquoise |
| ENSG00000176422 | SPRYD4 | turquoise |
| ENSG00000176490 | DIRAS1 | turquoise |
| ENSG00000176533 | GNG7 | turquoise |
| ENSG00000176542 | USF3 | turquoise |
| ENSG00000176597 | B3GNT5 | turquoise |
| ENSG00000176623 | RMDN1 | turquoise |
| ENSG00000176624 | MEX3C | turquoise |
| ENSG00000176659 | C20orf197 | turquoise |
| ENSG00000176681 | LRRC37A | turquoise |
| ENSG00000176700 | SCAND2P | turquoise |
| ENSG00000176714 | CCDC121 | turquoise |
| ENSG00000176715 | ACSF3 | turquoise |
| ENSG00000176732 | PFN4 | turquoise |
| ENSG00000176749 | CDK5R1 | turquoise |
| ENSG00000176761 | ZNF285B | turquoise |
| ENSG00000176783 | RUFY1 | turquoise |
| ENSG00000176809 | LRRC37A3 | turquoise |

| | | |
|---|---|---|
| ENSG00000176834 | VSIG10 | turquoise |
| ENSG00000176845 | METRNL | turquoise |
| ENSG00000176853 | FAM91A1 | turquoise |
| ENSG00000176871 | WSB2 | turquoise |
| ENSG00000176894 | PXMP2 | turquoise |
| ENSG00000176896 | TCEANC | turquoise |
| ENSG00000176903 | PNMA1 | turquoise |
| ENSG00000176909 | MAMSTR | turquoise |
| ENSG00000176919 | C8G | turquoise |
| ENSG00000176928 | GCNT4 | turquoise |
| ENSG00000176986 | SEC24C | turquoise |
| ENSG00000177000 | MTHFR | turquoise |
| ENSG00000177025 | C19orf18 | turquoise |
| ENSG00000177034 | MTX3 | turquoise |
| ENSG00000177042 | TMEM80 | turquoise |
| ENSG00000177051 | FBXO46 | turquoise |
| ENSG00000177054 | ZDHHC13 | turquoise |
| ENSG00000177058 | SLC38A9 | turquoise |
| ENSG00000177106 | EPS8L2 | turquoise |
| ENSG00000177112 | MRVI1-AS1 | turquoise |
| ENSG00000177119 | ANO6 | turquoise |
| ENSG00000177125 | ZBTB34 | turquoise |
| ENSG00000177138 | FAM9B | turquoise |
| ENSG00000177150 | FAM210A | turquoise |
| ENSG00000177189 | RPS6KA3 | turquoise |
| ENSG00000177200 | CHD9 | turquoise |
| ENSG00000177225 | GATD1 | turquoise |
| ENSG00000177239 | MAN1B1 | turquoise |
| ENSG00000177272 | KCNA3 | turquoise |
| ENSG00000177283 | FZD8 | turquoise |
| ENSG00000177302 | TOP3A | turquoise |
| ENSG00000177303 | CASKIN2 | turquoise |
| ENSG00000177311 | ZBTB38 | turquoise |
| ENSG00000177337 | DLGAP1-AS1 | turquoise |
| ENSG00000177352 | CCDC71 | turquoise |
| ENSG00000177380 | PPFIA3 | turquoise |
| ENSG00000177383 | MAGEF1 | turquoise |
| ENSG00000177398 | UMODL1 | turquoise |
| ENSG00000177425 | PAWR | turquoise |
| ENSG00000177426 | TGIF1 | turquoise |
| ENSG00000177427 | MIEF2 | turquoise |
| ENSG00000177432 | NAP1L5 | turquoise |
| ENSG00000177479 | ARIH2 | turquoise |
| ENSG00000177485 | ZBTB33 | turquoise |
| ENSG00000177535 | OR2B11 | turquoise |
| ENSG00000177548 | RABEP2 | turquoise |
| ENSG00000177556 | ATOX1 | turquoise |
| ENSG00000177565 | TBL1XR1 | turquoise |

| | | |
|---|---|---|
| ENSG00000177570 | SAMD12 | turquoise |
| ENSG00000177575 | CD163 | turquoise |
| ENSG00000177595 | PIDD1 | turquoise |
| ENSG00000177599 | ZNF491 | turquoise |
| ENSG00000177613 | CSTF2T | turquoise |
| ENSG00000177614 | PGBD5 | turquoise |
| ENSG00000177628 | GBA | turquoise |
| ENSG00000177666 | PNPLA2 | turquoise |
| ENSG00000177674 | AGTRAP | turquoise |
| ENSG00000177675 | CD163L1 | turquoise |
| ENSG00000177683 | THAP5 | turquoise |
| ENSG00000177707 | NECTIN3 | turquoise |
| ENSG00000177710 | SLC35G5 | turquoise |
| ENSG00000177728 | TMEM94 | turquoise |
| ENSG00000177732 | SOX12 | turquoise |
| ENSG00000177853 | ZNF518A | turquoise |
| ENSG00000177855 | CACYBPP2 | turquoise |
| ENSG00000177879 | AP3S1 | turquoise |
| ENSG00000177888 | ZBTB41 | turquoise |
| ENSG00000177889 | UBE2N | turquoise |
| ENSG00000177917 | ARL6IP6 | turquoise |
| ENSG00000177932 | ZNF354C | turquoise |
| ENSG00000177943 | MAMDC4 | turquoise |
| ENSG00000177946 | CENPBD1 | turquoise |
| ENSG00000177951 | BET1L | turquoise |
| ENSG00000177954 | RPS27 | turquoise |
| ENSG00000177963 | RIC8A | turquoise |
| ENSG00000177981 | ASB8 | turquoise |
| ENSG00000178026 | LRRC75B | turquoise |
| ENSG00000178028 | DMAP1 | turquoise |
| ENSG00000178033 | FAM26E | turquoise |
| ENSG00000178053 | MLF1 | turquoise |
| ENSG00000178074 | C2orf69 | turquoise |
| ENSG00000178082 | AC005412.1 | turquoise |
| ENSG00000178093 | TSSK6 | turquoise |
| ENSG00000178105 | DDX10 | turquoise |
| ENSG00000178149 | DALRD3 | turquoise |
| ENSG00000178162 | FAR2P2 | turquoise |
| ENSG00000178163 | ZNF518B | turquoise |
| ENSG00000178175 | ZNF366 | turquoise |
| ENSG00000178177 | LCORL | turquoise |
| ENSG00000178188 | SH2B1 | turquoise |
| ENSG00000178199 | ZC3H12D | turquoise |
| ENSG00000178202 | KDELC2 | turquoise |
| ENSG00000178209 | PLEC | turquoise |
| ENSG00000178222 | RNF212 | turquoise |
| ENSG00000178226 | PRSS36 | turquoise |
| ENSG00000178234 | GALNT11 | turquoise |

| | | |
|---|---|---|
| ENSG00000178252 | WDR6 | turquoise |
| ENSG00000178301 | AQP11 | turquoise |
| ENSG00000178307 | TMEM11 | turquoise |
| ENSG00000178397 | FAM220A | turquoise |
| ENSG00000178425 | NT5DC1 | turquoise |
| ENSG00000178429 | RPS3AP5 | turquoise |
| ENSG00000178467 | P4HTM | turquoise |
| ENSG00000178498 | DTX3 | turquoise |
| ENSG00000178537 | SLC25A20 | turquoise |
| ENSG00000178538 | CA8 | turquoise |
| ENSG00000178562 | CD28 | turquoise |
| ENSG00000178573 | MAF | turquoise |
| ENSG00000178585 | CTNNBIP1 | turquoise |
| ENSG00000178607 | ERN1 | turquoise |
| ENSG00000178623 | GPR35 | turquoise |
| ENSG00000178691 | SUZ12 | turquoise |
| ENSG00000178694 | NSUN3 | turquoise |
| ENSG00000178695 | KCTD12 | turquoise |
| ENSG00000178700 | DHFR2 | turquoise |
| ENSG00000178715 | AL450998.1 | turquoise |
| ENSG00000178718 | RPP25 | turquoise |
| ENSG00000178719 | GRINA | turquoise |
| ENSG00000178732 | GP5 | turquoise |
| ENSG00000178773 | CPNE7 | turquoise |
| ENSG00000178789 | CD300LB | turquoise |
| ENSG00000178826 | TMEM139 | turquoise |
| ENSG00000178878 | APOLD1 | turquoise |
| ENSG00000178913 | TAF7 | turquoise |
| ENSG00000178950 | GAK | turquoise |
| ENSG00000178951 | ZBTB7A | turquoise |
| ENSG00000178966 | RMI1 | turquoise |
| ENSG00000178971 | CTC1 | turquoise |
| ENSG00000178974 | FBXO34 | turquoise |
| ENSG00000178988 | MRFAP1L1 | turquoise |
| ENSG00000178996 | SNX18 | turquoise |
| ENSG00000179010 | MRFAP1 | turquoise |
| ENSG00000179021 | C3orf38 | turquoise |
| ENSG00000179057 | IGSF22 | turquoise |
| ENSG00000179088 | C12orf42 | turquoise |
| ENSG00000179094 | PER1 | turquoise |
| ENSG00000179104 | TMTC2 | turquoise |
| ENSG00000179119 | SPTY2D1 | turquoise |
| ENSG00000179134 | SAMD4B | turquoise |
| ENSG00000179144 | GIMAP7 | turquoise |
| ENSG00000179151 | EDC3 | turquoise |
| ENSG00000179152 | TCAIM | turquoise |
| ENSG00000179168 | GGN | turquoise |
| ENSG00000179195 | ZNF664 | turquoise |

| | | |
|---|---|---|
| ENSG00000179241 | LDLRAD3 | turquoise |
| ENSG00000179295 | PTPN11 | turquoise |
| ENSG00000179299 | NSUN7 | turquoise |
| ENSG00000179331 | RAB39A | turquoise |
| ENSG00000179335 | CLK3 | turquoise |
| ENSG00000179344 | HLA-DQB1 | turquoise |
| ENSG00000179361 | ARID3B | turquoise |
| ENSG00000179364 | PACS2 | turquoise |
| ENSG00000179387 | ELMOD2 | turquoise |
| ENSG00000179388 | EGR3 | turquoise |
| ENSG00000179406 | LINC00174 | turquoise |
| ENSG00000179454 | KLHL28 | turquoise |
| ENSG00000179455 | MKRN3 | turquoise |
| ENSG00000179456 | ZBTB18 | turquoise |
| ENSG00000179476 | C14orf28 | turquoise |
| ENSG00000179523 | EIF3J-AS1 | turquoise |
| ENSG00000179532 | DNHD1 | turquoise |
| ENSG00000179542 | SLITRK4 | turquoise |
| ENSG00000179583 | CIITA | turquoise |
| ENSG00000179588 | ZFPM1 | turquoise |
| ENSG00000179593 | ALOX15B | turquoise |
| ENSG00000179598 | PLD6 | turquoise |
| ENSG00000179611 | DGKZP1 | turquoise |
| ENSG00000179630 | LACC1 | turquoise |
| ENSG00000179639 | FCER1A | turquoise |
| ENSG00000179698 | WDR97 | turquoise |
| ENSG00000179832 | MROH1 | turquoise |
| ENSG00000179833 | SERTAD2 | turquoise |
| ENSG00000179841 | AKAP5 | turquoise |
| ENSG00000179846 | NKPD1 | turquoise |
| ENSG00000179859 | AC104581.1 | turquoise |
| ENSG00000179886 | TIGD5 | turquoise |
| ENSG00000179889 | PDXDC1 | turquoise |
| ENSG00000179902 | C1orf194 | turquoise |
| ENSG00000179915 | NRXN1 | turquoise |
| ENSG00000179918 | SEPHS2 | turquoise |
| ENSG00000179922 | ZNF784 | turquoise |
| ENSG00000179934 | CCR8 | turquoise |
| ENSG00000179941 | BBS10 | turquoise |
| ENSG00000179943 | FIZ1 | turquoise |
| ENSG00000179954 | SSC5D | turquoise |
| ENSG00000179965 | ZNF771 | turquoise |
| ENSG00000179981 | TSHZ1 | turquoise |
| ENSG00000179988 | PSTK | turquoise |
| ENSG00000180008 | SOCS4 | turquoise |
| ENSG00000180015 | AC093909.1 | turquoise |
| ENSG00000180035 | ZNF48 | turquoise |
| ENSG00000180066 | C10orf91 | turquoise |

| | | |
|---|---|---|
| ENSG00000180104 | EXOC3 | turquoise |
| ENSG00000180113 | TDRD6 | turquoise |
| ENSG00000180155 | LYNX1 | turquoise |
| ENSG00000180182 | MED14 | turquoise |
| ENSG00000180190 | TDRP | turquoise |
| ENSG00000180209 | MYLPF | turquoise |
| ENSG00000180211 | FO393411.1 | turquoise |
| ENSG00000180228 | PRKRA | turquoise |
| ENSG00000180233 | ZNRF2 | turquoise |
| ENSG00000180257 | ZNF816 | turquoise |
| ENSG00000180329 | CCDC43 | turquoise |
| ENSG00000180346 | TIGD2 | turquoise |
| ENSG00000180357 | ZNF609 | turquoise |
| ENSG00000180370 | PAK2 | turquoise |
| ENSG00000180376 | CCDC66 | turquoise |
| ENSG00000180398 | MCFD2 | turquoise |
| ENSG00000180422 | LINC00304 | turquoise |
| ENSG00000180425 | C11orf71 | turquoise |
| ENSG00000180448 | ARHGAP45 | turquoise |
| ENSG00000180479 | ZNF571 | turquoise |
| ENSG00000180488 | MIGA1 | turquoise |
| ENSG00000180530 | NRIP1 | turquoise |
| ENSG00000180543 | TSPYL5 | turquoise |
| ENSG00000180549 | FUT7 | turquoise |
| ENSG00000180573 | HIST1H2AC | turquoise |
| ENSG00000180574 | AC068775.1 | turquoise |
| ENSG00000180581 | SRP9P1 | turquoise |
| ENSG00000180626 | ZNF594 | turquoise |
| ENSG00000180667 | YOD1 | turquoise |
| ENSG00000180673 | EXOC5P1 | turquoise |
| ENSG00000180694 | TMEM64 | turquoise |
| ENSG00000180712 | LINC02363 | turquoise |
| ENSG00000180758 | GPR157 | turquoise |
| ENSG00000180769 | WDFY3-AS2 | turquoise |
| ENSG00000180773 | SLC36A4 | turquoise |
| ENSG00000180776 | ZDHHC20 | turquoise |
| ENSG00000180817 | PPA1 | turquoise |
| ENSG00000180855 | ZNF443 | turquoise |
| ENSG00000180867 | PDIA3P1 | turquoise |
| ENSG00000180875 | GREM2 | turquoise |
| ENSG00000180881 | CAPS2 | turquoise |
| ENSG00000180884 | ZNF792 | turquoise |
| ENSG00000180901 | KCTD2 | turquoise |
| ENSG00000180902 | D2HGDH | turquoise |
| ENSG00000180917 | CMTR2 | turquoise |
| ENSG00000180921 | FAM83H | turquoise |
| ENSG00000180938 | ZNF572 | turquoise |
| ENSG00000180953 | ST20 | turquoise |

| | | |
|---|---|---|
| ENSG00000180957 | PITPNB | turquoise |
| ENSG00000180964 | TCEAL8 | turquoise |
| ENSG00000181004 | BBS12 | turquoise |
| ENSG00000181007 | ZFP82 | turquoise |
| ENSG00000181027 | FKRP | turquoise |
| ENSG00000181029 | TRAPPC5 | turquoise |
| ENSG00000181038 | METTL23 | turquoise |
| ENSG00000181061 | HIGD1A | turquoise |
| ENSG00000181104 | F2R | turquoise |
| ENSG00000181135 | ZNF707 | turquoise |
| ENSG00000181163 | NPM1 | turquoise |
| ENSG00000181215 | C4orf50 | turquoise |
| ENSG00000181315 | ZNF322 | turquoise |
| ENSG00000181350 | LRRC75A | turquoise |
| ENSG00000181392 | SYNE4 | turquoise |
| ENSG00000181396 | OGFOD3 | turquoise |
| ENSG00000181467 | RAP2B | turquoise |
| ENSG00000181472 | ZBTB2 | turquoise |
| ENSG00000181513 | ACBD4 | turquoise |
| ENSG00000181523 | SGSH | turquoise |
| ENSG00000181555 | SETD2 | turquoise |
| ENSG00000181610 | MRPS23 | turquoise |
| ENSG00000181634 | TNFSF15 | turquoise |
| ENSG00000181638 | ZFP41 | turquoise |
| ENSG00000181652 | ATG9B | turquoise |
| ENSG00000181666 | HKR1 | turquoise |
| ENSG00000181690 | PLAG1 | turquoise |
| ENSG00000181704 | YIPF6 | turquoise |
| ENSG00000181744 | C3orf58 | turquoise |
| ENSG00000181751 | C5orf30 | turquoise |
| ENSG00000181773 | GPR3 | turquoise |
| ENSG00000181778 | TMEM252 | turquoise |
| ENSG00000181790 | ADGRB1 | turquoise |
| ENSG00000181817 | LSM10 | turquoise |
| ENSG00000181827 | RFX7 | turquoise |
| ENSG00000181830 | SLC35C1 | turquoise |
| ENSG00000181847 | TIGIT | turquoise |
| ENSG00000181873 | IBA57 | turquoise |
| ENSG00000181894 | ZNF329 | turquoise |
| ENSG00000181896 | ZNF101 | turquoise |
| ENSG00000181904 | C5orf24 | turquoise |
| ENSG00000181908 | AP003774.1 | turquoise |
| ENSG00000181915 | ADO | turquoise |
| ENSG00000181929 | PRKAG1 | turquoise |
| ENSG00000181938 | GINS3 | turquoise |
| ENSG00000181991 | MRPS11 | turquoise |
| ENSG00000182004 | SNRPE | turquoise |
| ENSG00000182010 | RTKN2 | turquoise |

| | | |
|---|---|---|
| ENSG00000182048 | TRPC2 | turquoise |
| ENSG00000182057 | OGFRP1 | turquoise |
| ENSG00000182087 | TMEM259 | turquoise |
| ENSG00000182093 | WRB | turquoise |
| ENSG00000182107 | TMEM30B | turquoise |
| ENSG00000182109 | AL365277.1 | turquoise |
| ENSG00000182117 | NOP10 | turquoise |
| ENSG00000182118 | FAM89A | turquoise |
| ENSG00000182134 | TDRKH | turquoise |
| ENSG00000182141 | ZNF708 | turquoise |
| ENSG00000182149 | IST1 | turquoise |
| ENSG00000182150 | ERCC6L2 | turquoise |
| ENSG00000182158 | CREB3L2 | turquoise |
| ENSG00000182175 | RGMA | turquoise |
| ENSG00000182183 | FAM159A | turquoise |
| ENSG00000182196 | ARL6IP4 | turquoise |
| ENSG00000182208 | MOB2 | turquoise |
| ENSG00000182220 | ATP6AP2 | turquoise |
| ENSG00000182230 | FAM153B | turquoise |
| ENSG00000182253 | SYNM | turquoise |
| ENSG00000182272 | B4GALNT4 | turquoise |
| ENSG00000182287 | AP1S2 | turquoise |
| ENSG00000182308 | DCAF4L1 | turquoise |
| ENSG00000182310 | SPACA6 | turquoise |
| ENSG00000182318 | ZSCAN22 | turquoise |
| ENSG00000182324 | KCNJ14 | turquoise |
| ENSG00000182325 | FBXL6 | turquoise |
| ENSG00000182326 | C1S | turquoise |
| ENSG00000182327 | GLTPD2 | turquoise |
| ENSG00000182359 | KBTBD3 | turquoise |
| ENSG00000182378 | PLCXD1 | turquoise |
| ENSG00000182379 | NXPH4 | turquoise |
| ENSG00000182393 | IFNL1 | turquoise |
| ENSG00000182400 | TRAPPC6B | turquoise |
| ENSG00000182446 | NPLOC4 | turquoise |
| ENSG00000182473 | EXOC7 | turquoise |
| ENSG00000182504 | CEP97 | turquoise |
| ENSG00000182552 | RWDD4 | turquoise |
| ENSG00000182566 | CLEC4G | turquoise |
| ENSG00000182568 | SATB1 | turquoise |
| ENSG00000182578 | CSF1R | turquoise |
| ENSG00000182580 | EPHB3 | turquoise |
| ENSG00000182600 | C2orf82 | turquoise |
| ENSG00000182606 | TRAK1 | turquoise |
| ENSG00000182612 | TSPAN10 | turquoise |
| ENSG00000182621 | PLCB1 | turquoise |
| ENSG00000182628 | SKA2 | turquoise |
| ENSG00000182685 | BRICD5 | turquoise |

| | | |
|---|---|---|
| ENSG00000182700 | IGIP | turquoise |
| ENSG00000182774 | RPS17 | turquoise |
| ENSG00000182796 | TMEM198B | turquoise |
| ENSG00000182827 | ACBD3 | turquoise |
| ENSG00000182831 | C16orf72 | turquoise |
| ENSG00000182858 | ALG12 | turquoise |
| ENSG00000182870 | GALNT9 | turquoise |
| ENSG00000182871 | COL18A1 | turquoise |
| ENSG00000182872 | RBM10 | turquoise |
| ENSG00000182873 | PRKCZ-AS1 | turquoise |
| ENSG00000182902 | SLC25A18 | turquoise |
| ENSG00000182903 | ZNF721 | turquoise |
| ENSG00000182923 | CEP63 | turquoise |
| ENSG00000182952 | HMGN4 | turquoise |
| ENSG00000182957 | SPATA13 | turquoise |
| ENSG00000182986 | ZNF320 | turquoise |
| ENSG00000183018 | SPNS2 | turquoise |
| ENSG00000183023 | SLC8A1 | turquoise |
| ENSG00000183049 | CAMK1D | turquoise |
| ENSG00000183054 | RGPD6 | turquoise |
| ENSG00000183060 | LYSMD4 | turquoise |
| ENSG00000183087 | GAS6 | turquoise |
| ENSG00000183092 | BEGAIN | turquoise |
| ENSG00000183137 | CEP57L1 | turquoise |
| ENSG00000183150 | GPR19 | turquoise |
| ENSG00000183154 | AC138356.1 | turquoise |
| ENSG00000183155 | RABIF | turquoise |
| ENSG00000183160 | TMEM119 | turquoise |
| ENSG00000183161 | FANCF | turquoise |
| ENSG00000183171 | BX322650.1 | turquoise |
| ENSG00000183199 | AC093768.1 | turquoise |
| ENSG00000183208 | GDPGP1 | turquoise |
| ENSG00000183248 | PRR36 | turquoise |
| ENSG00000183281 | PLGLB1 | turquoise |
| ENSG00000183283 | DAZAP2 | turquoise |
| ENSG00000183291 | SELENOF | turquoise |
| ENSG00000183307 | TMEM121B | turquoise |
| ENSG00000183309 | ZNF623 | turquoise |
| ENSG00000183337 | BCOR | turquoise |
| ENSG00000183340 | JRKL | turquoise |
| ENSG00000183386 | FHL3 | turquoise |
| ENSG00000183397 | C19orf71 | turquoise |
| ENSG00000183401 | CCDC159 | turquoise |
| ENSG00000183421 | RIPK4 | turquoise |
| ENSG00000183423 | LRIT3 | turquoise |
| ENSG00000183426 | NPIPA1 | turquoise |
| ENSG00000183439 | TRIM61 | turquoise |
| ENSG00000183474 | GTF2H2C | turquoise |

| | | |
|---|---|---|
| ENSG00000183475 | ASB7 | turquoise |
| ENSG00000183479 | TREX2 | turquoise |
| ENSG00000183484 | GPR132 | turquoise |
| ENSG00000183495 | EP400 | turquoise |
| ENSG00000183513 | COA5 | turquoise |
| ENSG00000183527 | PSMG1 | turquoise |
| ENSG00000183542 | KLRC4 | turquoise |
| ENSG00000183570 | PCBP3 | turquoise |
| ENSG00000183576 | SETD3 | turquoise |
| ENSG00000183579 | ZNRF3 | turquoise |
| ENSG00000183597 | TANGO2 | turquoise |
| ENSG00000183615 | FAM167B | turquoise |
| ENSG00000183625 | CCR3 | turquoise |
| ENSG00000183648 | NDUFB1 | turquoise |
| ENSG00000183662 | FAM19A1 | turquoise |
| ENSG00000183682 | BMP8A | turquoise |
| ENSG00000183691 | NOG | turquoise |
| ENSG00000183696 | UPP1 | turquoise |
| ENSG00000183718 | TRIM52 | turquoise |
| ENSG00000183722 | LHFPL6 | turquoise |
| ENSG00000183735 | TBK1 | turquoise |
| ENSG00000183741 | CBX6 | turquoise |
| ENSG00000183763 | TRAIP | turquoise |
| ENSG00000183765 | CHEK2 | turquoise |
| ENSG00000183773 | AIFM3 | turquoise |
| ENSG00000183801 | OLFML1 | turquoise |
| ENSG00000183808 | RBM12B | turquoise |
| ENSG00000183813 | CCR4 | turquoise |
| ENSG00000183814 | LIN9 | turquoise |
| ENSG00000183822 | NCF4-AS1 | turquoise |
| ENSG00000183826 | BTBD9 | turquoise |
| ENSG00000183864 | TOB2 | turquoise |
| ENSG00000183889 | AC138969.1 | turquoise |
| ENSG00000183891 | TTC32 | turquoise |
| ENSG00000183918 | SH2D1A | turquoise |
| ENSG00000183935 | HTR7P1 | turquoise |
| ENSG00000183943 | PRKX | turquoise |
| ENSG00000183960 | KCNH8 | turquoise |
| ENSG00000183963 | SMTN | turquoise |
| ENSG00000184005 | ST6GALNAC3 | turquoise |
| ENSG00000184007 | PTP4A2 | turquoise |
| ENSG00000184014 | DENND5A | turquoise |
| ENSG00000184047 | DIABLO | turquoise |
| ENSG00000184068 | AL021453.1 | turquoise |
| ENSG00000184100 | BRD7P2 | turquoise |
| ENSG00000184163 | C1QTNF12 | turquoise |
| ENSG00000184203 | PPP1R2 | turquoise |
| ENSG00000184205 | TSPYL2 | turquoise |

| | | |
|---|---|---|
| ENSG00000184207 | PGP | turquoise |
| ENSG00000184226 | PCDH9 | turquoise |
| ENSG00000184232 | OAF | turquoise |
| ENSG00000184277 | TM2D3 | turquoise |
| ENSG00000184292 | TACSTD2 | turquoise |
| ENSG00000184305 | CCSER1 | turquoise |
| ENSG00000184307 | ZDHHC23 | turquoise |
| ENSG00000184343 | SRPK3 | turquoise |
| ENSG00000184381 | PLA2G6 | turquoise |
| ENSG00000184384 | MAML2 | turquoise |
| ENSG00000184441 | AP001062.1 | turquoise |
| ENSG00000184445 | KNTC1 | turquoise |
| ENSG00000184451 | CCR10 | turquoise |
| ENSG00000184470 | TXNRD2 | turquoise |
| ENSG00000184497 | TMEM255B | turquoise |
| ENSG00000184500 | PROS1 | turquoise |
| ENSG00000184517 | ZFP1 | turquoise |
| ENSG00000184524 | CEND1 | turquoise |
| ENSG00000184575 | XPOT | turquoise |
| ENSG00000184588 | PDE4B | turquoise |
| ENSG00000184611 | KCNH7 | turquoise |
| ENSG00000184616 | SPDYE12P | turquoise |
| ENSG00000184634 | MED12 | turquoise |
| ENSG00000184635 | ZNF93 | turquoise |
| ENSG00000184677 | ZBTB40 | turquoise |
| ENSG00000184678 | HIST2H2BE | turquoise |
| ENSG00000184702 | 5-Sep | turquoise |
| ENSG00000184708 | EIF4ENIF1 | turquoise |
| ENSG00000184743 | ATL3 | turquoise |
| ENSG00000184752 | NDUFA12 | turquoise |
| ENSG00000184857 | TMEM186 | turquoise |
| ENSG00000184867 | ARMCX2 | turquoise |
| ENSG00000184887 | BTBD6 | turquoise |
| ENSG00000184898 | RBM43 | turquoise |
| ENSG00000184925 | LCN12 | turquoise |
| ENSG00000184939 | ZFP90 | turquoise |
| ENSG00000184990 | SIVA1 | turquoise |
| ENSG00000184992 | BRI3BP | turquoise |
| ENSG00000185000 | DGAT1 | turquoise |
| ENSG00000185009 | AP3M1 | turquoise |
| ENSG00000185010 | F8 | turquoise |
| ENSG00000185019 | UBOX5 | turquoise |
| ENSG00000185088 | RPS27L | turquoise |
| ENSG00000185090 | MANEAL | turquoise |
| ENSG00000185100 | ADSSL1 | turquoise |
| ENSG00000185101 | ANO9 | turquoise |
| ENSG00000185115 | NSMCE3 | turquoise |
| ENSG00000185122 | HSF1 | turquoise |

| | | |
|---|---|---|
| ENSG00000185127 | C6orf120 | turquoise |
| ENSG00000185129 | PURA | turquoise |
| ENSG00000185133 | INPP5J | turquoise |
| ENSG00000185163 | DDX51 | turquoise |
| ENSG00000185164 | NOMO2 | turquoise |
| ENSG00000185168 | LINC00482 | turquoise |
| ENSG00000185220 | PGBD2 | turquoise |
| ENSG00000185236 | RAB11B | turquoise |
| ENSG00000185238 | PRMT3 | turquoise |
| ENSG00000185246 | PRPF39 | turquoise |
| ENSG00000185252 | ZNF74 | turquoise |
| ENSG00000185267 | CDNF | turquoise |
| ENSG00000185272 | RBM11 | turquoise |
| ENSG00000185291 | IL3RA | turquoise |
| ENSG00000185305 | ARL15 | turquoise |
| ENSG00000185324 | CDK10 | turquoise |
| ENSG00000185344 | ATP6V0A2 | turquoise |
| ENSG00000185347 | C14orf80 | turquoise |
| ENSG00000185379 | RAD51D | turquoise |
| ENSG00000185386 | MAPK11 | turquoise |
| ENSG00000185414 | MRPL30 | turquoise |
| ENSG00000185418 | TARSL2 | turquoise |
| ENSG00000185432 | METTL7A | turquoise |
| ENSG00000185436 | IFNLR1 | turquoise |
| ENSG00000185437 | SH3BGR | turquoise |
| ENSG00000185477 | GPRIN3 | turquoise |
| ENSG00000185485 | SDHAP1 | turquoise |
| ENSG00000185495 | AC138393.1 | turquoise |
| ENSG00000185499 | MUC1 | turquoise |
| ENSG00000185504 | FAAP100 | turquoise |
| ENSG00000185515 | BRCC3 | turquoise |
| ENSG00000185596 | WASH3P | turquoise |
| ENSG00000185608 | MRPL40 | turquoise |
| ENSG00000185614 | FAM212A | turquoise |
| ENSG00000185621 | LMLN | turquoise |
| ENSG00000185650 | ZFP36L1 | turquoise |
| ENSG00000185658 | BRWD1 | turquoise |
| ENSG00000185662 | SMIM23 | turquoise |
| ENSG00000185669 | SNAI3 | turquoise |
| ENSG00000185670 | ZBTB3 | turquoise |
| ENSG00000185674 | LYG2 | turquoise |
| ENSG00000185697 | MYBL1 | turquoise |
| ENSG00000185728 | YTHDF3 | turquoise |
| ENSG00000185753 | CXorf38 | turquoise |
| ENSG00000185760 | KCNQ5 | turquoise |
| ENSG00000185774 | KCNIP4 | turquoise |
| ENSG00000185787 | MORF4L1 | turquoise |
| ENSG00000185798 | WDR53 | turquoise |

| | | |
|---|---|---|
| ENSG00000185800 | DMWD | turquoise |
| ENSG00000185811 | IKZF1 | turquoise |
| ENSG00000185813 | PCYT2 | turquoise |
| ENSG00000185818 | NAT8L | turquoise |
| ENSG00000185825 | BCAP31 | turquoise |
| ENSG00000185862 | EVI2B | turquoise |
| ENSG00000185869 | ZNF829 | turquoise |
| ENSG00000185875 | THNSL1 | turquoise |
| ENSG00000185883 | ATP6V0C | turquoise |
| ENSG00000185909 | KLHDC8B | turquoise |
| ENSG00000185915 | KLHL34 | turquoise |
| ENSG00000185917 | SETD4 | turquoise |
| ENSG00000185920 | PTCH1 | turquoise |
| ENSG00000185924 | RTN4RL1 | turquoise |
| ENSG00000185946 | RNPC3 | turquoise |
| ENSG00000185947 | ZNF267 | turquoise |
| ENSG00000185950 | IRS2 | turquoise |
| ENSG00000185955 | C7orf61 | turquoise |
| ENSG00000185958 | FAM186A | turquoise |
| ENSG00000185973 | TMLHE | turquoise |
| ENSG00000185989 | RASA3 | turquoise |
| ENSG00000186010 | NDUFA13 | turquoise |
| ENSG00000186017 | ZNF566 | turquoise |
| ENSG00000186056 | MATN1-AS1 | turquoise |
| ENSG00000186063 | AIDA | turquoise |
| ENSG00000186073 | C15orf41 | turquoise |
| ENSG00000186088 | GSAP | turquoise |
| ENSG00000186104 | CYP2R1 | turquoise |
| ENSG00000186105 | LRRC70 | turquoise |
| ENSG00000186106 | ANKRD46 | turquoise |
| ENSG00000186111 | PIP5K1C | turquoise |
| ENSG00000186130 | ZBTB6 | turquoise |
| ENSG00000186132 | C2orf76 | turquoise |
| ENSG00000186162 | CIDECP | turquoise |
| ENSG00000186185 | KIF18B | turquoise |
| ENSG00000186187 | ZNRF1 | turquoise |
| ENSG00000186193 | SAPCD2 | turquoise |
| ENSG00000186204 | CYP4F12 | turquoise |
| ENSG00000186230 | ZNF749 | turquoise |
| ENSG00000186231 | KLHL32 | turquoise |
| ENSG00000186260 | MKL2 | turquoise |
| ENSG00000186265 | BTLA | turquoise |
| ENSG00000186272 | ZNF17 | turquoise |
| ENSG00000186280 | KDM4D | turquoise |
| ENSG00000186298 | PPP1CC | turquoise |
| ENSG00000186300 | ZNF555 | turquoise |
| ENSG00000186312 | CA5BP1 | turquoise |
| ENSG00000186318 | BACE1 | turquoise |

| | | |
|---|---|---|
| ENSG00000186340 | THBS2 | turquoise |
| ENSG00000186376 | ZNF75D | turquoise |
| ENSG00000186416 | NKRF | turquoise |
| ENSG00000186432 | KPNA4 | turquoise |
| ENSG00000186448 | ZNF197 | turquoise |
| ENSG00000186462 | NAP1L2 | turquoise |
| ENSG00000186468 | RPS23 | turquoise |
| ENSG00000186469 | GNG2 | turquoise |
| ENSG00000186480 | INSIG1 | turquoise |
| ENSG00000186496 | ZNF396 | turquoise |
| ENSG00000186501 | TMEM222 | turquoise |
| ENSG00000186522 | 10-Sep | turquoise |
| ENSG00000186523 | FAM86B1 | turquoise |
| ENSG00000186564 | FOXD2 | turquoise |
| ENSG00000186566 | GPATCH8 | turquoise |
| ENSG00000186567 | CEACAM19 | turquoise |
| ENSG00000186583 | SPATC1 | turquoise |
| ENSG00000186603 | HPDL | turquoise |
| ENSG00000186625 | KATNA1 | turquoise |
| ENSG00000186642 | PDE2A | turquoise |
| ENSG00000186648 | CARMIL3 | turquoise |
| ENSG00000186654 | PRR5 | turquoise |
| ENSG00000186660 | ZFP91 | turquoise |
| ENSG00000186665 | C17orf58 | turquoise |
| ENSG00000186666 | BCDIN3D | turquoise |
| ENSG00000186687 | LYRM7 | turquoise |
| ENSG00000186716 | BCR | turquoise |
| ENSG00000186765 | FSCN2 | turquoise |
| ENSG00000186767 | SPIN4 | turquoise |
| ENSG00000186777 | ZNF732 | turquoise |
| ENSG00000186815 | TPCN1 | turquoise |
| ENSG00000186834 | HEXIM1 | turquoise |
| ENSG00000186862 | PDZD7 | turquoise |
| ENSG00000186907 | RTN4RL2 | turquoise |
| ENSG00000186908 | ZDHHC17 | turquoise |
| ENSG00000186940 | CHCHD2P9 | turquoise |
| ENSG00000186951 | PPARA | turquoise |
| ENSG00000186994 | KANK3 | turquoise |
| ENSG00000187045 | TMPRSS6 | turquoise |
| ENSG00000187049 | TMEM216 | turquoise |
| ENSG00000187066 | TMEM262 | turquoise |
| ENSG00000187091 | PLCD1 | turquoise |
| ENSG00000187097 | ENTPD5 | turquoise |
| ENSG00000187098 | MITF | turquoise |
| ENSG00000187105 | HEATR4 | turquoise |
| ENSG00000187109 | NAP1L1 | turquoise |
| ENSG00000187116 | LILRA5 | turquoise |
| ENSG00000187118 | CMC1 | turquoise |

| | | |
|---|---|---|
| ENSG00000187164 | SHTN1 | turquoise |
| ENSG00000187166 | H1FNT | turquoise |
| ENSG00000187189 | TSPYL4 | turquoise |
| ENSG00000187210 | GCNT1 | turquoise |
| ENSG00000187231 | SESTD1 | turquoise |
| ENSG00000187239 | FNBP1 | turquoise |
| ENSG00000187257 | RSBN1L | turquoise |
| ENSG00000187260 | WDR86 | turquoise |
| ENSG00000187266 | EPOR | turquoise |
| ENSG00000187325 | TAF9B | turquoise |
| ENSG00000187446 | CHP1 | turquoise |
| ENSG00000187474 | FPR3 | turquoise |
| ENSG00000187510 | PLEKHG7 | turquoise |
| ENSG00000187522 | HSPA14 | turquoise |
| ENSG00000187534 | PRR13P5 | turquoise |
| ENSG00000187546 | AGMO | turquoise |
| ENSG00000187555 | USP7 | turquoise |
| ENSG00000187583 | PLEKHN1 | turquoise |
| ENSG00000187595 | ZNF385C | turquoise |
| ENSG00000187601 | MAGEH1 | turquoise |
| ENSG00000187605 | TET3 | turquoise |
| ENSG00000187607 | ZNF286A | turquoise |
| ENSG00000187609 | EXD3 | turquoise |
| ENSG00000187626 | ZKSCAN4 | turquoise |
| ENSG00000187642 | PERM1 | turquoise |
| ENSG00000187676 | B3GLCT | turquoise |
| ENSG00000187695 | AC112484.1 | turquoise |
| ENSG00000187699 | C2orf88 | turquoise |
| ENSG00000187735 | TCEA1 | turquoise |
| ENSG00000187742 | SECISBP2 | turquoise |
| ENSG00000187790 | FANCM | turquoise |
| ENSG00000187796 | CARD9 | turquoise |
| ENSG00000187808 | SOWAHD | turquoise |
| ENSG00000187837 | HIST1H1C | turquoise |
| ENSG00000187860 | CCDC157 | turquoise |
| ENSG00000187866 | FAM122A | turquoise |
| ENSG00000187912 | CLEC17A | turquoise |
| ENSG00000187922 | LCN10 | turquoise |
| ENSG00000187942 | LDLRAD2 | turquoise |
| ENSG00000187954 | CYHR1 | turquoise |
| ENSG00000187961 | KLHL17 | turquoise |
| ENSG00000188001 | TPRG1 | turquoise |
| ENSG00000188002 | AC026412.1 | turquoise |
| ENSG00000188004 | SNHG28 | turquoise |
| ENSG00000188010 | MORN2 | turquoise |
| ENSG00000188021 | UBQLN2 | turquoise |
| ENSG00000188033 | ZNF490 | turquoise |
| ENSG00000188056 | TREML4 | turquoise |

| | | |
|---|---|---|
| ENSG00000188092 | GPR89B | turquoise |
| ENSG00000188130 | MAPK12 | turquoise |
| ENSG00000188158 | NHS | turquoise |
| ENSG00000188171 | ZNF626 | turquoise |
| ENSG00000188177 | ZC3H6 | turquoise |
| ENSG00000188227 | ZNF793 | turquoise |
| ENSG00000188229 | TUBB4B | turquoise |
| ENSG00000188243 | COMMD6 | turquoise |
| ENSG00000188283 | ZNF383 | turquoise |
| ENSG00000188295 | ZNF669 | turquoise |
| ENSG00000188321 | ZNF559 | turquoise |
| ENSG00000188322 | SBK1 | turquoise |
| ENSG00000188342 | GTF2F2 | turquoise |
| ENSG00000188343 | FAM92A | turquoise |
| ENSG00000188368 | PRR19 | turquoise |
| ENSG00000188372 | ZP3 | turquoise |
| ENSG00000188389 | PDCD1 | turquoise |
| ENSG00000188396 | TCTEX1D4 | turquoise |
| ENSG00000188419 | CHM | turquoise |
| ENSG00000188428 | BLOC1S5 | turquoise |
| ENSG00000188452 | CERKL | turquoise |
| ENSG00000188483 | IER5L | turquoise |
| ENSG00000188493 | C19orf54 | turquoise |
| ENSG00000188522 | FAM83G | turquoise |
| ENSG00000188529 | SRSF10 | turquoise |
| ENSG00000188542 | DUSP28 | turquoise |
| ENSG00000188549 | C15orf52 | turquoise |
| ENSG00000188554 | NBR1 | turquoise |
| ENSG00000188566 | NDOR1 | turquoise |
| ENSG00000188610 | FAM72B | turquoise |
| ENSG00000188611 | ASAH2 | turquoise |
| ENSG00000188636 | RTL6 | turquoise |
| ENSG00000188641 | DPYD | turquoise |
| ENSG00000188647 | PTAR1 | turquoise |
| ENSG00000188649 | CC2D2B | turquoise |
| ENSG00000188676 | IDO2 | turquoise |
| ENSG00000188677 | PARVB | turquoise |
| ENSG00000188681 | TEKT4P2 | turquoise |
| ENSG00000188706 | ZDHHC9 | turquoise |
| ENSG00000188707 | ZBED6CL | turquoise |
| ENSG00000188710 | QRFP | turquoise |
| ENSG00000188725 | SMIM15 | turquoise |
| ENSG00000188732 | FAM221A | turquoise |
| ENSG00000188735 | TMEM120B | turquoise |
| ENSG00000188747 | NOXA1 | turquoise |
| ENSG00000188766 | SPRED3 | turquoise |
| ENSG00000188785 | ZNF548 | turquoise |
| ENSG00000188786 | MTF1 | turquoise |

| | | |
|---|---|---|
| ENSG00000188811 | NHLRC3 | turquoise |
| ENSG00000188822 | CNR2 | turquoise |
| ENSG00000188825 | LINC00910 | turquoise |
| ENSG00000188827 | SLX4 | turquoise |
| ENSG00000188848 | BEND4 | turquoise |
| ENSG00000188859 | FAM78B | turquoise |
| ENSG00000188868 | ZNF563 | turquoise |
| ENSG00000188878 | FBF1 | turquoise |
| ENSG00000188886 | ASTL | turquoise |
| ENSG00000188897 | AC099489.1 | turquoise |
| ENSG00000188906 | LRRK2 | turquoise |
| ENSG00000188917 | TRMT2B | turquoise |
| ENSG00000188921 | HACD4 | turquoise |
| ENSG00000188958 | UTS2B | turquoise |
| ENSG00000188994 | ZNF292 | turquoise |
| ENSG00000189007 | ADAT2 | turquoise |
| ENSG00000189014 | FAM35DP | turquoise |
| ENSG00000189042 | ZNF567 | turquoise |
| ENSG00000189043 | NDUFA4 | turquoise |
| ENSG00000189045 | ANKDD1B | turquoise |
| ENSG00000189046 | ALKBH2 | turquoise |
| ENSG00000189050 | RNFT1 | turquoise |
| ENSG00000189057 | FAM111B | turquoise |
| ENSG00000189068 | VSTM1 | turquoise |
| ENSG00000189077 | TMEM120A | turquoise |
| ENSG00000189079 | ARID2 | turquoise |
| ENSG00000189114 | BLOC1S3 | turquoise |
| ENSG00000189152 | GRAPL | turquoise |
| ENSG00000189164 | ZNF527 | turquoise |
| ENSG00000189171 | S100A13 | turquoise |
| ENSG00000189190 | ZNF600 | turquoise |
| ENSG00000189241 | TSPYL1 | turquoise |
| ENSG00000189266 | PNRC2 | turquoise |
| ENSG00000189269 | DRICH1 | turquoise |
| ENSG00000189292 | ALKAL2 | turquoise |
| ENSG00000189298 | ZKSCAN3 | turquoise |
| ENSG00000189319 | FAM53B | turquoise |
| ENSG00000189339 | SLC35E2B | turquoise |
| ENSG00000189369 | GSPT2 | turquoise |
| ENSG00000189376 | C8orf76 | turquoise |
| ENSG00000189403 | HMGB1 | turquoise |
| ENSG00000189409 | MMP23B | turquoise |
| ENSG00000196072 | BLOC1S2 | turquoise |
| ENSG00000196074 | SYCP2 | turquoise |
| ENSG00000196083 | IL1RAP | turquoise |
| ENSG00000196118 | CCDC189 | turquoise |
| ENSG00000196126 | HLA-DRB1 | turquoise |
| ENSG00000196172 | ZNF681 | turquoise |

| | | |
|---|---|---|
| ENSG00000196177 | ACADSB | turquoise |
| ENSG00000196204 | RNF216P1 | turquoise |
| ENSG00000196205 | EEF1A1P5 | turquoise |
| ENSG00000196214 | ZNF766 | turquoise |
| ENSG00000196227 | FAM217B | turquoise |
| ENSG00000196235 | SUPT5H | turquoise |
| ENSG00000196247 | ZNF107 | turquoise |
| ENSG00000196263 | ZNF471 | turquoise |
| ENSG00000196267 | ZNF836 | turquoise |
| ENSG00000196290 | NIF3L1 | turquoise |
| ENSG00000196296 | ATP2A1 | turquoise |
| ENSG00000196305 | IARS | turquoise |
| ENSG00000196323 | ZBTB44 | turquoise |
| ENSG00000196338 | NLGN3 | turquoise |
| ENSG00000196357 | ZNF565 | turquoise |
| ENSG00000196366 | C9orf163 | turquoise |
| ENSG00000196367 | TRRAP | turquoise |
| ENSG00000196368 | NUDT11 | turquoise |
| ENSG00000196369 | SRGAP2B | turquoise |
| ENSG00000196371 | FUT4 | turquoise |
| ENSG00000196378 | ZNF34 | turquoise |
| ENSG00000196381 | ZNF781 | turquoise |
| ENSG00000196387 | ZNF140 | turquoise |
| ENSG00000196405 | EVL | turquoise |
| ENSG00000196411 | EPHB4 | turquoise |
| ENSG00000196418 | ZNF124 | turquoise |
| ENSG00000196420 | S100A5 | turquoise |
| ENSG00000196421 | C20orf204 | turquoise |
| ENSG00000196428 | TSC22D2 | turquoise |
| ENSG00000196437 | ZNF569 | turquoise |
| ENSG00000196440 | ARMCX4 | turquoise |
| ENSG00000196449 | YRDC | turquoise |
| ENSG00000196455 | PIK3R4 | turquoise |
| ENSG00000196458 | ZNF605 | turquoise |
| ENSG00000196459 | TRAPPC2 | turquoise |
| ENSG00000196465 | MYL6B | turquoise |
| ENSG00000196466 | ZNF799 | turquoise |
| ENSG00000196470 | SIAH1 | turquoise |
| ENSG00000196476 | C20orf96 | turquoise |
| ENSG00000196497 | IPO4 | turquoise |
| ENSG00000196498 | NCOR2 | turquoise |
| ENSG00000196502 | SULT1A1 | turquoise |
| ENSG00000196504 | PRPF40A | turquoise |
| ENSG00000196511 | TPK1 | turquoise |
| ENSG00000196535 | MYO18A | turquoise |
| ENSG00000196542 | SPTSSB | turquoise |
| ENSG00000196549 | MME | turquoise |
| ENSG00000196569 | LAMA2 | turquoise |

| | | |
|---|---|---|
| ENSG00000196581 | AJAP1 | turquoise |
| ENSG00000196586 | MYO6 | turquoise |
| ENSG00000196591 | HDAC2 | turquoise |
| ENSG00000196597 | ZNF782 | turquoise |
| ENSG00000196628 | TCF4 | turquoise |
| ENSG00000196632 | WNK3 | turquoise |
| ENSG00000196636 | SDHAF3 | turquoise |
| ENSG00000196646 | ZNF136 | turquoise |
| ENSG00000196656 | AC004057.1 | turquoise |
| ENSG00000196670 | ZFP62 | turquoise |
| ENSG00000196678 | ERI2 | turquoise |
| ENSG00000196683 | TOMM7 | turquoise |
| ENSG00000196689 | TRPV1 | turquoise |
| ENSG00000196693 | ZNF33B | turquoise |
| ENSG00000196696 | PDXDC2P-NPIPB1 | turquoise |
| ENSG00000196704 | AMZ2 | turquoise |
| ENSG00000196712 | NF1 | turquoise |
| ENSG00000196715 | VKORC1L1 | turquoise |
| ENSG00000196730 | DAPK1 | turquoise |
| ENSG00000196735 | HLA-DQA1 | turquoise |
| ENSG00000196739 | COL27A1 | turquoise |
| ENSG00000196747 | HIST1H2AI | turquoise |
| ENSG00000196756 | SNHG17 | turquoise |
| ENSG00000196776 | CD47 | turquoise |
| ENSG00000196781 | TLE1 | turquoise |
| ENSG00000196782 | MAML3 | turquoise |
| ENSG00000196787 | HIST1H2AG | turquoise |
| ENSG00000196792 | STRN3 | turquoise |
| ENSG00000196793 | ZNF239 | turquoise |
| ENSG00000196796 | NPIPB10P | turquoise |
| ENSG00000196810 | CTBP1-AS2 | turquoise |
| ENSG00000196839 | ADA | turquoise |
| ENSG00000196843 | ARID5A | turquoise |
| ENSG00000196850 | PPTC7 | turquoise |
| ENSG00000196865 | NHLRC2 | turquoise |
| ENSG00000196873 | CBWD3 | turquoise |
| ENSG00000196878 | LAMB3 | turquoise |
| ENSG00000196890 | HIST3H2BB | turquoise |
| ENSG00000196911 | KPNA5 | turquoise |
| ENSG00000196922 | ZNF252P | turquoise |
| ENSG00000196923 | PDLIM7 | turquoise |
| ENSG00000196924 | FLNA | turquoise |
| ENSG00000196937 | FAM3C | turquoise |
| ENSG00000196943 | NOP9 | turquoise |
| ENSG00000196950 | SLC39A10 | turquoise |
| ENSG00000196961 | AP2A1 | turquoise |
| ENSG00000196968 | FUT11 | turquoise |
| ENSG00000196981 | WDR5B | turquoise |

| | | |
|---|---|---|
| ENSG00000196998 | WDR45 | turquoise |
| ENSG00000197024 | ZNF398 | turquoise |
| ENSG00000197037 | ZSCAN25 | turquoise |
| ENSG00000197044 | ZNF441 | turquoise |
| ENSG00000197045 | GMFB | turquoise |
| ENSG00000197050 | ZNF420 | turquoise |
| ENSG00000197054 | ZNF763 | turquoise |
| ENSG00000197056 | ZMYM1 | turquoise |
| ENSG00000197062 | ZSCAN26 | turquoise |
| ENSG00000197063 | MAFG | turquoise |
| ENSG00000197070 | ARRDC1 | turquoise |
| ENSG00000197077 | KIAA1671 | turquoise |
| ENSG00000197114 | ZGPAT | turquoise |
| ENSG00000197119 | SLC25A29 | turquoise |
| ENSG00000197124 | ZNF682 | turquoise |
| ENSG00000197128 | ZNF772 | turquoise |
| ENSG00000197134 | ZNF257 | turquoise |
| ENSG00000197136 | PCNX3 | turquoise |
| ENSG00000197142 | ACSL5 | turquoise |
| ENSG00000197147 | LRRC8B | turquoise |
| ENSG00000197150 | ABCB8 | turquoise |
| ENSG00000197162 | ZNF785 | turquoise |
| ENSG00000197168 | NEK5 | turquoise |
| ENSG00000197170 | PSMD12 | turquoise |
| ENSG00000197180 | CH17-340M24.3 | turquoise |
| ENSG00000197182 | MIRLET7BHG | turquoise |
| ENSG00000197191 | CYSRT1 | turquoise |
| ENSG00000197208 | SLC22A4 | turquoise |
| ENSG00000197223 | C1D | turquoise |
| ENSG00000197226 | TBC1D9B | turquoise |
| ENSG00000197253 | TPSB2 | turquoise |
| ENSG00000197265 | GTF2E2 | turquoise |
| ENSG00000197275 | RAD54B | turquoise |
| ENSG00000197279 | ZNF165 | turquoise |
| ENSG00000197299 | BLM | turquoise |
| ENSG00000197302 | ZNF720 | turquoise |
| ENSG00000197312 | DDI2 | turquoise |
| ENSG00000197321 | SVIL | turquoise |
| ENSG00000197323 | TRIM33 | turquoise |
| ENSG00000197329 | PELI1 | turquoise |
| ENSG00000197363 | ZNF517 | turquoise |
| ENSG00000197372 | ZNF675 | turquoise |
| ENSG00000197381 | ADARB1 | turquoise |
| ENSG00000197417 | SHPK | turquoise |
| ENSG00000197442 | MAP3K5 | turquoise |
| ENSG00000197448 | GSTK1 | turquoise |
| ENSG00000197457 | STMN3 | turquoise |
| ENSG00000197471 | SPN | turquoise |

| | | |
|---|---|---|
| ENSG00000197483 | ZNF628 | turquoise |
| ENSG00000197498 | RPF2 | turquoise |
| ENSG00000197506 | SLC28A3 | turquoise |
| ENSG00000197520 | FAM177B | turquoise |
| ENSG00000197530 | MIB2 | turquoise |
| ENSG00000197549 | PRAMENP | turquoise |
| ENSG00000197557 | TTC30A | turquoise |
| ENSG00000197558 | SSPO | turquoise |
| ENSG00000197562 | RAB40C | turquoise |
| ENSG00000197563 | PIGN | turquoise |
| ENSG00000197566 | ZNF624 | turquoise |
| ENSG00000197579 | TOPORS | turquoise |
| ENSG00000197580 | BCO2 | turquoise |
| ENSG00000197586 | ENTPD6 | turquoise |
| ENSG00000197594 | ENPP1 | turquoise |
| ENSG00000197599 | CCDC154 | turquoise |
| ENSG00000197601 | FAR1 | turquoise |
| ENSG00000197603 | C5orf42 | turquoise |
| ENSG00000197619 | ZNF615 | turquoise |
| ENSG00000197620 | CXorf40A | turquoise |
| ENSG00000197632 | SERPINB2 | turquoise |
| ENSG00000197696 | NMB | turquoise |
| ENSG00000197705 | KLHL14 | turquoise |
| ENSG00000197713 | RPE | turquoise |
| ENSG00000197724 | PHF2 | turquoise |
| ENSG00000197728 | RPS26 | turquoise |
| ENSG00000197744 | PTMAP2 | turquoise |
| ENSG00000197763 | TXNRD3 | turquoise |
| ENSG00000197766 | CFD | turquoise |
| ENSG00000197768 | STPG3 | turquoise |
| ENSG00000197771 | MCMBP | turquoise |
| ENSG00000197774 | EME2 | turquoise |
| ENSG00000197776 | KLHDC1 | turquoise |
| ENSG00000197780 | TAF13 | turquoise |
| ENSG00000197808 | ZNF461 | turquoise |
| ENSG00000197841 | ZNF181 | turquoise |
| ENSG00000197847 | SLC22A20 | turquoise |
| ENSG00000197858 | GPAA1 | turquoise |
| ENSG00000197860 | SGTB | turquoise |
| ENSG00000197872 | FAM49A | turquoise |
| ENSG00000197879 | MYO1C | turquoise |
| ENSG00000197885 | NKIRAS1 | turquoise |
| ENSG00000197892 | KIF13B | turquoise |
| ENSG00000197894 | ADH5 | turquoise |
| ENSG00000197903 | HIST1H2BK | turquoise |
| ENSG00000197912 | SPG7 | turquoise |
| ENSG00000197928 | ZNF677 | turquoise |
| ENSG00000197930 | ERO1A | turquoise |

| | | |
|---|---|---|
| ENSG00000197933 | ZNF823 | turquoise |
| ENSG00000197937 | ZNF347 | turquoise |
| ENSG00000197948 | FCHSD1 | turquoise |
| ENSG00000197951 | ZNF71 | turquoise |
| ENSG00000197959 | DNM3 | turquoise |
| ENSG00000197965 | MPZL1 | turquoise |
| ENSG00000197969 | VPS13A | turquoise |
| ENSG00000197971 | MBP | turquoise |
| ENSG00000197980 | LEKR1 | turquoise |
| ENSG00000197992 | CLEC9A | turquoise |
| ENSG00000198000 | NOL8 | turquoise |
| ENSG00000198001 | IRAK4 | turquoise |
| ENSG00000198015 | MRPL42 | turquoise |
| ENSG00000198026 | ZNF335 | turquoise |
| ENSG00000198039 | ZNF273 | turquoise |
| ENSG00000198040 | ZNF84 | turquoise |
| ENSG00000198042 | MAK16 | turquoise |
| ENSG00000198055 | GRK6 | turquoise |
| ENSG00000198056 | PRIM1 | turquoise |
| ENSG00000198060 | 5-Mar | turquoise |
| ENSG00000198064 | NPIPB13 | turquoise |
| ENSG00000198081 | ZBTB14 | turquoise |
| ENSG00000198087 | CD2AP | turquoise |
| ENSG00000198088 | NUP62CL | turquoise |
| ENSG00000198089 | SFI1 | turquoise |
| ENSG00000198093 | ZNF649 | turquoise |
| ENSG00000198106 | AC025279.1 | turquoise |
| ENSG00000198121 | LPAR1 | turquoise |
| ENSG00000198130 | HIBCH | turquoise |
| ENSG00000198142 | SOWAHC | turquoise |
| ENSG00000198146 | ZNF770 | turquoise |
| ENSG00000198156 | NPIPB6 | turquoise |
| ENSG00000198157 | HMGN5 | turquoise |
| ENSG00000198160 | MIER1 | turquoise |
| ENSG00000198162 | MAN1A2 | turquoise |
| ENSG00000198168 | SVIP | turquoise |
| ENSG00000198176 | TFDP1 | turquoise |
| ENSG00000198182 | ZNF607 | turquoise |
| ENSG00000198185 | ZNF334 | turquoise |
| ENSG00000198189 | HSD17B11 | turquoise |
| ENSG00000198198 | SZT2 | turquoise |
| ENSG00000198205 | ZXDA | turquoise |
| ENSG00000198208 | RPS6KL1 | turquoise |
| ENSG00000198225 | FKBP1C | turquoise |
| ENSG00000198231 | DDX42 | turquoise |
| ENSG00000198246 | SLC29A3 | turquoise |
| ENSG00000198252 | STYX | turquoise |
| ENSG00000198265 | HELZ | turquoise |

| | | |
|---|---|---|
| ENSG00000198276 | UCKL1 | turquoise |
| ENSG00000198301 | SDAD1 | turquoise |
| ENSG00000198315 | ZKSCAN8 | turquoise |
| ENSG00000198324 | FAM109A | turquoise |
| ENSG00000198346 | ZNF813 | turquoise |
| ENSG00000198363 | ASPH | turquoise |
| ENSG00000198369 | SPRED2 | turquoise |
| ENSG00000198380 | GFPT1 | turquoise |
| ENSG00000198382 | UVRAG | turquoise |
| ENSG00000198393 | ZNF26 | turquoise |
| ENSG00000198406 | BZW1P2 | turquoise |
| ENSG00000198408 | MGEA5 | turquoise |
| ENSG00000198420 | TCAF1 | turquoise |
| ENSG00000198431 | TXNRD1 | turquoise |
| ENSG00000198440 | ZNF583 | turquoise |
| ENSG00000198453 | ZNF568 | turquoise |
| ENSG00000198455 | ZXDB | turquoise |
| ENSG00000198464 | ZNF480 | turquoise |
| ENSG00000198467 | TPM2 | turquoise |
| ENSG00000198478 | SH3BGRL2 | turquoise |
| ENSG00000198492 | YTHDF2 | turquoise |
| ENSG00000198498 | TMA16 | turquoise |
| ENSG00000198513 | ATL1 | turquoise |
| ENSG00000198522 | GPN1 | turquoise |
| ENSG00000198538 | ZNF28 | turquoise |
| ENSG00000198547 | C20orf203 | turquoise |
| ENSG00000198551 | ZNF627 | turquoise |
| ENSG00000198554 | WDHD1 | turquoise |
| ENSG00000198569 | SLC34A3 | turquoise |
| ENSG00000198580 | AC073343.1 | turquoise |
| ENSG00000198586 | TLK1 | turquoise |
| ENSG00000198589 | LRBA | turquoise |
| ENSG00000198590 | C3orf35 | turquoise |
| ENSG00000198598 | MMP17 | turquoise |
| ENSG00000198604 | BAZ1A | turquoise |
| ENSG00000198610 | AKR1C4 | turquoise |
| ENSG00000198612 | COPS8 | turquoise |
| ENSG00000198624 | CCDC69 | turquoise |
| ENSG00000198642 | KLHL9 | turquoise |
| ENSG00000198643 | FAM3D | turquoise |
| ENSG00000198646 | NCOA6 | turquoise |
| ENSG00000198648 | STK39 | turquoise |
| ENSG00000198658 | ABHD17AP1 | turquoise |
| ENSG00000198673 | FAM19A2 | turquoise |
| ENSG00000198677 | TTC37 | turquoise |
| ENSG00000198682 | PAPSS2 | turquoise |
| ENSG00000198689 | SLC9A6 | turquoise |
| ENSG00000198700 | IPO9 | turquoise |

| | | |
|---|---|---|
| ENSG00000198707 | CEP290 | turquoise |
| ENSG00000198715 | GLMP | turquoise |
| ENSG00000198718 | TOGARAM1 | turquoise |
| ENSG00000198720 | ANKRD13B | turquoise |
| ENSG00000198721 | ECI2 | turquoise |
| ENSG00000198727 | MT-CYB | turquoise |
| ENSG00000198730 | CTR9 | turquoise |
| ENSG00000198732 | SMOC1 | turquoise |
| ENSG00000198742 | SMURF1 | turquoise |
| ENSG00000198743 | SLC5A3 | turquoise |
| ENSG00000198752 | CDC42BPB | turquoise |
| ENSG00000198771 | RCSD1 | turquoise |
| ENSG00000198780 | FAM169A | turquoise |
| ENSG00000198783 | ZNF830 | turquoise |
| ENSG00000198791 | CNOT7 | turquoise |
| ENSG00000198792 | TMEM184B | turquoise |
| ENSG00000198793 | MTOR | turquoise |
| ENSG00000198794 | SCAMP5 | turquoise |
| ENSG00000198795 | ZNF521 | turquoise |
| ENSG00000198796 | ALPK2 | turquoise |
| ENSG00000198799 | LRIG2 | turquoise |
| ENSG00000198814 | GK | turquoise |
| ENSG00000198815 | FOXJ3 | turquoise |
| ENSG00000198818 | SFT2D1 | turquoise |
| ENSG00000198824 | CHAMP1 | turquoise |
| ENSG00000198829 | SUCNR1 | turquoise |
| ENSG00000198833 | UBE2J1 | turquoise |
| ENSG00000198836 | OPA1 | turquoise |
| ENSG00000198839 | ZNF277 | turquoise |
| ENSG00000198843 | SELENOT | turquoise |
| ENSG00000198846 | TOX | turquoise |
| ENSG00000198853 | RUSC2 | turquoise |
| ENSG00000198855 | FICD | turquoise |
| ENSG00000198856 | OSTC | turquoise |
| ENSG00000198858 | R3HDM4 | turquoise |
| ENSG00000198860 | TSEN15 | turquoise |
| ENSG00000198862 | LTN1 | turquoise |
| ENSG00000198863 | RUNDC1 | turquoise |
| ENSG00000198865 | CCDC152 | turquoise |
| ENSG00000198873 | GRK5 | turquoise |
| ENSG00000198874 | TYW1 | turquoise |
| ENSG00000198879 | SFMBT2 | turquoise |
| ENSG00000198883 | PNMA5 | turquoise |
| ENSG00000198887 | SMC5 | turquoise |
| ENSG00000198890 | PRMT6 | turquoise |
| ENSG00000198892 | SHISA4 | turquoise |
| ENSG00000198894 | CIPC | turquoise |
| ENSG00000198898 | CAPZA2 | turquoise |

| | | |
|---|---|---|
| ENSG00000198900 | TOP1 | turquoise |
| ENSG00000198910 | L1CAM | turquoise |
| ENSG00000198912 | C1orf174 | turquoise |
| ENSG00000198918 | RPL39 | turquoise |
| ENSG00000198919 | DZIP3 | turquoise |
| ENSG00000198924 | DCLRE1A | turquoise |
| ENSG00000198925 | ATG9A | turquoise |
| ENSG00000198932 | GPRASP1 | turquoise |
| ENSG00000198933 | TBKBP1 | turquoise |
| ENSG00000198934 | MAGEE1 | turquoise |
| ENSG00000198939 | ZFP2 | turquoise |
| ENSG00000198945 | L3MBTL3 | turquoise |
| ENSG00000198947 | DMD | turquoise |
| ENSG00000198948 | MFAP3L | turquoise |
| ENSG00000198952 | SMG5 | turquoise |
| ENSG00000198954 | KIF1BP | turquoise |
| ENSG00000198961 | PJA2 | turquoise |
| ENSG00000198964 | SGMS1 | turquoise |
| ENSG00000199023 | MIR339 | turquoise |
| ENSG00000199133 | MIRLET7D | turquoise |
| ENSG00000199266 | SNORA60 | turquoise |
| ENSG00000199804 | RNA5SP383 | turquoise |
| ENSG00000199906 | RNU5B-2P | turquoise |
| ENSG00000200534 | SNORA33 | turquoise |
| ENSG00000200651 | Y_RNA | turquoise |
| ENSG00000200714 | Y_RNA | turquoise |
| ENSG00000200879 | SNORD14E | turquoise |
| ENSG00000200913 | SNORD46 | turquoise |
| ENSG00000201151 | SNORD56 | turquoise |
| ENSG00000201302 | SNORA65 | turquoise |
| ENSG00000202314 | SNORD6 | turquoise |
| ENSG00000202474 | RNA5SP283 | turquoise |
| ENSG00000203286 | Metazoa_SRP | turquoise |
| ENSG00000203326 | ZNF525 | turquoise |
| ENSG00000203414 | BTBD7P1 | turquoise |
| ENSG00000203644 | AC083799.1 | turquoise |
| ENSG00000203668 | CHML | turquoise |
| ENSG00000203684 | IBA57-AS1 | turquoise |
| ENSG00000203697 | CAPN8 | turquoise |
| ENSG00000203705 | TATDN3 | turquoise |
| ENSG00000203710 | CR1 | turquoise |
| ENSG00000203772 | SPRN | turquoise |
| ENSG00000203778 | FAM229B | turquoise |
| ENSG00000203780 | FANK1 | turquoise |
| ENSG00000203791 | EEF1AKMT2 | turquoise |
| ENSG00000203799 | CCDC162P | turquoise |
| ENSG00000203804 | ADAMTSL4-AS1 | turquoise |
| ENSG00000203865 | ATP1A1-AS1 | turquoise |

| | | |
|---|---|---|
| ENSG00000203867 | RBM20 | turquoise |
| ENSG00000203879 | GDI1 | turquoise |
| ENSG00000203880 | PCMTD2 | turquoise |
| ENSG00000203896 | LIME1 | turquoise |
| ENSG00000203965 | EFCAB7 | turquoise |
| ENSG00000203993 | ARRDC1-AS1 | turquoise |
| ENSG00000204001 | LCN8 | turquoise |
| ENSG00000204044 | SLC12A5-AS1 | turquoise |
| ENSG00000204054 | LINC00963 | turquoise |
| ENSG00000204072 | ARMCX7P | turquoise |
| ENSG00000204104 | TRAF3IP1 | turquoise |
| ENSG00000204116 | CHIC1 | turquoise |
| ENSG00000204120 | GIGYF2 | turquoise |
| ENSG00000204130 | RUFY2 | turquoise |
| ENSG00000204147 | ASAH2B | turquoise |
| ENSG00000204165 | CXorf65 | turquoise |
| ENSG00000204177 | BMS1P1 | turquoise |
| ENSG00000204209 | DAXX | turquoise |
| ENSG00000204217 | BMPR2 | turquoise |
| ENSG00000204241 | AP000911.1 | turquoise |
| ENSG00000204256 | BRD2 | turquoise |
| ENSG00000204262 | COL5A2 | turquoise |
| ENSG00000204271 | SPIN3 | turquoise |
| ENSG00000204282 | TNRC6C-AS1 | turquoise |
| ENSG00000204310 | AGPAT1 | turquoise |
| ENSG00000204316 | MRPL38 | turquoise |
| ENSG00000204356 | NELFE | turquoise |
| ENSG00000204370 | SDHD | turquoise |
| ENSG00000204397 | CARD16 | turquoise |
| ENSG00000204406 | MBD5 | turquoise |
| ENSG00000204435 | CSNK2B | turquoise |
| ENSG00000204463 | BAG6 | turquoise |
| ENSG00000204482 | LST1 | turquoise |
| ENSG00000204514 | ZNF814 | turquoise |
| ENSG00000204519 | ZNF551 | turquoise |
| ENSG00000204520 | MICA | turquoise |
| ENSG00000204524 | ZNF805 | turquoise |
| ENSG00000204536 | CCHCR1 | turquoise |
| ENSG00000204584 | AC027801.1 | turquoise |
| ENSG00000204604 | ZNF468 | turquoise |
| ENSG00000204611 | ZNF616 | turquoise |
| ENSG00000204642 | HLA-F | turquoise |
| ENSG00000204653 | ASPDH | turquoise |
| ENSG00000204677 | FAM153C | turquoise |
| ENSG00000204685 | STARD7-AS1 | turquoise |
| ENSG00000204758 | AC008429.1 | turquoise |
| ENSG00000204778 | CBWD4P | turquoise |
| ENSG00000204789 | ZNF204P | turquoise |

| | | |
|---|---|---|
| ENSG00000204791 | SMPD5 | turquoise |
| ENSG00000204822 | MRPL53 | turquoise |
| ENSG00000204839 | MROH6 | turquoise |
| ENSG00000204843 | DCTN1 | turquoise |
| ENSG00000204851 | PNMA8B | turquoise |
| ENSG00000204852 | TCTN1 | turquoise |
| ENSG00000204859 | ZBTB48 | turquoise |
| ENSG00000204860 | FAM201A | turquoise |
| ENSG00000204869 | IGFL4 | turquoise |
| ENSG00000204899 | MZT1 | turquoise |
| ENSG00000204920 | ZNF155 | turquoise |
| ENSG00000204922 | UQCC3 | turquoise |
| ENSG00000204923 | FBXO48 | turquoise |
| ENSG00000204930 | FAM221B | turquoise |
| ENSG00000204934 | ATP6V0E2-AS1 | turquoise |
| ENSG00000204954 | C12orf73 | turquoise |
| ENSG00000204959 | ARHGEF34P | turquoise |
| ENSG00000204991 | SPIRE2 | turquoise |
| ENSG00000205018 | AC092384.1 | turquoise |
| ENSG00000205022 | PABPN1L | turquoise |
| ENSG00000205056 | LINC02397 | turquoise |
| ENSG00000205060 | SLC35B4 | turquoise |
| ENSG00000205078 | SYCE1L | turquoise |
| ENSG00000205084 | TMEM231 | turquoise |
| ENSG00000205133 | TRIQK | turquoise |
| ENSG00000205155 | PSENEN | turquoise |
| ENSG00000205189 | ZBTB10 | turquoise |
| ENSG00000205208 | C4orf46 | turquoise |
| ENSG00000205213 | LGR4 | turquoise |
| ENSG00000205250 | E2F4 | turquoise |
| ENSG00000205268 | PDE7A | turquoise |
| ENSG00000205269 | TMEM170B | turquoise |
| ENSG00000205302 | SNX2 | turquoise |
| ENSG00000205339 | IPO7 | turquoise |
| ENSG00000205356 | TECPR1 | turquoise |
| ENSG00000205413 | SAMD9 | turquoise |
| ENSG00000205423 | CNEP1R1 | turquoise |
| ENSG00000205426 | KRT81 | turquoise |
| ENSG00000205517 | RGL3 | turquoise |
| ENSG00000205534 | SMG1P2 | turquoise |
| ENSG00000205537 | AC121338.1 | turquoise |
| ENSG00000205560 | CPT1B | turquoise |
| ENSG00000205571 | SMN2 | turquoise |
| ENSG00000205581 | HMGN1 | turquoise |
| ENSG00000205583 | STAG3L1 | turquoise |
| ENSG00000205593 | DENND6B | turquoise |
| ENSG00000205643 | CDPF1 | turquoise |
| ENSG00000205659 | LIN52 | turquoise |

| | | |
|---|---|---|
| ENSG00000205707 | ETFRF1 | turquoise |
| ENSG00000205710 | C17orf107 | turquoise |
| ENSG00000205730 | ITPRIPL2 | turquoise |
| ENSG00000205744 | DENND1C | turquoise |
| ENSG00000205765 | C5orf51 | turquoise |
| ENSG00000205784 | ARRDC5 | turquoise |
| ENSG00000205790 | DPP9-AS1 | turquoise |
| ENSG00000205808 | PLPP6 | turquoise |
| ENSG00000205832 | C16orf96 | turquoise |
| ENSG00000205846 | CLEC6A | turquoise |
| ENSG00000205853 | RFPL3S | turquoise |
| ENSG00000205871 | AC068724.1 | turquoise |
| ENSG00000205890 | AC108134.1 | turquoise |
| ENSG00000205903 | ZNF316 | turquoise |
| ENSG00000205913 | SRRM2-AS1 | turquoise |
| ENSG00000205978 | NYNRIN | turquoise |
| ENSG00000205981 | DNAJC19 | turquoise |
| ENSG00000206053 | JPT2 | turquoise |
| ENSG00000206113 | CFAP99 | turquoise |
| ENSG00000206140 | TMEM191C | turquoise |
| ENSG00000206417 | H1FX-AS1 | turquoise |
| ENSG00000206418 | RAB12 | turquoise |
| ENSG00000206503 | HLA-A | turquoise |
| ENSG00000206527 | HACD2 | turquoise |
| ENSG00000206559 | ZCWPW2 | turquoise |
| ENSG00000206560 | ANKRD28 | turquoise |
| ENSG00000206561 | COLQ | turquoise |
| ENSG00000206567 | AC022007.1 | turquoise |
| ENSG00000206762 | RNU6-418P | turquoise |
| ENSG00000207554 | MIR647 | turquoise |
| ENSG00000207652 | MIR621 | turquoise |
| ENSG00000207697 | MIR573 | turquoise |
| ENSG00000207736 | MIR657 | turquoise |
| ENSG00000207757 | MIR93 | turquoise |
| ENSG00000207808 | MIR27A | turquoise |
| ENSG00000207926 | MIR135A1 | turquoise |
| ENSG00000207980 | MIR23A | turquoise |
| ENSG00000210082 | MT-RNR2 | turquoise |
| ENSG00000211445 | GPX3 | turquoise |
| ENSG00000211451 | GNRHR2 | turquoise |
| ENSG00000211452 | DIO1 | turquoise |
| ENSG00000211455 | STK38L | turquoise |
| ENSG00000211456 | SACM1L | turquoise |
| ENSG00000211460 | TSN | turquoise |
| ENSG00000211584 | SLC48A1 | turquoise |
| ENSG00000211597 | IGKJ1 | turquoise |
| ENSG00000211632 | IGKV3D-11 | turquoise |
| ENSG00000211638 | IGLV8-61 | turquoise |

| | | |
|---|---|---|
| ENSG00000211642 | IGLV10-54 | turquoise |
| ENSG00000211659 | IGLV3-25 | turquoise |
| ENSG00000211666 | IGLV2-14 | turquoise |
| ENSG00000211676 | IGLJ2 | turquoise |
| ENSG00000211683 | AP000346.1 | turquoise |
| ENSG00000211685 | IGLC7 | turquoise |
| ENSG00000211692 | TRGJP1 | turquoise |
| ENSG00000211694 | TRGV10 | turquoise |
| ENSG00000211695 | TRGV9 | turquoise |
| ENSG00000211699 | TRGV3 | turquoise |
| ENSG00000211706 | TRBV6-1 | turquoise |
| ENSG00000211710 | TRBV4-1 | turquoise |
| ENSG00000211721 | TRBV6-5 | turquoise |
| ENSG00000211724 | TRBV6-6 | turquoise |
| ENSG00000211725 | TRBV5-5 | turquoise |
| ENSG00000211747 | TRBV20-1 | turquoise |
| ENSG00000211751 | AC245427.1 | turquoise |
| ENSG00000211765 | TRBJ2-2 | turquoise |
| ENSG00000211782 | TRAV8-1 | turquoise |
| ENSG00000211784 | TRAV10 | turquoise |
| ENSG00000211786 | TRAV8-2 | turquoise |
| ENSG00000211792 | TRAV14DV4 | turquoise |
| ENSG00000211796 | TRAV16 | turquoise |
| ENSG00000211798 | TRAV18 | turquoise |
| ENSG00000211802 | TRAV22 | turquoise |
| ENSG00000211806 | TRAV25 | turquoise |
| ENSG00000211813 | TRAV34 | turquoise |
| ENSG00000211815 | TRAV36DV7 | turquoise |
| ENSG00000211828 | TRDJ3 | turquoise |
| ENSG00000211883 | TRAJ6 | turquoise |
| ENSG00000211941 | IGHV3-11 | turquoise |
| ENSG00000212123 | PRR22 | turquoise |
| ENSG00000212588 | SNORA26 | turquoise |
| ENSG00000212664 | AC064799.1 | turquoise |
| ENSG00000212719 | C17orf51 | turquoise |
| ENSG00000212907 | MT-ND4L | turquoise |
| ENSG00000212916 | MAP10 | turquoise |
| ENSG00000212993 | POU5F1B | turquoise |
| ENSG00000212994 | RPS26P6 | turquoise |
| ENSG00000213015 | ZNF580 | turquoise |
| ENSG00000213026 | CFL1P4 | turquoise |
| ENSG00000213036 | AL445305.1 | turquoise |
| ENSG00000213047 | DENND1B | turquoise |
| ENSG00000213064 | SFT2D2 | turquoise |
| ENSG00000213079 | SCAF8 | turquoise |
| ENSG00000213085 | CFAP45 | turquoise |
| ENSG00000213096 | ZNF254 | turquoise |
| ENSG00000213139 | CRYGS | turquoise |

| | | |
|---|---|---|
| ENSG00000213160 | KLHL23 | turquoise |
| ENSG00000213185 | FAM24B | turquoise |
| ENSG00000213186 | TRIM59 | turquoise |
| ENSG00000213189 | BTF3L4P2 | turquoise |
| ENSG00000213199 | ASIC3 | turquoise |
| ENSG00000213213 | CCDC183 | turquoise |
| ENSG00000213214 | ARHGEF35 | turquoise |
| ENSG00000213221 | DNLZ | turquoise |
| ENSG00000213236 | YWHAZP2 | turquoise |
| ENSG00000213246 | SUPT4H1 | turquoise |
| ENSG00000213253 | RPL12P42 | turquoise |
| ENSG00000213261 | EEF1B2P6 | turquoise |
| ENSG00000213279 | Z97192.2 | turquoise |
| ENSG00000213281 | NRAS | turquoise |
| ENSG00000213290 | PGK1P2 | turquoise |
| ENSG00000213316 | LTC4S | turquoise |
| ENSG00000213341 | CHUK | turquoise |
| ENSG00000213366 | GSTM2 | turquoise |
| ENSG00000213373 | LINC00671 | turquoise |
| ENSG00000213380 | COG8 | turquoise |
| ENSG00000213390 | ARHGAP19 | turquoise |
| ENSG00000213397 | HAUS7 | turquoise |
| ENSG00000213398 | LCAT | turquoise |
| ENSG00000213399 | AC022210.1 | turquoise |
| ENSG00000213406 | ANXA2P1 | turquoise |
| ENSG00000213430 | HSPD1P1 | turquoise |
| ENSG00000213438 | YBX2P1 | turquoise |
| ENSG00000213445 | SIPA1 | turquoise |
| ENSG00000213463 | SYNJ2BP | turquoise |
| ENSG00000213468 | FIRRE | turquoise |
| ENSG00000213516 | RBMXL1 | turquoise |
| ENSG00000213523 | SRA1 | turquoise |
| ENSG00000213551 | DNAJC9 | turquoise |
| ENSG00000213563 | C8orf82 | turquoise |
| ENSG00000213590 | AL807752.1 | turquoise |
| ENSG00000213609 | RPL7AP50 | turquoise |
| ENSG00000213621 | RPSAP54 | turquoise |
| ENSG00000213625 | LEPROT | turquoise |
| ENSG00000213638 | ADAT3 | turquoise |
| ENSG00000213639 | PPP1CB | turquoise |
| ENSG00000213654 | GPSM3 | turquoise |
| ENSG00000213658 | LAT | turquoise |
| ENSG00000213672 | NCKIPSD | turquoise |
| ENSG00000213676 | ATF6B | turquoise |
| ENSG00000213700 | RPL17P50 | turquoise |
| ENSG00000213714 | FAM209B | turquoise |
| ENSG00000213719 | CLIC1 | turquoise |
| ENSG00000213740 | SERBP1P1 | turquoise |

| | | |
|---|---|---|
| ENSG00000213753 | CENPBD1P1 | turquoise |
| ENSG00000213759 | UGT2B11 | turquoise |
| ENSG00000213762 | ZNF134 | turquoise |
| ENSG00000213793 | ZNF888 | turquoise |
| ENSG00000213799 | ZNF845 | turquoise |
| ENSG00000213846 | AC098614.1 | turquoise |
| ENSG00000213860 | RPL21P75 | turquoise |
| ENSG00000213862 | AC044787.1 | turquoise |
| ENSG00000213876 | AC120057.1 | turquoise |
| ENSG00000213889 | PPM1N | turquoise |
| ENSG00000213903 | LTB4R | turquoise |
| ENSG00000213930 | GALT | turquoise |
| ENSG00000213937 | CLDN9 | turquoise |
| ENSG00000213942 | AC005840.1 | turquoise |
| ENSG00000213949 | ITGA1 | turquoise |
| ENSG00000213965 | NUDT19 | turquoise |
| ENSG00000213976 | AC010615.1 | turquoise |
| ENSG00000213977 | TAX1BP3 | turquoise |
| ENSG00000213983 | AP1G2 | turquoise |
| ENSG00000213985 | AC078899.1 | turquoise |
| ENSG00000213988 | ZNF90 | turquoise |
| ENSG00000213995 | NAXD | turquoise |
| ENSG00000213999 | MEF2B | turquoise |
| ENSG00000214018 | RRM2P3 | turquoise |
| ENSG00000214022 | REPIN1 | turquoise |
| ENSG00000214027 | ARPC3P5 | turquoise |
| ENSG00000214063 | TSPAN4 | turquoise |
| ENSG00000214076 | CPSF1P1 | turquoise |
| ENSG00000214078 | CPNE1 | turquoise |
| ENSG00000214087 | ARL16 | turquoise |
| ENSG00000214110 | LDHAP4 | turquoise |
| ENSG00000214114 | MYCBP | turquoise |
| ENSG00000214140 | PRCD | turquoise |
| ENSG00000214160 | ALG3 | turquoise |
| ENSG00000214185 | XPOTP1 | turquoise |
| ENSG00000214192 | UBE2V1P2 | turquoise |
| ENSG00000214193 | SH3D21 | turquoise |
| ENSG00000214194 | SMIM30 | turquoise |
| ENSG00000214199 | EEF1A1P12 | turquoise |
| ENSG00000214223 | HNRNPA1P10 | turquoise |
| ENSG00000214293 | APTR | turquoise |
| ENSG00000214300 | SPDYE3 | turquoise |
| ENSG00000214309 | MBLAC1 | turquoise |
| ENSG00000214367 | HAUS3 | turquoise |
| ENSG00000214389 | RPS3AP26 | turquoise |
| ENSG00000214455 | RCN1P2 | turquoise |
| ENSG00000214485 | RPL7P1 | turquoise |
| ENSG00000214517 | PPME1 | turquoise |

| | | |
|---|---|---|
| ENSG00000214530 | STARD10 | turquoise |
| ENSG00000214552 | COPS8P2 | turquoise |
| ENSG00000214562 | NUTM2D | turquoise |
| ENSG00000214688 | C10orf105 | turquoise |
| ENSG00000214756 | METTL12 | turquoise |
| ENSG00000214765 | SEPT7P2 | turquoise |
| ENSG00000214783 | POLR2J4 | turquoise |
| ENSG00000214784 | AC010468.1 | turquoise |
| ENSG00000214787 | MS4A4E | turquoise |
| ENSG00000214797 | AP002358.1 | turquoise |
| ENSG00000214846 | AC114744.1 | turquoise |
| ENSG00000214867 | SRSF9P1 | turquoise |
| ENSG00000214941 | ZSWIM7 | turquoise |
| ENSG00000214954 | LRRC69 | turquoise |
| ENSG00000214960 | ISPD | turquoise |
| ENSG00000214973 | CHCHD3P3 | turquoise |
| ENSG00000215014 | AL645728.1 | turquoise |
| ENSG00000215022 | AL008729.1 | turquoise |
| ENSG00000215039 | CD27-AS1 | turquoise |
| ENSG00000215041 | NEURL4 | turquoise |
| ENSG00000215114 | UBXN2B | turquoise |
| ENSG00000215126 | CBWD6 | turquoise |
| ENSG00000215210 | RBMXP2 | turquoise |
| ENSG00000215251 | FASTKD5 | turquoise |
| ENSG00000215283 | HMGB3P24 | turquoise |
| ENSG00000215301 | DDX3X | turquoise |
| ENSG00000215305 | VPS16 | turquoise |
| ENSG00000215349 | MRPL3P1 | turquoise |
| ENSG00000215375 | MYL5 | turquoise |
| ENSG00000215414 | PSMA6P1 | turquoise |
| ENSG00000215417 | MIR17HG | turquoise |
| ENSG00000215421 | ZNF407 | turquoise |
| ENSG00000215440 | NPEPL1 | turquoise |
| ENSG00000215458 | AATBC | turquoise |
| ENSG00000215529 | EFCAB8 | turquoise |
| ENSG00000215571 | GRK6P1 | turquoise |
| ENSG00000215704 | CELA2B | turquoise |
| ENSG00000215712 | TMEM242 | turquoise |
| ENSG00000215717 | TMEM167B | turquoise |
| ENSG00000215769 | ARHGAP27P1-BPT | turquoise |
| ENSG00000215784 | FAM72D | turquoise |
| ENSG00000215788 | TNFRSF25 | turquoise |
| ENSG00000215817 | ZC3H11B | turquoise |
| ENSG00000215908 | CROCCP2 | turquoise |
| ENSG00000215915 | ATAD3C | turquoise |
| ENSG00000216285 | AC078819.1 | turquoise |
| ENSG00000216490 | IFI30 | turquoise |
| ENSG00000216588 | IGSF23 | turquoise |

| | | |
|---|---|---|
| ENSG00000216775 | AL109918.1 | turquoise |
| ENSG00000216863 | LY86-AS1 | turquoise |
| ENSG00000217027 | TPT1P4 | turquoise |
| ENSG00000217078 | AL021407.1 | turquoise |
| ENSG00000217128 | FNIP1 | turquoise |
| ENSG00000217165 | ANKRD18EP | turquoise |
| ENSG00000217241 | CBX3P9 | turquoise |
| ENSG00000217416 | ISCA1P1 | turquoise |
| ENSG00000217644 | AL355864.1 | turquoise |
| ENSG00000217648 | AL136116.3 | turquoise |
| ENSG00000217702 | AC073263.1 | turquoise |
| ENSG00000217801 | AL390719.1 | turquoise |
| ENSG00000218073 | AL021407.3 | turquoise |
| ENSG00000218283 | MORF4L1P1 | turquoise |
| ENSG00000218350 | LYPLA1P3 | turquoise |
| ENSG00000218418 | AL591135.1 | turquoise |
| ENSG00000218426 | AL590867.2 | turquoise |
| ENSG00000218739 | CEBPZOS | turquoise |
| ENSG00000218891 | ZNF579 | turquoise |
| ENSG00000219200 | RNASEK | turquoise |
| ENSG00000219201 | AC138392.1 | turquoise |
| ENSG00000219451 | RPL23P8 | turquoise |
| ENSG00000219470 | AL355802.2 | turquoise |
| ENSG00000219481 | NBPF1 | turquoise |
| ENSG00000219507 | FTH1P8 | turquoise |
| ENSG00000219545 | UMAD1 | turquoise |
| ENSG00000219607 | PPP1R3G | turquoise |
| ENSG00000220201 | ZGLP1 | turquoise |
| ENSG00000220517 | ASS1P1 | turquoise |
| ENSG00000220563 | PKMP3 | turquoise |
| ENSG00000220749 | RPL21P28 | turquoise |
| ENSG00000220842 | RPL21P16 | turquoise |
| ENSG00000220848 | RPS18P9 | turquoise |
| ENSG00000221500 | SNORD100 | turquoise |
| ENSG00000221598 | MIR1249 | turquoise |
| ENSG00000221829 | FANCG | turquoise |
| ENSG00000221843 | C2orf16 | turquoise |
| ENSG00000221866 | PLXNA4 | turquoise |
| ENSG00000221869 | CEBPD | turquoise |
| ENSG00000221883 | ARIH2OS | turquoise |
| ENSG00000221886 | ZBED8 | turquoise |
| ENSG00000221909 | FAM200A | turquoise |
| ENSG00000221916 | C19orf73 | turquoise |
| ENSG00000221923 | ZNF880 | turquoise |
| ENSG00000221926 | TRIM16 | turquoise |
| ENSG00000221930 | FAM45BP | turquoise |
| ENSG00000221946 | FXYD7 | turquoise |
| ENSG00000221968 | FADS3 | turquoise |

| | | |
|---|---|---|
| ENSG00000222020 | AC062017.1 | turquoise |
| ENSG00000222041 | CYTOR | turquoise |
| ENSG00000222267 | RNU6-892P | turquoise |
| ENSG00000222808 | RNU4-47P | turquoise |
| ENSG00000223274 | RNA5SP498 | turquoise |
| ENSG00000223350 | IGLV9-49 | turquoise |
| ENSG00000223361 | FTH1P10 | turquoise |
| ENSG00000223374 | AC005104.1 | turquoise |
| ENSG00000223478 | AL441992.1 | turquoise |
| ENSG00000223496 | EXOSC6 | turquoise |
| ENSG00000223509 | AC135983.2 | turquoise |
| ENSG00000223510 | CDRT15 | turquoise |
| ENSG00000223546 | LINC00630 | turquoise |
| ENSG00000223553 | SMPD4P1 | turquoise |
| ENSG00000223559 | AC073136.1 | turquoise |
| ENSG00000223573 | TINCR | turquoise |
| ENSG00000223705 | NSUN5P1 | turquoise |
| ENSG00000223711 | AC069213.1 | turquoise |
| ENSG00000223768 | LINC00205 | turquoise |
| ENSG00000223773 | CD99P1 | turquoise |
| ENSG00000223804 | AC244669.1 | turquoise |
| ENSG00000223823 | LINC01342 | turquoise |
| ENSG00000223825 | DAZAP2P1 | turquoise |
| ENSG00000223884 | AC068481.1 | turquoise |
| ENSG00000223891 | OSER1-AS1 | turquoise |
| ENSG00000223960 | AC009948.1 | turquoise |
| ENSG00000223969 | AC002456.1 | turquoise |
| ENSG00000224003 | YES1P1 | turquoise |
| ENSG00000224051 | CPTP | turquoise |
| ENSG00000224078 | SNHG14 | turquoise |
| ENSG00000224080 | UBE2FP1 | turquoise |
| ENSG00000224097 | AC021148.1 | turquoise |
| ENSG00000224114 | AL591846.1 | turquoise |
| ENSG00000224165 | DNAJC27-AS1 | turquoise |
| ENSG00000224203 | RPS23P10 | turquoise |
| ENSG00000224261 | RPSAP18 | turquoise |
| ENSG00000224281 | SLC25A5-AS1 | turquoise |
| ENSG00000224295 | OLFM5P | turquoise |
| ENSG00000224307 | AL161785.1 | turquoise |
| ENSG00000224383 | PRR29 | turquoise |
| ENSG00000224411 | HSP90AA2P | turquoise |
| ENSG00000224418 | STK24-AS1 | turquoise |
| ENSG00000224420 | ADM5 | turquoise |
| ENSG00000224424 | PRKAR2A-AS1 | turquoise |
| ENSG00000224470 | ATXN1L | turquoise |
| ENSG00000224478 | AL356417.1 | turquoise |
| ENSG00000224531 | SMIM13 | turquoise |
| ENSG00000224533 | TMLHE-AS1 | turquoise |

| | | |
|---|---|---|
| ENSG00000224536 | AC096677.1 | turquoise |
| ENSG00000224546 | EIF4BP3 | turquoise |
| ENSG00000224550 | AC114491.1 | turquoise |
| ENSG00000224596 | ZMIZ1-AS1 | turquoise |
| ENSG00000224607 | IGKV1D-27 | turquoise |
| ENSG00000224614 | TNK2-AS1 | turquoise |
| ENSG00000224631 | RPS27AP16 | turquoise |
| ENSG00000224738 | AC099850.1 | turquoise |
| ENSG00000224858 | RPL29P11 | turquoise |
| ENSG00000224863 | LINC01398 | turquoise |
| ENSG00000224870 | AL391244.1 | turquoise |
| ENSG00000224897 | POT1-AS1 | turquoise |
| ENSG00000224934 | AL391684.1 | turquoise |
| ENSG00000224940 | PRRT4 | turquoise |
| ENSG00000224945 | AL353150.1 | turquoise |
| ENSG00000224950 | AL390066.1 | turquoise |
| ENSG00000224976 | PARP4P2 | turquoise |
| ENSG00000224992 | AL445645.1 | turquoise |
| ENSG00000225018 | AC092647.1 | turquoise |
| ENSG00000225022 | UBE2D3P1 | turquoise |
| ENSG00000225031 | EIF4BP7 | turquoise |
| ENSG00000225071 | AC004552.1 | turquoise |
| ENSG00000225099 | ATP6V1E1P1 | turquoise |
| ENSG00000225125 | RANP4 | turquoise |
| ENSG00000225137 | DYNC1I2P1 | turquoise |
| ENSG00000225138 | SLC9A3-AS1 | turquoise |
| ENSG00000225159 | NPM1P39 | turquoise |
| ENSG00000225171 | DUTP6 | turquoise |
| ENSG00000225187 | AC073283.1 | turquoise |
| ENSG00000225194 | LINC00092 | turquoise |
| ENSG00000225217 | HSPA7 | turquoise |
| ENSG00000225218 | AP001628.1 | turquoise |
| ENSG00000225331 | LINC01678 | turquoise |
| ENSG00000225399 | AC121247.1 | turquoise |
| ENSG00000225402 | AC010878.1 | turquoise |
| ENSG00000225439 | BOLA3-AS1 | turquoise |
| ENSG00000225476 | MTCO3P5 | turquoise |
| ENSG00000225489 | AL354707.1 | turquoise |
| ENSG00000225507 | AC069282.1 | turquoise |
| ENSG00000225523 | IGKV6D-21 | turquoise |
| ENSG00000225568 | AC093155.1 | turquoise |
| ENSG00000225578 | NCBP2-AS1 | turquoise |
| ENSG00000225614 | ZNF469 | turquoise |
| ENSG00000225630 | MTND2P28 | turquoise |
| ENSG00000225643 | AL606491.1 | turquoise |
| ENSG00000225648 | SBDSP1 | turquoise |
| ENSG00000225675 | LINC01771 | turquoise |
| ENSG00000225690 | TREML5P | turquoise |

| | | |
|---|---|---|
| ENSG00000225697 | SLC26A6 | turquoise |
| ENSG00000225733 | FGD5-AS1 | turquoise |
| ENSG00000225756 | DBH-AS1 | turquoise |
| ENSG00000225808 | DNAJC19P5 | turquoise |
| ENSG00000225828 | FAM229A | turquoise |
| ENSG00000225830 | ERCC6 | turquoise |
| ENSG00000225872 | LINC01529 | turquoise |
| ENSG00000225889 | AC012368.1 | turquoise |
| ENSG00000225936 | AL731557.1 | turquoise |
| ENSG00000225938 | AL109741.1 | turquoise |
| ENSG00000225973 | PIGBOS1 | turquoise |
| ENSG00000226049 | AC123769.1 | turquoise |
| ENSG00000226084 | AC113935.1 | turquoise |
| ENSG00000226086 | EIF3LP3 | turquoise |
| ENSG00000226091 | LINC00937 | turquoise |
| ENSG00000226098 | SEC11B | turquoise |
| ENSG00000226124 | FTCDNL1 | turquoise |
| ENSG00000226125 | LINC01907 | turquoise |
| ENSG00000226137 | BAIAP2-AS1 | turquoise |
| ENSG00000226174 | TEX22 | turquoise |
| ENSG00000226179 | LINC00685 | turquoise |
| ENSG00000226180 | AC010536.1 | turquoise |
| ENSG00000226221 | AC022431.1 | turquoise |
| ENSG00000226252 | AL135960.1 | turquoise |
| ENSG00000226261 | AC064836.1 | turquoise |
| ENSG00000226268 | AC135977.1 | turquoise |
| ENSG00000226284 | ARPC3P1 | turquoise |
| ENSG00000226314 | ZNF192P1 | turquoise |
| ENSG00000226328 | NUP50-AS1 | turquoise |
| ENSG00000226329 | AC005682.1 | turquoise |
| ENSG00000226332 | AL354836.1 | turquoise |
| ENSG00000226396 | AL031727.1 | turquoise |
| ENSG00000226469 | ADAM1B | turquoise |
| ENSG00000226525 | RPS7P10 | turquoise |
| ENSG00000226564 | FTH1P20 | turquoise |
| ENSG00000226706 | AL161452.1 | turquoise |
| ENSG00000226711 | FAM66C | turquoise |
| ENSG00000226777 | FAM30A | turquoise |
| ENSG00000226784 | PGAM4 | turquoise |
| ENSG00000226791 | AC109826.1 | turquoise |
| ENSG00000226824 | AC006001.2 | turquoise |
| ENSG00000226828 | AL591885.1 | turquoise |
| ENSG00000226833 | AC092164.1 | turquoise |
| ENSG00000226849 | AL109811.1 | turquoise |
| ENSG00000226862 | AC104463.2 | turquoise |
| ENSG00000226942 | IL9RP3 | turquoise |
| ENSG00000226964 | RHEBP2 | turquoise |
| ENSG00000226976 | COX6A1P2 | turquoise |

| | | |
|---|---|---|
| ENSG00000226981 | ABHD17AP6 | turquoise |
| ENSG00000227008 | AL009174.1 | turquoise |
| ENSG00000227039 | ITGB2-AS1 | turquoise |
| ENSG00000227063 | RPL41P1 | turquoise |
| ENSG00000227076 | AL158166.1 | turquoise |
| ENSG00000227081 | AC005912.1 | turquoise |
| ENSG00000227159 | DDX11L16 | turquoise |
| ENSG00000227189 | AC092535.1 | turquoise |
| ENSG00000227195 | MIR663AHG | turquoise |
| ENSG00000227201 | CNN2P1 | turquoise |
| ENSG00000227203 | SUB1P1 | turquoise |
| ENSG00000227218 | AL157935.1 | turquoise |
| ENSG00000227232 | WASH7P | turquoise |
| ENSG00000227295 | ELL2P1 | turquoise |
| ENSG00000227329 | AL139396.1 | turquoise |
| ENSG00000227339 | THRAP3P1 | turquoise |
| ENSG00000227345 | PARG | turquoise |
| ENSG00000227354 | RBM26-AS1 | turquoise |
| ENSG00000227359 | AC017074.1 | turquoise |
| ENSG00000227372 | TP73-AS1 | turquoise |
| ENSG00000227376 | FTH1P16 | turquoise |
| ENSG00000227388 | AL133410.1 | turquoise |
| ENSG00000227398 | KIF9-AS1 | turquoise |
| ENSG00000227403 | LINC01806 | turquoise |
| ENSG00000227456 | LINC00310 | turquoise |
| ENSG00000227473 | TSSK5P | turquoise |
| ENSG00000227495 | AC004771.1 | turquoise |
| ENSG00000227500 | SCAMP4 | turquoise |
| ENSG00000227540 | DNAJC9-AS1 | turquoise |
| ENSG00000227591 | AL031316.1 | turquoise |
| ENSG00000227725 | GCOM2 | turquoise |
| ENSG00000227740 | AL513329.1 | turquoise |
| ENSG00000227741 | AL121987.2 | turquoise |
| ENSG00000227788 | MTCO3P43 | turquoise |
| ENSG00000227827 | AC138969.2 | turquoise |
| ENSG00000227848 | SUCLA2-AS1 | turquoise |
| ENSG00000227850 | SEPT2P1 | turquoise |
| ENSG00000227896 | AL731569.1 | turquoise |
| ENSG00000227963 | AL355488.1 | turquoise |
| ENSG00000227986 | TRIM60P18 | turquoise |
| ENSG00000228058 | LINC01736 | turquoise |
| ENSG00000228063 | LYPLAL1-AS1 | turquoise |
| ENSG00000228106 | AL392172.1 | turquoise |
| ENSG00000228223 | HCG11 | turquoise |
| ENSG00000228232 | GAPDHP1 | turquoise |
| ENSG00000228253 | MT-ATP8 | turquoise |
| ENSG00000228278 | ORM2 | turquoise |
| ENSG00000228283 | KATNBL1P6 | turquoise |

| | | |
|---|---|---|
| ENSG00000228329 | LINC01890 | turquoise |
| ENSG00000228343 | AC115618.2 | turquoise |
| ENSG00000228393 | LINC01004 | turquoise |
| ENSG00000228409 | CCT6P1 | turquoise |
| ENSG00000228451 | SDAD1P1 | turquoise |
| ENSG00000228477 | AL663070.1 | turquoise |
| ENSG00000228486 | LINC01125 | turquoise |
| ENSG00000228495 | LINC01013 | turquoise |
| ENSG00000228501 | RPL15P18 | turquoise |
| ENSG00000228502 | EEF1A1P11 | turquoise |
| ENSG00000228519 | AC097263.1 | turquoise |
| ENSG00000228544 | CCDC183-AS1 | turquoise |
| ENSG00000228570 | NUTM2E | turquoise |
| ENSG00000228589 | SPCS2P4 | turquoise |
| ENSG00000228623 | ZNF883 | turquoise |
| ENSG00000228655 | AC096558.1 | turquoise |
| ENSG00000228661 | AC090587.1 | turquoise |
| ENSG00000228672 | PROB1 | turquoise |
| ENSG00000228692 | AL445307.1 | turquoise |
| ENSG00000228818 | AL359918.1 | turquoise |
| ENSG00000228839 | PIK3IP1-AS1 | turquoise |
| ENSG00000228847 | ATP5G2P4 | turquoise |
| ENSG00000228897 | AC012441.2 | turquoise |
| ENSG00000228903 | RASA4CP | turquoise |
| ENSG00000228981 | AC097658.1 | turquoise |
| ENSG00000229000 | SEPT7P8 | turquoise |
| ENSG00000229043 | AC091729.3 | turquoise |
| ENSG00000229056 | AC020571.1 | turquoise |
| ENSG00000229106 | BTBD6P1 | turquoise |
| ENSG00000229107 | ABHD17AP4 | turquoise |
| ENSG00000229117 | RPL41 | turquoise |
| ENSG00000229122 | AGBL5-IT1 | turquoise |
| ENSG00000229124 | VIM-AS1 | turquoise |
| ENSG00000229151 | AC233976.1 | turquoise |
| ENSG00000229162 | AL445471.1 | turquoise |
| ENSG00000229180 | AC006001.3 | turquoise |
| ENSG00000229320 | KRT8P12 | turquoise |
| ENSG00000229344 | MTCO2P12 | turquoise |
| ENSG00000229388 | LINC01715 | turquoise |
| ENSG00000229413 | AC018638.1 | turquoise |
| ENSG00000229419 | RALGAPA1P1 | turquoise |
| ENSG00000229453 | SPINK8 | turquoise |
| ENSG00000229515 | FLT1P1 | turquoise |
| ENSG00000229605 | RPL21P93 | turquoise |
| ENSG00000229635 | AL137159.1 | turquoise |
| ENSG00000229644 | NAMPTP1 | turquoise |
| ENSG00000229648 | RPSAP22 | turquoise |
| ENSG00000229671 | LINC01150 | turquoise |

| | | |
|---|---|---|
| ENSG00000229692 | SOS1-IT1 | turquoise |
| ENSG00000229719 | MIR194-2HG | turquoise |
| ENSG00000229932 | YWHAZP3 | turquoise |
| ENSG00000229944 | EIF4EP2 | turquoise |
| ENSG00000229980 | TOB1-AS1 | turquoise |
| ENSG00000229994 | RPL5P4 | turquoise |
| ENSG00000230061 | TRPM2-AS | turquoise |
| ENSG00000230076 | RPL10P6 | turquoise |
| ENSG00000230082 | PRRT3-AS1 | turquoise |
| ENSG00000230099 | TRBV5-4 | turquoise |
| ENSG00000230107 | AL022316.1 | turquoise |
| ENSG00000230138 | AC119428.2 | turquoise |
| ENSG00000230148 | HOXB-AS1 | turquoise |
| ENSG00000230154 | AC018463.1 | turquoise |
| ENSG00000230163 | AL122010.1 | turquoise |
| ENSG00000230183 | CNOT6LP1 | turquoise |
| ENSG00000230204 | FTH1P5 | turquoise |
| ENSG00000230262 | MIRLET7DHG | turquoise |
| ENSG00000230289 | AL358781.2 | turquoise |
| ENSG00000230291 | AC078817.1 | turquoise |
| ENSG00000230311 | TOMM20P4 | turquoise |
| ENSG00000230383 | AC009245.1 | turquoise |
| ENSG00000230409 | TCEA1P2 | turquoise |
| ENSG00000230513 | THAP7-AS1 | turquoise |
| ENSG00000230537 | AL158071.2 | turquoise |
| ENSG00000230555 | AL450326.1 | turquoise |
| ENSG00000230561 | CCDC192 | turquoise |
| ENSG00000230562 | FAM133DP | turquoise |
| ENSG00000230590 | FTX | turquoise |
| ENSG00000230626 | AC011005.1 | turquoise |
| ENSG00000230629 | RPS23P8 | turquoise |
| ENSG00000230667 | SETSIP | turquoise |
| ENSG00000230715 | AC018638.2 | turquoise |
| ENSG00000230730 | AC074011.1 | turquoise |
| ENSG00000230733 | AC092171.2 | turquoise |
| ENSG00000230734 | RPL10P3 | turquoise |
| ENSG00000230747 | AC021188.1 | turquoise |
| ENSG00000230756 | RHOQP3 | turquoise |
| ENSG00000230793 | SMARCE1P5 | turquoise |
| ENSG00000230797 | YY2 | turquoise |
| ENSG00000230825 | AC005532.1 | turquoise |
| ENSG00000230844 | ZNF674-AS1 | turquoise |
| ENSG00000230869 | AGAP10P | turquoise |
| ENSG00000230970 | HHATL-AS1 | turquoise |
| ENSG00000231062 | AC103563.2 | turquoise |
| ENSG00000231064 | AC234582.1 | turquoise |
| ENSG00000231084 | AL136133.1 | turquoise |
| ENSG00000231113 | AL035587.1 | turquoise |

| | | |
|---|---|---|
| ENSG00000231133 | HAR1B | turquoise |
| ENSG00000231154 | MORF4L2-AS1 | turquoise |
| ENSG00000231167 | YBX1P2 | turquoise |
| ENSG00000231177 | LINC00852 | turquoise |
| ENSG00000231205 | ZNF826P | turquoise |
| ENSG00000231259 | AC125232.1 | turquoise |
| ENSG00000231304 | SGO1-AS1 | turquoise |
| ENSG00000231305 | AC112484.2 | turquoise |
| ENSG00000231313 | AC078875.1 | turquoise |
| ENSG00000231327 | LINC01816 | turquoise |
| ENSG00000231345 | BEND3P1 | turquoise |
| ENSG00000231351 | AC111200.2 | turquoise |
| ENSG00000231437 | LINC01750 | turquoise |
| ENSG00000231439 | WASIR2 | turquoise |
| ENSG00000231443 | AC124944.2 | turquoise |
| ENSG00000231445 | TIMM8AP1 | turquoise |
| ENSG00000231507 | LINC01353 | turquoise |
| ENSG00000231563 | AL670729.1 | turquoise |
| ENSG00000231584 | FAHD2CP | turquoise |
| ENSG00000231595 | AC005224.1 | turquoise |
| ENSG00000231680 | AP003774.3 | turquoise |
| ENSG00000231682 | LINC01891 | turquoise |
| ENSG00000231684 | EIF1P3 | turquoise |
| ENSG00000231686 | Z97180.1 | turquoise |
| ENSG00000231752 | EMBP1 | turquoise |
| ENSG00000231770 | TMEM44-AS1 | turquoise |
| ENSG00000231784 | DBIL5P | turquoise |
| ENSG00000231793 | DOC2GP | turquoise |
| ENSG00000231822 | AC019097.1 | turquoise |
| ENSG00000231830 | AC245140.1 | turquoise |
| ENSG00000231841 | FAM192BP | turquoise |
| ENSG00000231851 | UTAT33 | turquoise |
| ENSG00000231856 | AL162377.1 | turquoise |
| ENSG00000231890 | DARS-AS1 | turquoise |
| ENSG00000231999 | FLJ27354 | turquoise |
| ENSG00000232022 | FAAHP1 | turquoise |
| ENSG00000232024 | LSM12P1 | turquoise |
| ENSG00000232060 | SLC4A1APP1 | turquoise |
| ENSG00000232065 | LINC01063 | turquoise |
| ENSG00000232112 | TMA7 | turquoise |
| ENSG00000232119 | MCTS1 | turquoise |
| ENSG00000232125 | DYTN | turquoise |
| ENSG00000232176 | AL161909.1 | turquoise |
| ENSG00000232187 | FTH1P7 | turquoise |
| ENSG00000232237 | ASCL5 | turquoise |
| ENSG00000232259 | AL158166.2 | turquoise |
| ENSG00000232311 | AL512303.1 | turquoise |
| ENSG00000232346 | Z74021.1 | turquoise |

| | | |
|---|---|---|
| ENSG00000232368 | FTLP2 | turquoise |
| ENSG00000232453 | AC105277.1 | turquoise |
| ENSG00000232472 | EEF1B2P3 | turquoise |
| ENSG00000232499 | AL391058.1 | turquoise |
| ENSG00000232533 | AC093673.1 | turquoise |
| ENSG00000232578 | AC093311.1 | turquoise |
| ENSG00000232593 | KANTR | turquoise |
| ENSG00000232613 | AC007386.2 | turquoise |
| ENSG00000232618 | AL355304.1 | turquoise |
| ENSG00000232645 | LINC01431 | turquoise |
| ENSG00000232677 | LINC00665 | turquoise |
| ENSG00000232702 | AL158050.1 | turquoise |
| ENSG00000232706 | NUTM2HP | turquoise |
| ENSG00000232725 | U52111.1 | turquoise |
| ENSG00000232742 | RHOQP2 | turquoise |
| ENSG00000232748 | AC135050.1 | turquoise |
| ENSG00000232759 | AC002480.2 | turquoise |
| ENSG00000232788 | AC078883.3 | turquoise |
| ENSG00000232807 | AL137186.2 | turquoise |
| ENSG00000232811 | AL360270.1 | turquoise |
| ENSG00000232818 | RPS2P32 | turquoise |
| ENSG00000232838 | PET117 | turquoise |
| ENSG00000232855 | AF165147.1 | turquoise |
| ENSG00000232859 | LYRM9 | turquoise |
| ENSG00000232888 | AC127070.1 | turquoise |
| ENSG00000232907 | DLGAP4-AS1 | turquoise |
| ENSG00000232952 | AL512844.1 | turquoise |
| ENSG00000232973 | CYP1B1-AS1 | turquoise |
| ENSG00000233024 | AC126755.2 | turquoise |
| ENSG00000233038 | AC011899.2 | turquoise |
| ENSG00000233058 | LINC00884 | turquoise |
| ENSG00000233084 | RPL23AP25 | turquoise |
| ENSG00000233098 | CCDC144NL-AS1 | turquoise |
| ENSG00000233154 | LINC01762 | turquoise |
| ENSG00000233184 | AC093157.1 | turquoise |
| ENSG00000233198 | RNF224 | turquoise |
| ENSG00000233223 | AC016876.1 | turquoise |
| ENSG00000233225 | AC004987.2 | turquoise |
| ENSG00000233229 | CNOT7P1 | turquoise |
| ENSG00000233230 | AC079807.2 | turquoise |
| ENSG00000233247 | AC002524.1 | turquoise |
| ENSG00000233250 | AC233728.1 | turquoise |
| ENSG00000233261 | FAM238A | turquoise |
| ENSG00000233270 | SNRPEP4 | turquoise |
| ENSG00000233297 | RASA4DP | turquoise |
| ENSG00000233355 | CHRM3-AS2 | turquoise |
| ENSG00000233387 | AL121748.1 | turquoise |
| ENSG00000233452 | STXBP5-AS1 | turquoise |

| | | |
|---|---|---|
| ENSG00000233469 | ST6GALNAC4P1 | turquoise |
| ENSG00000233476 | EEF1A1P6 | turquoise |
| ENSG00000233554 | B4GALT1-AS1 | turquoise |
| ENSG00000233597 | AC133435.1 | turquoise |
| ENSG00000233621 | LINC01137 | turquoise |
| ENSG00000233622 | CYP2T1P | turquoise |
| ENSG00000233665 | AC060234.2 | turquoise |
| ENSG00000233680 | HNRNPA1P27 | turquoise |
| ENSG00000233695 | GAS6-AS1 | turquoise |
| ENSG00000233730 | LINC01765 | turquoise |
| ENSG00000233746 | LINC00656 | turquoise |
| ENSG00000233775 | AL109945.1 | turquoise |
| ENSG00000233830 | EIF4HP1 | turquoise |
| ENSG00000233834 | AC005083.1 | turquoise |
| ENSG00000233837 | EIF3LP2 | turquoise |
| ENSG00000233846 | AL133480.2 | turquoise |
| ENSG00000233895 | AL121761.1 | turquoise |
| ENSG00000233901 | LINC01503 | turquoise |
| ENSG00000233913 | RPL10P9 | turquoise |
| ENSG00000233954 | UQCRHL | turquoise |
| ENSG00000233966 | UBE2SP1 | turquoise |
| ENSG00000234009 | RPL5P34 | turquoise |
| ENSG00000234028 | AC062029.1 | turquoise |
| ENSG00000234072 | AC074117.1 | turquoise |
| ENSG00000234076 | TPRG1-AS1 | turquoise |
| ENSG00000234118 | RPL13AP6 | turquoise |
| ENSG00000234141 | AC006042.4 | turquoise |
| ENSG00000234160 | AL513165.1 | turquoise |
| ENSG00000234175 | AL355355.2 | turquoise |
| ENSG00000234184 | LINC01781 | turquoise |
| ENSG00000234199 | LINC01191 | turquoise |
| ENSG00000234222 | LIX1L-AS1 | turquoise |
| ENSG00000234284 | ZNF879 | turquoise |
| ENSG00000234287 | AC099560.2 | turquoise |
| ENSG00000234322 | ST13P18 | turquoise |
| ENSG00000234332 | BCAS2P2 | turquoise |
| ENSG00000234337 | AC026462.1 | turquoise |
| ENSG00000234353 | AP000346.2 | turquoise |
| ENSG00000234354 | RPS26P47 | turquoise |
| ENSG00000234409 | CCDC188 | turquoise |
| ENSG00000234432 | AC092171.3 | turquoise |
| ENSG00000234444 | ZNF736 | turquoise |
| ENSG00000234465 | PINLYP | turquoise |
| ENSG00000234492 | RPL34-AS1 | turquoise |
| ENSG00000234498 | RPL13AP20 | turquoise |
| ENSG00000234500 | AC008267.3 | turquoise |
| ENSG00000234545 | FAM133B | turquoise |
| ENSG00000234608 | MAPKAPK5-AS1 | turquoise |

| | | |
|---|---|---|
| ENSG00000234618 | RPSAP9 | turquoise |
| ENSG00000234636 | MED14OS | turquoise |
| ENSG00000234648 | AL162151.2 | turquoise |
| ENSG00000234663 | LINC01934 | turquoise |
| ENSG00000234678 | ELF3-AS1 | turquoise |
| ENSG00000234694 | AL139289.2 | turquoise |
| ENSG00000234705 | HMGA1P4 | turquoise |
| ENSG00000234741 | GAS5 | turquoise |
| ENSG00000234742 | AC144530.1 | turquoise |
| ENSG00000234771 | SLC25A25-AS1 | turquoise |
| ENSG00000234782 | TPT1P9 | turquoise |
| ENSG00000234797 | RPS3AP6 | turquoise |
| ENSG00000234801 | MORF4 | turquoise |
| ENSG00000234912 | SNHG20 | turquoise |
| ENSG00000234936 | AC010883.1 | turquoise |
| ENSG00000234965 | SHISA8 | turquoise |
| ENSG00000234975 | FTH1P2 | turquoise |
| ENSG00000235007 | AL161785.2 | turquoise |
| ENSG00000235016 | SEMA3F-AS1 | turquoise |
| ENSG00000235072 | AC012074.1 | turquoise |
| ENSG00000235095 | AC099654.2 | turquoise |
| ENSG00000235119 | AL138895.1 | turquoise |
| ENSG00000235162 | C12orf75 | turquoise |
| ENSG00000235174 | AC019205.3 | turquoise |
| ENSG00000235194 | PPP1R3E | turquoise |
| ENSG00000235217 | TSPY26P | turquoise |
| ENSG00000235237 | Z82188.2 | turquoise |
| ENSG00000235271 | LINC01422 | turquoise |
| ENSG00000235314 | LINC00957 | turquoise |
| ENSG00000235363 | SNRPGP10 | turquoise |
| ENSG00000235374 | SSR4P1 | turquoise |
| ENSG00000235437 | LINC01278 | turquoise |
| ENSG00000235459 | AC006463.2 | turquoise |
| ENSG00000235478 | LINC01664 | turquoise |
| ENSG00000235513 | AL035681.1 | turquoise |
| ENSG00000235552 | AP005202.1 | turquoise |
| ENSG00000235560 | AC002310.1 | turquoise |
| ENSG00000235576 | LINC01871 | turquoise |
| ENSG00000235609 | AF127577.4 | turquoise |
| ENSG00000235618 | FAM21EP | turquoise |
| ENSG00000235621 | LINC00494 | turquoise |
| ENSG00000235636 | NUS1P1 | turquoise |
| ENSG00000235652 | AL356599.1 | turquoise |
| ENSG00000235665 | LINC00298 | turquoise |
| ENSG00000235674 | LDHAP2 | turquoise |
| ENSG00000235706 | DICER1-AS1 | turquoise |
| ENSG00000235720 | GABPAP | turquoise |
| ENSG00000235768 | BRD7P5 | turquoise |

| | | |
|---|---|---|
| ENSG00000235782 | AL031429.1 | turquoise |
| ENSG00000235802 | HCFC1-AS1 | turquoise |
| ENSG00000235859 | AC006978.1 | turquoise |
| ENSG00000235872 | AC078777.1 | turquoise |
| ENSG00000235888 | AF064858.1 | turquoise |
| ENSG00000235897 | TM4SF19-AS1 | turquoise |
| ENSG00000235912 | AL031729.1 | turquoise |
| ENSG00000235958 | UBOX5-AS1 | turquoise |
| ENSG00000235978 | AC018816.1 | turquoise |
| ENSG00000236008 | LINC01814 | turquoise |
| ENSG00000236017 | ASMTL-AS1 | turquoise |
| ENSG00000236056 | GAPDHP14 | turquoise |
| ENSG00000236088 | COX10-AS1 | turquoise |
| ENSG00000236114 | AL450326.2 | turquoise |
| ENSG00000236144 | TMEM147-AS1 | turquoise |
| ENSG00000236152 | MRPS36P1 | turquoise |
| ENSG00000236213 | AC006369.1 | turquoise |
| ENSG00000236242 | MYO16-AS1 | turquoise |
| ENSG00000236283 | AC019197.1 | turquoise |
| ENSG00000236287 | ZBED5 | turquoise |
| ENSG00000236296 | GUSBP5 | turquoise |
| ENSG00000236297 | AC048351.1 | turquoise |
| ENSG00000236304 | AP001189.1 | turquoise |
| ENSG00000236305 | SLC12A9-AS1 | turquoise |
| ENSG00000236308 | AL138921.2 | turquoise |
| ENSG00000236345 | AL354719.2 | turquoise |
| ENSG00000236349 | SUCLG2P2 | turquoise |
| ENSG00000236397 | DDX11L2 | turquoise |
| ENSG00000236403 | AL353611.1 | turquoise |
| ENSG00000236409 | NRADDP | turquoise |
| ENSG00000236423 | LINC01134 | turquoise |
| ENSG00000236432 | AC097662.1 | turquoise |
| ENSG00000236439 | AC099336.2 | turquoise |
| ENSG00000236499 | LINC00896 | turquoise |
| ENSG00000236519 | LINC01424 | turquoise |
| ENSG00000236609 | ZNF853 | turquoise |
| ENSG00000236682 | AC068282.1 | turquoise |
| ENSG00000236698 | EIF1AXP1 | turquoise |
| ENSG00000236762 | RPL19P16 | turquoise |
| ENSG00000236792 | AL513175.2 | turquoise |
| ENSG00000236810 | ELOA-AS1 | turquoise |
| ENSG00000236814 | AC046176.1 | turquoise |
| ENSG00000236829 | Z97634.1 | turquoise |
| ENSG00000236859 | NIFK-AS1 | turquoise |
| ENSG00000236882 | LINC01554 | turquoise |
| ENSG00000236935 | AP003774.4 | turquoise |
| ENSG00000236936 | AL031005.1 | turquoise |
| ENSG00000236946 | HNRNPA1P70 | turquoise |

| | | |
|---|---|---|
| ENSG00000237015 | AL031186.1 | turquoise |
| ENSG00000237039 | AC018738.1 | turquoise |
| ENSG00000237101 | AC092809.4 | turquoise |
| ENSG00000237111 | IGHJ3P | turquoise |
| ENSG00000237126 | AC073254.1 | turquoise |
| ENSG00000237149 | ZNF503-AS2 | turquoise |
| ENSG00000237152 | DLEU7-AS1 | turquoise |
| ENSG00000237169 | RPL12P27 | turquoise |
| ENSG00000237254 | TRBV30 | turquoise |
| ENSG00000237264 | FTH1P11 | turquoise |
| ENSG00000237276 | ANO7L1 | turquoise |
| ENSG00000237310 | GS1-124K5.4 | turquoise |
| ENSG00000237350 | CDC42P6 | turquoise |
| ENSG00000237357 | BX088651.4 | turquoise |
| ENSG00000237372 | UNQ6494 | turquoise |
| ENSG00000237399 | PITRM1-AS1 | turquoise |
| ENSG00000237415 | NRBF2P3 | turquoise |
| ENSG00000237424 | FOXD2-AS1 | turquoise |
| ENSG00000237429 | BX293535.1 | turquoise |
| ENSG00000237438 | CECR7 | turquoise |
| ENSG00000237440 | ZNF737 | turquoise |
| ENSG00000237471 | AC073115.2 | turquoise |
| ENSG00000237476 | LINC01637 | turquoise |
| ENSG00000237499 | AL357060.2 | turquoise |
| ENSG00000237506 | RPSAP15 | turquoise |
| ENSG00000237513 | AC007384.1 | turquoise |
| ENSG00000237520 | AL391832.1 | turquoise |
| ENSG00000237550 | RPL9P9 | turquoise |
| ENSG00000237575 | PYY2 | turquoise |
| ENSG00000237638 | LINC02245 | turquoise |
| ENSG00000237749 | AL034379.1 | turquoise |
| ENSG00000237757 | EEF1A1P30 | turquoise |
| ENSG00000237758 | BANF1P3 | turquoise |
| ENSG00000237772 | AC092620.1 | turquoise |
| ENSG00000237773 | AC003075.1 | turquoise |
| ENSG00000237781 | AL356356.1 | turquoise |
| ENSG00000237803 | LINC00211 | turquoise |
| ENSG00000237821 | AC083873.1 | turquoise |
| ENSG00000237836 | PHKA2-AS1 | turquoise |
| ENSG00000237840 | FAM21FP | turquoise |
| ENSG00000237842 | AL157713.1 | turquoise |
| ENSG00000237886 | NALT1 | turquoise |
| ENSG00000237927 | AL078604.2 | turquoise |
| ENSG00000237950 | AL357079.1 | turquoise |
| ENSG00000237984 | PTENP1 | turquoise |
| ENSG00000238005 | AL391832.2 | turquoise |
| ENSG00000238041 | AP004245.1 | turquoise |
| ENSG00000238045 | AC009133.1 | turquoise |

| | | |
|---|---|---|
| ENSG00000238103 | RPL9P7 | turquoise |
| ENSG00000238105 | GOLGA2P5 | turquoise |
| ENSG00000238110 | AL353572.2 | turquoise |
| ENSG00000238121 | LINC00426 | turquoise |
| ENSG00000238186 | AL603839.2 | turquoise |
| ENSG00000238222 | MKRN4P | turquoise |
| ENSG00000238241 | CCR12P | turquoise |
| ENSG00000238246 | AC069549.1 | turquoise |
| ENSG00000238251 | AL133477.1 | turquoise |
| ENSG00000238260 | AL513320.1 | turquoise |
| ENSG00000238269 | PAGE2B | turquoise |
| ENSG00000238279 | BX470102.1 | turquoise |
| ENSG00000239213 | NCK1-AS1 | turquoise |
| ENSG00000239282 | CASTOR1 | turquoise |
| ENSG00000239305 | RNF103 | turquoise |
| ENSG00000239306 | RBM14 | turquoise |
| ENSG00000239335 | LLPH-AS1 | turquoise |
| ENSG00000239382 | ALKBH6 | turquoise |
| ENSG00000239415 | AP001469.3 | turquoise |
| ENSG00000239470 | AC011979.1 | turquoise |
| ENSG00000239523 | MYLK-AS1 | turquoise |
| ENSG00000239556 | AC004951.2 | turquoise |
| ENSG00000239559 | AP003385.3 | turquoise |
| ENSG00000239569 | KMT2E-AS1 | turquoise |
| ENSG00000239779 | WBP1 | turquoise |
| ENSG00000239797 | RPL21P39 | turquoise |
| ENSG00000239887 | C1orf226 | turquoise |
| ENSG00000239911 | PRKAG2-AS1 | turquoise |
| ENSG00000240024 | LINC00888 | turquoise |
| ENSG00000240038 | AMY2B | turquoise |
| ENSG00000240048 | DDX50P2 | turquoise |
| ENSG00000240050 | AL590542.1 | turquoise |
| ENSG00000240219 | AL512306.2 | turquoise |
| ENSG00000240230 | COX19 | turquoise |
| ENSG00000240344 | PPIL3 | turquoise |
| ENSG00000240356 | RPL23AP7 | turquoise |
| ENSG00000240370 | RPL13P5 | turquoise |
| ENSG00000240399 | AC004801.2 | turquoise |
| ENSG00000240401 | AC012358.3 | turquoise |
| ENSG00000240445 | FOXO3B | turquoise |
| ENSG00000240489 | SETP14 | turquoise |
| ENSG00000240505 | TNFRSF13B | turquoise |
| ENSG00000240509 | RPL34P18 | turquoise |
| ENSG00000240695 | AC117382.1 | turquoise |
| ENSG00000240823 | RN7SL23P | turquoise |
| ENSG00000240854 | AC117409.1 | turquoise |
| ENSG00000240857 | RDH14 | turquoise |
| ENSG00000240889 | NDUFB2-AS1 | turquoise |

| | | |
|---|---|---|
| ENSG00000240891 | PLCXD2 | turquoise |
| ENSG00000240898 | AC132942.1 | turquoise |
| ENSG00000240914 | AL121612.1 | turquoise |
| ENSG00000240954 | RPL4P1 | turquoise |
| ENSG00000241007 | SEPT7P6 | turquoise |
| ENSG00000241057 | AC004951.3 | turquoise |
| ENSG00000241058 | NSUN6 | turquoise |
| ENSG00000241127 | YAE1D1 | turquoise |
| ENSG00000241163 | LINC00877 | turquoise |
| ENSG00000241244 | IGKV1D-16 | turquoise |
| ENSG00000241506 | PSMC1P1 | turquoise |
| ENSG00000241553 | ARPC4 | turquoise |
| ENSG00000241556 | AC018475.1 | turquoise |
| ENSG00000241627 | UBQLN4P1 | turquoise |
| ENSG00000241678 | AC091564.1 | turquoise |
| ENSG00000241721 | SUMO1P1 | turquoise |
| ENSG00000241764 | AC002467.1 | turquoise |
| ENSG00000241837 | ATP5O | turquoise |
| ENSG00000241852 | C8orf58 | turquoise |
| ENSG00000241889 | AC079944.2 | turquoise |
| ENSG00000241945 | PWP2 | turquoise |
| ENSG00000241962 | AC079447.1 | turquoise |
| ENSG00000241973 | PI4KA | turquoise |
| ENSG00000241990 | PRR34-AS1 | turquoise |
| ENSG00000242048 | AC093583.1 | turquoise |
| ENSG00000242083 | AC068620.1 | turquoise |
| ENSG00000242193 | AL359075.1 | turquoise |
| ENSG00000242247 | ARFGAP3 | turquoise |
| ENSG00000242259 | C22orf39 | turquoise |
| ENSG00000242265 | PEG10 | turquoise |
| ENSG00000242338 | BMS1P4 | turquoise |
| ENSG00000242349 | NPPA-AS1 | turquoise |
| ENSG00000242445 | RPL7AP11 | turquoise |
| ENSG00000242550 | SERPINB10 | turquoise |
| ENSG00000242551 | POU5F1P6 | turquoise |
| ENSG00000242571 | RPL21P11 | turquoise |
| ENSG00000242615 | AC022415.1 | turquoise |
| ENSG00000242687 | AC004893.2 | turquoise |
| ENSG00000242732 | RTL5 | turquoise |
| ENSG00000242779 | ZNF702P | turquoise |
| ENSG00000242802 | AP5Z1 | turquoise |
| ENSG00000242951 | AC007182.2 | turquoise |
| ENSG00000242960 | FTH1P23 | turquoise |
| ENSG00000243056 | EIF4EBP3 | turquoise |
| ENSG00000243071 | AC107032.1 | turquoise |
| ENSG00000243199 | AC115223.1 | turquoise |
| ENSG00000243264 | IGKV2D-29 | turquoise |
| ENSG00000243273 | AC020636.1 | turquoise |

| | | |
|---|---|---|
| ENSG00000243279 | PRAF2 | turquoise |
| ENSG00000243364 | EFNA4 | turquoise |
| ENSG00000243389 | AC012442.2 | turquoise |
| ENSG00000243403 | AC090543.2 | turquoise |
| ENSG00000243477 | NAT6 | turquoise |
| ENSG00000243491 | AC082651.3 | turquoise |
| ENSG00000243517 | AC024940.2 | turquoise |
| ENSG00000243560 | RN7SL364P | turquoise |
| ENSG00000243566 | UPK3B | turquoise |
| ENSG00000243659 | FO393419.3 | turquoise |
| ENSG00000243660 | ZNF487 | turquoise |
| ENSG00000243667 | WDR92 | turquoise |
| ENSG00000243679 | AC018638.5 | turquoise |
| ENSG00000243696 | AC006254.1 | turquoise |
| ENSG00000243701 | DUBR | turquoise |
| ENSG00000243716 | NPIPB5 | turquoise |
| ENSG00000243806 | RPL7P18 | turquoise |
| ENSG00000243819 | RN7SL832P | turquoise |
| ENSG00000243824 | RPL12P6 | turquoise |
| ENSG00000243829 | AC011495.1 | turquoise |
| ENSG00000244005 | NFS1 | turquoise |
| ENSG00000244026 | FAM86DP | turquoise |
| ENSG00000244045 | TMEM199 | turquoise |
| ENSG00000244119 | PDCL3P4 | turquoise |
| ENSG00000244151 | AC010973.2 | turquoise |
| ENSG00000244153 | WWP1P1 | turquoise |
| ENSG00000244165 | P2RY11 | turquoise |
| ENSG00000244187 | TMEM141 | turquoise |
| ENSG00000244219 | TMEM225B | turquoise |
| ENSG00000244274 | DBNDD2 | turquoise |
| ENSG00000244363 | RPL7P23 | turquoise |
| ENSG00000244398 | AC116533.1 | turquoise |
| ENSG00000244462 | RBM12 | turquoise |
| ENSG00000244479 | OR2A1-AS1 | turquoise |
| ENSG00000244486 | SCARF2 | turquoise |
| ENSG00000244556 | ODCP | turquoise |
| ENSG00000244607 | CCDC13 | turquoise |
| ENSG00000244625 | MIATNB | turquoise |
| ENSG00000244668 | SNRPCP3 | turquoise |
| ENSG00000244716 | BX679664.3 | turquoise |
| ENSG00000244733 | AL132656.2 | turquoise |
| ENSG00000244926 | ALKBH3-AS1 | turquoise |
| ENSG00000245008 | AP001122.1 | turquoise |
| ENSG00000245017 | LINC02453 | turquoise |
| ENSG00000245025 | AC107959.1 | turquoise |
| ENSG00000245156 | AP001107.1 | turquoise |
| ENSG00000245164 | LINC00861 | turquoise |
| ENSG00000245213 | AC105285.1 | turquoise |

| | | |
|---|---|---|
| ENSG00000245532 | NEAT1 | turquoise |
| ENSG00000245534 | RORA-AS1 | turquoise |
| ENSG00000245552 | AP000787.1 | turquoise |
| ENSG00000245573 | BDNF-AS | turquoise |
| ENSG00000245680 | ZNF585B | turquoise |
| ENSG00000245812 | LINC02202 | turquoise |
| ENSG00000245849 | RAD51-AS1 | turquoise |
| ENSG00000245888 | FLJ21408 | turquoise |
| ENSG00000245910 | SNHG6 | turquoise |
| ENSG00000245954 | LINC02273 | turquoise |
| ENSG00000245970 | AP003352.1 | turquoise |
| ENSG00000246084 | LINC02325 | turquoise |
| ENSG00000246089 | AC016065.1 | turquoise |
| ENSG00000246100 | LINC00900 | turquoise |
| ENSG00000246174 | KCTD21-AS1 | turquoise |
| ENSG00000246263 | UBR5-AS1 | turquoise |
| ENSG00000246366 | LACTB2-AS1 | turquoise |
| ENSG00000246430 | LINC00968 | turquoise |
| ENSG00000246548 | LINC02288 | turquoise |
| ENSG00000246705 | H2AFJ | turquoise |
| ENSG00000246859 | STARD4-AS1 | turquoise |
| ENSG00000246898 | LINC00920 | turquoise |
| ENSG00000246985 | SOCS2-AS1 | turquoise |
| ENSG00000247121 | AC009126.1 | turquoise |
| ENSG00000247134 | AC090204.1 | turquoise |
| ENSG00000247157 | LINC01252 | turquoise |
| ENSG00000247315 | ZCCHC3 | turquoise |
| ENSG00000247317 | AC105202.1 | turquoise |
| ENSG00000247363 | AC090061.1 | turquoise |
| ENSG00000247373 | AC055713.1 | turquoise |
| ENSG00000247400 | DNAJC3-AS1 | turquoise |
| ENSG00000247516 | MIR4458HG | turquoise |
| ENSG00000247556 | OIP5-AS1 | turquoise |
| ENSG00000247572 | CKMT2-AS1 | turquoise |
| ENSG00000247596 | TWF2 | turquoise |
| ENSG00000247626 | MARS2 | turquoise |
| ENSG00000247627 | MTND4P12 | turquoise |
| ENSG00000247679 | AC139795.2 | turquoise |
| ENSG00000247708 | STX18-AS1 | turquoise |
| ENSG00000247796 | AC008966.1 | turquoise |
| ENSG00000247903 | AC024896.1 | turquoise |
| ENSG00000247934 | AC022364.1 | turquoise |
| ENSG00000247950 | SEC24B-AS1 | turquoise |
| ENSG00000248008 | NRAV | turquoise |
| ENSG00000248019 | FAM13A-AS1 | turquoise |
| ENSG00000248092 | NNT-AS1 | turquoise |
| ENSG00000248256 | OCIAD1-AS1 | turquoise |
| ENSG00000248265 | FLJ12825 | turquoise |

| | | |
|---|---|---|
| ENSG00000248275 | TRIM52-AS1 | turquoise |
| ENSG00000248309 | MEF2C-AS1 | turquoise |
| ENSG00000248318 | AC104958.1 | turquoise |
| ENSG00000248333 | CDK11B | turquoise |
| ENSG00000248367 | AC008610.1 | turquoise |
| ENSG00000248508 | SRP14-AS1 | turquoise |
| ENSG00000248514 | AC008443.4 | turquoise |
| ENSG00000248516 | AC105415.1 | turquoise |
| ENSG00000248569 | AC026410.3 | turquoise |
| ENSG00000248592 | TMEM110-MUSTN | turquoise |
| ENSG00000248632 | AC106872.5 | turquoise |
| ENSG00000248641 | HMGA1P2 | turquoise |
| ENSG00000248671 | ALG1L9P | turquoise |
| ENSG00000248712 | CCDC153 | turquoise |
| ENSG00000248810 | LINC02432 | turquoise |
| ENSG00000248858 | FLJ46284 | turquoise |
| ENSG00000248873 | SERBP1P6 | turquoise |
| ENSG00000248923 | MTND5P11 | turquoise |
| ENSG00000248925 | AC021087.1 | turquoise |
| ENSG00000248932 | AC097103.2 | turquoise |
| ENSG00000248936 | AC027607.1 | turquoise |
| ENSG00000248971 | KRT8P46 | turquoise |
| ENSG00000248996 | AC145098.1 | turquoise |
| ENSG00000249109 | AC140125.2 | turquoise |
| ENSG00000249115 | HAUS5 | turquoise |
| ENSG00000249249 | AC010226.1 | turquoise |
| ENSG00000249309 | AC020703.1 | turquoise |
| ENSG00000249412 | AC010285.2 | turquoise |
| ENSG00000249437 | NAIP | turquoise |
| ENSG00000249454 | GZMAP1 | turquoise |
| ENSG00000249459 | ZNF286B | turquoise |
| ENSG00000249471 | ZNF324B | turquoise |
| ENSG00000249485 | RBBP4P1 | turquoise |
| ENSG00000249565 | SERBP1P5 | turquoise |
| ENSG00000249602 | AL589765.4 | turquoise |
| ENSG00000249626 | AC024560.2 | turquoise |
| ENSG00000249646 | OR7E94P | turquoise |
| ENSG00000249662 | LINC02218 | turquoise |
| ENSG00000249673 | NOP14-AS1 | turquoise |
| ENSG00000249763 | AC114786.1 | turquoise |
| ENSG00000249790 | AC092490.1 | turquoise |
| ENSG00000249849 | AC138819.1 | turquoise |
| ENSG00000249884 | RNF103-CHMP3 | turquoise |
| ENSG00000249915 | PDCD6 | turquoise |
| ENSG00000249936 | RAC1P2 | turquoise |
| ENSG00000249993 | BFSP2-AS1 | turquoise |
| ENSG00000250011 | HMGB1P3 | turquoise |
| ENSG00000250067 | YJEFN3 | turquoise |

| | | |
|---|---|---|
| ENSG00000250131 | AC078881.1 | turquoise |
| ENSG00000250132 | AC004803.1 | turquoise |
| ENSG00000250138 | AC139495.3 | turquoise |
| ENSG00000250159 | AC106791.1 | turquoise |
| ENSG00000250220 | AC053527.1 | turquoise |
| ENSG00000250299 | MRPS31P4 | turquoise |
| ENSG00000250317 | SMIM20 | turquoise |
| ENSG00000250318 | AC003072.1 | turquoise |
| ENSG00000250321 | AC079140.2 | turquoise |
| ENSG00000250326 | AC104596.1 | turquoise |
| ENSG00000250334 | LINC00989 | turquoise |
| ENSG00000250397 | AP006623.1 | turquoise |
| ENSG00000250486 | FAM218A | turquoise |
| ENSG00000250492 | INTS6P1 | turquoise |
| ENSG00000250506 | CDK3 | turquoise |
| ENSG00000250510 | GPR162 | turquoise |
| ENSG00000250565 | ATP6V1E2 | turquoise |
| ENSG00000250571 | GLI4 | turquoise |
| ENSG00000250575 | AL732372.3 | turquoise |
| ENSG00000250644 | AC068580.4 | turquoise |
| ENSG00000250651 | PABPC1P7 | turquoise |
| ENSG00000250654 | AC023794.3 | turquoise |
| ENSG00000250673 | AC097372.1 | turquoise |
| ENSG00000250696 | AC111000.4 | turquoise |
| ENSG00000250722 | SELENOP | turquoise |
| ENSG00000250764 | AC025178.1 | turquoise |
| ENSG00000250831 | AC074131.1 | turquoise |
| ENSG00000250850 | AL161781.2 | turquoise |
| ENSG00000250878 | METTL21EP | turquoise |
| ENSG00000250892 | AC108475.1 | turquoise |
| ENSG00000250896 | RNPS1P1 | turquoise |
| ENSG00000250988 | SNHG21 | turquoise |
| ENSG00000251002 | AC244502.1 | turquoise |
| ENSG00000251022 | THAP9-AS1 | turquoise |
| ENSG00000251050 | AC112184.1 | turquoise |
| ENSG00000251102 | CTBP2P4 | turquoise |
| ENSG00000251161 | AC020661.1 | turquoise |
| ENSG00000251179 | TMEM92-AS1 | turquoise |
| ENSG00000251192 | ZNF674 | turquoise |
| ENSG00000251247 | ZNF345 | turquoise |
| ENSG00000251259 | AC004069.1 | turquoise |
| ENSG00000251323 | AP003086.1 | turquoise |
| ENSG00000251369 | ZNF550 | turquoise |
| ENSG00000251405 | AC008676.2 | turquoise |
| ENSG00000251409 | AC008592.4 | turquoise |
| ENSG00000251411 | AC093827.3 | turquoise |
| ENSG00000251429 | AC098679.3 | turquoise |
| ENSG00000251442 | LINC01094 | turquoise |

| | | |
|---|---|---|
| ENSG00000251455 | AC092611.1 | turquoise |
| ENSG00000251485 | AC068134.3 | turquoise |
| ENSG00000251593 | MSNP1 | turquoise |
| ENSG00000251595 | ABCA11P | turquoise |
| ENSG00000251615 | AC104825.2 | turquoise |
| ENSG00000251634 | AC145138.1 | turquoise |
| ENSG00000251637 | AP003716.1 | turquoise |
| ENSG00000251667 | BRCC3P1 | turquoise |
| ENSG00000251669 | FAM86EP | turquoise |
| ENSG00000251867 | AC009812.1 | turquoise |
| ENSG00000252103 | RNU6-917P | turquoise |
| ENSG00000252274 | SCARNA24 | turquoise |
| ENSG00000252412 | Y_RNA | turquoise |
| ENSG00000252577 | SCARNA20 | turquoise |
| ENSG00000252965 | Y_RNA | turquoise |
| ENSG00000253047 | SNORA40 | turquoise |
| ENSG00000253087 | RNU6-1174P | turquoise |
| ENSG00000253234 | IGLV2-5 | turquoise |
| ENSG00000253250 | C8orf88 | turquoise |
| ENSG00000253251 | AC008560.1 | turquoise |
| ENSG00000253269 | AC008680.1 | turquoise |
| ENSG00000253276 | CCDC71L | turquoise |
| ENSG00000253307 | AC011676.1 | turquoise |
| ENSG00000253364 | AL928742.1 | turquoise |
| ENSG00000253394 | LINC00534 | turquoise |
| ENSG00000253519 | AC106801.1 | turquoise |
| ENSG00000253522 | MIR3142HG | turquoise |
| ENSG00000253570 | AC069120.2 | turquoise |
| ENSG00000253616 | AC107959.3 | turquoise |
| ENSG00000253651 | SOD1P3 | turquoise |
| ENSG00000253669 | AP003356.1 | turquoise |
| ENSG00000253676 | TAGLN2P1 | turquoise |
| ENSG00000253683 | AC027309.2 | turquoise |
| ENSG00000253716 | MINCR | turquoise |
| ENSG00000253719 | ATXN7L3B | turquoise |
| ENSG00000253729 | PRKDC | turquoise |
| ENSG00000253738 | OTUD6B-AS1 | turquoise |
| ENSG00000253785 | AC008429.2 | turquoise |
| ENSG00000253797 | UTP14C | turquoise |
| ENSG00000253829 | AC067817.2 | turquoise |
| ENSG00000253882 | AC099548.2 | turquoise |
| ENSG00000253919 | THAP12P7 | turquoise |
| ENSG00000253954 | HMGN1P38 | turquoise |
| ENSG00000254004 | ZNF260 | turquoise |
| ENSG00000254013 | AC044849.1 | turquoise |
| ENSG00000254016 | ALG1L10P | turquoise |
| ENSG00000254017 | IGHEP2 | turquoise |
| ENSG00000254087 | LYN | turquoise |

| | | |
|---|---|---|
| ENSG00000254126 | CD8B2 | turquoise |
| ENSG00000254166 | CASC19 | turquoise |
| ENSG00000254206 | NPIPB11 | turquoise |
| ENSG00000254231 | AC103760.1 | turquoise |
| ENSG00000254286 | AC083923.2 | turquoise |
| ENSG00000254332 | AF201337.1 | turquoise |
| ENSG00000254363 | AC011379.2 | turquoise |
| ENSG00000254409 | AC087521.3 | turquoise |
| ENSG00000254419 | AL139349.1 | turquoise |
| ENSG00000254427 | AC103736.1 | turquoise |
| ENSG00000254502 | AP003097.1 | turquoise |
| ENSG00000254505 | CHMP4A | turquoise |
| ENSG00000254531 | FLJ20021 | turquoise |
| ENSG00000254577 | AC087276.2 | turquoise |
| ENSG00000254593 | OR7E126P | turquoise |
| ENSG00000254635 | WAC-AS1 | turquoise |
| ENSG00000254682 | AP002387.1 | turquoise |
| ENSG00000254685 | FPGT | turquoise |
| ENSG00000254703 | SENCR | turquoise |
| ENSG00000254719 | AC080023.2 | turquoise |
| ENSG00000254756 | AP001107.6 | turquoise |
| ENSG00000254789 | AC073172.1 | turquoise |
| ENSG00000254810 | AP001189.3 | turquoise |
| ENSG00000254837 | AP001372.2 | turquoise |
| ENSG00000254838 | GVINP1 | turquoise |
| ENSG00000254858 | MPV17L2 | turquoise |
| ENSG00000254887 | AC010247.1 | turquoise |
| ENSG00000254893 | AC113404.3 | turquoise |
| ENSG00000254910 | AC136475.2 | turquoise |
| ENSG00000254952 | AP001257.1 | turquoise |
| ENSG00000254965 | C1DP5 | turquoise |
| ENSG00000255045 | AP000866.5 | turquoise |
| ENSG00000255046 | AC069185.1 | turquoise |
| ENSG00000255112 | CHMP1B | turquoise |
| ENSG00000255150 | EID3 | turquoise |
| ENSG00000255182 | AC084125.2 | turquoise |
| ENSG00000255224 | AC109322.1 | turquoise |
| ENSG00000255237 | AC138230.1 | turquoise |
| ENSG00000255240 | AP001636.3 | turquoise |
| ENSG00000255284 | AP006621.3 | turquoise |
| ENSG00000255302 | EID1 | turquoise |
| ENSG00000255320 | AP000759.1 | turquoise |
| ENSG00000255354 | AC022239.2 | turquoise |
| ENSG00000255389 | Z97989.1 | turquoise |
| ENSG00000255458 | AC108471.2 | turquoise |
| ENSG00000255468 | AP001107.9 | turquoise |
| ENSG00000255478 | AP000944.1 | turquoise |
| ENSG00000255503 | AP001767.3 | turquoise |

| | | |
|---|---|---|
| ENSG00000255508 | AP002990.1 | turquoise |
| ENSG00000255517 | AP002748.3 | turquoise |
| ENSG00000255518 | AC022239.3 | turquoise |
| ENSG00000255521 | AL356215.1 | turquoise |
| ENSG00000255529 | POLR2M | turquoise |
| ENSG00000255561 | FDXACB1 | turquoise |
| ENSG00000255569 | TRAV1-1 | turquoise |
| ENSG00000255571 | MIR9-3HG | turquoise |
| ENSG00000255725 | TDGP1 | turquoise |
| ENSG00000255760 | LINC02422 | turquoise |
| ENSG00000255769 | GOLGA2P10 | turquoise |
| ENSG00000255819 | KLRC4-KLRK1 | turquoise |
| ENSG00000255823 | MTRNR2L8 | turquoise |
| ENSG00000255833 | TIFAB | turquoise |
| ENSG00000255968 | AC024145.1 | turquoise |
| ENSG00000256039 | LINC02446 | turquoise |
| ENSG00000256060 | AC003002.1 | turquoise |
| ENSG00000256087 | ZNF432 | turquoise |
| ENSG00000256171 | GCSHP4 | turquoise |
| ENSG00000256185 | AC055720.2 | turquoise |
| ENSG00000256223 | ZNF10 | turquoise |
| ENSG00000256229 | ZNF486 | turquoise |
| ENSG00000256294 | ZNF225 | turquoise |
| ENSG00000256379 | TRAV8-5 | turquoise |
| ENSG00000256393 | AC138123.1 | turquoise |
| ENSG00000256427 | AC010175.1 | turquoise |
| ENSG00000256448 | AP000763.3 | turquoise |
| ENSG00000256525 | POLG2 | turquoise |
| ENSG00000256553 | TRAV1-2 | turquoise |
| ENSG00000256594 | AC010186.2 | turquoise |
| ENSG00000256628 | ZBTB11-AS1 | turquoise |
| ENSG00000256633 | AP005019.1 | turquoise |
| ENSG00000256660 | CLEC12B | turquoise |
| ENSG00000256673 | AC141557.1 | turquoise |
| ENSG00000256683 | ZNF350 | turquoise |
| ENSG00000256690 | AP001160.1 | turquoise |
| ENSG00000256745 | AP002784.2 | turquoise |
| ENSG00000256771 | ZNF253 | turquoise |
| ENSG00000256812 | CAPNS2 | turquoise |
| ENSG00000256826 | ATP5J2P4 | turquoise |
| ENSG00000256862 | AC005842.1 | turquoise |
| ENSG00000257017 | HP | turquoise |
| ENSG00000257027 | AC010186.3 | turquoise |
| ENSG00000257093 | KIAA1147 | turquoise |
| ENSG00000257108 | NHLRC4 | turquoise |
| ENSG00000257135 | AC007249.2 | turquoise |
| ENSG00000257151 | PWAR6 | turquoise |
| ENSG00000257218 | GATC | turquoise |

| | | |
|---|---|---|
| ENSG00000257242 | LINC01619 | turquoise |
| ENSG00000257267 | ZNF271P | turquoise |
| ENSG00000257275 | AL139020.1 | turquoise |
| ENSG00000257285 | AL132780.1 | turquoise |
| ENSG00000257303 | AC073896.2 | turquoise |
| ENSG00000257322 | AC138123.2 | turquoise |
| ENSG00000257345 | LINC02413 | turquoise |
| ENSG00000257365 | FNTB | turquoise |
| ENSG00000257511 | AC084824.1 | turquoise |
| ENSG00000257524 | AL157935.2 | turquoise |
| ENSG00000257543 | AC063948.1 | turquoise |
| ENSG00000257557 | PPP1R12A-AS1 | turquoise |
| ENSG00000257576 | HSPD1P4 | turquoise |
| ENSG00000257595 | LINC02356 | turquoise |
| ENSG00000257613 | LINC01481 | turquoise |
| ENSG00000257621 | PSMA3-AS1 | turquoise |
| ENSG00000257702 | LBX2-AS1 | turquoise |
| ENSG00000257704 | INAFM1 | turquoise |
| ENSG00000257727 | CNPY2 | turquoise |
| ENSG00000257802 | AC089984.2 | turquoise |
| ENSG00000257950 | P2RX5-TAX1BP3 | turquoise |
| ENSG00000258056 | AC009779.2 | turquoise |
| ENSG00000258101 | AC010173.1 | turquoise |
| ENSG00000258130 | AC106782.1 | turquoise |
| ENSG00000258168 | AC025569.1 | turquoise |
| ENSG00000258181 | AC008083.3 | turquoise |
| ENSG00000258289 | CHURC1 | turquoise |
| ENSG00000258301 | VASH1-AS1 | turquoise |
| ENSG00000258311 | AC009779.3 | turquoise |
| ENSG00000258315 | C17orf49 | turquoise |
| ENSG00000258352 | AC119044.1 | turquoise |
| ENSG00000258471 | AL355922.2 | turquoise |
| ENSG00000258472 | AC005726.2 | turquoise |
| ENSG00000258476 | LINC02207 | turquoise |
| ENSG00000258501 | EIF3LP1 | turquoise |
| ENSG00000258602 | LINC01629 | turquoise |
| ENSG00000258634 | AL160006.1 | turquoise |
| ENSG00000258645 | HSPE1P2 | turquoise |
| ENSG00000258674 | AC011448.1 | turquoise |
| ENSG00000258725 | PRC1-AS1 | turquoise |
| ENSG00000258727 | AL135999.1 | turquoise |
| ENSG00000258732 | AC025884.1 | turquoise |
| ENSG00000258733 | LINC02328 | turquoise |
| ENSG00000258738 | AL121603.2 | turquoise |
| ENSG00000258754 | LINC01579 | turquoise |
| ENSG00000258768 | AL356019.2 | turquoise |
| ENSG00000258810 | AL133371.2 | turquoise |
| ENSG00000258811 | AL583810.2 | turquoise |

| | | |
|---|---|---|
| ENSG00000258818 | RNASE4 | turquoise |
| ENSG00000258819 | LINC02289 | turquoise |
| ENSG00000258824 | AL122035.1 | turquoise |
| ENSG00000258839 | MC1R | turquoise |
| ENSG00000258867 | LINC01146 | turquoise |
| ENSG00000258875 | AL135818.1 | turquoise |
| ENSG00000258890 | CEP95 | turquoise |
| ENSG00000258900 | HNRNPCP1 | turquoise |
| ENSG00000258919 | AL049836.1 | turquoise |
| ENSG00000258920 | FOXN3-AS1 | turquoise |
| ENSG00000258922 | AC106028.2 | turquoise |
| ENSG00000258944 | AC004846.2 | turquoise |
| ENSG00000258976 | AC013451.2 | turquoise |
| ENSG00000259004 | LINC02285 | turquoise |
| ENSG00000259015 | AL442663.4 | turquoise |
| ENSG00000259032 | ENSAP2 | turquoise |
| ENSG00000259042 | AC244502.3 | turquoise |
| ENSG00000259051 | HNRNPUP1 | turquoise |
| ENSG00000259065 | AC005520.2 | turquoise |
| ENSG00000259070 | LINC00639 | turquoise |
| ENSG00000259081 | AF111169.4 | turquoise |
| ENSG00000259086 | AL136298.3 | turquoise |
| ENSG00000259092 | TRAV30 | turquoise |
| ENSG00000259097 | AL163932.1 | turquoise |
| ENSG00000259146 | AC005476.2 | turquoise |
| ENSG00000259153 | AC004816.1 | turquoise |
| ENSG00000259158 | ADAM20P1 | turquoise |
| ENSG00000259162 | AL355075.5 | turquoise |
| ENSG00000259225 | LINC02345 | turquoise |
| ENSG00000259291 | ZNF710-AS1 | turquoise |
| ENSG00000259315 | ACTG1P17 | turquoise |
| ENSG00000259330 | INAFM2 | turquoise |
| ENSG00000259343 | TMC3-AS1 | turquoise |
| ENSG00000259376 | AC090907.1 | turquoise |
| ENSG00000259417 | CTXND1 | turquoise |
| ENSG00000259431 | THTPA | turquoise |
| ENSG00000259448 | LINC02352 | turquoise |
| ENSG00000259479 | SORD2P | turquoise |
| ENSG00000259531 | AC022306.1 | turquoise |
| ENSG00000259536 | AC091045.1 | turquoise |
| ENSG00000259618 | AC012170.3 | turquoise |
| ENSG00000259623 | AC125257.1 | turquoise |
| ENSG00000259641 | PCAT29 | turquoise |
| ENSG00000259658 | AC027559.1 | turquoise |
| ENSG00000259674 | AC092868.1 | turquoise |
| ENSG00000259704 | AC124248.1 | turquoise |
| ENSG00000259706 | HSP90B2P | turquoise |
| ENSG00000259755 | AC090907.2 | turquoise |

| | | |
|---|---|---|
| ENSG00000259781 | HMGB1P6 | turquoise |
| ENSG00000259807 | AC009093.1 | turquoise |
| ENSG00000259820 | AC083843.2 | turquoise |
| ENSG00000259834 | AL365361.1 | turquoise |
| ENSG00000259865 | AL390728.6 | turquoise |
| ENSG00000259869 | AL022344.2 | turquoise |
| ENSG00000259877 | AC009113.1 | turquoise |
| ENSG00000259959 | AC107068.1 | turquoise |
| ENSG00000260000 | AL133338.1 | turquoise |
| ENSG00000260005 | AC027601.1 | turquoise |
| ENSG00000260018 | AC040169.1 | turquoise |
| ENSG00000260032 | NORAD | turquoise |
| ENSG00000260038 | AC009090.1 | turquoise |
| ENSG00000260052 | AC023813.3 | turquoise |
| ENSG00000260060 | AC009088.1 | turquoise |
| ENSG00000260063 | AL512408.1 | turquoise |
| ENSG00000260070 | AC006960.3 | turquoise |
| ENSG00000260078 | AC007342.3 | turquoise |
| ENSG00000260082 | AC106886.1 | turquoise |
| ENSG00000260086 | AC007611.1 | turquoise |
| ENSG00000260095 | AC106820.3 | turquoise |
| ENSG00000260103 | AC012435.1 | turquoise |
| ENSG00000260105 | AOC4P | turquoise |
| ENSG00000260136 | AC008915.2 | turquoise |
| ENSG00000260179 | AL162741.1 | turquoise |
| ENSG00000260193 | AL138781.1 | turquoise |
| ENSG00000260219 | AC106782.2 | turquoise |
| ENSG00000260220 | CCDC187 | turquoise |
| ENSG00000260228 | AC009119.1 | turquoise |
| ENSG00000260244 | AC104083.1 | turquoise |
| ENSG00000260252 | AC009087.1 | turquoise |
| ENSG00000260259 | LINC02166 | turquoise |
| ENSG00000260273 | AL359711.2 | turquoise |
| ENSG00000260274 | AC068338.2 | turquoise |
| ENSG00000260276 | AC022167.2 | turquoise |
| ENSG00000260285 | AL133367.1 | turquoise |
| ENSG00000260290 | AC092115.1 | turquoise |
| ENSG00000260296 | AC095057.3 | turquoise |
| ENSG00000260302 | AP005482.1 | turquoise |
| ENSG00000260314 | MRC1 | turquoise |
| ENSG00000260316 | AL008727.1 | turquoise |
| ENSG00000260317 | AC009812.4 | turquoise |
| ENSG00000260329 | AC007541.1 | turquoise |
| ENSG00000260339 | HEXA-AS1 | turquoise |
| ENSG00000260361 | AC106028.3 | turquoise |
| ENSG00000260367 | AC109460.1 | turquoise |
| ENSG00000260368 | AC027373.1 | turquoise |
| ENSG00000260388 | LINC00562 | turquoise |

| | | |
|---|---|---|
| ENSG00000260398 | AC068700.1 | turquoise |
| ENSG00000260400 | AL513534.1 | turquoise |
| ENSG00000260401 | AP002761.4 | turquoise |
| ENSG00000260404 | AC110079.1 | turquoise |
| ENSG00000260423 | LINC02367 | turquoise |
| ENSG00000260479 | AC009145.1 | turquoise |
| ENSG00000260495 | AC009148.1 | turquoise |
| ENSG00000260517 | AC009093.2 | turquoise |
| ENSG00000260526 | AC109347.1 | turquoise |
| ENSG00000260563 | AC132872.1 | turquoise |
| ENSG00000260588 | AC027702.1 | turquoise |
| ENSG00000260589 | STAM-AS1 | turquoise |
| ENSG00000260630 | SNAI3-AS1 | turquoise |
| ENSG00000260645 | AL359715.3 | turquoise |
| ENSG00000260660 | AC113208.3 | turquoise |
| ENSG00000260669 | AL096870.2 | turquoise |
| ENSG00000260686 | AC008669.1 | turquoise |
| ENSG00000260698 | AL591848.3 | turquoise |
| ENSG00000260747 | AC022968.1 | turquoise |
| ENSG00000260766 | AL513302.2 | turquoise |
| ENSG00000260773 | AC055855.2 | turquoise |
| ENSG00000260793 | AC003102.1 | turquoise |
| ENSG00000260803 | Z84723.1 | turquoise |
| ENSG00000260804 | LINC01963 | turquoise |
| ENSG00000260805 | AC092803.2 | turquoise |
| ENSG00000260822 | AC004656.1 | turquoise |
| ENSG00000260855 | AL591848.4 | turquoise |
| ENSG00000260891 | AC020978.4 | turquoise |
| ENSG00000260911 | AC135050.3 | turquoise |
| ENSG00000260912 | AL158206.1 | turquoise |
| ENSG00000260916 | CCPG1 | turquoise |
| ENSG00000260920 | AL031985.3 | turquoise |
| ENSG00000260924 | LINC01311 | turquoise |
| ENSG00000260940 | AL109741.3 | turquoise |
| ENSG00000260941 | LINC00622 | turquoise |
| ENSG00000260942 | CAPN10-AS1 | turquoise |
| ENSG00000260948 | AL390195.2 | turquoise |
| ENSG00000260966 | AP001486.2 | turquoise |
| ENSG00000260977 | AC016722.3 | turquoise |
| ENSG00000260992 | DOCK9-AS2 | turquoise |
| ENSG00000261052 | SULT1A3 | turquoise |
| ENSG00000261067 | AC109460.3 | turquoise |
| ENSG00000261098 | AP000766.1 | turquoise |
| ENSG00000261126 | RBFADN | turquoise |
| ENSG00000261159 | AC112484.4 | turquoise |
| ENSG00000261167 | AC107027.3 | turquoise |
| ENSG00000261172 | AC133919.2 | turquoise |
| ENSG00000261183 | SPINT1-AS1 | turquoise |

| | | |
|---|---|---|
| ENSG00000261187 | AC079322.1 | turquoise |
| ENSG00000261188 | Z95115.1 | turquoise |
| ENSG00000261218 | AC099524.1 | turquoise |
| ENSG00000261221 | ZNF865 | turquoise |
| ENSG00000261226 | AC092384.3 | turquoise |
| ENSG00000261236 | BOP1 | turquoise |
| ENSG00000261302 | AC106779.1 | turquoise |
| ENSG00000261338 | AC021016.2 | turquoise |
| ENSG00000261342 | AC006538.1 | turquoise |
| ENSG00000261346 | AC116348.3 | turquoise |
| ENSG00000261349 | AL031432.2 | turquoise |
| ENSG00000261359 | PYCARD-AS1 | turquoise |
| ENSG00000261366 | MANEA-AS1 | turquoise |
| ENSG00000261367 | AC012645.2 | turquoise |
| ENSG00000261386 | AC027682.4 | turquoise |
| ENSG00000261408 | TEN1-CDK3 | turquoise |
| ENSG00000261416 | AC012645.3 | turquoise |
| ENSG00000261423 | TMEM202-AS1 | turquoise |
| ENSG00000261448 | AC109446.3 | turquoise |
| ENSG00000261461 | UBE2MP1 | turquoise |
| ENSG00000261468 | AC096921.2 | turquoise |
| ENSG00000261474 | AC026471.4 | turquoise |
| ENSG00000261512 | AC092368.3 | turquoise |
| ENSG00000261534 | AL596244.1 | turquoise |
| ENSG00000261542 | AC011978.2 | turquoise |
| ENSG00000261560 | AC007216.3 | turquoise |
| ENSG00000261609 | GAN | turquoise |
| ENSG00000261613 | AC093525.6 | turquoise |
| ENSG00000261662 | AL359752.1 | turquoise |
| ENSG00000261663 | AC009065.8 | turquoise |
| ENSG00000261671 | AL158211.1 | turquoise |
| ENSG00000261693 | AC134682.1 | turquoise |
| ENSG00000261754 | AC008555.1 | turquoise |
| ENSG00000261773 | AC244090.2 | turquoise |
| ENSG00000261777 | AC012184.3 | turquoise |
| ENSG00000261798 | AL033527.3 | turquoise |
| ENSG00000261799 | AC007406.5 | turquoise |
| ENSG00000261824 | LINC00662 | turquoise |
| ENSG00000261857 | MIA | turquoise |
| ENSG00000261864 | AC130462.1 | turquoise |
| ENSG00000261879 | AC087500.1 | turquoise |
| ENSG00000261884 | AC040162.1 | turquoise |
| ENSG00000261997 | AC007336.1 | turquoise |
| ENSG00000262001 | DLGAP1-AS2 | turquoise |
| ENSG00000262049 | AC139530.1 | turquoise |
| ENSG00000262089 | AC040977.1 | turquoise |
| ENSG00000262152 | LINC00514 | turquoise |
| ENSG00000262155 | LINC02175 | turquoise |

| | | |
|---|---|---|
| ENSG00000262222 | AC133065.2 | turquoise |
| ENSG00000262333 | HNRNPA1P16 | turquoise |
| ENSG00000262410 | AC024361.1 | turquoise |
| ENSG00000262468 | LINC01569 | turquoise |
| ENSG00000262482 | AC004034.1 | turquoise |
| ENSG00000262580 | AC087741.1 | turquoise |
| ENSG00000262691 | AC040160.1 | turquoise |
| ENSG00000262703 | AC009121.1 | turquoise |
| ENSG00000262714 | AC007342.5 | turquoise |
| ENSG00000262873 | AC127496.5 | turquoise |
| ENSG00000262877 | AC110285.2 | turquoise |
| ENSG00000262879 | AC068152.1 | turquoise |
| ENSG00000262903 | AC027796.4 | turquoise |
| ENSG00000262943 | ALOX12P2 | turquoise |
| ENSG00000262979 | AC124319.1 | turquoise |
| ENSG00000263001 | GTF2I | turquoise |
| ENSG00000263004 | AC007114.1 | turquoise |
| ENSG00000263006 | ROCK1P1 | turquoise |
| ENSG00000263011 | AC108134.4 | turquoise |
| ENSG00000263033 | AC007220.1 | turquoise |
| ENSG00000263050 | AC090617.6 | turquoise |
| ENSG00000263072 | ZNF213-AS1 | turquoise |
| ENSG00000263126 | AC040162.3 | turquoise |
| ENSG00000263155 | MYZAP | turquoise |
| ENSG00000263164 | AC087500.2 | turquoise |
| ENSG00000263266 | RPS7P1 | turquoise |
| ENSG00000263272 | AC004148.2 | turquoise |
| ENSG00000263276 | AC020978.8 | turquoise |
| ENSG00000263368 | AC069366.1 | turquoise |
| ENSG00000263400 | TMEM220-AS1 | turquoise |
| ENSG00000263413 | MIR4538 | turquoise |
| ENSG00000263417 | GTSCR1 | turquoise |
| ENSG00000263465 | SRSF8 | turquoise |
| ENSG00000263528 | IKBKE | turquoise |
| ENSG00000263627 | PPP4R1-AS1 | turquoise |
| ENSG00000263724 | DLGAP1-AS3 | turquoise |
| ENSG00000263753 | LINC00667 | turquoise |
| ENSG00000263812 | LINC00908 | turquoise |
| ENSG00000263956 | NBPF11 | turquoise |
| ENSG00000263961 | C1orf186 | turquoise |
| ENSG00000263982 | AC009716.1 | turquoise |
| ENSG00000264049 | MIR4737 | turquoise |
| ENSG00000264083 | AC005899.1 | turquoise |
| ENSG00000264176 | MAGOH2P | turquoise |
| ENSG00000264204 | AGAP7P | turquoise |
| ENSG00000264235 | AP005329.1 | turquoise |
| ENSG00000264247 | LINC00909 | turquoise |
| ENSG00000264493 | MIR4298 | turquoise |

| | | |
|---|---|---|
| ENSG00000264548 | AC132872.2 | turquoise |
| ENSG00000264575 | LINC00526 | turquoise |
| ENSG00000264644 | KRT18P8 | turquoise |
| ENSG00000264772 | AC016876.2 | turquoise |
| ENSG00000264781 | MIR4537 | turquoise |
| ENSG00000264885 | AC026271.4 | turquoise |
| ENSG00000264920 | AC018521.5 | turquoise |
| ENSG00000264924 | AC090772.1 | turquoise |
| ENSG00000265018 | AGAP12P | turquoise |
| ENSG00000265100 | AC005332.1 | turquoise |
| ENSG00000265206 | AC004687.1 | turquoise |
| ENSG00000265452 | MIR3682 | turquoise |
| ENSG00000265478 | AC107982.3 | turquoise |
| ENSG00000265491 | RNF115 | turquoise |
| ENSG00000265511 | AC020558.2 | turquoise |
| ENSG00000265574 | WDR45BP1 | turquoise |
| ENSG00000265646 | AC069061.4 | turquoise |
| ENSG00000265692 | LINC01970 | turquoise |
| ENSG00000265728 | AP001099.1 | turquoise |
| ENSG00000265743 | AC138207.5 | turquoise |
| ENSG00000265752 | AC010754.1 | turquoise |
| ENSG00000265787 | CYP4F35P | turquoise |
| ENSG00000265808 | SEC22B | turquoise |
| ENSG00000265874 | MIR4489 | turquoise |
| ENSG00000265907 | AP000919.2 | turquoise |
| ENSG00000265917 | MIR3685 | turquoise |
| ENSG00000265936 | AC091042.1 | turquoise |
| ENSG00000265972 | TXNIP | turquoise |
| ENSG00000265975 | AC002091.1 | turquoise |
| ENSG00000265982 | AC103810.3 | turquoise |
| ENSG00000265992 | ESRG | turquoise |
| ENSG00000266053 | NDUFV2-AS1 | turquoise |
| ENSG00000266066 | AC008158.1 | turquoise |
| ENSG00000266094 | RASSF5 | turquoise |
| ENSG00000266208 | AC080112.2 | turquoise |
| ENSG00000266274 | RN7SL138P | turquoise |
| ENSG00000266405 | CBX3P2 | turquoise |
| ENSG00000266412 | NCOA4 | turquoise |
| ENSG00000266469 | AC005288.1 | turquoise |
| ENSG00000266473 | AC007448.3 | turquoise |
| ENSG00000266501 | AC025198.1 | turquoise |
| ENSG00000266651 | AC093484.5 | turquoise |
| ENSG00000266709 | AC005224.4 | turquoise |
| ENSG00000266714 | MYO15B | turquoise |
| ENSG00000266777 | AC090616.6 | turquoise |
| ENSG00000266865 | AC138207.8 | turquoise |
| ENSG00000266904 | LINC00663 | turquoise |
| ENSG00000266910 | AC008507.1 | turquoise |

| | | |
|---|---|---|
| ENSG00000266916 | ZNF793-AS1 | turquoise |
| ENSG00000266931 | AC125232.2 | turquoise |
| ENSG00000266947 | AC022916.1 | turquoise |
| ENSG00000266967 | AARSD1 | turquoise |
| ENSG00000266980 | AC087289.1 | turquoise |
| ENSG00000266998 | AC111182.1 | turquoise |
| ENSG00000267002 | AC060780.1 | turquoise |
| ENSG00000267080 | ASB16-AS1 | turquoise |
| ENSG00000267102 | AC060766.1 | turquoise |
| ENSG00000267106 | ZNF561-AS1 | turquoise |
| ENSG00000267114 | AC011481.1 | turquoise |
| ENSG00000267117 | AC010525.1 | turquoise |
| ENSG00000267119 | RPL10P15 | turquoise |
| ENSG00000267121 | AC008105.3 | turquoise |
| ENSG00000267141 | AC012615.4 | turquoise |
| ENSG00000267152 | AC093227.1 | turquoise |
| ENSG00000267169 | AC022098.1 | turquoise |
| ENSG00000267174 | AC011472.2 | turquoise |
| ENSG00000267185 | AC067852.4 | turquoise |
| ENSG00000267194 | AC002546.1 | turquoise |
| ENSG00000267197 | AC011461.1 | turquoise |
| ENSG00000267216 | AC020915.2 | turquoise |
| ENSG00000267221 | C17orf113 | turquoise |
| ENSG00000267249 | AP005482.3 | turquoise |
| ENSG00000267253 | AC055813.1 | turquoise |
| ENSG00000267272 | LINC01140 | turquoise |
| ENSG00000267277 | AC024575.1 | turquoise |
| ENSG00000267281 | AC023509.3 | turquoise |
| ENSG00000267283 | AC005306.1 | turquoise |
| ENSG00000267288 | AC138150.2 | turquoise |
| ENSG00000267296 | CEBPA-AS1 | turquoise |
| ENSG00000267321 | LINC02001 | turquoise |
| ENSG00000267342 | AC087289.2 | turquoise |
| ENSG00000267355 | AC022966.1 | turquoise |
| ENSG00000267364 | AC022706.1 | turquoise |
| ENSG00000267365 | KCNJ2-AS1 | turquoise |
| ENSG00000267369 | AC015911.7 | turquoise |
| ENSG00000267370 | AC008752.3 | turquoise |
| ENSG00000267414 | AC120049.1 | turquoise |
| ENSG00000267416 | AC025048.4 | turquoise |
| ENSG00000267422 | AC016582.2 | turquoise |
| ENSG00000267454 | ZNF582-AS1 | turquoise |
| ENSG00000267470 | ZNF571-AS1 | turquoise |
| ENSG00000267480 | AP001542.3 | turquoise |
| ENSG00000267496 | FAM215A | turquoise |
| ENSG00000267501 | AC104365.2 | turquoise |
| ENSG00000267508 | ZNF285 | turquoise |
| ENSG00000267510 | AC011451.1 | turquoise |

| | | |
|---|---|---|
| ENSG00000267519 | AC020916.1 | turquoise |
| ENSG00000267533 | AP002414.4 | turquoise |
| ENSG00000267543 | AC015802.3 | turquoise |
| ENSG00000267575 | AC006504.5 | turquoise |
| ENSG00000267648 | AC060766.5 | turquoise |
| ENSG00000267649 | AC010327.4 | turquoise |
| ENSG00000267676 | THA1P | turquoise |
| ENSG00000267680 | ZNF224 | turquoise |
| ENSG00000267681 | AC135721.2 | turquoise |
| ENSG00000267698 | AC002116.2 | turquoise |
| ENSG00000267724 | AC012254.3 | turquoise |
| ENSG00000267731 | AC005332.5 | turquoise |
| ENSG00000267736 | HMGB2P1 | turquoise |
| ENSG00000267737 | AC087645.2 | turquoise |
| ENSG00000267742 | FAM60CP | turquoise |
| ENSG00000267745 | AC060766.7 | turquoise |
| ENSG00000267751 | AC009005.1 | turquoise |
| ENSG00000267767 | LINC01801 | turquoise |
| ENSG00000267769 | AC011498.6 | turquoise |
| ENSG00000267787 | AC027097.2 | turquoise |
| ENSG00000267796 | LIN37 | turquoise |
| ENSG00000267855 | NDUFA7 | turquoise |
| ENSG00000267858 | MZF1-AS1 | turquoise |
| ENSG00000267871 | AC005261.1 | turquoise |
| ENSG00000267898 | AC026803.2 | turquoise |
| ENSG00000267938 | EIF1P6 | turquoise |
| ENSG00000267940 | AC022762.2 | turquoise |
| ENSG00000267980 | AC007292.1 | turquoise |
| ENSG00000268001 | CARD8-AS1 | turquoise |
| ENSG00000268006 | PTOV1-AS1 | turquoise |
| ENSG00000268034 | AC243960.2 | turquoise |
| ENSG00000268041 | AC010616.1 | turquoise |
| ENSG00000268061 | NAPA-AS1 | turquoise |
| ENSG00000268154 | Metazoa_SRP | turquoise |
| ENSG00000268170 | AC073342.2 | turquoise |
| ENSG00000268189 | AC005785.1 | turquoise |
| ENSG00000268199 | AC010335.1 | turquoise |
| ENSG00000268204 | AC008763.1 | turquoise |
| ENSG00000268205 | AC005261.2 | turquoise |
| ENSG00000268230 | AC012313.3 | turquoise |
| ENSG00000268262 | AC011445.1 | turquoise |
| ENSG00000268350 | FAM156A | turquoise |
| ENSG00000268403 | AC132192.2 | turquoise |
| ENSG00000268433 | MTDHP3 | turquoise |
| ENSG00000268471 | LINC02486 | turquoise |
| ENSG00000268520 | AC008750.5 | turquoise |
| ENSG00000268543 | AC012313.4 | turquoise |
| ENSG00000268581 | SIGLEC18P | turquoise |

| | | |
|---|---|---|
| ENSG00000268615 | AL353803.5 | turquoise |
| ENSG00000268649 | AL132655.2 | turquoise |
| ENSG00000268758 | ADGRE4P | turquoise |
| ENSG00000268804 | LINC02132 | turquoise |
| ENSG00000268858 | AL118506.1 | turquoise |
| ENSG00000268895 | A1BG-AS1 | turquoise |
| ENSG00000268912 | AC012313.5 | turquoise |
| ENSG00000268938 | AC005387.1 | turquoise |
| ENSG00000268996 | MAN1B1-AS1 | turquoise |
| ENSG00000269019 | AC002985.2 | turquoise |
| ENSG00000269054 | AC012313.6 | turquoise |
| ENSG00000269072 | AC063977.6 | turquoise |
| ENSG00000269148 | AC092301.1 | turquoise |
| ENSG00000269153 | LYPLA2P2 | turquoise |
| ENSG00000269161 | AC010618.2 | turquoise |
| ENSG00000269191 | AC005387.2 | turquoise |
| ENSG00000269194 | AC006942.1 | turquoise |
| ENSG00000269220 | LINC00528 | turquoise |
| ENSG00000269313 | MAGIX | turquoise |
| ENSG00000269352 | PTOV1-AS2 | turquoise |
| ENSG00000269378 | AC022149.1 | turquoise |
| ENSG00000269386 | RAB11B-AS1 | turquoise |
| ENSG00000269388 | AC018755.3 | turquoise |
| ENSG00000269399 | AC008764.6 | turquoise |
| ENSG00000269404 | SPIB | turquoise |
| ENSG00000269439 | AC010618.3 | turquoise |
| ENSG00000269463 | AP001160.4 | turquoise |
| ENSG00000269481 | AC010319.4 | turquoise |
| ENSG00000269502 | DMRTC1 | turquoise |
| ENSG00000269534 | AC011466.3 | turquoise |
| ENSG00000269556 | TMEM185A | turquoise |
| ENSG00000269560 | AC010422.4 | turquoise |
| ENSG00000269609 | RPARP-AS1 | turquoise |
| ENSG00000269646 | AC010487.2 | turquoise |
| ENSG00000269681 | AC063977.7 | turquoise |
| ENSG00000269696 | AC005498.3 | turquoise |
| ENSG00000269834 | ZNF528-AS1 | turquoise |
| ENSG00000269837 | IPO5P1 | turquoise |
| ENSG00000269858 | EGLN2 | turquoise |
| ENSG00000269867 | AC010326.3 | turquoise |
| ENSG00000269877 | AC008753.3 | turquoise |
| ENSG00000269890 | AL353593.1 | turquoise |
| ENSG00000269892 | AC125494.2 | turquoise |
| ENSG00000269893 | SNHG8 | turquoise |
| ENSG00000269896 | AL513477.1 | turquoise |
| ENSG00000269910 | AL049840.2 | turquoise |
| ENSG00000269924 | AC024451.4 | turquoise |
| ENSG00000269946 | AL158152.2 | turquoise |

| | | |
|---|---|---|
| ENSG00000269958 | AL049840.4 | turquoise |
| ENSG00000269961 | AC010359.1 | turquoise |
| ENSG00000269970 | AL162424.1 | turquoise |
| ENSG00000269976 | AC012065.3 | turquoise |
| ENSG00000269982 | AC018809.2 | turquoise |
| ENSG00000270012 | AC232271.1 | turquoise |
| ENSG00000270039 | AC025165.5 | turquoise |
| ENSG00000270055 | AC127502.2 | turquoise |
| ENSG00000270062 | AL606834.3 | turquoise |
| ENSG00000270069 | MIR222HG | turquoise |
| ENSG00000270076 | AF131215.7 | turquoise |
| ENSG00000270084 | GAS5-AS1 | turquoise |
| ENSG00000270115 | AL513327.3 | turquoise |
| ENSG00000270124 | AC092127.2 | turquoise |
| ENSG00000270127 | AC027020.2 | turquoise |
| ENSG00000270164 | LINC01480 | turquoise |
| ENSG00000270170 | NCBP2-AS2 | turquoise |
| ENSG00000270179 | AP002840.2 | turquoise |
| ENSG00000270195 | AC016773.1 | turquoise |
| ENSG00000270210 | AC104695.2 | turquoise |
| ENSG00000270228 | AC079880.1 | turquoise |
| ENSG00000270276 | HIST2H4B | turquoise |
| ENSG00000270277 | AC009948.3 | turquoise |
| ENSG00000270325 | BNIP3P9 | turquoise |
| ENSG00000270441 | AC135506.1 | turquoise |
| ENSG00000270557 | AC013731.1 | turquoise |
| ENSG00000270562 | AC097634.1 | turquoise |
| ENSG00000270587 | AC046185.2 | turquoise |
| ENSG00000270629 | NBPF14 | turquoise |
| ENSG00000270760 | AD001527.1 | turquoise |
| ENSG00000270804 | AC010326.4 | turquoise |
| ENSG00000270890 | AL049844.2 | turquoise |
| ENSG00000270933 | AC010719.1 | turquoise |
| ENSG00000270983 | AL589674.2 | turquoise |
| ENSG00000271020 | AC112220.2 | turquoise |
| ENSG00000271092 | TMEM56-RWDD3 | turquoise |
| ENSG00000271109 | AC008555.5 | turquoise |
| ENSG00000271122 | AC018647.2 | turquoise |
| ENSG00000271147 | ARMCX5-GPRASP2 | turquoise |
| ENSG00000271151 | AC016737.1 | turquoise |
| ENSG00000271228 | AL121655.1 | turquoise |
| ENSG00000271344 | AC018638.6 | turquoise |
| ENSG00000271383 | NBPF19 | turquoise |
| ENSG00000271424 | AL157791.2 | turquoise |
| ENSG00000271427 | AL358072.1 | turquoise |
| ENSG00000271533 | Z83843.1 | turquoise |
| ENSG00000271554 | AL138787.2 | turquoise |
| ENSG00000271601 | LIX1L | turquoise |

| | | |
|---|---|---|
| ENSG00000271607 | Z84480.1 | turquoise |
| ENSG00000271659 | AL161729.4 | turquoise |
| ENSG00000271730 | AL390208.1 | turquoise |
| ENSG00000271743 | AF287957.1 | turquoise |
| ENSG00000271788 | AC008875.1 | turquoise |
| ENSG00000271795 | AC011337.1 | turquoise |
| ENSG00000271806 | AL590822.2 | turquoise |
| ENSG00000271815 | AC008897.3 | turquoise |
| ENSG00000271840 | AL031281.2 | turquoise |
| ENSG00000271849 | AC012603.1 | turquoise |
| ENSG00000271856 | LINC01215 | turquoise |
| ENSG00000271857 | AL096865.1 | turquoise |
| ENSG00000271858 | CYB561D2 | turquoise |
| ENSG00000271870 | AC024060.1 | turquoise |
| ENSG00000271871 | AC005740.4 | turquoise |
| ENSG00000271889 | AC016747.2 | turquoise |
| ENSG00000271895 | AL109811.3 | turquoise |
| ENSG00000271938 | AC103724.4 | turquoise |
| ENSG00000271941 | AC104184.1 | turquoise |
| ENSG00000271967 | AL583856.2 | turquoise |
| ENSG00000271971 | AC120053.1 | turquoise |
| ENSG00000271976 | AC012467.2 | turquoise |
| ENSG00000272009 | AL121944.1 | turquoise |
| ENSG00000272010 | AC100814.1 | turquoise |
| ENSG00000272031 | ANKRD34A | turquoise |
| ENSG00000272047 | GTF2H5 | turquoise |
| ENSG00000272056 | AC013472.3 | turquoise |
| ENSG00000272078 | AL139423.1 | turquoise |
| ENSG00000272084 | AL137127.1 | turquoise |
| ENSG00000272086 | AC025181.2 | turquoise |
| ENSG00000272100 | AL513218.1 | turquoise |
| ENSG00000272102 | AL133406.3 | turquoise |
| ENSG00000272106 | AL691432.2 | turquoise |
| ENSG00000272129 | AL359715.4 | turquoise |
| ENSG00000272143 | FGF14-AS2 | turquoise |
| ENSG00000272144 | AC025171.4 | turquoise |
| ENSG00000272148 | AC013403.2 | turquoise |
| ENSG00000272150 | NBPF25P | turquoise |
| ENSG00000272155 | AC055822.1 | turquoise |
| ENSG00000272156 | AC008280.3 | turquoise |
| ENSG00000272158 | AL139022.2 | turquoise |
| ENSG00000272163 | AF106564.1 | turquoise |
| ENSG00000272168 | CASC15 | turquoise |
| ENSG00000272172 | AC138696.2 | turquoise |
| ENSG00000272181 | AC012557.2 | turquoise |
| ENSG00000272182 | AC135507.1 | turquoise |
| ENSG00000272196 | HIST2H2AA4 | turquoise |
| ENSG00000272205 | AL451050.2 | turquoise |

| | | |
|---|---|---|
| ENSG00000272223 | AL136304.1 | turquoise |
| ENSG00000272275 | AC092687.3 | turquoise |
| ENSG00000272279 | AL512329.2 | turquoise |
| ENSG00000272288 | AL451165.2 | turquoise |
| ENSG00000272301 | AP002360.4 | turquoise |
| ENSG00000272316 | AL021368.2 | turquoise |
| ENSG00000272323 | AC026801.3 | turquoise |
| ENSG00000272335 | AC093297.3 | turquoise |
| ENSG00000272338 | AC067838.1 | turquoise |
| ENSG00000272343 | AC107952.2 | turquoise |
| ENSG00000272345 | AL031775.1 | turquoise |
| ENSG00000272356 | AL080317.3 | turquoise |
| ENSG00000272361 | AC005014.2 | turquoise |
| ENSG00000272369 | AC008035.1 | turquoise |
| ENSG00000272374 | Z97832.2 | turquoise |
| ENSG00000272391 | POM121C | turquoise |
| ENSG00000272398 | CD24 | turquoise |
| ENSG00000272416 | AC025175.1 | turquoise |
| ENSG00000272449 | AL139246.5 | turquoise |
| ENSG00000272455 | AL391244.3 | turquoise |
| ENSG00000272463 | AL357054.4 | turquoise |
| ENSG00000272502 | AC104958.2 | turquoise |
| ENSG00000272506 | AL357078.3 | turquoise |
| ENSG00000272518 | AC036214.2 | turquoise |
| ENSG00000272529 | AC090948.3 | turquoise |
| ENSG00000272541 | AL021368.3 | turquoise |
| ENSG00000272556 | GTF2IP13 | turquoise |
| ENSG00000272567 | AC109347.2 | turquoise |
| ENSG00000272579 | AL136370.1 | turquoise |
| ENSG00000272583 | AL592494.3 | turquoise |
| ENSG00000272602 | ZNF595 | turquoise |
| ENSG00000272617 | COG8 | turquoise |
| ENSG00000272625 | AP000919.4 | turquoise |
| ENSG00000272632 | AC097504.2 | turquoise |
| ENSG00000272645 | GTF2IP20 | turquoise |
| ENSG00000272654 | AL358472.2 | turquoise |
| ENSG00000272655 | POLR2J4 | turquoise |
| ENSG00000272667 | AC012306.2 | turquoise |
| ENSG00000272668 | AL590560.2 | turquoise |
| ENSG00000272681 | FAM223B | turquoise |
| ENSG00000272686 | AC006333.2 | turquoise |
| ENSG00000272693 | AC073107.1 | turquoise |
| ENSG00000272696 | AL359091.3 | turquoise |
| ENSG00000272707 | AC046143.2 | turquoise |
| ENSG00000272732 | AC004982.1 | turquoise |
| ENSG00000272733 | AP000345.2 | turquoise |
| ENSG00000272734 | ADIRF-AS1 | turquoise |
| ENSG00000272750 | AL592148.3 | turquoise |

| | | |
|---|---|---|
| ENSG00000272752 | STAG3L5P-PVRIG2 | turquoise |
| ENSG00000272760 | AC093726.1 | turquoise |
| ENSG00000272764 | AL596094.1 | turquoise |
| ENSG00000272807 | AC007038.2 | turquoise |
| ENSG00000272812 | AC004908.2 | turquoise |
| ENSG00000272825 | AL844908.1 | turquoise |
| ENSG00000272829 | AC002470.1 | turquoise |
| ENSG00000272836 | AL022328.1 | turquoise |
| ENSG00000272841 | AL139393.2 | turquoise |
| ENSG00000272843 | AC211476.2 | turquoise |
| ENSG00000272849 | AC084018.1 | turquoise |
| ENSG00000272870 | AC097534.2 | turquoise |
| ENSG00000272886 | DCP1A | turquoise |
| ENSG00000272888 | LINC01578 | turquoise |
| ENSG00000272892 | AL133551.1 | turquoise |
| ENSG00000272899 | AC025594.2 | turquoise |
| ENSG00000272902 | TBC1D8-AS1 | turquoise |
| ENSG00000272906 | AL353708.3 | turquoise |
| ENSG00000272911 | AC005696.3 | turquoise |
| ENSG00000272913 | AC009237.14 | turquoise |
| ENSG00000272914 | AL359532.1 | turquoise |
| ENSG00000272933 | AL391121.1 | turquoise |
| ENSG00000272948 | AP001412.1 | turquoise |
| ENSG00000272950 | AC093799.2 | turquoise |
| ENSG00000272953 | AC092171.4 | turquoise |
| ENSG00000272988 | AC022392.1 | turquoise |
| ENSG00000272990 | AC084036.1 | turquoise |
| ENSG00000273000 | AP000347.2 | turquoise |
| ENSG00000273002 | AL355388.2 | turquoise |
| ENSG00000273004 | AL078644.1 | turquoise |
| ENSG00000273007 | AC021205.3 | turquoise |
| ENSG00000273008 | AC010864.1 | turquoise |
| ENSG00000273015 | AC008124.1 | turquoise |
| ENSG00000273018 | AC107983.2 | turquoise |
| ENSG00000273024 | INTS4P2 | turquoise |
| ENSG00000273027 | AL844908.2 | turquoise |
| ENSG00000273038 | AL365203.3 | turquoise |
| ENSG00000273045 | C2orf15 | turquoise |
| ENSG00000273056 | AL354694.1 | turquoise |
| ENSG00000273058 | AL359921.2 | turquoise |
| ENSG00000273061 | CDC37L1-AS1 | turquoise |
| ENSG00000273064 | AC017083.2 | turquoise |
| ENSG00000273066 | AL355987.4 | turquoise |
| ENSG00000273084 | AC092171.5 | turquoise |
| ENSG00000273096 | AL021707.8 | turquoise |
| ENSG00000273108 | AL121929.2 | turquoise |
| ENSG00000273133 | AC116651.1 | turquoise |
| ENSG00000273137 | AL022328.2 | turquoise |

| | | |
|---|---|---|
| ENSG00000273139 | AC007663.3 | turquoise |
| ENSG00000273142 | AC073335.2 | turquoise |
| ENSG00000273145 | BX537318.1 | turquoise |
| ENSG00000273148 | AL035563.1 | turquoise |
| ENSG00000273153 | AC067747.1 | turquoise |
| ENSG00000273156 | AC124016.2 | turquoise |
| ENSG00000273164 | DGCR10 | turquoise |
| ENSG00000273173 | SNURF | turquoise |
| ENSG00000273189 | AC010619.2 | turquoise |
| ENSG00000273203 | AC006946.2 | turquoise |
| ENSG00000273210 | AP001437.1 | turquoise |
| ENSG00000273216 | AC002059.1 | turquoise |
| ENSG00000273218 | AC005776.2 | turquoise |
| ENSG00000273230 | AC102953.2 | turquoise |
| ENSG00000273237 | AC004520.1 | turquoise |
| ENSG00000273253 | AL022328.4 | turquoise |
| ENSG00000273261 | AC092953.2 | turquoise |
| ENSG00000273270 | AC090114.2 | turquoise |
| ENSG00000273271 | AP000254.1 | turquoise |
| ENSG00000273302 | AC016747.3 | turquoise |
| ENSG00000273344 | PAXIP1-AS1 | turquoise |
| ENSG00000273348 | AC027449.1 | turquoise |
| ENSG00000273363 | AL353801.3 | turquoise |
| ENSG00000273366 | Z83851.2 | turquoise |
| ENSG00000273367 | AL355472.4 | turquoise |
| ENSG00000273369 | AC096586.2 | turquoise |
| ENSG00000273373 | AL355488.2 | turquoise |
| ENSG00000273374 | AC069222.1 | turquoise |
| ENSG00000273382 | AL356488.3 | turquoise |
| ENSG00000273402 | AC004908.3 | turquoise |
| ENSG00000273419 | AC004877.1 | turquoise |
| ENSG00000273424 | AL008582.1 | turquoise |
| ENSG00000273437 | AC108673.3 | turquoise |
| ENSG00000273444 | AC006023.2 | turquoise |
| ENSG00000273447 | AC004067.1 | turquoise |
| ENSG00000273449 | AC093788.1 | turquoise |
| ENSG00000273472 | AC096733.2 | turquoise |
| ENSG00000273478 | AC099676.1 | turquoise |
| ENSG00000273513 | TBC1D3K | turquoise |
| ENSG00000273654 | AC020904.2 | turquoise |
| ENSG00000273669 | AC015819.1 | turquoise |
| ENSG00000273679 | AC100757.1 | turquoise |
| ENSG00000273723 | AL139089.1 | turquoise |
| ENSG00000273724 | AC106782.5 | turquoise |
| ENSG00000273748 | AL592183.1 | turquoise |
| ENSG00000273783 | AL136040.2 | turquoise |
| ENSG00000273820 | USP27X | turquoise |
| ENSG00000273824 | AC008033.3 | turquoise |

| | | |
|---|---|---|
| ENSG00000273855 | AC020658.5 | turquoise |
| ENSG00000273899 | FUCA2 | turquoise |
| ENSG00000273972 | LAP3 | turquoise |
| ENSG00000274020 | LASP1 | turquoise |
| ENSG00000274029 | CASP10 | turquoise |
| ENSG00000274034 | CFLAR | turquoise |
| ENSG00000274104 | CD38 | turquoise |
| ENSG00000274114 | HCCS | turquoise |
| ENSG00000274213 | THSD7A | turquoise |
| ENSG00000274220 | CREBBP | turquoise |
| ENSG00000274265 | CDKL5 | turquoise |
| ENSG00000274270 | NADK | turquoise |
| ENSG00000274272 | CYB561 | turquoise |
| ENSG00000274276 | ETV7 | turquoise |
| ENSG00000274294 | IDS | turquoise |
| ENSG00000274307 | ABHD5 | turquoise |
| ENSG00000274315 | PLAUR | turquoise |
| ENSG00000274341 | NUB1 | turquoise |
| ENSG00000274349 | ANGEL1 | turquoise |
| ENSG00000274419 | HEBP1 | turquoise |
| ENSG00000274423 | ACPP | turquoise |
| ENSG00000274471 | PRDM11 | turquoise |
| ENSG00000274501 | SAMD4A | turquoise |
| ENSG00000274515 | SERPINB1 | turquoise |
| ENSG00000274525 | GRAMD1B | turquoise |
| ENSG00000274600 | ERP44 | turquoise |
| ENSG00000274602 | TYMP | turquoise |
| ENSG00000274615 | BTN3A1 | turquoise |
| ENSG00000274641 | SNX1 | turquoise |
| ENSG00000274678 | ARNTL2 | turquoise |
| ENSG00000274712 | GAB2 | turquoise |
| ENSG00000274717 | PIAS1 | turquoise |
| ENSG00000274736 | MSR1 | turquoise |
| ENSG00000274737 | LCP2 | turquoise |
| ENSG00000274750 | CNTLN | turquoise |
| ENSG00000274751 | WWC3 | turquoise |
| ENSG00000274765 | VAMP3 | turquoise |
| ENSG00000274818 | DKK3 | turquoise |
| ENSG00000274897 | HEBP2 | turquoise |
| ENSG00000274902 | KIF1B | turquoise |
| ENSG00000274911 | CUL1 | turquoise |
| ENSG00000274922 | EIF2AK2 | turquoise |
| ENSG00000274929 | TMCC3 | turquoise |
| ENSG00000274943 | YIPF1 | turquoise |
| ENSG00000274979 | PARP12 | turquoise |
| ENSG00000275052 | SLC2A3 | turquoise |
| ENSG00000275063 | OGFR | turquoise |
| ENSG00000275066 | PTPRU | turquoise |

| | | |
|---|---|---|
| ENSG00000275070 | FAR2 | turquoise |
| ENSG00000275074 | ANKS1A | turquoise |
| ENSG00000275160 | ERBB3 | turquoise |
| ENSG00000275180 | KARS | turquoise |
| ENSG00000275216 | SP100 | turquoise |
| ENSG00000275329 | PDK3 | turquoise |
| ENSG00000275344 | HDAC4 | turquoise |
| ENSG00000275367 | IFI35 | turquoise |
| ENSG00000275371 | INPP5A | turquoise |
| ENSG00000275393 | LAPTM4A | turquoise |
| ENSG00000275409 | ATP1B3 | turquoise |
| ENSG00000275418 | GNB5 | turquoise |
| ENSG00000275437 | PHRF1 | turquoise |
| ENSG00000275479 | POLB | turquoise |
| ENSG00000275481 | ST6GALNAC2 | turquoise |
| ENSG00000275484 | TESK2 | turquoise |
| ENSG00000275485 | MAP4K4 | turquoise |
| ENSG00000275549 | ACTN1 | turquoise |
| ENSG00000275557 | ZFYVE26 | turquoise |
| ENSG00000275569 | FCGR2B | turquoise |
| ENSG00000275580 | ALPK1 | turquoise |
| ENSG00000275709 | DHRS9 | turquoise |
| ENSG00000275764 | NOTCH3 | turquoise |
| ENSG00000275765 | ZZEF1 | turquoise |
| ENSG00000275772 | CELSR1 | turquoise |
| ENSG00000275807 | MARK3 | turquoise |
| ENSG00000275854 | FOSL2 | turquoise |
| ENSG00000275882 | RAB7A | turquoise |
| ENSG00000275964 | ARHGAP15 | turquoise |
| ENSG00000276029 | BAZ2A | turquoise |
| ENSG00000276141 | ANKRD13A | turquoise |
| ENSG00000276148 | ACACB | turquoise |
| ENSG00000276180 | DGKD | turquoise |
| ENSG00000276182 | LAMP3 | turquoise |
| ENSG00000276216 | ACER3 | turquoise |
| ENSG00000276234 | NRDC | turquoise |
| ENSG00000276250 | OSBPL6 | turquoise |
| ENSG00000276259 | SP140 | turquoise |
| ENSG00000276289 | MKNK1 | turquoise |
| ENSG00000276291 | CEACAM1 | turquoise |
| ENSG00000276334 | DDX43 | turquoise |
| ENSG00000276471 | PSEN1 | turquoise |
| ENSG00000276533 | IGSF9B | turquoise |
| ENSG00000276550 | PHLPP1 | turquoise |
| ENSG00000276564 | EPB41L3 | turquoise |
| ENSG00000276566 | HAL | turquoise |
| ENSG00000276571 | CD59 | turquoise |
| ENSG00000276600 | IGSF9 | turquoise |

| | | |
|---|---|---|
| ENSG00000276644 | WNT11 | turquoise |
| ENSG00000276698 | NME8 | turquoise |
| ENSG00000276712 | PGS1 | turquoise |
| ENSG00000276718 | UIMC1 | turquoise |
| ENSG00000276728 | CETP | turquoise |
| ENSG00000276744 | GNAO1 | turquoise |
| ENSG00000276754 | CASS4 | turquoise |
| ENSG00000276791 | METTL2A | turquoise |
| ENSG00000276805 | SIGLEC1 | turquoise |
| ENSG00000276842 | OAS1 | turquoise |
| ENSG00000276953 | MAEA | turquoise |
| ENSG00000276957 | ICAM1 | turquoise |
| ENSG00000276997 | CD209 | turquoise |
| ENSG00000277007 | HAUS4 | turquoise |
| ENSG00000277075 | RNF31 | turquoise |
| ENSG00000277147 | VNN3 | turquoise |
| ENSG00000277150 | LRRFIP2 | turquoise |
| ENSG00000277151 | NRP1 | turquoise |
| ENSG00000277203 | GADD45B | turquoise |
| ENSG00000277232 | P2RX6 | turquoise |
| ENSG00000277369 | CARD10 | turquoise |
| ENSG00000277383 | GRK3 | turquoise |
| ENSG00000277435 | RTCB | turquoise |
| ENSG00000277443 | GTPBP1 | turquoise |
| ENSG00000277462 | PACSIN2 | turquoise |
| ENSG00000277463 | MTMR3 | turquoise |
| ENSG00000277476 | APOL4 | turquoise |
| ENSG00000277491 | APOL1 | turquoise |
| ENSG00000277496 | CERK | turquoise |
| ENSG00000277534 | POLE2 | turquoise |
| ENSG00000277566 | PYGL | turquoise |
| ENSG00000277662 | SPTLC2 | turquoise |
| ENSG00000277715 | PCNX1 | turquoise |
| ENSG00000277744 | PSME2 | turquoise |
| ENSG00000277763 | REC8 | turquoise |
| ENSG00000277767 | TM9SF1 | turquoise |
| ENSG00000277855 | GMPR2 | turquoise |
| ENSG00000277856 | RNF24 | turquoise |
| ENSG00000277938 | RASSF2 | turquoise |
| ENSG00000277954 | TLDC2 | turquoise |
| ENSG00000277959 | SAMHD1 | turquoise |
| ENSG00000277998 | APMAP | turquoise |
| ENSG00000277999 | ZNF516 | turquoise |
| ENSG00000278000 | STS | turquoise |
| ENSG00000278002 | TBL1X | turquoise |
| ENSG00000278112 | BMX | turquoise |
| ENSG00000278133 | ELF4 | turquoise |
| ENSG00000278224 | PIM2 | turquoise |

| | | |
|---|---|---|
| ENSG00000278231 | TNFSF13B | turquoise |
| ENSG00000278238 | PARP4 | turquoise |
| ENSG00000278267 | LYRM1 | turquoise |
| ENSG00000278318 | N4BP1 | turquoise |
| ENSG00000278330 | MMP15 | turquoise |
| ENSG00000278376 | MON1B | turquoise |
| ENSG00000278390 | USP10 | turquoise |
| ENSG00000278420 | MEFV | turquoise |
| ENSG00000278434 | EARS2 | turquoise |
| ENSG00000278540 | AQP9 | turquoise |
| ENSG00000278558 | LACTB | turquoise |
| ENSG00000278599 | ERICH1 | turquoise |
| ENSG00000278607 | TUBB4A | turquoise |
| ENSG00000278619 | ARHGEF18 | turquoise |
| ENSG00000278727 | IL4I1 | turquoise |
| ENSG00000278739 | PDCD5 | turquoise |
| ENSG00000278740 | DENND3 | turquoise |
| ENSG00000278743 | NAPA | turquoise |
| ENSG00000278745 | ELL | turquoise |
| ENSG00000278784 | PBX4 | turquoise |
| ENSG00000278817 | ZC3HAV1 | turquoise |
| ENSG00000278869 | TTC26 | turquoise |
| ENSG00000278876 | GRB10 | turquoise |
| ENSG00000278896 | NOD1 | turquoise |
| ENSG00000278922 | BLVRA | turquoise |
| ENSG00000278948 | TRIM14 | turquoise |
| ENSG00000278962 | C5 | turquoise |
| ENSG00000278966 | SUSD1 | turquoise |
| ENSG00000278974 | DDX58 | turquoise |
| ENSG00000278978 | SHB | turquoise |
| ENSG00000279024 | NOL12 | turquoise |
| ENSG00000279066 | AC068722.2 | turquoise |
| ENSG00000279069 | LINC01138 | turquoise |
| ENSG00000279086 | AC069209.1 | turquoise |
| ENSG00000279095 | MIR6813 | turquoise |
| ENSG00000279140 | AC020910.4 | turquoise |
| ENSG00000279141 | AC004706.3 | turquoise |
| ENSG00000279164 | AC015912.3 | turquoise |
| ENSG00000279168 | AC009163.7 | turquoise |
| ENSG00000279198 | AC245297.3 | turquoise |
| ENSG00000279207 | AL137060.1 | turquoise |
| ENSG00000279253 | AC069281.2 | turquoise |
| ENSG00000279255 | CBSL | turquoise |
| ENSG00000279265 | AC015660.4 | turquoise |
| ENSG00000279267 | AC023449.2 | turquoise |
| ENSG00000279314 | AC009318.3 | turquoise |
| ENSG00000279345 | AC005899.6 | turquoise |
| ENSG00000279348 | ZNF658 | turquoise |

| | | |
|---|---|---|
| ENSG00000279355 | TBC1D3D | turquoise |
| ENSG00000279369 | AC242843.1 | turquoise |
| ENSG00000279377 | AC242376.2 | turquoise |
| ENSG00000279396 | AL121845.4 | turquoise |
| ENSG00000279406 | AC105020.5 | turquoise |
| ENSG00000279413 | AC008443.7 | turquoise |
| ENSG00000279430 | RIMBP3B | turquoise |
| ENSG00000279457 | PI4KAP1 | turquoise |
| ENSG00000279483 | AC233968.1 | turquoise |
| ENSG00000279491 | HIST1H2BO | turquoise |
| ENSG00000279518 | AC106886.3 | turquoise |
| ENSG00000279532 | AC005332.7 | turquoise |
| ENSG00000279536 | AL049757.1 | turquoise |
| ENSG00000279537 | CCL23 | turquoise |
| ENSG00000279539 | AC004466.2 | turquoise |
| ENSG00000279549 | HIST1H3E | turquoise |
| ENSG00000279583 | AC120498.9 | turquoise |
| ENSG00000279641 | AC018926.1 | turquoise |
| ENSG00000279673 | AC004825.2 | turquoise |
| ENSG00000279696 | PANO1 | turquoise |
| ENSG00000279716 | AC004241.2 | turquoise |
| ENSG00000279766 | AL627230.2 | turquoise |
| ENSG00000279799 | AL139384.1 | turquoise |
| ENSG00000279800 | AL157813.1 | turquoise |
| ENSG00000279801 | AC079684.1 | turquoise |
| ENSG00000279803 | AC020656.2 | turquoise |
| ENSG00000279858 | PPP4R3B | turquoise |
| ENSG00000279865 | AC233755.1 | turquoise |
| ENSG00000279917 | SYNRG | turquoise |
| ENSG00000279923 | Metazoa_SRP | turquoise |
| ENSG00000279930 | NUDT18 | turquoise |
| ENSG00000279968 | AL354718.1 | turquoise |
| ENSG00000280003 | AC048341.3 | turquoise |
| ENSG00000280007 | AL161431.1 | turquoise |
| ENSG00000280027 | AL138781.2 | turquoise |
| ENSG00000280033 | MIR6503 | turquoise |
| ENSG00000280035 | AC092111.1 | turquoise |
| ENSG00000280071 | AC012645.4 | turquoise |
| ENSG00000280077 | AC018695.6 | turquoise |
| ENSG00000280079 | AC026367.2 | turquoise |
| ENSG00000280106 | AC104971.4 | turquoise |
| ENSG00000280123 | AL121832.3 | turquoise |
| ENSG00000280135 | AC087741.2 | turquoise |
| ENSG00000280143 | AC025031.4 | turquoise |
| ENSG00000280149 | AP003419.4 | turquoise |
| ENSG00000280153 | AL512652.1 | turquoise |
| ENSG00000280157 | STPG3-AS1 | turquoise |
| ENSG00000280160 | AC242842.1 | turquoise |

| | | |
|---|---|---|
| ENSG00000280163 | AL355073.2 | turquoise |
| ENSG00000280187 | AC022306.2 | turquoise |
| ENSG00000280194 | AC090527.3 | turquoise |
| ENSG00000280198 | AC092747.4 | turquoise |
| ENSG00000280202 | AC091982.4 | turquoise |
| ENSG00000280211 | AC244157.2 | turquoise |
| ENSG00000280216 | AC145285.7 | turquoise |
| ENSG00000280231 | AC084824.4 | turquoise |
| ENSG00000280237 | IKBKGP1 | turquoise |
| ENSG00000280239 | AL355001.2 | turquoise |
| ENSG00000280254 | MIR4477A | turquoise |
| ENSG00000280303 | WHAMMP3 | turquoise |
| ENSG00000280334 | AC084824.5 | turquoise |
| ENSG00000280351 | HIST1H4I | turquoise |
| ENSG00000280367 | AL163051.2 | turquoise |
| ENSG00000280374 | AC245014.3 | turquoise |
| ENSG00000280385 | TADA2A | turquoise |
| ENSG00000280401 | AC127024.6 | turquoise |
| ENSG00000280402 | AC009118.3 | turquoise |
| ENSG00000280417 | KCNE1B | turquoise |
| ENSG00000280433 | FRG1HP | turquoise |
| ENSG00000280434 | AL133243.2 | turquoise |
| ENSG00000280486 | AC105265.4 | turquoise |
| ENSG00000280543 | AC018926.2 | turquoise |
| ENSG00000280604 | HERC2P2 | turquoise |
| ENSG00000280649 | AC130650.2 | turquoise |
| ENSG00000280670 | IGKV1D-13 | turquoise |
| ENSG00000280721 | AC002550.2 | turquoise |
| ENSG00000280734 | RAB7B | turquoise |
| ENSG00000280798 | DACH1 | turquoise |
| ENSG00000280852 | AL136295.6 | turquoise |
| ENSG00000281103 | MIR7111 | turquoise |
| ENSG00000281162 | AC005840.5 | turquoise |
| ENSG00000281332 | AC142472.1 | turquoise |
| ENSG00000281404 | AC105137.3 | turquoise |
| ENSG00000281469 | AC006111.3 | turquoise |
| ENSG00000281571 | AC092117.1 | turquoise |
| ENSG00000281649 | AL133216.2 | turquoise |
| ENSG00000282100 | AC023510.2 | turquoise |
| ENSG00000282386 | AC244196.5 | turquoise |
| ENSG00000282393 | AL158063.1 | turquoise |
| ENSG00000282416 | AL513314.2 | turquoise |
| ENSG00000282431 | AC096642.1 | turquoise |
| ENSG00000282458 | HIST1H2AE | turquoise |
| ENSG00000282499 | LINC00869 | turquoise |
| ENSG00000282951 | F8A3 | turquoise |
| ENSG00000282978 | AL138820.1 | turquoise |
| ENSG00000283023 | F8A1 | turquoise |

| | | |
|---|---|---|
| ENSG00000283064 | GTSE1-AS1 | turquoise |
| ENSG00000283078 | AC010654.1 | turquoise |
| ENSG00000283103 | AC010331.1 | turquoise |
| ENSG00000283196 | AC138649.2 | turquoise |
| ENSG00000283209 | MARCKS | turquoise |
| ENSG00000283234 | ZNF670 | turquoise |
| ENSG00000283236 | AC080038.2 | turquoise |
| ENSG00000283317 | AC005332.8 | turquoise |
| ENSG00000283498 | AC087392.5 | turquoise |
| ENSG00000283525 | AL357033.4 | turquoise |
| ENSG00000283602 | AC007996.1 | turquoise |
| ENSG00000283632 | AC089999.3 | turquoise |
| ENSG00000283633 | AL354696.1 | turquoise |
| ENSG00000283696 | AC079174.2 | turquoise |
| ENSG00000283709 | AC011462.4 | turquoise |
| ENSG00000283787 | AL138995.1 | turquoise |
| ENSG00000283839 | AL442128.2 | turquoise |
| ENSG00000283897 | AC097634.3 | turquoise |
| ENSG00000283959 | AC233755.2 | turquoise |
| ENSG00000284128 | AL035252.3 | turquoise |
| ENSG00000284308 | AC092376.2 | turquoise |
| ENSG00000284543 | AL162274.2 | turquoise |
| ENSG00000284552 | AC107075.1 | turquoise |
| ENSG00000284606 | AC009093.6 | turquoise |
| ENSG00000284644 | AC139100.2 | turquoise |
| ENSG00000284649 | AL627171.1 | turquoise |
| ENSG00000284664 | AC145423.3 | turquoise |
| ENSG00000284691 | AC135050.6 | turquoise |
| ENSG00000284707 | PRICKLE4 | turquoise |
| ENSG00000284719 | AL133342.1 | turquoise |
| ENSG00000284734 | AL359513.1 | turquoise |
| ENSG00000284735 | MIR6859-1 | turquoise |
| ENSG00000001036 | ZNF229 | yellow |
| ENSG00000002549 | AC018529.2 | yellow |
| ENSG00000002834 | AP004609.3 | yellow |
| ENSG00000003400 | AL354696.2 | yellow |
| ENSG00000003402 | MIR6819 | yellow |
| ENSG00000004468 | AC023830.3 | yellow |
| ENSG00000004961 | ACACA | yellow |
| ENSG00000005108 | TMEM191B | yellow |
| ENSG00000005339 | TBC1D3E | yellow |
| ENSG00000008086 | AC015819.2 | yellow |
| ENSG00000008130 | MRM1 | yellow |
| ENSG00000008283 | AC000403.1 | yellow |
| ENSG00000010030 | MIR6859-4 | yellow |
| ENSG00000010404 | AC005332.10 | yellow |
| ENSG00000011198 | AC087239.1 | yellow |
| ENSG00000011422 | AL391903.3 | yellow |

| | | |
|---|---|---|
| ENSG00000013374 | AL136295.7 | yellow |
| ENSG00000013523 | AC007325.4 | yellow |
| ENSG00000013583 | BX539320.1 | yellow |
| ENSG00000014257 | AC145207.9 | yellow |
| ENSG00000019485 | AC025031.5 | yellow |
| ENSG00000020577 | AC002310.6 | yellow |
| ENSG00000021355 | AL031587.5 | yellow |
| ENSG00000023171 | AC092645.1 | yellow |
| ENSG00000023318 | AL031602.1 | yellow |
| ENSG00000025708 | AC093909.6 | yellow |
| ENSG00000026950 | AC092611.3 | yellow |
| ENSG00000028528 | AC112255.1 | yellow |
| ENSG00000029153 | AC132938.5 | yellow |
| ENSG00000033327 | AC015813.5 | yellow |
| ENSG00000033800 | AC073130.3 | yellow |
| ENSG00000038945 | AC243964.3 | yellow |
| ENSG00000043462 | AL590326.2 | yellow |
| ENSG00000044459 | LINC01451 | yellow |
| ENSG00000047644 | AL118508.3 | yellow |
| ENSG00000049245 | AC105052.3 | yellow |
| ENSG00000050165 | AC008894.3 | yellow |
| ENSG00000051620 | AC015813.6 | yellow |
| ENSG00000054523 | AL121753.2 | yellow |
| ENSG00000055130 | Z97653.2 | yellow |
| ENSG00000055332 | AC000123.2 | yellow |
| ENSG00000057704 | AL078621.3 | yellow |
| ENSG00000058799 | AC002525.1 | yellow |
| ENSG00000059378 | Z98885.3 | yellow |
| ENSG00000059804 | AC012513.3 | yellow |
| ENSG00000060491 | AGPAT4-IT1 | yellow |
| ENSG00000060656 | AC046185.3 | yellow |
| ENSG00000064763 | AC003973.3 | yellow |
| ENSG00000064999 | AC130469.2 | yellow |
| ENSG00000065361 | AL359183.1 | yellow |
| ENSG00000065427 | AC112497.1 | yellow |
| ENSG00000067066 | AL590560.3 | yellow |
| ENSG00000067992 | FO538757.1 | yellow |
| ENSG00000068024 | AC090498.1 | yellow |
| ENSG00000068079 | AP003733.4 | yellow |
| ENSG00000068383 | AC083843.3 | yellow |
| ENSG00000068697 | AC002094.5 | yellow |
| ENSG00000069849 | AC093458.2 | yellow |
| ENSG00000069966 | AC020658.7 | yellow |
| ENSG00000070047 | AC006486.2 | yellow |
| ENSG00000070501 | AP000437.1 | yellow |
| ENSG00000070731 | AC009086.3 | yellow |
| ENSG00000070759 | AC120057.4 | yellow |
| ENSG00000071054 | AC092919.2 | yellow |

| | | |
|---|---|---|
| ENSG00000072110 | AP001273.1 | yellow |
| ENSG00000072121 | AC006128.1 | yellow |
| ENSG00000072694 | AC067931.1 | yellow |
| ENSG00000073331 | AC006077.2 | yellow |
| ENSG00000073737 | BCLAF1P2 | yellow |
| ENSG00000074181 | AC111170.3 | yellow |
| ENSG00000074755 | AC009090.5 | yellow |
| ENSG00000075275 | AC068880.4 | yellow |
| ENSG00000075413 | AC006511.3 | yellow |
| ENSG00000075426 | AC079331.2 | yellow |
| ENSG00000075785 | AC022417.1 | yellow |
| ENSG00000075884 | AL032819.2 | yellow |
| ENSG00000076108 | GVQW2 | yellow |
| ENSG00000076513 | AC097637.2 | yellow |
| ENSG00000076555 | AC008079.2 | yellow |
| ENSG00000077044 | AC007342.9 | yellow |
| ENSG00000078081 | AC116407.4 | yellow |
| ENSG00000078124 | AC011676.5 | yellow |
| ENSG00000078618 | FP565260.6 | yellow |
| ENSG00000079156 | AL353763.2 | yellow |
| ENSG00000079263 | AC011447.7 | yellow |
| ENSG00000079277 | AC008555.8 | yellow |
| ENSG00000079385 | AC023632.6 | yellow |
| ENSG00000080007 | AL096816.1 | yellow |
| ENSG00000080815 | AP000892.3 | yellow |
| ENSG00000080854 | AC004877.2 | yellow |
| ENSG00000081913 | AC133065.6 | yellow |
| ENSG00000082397 | AL359510.2 | yellow |
| ENSG00000084110 | AC135050.7 | yellow |
| ENSG00000085063 | AC040160.2 | yellow |
| ENSG00000085552 | AC022107.1 | yellow |
| ENSG00000085741 | AD000864.1 | yellow |
| ENSG00000086288 | AC087163.3 | yellow |
| ENSG00000087157 | AC005831.1 | yellow |
| ENSG00000087206 | AC106886.4 | yellow |
| ENSG00000087237 | AL022326.2 | yellow |
| ENSG00000087258 | AL031719.2 | yellow |
| ENSG00000087589 | MIR4697HG | yellow |
| ENSG00000087995 | AC011498.7 | yellow |
| ENSG00000088827 | AC233723.2 | yellow |
| ENSG00000089127 | AC067931.2 | yellow |
| ENSG00000090316 | AC009084.2 | yellow |
| ENSG00000090339 | AC127496.7 | yellow |
| ENSG00000090659 | AP002364.1 | yellow |
| ENSG00000092036 | AC019080.5 | yellow |
| ENSG00000092098 | AP000648.3 | yellow |
| ENSG00000093134 | AC022532.1 | yellow |
| ENSG00000093167 | AC093525.10 | yellow |

| | | |
|---|---|---|
| ENSG00000099250 | AC096887.2 | yellow |
| ENSG00000099860 | FP565260.7 | yellow |
| ENSG00000099957 | AL031595.3 | yellow |
| ENSG00000100065 | AC005329.3 | yellow |
| ENSG00000100077 | ASAP1-IT2 | yellow |
| ENSG00000100220 | AJ239328.1 | yellow |
| ENSG00000100226 | AC245100.8 | yellow |
| ENSG00000100266 | CCDC163 | yellow |
| ENSG00000100330 | LINC01943 | yellow |
| ENSG00000100336 | LINC01232 | yellow |
| ENSG00000100342 | LINC00294 | yellow |
| ENSG00000100422 | AC025048.6 | yellow |
| ENSG00000100479 | TRG-AS1 | yellow |
| ENSG00000100504 | AC005035.1 | yellow |
| ENSG00000100596 | AC018645.3 | yellow |
| ENSG00000100731 | LINC01176 | yellow |
| ENSG00000100911 | AC019226.1 | yellow |
| ENSG00000100918 | AC241585.3 | yellow |
| ENSG00000100926 | EBLN3P | yellow |
| ENSG00000100938 | AC091046.3 | yellow |
| ENSG00000101236 | AL358472.4 | yellow |
| ENSG00000101265 | AC016588.2 | yellow |
| ENSG00000101342 | AC092299.1 | yellow |
| ENSG00000101347 | AC245427.8 | yellow |
| ENSG00000101474 | WASH5P | yellow |
| ENSG00000101493 | TRBV25-1 | yellow |
| ENSG00000101846 | AC008537.4 | yellow |
| ENSG00000101849 | AC110994.2 | yellow |
| ENSG00000102010 | AC116618.1 | yellow |
| ENSG00000102034 | AL353759.1 | yellow |
| ENSG00000102096 | AL137077.2 | yellow |
| ENSG00000102524 | AC010642.2 | yellow |
| ENSG00000102699 | AC006453.2 | yellow |
| ENSG00000102897 | AC106858.1 | yellow |
| ENSG00000102921 | AL590556.2 | yellow |
| ENSG00000102996 | AC074141.1 | yellow |
| ENSG00000103111 | AL831711.1 | yellow |
| ENSG00000103194 | MIR1244-2 | yellow |
| ENSG00000103313 | AC005622.1 | yellow |
| ENSG00000103356 | AC116353.5 | yellow |
| ENSG00000103569 | EXOC3L2 | yellow |
| ENSG00000103642 | AP000547.3 | yellow |
| ENSG00000104714 | AL592295.4 | yellow |
| ENSG00000104833 | FAM238C | yellow |
| ENSG00000104880 | AC051649.2 | yellow |
| ENSG00000104951 | AC096667.1 | yellow |
| ENSG00000105185 | AC011416.4 | yellow |
| ENSG00000105339 | AP002851.1 | yellow |

| | | |
|---|---|---|
| ENSG00000105402 | AP000356.3 | yellow |
| ENSG00000105656 | C2orf81 | yellow |
| ENSG00000105717 | LINC01226 | yellow |
| ENSG00000105939 | AC106774.10 | yellow |
| ENSG00000105948 | AC105233.5 | yellow |
| ENSG00000106070 | AC074386.1 | yellow |
| ENSG00000106100 | AC009093.8 | yellow |
| ENSG00000106605 | AL161756.3 | yellow |
| ENSG00000106785 | AC073111.5 | yellow |
| ENSG00000106804 | AC079781.5 | yellow |
| ENSG00000106868 | AL033527.5 | yellow |
| ENSG00000107201 | AC099063.4 | yellow |
| ENSG00000107338 | AL139424.3 | yellow |
| ENSG00000107341 | UBE2R2 | yellow |
| ENSG00000107537 | PHYH | yellow |
| ENSG00000107611 | CUBN | yellow |
| ENSG00000107796 | ACTA2 | yellow |
| ENSG00000107960 | STN1 | yellow |
| ENSG00000108100 | CCNY | yellow |
| ENSG00000108387 | 4-Sep | yellow |
| ENSG00000108424 | KPNB1 | yellow |
| ENSG00000108448 | TRIM16L | yellow |
| ENSG00000108679 | LGALS3BP | yellow |
| ENSG00000108691 | CCL2 | yellow |
| ENSG00000108771 | DHX58 | yellow |
| ENSG00000108861 | DUSP3 | yellow |
| ENSG00000108984 | MAP2K6 | yellow |
| ENSG00000109047 | RCVRN | yellow |
| ENSG00000109220 | CHIC2 | yellow |
| ENSG00000109743 | BST1 | yellow |
| ENSG00000109854 | HTATIP2 | yellow |
| ENSG00000109861 | CTSC | yellow |
| ENSG00000110057 | UNC93B1 | yellow |
| ENSG00000110077 | MS4A6A | yellow |
| ENSG00000110079 | MS4A4A | yellow |
| ENSG00000110492 | MDK | yellow |
| ENSG00000110660 | SLC35F2 | yellow |
| ENSG00000110713 | NUP98 | yellow |
| ENSG00000111181 | SLC6A12 | yellow |
| ENSG00000111331 | OAS3 | yellow |
| ENSG00000111335 | OAS2 | yellow |
| ENSG00000111490 | TBC1D30 | yellow |
| ENSG00000111674 | ENO2 | yellow |
| ENSG00000111801 | BTN3A3 | yellow |
| ENSG00000111837 | MAK | yellow |
| ENSG00000111913 | RIPOR2 | yellow |
| ENSG00000112053 | SLC26A8 | yellow |
| ENSG00000112096 | SOD2 | yellow |

| | | |
|---|---|---|
| ENSG00000112335 | SNX3 | yellow |
| ENSG00000112343 | TRIM38 | yellow |
| ENSG00000112367 | FIG4 | yellow |
| ENSG00000112584 | FAM120B | yellow |
| ENSG00000112773 | FAM46A | yellow |
| ENSG00000113269 | RNF130 | yellow |
| ENSG00000113273 | ARSB | yellow |
| ENSG00000113368 | LMNB1 | yellow |
| ENSG00000113494 | PRLR | yellow |
| ENSG00000114013 | CD86 | yellow |
| ENSG00000114268 | PFKFB4 | yellow |
| ENSG00000114491 | UMPS | yellow |
| ENSG00000114544 | SLC41A3 | yellow |
| ENSG00000114554 | PLXNA1 | yellow |
| ENSG00000114670 | NEK11 | yellow |
| ENSG00000114745 | GORASP1 | yellow |
| ENSG00000114784 | EIF1B | yellow |
| ENSG00000115155 | OTOF | yellow |
| ENSG00000115163 | CENPA | yellow |
| ENSG00000115267 | IFIH1 | yellow |
| ENSG00000115415 | STAT1 | yellow |
| ENSG00000115687 | PASK | yellow |
| ENSG00000115828 | QPCT | yellow |
| ENSG00000115919 | KYNU | yellow |
| ENSG00000115956 | PLEK | yellow |
| ENSG00000116514 | RNF19B | yellow |
| ENSG00000116663 | FBXO6 | yellow |
| ENSG00000116701 | NCF2 | yellow |
| ENSG00000117139 | KDM5B | yellow |
| ENSG00000117228 | GBP1 | yellow |
| ENSG00000117594 | HSD11B1 | yellow |
| ENSG00000117758 | STX12 | yellow |
| ENSG00000118162 | KPTN | yellow |
| ENSG00000118217 | ATF6 | yellow |
| ENSG00000118900 | UBN1 | yellow |
| ENSG00000119139 | TJP2 | yellow |
| ENSG00000119203 | CPSF3 | yellow |
| ENSG00000119686 | FLVCR2 | yellow |
| ENSG00000119714 | GPR68 | yellow |
| ENSG00000119723 | COQ6 | yellow |
| ENSG00000119862 | LGALSL | yellow |
| ENSG00000119917 | IFIT3 | yellow |
| ENSG00000119922 | IFIT2 | yellow |
| ENSG00000120217 | CD274 | yellow |
| ENSG00000120318 | ARAP3 | yellow |
| ENSG00000120594 | PLXDC2 | yellow |
| ENSG00000120709 | FAM53C | yellow |
| ENSG00000121060 | TRIM25 | yellow |

| | | |
|---|---|---|
| ENSG00000121236 | TRIM6 | yellow |
| ENSG00000121297 | TSHZ3 | yellow |
| ENSG00000121542 | SEC22A | yellow |
| ENSG00000121691 | CAT | yellow |
| ENSG00000121797 | CCRL2 | yellow |
| ENSG00000121858 | TNFSF10 | yellow |
| ENSG00000122122 | SASH3 | yellow |
| ENSG00000122378 | FAM213A | yellow |
| ENSG00000122729 | ACO1 | yellow |
| ENSG00000122783 | C7orf49 | yellow |
| ENSG00000123130 | ACOT9 | yellow |
| ENSG00000123191 | ATP7B | yellow |
| ENSG00000123213 | NLN | yellow |
| ENSG00000123444 | KBTBD4 | yellow |
| ENSG00000123453 | SARDH | yellow |
| ENSG00000123609 | NMI | yellow |
| ENSG00000123610 | TNFAIP6 | yellow |
| ENSG00000124126 | PREX1 | yellow |
| ENSG00000124201 | ZNFX1 | yellow |
| ENSG00000124226 | RNF114 | yellow |
| ENSG00000124256 | ZBP1 | yellow |
| ENSG00000124356 | STAMBP | yellow |
| ENSG00000124357 | NAGK | yellow |
| ENSG00000124688 | MAD2L1BP | yellow |
| ENSG00000125148 | MT2A | yellow |
| ENSG00000125347 | IRF1 | yellow |
| ENSG00000125355 | TMEM255A | yellow |
| ENSG00000125485 | DDX31 | yellow |
| ENSG00000125538 | IL1B | yellow |
| ENSG00000125779 | PANK2 | yellow |
| ENSG00000125826 | RBCK1 | yellow |
| ENSG00000125834 | STK35 | yellow |
| ENSG00000125875 | TBC1D20 | yellow |
| ENSG00000125952 | MAX | yellow |
| ENSG00000126012 | KDM5C | yellow |
| ENSG00000126067 | PSMB2 | yellow |
| ENSG00000126251 | GPR42 | yellow |
| ENSG00000126262 | FFAR2 | yellow |
| ENSG00000126351 | THRA | yellow |
| ENSG00000126709 | IFI6 | yellow |
| ENSG00000126953 | TIMM8A | yellow |
| ENSG00000128016 | ZFP36 | yellow |
| ENSG00000128203 | ASPHD2 | yellow |
| ENSG00000128284 | APOL3 | yellow |
| ENSG00000128335 | APOL2 | yellow |
| ENSG00000128342 | LIF | yellow |
| ENSG00000128383 | APOBEC3A | yellow |
| ENSG00000128512 | DOCK4 | yellow |

| | | |
|---|---|---|
| ENSG00000128594 | LRRC4 | yellow |
| ENSG00000128604 | IRF5 | yellow |
| ENSG00000129295 | LRRC6 | yellow |
| ENSG00000129657 | SEC14L1 | yellow |
| ENSG00000129667 | RHBDF2 | yellow |
| ENSG00000129682 | FGF13 | yellow |
| ENSG00000129691 | ASH2L | yellow |
| ENSG00000129933 | MAU2 | yellow |
| ENSG00000130038 | CRACR2A | yellow |
| ENSG00000130066 | SAT1 | yellow |
| ENSG00000130303 | BST2 | yellow |
| ENSG00000130487 | KLHDC7B | yellow |
| ENSG00000130489 | SCO2 | yellow |
| ENSG00000130589 | HELZ2 | yellow |
| ENSG00000130703 | OSBPL2 | yellow |
| ENSG00000130813 | C19orf66 | yellow |
| ENSG00000131126 | TEX101 | yellow |
| ENSG00000131236 | CAP1 | yellow |
| ENSG00000131389 | SLC6A6 | yellow |
| ENSG00000131724 | IL13RA1 | yellow |
| ENSG00000131778 | CHD1L | yellow |
| ENSG00000132109 | TRIM21 | yellow |
| ENSG00000132155 | RAF1 | yellow |
| ENSG00000132256 | TRIM5 | yellow |
| ENSG00000132259 | CNGA4 | yellow |
| ENSG00000132274 | TRIM22 | yellow |
| ENSG00000132357 | CARD6 | yellow |
| ENSG00000132359 | RAP1GAP2 | yellow |
| ENSG00000132475 | H3F3B | yellow |
| ENSG00000132530 | XAF1 | yellow |
| ENSG00000132694 | ARHGEF11 | yellow |
| ENSG00000132780 | NASP | yellow |
| ENSG00000133106 | EPSTI1 | yellow |
| ENSG00000133216 | EPHB2 | yellow |
| ENSG00000133313 | CNDP2 | yellow |
| ENSG00000133318 | RTN3 | yellow |
| ENSG00000133574 | GIMAP4 | yellow |
| ENSG00000133687 | TMTC1 | yellow |
| ENSG00000134030 | CTIF | yellow |
| ENSG00000134253 | TRIM45 | yellow |
| ENSG00000134321 | RSAD2 | yellow |
| ENSG00000134326 | CMPK2 | yellow |
| ENSG00000134333 | LDHA | yellow |
| ENSG00000134470 | IL15RA | yellow |
| ENSG00000134531 | EMP1 | yellow |
| ENSG00000134575 | ACP2 | yellow |
| ENSG00000134809 | TIMM10 | yellow |
| ENSG00000134986 | NREP | yellow |

| | | |
|---|---|---|
| ENSG00000134996 | OSTF1 | yellow |
| ENSG00000135047 | CTSL | yellow |
| ENSG00000135114 | OASL | yellow |
| ENSG00000135148 | TRAFD1 | yellow |
| ENSG00000135211 | TMEM60 | yellow |
| ENSG00000135218 | CD36 | yellow |
| ENSG00000135245 | HILPDA | yellow |
| ENSG00000135363 | LMO2 | yellow |
| ENSG00000135365 | PHF21A | yellow |
| ENSG00000135378 | PRRG4 | yellow |
| ENSG00000135414 | GDF11 | yellow |
| ENSG00000135469 | COQ10A | yellow |
| ENSG00000135540 | NHSL1 | yellow |
| ENSG00000135604 | STX11 | yellow |
| ENSG00000135636 | DYSF | yellow |
| ENSG00000135821 | GLUL | yellow |
| ENSG00000135838 | NPL | yellow |
| ENSG00000135842 | FAM129A | yellow |
| ENSG00000135899 | SP110 | yellow |
| ENSG00000135900 | MRPL44 | yellow |
| ENSG00000136147 | PHF11 | yellow |
| ENSG00000136156 | ITM2B | yellow |
| ENSG00000136167 | LCP1 | yellow |
| ENSG00000136193 | SCRN1 | yellow |
| ENSG00000136231 | IGF2BP3 | yellow |
| ENSG00000136319 | TTC5 | yellow |
| ENSG00000136436 | CALCOCO2 | yellow |
| ENSG00000136449 | MYCBPAP | yellow |
| ENSG00000136514 | RTP4 | yellow |
| ENSG00000136689 | IL1RN | yellow |
| ENSG00000136770 | DNAJC1 | yellow |
| ENSG00000136816 | TOR1B | yellow |
| ENSG00000136826 | KLF4 | yellow |
| ENSG00000136827 | TOR1A | yellow |
| ENSG00000136868 | SLC31A1 | yellow |
| ENSG00000136891 | TEX10 | yellow |
| ENSG00000136932 | TRMO | yellow |
| ENSG00000137094 | DNAJB5 | yellow |
| ENSG00000137200 | CMTR1 | yellow |
| ENSG00000137275 | RIPK1 | yellow |
| ENSG00000137434 | C6orf52 | yellow |
| ENSG00000137462 | TLR2 | yellow |
| ENSG00000137628 | DDX60 | yellow |
| ENSG00000137752 | CASP1 | yellow |
| ENSG00000137757 | CASP5 | yellow |
| ENSG00000137767 | SQOR | yellow |
| ENSG00000137819 | PAQR5 | yellow |
| ENSG00000137842 | TMEM62 | yellow |

| | | |
|---|---|---|
| ENSG00000137959 | IFI44L | yellow |
| ENSG00000137965 | IFI44 | yellow |
| ENSG00000138029 | HADHB | yellow |
| ENSG00000138035 | PNPT1 | yellow |
| ENSG00000138119 | MYOF | yellow |
| ENSG00000138131 | LOXL4 | yellow |
| ENSG00000138185 | ENTPD1 | yellow |
| ENSG00000138433 | CIR1 | yellow |
| ENSG00000138496 | PARP9 | yellow |
| ENSG00000138613 | APH1B | yellow |
| ENSG00000138639 | ARHGAP24 | yellow |
| ENSG00000138642 | HERC6 | yellow |
| ENSG00000138646 | HERC5 | yellow |
| ENSG00000138755 | CXCL9 | yellow |
| ENSG00000138760 | SCARB2 | yellow |
| ENSG00000138785 | INTS12 | yellow |
| ENSG00000139083 | ETV6 | yellow |
| ENSG00000139174 | PRICKLE1 | yellow |
| ENSG00000139178 | C1RL | yellow |
| ENSG00000139438 | FAM222A | yellow |
| ENSG00000139572 | GPR84 | yellow |
| ENSG00000139618 | BRCA2 | yellow |
| ENSG00000139718 | SETD1B | yellow |
| ENSG00000139880 | CDH24 | yellow |
| ENSG00000140105 | WARS | yellow |
| ENSG00000140280 | LYSMD2 | yellow |
| ENSG00000140332 | TLE3 | yellow |
| ENSG00000140350 | ANP32A | yellow |
| ENSG00000140443 | IGF1R | yellow |
| ENSG00000140455 | USP3 | yellow |
| ENSG00000140459 | CYP11A1 | yellow |
| ENSG00000140464 | PML | yellow |
| ENSG00000140526 | ABHD2 | yellow |
| ENSG00000140563 | MCTP2 | yellow |
| ENSG00000140749 | IGSF6 | yellow |
| ENSG00000140750 | ARHGAP17 | yellow |
| ENSG00000140795 | MYLK3 | yellow |
| ENSG00000140807 | NKD1 | yellow |
| ENSG00000140853 | NLRC5 | yellow |
| ENSG00000140941 | MAP1LC3B | yellow |
| ENSG00000140945 | CDH13 | yellow |
| ENSG00000140968 | IRF8 | yellow |
| ENSG00000141013 | GAS8 | yellow |
| ENSG00000141298 | SSH2 | yellow |
| ENSG00000141506 | PIK3R5 | yellow |
| ENSG00000141542 | RAB40B | yellow |
| ENSG00000141560 | FN3KRP | yellow |
| ENSG00000141569 | TRIM65 | yellow |

| | | |
|---|---|---|
| ENSG00000141574 | SECTM1 | yellow |
| ENSG00000141655 | TNFRSF11A | yellow |
| ENSG00000141664 | ZCCHC2 | yellow |
| ENSG00000142089 | IFITM3 | yellow |
| ENSG00000142208 | AKT1 | yellow |
| ENSG00000142444 | TIMM29 | yellow |
| ENSG00000142599 | RERE | yellow |
| ENSG00000142920 | AZIN2 | yellow |
| ENSG00000142961 | MOB3C | yellow |
| ENSG00000143226 | FCGR2A | yellow |
| ENSG00000143344 | RGL1 | yellow |
| ENSG00000143376 | SNX27 | yellow |
| ENSG00000143390 | RFX5 | yellow |
| ENSG00000143486 | EIF2D | yellow |
| ENSG00000143753 | DEGS1 | yellow |
| ENSG00000143869 | GDF7 | yellow |
| ENSG00000143878 | RHOB | yellow |
| ENSG00000143891 | GALM | yellow |
| ENSG00000144118 | RALB | yellow |
| ENSG00000144130 | NT5DC4 | yellow |
| ENSG00000144655 | CSRNP1 | yellow |
| ENSG00000144711 | IQSEC1 | yellow |
| ENSG00000145016 | RUBCN | yellow |
| ENSG00000145214 | DGKQ | yellow |
| ENSG00000145287 | PLAC8 | yellow |
| ENSG00000145545 | SRD5A1 | yellow |
| ENSG00000145555 | MYO10 | yellow |
| ENSG00000145685 | LHFPL2 | yellow |
| ENSG00000145819 | ARHGAP26 | yellow |
| ENSG00000146143 | PRIM2 | yellow |
| ENSG00000146416 | AIG1 | yellow |
| ENSG00000146425 | DYNLT1 | yellow |
| ENSG00000146858 | ZC3HAV1L | yellow |
| ENSG00000146859 | TMEM140 | yellow |
| ENSG00000147100 | SLC16A2 | yellow |
| ENSG00000147119 | CHST7 | yellow |
| ENSG00000147202 | DIAPH2 | yellow |
| ENSG00000147364 | FBXO25 | yellow |
| ENSG00000147416 | ATP6V1B2 | yellow |
| ENSG00000147642 | SYBU | yellow |
| ENSG00000148175 | STOM | yellow |
| ENSG00000148187 | MRRF | yellow |
| ENSG00000148219 | ASTN2 | yellow |
| ENSG00000148384 | INPP5E | yellow |
| ENSG00000148400 | NOTCH1 | yellow |
| ENSG00000148411 | NACC2 | yellow |
| ENSG00000148600 | CDHR1 | yellow |
| ENSG00000148841 | ITPRIP | yellow |

| | | |
|---|---|---|
| ENSG00000149131 | SERPING1 | yellow |
| ENSG00000149177 | PTPRJ | yellow |
| ENSG00000149201 | CCDC81 | yellow |
| ENSG00000149269 | PAK1 | yellow |
| ENSG00000149531 | FRG1BP | yellow |
| ENSG00000149634 | SPATA25 | yellow |
| ENSG00000150337 | FCGR1A | yellow |
| ENSG00000150527 | CTAGE5 | yellow |
| ENSG00000150556 | LYPD6B | yellow |
| ENSG00000150873 | C2orf50 | yellow |
| ENSG00000150961 | SEC24D | yellow |
| ENSG00000151532 | VTI1A | yellow |
| ENSG00000151552 | QDPR | yellow |
| ENSG00000151729 | SLC25A4 | yellow |
| ENSG00000151882 | CCL28 | yellow |
| ENSG00000152147 | GEMIN6 | yellow |
| ENSG00000152213 | ARL11 | yellow |
| ENSG00000152229 | PSTPIP2 | yellow |
| ENSG00000152766 | ANKRD22 | yellow |
| ENSG00000152778 | IFIT5 | yellow |
| ENSG00000153029 | MR1 | yellow |
| ENSG00000153071 | DAB2 | yellow |
| ENSG00000153815 | CMIP | yellow |
| ENSG00000154122 | ANKH | yellow |
| ENSG00000154258 | ABCA9 | yellow |
| ENSG00000154305 | MIA3 | yellow |
| ENSG00000154451 | GBP5 | yellow |
| ENSG00000154645 | CHODL | yellow |
| ENSG00000154928 | EPHB1 | yellow |
| ENSG00000155363 | MOV10 | yellow |
| ENSG00000155629 | PIK3AP1 | yellow |
| ENSG00000156500 | FAM122C | yellow |
| ENSG00000156587 | UBE2L6 | yellow |
| ENSG00000156639 | ZFAND3 | yellow |
| ENSG00000156675 | RAB11FIP1 | yellow |
| ENSG00000157064 | NMNAT2 | yellow |
| ENSG00000157227 | MMP14 | yellow |
| ENSG00000157470 | FAM81A | yellow |
| ENSG00000157601 | MX1 | yellow |
| ENSG00000157693 | TMEM268 | yellow |
| ENSG00000157827 | FMNL2 | yellow |
| ENSG00000157933 | SKI | yellow |
| ENSG00000158050 | DUSP2 | yellow |
| ENSG00000158163 | DZIP1L | yellow |
| ENSG00000158186 | MRAS | yellow |
| ENSG00000158270 | COLEC12 | yellow |
| ENSG00000158406 | HIST1H4H | yellow |
| ENSG00000158488 | CD1E | yellow |

| | | |
|---|---|---|
| ENSG00000158714 | SLAMF8 | yellow |
| ENSG00000158769 | F11R | yellow |
| ENSG00000158806 | NPM2 | yellow |
| ENSG00000158859 | ADAMTS4 | yellow |
| ENSG00000159189 | C1QC | yellow |
| ENSG00000159228 | CBR1 | yellow |
| ENSG00000159784 | FAM131B | yellow |
| ENSG00000159904 | ZNF890P | yellow |
| ENSG00000160117 | ANKLE1 | yellow |
| ENSG00000160190 | SLC37A1 | yellow |
| ENSG00000160201 | U2AF1 | yellow |
| ENSG00000160216 | AGPAT3 | yellow |
| ENSG00000160271 | RALGDS | yellow |
| ENSG00000160310 | PRMT2 | yellow |
| ENSG00000160695 | VPS11 | yellow |
| ENSG00000160710 | ADAR | yellow |
| ENSG00000160785 | SLC25A44 | yellow |
| ENSG00000160932 | LY6E | yellow |
| ENSG00000161217 | PCYT1A | yellow |
| ENSG00000161381 | PLXDC1 | yellow |
| ENSG00000161405 | IKZF3 | yellow |
| ENSG00000161647 | MPP3 | yellow |
| ENSG00000162065 | TBC1D24 | yellow |
| ENSG00000162413 | KLHL21 | yellow |
| ENSG00000162433 | AK4 | yellow |
| ENSG00000162512 | SDC3 | yellow |
| ENSG00000162545 | CAMK2N1 | yellow |
| ENSG00000162614 | NEXN | yellow |
| ENSG00000162645 | GBP2 | yellow |
| ENSG00000162654 | GBP4 | yellow |
| ENSG00000162714 | ZNF496 | yellow |
| ENSG00000162745 | OLFML2B | yellow |
| ENSG00000162747 | FCGR3B | yellow |
| ENSG00000162772 | ATF3 | yellow |
| ENSG00000162777 | DENND2D | yellow |
| ENSG00000162909 | CAPN2 | yellow |
| ENSG00000162946 | DISC1 | yellow |
| ENSG00000163026 | WDCP | yellow |
| ENSG00000163041 | H3F3A | yellow |
| ENSG00000163083 | INHBB | yellow |
| ENSG00000163121 | NEURL3 | yellow |
| ENSG00000163131 | CTSS | yellow |
| ENSG00000163219 | ARHGAP25 | yellow |
| ENSG00000163251 | FZD5 | yellow |
| ENSG00000163328 | GPR155 | yellow |
| ENSG00000163520 | FBLN2 | yellow |
| ENSG00000163563 | MNDA | yellow |
| ENSG00000163565 | IFI16 | yellow |

| | | |
|---|---|---|
| ENSG00000163568 | AIM2 | yellow |
| ENSG00000163576 | EFHB | yellow |
| ENSG00000163666 | HESX1 | yellow |
| ENSG00000163754 | GYG1 | yellow |
| ENSG00000163823 | CCR1 | yellow |
| ENSG00000163840 | DTX3L | yellow |
| ENSG00000163932 | PRKCD | yellow |
| ENSG00000163993 | S100P | yellow |
| ENSG00000164054 | SHISA5 | yellow |
| ENSG00000164061 | BSN | yellow |
| ENSG00000164096 | C4orf3 | yellow |
| ENSG00000164111 | ANXA5 | yellow |
| ENSG00000164509 | IL31RA | yellow |
| ENSG00000164574 | GALNT10 | yellow |
| ENSG00000164597 | COG5 | yellow |
| ENSG00000165006 | UBAP1 | yellow |
| ENSG00000165030 | NFIL3 | yellow |
| ENSG00000165092 | ALDH1A1 | yellow |
| ENSG00000165119 | HNRNPK | yellow |
| ENSG00000165168 | CYBB | yellow |
| ENSG00000165185 | KIAA1958 | yellow |
| ENSG00000165526 | RPUSD4 | yellow |
| ENSG00000165646 | SLC18A2 | yellow |
| ENSG00000165949 | IFI27 | yellow |
| ENSG00000166002 | SMCO4 | yellow |
| ENSG00000166016 | ABTB2 | yellow |
| ENSG00000166025 | AMOTL1 | yellow |
| ENSG00000166086 | JAM3 | yellow |
| ENSG00000166272 | WBP1L | yellow |
| ENSG00000166394 | CYB5R2 | yellow |
| ENSG00000166523 | CLEC4E | yellow |
| ENSG00000166579 | NDEL1 | yellow |
| ENSG00000166582 | CENPV | yellow |
| ENSG00000166889 | PATL1 | yellow |
| ENSG00000166924 | NYAP1 | yellow |
| ENSG00000166946 | CCNDBP1 | yellow |
| ENSG00000166997 | CNPY4 | yellow |
| ENSG00000167014 | TERB2 | yellow |
| ENSG00000167186 | COQ7 | yellow |
| ENSG00000167207 | NOD2 | yellow |
| ENSG00000167208 | SNX20 | yellow |
| ENSG00000167460 | TPM4 | yellow |
| ENSG00000167470 | MIDN | yellow |
| ENSG00000167528 | ZNF641 | yellow |
| ENSG00000167601 | AXL | yellow |
| ENSG00000167695 | FAM57A | yellow |
| ENSG00000167995 | BEST1 | yellow |
| ENSG00000168016 | TRANK1 | yellow |

| | | |
|---|---|---|
| ENSG00000168026 | TTC21A | yellow |
| ENSG00000168062 | BATF2 | yellow |
| ENSG00000168214 | RBPJ | yellow |
| ENSG00000168259 | DNAJC7 | yellow |
| ENSG00000168275 | COA6 | yellow |
| ENSG00000168310 | IRF2 | yellow |
| ENSG00000168329 | CX3CR1 | yellow |
| ENSG00000168404 | MLKL | yellow |
| ENSG00000168461 | RAB31 | yellow |
| ENSG00000168610 | STAT3 | yellow |
| ENSG00000168763 | CNNM3 | yellow |
| ENSG00000168824 | HGNC:18790 | yellow |
| ENSG00000168961 | LGALS9 | yellow |
| ENSG00000169136 | ATF5 | yellow |
| ENSG00000169228 | RAB24 | yellow |
| ENSG00000169245 | CXCL10 | yellow |
| ENSG00000169432 | SCN9A | yellow |
| ENSG00000169871 | TRIM56 | yellow |
| ENSG00000169884 | WNT10B | yellow |
| ENSG00000169891 | REPS2 | yellow |
| ENSG00000169905 | TOR1AIP2 | yellow |
| ENSG00000169951 | ZNF764 | yellow |
| ENSG00000170089 | AC106795.1 | yellow |
| ENSG00000170100 | ZNF778 | yellow |
| ENSG00000170439 | METTL7B | yellow |
| ENSG00000170581 | STAT2 | yellow |
| ENSG00000171115 | GIMAP8 | yellow |
| ENSG00000171189 | GRIK1 | yellow |
| ENSG00000171223 | JUNB | yellow |
| ENSG00000171224 | C10orf35 | yellow |
| ENSG00000171310 | CHST11 | yellow |
| ENSG00000171314 | PGAM1 | yellow |
| ENSG00000171365 | CLCN5 | yellow |
| ENSG00000171368 | TPPP | yellow |
| ENSG00000171456 | ASXL1 | yellow |
| ENSG00000171475 | WIPF2 | yellow |
| ENSG00000171608 | PIK3CD | yellow |
| ENSG00000171631 | P2RY6 | yellow |
| ENSG00000171643 | S100Z | yellow |
| ENSG00000171658 | NMRAL2P | yellow |
| ENSG00000171680 | PLEKHG5 | yellow |
| ENSG00000171729 | TMEM51 | yellow |
| ENSG00000171860 | C3AR1 | yellow |
| ENSG00000171914 | TLN2 | yellow |
| ENSG00000172159 | FRMD3 | yellow |
| ENSG00000172183 | ISG20 | yellow |
| ENSG00000172216 | CEBPB | yellow |
| ENSG00000172426 | RSPH9 | yellow |

| | | |
|---|---|---|
| ENSG00000172432 | GTPBP2 | yellow |
| ENSG00000172867 | KRT2 | yellow |
| ENSG00000172878 | METAP1D | yellow |
| ENSG00000172936 | MYD88 | yellow |
| ENSG00000173110 | HSPA6 | yellow |
| ENSG00000173193 | PARP14 | yellow |
| ENSG00000173369 | C1QB | yellow |
| ENSG00000173436 | MINOS1 | yellow |
| ENSG00000173757 | STAT5B | yellow |
| ENSG00000173786 | CNP | yellow |
| ENSG00000173821 | RNF213 | yellow |
| ENSG00000174125 | TLR1 | yellow |
| ENSG00000174151 | CYB561D1 | yellow |
| ENSG00000174721 | FGFBP3 | yellow |
| ENSG00000174837 | ADGRE1 | yellow |
| ENSG00000174876 | AMY1B | yellow |
| ENSG00000175215 | CTDSP2 | yellow |
| ENSG00000175356 | SCUBE2 | yellow |
| ENSG00000175518 | UBQLNL | yellow |
| ENSG00000175550 | DRAP1 | yellow |
| ENSG00000175643 | RMI2 | yellow |
| ENSG00000175764 | TTLL11 | yellow |
| ENSG00000175970 | UNC119B | yellow |
| ENSG00000176083 | ZNF683 | yellow |
| ENSG00000176371 | ZSCAN2 | yellow |
| ENSG00000176407 | KCMF1 | yellow |
| ENSG00000176454 | LPCAT4 | yellow |
| ENSG00000176788 | BASP1 | yellow |
| ENSG00000176915 | ANKLE2 | yellow |
| ENSG00000177084 | POLE | yellow |
| ENSG00000177294 | FBXO39 | yellow |
| ENSG00000177409 | SAMD9L | yellow |
| ENSG00000177663 | IL17RA | yellow |
| ENSG00000177738 | AC025171.1 | yellow |
| ENSG00000177885 | GRB2 | yellow |
| ENSG00000177989 | ODF3B | yellow |
| ENSG00000178015 | GPR150 | yellow |
| ENSG00000178146 | AL672207.1 | yellow |
| ENSG00000178458 | H3F3AP6 | yellow |
| ENSG00000178685 | PARP10 | yellow |
| ENSG00000178726 | THBD | yellow |
| ENSG00000178947 | SMIM10L2A | yellow |
| ENSG00000179044 | EXOC3L1 | yellow |
| ENSG00000179101 | AL590139.1 | yellow |
| ENSG00000180263 | FGD6 | yellow |
| ENSG00000180423 | HARBI1 | yellow |
| ENSG00000180447 | GAS1 | yellow |
| ENSG00000180596 | HIST1H2BC | yellow |

| | | |
|---|---|---|
| ENSG00000180815 | MAP3K15 | yellow |
| ENSG00000180871 | CXCR2 | yellow |
| ENSG00000181026 | AEN | yellow |
| ENSG00000181045 | SLC26A11 | yellow |
| ENSG00000181192 | DHTKD1 | yellow |
| ENSG00000181220 | ZNF746 | yellow |
| ENSG00000181274 | FRAT2 | yellow |
| ENSG00000181381 | DDX60L | yellow |
| ENSG00000181481 | RNF135 | yellow |
| ENSG00000181631 | P2RY13 | yellow |
| ENSG00000181656 | GPR88 | yellow |
| ENSG00000181804 | SLC9A9 | yellow |
| ENSG00000182022 | CHST15 | yellow |
| ENSG00000182054 | IDH2 | yellow |
| ENSG00000182179 | UBA7 | yellow |
| ENSG00000182185 | RAD51B | yellow |
| ENSG00000182197 | EXT1 | yellow |
| ENSG00000182247 | UBE2E2 | yellow |
| ENSG00000182541 | LIMK2 | yellow |
| ENSG00000182782 | HCAR2 | yellow |
| ENSG00000182912 | TSPEAR-AS2 | yellow |
| ENSG00000182944 | EWSR1 | yellow |
| ENSG00000182979 | MTA1 | yellow |
| ENSG00000182985 | CADM1 | yellow |
| ENSG00000183255 | PTTG1IP | yellow |
| ENSG00000183347 | GBP6 | yellow |
| ENSG00000183486 | MX2 | yellow |
| ENSG00000183621 | ZNF438 | yellow |
| ENSG00000183647 | ZNF530 | yellow |
| ENSG00000183726 | TMEM50A | yellow |
| ENSG00000183762 | KREMEN1 | yellow |
| ENSG00000183785 | TUBA8 | yellow |
| ENSG00000184182 | UBE2F | yellow |
| ENSG00000184208 | C22orf46 | yellow |
| ENSG00000184319 | RPL23AP82 | yellow |
| ENSG00000184371 | CSF1 | yellow |
| ENSG00000184557 | SOCS3 | yellow |
| ENSG00000184602 | SNN | yellow |
| ENSG00000184795 | UNC93B5 | yellow |
| ENSG00000184903 | IMMP2L | yellow |
| ENSG00000184979 | USP18 | yellow |
| ENSG00000184988 | TMEM106A | yellow |
| ENSG00000185112 | FAM43A | yellow |
| ENSG00000185215 | TNFAIP2 | yellow |
| ENSG00000185250 | PPIL6 | yellow |
| ENSG00000185271 | KLHL33 | yellow |
| ENSG00000185338 | SOCS1 | yellow |
| ENSG00000185404 | SP140L | yellow |

| | | |
|---|---|---|
| ENSG00000185482 | STAC3 | yellow |
| ENSG00000185507 | IRF7 | yellow |
| ENSG00000185591 | SP1 | yellow |
| ENSG00000185722 | ANKFY1 | yellow |
| ENSG00000185745 | IFIT1 | yellow |
| ENSG00000185880 | TRIM69 | yellow |
| ENSG00000185885 | IFITM1 | yellow |
| ENSG00000185897 | FFAR3 | yellow |
| ENSG00000185905 | C16orf54 | yellow |
| ENSG00000186115 | CYP4F2 | yellow |
| ENSG00000186205 | 1-Mar | yellow |
| ENSG00000186352 | ANKRD37 | yellow |
| ENSG00000186407 | CD300E | yellow |
| ENSG00000186615 | KTN1-AS1 | yellow |
| ENSG00000186806 | VSIG10L | yellow |
| ENSG00000187037 | GPR141 | yellow |
| ENSG00000187554 | TLR5 | yellow |
| ENSG00000187608 | ISG15 | yellow |
| ENSG00000187741 | FANCA | yellow |
| ENSG00000187764 | SEMA4D | yellow |
| ENSG00000187775 | DNAH17 | yellow |
| ENSG00000188015 | S100A3 | yellow |
| ENSG00000188157 | AGRN | yellow |
| ENSG00000188215 | DCUN1D3 | yellow |
| ENSG00000188282 | RUFY4 | yellow |
| ENSG00000188290 | HES4 | yellow |
| ENSG00000188313 | PLSCR1 | yellow |
| ENSG00000188375 | H3F3C | yellow |
| ENSG00000188404 | SELL | yellow |
| ENSG00000188559 | RALGAPA2 | yellow |
| ENSG00000188659 | SAXO2 | yellow |
| ENSG00000188738 | FSIP2 | yellow |
| ENSG00000188820 | FAM26F | yellow |
| ENSG00000188938 | FAM120AOS | yellow |
| ENSG00000189060 | H1F0 | yellow |
| ENSG00000189067 | LITAF | yellow |
| ENSG00000189227 | C15orf61 | yellow |
| ENSG00000189337 | KAZN | yellow |
| ENSG00000196116 | TDRD7 | yellow |
| ENSG00000196123 | KIAA0895L | yellow |
| ENSG00000196141 | SPATS2L | yellow |
| ENSG00000196150 | ZNF250 | yellow |
| ENSG00000196242 | OR2C3 | yellow |
| ENSG00000196284 | SUPT3H | yellow |
| ENSG00000196352 | CD55 | yellow |
| ENSG00000196358 | NTNG2 | yellow |
| ENSG00000196550 | FAM72A | yellow |
| ENSG00000196652 | ZKSCAN5 | yellow |

| | | |
|---|---|---|
| ENSG00000196663 | TECPR2 | yellow |
| ENSG00000196664 | TLR7 | yellow |
| ENSG00000196684 | HSH2D | yellow |
| ENSG00000196743 | GM2A | yellow |
| ENSG00000196778 | OR52K1 | yellow |
| ENSG00000196954 | CASP4 | yellow |
| ENSG00000196975 | ANXA4 | yellow |
| ENSG00000197081 | IGF2R | yellow |
| ENSG00000197165 | SULT1A2 | yellow |
| ENSG00000197181 | PIWIL2 | yellow |
| ENSG00000197301 | AC090673.1 | yellow |
| ENSG00000197405 | C5AR1 | yellow |
| ENSG00000197536 | C5orf56 | yellow |
| ENSG00000197555 | SIPA1L1 | yellow |
| ENSG00000197629 | MPEG1 | yellow |
| ENSG00000197646 | PDCD1LG2 | yellow |
| ENSG00000197798 | FAM118B | yellow |
| ENSG00000197989 | SNHG12 | yellow |
| ENSG00000198019 | FCGR1B | yellow |
| ENSG00000198053 | SIRPA | yellow |
| ENSG00000198131 | ZNF544 | yellow |
| ENSG00000198133 | TMEM229B | yellow |
| ENSG00000198216 | CACNA1E | yellow |
| ENSG00000198355 | PIM3 | yellow |
| ENSG00000198585 | NUDT16 | yellow |
| ENSG00000198734 | F5 | yellow |
| ENSG00000198785 | GRIN3A | yellow |
| ENSG00000198830 | HMGN2 | yellow |
| ENSG00000202533 | Y_RNA | yellow |
| ENSG00000203666 | EFCAB2 | yellow |
| ENSG00000203747 | FCGR3A | yellow |
| ENSG00000203797 | DDO | yellow |
| ENSG00000203999 | LINC01270 | yellow |
| ENSG00000204103 | MAFB | yellow |
| ENSG00000204179 | PTPN20 | yellow |
| ENSG00000204560 | DHX16 | yellow |
| ENSG00000204619 | PPP1R11 | yellow |
| ENSG00000204620 | AC115618.1 | yellow |
| ENSG00000204634 | TBC1D8 | yellow |
| ENSG00000205089 | CCNI2 | yellow |
| ENSG00000205664 | BX890604.1 | yellow |
| ENSG00000205726 | ITSN1 | yellow |
| ENSG00000205837 | LINC00487 | yellow |
| ENSG00000211779 | TRAV5 | yellow |
| ENSG00000211805 | TRAV24 | yellow |
| ENSG00000211812 | TRAV26-2 | yellow |
| ENSG00000211821 | TRDV2 | yellow |
| ENSG00000212283 | SNORD89 | yellow |

| | | |
|---|---|---|
| ENSG00000212807 | OR2A42 | yellow |
| ENSG00000213025 | COX20P1 | yellow |
| ENSG00000213123 | TCTEX1D2 | yellow |
| ENSG00000213337 | ANKRD39 | yellow |
| ENSG00000213492 | NT5C3AP1 | yellow |
| ENSG00000213533 | TMEM110 | yellow |
| ENSG00000213694 | S1PR3 | yellow |
| ENSG00000213722 | DDAH2 | yellow |
| ENSG00000213742 | ZNF337-AS1 | yellow |
| ENSG00000213923 | CSNK1E | yellow |
| ENSG00000213928 | IRF9 | yellow |
| ENSG00000213967 | ZNF726 | yellow |
| ENSG00000214026 | MRPL23 | yellow |
| ENSG00000214711 | CAPN14 | yellow |
| ENSG00000214872 | SMTNL1 | yellow |
| ENSG00000214975 | PPIAP29 | yellow |
| ENSG00000214999 | AC129492.1 | yellow |
| ENSG00000215481 | BCRP3 | yellow |
| ENSG00000215483 | LINC00598 | yellow |
| ENSG00000215533 | LINC00189 | yellow |
| ENSG00000217159 | LARP1P1 | yellow |
| ENSG00000220785 | MTMR9LP | yellow |
| ENSG00000221823 | PPP3R1 | yellow |
| ENSG00000221890 | NPTXR | yellow |
| ENSG00000221914 | PPP2R2A | yellow |
| ENSG00000221955 | SLC12A8 | yellow |
| ENSG00000221963 | APOL6 | yellow |
| ENSG00000223177 | RNA5SP39 | yellow |
| ENSG00000223511 | AL683807.1 | yellow |
| ENSG00000223552 | AC098613.1 | yellow |
| ENSG00000223722 | AC023157.1 | yellow |
| ENSG00000223914 | LINC02471 | yellow |
| ENSG00000223929 | MIR4432HG | yellow |
| ENSG00000223984 | HNRNPRP1 | yellow |
| ENSG00000224152 | AC009506.1 | yellow |
| ENSG00000224959 | AC017002.1 | yellow |
| ENSG00000225075 | AL603832.1 | yellow |
| ENSG00000225101 | OR52K3P | yellow |
| ENSG00000225131 | PSME2P2 | yellow |
| ENSG00000225151 | GOLGA2P7 | yellow |
| ENSG00000225265 | TAF1A-AS1 | yellow |
| ENSG00000225450 | AL021707.1 | yellow |
| ENSG00000225492 | GBP1P1 | yellow |
| ENSG00000225684 | FAM225B | yellow |
| ENSG00000225886 | AL445490.1 | yellow |
| ENSG00000225964 | NRIR | yellow |
| ENSG00000225978 | HAR1A | yellow |
| ENSG00000226155 | AC124944.1 | yellow |

| | | |
|---|---|---|
| ENSG00000226337 | AL353616.2 | yellow |
| ENSG00000226416 | MRPL23-AS1 | yellow |
| ENSG00000226479 | TMEM185B | yellow |
| ENSG00000226738 | U62317.1 | yellow |
| ENSG00000226800 | CACTIN-AS1 | yellow |
| ENSG00000227288 | AL138799.1 | yellow |
| ENSG00000227527 | AC096540.1 | yellow |
| ENSG00000228203 | RNF144A-AS1 | yellow |
| ENSG00000228314 | CYP4F29P | yellow |
| ENSG00000228318 | AP001610.1 | yellow |
| ENSG00000228439 | TSTD3 | yellow |
| ENSG00000228624 | HDAC2-AS2 | yellow |
| ENSG00000228668 | TRGV5P | yellow |
| ENSG00000228794 | LINC01128 | yellow |
| ENSG00000229331 | GK-IT1 | yellow |
| ENSG00000229754 | CXCR2P1 | yellow |
| ENSG00000229894 | GK3P | yellow |
| ENSG00000230091 | TMEM254-AS1 | yellow |
| ENSG00000230149 | AL021707.3 | yellow |
| ENSG00000230191 | AC006970.1 | yellow |
| ENSG00000230266 | XXYLT1-AS2 | yellow |
| ENSG00000230438 | SERPINB9P1 | yellow |
| ENSG00000231233 | CFAP58-AS1 | yellow |
| ENSG00000231365 | AL359915.2 | yellow |
| ENSG00000231528 | FAM225A | yellow |
| ENSG00000231607 | DLEU2 | yellow |
| ENSG00000231621 | AC013264.1 | yellow |
| ENSG00000231711 | LINC00899 | yellow |
| ENSG00000232093 | DCST1-AS1 | yellow |
| ENSG00000232801 | SDCBPP3 | yellow |
| ENSG00000232815 | DUX4L50 | yellow |
| ENSG00000232938 | RPL23AP87 | yellow |
| ENSG00000233029 | AC244453.2 | yellow |
| ENSG00000233030 | AC243772.2 | yellow |
| ENSG00000233077 | LINC01271 | yellow |
| ENSG00000233214 | AC002511.2 | yellow |
| ENSG00000233369 | GTF2IP4 | yellow |
| ENSG00000233392 | AC104809.2 | yellow |
| ENSG00000233586 | AC246785.2 | yellow |
| ENSG00000233672 | RNASEH2B-AS1 | yellow |
| ENSG00000233673 | ANAPC1P1 | yellow |
| ENSG00000233791 | LINC01136 | yellow |
| ENSG00000233822 | HIST1H2BN | yellow |
| ENSG00000233825 | AL391839.2 | yellow |
| ENSG00000234231 | AC093616.1 | yellow |
| ENSG00000234282 | AL109809.2 | yellow |
| ENSG00000234518 | PTGES3P1 | yellow |
| ENSG00000234571 | AC239798.2 | yellow |

| | | |
|---|---|---|
| ENSG00000234745 | HLA-B | yellow |
| ENSG00000234917 | AC098484.2 | yellow |
| ENSG00000235036 | AL035456.1 | yellow |
| ENSG00000235106 | LINC00094 | yellow |
| ENSG00000235655 | H3F3AP4 | yellow |
| ENSG00000235750 | KIAA0040 | yellow |
| ENSG00000236021 | AL359265.3 | yellow |
| ENSG00000236449 | AC010894.3 | yellow |
| ENSG00000236534 | H3F3BP1 | yellow |
| ENSG00000236565 | HNRNPA3P5 | yellow |
| ENSG00000236618 | PITPNA-AS1 | yellow |
| ENSG00000236679 | RPL23AP24 | yellow |
| ENSG00000237190 | CDKN2AIPNL | yellow |
| ENSG00000237436 | AL590128.2 | yellow |
| ENSG00000237604 | AP001056.1 | yellow |
| ENSG00000237977 | EIF4HP2 | yellow |
| ENSG00000238057 | ZEB2-AS1 | yellow |
| ENSG00000238062 | SPATA3-AS1 | yellow |
| ENSG00000238113 | LINC01410 | yellow |
| ENSG00000239557 | AC092045.1 | yellow |
| ENSG00000239713 | APOBEC3G | yellow |
| ENSG00000240204 | SMKR1 | yellow |
| ENSG00000240809 | AC026877.1 | yellow |
| ENSG00000241061 | RPL5P1 | yellow |
| ENSG00000242258 | LINC00996 | yellow |
| ENSG00000242616 | GNG10 | yellow |
| ENSG00000243646 | IL10RB | yellow |
| ENSG00000244063 | AC104653.2 | yellow |
| ENSG00000244482 | LILRA6 | yellow |
| ENSG00000244617 | ASPRV1 | yellow |
| ENSG00000244682 | FCGR2C | yellow |
| ENSG00000245556 | SCAMP1-AS1 | yellow |
| ENSG00000245648 | AC022075.1 | yellow |
| ENSG00000245869 | AP004609.1 | yellow |
| ENSG00000245937 | LINC01184 | yellow |
| ENSG00000246465 | AC138904.1 | yellow |
| ENSG00000246731 | MGC16275 | yellow |
| ENSG00000248049 | UBA6-AS1 | yellow |
| ENSG00000248124 | RRN3P1 | yellow |
| ENSG00000248429 | AC098679.1 | yellow |
| ENSG00000248477 | AC139495.1 | yellow |
| ENSG00000248554 | AC114956.2 | yellow |
| ENSG00000248794 | AC026436.1 | yellow |
| ENSG00000249173 | LINC01093 | yellow |
| ENSG00000249476 | AC008467.1 | yellow |
| ENSG00000249502 | AC006160.1 | yellow |
| ENSG00000250274 | AC034199.1 | yellow |
| ENSG00000250303 | AP002884.3 | yellow |

| | | |
|---|---|---|
| ENSG00000250415 | AC022113.2 | yellow |
| ENSG00000250539 | KRT8P33 | yellow |
| ENSG00000250616 | AC012645.1 | yellow |
| ENSG00000250771 | AC106865.1 | yellow |
| ENSG00000251301 | LINC02384 | yellow |
| ENSG00000251333 | RTN3P1 | yellow |
| ENSG00000251580 | LINC02482 | yellow |
| ENSG00000253214 | AC079209.1 | yellow |
| ENSG00000253409 | TRBV7-4 | yellow |
| ENSG00000253958 | CLDN23 | yellow |
| ENSG00000254288 | AC087672.3 | yellow |
| ENSG00000254470 | AP5B1 | yellow |
| ENSG00000254503 | AC010319.1 | yellow |
| ENSG00000254554 | AC080023.1 | yellow |
| ENSG00000254612 | AP001000.1 | yellow |
| ENSG00000254691 | AP002812.3 | yellow |
| ENSG00000254851 | AP005018.2 | yellow |
| ENSG00000255221 | CARD17 | yellow |
| ENSG00000255337 | AP001830.1 | yellow |
| ENSG00000255398 | HCAR3 | yellow |
| ENSG00000255449 | AP002812.5 | yellow |
| ENSG00000255717 | SNHG1 | yellow |
| ENSG00000256576 | LINC02361 | yellow |
| ENSG00000256581 | NLRP9P1 | yellow |
| ENSG00000256590 | TRDV3 | yellow |
| ENSG00000256937 | KRT17P8 | yellow |
| ENSG00000257599 | OVCH1-AS1 | yellow |
| ENSG00000257715 | AC007298.1 | yellow |
| ENSG00000257773 | AC011595.2 | yellow |
| ENSG00000258429 | PDF | yellow |
| ENSG00000258521 | AL157871.2 | yellow |
| ENSG00000258581 | AL157871.3 | yellow |
| ENSG00000258655 | ARHGAP5-AS1 | yellow |
| ENSG00000259088 | AL137779.2 | yellow |
| ENSG00000259144 | RANBP20P | yellow |
| ENSG00000259182 | AC019254.1 | yellow |
| ENSG00000259321 | AL136295.2 | yellow |
| ENSG00000259768 | AC004943.2 | yellow |
| ENSG00000260806 | AL163051.1 | yellow |
| ENSG00000260927 | AC009107.2 | yellow |
| ENSG00000260943 | LINC02555 | yellow |
| ENSG00000261269 | AC093278.2 | yellow |
| ENSG00000261428 | AC097461.1 | yellow |
| ENSG00000261438 | AL157394.1 | yellow |
| ENSG00000261644 | AC007728.2 | yellow |
| ENSG00000261659 | Z92544.2 | yellow |
| ENSG00000262636 | AC099489.2 | yellow |
| ENSG00000262823 | AC127521.1 | yellow |

| | | |
|---|---|---|
| ENSG00000263766 | AC025682.1 | yellow |
| ENSG00000264522 | OTUD7B | yellow |
| ENSG00000265415 | AC099850.3 | yellow |
| ENSG00000265458 | AC132938.3 | yellow |
| ENSG00000265531 | FCGR1CP | yellow |
| ENSG00000266017 | MIR4477B | yellow |
| ENSG00000266028 | SRGAP2 | yellow |
| ENSG00000266604 | LINC01887 | yellow |
| ENSG00000266643 | MIR3677 | yellow |
| ENSG00000266750 | MIR4645 | yellow |
| ENSG00000266786 | LGALS9DP | yellow |
| ENSG00000267383 | AC011447.3 | yellow |
| ENSG00000267387 | AC020931.1 | yellow |
| ENSG00000267547 | AC060766.4 | yellow |
| ENSG00000267711 | AC060766.6 | yellow |
| ENSG00000267733 | AP005264.6 | yellow |
| ENSG00000269119 | HNRNPA1P52 | yellow |
| ENSG00000269392 | AC008655.2 | yellow |
| ENSG00000269720 | CCDC194 | yellow |
| ENSG00000269743 | SLC25A53 | yellow |
| ENSG00000270332 | SMC2-AS1 | yellow |
| ENSG00000270426 | AC099343.2 | yellow |
| ENSG00000271304 | AL133507.1 | yellow |
| ENSG00000271425 | NBPF10 | yellow |
| ENSG00000271605 | MILR1 | yellow |
| ENSG00000271855 | AC073195.1 | yellow |
| ENSG00000271964 | AC090948.1 | yellow |
| ENSG00000272109 | AC008906.2 | yellow |
| ENSG00000272334 | AC011816.2 | yellow |
| ENSG00000272462 | U91328.2 | yellow |
| ENSG00000272491 | AL109659.2 | yellow |
| ENSG00000272512 | AL645608.8 | yellow |
| ENSG00000272564 | AC012511.1 | yellow |
| ENSG00000272599 | AC016394.1 | yellow |
| ENSG00000272666 | U62317.2 | yellow |
| ENSG00000272669 | AL021707.6 | yellow |
| ENSG00000272821 | U62317.3 | yellow |
| ENSG00000272941 | AC083862.2 | yellow |
| ENSG00000272954 | AP000553.1 | yellow |
| ENSG00000273265 | AC092636.1 | yellow |
| ENSG00000273314 | AC005229.4 | yellow |
| ENSG00000273356 | LINC02019 | yellow |
| ENSG00000274536 | AL034397.3 | yellow |
| ENSG00000274752 | AC244196.1 | yellow |
| ENSG00000275106 | AC025594.3 | yellow |
| ENSG00000275464 | FP565260.1 | yellow |
| ENSG00000275791 | TRBV10-3 | yellow |
| ENSG00000275895 | U2AF1L5 | yellow |

| | | |
|---|---|---|
| ENSG00000275898 | AC125603.4 | yellow |
| ENSG00000276116 | FUT8-AS1 | yellow |
| ENSG00000276317 | AL357033.3 | yellow |
| ENSG00000276390 | AC004241.3 | yellow |
| ENSG00000277053 | GTF2IP1 | yellow |
| ENSG00000277511 | AC116407.2 | yellow |
| ENSG00000277701 | AC159540.2 | yellow |
| ENSG00000278989 | AP001148.1 | yellow |
| ENSG00000279296 | PRAL | yellow |
| ENSG00000279320 | AC069528.2 | yellow |
| ENSG00000279453 | Z99129.4 | yellow |
| ENSG00000279522 | AC008536.3 | yellow |
| ENSG00000279727 | LINC02033 | yellow |
| ENSG00000279861 | AC073548.1 | yellow |
| ENSG00000279884 | AC010894.5 | yellow |
| ENSG00000280088 | AC126474.2 | yellow |
| ENSG00000280411 | AC245369.3 | yellow |
| ENSG00000281100 | AC105749.1 | yellow |
| ENSG00000282164 | PEG13 | yellow |
| ENSG00000282851 | BISPR | yellow |
| ENSG00000283031 | AC009242.1 | yellow |
| ENSG00000283384 | AL138694.1 | yellow |
| ENSG00000283486 | FAM95C | yellow |
| ENSG00000284642 | AL139424.2 | yellow |
